# Differentially Expressed Genes in Head and Neck Squamous Cell Carcinoma: Exploratory Research Using the Cancer Genome Atlas (TCGA) RNA Sequence Data and DESeq2 Package

**DOI:** 10.7759/cureus.94537

**Published:** 2025-10-14

**Authors:** Naoki Katase, Yae Sakamoto, Hiroki Suda, Rin Miyahara, Shuichi Fujita

**Affiliations:** 1 Department of Oral Pathology, Nagasaki University, Graduate School of Biomedical Sciences, Nagasaki, JPN; 2 Dental School, Nagasaki University, Nagasaki, JPN

**Keywords:** bioinformatics, deseq2, head and neck squamous cell carcinoma, squamous cell carcinoma, tcga database

## Abstract

Introduction

Head and neck squamous cell carcinoma (HNSCC) is the most common cancer of the head and neck region, including the oral cavity, larynx, pharynx, nasal cavity, and paranasal sinuses. Cancer arises because of cumulative genetic and epigenetic alterations in cancer-associated genes. It is important to understand the genetic/epigenetic background of the tumors to establish molecular targeted therapies. So far, the knowledge of key genes or molecules, which are closely associated with the carcinogenesis and development of HNSCC, is insufficient for targeted therapies. On the other hand, recent advances in next-generation sequencing (NGS) have greatly contributed to cancer genome research. In this research, using RNA sequence data of HNSCC stored in The Cancer Genome Atlas (TCGA) database, we identified differentially expressed genes (DEGs), functionally enriched gene sets, and new prognostic markers or candidate therapeutic targets. This exploratory study investigated whether novel prognostic markers and candidate therapeutic targets for HNSCC could be identified from TCGA RNA-seq data.

Methods

The RNA sequence data were downloaded from TCGA, including 504 cases from cancer and 44 cases from corresponding normal tissue. The DEGs between cancer and normal samples were detected using the DESeq2 package in R software. Differences with | log2 fold change (FC) | > 1.0 and p-value <0.05 were considered as DEGs. Functional enrichment analyses were performed by ShinyGO 0.85 with Gene Ontology (GO) terms and Kyoto Encyclopedia of Genes and Genomes (KEGG) pathways. A gene set enrichment analysis (GSEA) was also performed using GSEA software. We also analyzed the top 10 up- and down-regulated genes, which were sorted by adjusted p-value, by using Kaplan-Meier analysis to assess their potential as prognostic markers.

Results

Using the DESeq2 package, 10,976 DEGs were detected, including 6,932 up-regulated genes and 4,044 down-regulated genes in cancer. As expected, functional enrichment analyses revealed enrichment of KEGG terms associated with cancers, including “Pathway in Cancer”, “Human Papillomavirus infection”, and “PI3K-Akt signaling pathway” in up-regulated genes, whereas KEGG terms enriched in down-regulated genes were mainly “Metabolic pathways”. GO terms for “Cell differentiation (GOBP)” and “Extracellular region (GOCC)” were enriched both in up- and down-regulated genes, suggesting aberrant expression of genes associated with cell differentiation and remodeling of the extracellular matrix. GSEA data supported the enrichment analyses data. Kaplan-Meier analyses revealed that high expression of homeobox C6 (*HOXC6*) (p=0.048), nucleobindin 2 (*NUCB2*) (p=0.007), IL12A antisense RNA 1 (*IL12A-AS1*) (p=0.001), calcium-binding protein 39-like (*CAB39L*)(p=0.038)*, *nitric oxide synthase traffickin*g* (*NOSTRIN*) (p=0.024), SLC8A1 antisense RNA 1 (*SLC8A1-AS1*) (p=0.016), were the significantly correlated with poorer prognosis.

Conclusions

Based on bioinformatical approaches, we identified significantly enriched gene sets and novel candidates for prognostic markers or therapeutic targets in HNSCC. Further investigation would aid in determining the anti-cancer effects of these candidates.

## Introduction

Head and neck squamous cell carcinoma (HNSCC) is the sixth or seventh most common cancer worldwide, which is reported to account for 6% of all malignant tumors [1-3]. The incidence of HNSCC has been increasing in recent years, with 660,000 new cases and 325,000 deaths per year [1,2,4]. In general, genetic and epigenetic alterations in cancer-associated genes accumulate as carcinogenesis steps progress, and differences in these abnormal profiles cause intra- and inter-tumoral heterogeneity. Therefore, it is important to understand the underlying genetic/epigenetic profiles of the cancer-associated genes for the practice of precision medicine and development of molecular targeted therapies. Recent developments in next-generation sequencing (NGS) methods and increased utilization of public cancer genome databases such as The Cancer Genome Atlas (TCGA) are contributing to this purpose [5,6].

The TCGA consortium published a comprehensive genetic characterization of HNSCC in 2015, where helical domain mutations of the oncogene *PIK3CA* in human papilloma virus-related HNSCC, and loss-of-function *TP53* mutations and *CDKN2A* inactivation in smoking-related HNSCC are demonstrated [7]. Other reports also showed that genetic alterations such as *TP53* mutation, loss of *CDKN2A*, amplification of *CCND1,* and *PI3KCA* are commonly observed in HNSCC [8,9]. These data may indicate therapeutic candidate alterations in HNSCC; nevertheless, these results have not yet been put into practical use in precision medicine because these candidates are common and not specific.

Research using the TCGA database often analyzes predefined target genes and examines consistency with *in vitro* and *in vivo* findings. However, in this research, we aimed to extract differentially expressed genes (DEGs) between HNSCC tumor tissue and corresponding normal tissue in the TCGA database (TCGA-HNSC) as exploratory or nascent research and attempted data mining for novel prognostic predictors and therapeutic target candidates. We extracted DEGs using R software and the DESeq2 package and analyzed them by enrichment analyses and gene set enrichment analysis (GSEA) to grasp the biological differences between normal and HNSCC tissue. Moreover, we tried to characterize the top 10 up- and down-regulated DEGs using Kaplan-Meier analysis.

## Materials and methods

Data acquisition

The RNA sequence data from HNSCC patients (TCGA-HNSC) were downloaded from the TCGA data portal (https://portal.gdc.cancer.gov/) in June 2025. In total, 504 tumor samples and 44 normal samples were included in the cohort. The overall survival (months) of the samples was 30.01 ±28.95. The raw data of RNA sequence data (counts) were extracted, and a data table was created using R (version 4.2.3) [10], and clinical data corresponding to the TCGA-HNSC cohort were downloaded from cBioportal (https://www.cbioportal.org/) [11,12]. Because TCGA is an open public database, obtaining relevant information does not require additional ethical approval. In this research, subgroups (such as human papillomavirus-related or smoking-related) were not set because the lack of information in many cases raised concerns that the sample size might be insufficient for subclassification. Then we attempted to identify the universal therapeutic targets that may be expressed in cancer, independent of these subtypes. The gene name and Entrez gene ID corresponding to ENSEMBL were obtained using the R language-related package bioMart (version 2.52.0) [13,14].

Analysis of DEGs between the cancer and normal tissues

Gene expression profiles of the cancer tissues and normal tissues were compared using R version 4.2.3. The DEGs were extracted using the R language-related package DESEq2 (version 1.36.0) [15]. Differences with | log2 fold change (FC)| >1.0 and p-value <0.05 were considered as DEGs. Volcano plot was generated using EnhancedVolcano (version 1.14.0) [16] and ggplot2 package (version 3.4.1) [17]. Enrichment analysis was also performed using ShinyGO 0.85 [18]. Enriched processes or pathways were determined with the criteria of false discovery rate (FDR) <0.05, and a pathway size between 2-2000, and the top 10 enriched processes or pathways were shown sorted by genes.

We also included the whole data of the DEGs data as supplementary materials so that one can trace the experimental procedures and perform further investigations.

Gene set enrichment analysis (GSEA)

GSEA was used to assess the concordance between our data set and a priori-defined gene set, which was performed using GSEA software version 4.2.1 (https://www.gsea-msigdb.org/) [19]. A function or pathway term with FDR q-val (FDR) <0.25 or nominal p-value (NOM p-val) <0.01 was considered significant. The gene sets database used for GSEA with Gene Ontology (GO) biological process (GOBP), GO cellular component (GOCC), GO molecular function (GOMF), and KEGG pathways were “c5.go.bp.v2025.1.Hs.symbols.gmt, c5.go.cc.v2025.1.Hs.symbols.gmt”, “c5.go.mf.v2025.1.Hs.symbols.gmt” and “c2.cp.kegg_legacy.v2025.1.Hs.symbols.gmt”, respectively. The number of permutations was 1000, and the chip platform used was “Human_Gene_Symbol_with_Remapping_MSigDB.v2025.1.Hs.chip”.

Kaplan-Meier analysis

Kaplan-Meier analysis was performed to determine the survival analysis. The log-rank test was used to compare survival between the high and low groups. The average TPM value in the tumor tissue is set as a cutoff value determining high or low expression. Overall survival (OS) in months was calculated from the day after surgery to the last follow-up appointment. Logistic regression analysis was used for univariate and multivariate analysis.

Statistical analysis

All values are presented as the means ± standard deviation (SD). Significant differences in gene expression between normal and tumor samples were determined using Student's t-test. All analyses were conducted using R version 4.5.1 (The R Foundation for Statistical Computing, Vienna, Austria; http://www.r-project.org/). A p-value <0.05 was considered statistically significant.

## Results

Extraction of DEGs using the DESEq2 package

**Figure 1:**
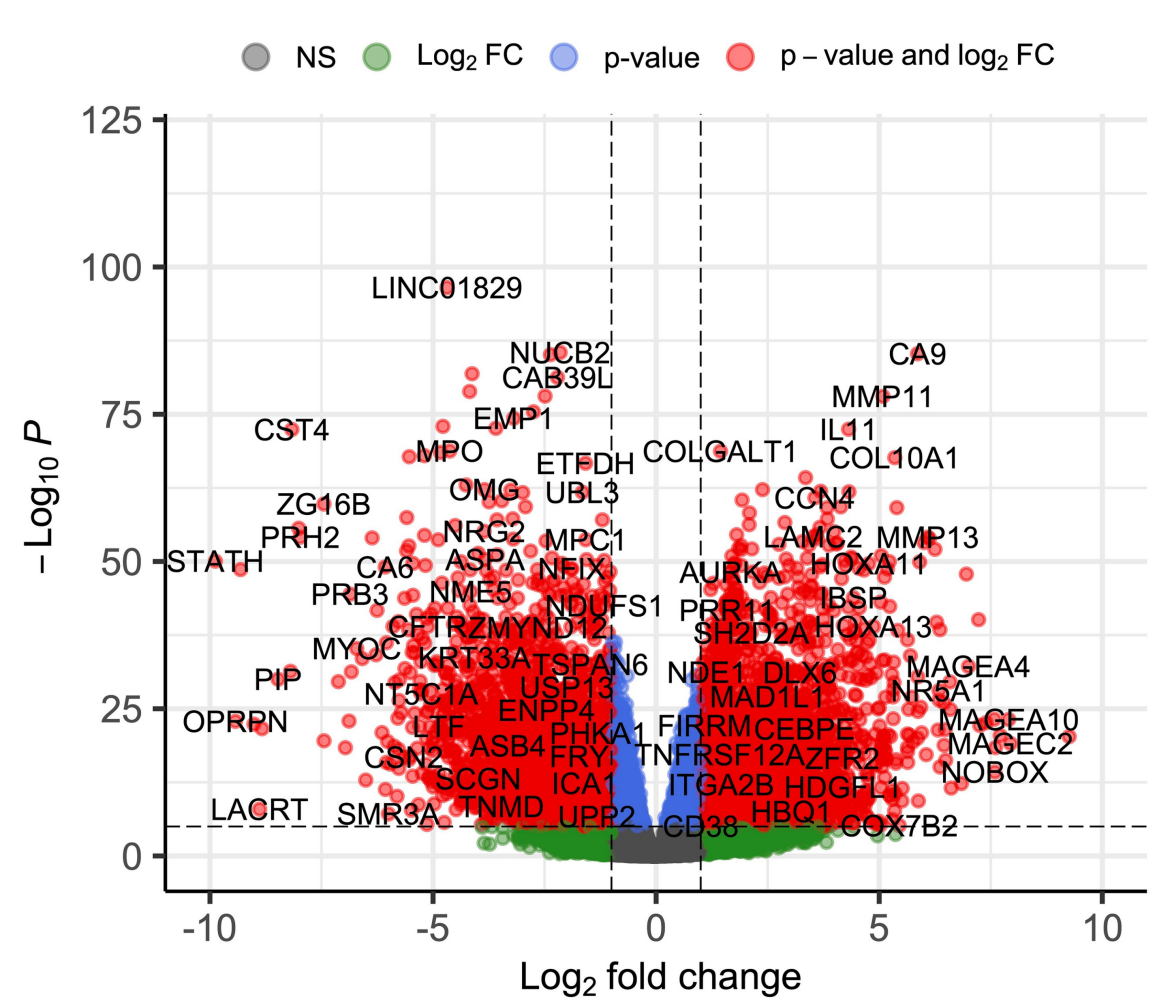
Volcano plot for DEGs The relationship between DEG expression ratio and statistical significance (p-value) is shown in the plot. Not significant genes (NS) are indicated as gray dots. Genes with | log_2_ FC | >1 but p-value >0.05, and genes with | log_2_ FC | <1 but p-value <0.05 are indicated as green dots and blue dots, respectively. Genes with | log_2_ FC | >1 and p-value <0.05 are labeled as red dots with gene names DEG: differentially expressed gene; FC: fold change

Using the R package DESeq2, we identified 10,976 DEGs in total; 6,932 up-regulated genes and 4,044 down-regulated genes in cancer tissue compared with normal tissue. The details of the top 10 DEGs (up-regulated and down-regulated), sorted by adjusted p-value (padj) with ENSEMBL ID, gene name, Entrez ID, log FC, and p-value, are shown in Tables 1, 2, respectively. The original data, including 6.932 up-regulated genes and 4.044 down-regulated genes, are shown in the Appendix. The relationship between DEG expression ratio and statistical significance (p-value) is shown in a volcano plot (Figure 1).

**Table 1:** Top 10 up-regulated DEGs DEG: differentially expressed gene

No.	ENSEMBL	Gene name	Entrez gene_id	log_2 _fold change	P-value	padj
1	ENSG00000107159.13	CA9	768	5.859026736	5.35E-86	7.08E-82
2	ENSG00000099953.10	MMP11	4320	5.084702846	9.29E-79	4.09E-75
3	ENSG00000095752.7	IL11	3589	4.313230325	3.51E-73	9.94E-70
4	ENSG00000130309.11	COLGALT1	79709	1.442430407	1.94E-69	4.52E-66
5	ENSG00000123500.10	COL10A1	1300	5.348843778	2.35E-68	4.44E-65
6	ENSG00000248554.1	C5orf34-AS1		3.348878001	5.73E-65	9.09E-62
7	ENSG00000237424.1	FOXD2-AS1	84793	2.384643413	6.25E-63	8.91E-60
8	ENSG00000197757.8	HOXC6	3223	3.687591199	1.20E-62	1.58E-59
9	ENSG00000261327.5	AC134312.5	105371401	4.321038861	1.47E-62	1.88E-59
10	ENSG00000104415.14	CCN4	8840	3.557303974	1.54E-61	1.79E-58

**Table 2:** Top 10 down-regulated DEGs DEG: differentially expressed gene

No.	ENSEMBL	Gene name	Entrez gene_id	Log_2 _fold change	P-value	padj
1	ENSG00000236780.7	LINC01829	644838	-4.687076089	3.15E-97	1.25E-92
2	ENSG00000070081.17	NUCB2	4925	-2.151594889	3.16E-86	6.27E-82
3	ENSG00000106351.13	AGFG2	3268	-2.36880628	7.60E-86	7.53E-82
4	ENSG00000244040.7	IL12A-AS1	101928376	-4.125123063	1.33E-82	1.06E-78
5	ENSG00000102547.19	CAB39L	81617	-2.211503108	5.52E-82	3.65E-78
6	ENSG00000151882.11	CCL28	56477	-4.180018322	1.36E-79	7.72E-76
7	ENSG00000163072.16	NOSTRIN	115677	-2.49002706	8.36E-79	4.09E-75
8	ENSG00000152642.11	GPD1L	23171	-2.750437316	3.83E-76	1.52E-72
9	ENSG00000134531.10	EMP1	2012	-3.192275656	4.94E-75	1.78E-71
10	ENSG00000227028.6	SLC8A1-AS1		-4.777969597	1.14E-73	3.76E-70

Enrichment analysis of DEGs

We also performed enrichment analysis using ShinyGO 0.85 with GO terms and KEGG pathways. As for GO biological process (GOBP), genes related to “Cell differentiation” (UP: FDR=1.91E-23, fold enrichment=1.414, DOWN: FDR=4.58E-10, fold enrichment=1.266) and “Cellular developmental process” (UP: FDR=1.91E-23, fold enrichment=1.414, DOWN: FDR=4.69E-10, fold enrichment=1.266) are enriched both in up- and down-regulated DEGs.

Results for GO cellular component (GOCC) showed that genes related to “Extracellular region” (UP: FDR=6.25E-18, fold enrichment=1.359, DOWN: FDR=5.67E-26, fold enrichment=1.437), “Extracellular space” (UP: FDR=4.49E-11, fold enrichment=1.329, DOWN: FDR=2.72E-20, fold enrichment=1.451) were enriched both in up- and down-regulated DEGs.

GO molecular function (GOMF) showed that “DNA binding” (FDR=7.12E-12, fold enrichment=1.412) and “Signaling receptor binding” (FDR=6.18E-14, fold enrichment=1.579) were significantly enriched in up-regulated DEGs, whereas “Transporter activity” (FDR=8.04E-17, fold enrichment=1.750) was significantly enriched in down-regulated DEGs.

**Figure 2:**
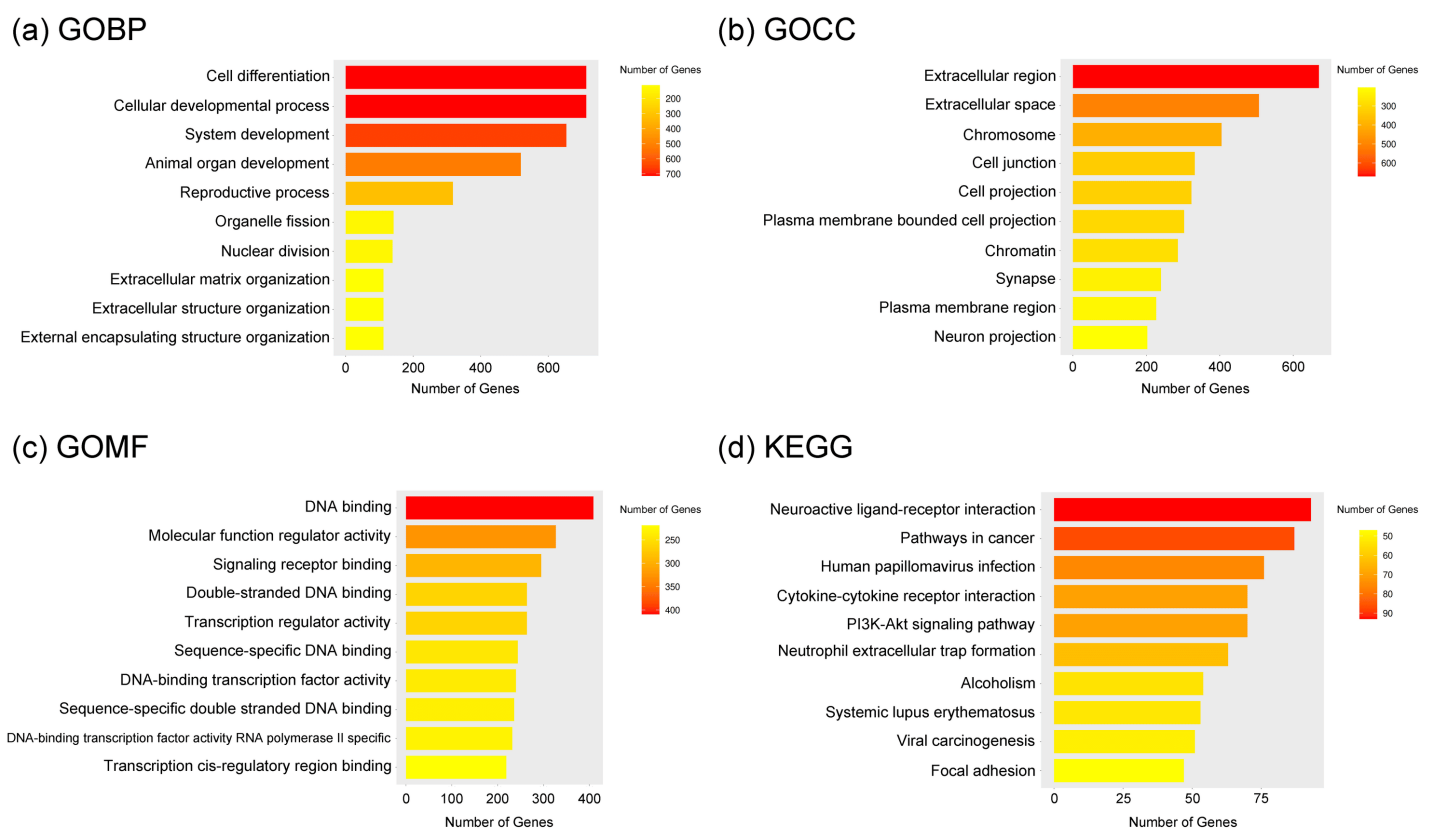
Enrichment analyses data with ShinyGO 0.85 (a) Enriched GOBP terms for up-regulated DEGs were cell differentiation (FDR=1.91E-23), cellular developmental process (FDR=1.91E-23), system development (FDR=1.68E-23), animal organ development (FDR=8.75E-23), reproductive process (FDR=8.18E-23), organelle fission (FDR=6.76E-22), nuclear division (FDR=5.06E-24), extracellular matrix organization (FDR=1.53E-23), extracellular structure organization (FDR=1.53E-23), and external encapsulating structure organization (FDR=1.68E-23) (b) Enriched GOCC terms for up-regulated DEGs were extracellular region (FDR=6.25E-18), extracellular space (FDR=4.49E-11), chromosome (FDR=5.19E-31), cell junction (FDR=1.55E-03), cell projection (FDR=4.78E-03), plasma membrane-bounded cell projection (FDR=1.53E-02), chromatin (FDR=1.35E-21), synapse (FDR=6.74E-04), plasma membrane region (FDR=2.04E-09), and neuron projection (FDR=9.02E-05) (c) Enriched GOMF terms for up-regulated DEGs were DNA binding (FDR=7.12E-12), molecular function regulator activity (FDR=1.38E-04), signaling receptor binding (FDR=6.18E-14), double-stranded DNA binding (FDR=5.24E-07), transcription regulator activity (FDR=2.58E-02), sequence-specific DNA binding (FDR=3.77E-04), DNA-binding transcription factor activity (FDR=1.00E-07), sequence-specific double-stranded DNA binding (FDR=6.09E-05), DNA-binding transcription factor activity rna polymerase II-specific (FDR=1.64E-08), and transcription cis-regulatory region binding (FDR=1.18E-03) (d) Enriched KEGG pathways for up-regulated DEGs were neuroactive ligand-receptor interaction (FDR=9.04E-14), pathways in cancer (FDR=4.06E-04), human papillomavirus infection (FDR=2.36E-09), cytokine-cytokine receptor interaction (FDR=2.99E-09), PI3K-Akt signaling pathway (FDR=1.10E-05), neutrophil extracellular trap formation (FDR=4.73E-15), alcoholism (FDR=2.44E-10), systemic lupus erythematosus (FDR=2.18E-15), viral carcinogenesis (FDR=1.27E-07), and focal adhesion (FDR=4.18E-06)

**Figure 3:**
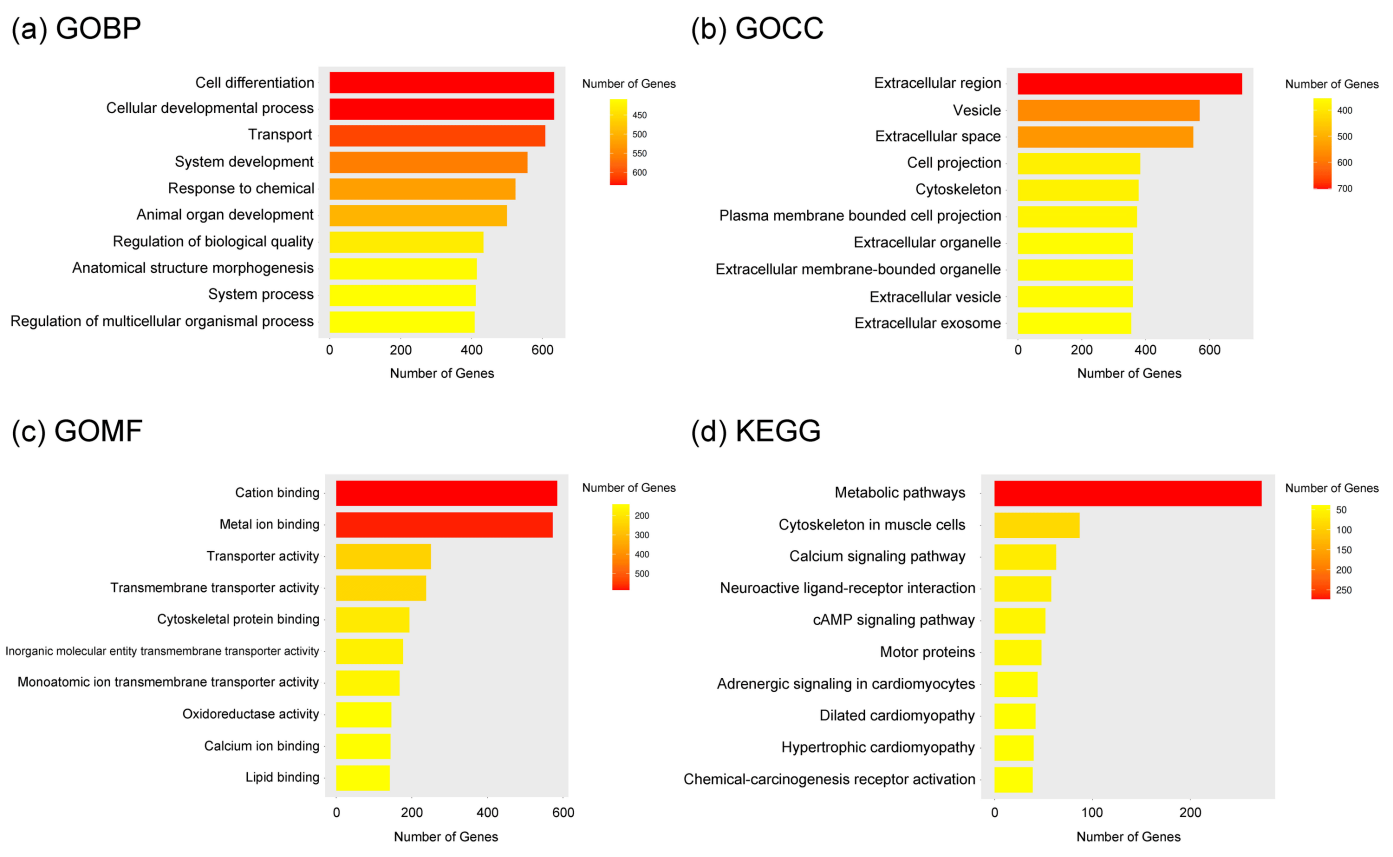
Enrichment analyses data with ShinyGO 0.85 (a) Enriched GOBP terms for gown-regulated DEGs were cell differentiation (FDR=4.58E-10), cellular developmental process (FDR=4.69E-10), transport (FDR=1.24E-06), system development (FDR=1.55E-07), response to chemical (FDR=8.36E-03), animal organ development (FDR=3.29E-19), regulation of biological quality (FDR=5.80E-08), anatomical structure morphogenesis (FDR=5.23E-11), system process (FDR=7.00E-18), and regulation of multicellular organismal process (FDR=7.00E-18) (b) Enriched GOCC terms for down-regulated DEGs were extracellular region (FDR=5.67E-26), vesicle (FDR=1.05E-05), extracellular space (FDR=2.72E-20), cell projection (FDR=1.89E-12), plasma membrane-bounded cell projection (FDR=2.37E-13), extracellular organelle (FDR=1.75E-12), extracellular membrane-bounded organelle (FDR=1.75E-12), extracellular vesicle (FDR=1.75E-12), and extracellular exosome (FDR=2.38E-12) (c) Enriched GOMF terms for down-regulated DEGs were cation binding (FDR=4.99E-03), metal ion binding (FDR=5.52E-03), transporter activity (FDR=8.04E-17), transmembrane transporter activity (FDR=5.20E-18), cytoskeletal protein binding (FDR=1.89E-13), inorganic molecular entity transmembrane transporter activity (FDR=1.02E-18), monoatomic ion transmembrane transporter activity (FDR=1.62E-14), oxidoreductase activity (FDR=9.31E-09), calcium ion binding (FDR=1.90E-08), and lipid binding (FDR=2.12E-05) (d) Enriched KEGG pathways for down-regulated DEGs were metabolic pathways (FDR=1.71E-16), cytoskeleton in muscle cells (FDR=2.13E-25), calcium signaling pathway (FDR=1.51E-09), neuroactive ligand-receptor interaction (FDR=7.48E-03), cAMP signaling pathway (FDR=7.91E-07), motor proteins (FDR=2.49E-07), adrenergic signaling in cardiomyocytes (FDR=8.41E-09), dilated cardiomyopathy (FDR=2.55E-13), hypertrophic cardiomyopathy (FDR=5.88E-13), and chemical carcinogenesis-receptor activation (FDR=5.88E-13)

Enrichment analysis with KEGG pathway showed that cancer-associated terms, including “Pathways in Cancer” (FDR=4.06E-04, fold enrichment=1.560), “PI3K-Akt signaling pathway” (FDR=1.10E-05, fold enrichment=1.834), and “Focal adhesion” (FDR=4.18E-06, fold enrichment=2.207) were significantly enriched in up-regulated DEGs. On the other hand, KEGG terms associated with “Metabolic pathways” (FDR=1.71E-16, fold enrichment=1.679) were significantly enriched in down-regulated DEGs (Figures 2, 3). All the test statistic values, including ones that were not mentioned above, are shown in the figure legends.

Gene set enrichment analysis

**Figure 4:**
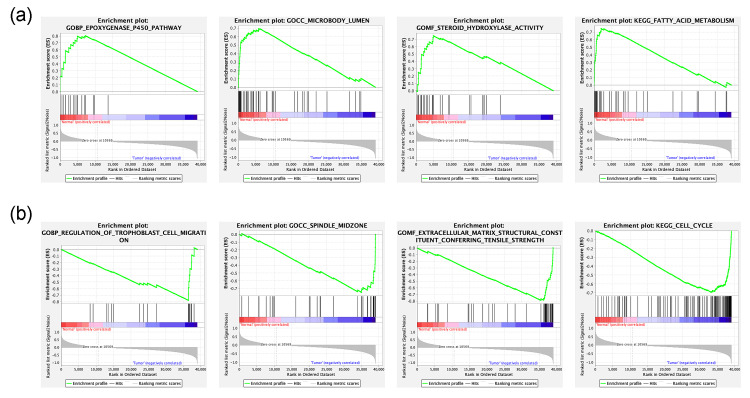
Gene set enrichment analysis (a) Top enriched GO term or KEGG pathways in Normal tissue are “GOBP_EPOXYGENASE_P450_PATHWAY” (NES=2.17, FDR q-val=0.011), “GOCC_MICROBODY_LUMEN” (NES=2.13, FDR q-val=0.004), “GOMF_STEROID_HYDROXYLASE_ACTIVITY” (NES=2.39, FDR q-val <0.001), and “KEGG_FATTY_ACID_METABOLISM” (NES=2.14, FDR q-val=0.003) (b) Top enriched GO term or KEGG pathways in tumor tissue are “GOBP_REGULATION_OF_TROPHOBLAST_CELL_MIGRATION” (NES=-2.15, FDR q-val=0.009), “GOCC_SPINDLE_MIDZONE” (NES=-2.05, FDR q-val=0.005), “GOMF_EXTRACELLULAR_MATRIX_STRUCTURAL_CONSTITUENT_CONFERRING_TENSILE_STRENGTH” (NES=-1.99, FDR q-val=0.002), and “KEGG_CELL_CYCLE” (NES=-1.99, FDR q-val=0.026)

GSEA was used to identify GO terms (GOBP, GOCC, and GOMF) and KEGG pathways associated with HNSCC between the Normal and Tumor groups. In the normal group. 1,038/4,209 gene sets (GOBP), 120/513 gene sets (GOCC), 254/850 gene sets (GOMF), and 64/178 gene sets (KEGG) were up-regulated. In the tumor group, 3,171/4,209 gene sets (GOBP), 393/513 gene sets (GOCC), 596/850 gene sets (GOMF), and 114/178 gene sets (KEGG) were up-regulated. Figure 4 shows the most significantly enriched GO terms and KEGG pathways. “GOBP_EPOXYGENASE_P450_PATHWAY” (NES=2.17, FDR q-val=0.011), “GOCC_MICROBODY_LUMEN”, (NES=2.13, FDR q-val=0.004), “GOMF_STEROID_HYDROXYLASE_ACTIVITY” (NES=2.39, FDR q-val.<0.001), and “KEGG_FATTY_ACID_METABOLISM” (NES=2.14, FDR q-val=0.003) were significantly enriched in Normal tissue, whereas “GOBP_REGULATION_OF_TROPHOBLAST_CELL_MIGRATION” (NES=-2.15, FDR q-val=0.009), “GOCC_SPINDLE_MIDZONE” (NES=-2.05, FDR q-val=0.005), “GOMF_EXTRACELLULAR_MATRIX_STRUCTURAL_CONSTITUENT_CONFERRING_TENSILE_STRENGTH” (NES=-1.99, FDR q-val=0.002), and “KEGG_CELL_CYCLE” (NES=-1.99, FDR q-val=0.026) were significantly enriched in Tumor tissue.

Expression and Kaplan-Meier analyses of Top 10 DEGs

After checking the enriched GO terms or KEGG pathways, we focused on the individual genes and investigated their potential as prognostic markers or novel candidates for therapeutic targets. Since there are too many genes listed as DEGs, we chose the top 10 up- and down-regulated genes by sorting the DEGs by padj. We confirmed that all the top 10 up- and down-regulated genes were expressed at a constant level that is not too low, both in normal (N=44) and tumor (N=504).

**Figure 5:**
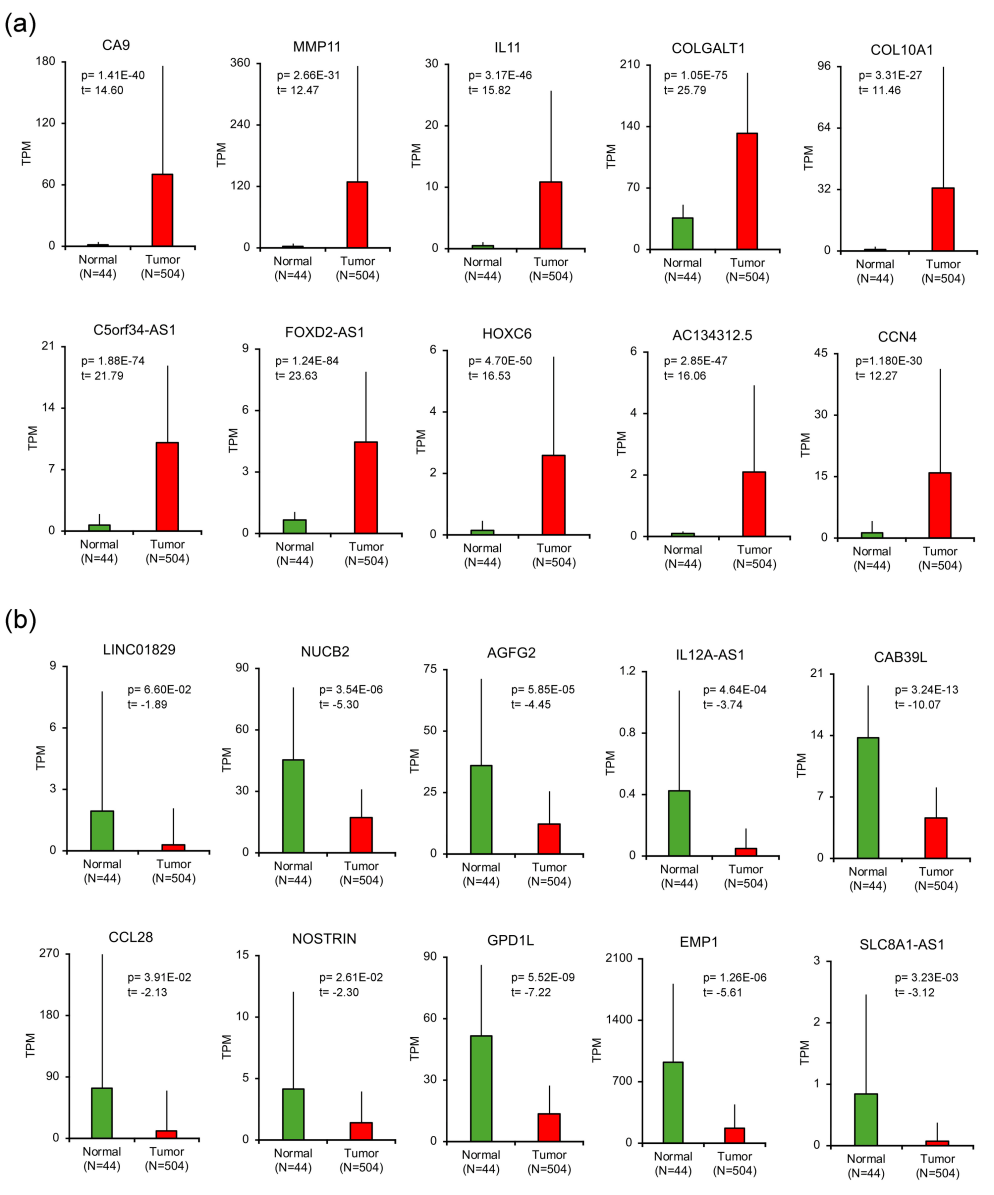
Expression changes of the DEGs Expression changes of the DEGs. (a) All up-regulated DEGs showed significantly higher expression in tumor samples (N=504) than in normal samples (N=44). (b) All down-regulated DEGs showed significantly lower expression in tumor samples (N=504) than in normal samples (N=44) DEG: differentially expressed gene

All the up-regulated genes showed significantly higher expression in tumor sample than those in normal samples: carbonic anhydrase 9 (*CA9*) (p=1.41E-40, t=14.60), matrix metalloproteinase-11 (*MMP11*) (p=2.66E-31, t=12.47), interleukin11 (*IL11*) (p=3.17E-46, t=15.82), collagen beta (1-O)galactosyltransferase 1 (*COLGALT1*) (p=1.05E-75, t=25.79), collagen type X alpha 1 chain (*COL10A1*) (p=3.31E-27, t=11.46), C5orf34 antisense RNA 1 (*C5orf34-AS1A5*) (p=1.88E-74, t=21.79), FOXD2 adjacent opposite strand RNA 1 (*FOXD2-AS1*) (p=1.24E-84, t=23.63), homeobox C6 (*HOXC6*) (p=4.70E-50, t=16.53), AC134312.5 (p=2.85E-47, t=16.06), and cellular communication network factor 4 (*CCN4*) (p=1.18E-30, t=12.27) (Figure 5a), and all the down-regulated genes showed significantly lower expression in tumor sample than those in normal samples: long intergenic non-protein coding RNA 1829 (*LINC01829*) (p=6.60E-02, t=-1.89), nucleobindin2 (*NUCB2*) (p=3.54E-06, t=-5.30) , ArfGAP with FG repeats 2 (*AGFG2*) (p=5.85E-05, t=-4.45), IL12A antisense RNA 1 (*IL12A-AS1*) (p=4.64E-04, t=-3.74), calcium-binding protein 39-like (*CAB39L*) (p=3.24E-13, t=-10.07), C-C motif chemokine ligand 28 (*CCL28*) (p=3.91E-02, t=-2.13), nitric oxide synthase trafficking (*NOSTRIN*) (p=2.61E-02, t=-2.30), glycerol-3-phosphate dehydrogenase 1 like (*GPD1L*) (p=5.46E-09, t=-7.22), epithelial membrane protein 1 (*EMP1*) (p=1.26E-06, t=-5.61), and SLC8A1 antisense RNA 1 (*SLC8A1-AS1*) (p=3.23E-03, t=-3.12) (Figure 5b).

**Figure 6:**
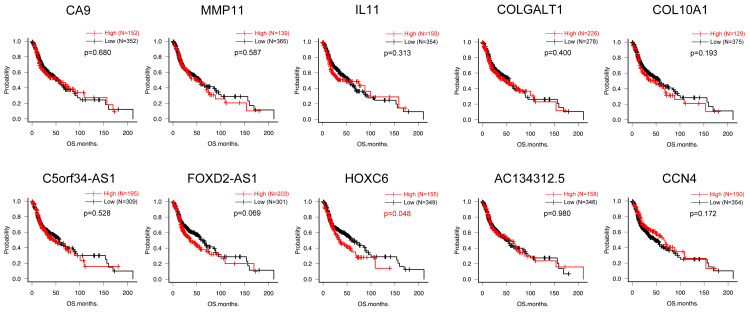
Kaplan-Meier analysis for top 10 up-regulated DEGs Kaplan-Meier analyses revealed that high expression of *HOXC6* (high=155, low=349, p=0.048) was significantly correlated with poorer prognosis DEG: differentially expressed gene

**Figure 7:**
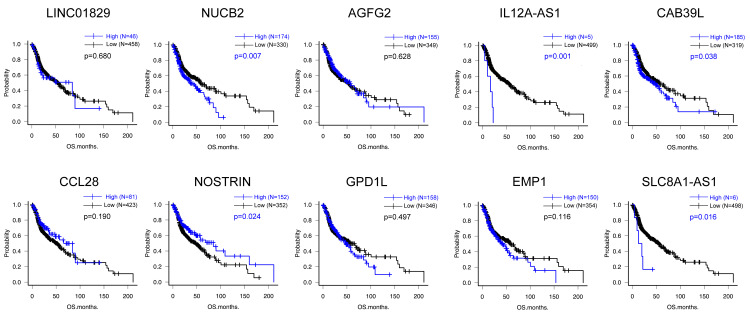
Kaplan-Meier analysis for top 10 down-regulated DEGs Kaplan-Meier analyses revealed that high expression of *NUCB2 *(high=174, low=330, p=0.007), *IL12A-AS1* (high=5, low=499, p=0.001), *CAB39L* (high=185, low=319, p=0.038), and *SLC8A1-AS1* (high=6, low=498, p=0.016) were the significantly correlated with poorer prognosis, whereas low expression of *NOSTRIN* (high=152, low=352, p=0.024) was significantly correlated with poorer prognosis DEG: differentially expressed gene

Kaplan-Meier analyses revealed that high expression of *HOXC6* (high=155, low=349, p=0.048), *NUCB2* (high=174, low=330, p=0.007), *IL12A-AS1* (high=5, low=499, p=0.001), *CAB39L* (high=185, low=319, p=0.038) and *SLC8A1-AS1 *(high=6, low=498, p=0.016) were the significantly correlated with poorer prognosis, whereas low expression of *NOSTRIN* (high=152, low=352, p=0.024) was significantly correlated with poorer prognosis (Figures 6, 7).

## Discussion

The NGS technology has revolutionized the ability to sequence millions of DNA fragments at once and provides detailed information about genetic variations and changes in gene function; it has rapidly advanced genomics and the understanding of diseases, and has a major impact on precision medicine [20,21]. Developments in NGS technology have enabled reading a full diploid human genome sequence in just a few days at low cost. Nowadays, NGS has also been utilized in the medical field for diagnosis and decision-making for therapeutic approaches. For example, by analyzing cancer cell genes using NGS to identify the genetic mutations causing the cancer, and then using molecularly targeted drugs designed to be effective against those specific genetic mutations. This approach is known as precision medicine.

Along with the development of NGS, the establishment of public databases that store RNA sequence data, such as Surveillance, Epidemiology, and End Results (SEER), National Health and Nutrition Examination Survey (NHANES), TCGA, and Medical Information Mart for Intensive Care (MIMIC), has also witnessed significant progress. Using these databases and data mining methods, studies leading to the elucidation of disease mechanisms have been emerging [22]. HNSCC is one of the cancers with a high incidence and high mortality rate worldwide. HNSCC arises in the oral cavity, pharynx, and larynx with distinct risk factors; oral cavity and larynx cancers are generally associated with tobacco and/or alcohol consumption, whereas pharynx cancers are increasingly attributed to human papillomavirus (HPV) infection [1]. Therefore, HNSCC shows highly heterogeneous clinical manifestations, tumor progression or metastasis pattern, resistance or sensitivity to the treatment. This may explain why few attempts have led to the development of actual treatments despite many previous studies showing cancer-associated genes and pathways.

In this research, we attempted to identify new, universal prognostic or therapeutic candidate genes for HNSCC, regardless of the location of occurrence or risk factors. Previous bioinformatics studies have often followed a context where confirming the function of known genes through *in silico* analysis after functional analyses *in vitro* and *in vivo* have been done. However, this study was designed as an exploratory investigation aiming to identify novel prognostic factors and potential therapeutic targets by comparing tumor groups with normal groups. To our knowledge, it is a pioneering study with no comparable research to date.

Using RNA-sequence data stored in TCGA and the DESeq2 package of R software, we extracted DEGs between normal and cancer tissues. DESeq2 is a representative R package that estimates the dependence between variance and mean in count data obtained from high-throughput sequencing analysis and tests differential expression using a model based on the negative binomial distribution [23]. Using DESeq2, we could efficiently extract reliable DEGs from RNA-seq data, with 6,932 up-regulated genes and 4,044 down-regulated genes. Enrichment analyses revealed that both up- and down-regulated DEGs were enriched in “Cell differentiation”. This finding may reflect the aberration of cell differentiation in the cancer cells, such as dedifferentiation or abnormal maturation. As for GO cellular component (GOCC), “Extracellular region” and “Extracellular space” were enriched both in up- and down-regulated DEGs. It is considered that this finding may reflect the remodeling of extracellular matrices in the cancer microenvironment. The up-regulation of *MMP11*, *COLGALT1*, and *COL10A1* may support this hypothesis. Alternatively, data derived from the stroma within the tumor tissue may be mixed in, but there may be limitations to the analysis when using this RNA-seq data.

Enrichment analysis using KEGG pathway showed that up-regulated DEGs were significantly enriched in cancer-associated signals such as “Pathways in cancer” and “PI3K-Akt signaling”, which was considered reasonable; however, this merely captures the “phenomenon resulting from cancer”. Enrichment analysis is defined as an analysis that determines whether the proportion of genes with specific functions (GO terms) among DEGs is statistically significantly higher. We need to note that enrichment analysis methods and statistical approaches are limited, and that the identification of functions does not necessarily indicate causality. A drawback of DEG enrichment analysis is that results vary depending on the threshold, making it difficult to detect pathways with weak overall expression fluctuations. GSEA analysis treats all genes as expression change rankings, enabling the detection of pathways with mild fluctuations. However, it does not necessarily detect more significant pathways than DEG enrichment. GSEA enables interpretation of biological meaning at the gene set level, even for “subtle expression variations” that are difficult to detect in individual gene expression differences, thereby facilitating a more comprehensive understanding of molecular mechanisms. However, the results in this study did not illustrate a specific enrichment signature of HNSCC.

To derive some new insights from this analysis, we validated the top 10 up- and down-regulated DEGs with Kaplan-Meier analysis. We set the high/low cutoff value at the average TPM value. The data illustrated that high expression of *HOXC6*, *NUCB2*, *IL12A-AS1*, *CAB39L*, and S*LC8A1-AS1* or low expression of *NOSTRIN *were associated with poorer prognosis. Recently, *HOXC6* has been reported to directly activate oncogenes enolase 2 (ENO2) and activate cancer cell aggressiveness, orchestrating the Warburg effect in oral squamous cell carcinoma cells [24]. It is reported that high NUCB2 expression is correlated with poorer prognosis in nasopharyngeal carcinoma, a type of HNSCC [25]. Long non-cpding RNAs (lncRNAs) are known to be involved in the onset of cancer and various diseases, and are attracting attention as new biomarkers and therapeutic targets and some reports showed its potential use of diagnostic or prognostic marker, including *IL12A-AS1* or *SLC8A1-AS1* candidate of key lncRNA [26, 27], although the biological role of these lncRNA in HNSCC has not been investigated yet. *CAB39L* plays an anti-tumor role in many cancers, such as breast cancer and gastric cancer, and is reported to be down-regulated and possesses diagnostic and prognostic values in several types of cancer. *CAB39L* encodes a scaffold protein that can interact with STE20-related adaptor alpha (STRADA) pseudokinase to activate liver kinase B1 (LKB1) tumor suppressive kinase activity [28]. However, to the best of our knowledge, the involvement of *CAB39L* in HNSCC or the function of *NOSTRIN* has not been reported yet.

Further investigations clarifying the detailed biological roles of these molecules are required. Nevertheless, we could identify novel candidates for prognostic markers or therapeutic targets from the vast and elusive big data.

Study limitations

The cohort we used was from the TCGA database, but the case number of normal samples (N=44) was low compared with cancer samples (N=504). It is important to note that we did not perform analyses subdividing cases into subgroups based on factors such as sex, age, or HPV infection status. This decision was driven by the presence of numerous missing data points, which could have led to insufficient case numbers in any subgroups, and by our intention to identify gene sets considered universally important regardless of such subgrouping.

Whether the candidate genes identified in this research are truly associated with the nature of HNSCC, carcinogenesis, progression, and prognosis should be validated in further investigation, including immunohistochemical analyses using HNSCC tissue samples and in vitro and in vivo assays using HNSCC-derived cell lines. The objective of this study is to determine whether novel targets can be extracted from the TCGA database; elucidating their function is considered a separate challenge. Therefore, while we have provided literature-based explanations of the significance of the identified genes in this study, we believe that essential functional analysis should be addressed in a different study. Also, a new analytical algorithm is needed to identify truly meaningful gene expression variations from DEGs data. To contribute to this goal, we attached the DEGs list and GSEA data obtained by the research as supplementary materials.

## Conclusions

Using the TCGA database and the DESeq2 packages in R software, we were able to identify DEGs in the tumor tissue of HNSCC. The results of enrichment analyses were difficult to interpret and were still ambiguous. Kaplan-Meier analysis with the top 10 DEGs could suggest candidates for novel prognostic markers of HNSCC. Further investigations are required to clarify the detailed biological roles of these molecules and the possibility of finding novel therapeutic targets.

## Appendices

**Table 3:** Raw data of Up-regulated DEGs

	ENSEMBL	GENENAME	entrezgene_id	log2FoldChange	p-value	padj
1	ENSG00000107159.13	CA9	768	5.859026736	5.35E-86	7.08E-82
2	ENSG00000099953.10	MMP11	4320	5.084702846	9.29E-79	4.09E-75
3	ENSG00000095752.7	IL11	3589	4.313230325	3.51E-73	9.94E-70
4	ENSG00000130309.11	COLGALT1	79709	1.442430407	1.94E-69	4.52E-66
5	ENSG00000123500.10	COL10A1	1300	5.348843778	2.35E-68	4.44E-65
6	ENSG00000248554.1	C5orf34-AS1		3.348878001	5.73E-65	9.09E-62
7	ENSG00000237424.1	FOXD2-AS1	84793	2.384643413	6.25E-63	8.91E-60
8	ENSG00000197757.8	HOXC6	3223	3.687591199	1.20E-62	1.58E-59
9	ENSG00000261327.5		105371401	4.321038861	1.47E-62	1.88E-59
10	ENSG00000104415.14	CCN4	8840	3.557303974	1.54E-61	1.79E-58
11	ENSG00000146670.10	CDCA5	113130	1.926272281	3.71E-61	4.20E-58
12	ENSG00000267123.7	SCAT1	101928710	4.136053949	5.30E-60	5.25E-57
13	ENSG00000228509.6			5.393485764	7.24E-60	7.00E-57
14	ENSG00000105464.3	GRIN2D	2906	3.831142148	7.43E-60	7.02E-57
15	ENSG00000169258.7	GPRIN1	114787	2.091233765	5.57E-59	5.14E-56
16	ENSG00000250133.2	HOXC-AS2	100874364	3.836898626	5.78E-58	4.98E-55
17	ENSG00000117122.14	MFAP2	4237	2.883863763	2.42E-57	1.96E-54
18	ENSG00000101057.16	MYBL2	4605	2.084944403	5.62E-57	4.46E-54
19	ENSG00000180806.5	HOXC9	3225	3.67057782	1.59E-56	1.21E-53
20	ENSG00000122641.11	INHBA	3624	3.981498939	8.61E-56	6.21E-53
21	ENSG00000142945.13	KIF2C	11004	1.804932343	2.85E-55	2.00E-52
22	ENSG00000253293.5	HOXA10	3206	3.596802365	2.88E-55	2.00E-52
23	ENSG00000058085.15	LAMC2	3918	3.522490432	6.11E-55	4.11E-52
24	ENSG00000137745.12	MMP13	4322	6.085598348	7.45E-55	4.84E-52
25	ENSG00000170689.10	HOXB9	3219	6.12405757	1.24E-54	7.79E-52
26	ENSG00000128710.6	HOXD10	3236	3.610527071	1.30E-54	8.04E-52
27	ENSG00000117407.17	ARTN	9048	3.276822242	4.14E-54	2.38E-51
28	ENSG00000159217.10	IGF2BP1	10642	6.003739928	6.05E-54	3.42E-51
29	ENSG00000128713.14	HOXD11	3237	4.058875314	7.10E-54	3.96E-51
30	ENSG00000037965.6	HOXC8	3224	4.038021449	1.09E-53	5.98E-51
31	ENSG00000182963.11	GJC1	10052	2.753605729	1.24E-53	6.73E-51
32	ENSG00000100985.7	MMP9	4318	3.873703147	1.65E-53	8.76E-51
33	ENSG00000248240.1			3.426560231	1.66E-53	8.76E-51
34	ENSG00000154920.15	EME1	146956	1.903655676	1.97E-53	1.03E-50
35	ENSG00000092853.14	CLSPN	63967	1.847050298	5.68E-53	2.89E-50
36	ENSG00000025423.11	HSD17B6	8630	2.145704837	7.31E-53	3.67E-50
37	ENSG00000272620.2	AFAP1-AS1	84740	6.248643839	8.34E-53	4.13E-50
38	ENSG00000171208.10	NETO2	81831	1.898134774	1.01E-52	4.94E-50
39	ENSG00000238125.1	SLC9A3P2		5.045429459	1.02E-51	4.72E-49
40	ENSG00000127423.11	AUNIP	79000	1.696428247	1.16E-51	5.27E-49
41	ENSG00000149948.14	HMGA2	8091	4.368807871	1.77E-51	7.96E-49
42	ENSG00000134668.12	SPOCD1	90853	3.385532901	2.67E-51	1.18E-48
43	ENSG00000170965.10	PLAC1	10761	4.388110155	3.36E-51	1.47E-48
44	ENSG00000134013.16	LOXL2	4017	2.91383705	3.72E-51	1.60E-48
45	ENSG00000234041.1			4.23069876	6.68E-51	2.76E-48
46	ENSG00000286042.1	LCAL1	80078	5.225682838	1.07E-50	4.28E-48
47	ENSG00000230838.1	LINC01614	105373869	5.906177056	1.21E-50	4.75E-48
48	ENSG00000115163.15	CENPA	1058	1.764437821	1.62E-50	6.28E-48
49	ENSG00000005073.6	HOXA11	3207	4.76436737	2.38E-50	8.98E-48
50	ENSG00000094804.12	CDC6	990	1.751333013	3.28E-50	1.22E-47
51	ENSG00000262406.3	MMP12	4321	3.726447242	3.29E-50	1.22E-47
52	ENSG00000127564.17	PKMYT1	9088	1.908255101	4.62E-50	1.68E-47
53	ENSG00000203805.11	PLPP4	196051	4.245769303	4.87E-50	1.76E-47
54	ENSG00000183682.8	BMP8A	353500	2.981242995	4.98E-50	1.78E-47
55	ENSG00000178150.10	ZNF114	163071	3.671809556	8.18E-50	2.84E-47
56	ENSG00000196611.5	MMP1	4312	4.543487034	1.42E-49	4.85E-47
57	ENSG00000011332.19	DPF1	8193	3.157150791	1.45E-49	4.93E-47
58	ENSG00000267013.6	LINC01929	101927229	5.183652037	1.82E-49	6.06E-47
59	ENSG00000089116.4	LHX5	64211	4.659275139	2.89E-49	9.26E-47
60	ENSG00000090889.12	KIF4A	24137	1.664815949	5.66E-49	1.75E-46
61	ENSG00000149257.16	SERPINH1	871	2.165600066	6.01E-49	1.85E-46
62	ENSG00000087586.18	AURKA	6790	1.676465652	6.36E-49	1.94E-46
63	ENSG00000187498.16	COL4A1	1282	2.428711938	8.97E-49	2.71E-46
64	ENSG00000104327.7	CALB1	793	6.952781853	1.37E-48	4.11E-46
65	ENSG00000148848.14	ADAM12	8038	3.483834515	1.81E-48	5.39E-46
66	ENSG00000287839.1			2.487359402	2.53E-48	7.50E-46
67	ENSG00000214544.7	GTF2IRD2P1		2.882564784	3.97E-48	1.16E-45
68	ENSG00000228203.7	GRASLND	386597	3.182250634	4.33E-48	1.25E-45
69	ENSG00000184502.4	GAST	2520	5.118182498	4.61E-48	1.33E-45
70	ENSG00000088325.16	TPX2	22974	1.722563016	6.63E-48	1.87E-45
71	ENSG00000257906.3	LINC02156		2.751425238	6.72E-48	1.88E-45
72	ENSG00000251003.9	ZFPM2-AS1		4.155298265	8.22E-48	2.28E-45
73	ENSG00000174371.17	EXO1	9156	1.714065876	1.32E-47	3.59E-45
74	ENSG00000088305.18	DNMT3B	1789	2.319223281	3.62E-47	9.44E-45
75	ENSG00000138092.11	CENPO	79172	1.278876495	4.93E-47	1.26E-44
76	ENSG00000105329.11	TGFB1	7040	1.576056751	8.86E-47	2.22E-44
77	ENSG00000043355.12	ZIC2	7546	3.46118459	1.30E-46	3.23E-44
78	ENSG00000086717.18	PPEF1	5475	3.496052403	1.86E-46	4.59E-44
79	ENSG00000258711.2			3.191549682	2.32E-46	5.68E-44
80	ENSG00000138180.16	CEP55	55165	1.765178526	2.77E-46	6.68E-44
81	ENSG00000103855.18	CD276	80381	1.798779663	4.24E-46	1.01E-43
82	ENSG00000146143.19	PRIM2	5558	1.221359173	5.80E-46	1.38E-43
83	ENSG00000163568.15	AIM2	9447	3.316696689	9.87E-46	2.28E-43
84	ENSG00000187608.10	ISG15	9636	3.723046374	2.37E-45	5.40E-43
85	ENSG00000278966.2			3.429911072	2.40E-45	5.43E-43
86	ENSG00000163507.14	CIP2A	57650	1.677114986	2.43E-45	5.48E-43
87	ENSG00000175063.17	UBE2C	11065	1.798673862	2.92E-45	6.54E-43
88	ENSG00000120708.17	TGFBI	7045	3.232959098	3.25E-45	7.19E-43
89	ENSG00000113739.10	STC2	8614	2.947406179	3.49E-45	7.68E-43
90	ENSG00000213468.7	FIRRE	286467	3.685080777	4.22E-45	9.10E-43
91	ENSG00000093009.11	CDC45	8318	1.797945636	6.93E-45	1.45E-42
92	ENSG00000143476.18	DTL	51514	1.744598122	7.39E-45	1.54E-42
93	ENSG00000162062.15	TEDC2	80178	1.910601151	1.04E-44	2.15E-42
94	ENSG00000133466.14	C1QTNF6	114904	2.573435239	1.37E-44	2.77E-42
95	ENSG00000029559.7	IBSP	3381	4.420463991	1.37E-44	2.77E-42
96	ENSG00000186185.14	KIF18B	146909	1.699454042	1.40E-44	2.81E-42
97	ENSG00000167900.12	TK1	7083	1.787088498	2.40E-44	4.74E-42
98	ENSG00000166851.15	PLK1	5347	1.592716241	3.65E-44	7.15E-42
99	ENSG00000168487.20	BMP1	649	1.679769109	3.97E-44	7.71E-42
100	ENSG00000260027.5	HOXB7	3217	3.459326341	5.09E-44	9.84E-42
101	ENSG00000076382.17	SPAG5	10615	1.56333094	7.27E-44	1.39E-41
102	ENSG00000272763.1	LINC03057	127379715	5.031133778	7.87E-44	1.50E-41
103	ENSG00000105376.5	ICAM5	7087	2.822460099	1.10E-43	2.08E-41
104	ENSG00000163918.11	RFC4	5984	1.787715032	1.53E-43	2.86E-41
105	ENSG00000118193.12	KIF14	9928	1.846449609	1.71E-43	3.19E-41
106	ENSG00000172167.8	MTBP	27085	1.548603522	2.04E-43	3.78E-41
107	ENSG00000180340.7	FZD2	2535	2.034323545	2.06E-43	3.80E-41
108	ENSG00000091651.9	ORC6	23594	1.686340793	2.07E-43	3.81E-41
109	ENSG00000172156.4	CCL11	6356	3.873052815	2.58E-43	4.67E-41
110	ENSG00000111206.13	FOXM1	2305	1.849668165	3.91E-43	6.95E-41
111	ENSG00000114270.17	COL7A1	1294	2.192000116	4.21E-43	7.46E-41
112	ENSG00000122133.17	PAEP	5047	5.236322919	4.26E-43	7.50E-41
113	ENSG00000241749.4	RPSAP52		4.309167685	5.64E-43	9.89E-41
114	ENSG00000112655.16	PTK7	5754	1.561207456	5.94E-43	1.04E-40
115	ENSG00000111665.12	CDCA3	83461	1.719645089	7.04E-43	1.22E-40
116	ENSG00000233532.6	LINC00460	728192	4.553225191	7.14E-43	1.24E-40
117	ENSG00000123388.4	HOXC11	3227	3.731913472	1.04E-42	1.78E-40
118	ENSG00000285517.1	LINC00941	645485	3.200367641	1.10E-42	1.87E-40
119	ENSG00000122861.16	PLAU	5328	2.445229583	1.19E-42	2.02E-40
120	ENSG00000068489.13	PRR11	55771	1.590730412	1.85E-42	3.09E-40
121	ENSG00000228742.11	LINC02577	111216280	3.983635598	2.12E-42	3.50E-40
122	ENSG00000075218.19	GTSE1	51512	1.588612734	2.98E-42	4.89E-40
123	ENSG00000163888.4	CAMK2N2	94032	2.516371347	3.57E-42	5.82E-40
124	ENSG00000139880.19	CDH24	64403	1.654981749	3.87E-42	6.26E-40
125	ENSG00000086300.16	SNX10	29887	2.016251441	4.03E-42	6.50E-40
126	ENSG00000272666.1	KLHDC7B-DT		3.030209198	4.14E-42	6.65E-40
127	ENSG00000141391.14	PRELID3A	10650	1.884426411	5.53E-42	8.80E-40
128	ENSG00000196584.3	XRCC2	7516	1.753653475	6.25E-42	9.91E-40
129	ENSG00000162849.16	KIF26B	55083	2.554917337	6.50E-42	1.03E-39
130	ENSG00000136295.15	TTYH3	80727	1.743389079	7.83E-42	1.23E-39
131	ENSG00000111674.9	ENO2	2026	2.592106713	1.51E-41	2.34E-39
132	ENSG00000225697.13	SLC26A6	65010	1.379051651	1.54E-41	2.36E-39
133	ENSG00000123485.12	HJURP	55355	1.685847685	1.69E-41	2.58E-39
134	ENSG00000151388.11	ADAMTS12	81792	3.21736047	2.64E-41	4.00E-39
135	ENSG00000089685.15	BIRC5	332	1.692079013	2.66E-41	4.00E-39
136	ENSG00000139800.8	ZIC5	85416	3.751861416	5.80E-41	8.58E-39
137	ENSG00000175352.11	NRIP3	56675	2.563716359	7.25E-41	1.06E-38
138	ENSG00000257636.6	G2E3-AS1		7.226861976	7.51E-41	1.10E-38
139	ENSG00000196739.15	COL27A1	85301	2.03368069	7.78E-41	1.13E-38
140	ENSG00000181355.21	OFCC1		4.471553415	8.59E-41	1.24E-38
141	ENSG00000119547.6	ONECUT2	9480	3.466028985	1.57E-40	2.24E-38
142	ENSG00000087494.16	PTHLH	5744	3.398932867	1.57E-40	2.24E-38
143	ENSG00000249550.7	LINC01234	100506465	6.287214197	2.00E-40	2.82E-38
144	ENSG00000135451.13	TROAP	10024	1.703449766	2.06E-40	2.90E-38
145	ENSG00000160293.17	VAV2	7410	1.690657799	2.16E-40	3.03E-38
146	ENSG00000101189.7	MRGBP	55257	1.235184923	4.39E-40	6.00E-38
147	ENSG00000204262.14	COL5A2	1290	2.995896242	4.83E-40	6.56E-38
148	ENSG00000206195.11	DUXAP8	503637	3.508149242	4.94E-40	6.69E-38
149	ENSG00000111799.21	COL12A1	1303	2.766117717	6.51E-40	8.72E-38
150	ENSG00000168505.7	GBX2	2637	4.194096347	6.69E-40	8.93E-38
151	ENSG00000171241.9	SHCBP1	79801	1.484565847	6.85E-40	9.11E-38
152	ENSG00000179528.16	LBX2	85474	2.439962849	7.41E-40	9.82E-38
153	ENSG00000106031.9	HOXA13	3209	4.869648046	1.11E-39	1.45E-37
154	ENSG00000034053.14	APBA2	321	2.564211987	1.13E-39	1.46E-37
155	ENSG00000168005.9	SPINDOC	144097	1.378768278	1.24E-39	1.61E-37
156	ENSG00000146535.14	GNA12	2768	1.354609415	1.32E-39	1.69E-37
157	ENSG00000106397.12	PLOD3	8985	1.685481862	1.34E-39	1.71E-37
158	ENSG00000127928.13	GNGT1	2792	3.471648218	1.48E-39	1.89E-37
159	ENSG00000102384.13	CENPI	2491	1.565406586	1.50E-39	1.91E-37
160	ENSG00000224594.2	RPL29P19		3.801721967	2.53E-39	3.14E-37
161	ENSG00000134871.19	COL4A2	1284	2.047850632	2.73E-39	3.37E-37
162	ENSG00000203740.4	NTMT2	149281	4.682829133	2.80E-39	3.44E-37
163	ENSG00000171310.11	CHST11	50515	1.98950377	3.54E-39	4.32E-37
164	ENSG00000163577.8	EIF5A2	56648	1.800008484	3.57E-39	4.34E-37
165	ENSG00000146410.12	MTFR2	113115	1.564817584	3.60E-39	4.37E-37
166	ENSG00000250874.1			6.360768749	3.80E-39	4.59E-37
167	ENSG00000071539.14	TRIP13	9319	1.702422365	3.86E-39	4.64E-37
168	ENSG00000226363.3	HAGLROS		3.336920873	4.86E-39	5.77E-37
169	ENSG00000173157.17	ADAMTS20	80070	5.417096081	4.95E-39	5.86E-37
170	ENSG00000160949.17	TONSL	4796	1.539642807	5.25E-39	6.19E-37
171	ENSG00000164400.6	CSF2	1437	4.443348344	5.50E-39	6.48E-37
172	ENSG00000168067.12	MAP4K2	5871	1.264685252	6.86E-39	7.99E-37
173	ENSG00000060718.22	COL11A1	1301	4.459876037	1.20E-38	1.39E-36
174	ENSG00000027869.12	SH2D2A	9047	2.131116931	1.30E-38	1.49E-36
175	ENSG00000229647.2	MYOSLID		3.105454243	1.32E-38	1.52E-36
176	ENSG00000130829.18	DUSP9	1852	3.186159531	1.34E-38	1.53E-36
177	ENSG00000280832.1	GSEC		2.070279704	1.80E-38	2.05E-36
178	ENSG00000197467.16	COL13A1	1305	2.057744863	1.84E-38	2.09E-36
179	ENSG00000065308.5	TRAM2	9697	1.568884581	2.25E-38	2.53E-36
180	ENSG00000163872.16	YEATS2	55689	1.290161764	2.68E-38	2.99E-36
181	ENSG00000162892.16	IL24	11009	4.276317376	2.80E-38	3.11E-36
182	ENSG00000085999.13	RAD54L	8438	1.531709878	2.87E-38	3.19E-36
183	ENSG00000130635.16	COL5A1	1289	2.959173964	3.04E-38	3.36E-36
184	ENSG00000256128.6	LINC00944	100996671	3.051974598	3.86E-38	4.24E-36
185	ENSG00000121152.10	NCAPH	23397	1.468646377	3.89E-38	4.26E-36
186	ENSG00000087116.16	ADAMTS2	9509	2.806864134	4.35E-38	4.76E-36
187	ENSG00000145681.11	HAPLN1	1404	2.893890213	4.67E-38	5.08E-36
188	ENSG00000051180.17	RAD51	5888	1.454672995	4.79E-38	5.18E-36
189	ENSG00000178999.13	AURKB	9212	1.788578382	5.57E-38	6.00E-36
190	ENSG00000198554.12	WDHD1	11169	1.374907374	7.79E-38	8.26E-36
191	ENSG00000235897.1	TM4SF19-AS1	100874214	2.833338072	1.28E-37	1.34E-35
192	ENSG00000181544.15	FANCB	2187	1.490094155	1.48E-37	1.55E-35
193	ENSG00000223553.6	SMPD4P1		4.346903668	1.55E-37	1.62E-35
194	ENSG00000137807.16	KIF23	9493	1.399214049	1.59E-37	1.65E-35
195	ENSG00000244306.11	DUXAP10		3.422549265	1.68E-37	1.74E-35
196	ENSG00000157456.8	CCNB2	9133	1.460336331	1.70E-37	1.76E-35
197	ENSG00000185686.18	PRAME	23532	5.634108709	2.00E-37	2.06E-35
198	ENSG00000126709.15	IFI6	2537	3.056466138	2.02E-37	2.07E-35
199	ENSG00000235385.2	LINC02154	109729169	4.775641235	2.16E-37	2.21E-35
200	ENSG00000128714.6	HOXD13	3239	4.490530078	2.98E-37	3.03E-35
201	ENSG00000138829.12	FBN2	2201	3.791390474	3.45E-37	3.50E-35
202	ENSG00000218358.2	RAET1K		3.614216882	3.52E-37	3.55E-35
203	ENSG00000160326.14	SLC2A6	11182	2.072330273	4.12E-37	4.13E-35
204	ENSG00000251365.4	LINC02236	109729133	3.410970937	4.37E-37	4.36E-35
205	ENSG00000110203.9	FOLR3	2352	4.728592544	6.60E-37	6.51E-35
206	ENSG00000188153.14	COL4A5	1287	2.088623634	6.65E-37	6.54E-35
207	ENSG00000165891.16	E2F7	144455	1.933348908	6.77E-37	6.65E-35
208	ENSG00000133110.15	POSTN	10631	3.434014196	7.93E-37	7.72E-35
209	ENSG00000223485.4	LINC01615		3.332697258	1.69E-36	1.64E-34
210	ENSG00000117385.16	P3H1	64175	1.738504678	2.03E-36	1.95E-34
211	ENSG00000181135.16	ZNF707	286075	1.134876772	2.11E-36	2.02E-34
212	ENSG00000169679.15	BUB1	699	1.383691899	2.57E-36	2.44E-34
213	ENSG00000117399.14	CDC20	991	1.626433093	2.79E-36	2.64E-34
214	ENSG00000134690.11	CDCA8	55143	1.428067364	3.01E-36	2.84E-34
215	ENSG00000114554.11	PLXNA1	5361	1.315967467	5.65E-36	5.25E-34
216	ENSG00000182197.12	EXT1	2131	1.313999641	5.70E-36	5.28E-34
217	ENSG00000073792.16	IGF2BP2	10644	2.356309484	5.94E-36	5.49E-34
218	ENSG00000156802.13	ATAD2	29028	1.46803673	5.98E-36	5.51E-34
219	ENSG00000197275.14	RAD54B	25788	1.592673597	6.28E-36	5.76E-34
220	ENSG00000158402.20	CDC25C	995	1.748354145	7.85E-36	7.15E-34
221	ENSG00000230570.1	ACCSLP1		3.191540031	7.87E-36	7.16E-34
222	ENSG00000168779.20	SHOX2	6474	2.525073497	9.20E-36	8.29E-34
223	ENSG00000180818.5	HOXC10	3226	4.071792527	9.34E-36	8.39E-34
224	ENSG00000101412.13	E2F1	1869	2.038158724	1.03E-35	9.26E-34
225	ENSG00000287126.1		124907726	2.071676726	1.17E-35	1.04E-33
226	ENSG00000146242.9	TPBG	7162	1.44693443	1.22E-35	1.09E-33
227	ENSG00000100526.20	CDKN3	1033	1.559145111	1.53E-35	1.35E-33
228	ENSG00000161638.11	ITGA5	3678	2.267444383	2.04E-35	1.80E-33
229	ENSG00000182545.6	RNASE10	338879	4.523297092	2.33E-35	2.05E-33
230	ENSG00000179546.4	HTR1D	3352	3.619113539	2.45E-35	2.14E-33
231	ENSG00000250697.2			4.498345873	3.13E-35	2.72E-33
232	ENSG00000168496.4	FEN1	2237	1.259124161	3.43E-35	2.97E-33
233	ENSG00000019549.13	SNAI2	6591	1.66601512	4.24E-35	3.65E-33
234	ENSG00000106089.12	STX1A	6804	1.776271416	4.65E-35	3.99E-33
235	ENSG00000084731.15	KIF3C	3797	1.755097628	4.91E-35	4.19E-33
236	ENSG00000059378.13	PARP12	64761	1.467658382	5.12E-35	4.36E-33
237	ENSG00000182481.10	KPNA2	3838	1.138131107	6.21E-35	5.28E-33
238	ENSG00000130720.13	FIBCD1	84929	4.620294292	7.76E-35	6.56E-33
239	ENSG00000179046.8	TRIML2	205860	5.698247943	9.94E-35	8.36E-33
240	ENSG00000117650.13	NEK2	4751	1.539356532	9.95E-35	8.36E-33
241	ENSG00000006118.15	TMEM132A	54972	1.57875204	1.07E-34	9.00E-33
242	ENSG00000273706.5	LHX1	3975	4.706021953	1.57E-34	1.30E-32
243	ENSG00000224271.7	EPIC1	284930	2.685742642	2.02E-34	1.67E-32
244	ENSG00000244128.7	LINC01322	103695433	4.307008747	2.81E-34	2.29E-32
245	ENSG00000035499.13	DEPDC1B	55789	1.556906104	2.86E-34	2.33E-32
246	ENSG00000105219.10	CCNP	79935	3.455067149	2.87E-34	2.33E-32
247	ENSG00000053747.17	LAMA3	3909	2.689994759	3.67E-34	2.98E-32
248	ENSG00000080986.13	NDC80	10403	1.628822848	4.20E-34	3.39E-32
249	ENSG00000111247.15	RAD51AP1	10635	1.638765113	4.25E-34	3.42E-32
250	ENSG00000184445.12	KNTC1	9735	1.341138527	4.52E-34	3.63E-32
251	ENSG00000271936.1			1.514017967	4.58E-34	3.67E-32
252	ENSG00000173207.13	CKS1B	1163	1.434869522	4.67E-34	3.74E-32
253	ENSG00000126787.13	DLGAP5	9787	1.465279673	4.95E-34	3.94E-32
254	ENSG00000170178.7	HOXD12	3238	5.125106563	5.24E-34	4.15E-32
255	ENSG00000164465.19	DCBLD1	285761	1.967865611	5.28E-34	4.18E-32
256	ENSG00000260976.2	LINC01633		4.4018517	5.85E-34	4.61E-32
257	ENSG00000166073.11	GPR176	11245	2.427104038	6.00E-34	4.72E-32
258	ENSG00000134901.13	POGLUT2	79070	1.471625401	6.01E-34	4.72E-32
259	ENSG00000214429.3	CYCSP6		5.070133683	6.87E-34	5.35E-32
260	ENSG00000165661.17	QSOX2	169714	1.120336156	8.69E-34	6.74E-32
261	ENSG00000188157.15	AGRN	375790	1.585469616	8.86E-34	6.86E-32
262	ENSG00000135622.13	SEMA4F	10505	1.503562417	9.44E-34	7.28E-32
263	ENSG00000232383.1			4.32845316	9.65E-34	7.42E-32
264	ENSG00000105928.16	GSDME	1687	2.084812847	1.07E-33	8.14E-32
265	ENSG00000257042.1			4.127184311	1.19E-33	9.06E-32
266	ENSG00000288062.1		101929748	5.323294045	1.22E-33	9.26E-32
267	ENSG00000156787.17	TBC1D31	93594	1.051804218	1.32E-33	9.95E-32
268	ENSG00000114346.14	ECT2	1894	1.539356283	1.42E-33	1.07E-31
269	ENSG00000153395.10	LPCAT1	79888	1.656381179	1.44E-33	1.08E-31
270	ENSG00000134363.12	FST	10468	2.581240838	1.84E-33	1.37E-31
271	ENSG00000075618.18	FSCN1	6624	1.664069081	1.86E-33	1.38E-31
272	ENSG00000070729.14	CNGB1	1258	3.969098513	1.90E-33	1.40E-31
273	ENSG00000049192.15	ADAMTS6	11174	2.514076191	1.98E-33	1.45E-31
274	ENSG00000251144.1			4.800537064	2.53E-33	1.85E-31
275	ENSG00000286561.1			2.698205885	2.58E-33	1.89E-31
276	ENSG00000149554.13	CHEK1	1111	1.227729442	2.79E-33	2.03E-31
277	ENSG00000164430.16	CGAS	115004	1.535128172	3.12E-33	2.26E-31
278	ENSG00000231131.8	LNCAROD	105378305	3.743500308	3.22E-33	2.33E-31
279	ENSG00000277268.2	LHX1-DT	102723471	4.514367646	4.34E-33	3.10E-31
280	ENSG00000125869.10	LAMP5	24141	2.969134826	4.59E-33	3.26E-31
281	ENSG00000137404.14	NRM	11270	1.213872661	4.66E-33	3.31E-31
282	ENSG00000124225.16	PMEPA1	56937	2.212964386	5.84E-33	4.13E-31
283	ENSG00000237575.4	PYY2		3.941425451	6.38E-33	4.50E-31
284	ENSG00000147381.11	MAGEA4	4103	7.001666599	6.54E-33	4.59E-31
285	ENSG00000131351.15	HAUS8	93323	1.223179876	8.18E-33	5.71E-31
286	ENSG00000111602.12	TIMELESS	8914	1.208191394	8.52E-33	5.93E-31
287	ENSG00000276043.5	UHRF1	29128	1.526478295	1.25E-32	8.64E-31
288	ENSG00000105989.10	WNT2	7472	3.311242321	1.35E-32	9.31E-31
289	ENSG00000106366.9	SERPINE1	5054	2.854965254	1.39E-32	9.54E-31
290	ENSG00000160886.13	LY6K	54742	2.298537111	1.41E-32	9.67E-31
291	ENSG00000139219.19	COL2A1	1280	3.802502526	1.72E-32	1.17E-30
292	ENSG00000136982.6	DSCC1	79075	1.34219882	1.95E-32	1.32E-30
293	ENSG00000140451.13	PIF1	80119	1.827324734	2.58E-32	1.75E-30
294	ENSG00000051128.19	HOMER3	9454	1.644571564	2.93E-32	1.97E-30
295	ENSG00000131094.4	C1QL1	10882	3.092115426	3.04E-32	2.04E-30
296	ENSG00000130429.15	ARPC1B	10095	1.526478167	3.15E-32	2.11E-30
297	ENSG00000121621.7	KIF18A	81930	1.47015122	3.20E-32	2.14E-30
298	ENSG00000183770.7	FOXL2	668	3.758956295	3.27E-32	2.18E-30
299	ENSG00000161692.18	DBF4B	80174	1.206667901	3.55E-32	2.36E-30
300	ENSG00000187775.17	DNAH17	8632	3.131680573	4.03E-32	2.68E-30
301	ENSG00000018280.17	SLC11A1	6556	2.057737244	5.03E-32	3.31E-30
302	ENSG00000151025.11	GPR158	57512	3.571185758	5.14E-32	3.38E-30
303	ENSG00000198353.8	HOXC4	3221	2.449979011	5.15E-32	3.38E-30
304	ENSG00000189238.6	LINC00943	100507206	2.918282989	5.16E-32	3.38E-30
305	ENSG00000164932.13	CTHRC1	115908	2.607682071	5.27E-32	3.45E-30
306	ENSG00000130303.13	BST2	684	2.542650373	6.09E-32	3.97E-30
307	ENSG00000203650.9	LINC01285	101928287	3.849961537	6.44E-32	4.19E-30
308	ENSG00000072864.16	NDE1	54820	1.136101999	6.48E-32	4.21E-30
309	ENSG00000146839.19	ZAN	7455	3.510678754	6.64E-32	4.30E-30
310	ENSG00000006377.11	DLX6	1750	3.225117595	6.78E-32	4.39E-30
311	ENSG00000158023.10	CFAP251	144406	1.932082235	7.14E-32	4.61E-30
312	ENSG00000283178.1			4.935348335	8.24E-32	5.27E-30
313	ENSG00000166845.15	C18orf54	162681	1.431929955	1.10E-31	7.02E-30
314	ENSG00000267284.2		105372126	3.354830706	1.28E-31	8.11E-30
315	ENSG00000173805.16	HAP1	9001	2.882790732	1.40E-31	8.83E-30
316	ENSG00000108106.14	UBE2S	27338	1.631830364	1.45E-31	9.17E-30
317	ENSG00000189420.8	ZFP92	139735	2.28922838	1.48E-31	9.34E-30
318	ENSG00000240280.7	TCAM1P		5.28288667	1.51E-31	9.45E-30
319	ENSG00000198792.13	TMEM184B	25829	1.004712489	1.68E-31	1.04E-29
320	ENSG00000117586.11	TNFSF4	7292	2.413744853	1.71E-31	1.06E-29
321	ENSG00000115297.11	TLX2	3196	4.135706626	1.72E-31	1.07E-29
322	ENSG00000149968.12	MMP3	4314	3.772146133	1.85E-31	1.15E-29
323	ENSG00000287626.1	DTNBP1-AS1		3.084401914	2.16E-31	1.33E-29
324	ENSG00000150276.8	PPIAP26		4.748036458	2.19E-31	1.34E-29
325	ENSG00000170779.11	CDCA4	55038	1.141787098	2.63E-31	1.60E-29
326	ENSG00000129195.16	PIMREG	54478	1.795885897	2.80E-31	1.70E-29
327	ENSG00000225210.10	DUXAP9		3.338647997	2.84E-31	1.72E-29
328	ENSG00000115414.21	FN1	2335	3.303241863	2.90E-31	1.75E-29
329	ENSG00000131470.15	PSMC3IP	29893	1.301014935	3.33E-31	2.01E-29
330	ENSG00000077152.11	UBE2T	29089	1.447909663	3.40E-31	2.05E-29
331	ENSG00000198930.13	CSAG1	158511	5.862883901	3.71E-31	2.23E-29
332	ENSG00000164744.13	SUN3	256979	4.316771104	4.03E-31	2.42E-29
333	ENSG00000101003.10	GINS1	9837	1.355095842	4.36E-31	2.61E-29
334	ENSG00000083444.17	PLOD1	5351	1.436822174	5.15E-31	3.07E-29
335	ENSG00000187678.10	SPRY4	81848	1.617804346	5.29E-31	3.15E-29
336	ENSG00000157227.13	MMP14	4323	1.465708291	5.35E-31	3.18E-29
337	ENSG00000077935.17	SMC1B	27127	4.817809201	5.55E-31	3.29E-29
338	ENSG00000120334.15	CENPL	91687	1.096669348	5.73E-31	3.39E-29
339	ENSG00000101115.13	SALL4	57167	2.720572199	6.12E-31	3.61E-29
340	ENSG00000165304.8	MELK	9833	1.572108354	6.31E-31	3.72E-29
341	ENSG00000128944.13	KNSTRN	90417	1.087996275	6.74E-31	3.97E-29
342	ENSG00000166881.10	NEMP1	23306	1.370537707	6.88E-31	4.05E-29
343	ENSG00000149380.12	P4HA3	283208	2.657507248	6.97E-31	4.08E-29
344	ENSG00000167941.3	SOST	50964	4.507297526	7.25E-31	4.23E-29
345	ENSG00000167550.11	RHEBL1	121268	1.874697512	8.77E-31	5.10E-29
346	ENSG00000141696.13	P3H4	10609	1.705410195	9.07E-31	5.27E-29
347	ENSG00000205922.5	ONECUT3	390874	4.590182134	1.08E-30	6.23E-29
348	ENSG00000145040.4	UCN2	90226	2.23812621	1.21E-30	6.95E-29
349	ENSG00000287625.1			2.128421431	1.26E-30	7.24E-29
350	ENSG00000223749.11	MIR503HG		2.944967166	1.42E-30	8.11E-29
351	ENSG00000177234.7	LINC01561	404216	4.695324226	1.46E-30	8.33E-29
352	ENSG00000256268.2	LINC02454		3.542254202	2.20E-30	1.22E-28
353	ENSG00000253616.5	CHILL1		2.712870185	2.34E-30	1.30E-28
354	ENSG00000280206.1			1.396446342	2.49E-30	1.38E-28
355	ENSG00000124731.13	TREM1	54210	2.926148287	2.62E-30	1.45E-28
356	ENSG00000185247.15	MAGEA11	4110	6.586423678	2.75E-30	1.52E-28
357	ENSG00000177602.5	HASPIN	83903	1.32972524	2.80E-30	1.54E-28
358	ENSG00000239620.3	PRR20G	100419008	5.332408287	3.56E-30	1.96E-28
359	ENSG00000127586.17	CHTF18	63922	1.408711267	4.10E-30	2.24E-28
360	ENSG00000143228.13	NUF2	83540	1.492331556	4.14E-30	2.27E-28
361	ENSG00000135314.12	KHDC1	80759	1.630662853	4.24E-30	2.31E-28
362	ENSG00000283154.2	IQCJ-SCHIP1	100505385	1.734570382	4.75E-30	2.58E-28
363	ENSG00000249395.4	CASC9	101805492	3.735840737	4.82E-30	2.62E-28
364	ENSG00000040608.14	RTN4R	65078	1.674857255	5.07E-30	2.75E-28
365	ENSG00000156510.13	HKDC1	80201	3.796401442	5.55E-30	3.00E-28
366	ENSG00000169174.11	PCSK9	255738	2.718475584	6.15E-30	3.32E-28
367	ENSG00000085840.13	ORC1	4998	1.400836447	6.74E-30	3.63E-28
368	ENSG00000065485.20	PDIA5	10954	1.204521766	7.74E-30	4.17E-28
369	ENSG00000236212.2			3.397538216	7.95E-30	4.27E-28
370	ENSG00000118263.15	KLF7	8609	1.567445748	8.63E-30	4.62E-28
371	ENSG00000227440.1	ATP5MC1P4		2.310478122	8.78E-30	4.69E-28
372	ENSG00000287055.1			3.280154155	1.07E-29	5.72E-28
373	ENSG00000244649.5	LINC02086		3.901331715	1.19E-29	6.32E-28
374	ENSG00000260517.3			2.859990446	1.20E-29	6.38E-28
375	ENSG00000166130.15	IKBIP	121457	1.191964924	1.28E-29	6.73E-28
376	ENSG00000161791.14	FMNL3	91010	1.276527905	1.37E-29	7.22E-28
377	ENSG00000286177.1			1.605133768	1.49E-29	7.82E-28
378	ENSG00000131650.14	KREMEN2	79412	2.886318195	1.59E-29	8.29E-28
379	ENSG00000002079.14			2.999850556	1.83E-29	9.53E-28
380	ENSG00000259807.2		101928188	2.575187684	2.04E-29	1.06E-27
381	ENSG00000244675.2			3.866881602	2.28E-29	1.18E-27
382	ENSG00000111012.10	CYP27B1	1594	2.420210168	2.35E-29	1.21E-27
383	ENSG00000162063.13	CCNF	899	1.247641319	2.79E-29	1.44E-27
384	ENSG00000272183.1			1.851042429	2.79E-29	1.44E-27
385	ENSG00000108821.14	COL1A1	1277	2.910641282	3.01E-29	1.54E-27
386	ENSG00000136378.15	ADAMTS7	11173	2.029133098	3.22E-29	1.64E-27
387	ENSG00000187741.15	FANCA	2175	1.270360622	4.00E-29	2.03E-27
388	ENSG00000131153.9	GINS2	51659	1.50126273	4.36E-29	2.21E-27
389	ENSG00000131944.10	FAAP24	91442	1.074364917	4.37E-29	2.21E-27
390	ENSG00000287721.1			2.292039169	4.74E-29	2.39E-27
391	ENSG00000277883.1	NLRP3P1		2.538157624	4.86E-29	2.45E-27
392	ENSG00000167634.12	NLRP7	199713	3.871662035	4.92E-29	2.46E-27
393	ENSG00000242207.1	HOXB-AS4	100874351	5.317364297	4.95E-29	2.48E-27
394	ENSG00000228630.5	HOTAIR	100124700	3.618818229	4.97E-29	2.49E-27
395	ENSG00000175040.6	CHST2	9435	2.502510027	5.25E-29	2.62E-27
396	ENSG00000180263.14	FGD6	55785	1.29308781	6.15E-29	3.05E-27
397	ENSG00000182379.10	NXPH4	11247	2.372167307	7.74E-29	3.80E-27
398	ENSG00000136931.10	NR5A1	2516	6.339877506	8.44E-29	4.13E-27
399	ENSG00000169607.13	CKAP2L	150468	1.38770671	8.55E-29	4.18E-27
400	ENSG00000196787.3	H2AC11	8969	2.019786291	8.74E-29	4.27E-27
401	ENSG00000103005.12	USB1	79650	1.145299055	9.74E-29	4.74E-27
402	ENSG00000113722.17	CDX1	1044	2.832353886	1.11E-28	5.37E-27
403	ENSG00000274964.1			1.580549003	1.21E-28	5.85E-27
404	ENSG00000105011.9	ASF1B	55723	1.487713445	1.37E-28	6.60E-27
405	ENSG00000237649.8	KIFC1	3833	1.285674338	1.41E-28	6.80E-27
406	ENSG00000268655.2	SAXO3	101059948	2.569013775	1.71E-28	8.19E-27
407	ENSG00000175643.10	RMI2	116028	1.609651073	1.77E-28	8.49E-27
408	ENSG00000126215.14	XRCC3	7517	1.193223678	2.03E-28	9.70E-27
409	ENSG00000105255.11	FSD1	79187	2.737849141	2.07E-28	9.88E-27
410	ENSG00000283554.1	LINC02341		2.587495722	2.22E-28	1.06E-26
411	ENSG00000100297.16	MCM5	4174	1.225007697	2.37E-28	1.12E-26
412	ENSG00000013810.20	TACC3	10460	1.247873369	2.47E-28	1.17E-26
413	ENSG00000205634.7	LINC00898	400932	4.103014801	2.70E-28	1.28E-26
414	ENSG00000236347.1			2.963388126	2.80E-28	1.32E-26
415	ENSG00000258791.9	LINC00520	645687	3.332618962	2.98E-28	1.40E-26
416	ENSG00000078098.14	FAP	2191	2.599488276	3.17E-28	1.49E-26
417	ENSG00000273983.1	H3C8	8355	2.635863603	3.33E-28	1.56E-26
418	ENSG00000008300.17	CELSR3	1951	2.380232031	3.39E-28	1.58E-26
419	ENSG00000055332.18	EIF2AK2	5610	1.126402191	3.41E-28	1.59E-26
420	ENSG00000176826.15	FKBP9P1		2.424768908	3.61E-28	1.68E-26
421	ENSG00000162493.16	PDPN	10630	2.038590704	3.73E-28	1.73E-26
422	ENSG00000184613.11	NELL2	4753	3.034359915	3.80E-28	1.76E-26
423	ENSG00000265933.5	LINC00668	400643	4.452191185	3.86E-28	1.79E-26
424	ENSG00000226468.2			3.323889105	3.99E-28	1.84E-26
425	ENSG00000229618.3			2.786330844	4.37E-28	2.01E-26
426	ENSG00000126453.9	BCL2L12	83596	1.106169705	4.91E-28	2.25E-26
427	ENSG00000080573.7	COL5A3	50509	2.091249754	5.09E-28	2.33E-26
428	ENSG00000287064.1			3.99302554	5.89E-28	2.69E-26
429	ENSG00000260597.1			4.489323209	6.20E-28	2.83E-26
430	ENSG00000167513.9	CDT1	81620	1.473139721	6.45E-28	2.94E-26
431	ENSG00000161888.11	SPC24	147841	1.657347233	6.92E-28	3.14E-26
432	ENSG00000155254.13	MARVELD1	83742	1.394704404	7.21E-28	3.27E-26
433	ENSG00000198443.6	KRTAP4-1	85285	3.326949514	7.45E-28	3.37E-26
434	ENSG00000002822.15	MAD1L1	8379	2.503800758	8.77E-28	3.96E-26
435	ENSG00000257114.3	LINC02450	105369738	3.701502248	9.37E-28	4.23E-26
436	ENSG00000196550.10	FAM72A	729533	1.488551933	9.40E-28	4.24E-26
437	ENSG00000251281.1			5.309381016	9.73E-28	4.38E-26
438	ENSG00000091409.15	ITGA6	3655	1.874951395	1.01E-27	4.54E-26
439	ENSG00000173638.19	SLC19A1	6573	1.305765667	1.05E-27	4.72E-26
440	ENSG00000250920.3			5.143360199	1.07E-27	4.81E-26
441	ENSG00000163283.7	ALPP	250	5.726964535	1.08E-27	4.83E-26
442	ENSG00000170312.16	CDK1	983	1.262092684	1.28E-27	5.68E-26
443	ENSG00000204624.8	DISP3	57540	3.77270835	1.51E-27	6.71E-26
444	ENSG00000065328.17	MCM10	55388	1.339414621	1.54E-27	6.81E-26
445	ENSG00000233818.1			2.218209892	1.64E-27	7.26E-26
446	ENSG00000148942.15	SLC5A12	159963	3.545024996	1.70E-27	7.50E-26
447	ENSG00000213420.8	GPC2	221914	2.405642317	1.73E-27	7.61E-26
448	ENSG00000126091.21	ST3GAL3	6487	1.56585752	1.82E-27	7.98E-26
449	ENSG00000284719.1		130932201	2.176154764	1.97E-27	8.62E-26
450	ENSG00000214160.10	ALG3	10195	1.059223238	2.13E-27	9.28E-26
451	ENSG00000149485.19	FADS1	3992	1.884157754	2.20E-27	9.56E-26
452	ENSG00000185338.6	SOCS1	8651	1.78490739	2.39E-27	1.04E-25
453	ENSG00000255571.9	MIR9-3HG	254559	3.369047966	2.69E-27	1.16E-25
454	ENSG00000272405.1			3.172337263	2.74E-27	1.18E-25
455	ENSG00000133216.17	EPHB2	2048	2.126241875	2.90E-27	1.25E-25
456	ENSG00000151651.16	ADAM8	101	1.920097894	3.12E-27	1.35E-25
457	ENSG00000261780.3	LINC02582	100505817	6.341453235	3.39E-27	1.46E-25
458	ENSG00000188610.12	FAM72B	653820	1.531063422	3.61E-27	1.55E-25
459	ENSG00000278828.1	H3C10	8357	2.209042965	4.33E-27	1.85E-25
460	ENSG00000237989.1	LINC01679	101928399	1.932818795	4.58E-27	1.95E-25
461	ENSG00000110446.11	SLC15A3	51296	1.804597757	4.79E-27	2.04E-25
462	ENSG00000274641.2	H2BC17	8348	3.056508334	5.32E-27	2.25E-25
463	ENSG00000179772.8	FOXS1	2307	2.009754652	6.01E-27	2.54E-25
464	ENSG00000267432.6	DNAH17-AS1		3.195521728	6.40E-27	2.70E-25
465	ENSG00000170289.12	CNGB3	54714	2.931873374	7.46E-27	3.12E-25
466	ENSG00000225614.4	ZNF469	84627	2.475393943	8.16E-27	3.40E-25
467	ENSG00000169436.17	COL22A1	169044	3.525864215	8.58E-27	3.58E-25
468	ENSG00000261787.2	TCF24	100129654	2.825308558	8.87E-27	3.69E-25
469	ENSG00000257596.1	SCAT2	112935960	2.881495326	1.01E-26	4.17E-25
470	ENSG00000197172.10	MAGEA6	4102	6.452246724	1.04E-26	4.31E-25
471	ENSG00000121594.12	CD80	941	2.085508759	1.12E-26	4.62E-25
472	ENSG00000122884.12	P4HA1	5033	1.382788243	1.26E-26	5.15E-25
473	ENSG00000130822.16	PNCK	139728	3.431236677	1.39E-26	5.69E-25
474	ENSG00000151746.15	BICD1	636	1.343620124	1.41E-26	5.78E-25
475	ENSG00000215784.6	FAM72D	728833	1.669051269	1.52E-26	6.21E-25
476	ENSG00000132031.13	MATN3	4148	2.633329022	1.63E-26	6.65E-25
477	ENSG00000287125.1			2.034363607	1.73E-26	7.04E-25
478	ENSG00000260086.3		105371240	2.061430961	1.85E-26	7.49E-25
479	ENSG00000287335.1			2.921848729	2.00E-26	8.06E-25
480	ENSG00000139211.7	AMIGO2	347902	2.242810378	2.09E-26	8.41E-25
481	ENSG00000254605.1			3.187424639	2.11E-26	8.49E-25
482	ENSG00000117152.14	RGS4	5999	2.554897047	2.13E-26	8.52E-25
483	ENSG00000102034.17	ELF4	2000	1.101794918	2.13E-26	8.52E-25
484	ENSG00000230479.1	LNCTSI		2.272791025	2.13E-26	8.52E-25
485	ENSG00000153404.14	PLEKHG4B	153478	2.636682368	2.40E-26	9.56E-25
486	ENSG00000146112.12	PPP1R18	170954	1.082394709	2.64E-26	1.05E-24
487	ENSG00000164362.21	TERT	7015	2.739794606	2.67E-26	1.06E-24
488	ENSG00000183856.11	IQGAP3	128239	1.345501562	2.69E-26	1.06E-24
489	ENSG00000286010.1		105375754	2.437058381	2.69E-26	1.06E-24
490	ENSG00000204516.10	MICB	4277	1.790501438	2.83E-26	1.12E-24
491	ENSG00000169248.13	CXCL11	6373	3.845662692	3.17E-26	1.25E-24
492	ENSG00000148408.13	CACNA1B	774	3.972583938	3.34E-26	1.31E-24
493	ENSG00000163462.18	TRIM46	80128	1.655483262	3.67E-26	1.44E-24
494	ENSG00000286355.1			3.104300856	4.04E-26	1.57E-24
495	ENSG00000137965.11	IFI44	10561	2.125250676	4.14E-26	1.61E-24
496	ENSG00000176887.7	SOX11	6664	3.02530399	5.30E-26	2.05E-24
497	ENSG00000204710.2	SPDYC	387778	5.930274635	5.55E-26	2.14E-24
498	ENSG00000196747.4	H2AC13	8329	2.455653005	5.65E-26	2.17E-24
499	ENSG00000197859.11	ADAMTSL2	9719	1.538908778	5.96E-26	2.29E-24
500	ENSG00000196932.12	TMEM26	219623	2.164841012	6.00E-26	2.30E-24
501	ENSG00000143355.16	LHX9	56956	4.178202099	6.28E-26	2.40E-24
502	ENSG00000154102.11	C16orf74	404550	1.732904982	6.65E-26	2.54E-24
503	ENSG00000251026.2	NIHCOLE		5.002539444	6.73E-26	2.57E-24
504	ENSG00000197565.16	COL4A6	1288	2.711341771	6.99E-26	2.66E-24
505	ENSG00000140525.19	FANCI	55215	1.188035879	7.15E-26	2.72E-24
506	ENSG00000154839.10	SKA1	220134	1.333799821	7.19E-26	2.73E-24
507	ENSG00000196878.15	LAMB3	3914	1.800589154	7.33E-26	2.79E-24
508	ENSG00000160161.9	CILP2	148113	2.868435847	7.37E-26	2.79E-24
509	ENSG00000051341.14	POLQ	10721	1.474709931	8.43E-26	3.19E-24
510	ENSG00000272068.1	BCAN-AS1		3.778416011	8.57E-26	3.24E-24
511	ENSG00000240764.4	PCDHGC5	56097	3.515666617	8.87E-26	3.35E-24
512	ENSG00000166508.18	MCM7	4176	1.219689807	9.14E-26	3.45E-24
513	ENSG00000175874.10	CREG2	200407	3.00735386	9.49E-26	3.57E-24
514	ENSG00000177732.8	SOX12	6666	1.392511157	1.01E-25	3.79E-24
515	ENSG00000073111.14	MCM2	4171	1.660013919	1.03E-25	3.84E-24
516	ENSG00000128340.15	RAC2	5880	1.601998768	1.07E-25	4.01E-24
517	ENSG00000270372.1	LINC03131		4.969490805	1.09E-25	4.07E-24
518	ENSG00000250564.2	LINC02999		4.717313312	1.09E-25	4.07E-24
519	ENSG00000142552.8	RCN3	57333	2.181398235	1.11E-25	4.12E-24
520	ENSG00000280734.3	LINC01232		1.444825544	1.16E-25	4.33E-24
521	ENSG00000066697.15	MSANTD3	91283	1.010880048	1.25E-25	4.63E-24
522	ENSG00000233085.5			5.66054609	1.25E-25	4.64E-24
523	ENSG00000286058.1			6.592266158	1.31E-25	4.82E-24
524	ENSG00000275216.2			3.221893789	1.36E-25	5.01E-24
525	ENSG00000117724.13	CENPF	1063	1.34416927	1.54E-25	5.62E-24
526	ENSG00000128641.19	MYO1B	4430	1.592114999	1.61E-25	5.87E-24
527	ENSG00000121904.17	CSMD2	114784	2.674576129	1.62E-25	5.92E-24
528	ENSG00000198826.11	ARHGAP11A	9824	1.150627962	1.67E-25	6.07E-24
529	ENSG00000222032.1			4.195946381	1.71E-25	6.21E-24
530	ENSG00000054356.14	PTPRN	5798	3.264987158	1.78E-25	6.46E-24
531	ENSG00000130270.16	ATP8B3	148229	1.860571087	1.80E-25	6.52E-24
532	ENSG00000124657.1	OR2B6	26212	4.093623584	1.84E-25	6.66E-24
533	ENSG00000137135.18	ARHGEF39	84904	1.607152338	1.86E-25	6.71E-24
534	ENSG00000169213.7	RAB3B	5865	3.537866509	1.95E-25	7.02E-24
535	ENSG00000090530.10	P3H2	55214	2.526155083	2.02E-25	7.22E-24
536	ENSG00000130487.9	KLHDC7B	113730	3.262609862	2.05E-25	7.32E-24
537	ENSG00000213928.9	IRF9	10379	1.763691717	2.31E-25	8.25E-24
538	ENSG00000196839.13	ADA	100	1.54662891	2.43E-25	8.63E-24
539	ENSG00000197261.11	C6orf141	135398	2.309743021	2.44E-25	8.66E-24
540	ENSG00000128709.13	HOXD9	3235	1.716879028	2.50E-25	8.89E-24
541	ENSG00000248693.1	LINC02100		3.098493667	2.58E-25	9.16E-24
542	ENSG00000166451.13	CENPN	55839	1.056422419	2.76E-25	9.76E-24
543	ENSG00000101224.17	CDC25B	994	1.37522167	2.78E-25	9.80E-24
544	ENSG00000223812.8	PYDC2-AS1		5.871045142	2.90E-25	1.02E-23
545	ENSG00000258955.2	LINC00519		2.381237644	3.16E-25	1.11E-23
546	ENSG00000144824.21	PHLDB2	90102	1.675053749	3.61E-25	1.26E-23
547	ENSG00000152253.9	SPC25	57405	1.196357734	3.67E-25	1.28E-23
548	ENSG00000100558.9	PLEK2	26499	1.873124173	4.18E-25	1.45E-23
549	ENSG00000170627.11	GTSF1	121355	3.980050811	4.75E-25	1.64E-23
550	ENSG00000109805.10	NCAPG	64151	1.238390635	4.90E-25	1.69E-23
551	ENSG00000196460.14	RFX8	731220	2.377107002	4.99E-25	1.72E-23
552	ENSG00000145107.16	TM4SF19	116211	2.662785056	5.43E-25	1.86E-23
553	ENSG00000070404.10	FSTL3	10272	2.270053855	5.47E-25	1.87E-23
554	ENSG00000230988.3	RPL23AP11		4.36343139	6.12E-25	2.09E-23
555	ENSG00000138316.11	ADAMTS14	140766	1.966833315	6.79E-25	2.30E-23
556	ENSG00000276067.2	SLC23A4P		3.876404971	7.21E-25	2.44E-23
557	ENSG00000138658.16	ZGRF1	55345	1.167350738	7.32E-25	2.48E-23
558	ENSG00000115718.18	PROC	5624	2.672248651	8.02E-25	2.71E-23
559	ENSG00000284095.1			3.768275668	8.12E-25	2.74E-23
560	ENSG00000100253.13	MIOX	55586	4.496092816	8.58E-25	2.89E-23
561	ENSG00000237548.1			3.381762904	9.20E-25	3.09E-23
562	ENSG00000144136.11	SLC20A1	6574	1.10804197	9.34E-25	3.14E-23
563	ENSG00000134222.16	PSRC1	84722	1.230723841	1.21E-24	4.05E-23
564	ENSG00000173193.15	PARP14	54625	1.325044811	1.23E-24	4.12E-23
565	ENSG00000091136.15	LAMB1	3912	1.354272162	1.30E-24	4.34E-23
566	ENSG00000251493.5	FOXD1	2297	1.917272193	1.31E-24	4.36E-23
567	ENSG00000240498.9	CDKN2B-AS1	100048912	2.39077191	1.45E-24	4.80E-23
568	ENSG00000165949.12	IFI27	3429	2.08949639	1.46E-24	4.85E-23
569	ENSG00000138346.15	DNA2	1763	1.165650127	1.81E-24	5.94E-23
570	ENSG00000123364.5	HOXC13	3229	2.497872075	1.81E-24	5.94E-23
571	ENSG00000278399.1			3.702807475	1.85E-24	6.04E-23
572	ENSG00000183150.8	GPR19	2842	2.084306594	1.87E-24	6.12E-23
573	ENSG00000236841.8			3.193754827	2.04E-24	6.65E-23
574	ENSG00000115523.17	GNLY	10578	2.588367065	2.08E-24	6.77E-23
575	ENSG00000104321.11	TRPA1	8989	2.67972338	2.23E-24	7.23E-23
576	ENSG00000268916.6	CSAG3	389903	4.350231912	2.42E-24	7.82E-23
577	ENSG00000118785.14	SPP1	6696	3.259602073	2.48E-24	8.02E-23
578	ENSG00000178217.14	SH2D4B	387694	2.508987184	2.78E-24	8.95E-23
579	ENSG00000273186.1			2.09277883	2.91E-24	9.38E-23
580	ENSG00000197847.12	SLC22A20P		2.803601584	2.99E-24	9.62E-23
581	ENSG00000175455.16	CCDC14	64770	1.158667015	2.99E-24	9.62E-23
582	ENSG00000250451.5	HOXC-AS1	100874363	2.983519338	3.01E-24	9.66E-23
583	ENSG00000138778.13	CENPE	1062	1.244723462	3.08E-24	9.87E-23
584	ENSG00000112312.10	GMNN	51053	1.147979686	3.14E-24	1.00E-22
585	ENSG00000231560.1	CLEC12A-AS1	400002	3.197469098	3.34E-24	1.07E-22
586	ENSG00000166922.8	SCG5	6447	2.382633134	3.37E-24	1.07E-22
587	ENSG00000164109.14	MAD2L1	4085	1.19245538	3.45E-24	1.10E-22
588	ENSG00000119915.5	ELOVL3	83401	2.381965622	3.51E-24	1.12E-22
589	ENSG00000125726.11	CD70	970	2.863827388	3.83E-24	1.22E-22
590	ENSG00000231948.2	HS1BP3-IT1	100874343	3.335467703	4.06E-24	1.28E-22
591	ENSG00000189423.13	USP32P3		2.653455421	4.42E-24	1.39E-22
592	ENSG00000170537.13	TMC7	79905	1.639663465	4.49E-24	1.42E-22
593	ENSG00000213030.5	CGB8	94115	5.314542639	4.89E-24	1.54E-22
594	ENSG00000203446.2	SUGCT-AS1		3.233397557	5.03E-24	1.58E-22
595	ENSG00000163064.7	EN1	2019	2.936973607	5.20E-24	1.63E-22
596	ENSG00000186862.20	PDZD7	79955	2.471813623	5.73E-24	1.79E-22
597	ENSG00000237380.7	HOXD-AS2		2.459925385	5.96E-24	1.86E-22
598	ENSG00000117394.24	SLC2A1	6513	1.703498069	6.04E-24	1.88E-22
599	ENSG00000213401.10	MAGEA12	4111	7.578120297	6.12E-24	1.91E-22
600	ENSG00000124260.12	MAGEA10	4109	7.901865245	6.66E-24	2.07E-22
601	ENSG00000233589.1	DEPDC1-AS2		4.049822521	6.87E-24	2.14E-22
602	ENSG00000236081.1	ELFN1-AS1	101927125	4.564819398	7.20E-24	2.23E-22
603	ENSG00000171658.8	NMRAL2P		3.439346295	7.35E-24	2.28E-22
604	ENSG00000176383.9	B3GNT4	79369	1.83139297	7.60E-24	2.35E-22
605	ENSG00000104738.18	MCM4	4173	1.039659281	7.90E-24	2.44E-22
606	ENSG00000223960.7	CHROMR	101927027	1.057925134	8.12E-24	2.50E-22
607	ENSG00000227777.1	RPL7AP19		3.710612261	8.78E-24	2.70E-22
608	ENSG00000228168.1	HNRNPA1P21		1.853387257	9.14E-24	2.81E-22
609	ENSG00000275393.1			2.269976856	9.69E-24	2.97E-22
610	ENSG00000183929.7	DUSP5P1		3.109088627	9.83E-24	3.02E-22
611	ENSG00000287929.1			2.922217176	1.00E-23	3.06E-22
612	ENSG00000279739.1			3.44980196	1.01E-23	3.09E-22
613	ENSG00000173621.9	LRFN4	78999	1.447968249	1.02E-23	3.12E-22
614	ENSG00000123473.16	STIL	6491	1.219579866	1.02E-23	3.12E-22
615	ENSG00000276180.1	H4C9	8294	1.65685175	1.07E-23	3.25E-22
616	ENSG00000263745.7		105371956	2.058709255	1.09E-23	3.32E-22
617	ENSG00000128656.15	CHN1	1123	1.60091043	1.13E-23	3.43E-22
618	ENSG00000224189.8	HAGLR	401022	2.199747393	1.26E-23	3.83E-22
619	ENSG00000109674.4	NEIL3	55247	1.452211245	1.28E-23	3.88E-22
620	ENSG00000181126.13	HLA-V		2.465472795	1.29E-23	3.89E-22
621	ENSG00000119698.12	PPP4R4	57718	2.510772841	1.29E-23	3.91E-22
622	ENSG00000163623.9	NKX6-1	4825	2.922123222	1.31E-23	3.95E-22
623	ENSG00000145555.15	MYO10	4651	1.247888059	1.38E-23	4.16E-22
624	ENSG00000182325.11	FBXL6	26233	1.136408492	1.40E-23	4.22E-22
625	ENSG00000134247.10	PTGFRN	5738	1.206198778	1.56E-23	4.68E-22
626	ENSG00000105173.14	CCNE1	898	1.453438998	1.67E-23	4.98E-22
627	ENSG00000131747.15	TOP2A	7153	1.327196135	1.70E-23	5.07E-22
628	ENSG00000163359.16	COL6A3	1293	2.375640793	1.74E-23	5.18E-22
629	ENSG00000149557.14	FEZ1	9638	2.071584162	1.94E-23	5.76E-22
630	ENSG00000138646.9	HERC5	51191	1.931198119	2.11E-23	6.25E-22
631	ENSG00000225886.3			3.183866328	2.14E-23	6.33E-22
632	ENSG00000099399.6	MAGEB2	4113	7.395234611	2.36E-23	6.97E-22
633	ENSG00000164211.13	STARD4	134429	1.316177231	2.55E-23	7.50E-22
634	ENSG00000114993.17	RTKN	6242	1.02020241	2.69E-23	7.89E-22
635	ENSG00000104826.15	LHB	3972	2.624815514	3.11E-23	9.08E-22
636	ENSG00000131015.5	ULBP2	80328	1.743617821	3.14E-23	9.17E-22
637	ENSG00000280721.1	LINC01943	101928173	1.818049353	3.23E-23	9.41E-22
638	ENSG00000249001.5	DMP1-AS1	138842265	3.164639358	3.25E-23	9.47E-22
639	ENSG00000197301.7	HMGA2-AS1	100129940	2.298404873	3.28E-23	9.55E-22
640	ENSG00000231764.10	DLX6-AS1		2.398415461	3.34E-23	9.71E-22
641	ENSG00000229656.7	ITGB1-DT		3.193401577	3.55E-23	1.03E-21
642	ENSG00000261008.7	LINC01572	101927957	1.871509612	3.60E-23	1.04E-21
643	ENSG00000164778.4	EN2	2020	2.865317071	3.65E-23	1.06E-21
644	ENSG00000285280.2	RGS2-AS1		2.060950102	3.87E-23	1.12E-21
645	ENSG00000142731.11	PLK4	10733	1.083640357	3.93E-23	1.14E-21
646	ENSG00000090776.6	EFNB1	1947	1.268783227	4.08E-23	1.17E-21
647	ENSG00000261072.2			3.56541655	4.13E-23	1.19E-21
648	ENSG00000029993.15	HMGB3	3149	1.266822056	4.55E-23	1.30E-21
649	ENSG00000133063.16	CHIT1	1118	3.798581582	4.58E-23	1.31E-21
650	ENSG00000124191.18	TOX2	84969	1.950255122	4.61E-23	1.32E-21
651	ENSG00000287263.1			1.78800816	4.89E-23	1.39E-21
652	ENSG00000146070.17	PLA2G7	7941	1.917652775	4.90E-23	1.39E-21
653	ENSG00000112742.10	TTK	7272	1.172287353	5.24E-23	1.48E-21
654	ENSG00000258884.2	LINC02321		2.348851313	5.55E-23	1.57E-21
655	ENSG00000084636.18	COL16A1	1307	1.665858866	5.78E-23	1.63E-21
656	ENSG00000229107.2	ABHD17AP4		3.531500763	5.88E-23	1.66E-21
657	ENSG00000024526.17	DEPDC1	55635	1.202434411	5.92E-23	1.67E-21
658	ENSG00000137948.18	BRDT	676	7.253166225	6.79E-23	1.91E-21
659	ENSG00000000460.17	FIRRM	55732	1.085287995	6.97E-23	1.96E-21
660	ENSG00000185803.12	SLC52A2	79581	1.057153898	7.28E-23	2.04E-21
661	ENSG00000184979.10	USP18	11274	1.720992653	7.55E-23	2.11E-21
662	ENSG00000256967.1			1.83547761	7.65E-23	2.14E-21
663	ENSG00000176678.6	FOXL1	2300	2.297498775	7.84E-23	2.19E-21
664	ENSG00000144395.18	CCDC150	284992	1.605513333	7.94E-23	2.22E-21
665	ENSG00000186564.6	FOXD2	2306	1.582073203	8.40E-23	2.34E-21
666	ENSG00000147246.10	HTR2C	3358	5.01093001	8.58E-23	2.39E-21
667	ENSG00000258947.7	TUBB3	10381	2.039577416	9.93E-23	2.75E-21
668	ENSG00000133107.15	TRPC4	7223	1.855624474	9.95E-23	2.76E-21
669	ENSG00000248458.2			4.212087057	1.07E-22	2.95E-21
670	ENSG00000122952.17	ZWINT	11130	1.096099938	1.08E-22	2.97E-21
671	ENSG00000072571.20	HMMR	3161	1.32904239	1.08E-22	2.97E-21
672	ENSG00000135114.12	OASL	8638	2.436330002	1.11E-22	3.04E-21
673	ENSG00000182612.10	TSPAN10	83882	2.087166724	1.14E-22	3.13E-21
674	ENSG00000114115.10	RBP1	5947	2.066936423	1.14E-22	3.13E-21
675	ENSG00000175600.16	SUGCT	79783	1.859841133	1.15E-22	3.16E-21
676	ENSG00000156234.7	CXCL13	10563	2.508173008	1.19E-22	3.27E-21
677	ENSG00000127561.15	SYNGR3	9143	2.651732077	1.20E-22	3.27E-21
678	ENSG00000254417.1	LINC02584		3.74115027	1.30E-22	3.54E-21
679	ENSG00000286878.1			2.990253132	1.40E-22	3.80E-21
680	ENSG00000271134.1	IFITM3P9		2.347677329	1.45E-22	3.94E-21
681	ENSG00000101194.18	SLC17A9	63910	1.983739668	1.45E-22	3.94E-21
682	ENSG00000258116.1	PPIAP45		2.203557039	1.62E-22	4.38E-21
683	ENSG00000123975.5	CKS2	1164	1.310099254	1.65E-22	4.45E-21
684	ENSG00000226659.1	TSPAN14-AS1		2.198479786	1.70E-22	4.59E-21
685	ENSG00000179715.13	PCED1B	91523	1.332856064	1.80E-22	4.84E-21
686	ENSG00000090932.11	DLL3	10683	2.931199524	1.84E-22	4.95E-21
687	ENSG00000197587.11	DMBX1	127343	3.490507979	1.89E-22	5.07E-21
688	ENSG00000163364.10	LINC01116	375295	2.166924578	1.91E-22	5.13E-21
689	ENSG00000182912.6			3.874544307	1.92E-22	5.15E-21
690	ENSG00000134321.12	RSAD2	91543	2.453919074	2.01E-22	5.38E-21
691	ENSG00000177359.20			3.219602887	2.14E-22	5.72E-21
692	ENSG00000282851.2	BISPR	105221694	1.544365873	2.31E-22	6.15E-21
693	ENSG00000162702.8	ZNF281	23528	1.011786698	2.39E-22	6.36E-21
694	ENSG00000147119.4	CHST7	56548	1.724908247	2.49E-22	6.59E-21
695	ENSG00000092067.6	CEBPE	1053	3.326790355	2.60E-22	6.89E-21
696	ENSG00000236064.2		101928335	2.030949855	2.63E-22	6.96E-21
697	ENSG00000012048.23	BRCA1	672	1.068840601	2.82E-22	7.45E-21
698	ENSG00000182574.8	CSGALNACT2P1		2.600639658	3.01E-22	7.93E-21
699	ENSG00000183971.10	NPW	283869	3.159599332	3.09E-22	8.11E-21
700	ENSG00000111700.13	SLCO1B3	28234	3.903496219	3.11E-22	8.16E-21
701	ENSG00000234380.2	LINC01426	100506385	2.388309072	3.11E-22	8.16E-21
702	ENSG00000245149.4	RNF139-DT	101927612	1.326272877	3.25E-22	8.52E-21
703	ENSG00000267577.1	DNAAF3-AS1		4.430725007	3.29E-22	8.62E-21
704	ENSG00000224402.2	OR6D1P		3.113768815	3.41E-22	8.90E-21
705	ENSG00000228295.2	LINC00392		6.480627425	3.45E-22	9.00E-21
706	ENSG00000285278.1	TFAP2A-AS2	109729173	1.487670259	3.56E-22	9.28E-21
707	ENSG00000168542.16	COL3A1	1281	2.542584136	3.63E-22	9.47E-21
708	ENSG00000109790.16	KLHL5	51088	1.082796108	3.73E-22	9.71E-21
709	ENSG00000184661.14	CDCA2	157313	1.292175411	3.78E-22	9.84E-21
710	ENSG00000260757.1			3.519940334	3.81E-22	9.90E-21
711	ENSG00000226419.8	SLC16A1-AS1	100506392	1.151717061	3.85E-22	9.99E-21
712	ENSG00000145247.12	OCIAD2	132299	1.284490617	3.95E-22	1.03E-20
713	ENSG00000167123.19	CERCAM	51148	1.709286758	4.01E-22	1.04E-20
714	ENSG00000227674.3	LINC00355	144766	6.318452208	4.17E-22	1.08E-20
715	ENSG00000179406.8	LINC00174	285908	1.689131811	4.23E-22	1.09E-20
716	ENSG00000013573.17	DDX11	1663	1.187692613	4.45E-22	1.15E-20
717	ENSG00000156509.14	FBXO43	286151	2.095915965	4.51E-22	1.16E-20
718	ENSG00000249916.1	SNCAIP-AS1		3.227772671	4.54E-22	1.17E-20
719	ENSG00000166342.19	NETO1	81832	3.408126388	5.01E-22	1.28E-20
720	ENSG00000136518.17	ACTL6A	86	1.049955625	5.71E-22	1.46E-20
721	ENSG00000141012.13	GALNS	2588	1.089882826	6.16E-22	1.57E-20
722	ENSG00000234948.2	LINC01524	101927700	3.087215849	6.33E-22	1.61E-20
723	ENSG00000167618.10	LAIR2	3904	2.281325988	6.65E-22	1.69E-20
724	ENSG00000168743.13	NPNT	255743	1.7457754	6.65E-22	1.69E-20
725	ENSG00000162723.10	SLAMF9	89886	2.905229763	7.04E-22	1.79E-20
726	ENSG00000125319.15	HROB	78995	1.234496994	7.43E-22	1.88E-20
727	ENSG00000221968.9	FADS3	3995	1.773897891	7.61E-22	1.93E-20
728	ENSG00000011426.11	ANLN	54443	1.329325467	7.87E-22	1.99E-20
729	ENSG00000261136.1			2.288590309	8.01E-22	2.02E-20
730	ENSG00000186871.7	ERCC6L	54821	1.184753478	9.17E-22	2.31E-20
731	ENSG00000258018.2	LINC02457		2.805305559	9.45E-22	2.38E-20
732	ENSG00000143322.21	ABL2	27	1.08278493	1.01E-21	2.53E-20
733	ENSG00000097021.20	ACOT7	11332	1.165096218	1.03E-21	2.57E-20
734	ENSG00000261488.1	TBILA	112806053	1.815354459	1.04E-21	2.59E-20
735	ENSG00000120254.16	MTHFD1L	25902	1.137404406	1.04E-21	2.61E-20
736	ENSG00000156970.13	BUB1B	701	1.220425999	1.06E-21	2.64E-20
737	ENSG00000137809.17	ITGA11	22801	2.52768873	1.09E-21	2.73E-20
738	ENSG00000159261.12	CLDN14	23562	2.6336833	1.15E-21	2.85E-20
739	ENSG00000279692.1			2.021009701	1.19E-21	2.96E-20
740	ENSG00000287394.1	MAGEA3-DT		5.950330032	1.23E-21	3.07E-20
741	ENSG00000249102.3			4.478159915	1.27E-21	3.16E-20
742	ENSG00000258806.2	OR11H7	390441	3.674967392	1.27E-21	3.16E-20
743	ENSG00000148680.16	HTR7	3363	2.399632564	1.30E-21	3.22E-20
744	ENSG00000185697.16	MYBL1	4603	1.397170553	1.33E-21	3.29E-20
745	ENSG00000272071.1			4.097822855	1.33E-21	3.30E-20
746	ENSG00000174332.5	GLIS1	148979	2.269315916	1.36E-21	3.37E-20
747	ENSG00000186897.5	C1QL4	338761	3.249742866	1.42E-21	3.50E-20
748	ENSG00000234902.6			2.38211511	1.44E-21	3.55E-20
749	ENSG00000262943.7	ALOX12P2		1.913490983	1.56E-21	3.82E-20
750	ENSG00000112984.12	KIF20A	10112	1.266819888	1.58E-21	3.87E-20
751	ENSG00000144130.11	NT5DC4	284958	3.156977838	1.59E-21	3.89E-20
752	ENSG00000049249.9	TNFRSF9	3604	1.874429725	1.66E-21	4.06E-20
753	ENSG00000105889.16	STEAP1B	256227	2.73703539	1.75E-21	4.29E-20
754	ENSG00000075420.13	FNDC3B	64778	1.148855211	1.83E-21	4.48E-20
755	ENSG00000162344.4	FGF19	9965	5.63005434	1.93E-21	4.72E-20
756	ENSG00000273004.1			1.489236265	2.00E-21	4.89E-20
757	ENSG00000259756.1			4.912330297	2.07E-21	5.04E-20
758	ENSG00000253154.2			4.25144827	2.12E-21	5.14E-20
759	ENSG00000259712.1			2.348044287	2.15E-21	5.22E-20
760	ENSG00000147889.18	CDKN2A	1029	3.323340139	2.19E-21	5.29E-20
761	ENSG00000149636.16	DSN1	79980	1.00166913	2.29E-21	5.53E-20
762	ENSG00000146263.11	MMS22L	253714	1.001608879	2.30E-21	5.56E-20
763	ENSG00000106819.13	ASPN	54829	2.610462147	2.36E-21	5.69E-20
764	ENSG00000276115.1			1.613085676	2.37E-21	5.72E-20
765	ENSG00000102195.10	GPR50	9248	5.194072474	2.52E-21	6.05E-20
766	ENSG00000250546.6	LINC02994	101928978	3.252161371	2.53E-21	6.09E-20
767	ENSG00000205885.7	C1RL-AS1	283314	1.353513103	2.57E-21	6.16E-20
768	ENSG00000086991.13	NOX4	50507	1.777845289	2.67E-21	6.39E-20
769	ENSG00000145506.14	NKD2	85409	1.965215927	2.67E-21	6.40E-20
770	ENSG00000130508.11	PXDN	7837	1.95491702	2.75E-21	6.57E-20
771	ENSG00000243537.1	RPL32P20		2.948116197	2.77E-21	6.61E-20
772	ENSG00000221867.9	MAGEA3	4102	5.673611596	2.79E-21	6.66E-20
773	ENSG00000162676.12	GFI1	2672	1.840938234	2.83E-21	6.75E-20
774	ENSG00000010292.13	NCAPD2	9918	1.109477773	2.95E-21	7.03E-20
775	ENSG00000116962.15	NID1	4811	1.952698457	2.96E-21	7.04E-20
776	ENSG00000186827.11	TNFRSF4	7293	1.638030466	3.02E-21	7.19E-20
777	ENSG00000142156.15	COL6A1	1291	1.944297188	3.09E-21	7.34E-20
778	ENSG00000255535.2			4.629495364	3.11E-21	7.39E-20
779	ENSG00000169856.9	ONECUT1	3175	3.822815157	3.11E-21	7.39E-20
780	ENSG00000164651.17	SP8	221833	3.963205964	3.45E-21	8.16E-20
781	ENSG00000198681.7	MAGEA1	4100	7.710013697	3.51E-21	8.31E-20
782	ENSG00000269976.1			2.890802556	3.95E-21	9.31E-20
783	ENSG00000224116.7	INHBA-AS1	285954	2.761045717	4.07E-21	9.57E-20
784	ENSG00000133816.17	MICAL2	9645	1.629255933	4.08E-21	9.60E-20
785	ENSG00000164692.18	COL1A2	1278	2.405296564	4.35E-21	1.02E-19
786	ENSG00000248112.1		105379051	5.100126671	4.61E-21	1.08E-19
787	ENSG00000134057.15	CCNB1	891	1.145137851	4.84E-21	1.13E-19
788	ENSG00000262503.1	SPICP3		2.699825885	4.99E-21	1.17E-19
789	ENSG00000232023.3	LINC01807	101928765	5.253614094	5.19E-21	1.21E-19
790	ENSG00000269586.7	CT45A10	441521	9.257119597	5.27E-21	1.23E-19
791	ENSG00000117632.23	STMN1	3925	1.184589487	5.59E-21	1.30E-19
792	ENSG00000269927.1			3.672335567	5.64E-21	1.31E-19
793	ENSG00000164220.7	F2RL2	2151	2.154068305	5.69E-21	1.32E-19
794	ENSG00000225362.10	CT62	196993	3.426619438	5.91E-21	1.37E-19
795	ENSG00000122642.11	FKBP9	11328	1.084705606	5.92E-21	1.37E-19
796	ENSG00000255624.3	C10orf88B		2.20278785	6.16E-21	1.42E-19
797	ENSG00000179059.10	ZFP42	132625	5.092285486	6.33E-21	1.46E-19
798	ENSG00000256980.5	KHDC1L	100129128	4.628650514	6.38E-21	1.47E-19
799	ENSG00000229261.1		101928994	4.204925836	6.44E-21	1.48E-19
800	ENSG00000005884.18	ITGA3	3675	1.645653666	6.49E-21	1.49E-19
801	ENSG00000157766.19	ACAN	176	2.305864273	6.66E-21	1.53E-19
802	ENSG00000226421.1	SLC25A5P5		2.216085994	6.79E-21	1.56E-19
803	ENSG00000271947.1			3.018653308	7.11E-21	1.63E-19
804	ENSG00000233515.2	LINC01518	101929397	3.85114558	7.71E-21	1.76E-19
805	ENSG00000103154.9	NECAB2	54550	3.245791269	8.61E-21	1.96E-19
806	ENSG00000133106.15	EPSTI1	94240	1.94828338	8.70E-21	1.98E-19
807	ENSG00000068079.8	IFI35	3430	1.61532594	8.85E-21	2.02E-19
808	ENSG00000163915.7	IGF2BP2-AS1	646600	2.873449839	9.25E-21	2.10E-19
809	ENSG00000225764.2	P3H2-AS1		3.131433841	9.41E-21	2.14E-19
810	ENSG00000132470.14	ITGB4	3691	1.308837781	9.52E-21	2.16E-19
811	ENSG00000215493.3	KLHL12P1		1.987060709	9.70E-21	2.20E-19
812	ENSG00000180178.11	FAR2P1		5.859497028	9.71E-21	2.20E-19
813	ENSG00000181381.14	DDX60L	91351	1.558594067	9.87E-21	2.24E-19
814	ENSG00000233922.3	LINC01694	105372840	3.55596412	1.00E-20	2.27E-19
815	ENSG00000250261.1	CCDC74BP1		4.393124696	1.02E-20	2.31E-19
816	ENSG00000256546.2	LINC02985		3.128097368	1.09E-20	2.46E-19
817	ENSG00000174945.13	AMZ1	155185	2.466325382	1.13E-20	2.54E-19
818	ENSG00000155886.11	SLC24A2	25769	2.41538012	1.16E-20	2.60E-19
819	ENSG00000145934.16	TENM2	57451	2.245940184	1.18E-20	2.65E-19
820	ENSG00000101000.6	PROCR	10544	1.622564484	1.21E-20	2.72E-19
821	ENSG00000266869.2			3.168373437	1.34E-20	3.00E-19
822	ENSG00000248347.3			4.252532174	1.41E-20	3.15E-19
823	ENSG00000171488.15	LRRC8C	84230	1.283689117	1.44E-20	3.23E-19
824	ENSG00000152952.12	PLOD2	5352	1.517417088	1.46E-20	3.26E-19
825	ENSG00000234147.1			2.874073918	1.46E-20	3.27E-19
826	ENSG00000197903.8	H2BC12	85236	1.556797713	1.47E-20	3.29E-19
827	ENSG00000236651.1	DLX2-DT	104326193	3.399580573	1.56E-20	3.48E-19
828	ENSG00000111335.13	OAS2	4939	1.726717931	1.66E-20	3.69E-19
829	ENSG00000272476.1			1.330488165	1.90E-20	4.20E-19
830	ENSG00000213186.8	TRIM59	286827	1.19004868	1.94E-20	4.27E-19
831	ENSG00000268041.2	ERFL	390937	2.009034592	1.94E-20	4.28E-19
832	ENSG00000183840.7	GPR39	2863	2.121834954	2.16E-20	4.76E-19
833	ENSG00000166670.10	MMP10	4319	3.280591852	2.31E-20	5.07E-19
834	ENSG00000110328.6	GALNT18	374378	1.200292738	2.33E-20	5.11E-19
835	ENSG00000189052.7	CGB5	93659	4.701862182	2.47E-20	5.41E-19
836	ENSG00000287944.1			3.510964478	2.50E-20	5.47E-19
837	ENSG00000057019.16	DCBLD2	131566	1.479739129	2.62E-20	5.75E-19
838	ENSG00000234997.2	LINC02966		2.043564875	2.63E-20	5.76E-19
839	ENSG00000095970.17	TREM2	54209	1.953612265	2.67E-20	5.84E-19
840	ENSG00000287543.1			1.705915144	2.82E-20	6.12E-19
841	ENSG00000189410.12	SH2D5	400745	2.591011269	3.24E-20	7.02E-19
842	ENSG00000137273.6	FOXF2	2295	1.503985165	3.46E-20	7.47E-19
843	ENSG00000072682.19	P4HA2	8974	1.275337171	3.59E-20	7.72E-19
844	ENSG00000147065.17	MSN	4478	1.161505215	3.73E-20	7.99E-19
845	ENSG00000287390.1			3.777678989	3.75E-20	8.04E-19
846	ENSG00000138944.8	SHISAL1	85352	2.355017848	4.20E-20	8.95E-19
847	ENSG00000124243.17	BCAS4	55653	1.568616356	4.32E-20	9.19E-19
848	ENSG00000155380.12	SLC16A1	6566	1.294090121	4.46E-20	9.49E-19
849	ENSG00000135638.14	EMX1	2016	2.720663223	4.46E-20	9.49E-19
850	ENSG00000224999.1	VTA1P1		5.754100407	4.47E-20	9.50E-19
851	ENSG00000041982.16	TNC	3371	2.105828043	4.70E-20	9.97E-19
852	ENSG00000228952.1	LINC02041		3.300071081	4.74E-20	1.00E-18
853	ENSG00000128683.14	GAD1	2571	2.463019242	4.83E-20	1.02E-18
854	ENSG00000259768.6	ZFHX3-AS1		1.120259094	4.97E-20	1.05E-18
855	ENSG00000151790.9	TDO2	6999	2.240029559	4.98E-20	1.05E-18
856	ENSG00000237423.3	LINC01522		3.345242557	5.02E-20	1.06E-18
857	ENSG00000175003.15	SLC22A1	6580	2.78173566	5.27E-20	1.11E-18
858	ENSG00000170581.14	STAT2	6773	1.053114352	5.32E-20	1.12E-18
859	ENSG00000257500.1			3.63593389	5.52E-20	1.16E-18
860	ENSG00000254521.7	SIGLEC12	89858	2.592441471	5.53E-20	1.17E-18
861	ENSG00000230524.8	COL6A4P1		2.127214507	5.63E-20	1.18E-18
862	ENSG00000287407.1			3.803571902	5.75E-20	1.21E-18
863	ENSG00000175130.7	MARCKSL1	65108	1.243174461	5.78E-20	1.21E-18
864	ENSG00000163013.12	FBXO41	150726	1.331609797	5.82E-20	1.22E-18
865	ENSG00000278709.2	NKILA	105416157	2.033941297	5.98E-20	1.25E-18
866	ENSG00000174792.11	ODAPH	152816	3.321376739	6.02E-20	1.26E-18
867	ENSG00000137573.14	SULF1	23213	2.080304903	6.03E-20	1.26E-18
868	ENSG00000113140.11	SPARC	6678	1.872653581	6.19E-20	1.30E-18
869	ENSG00000237445.2			2.847033557	6.54E-20	1.37E-18
870	ENSG00000185615.16	PDIA2	64714	3.81357977	6.75E-20	1.41E-18
871	ENSG00000214826.5	DDX12P		1.538127995	6.88E-20	1.43E-18
872	ENSG00000277511.1			1.734167728	7.19E-20	1.50E-18
873	ENSG00000161647.19	MPP3	4356	1.242354568	7.45E-20	1.55E-18
874	ENSG00000046774.10	MAGEC2	51438	7.941260024	7.93E-20	1.65E-18
875	ENSG00000279529.1			1.541402316	8.13E-20	1.69E-18
876	ENSG00000189366.9	ALG1L1P		1.78358532	8.51E-20	1.76E-18
877	ENSG00000049323.16	LTBP1	4052	1.726151479	8.56E-20	1.77E-18
878	ENSG00000251577.5	TACR3-AS1		5.040531844	8.88E-20	1.83E-18
879	ENSG00000224969.1			2.831195616	9.08E-20	1.87E-18
880	ENSG00000278023.7	RDM1	201299	2.069189609	9.35E-20	1.93E-18
881	ENSG00000249500.1	LINC01179	101928151	4.811631915	9.83E-20	2.02E-18
882	ENSG00000267147.2	LINC01842		2.63748777	9.90E-20	2.03E-18
883	ENSG00000180264.11	ADGRD2	347088	2.338376019	1.05E-19	2.16E-18
884	ENSG00000225783.8	MIAT	440823	2.238865301	1.05E-19	2.16E-18
885	ENSG00000143641.10	GALNT2	2590	1.013223112	1.07E-19	2.18E-18
886	ENSG00000226779.1	NAALADL2-AS2		5.116696091	1.09E-19	2.22E-18
887	ENSG00000273272.2			2.224725043	1.11E-19	2.27E-18
888	ENSG00000286223.1			1.828031953	1.16E-19	2.37E-18
889	ENSG00000145386.11	CCNA2	890	1.120660724	1.18E-19	2.41E-18
890	ENSG00000254968.7	LINC02763	101928823	3.160252958	1.19E-19	2.42E-18
891	ENSG00000287235.1		105376381	2.558451214	1.25E-19	2.55E-18
892	ENSG00000104341.17	LAPTM4B	55353	1.011863706	1.26E-19	2.56E-18
893	ENSG00000279296.1	PRAL		2.46700565	1.27E-19	2.58E-18
894	ENSG00000164304.16	CAGE1	285782	1.547675601	1.28E-19	2.59E-18
895	ENSG00000169071.15	ROR2	4920	2.006518415	1.28E-19	2.59E-18
896	ENSG00000170955.10	CAVIN3	112464	1.653409207	1.32E-19	2.69E-18
897	ENSG00000215270.3	TOMM40P2		2.560890659	1.41E-19	2.86E-18
898	ENSG00000005448.17	WDR54	84058	1.327836436	1.45E-19	2.94E-18
899	ENSG00000284526.1		122455342	1.449078677	1.50E-19	3.02E-18
900	ENSG00000163923.10	RPL39L	116832	2.009862508	1.54E-19	3.10E-18
901	ENSG00000232855.7	LINC03138		2.881734022	1.55E-19	3.11E-18
902	ENSG00000257167.2	TMPO-AS1	100128191	1.329252385	1.56E-19	3.13E-18
903	ENSG00000135454.14	B4GALNT1	2583	2.042352399	1.57E-19	3.16E-18
904	ENSG00000165643.11	SOHLH1	402381	6.453838948	1.58E-19	3.18E-18
905	ENSG00000185269.12	NOTUM	147111	2.278303559	1.59E-19	3.20E-18
906	ENSG00000218336.9	TENM3	55714	2.036911167	1.61E-19	3.24E-18
907	ENSG00000186684.14	CYP27C1	339761	2.044332342	1.75E-19	3.50E-18
908	ENSG00000261241.7	LINC02128	102724940	2.918069229	1.79E-19	3.59E-18
909	ENSG00000184108.8	TRIML1	339976	4.032218061	1.80E-19	3.60E-18
910	ENSG00000260302.3	LINC01882		2.904907567	1.81E-19	3.62E-18
911	ENSG00000136943.11	CTSV	1515	1.816061064	1.82E-19	3.63E-18
912	ENSG00000254842.6	LINC02551		2.802964155	1.86E-19	3.72E-18
913	ENSG00000172965.17	MIR4435-2HG	541471	1.040734723	1.88E-19	3.75E-18
914	ENSG00000241288.8	LINC02614	101927056	1.771040386	1.91E-19	3.81E-18
915	ENSG00000130427.3	EPO	2056	2.575968028	1.92E-19	3.81E-18
916	ENSG00000271826.5	PLS3-AS1	101927352	1.799086252	1.93E-19	3.84E-18
917	ENSG00000159184.8	HOXB13	10481	4.322056383	1.95E-19	3.88E-18
918	ENSG00000231453.1	LINC01305	285084	3.7991005	1.96E-19	3.88E-18
919	ENSG00000143324.14	XPR1	9213	1.01437337	1.97E-19	3.90E-18
920	ENSG00000196470.12	SIAH1	6477	1.009456944	2.09E-19	4.13E-18
921	ENSG00000139618.16	BRCA2	675	1.210745632	2.14E-19	4.23E-18
922	ENSG00000257925.1			3.180345125	2.27E-19	4.48E-18
923	ENSG00000287188.1			2.530788666	2.34E-19	4.62E-18
924	ENSG00000104147.9	OIP5	11339	1.228140896	2.52E-19	4.94E-18
925	ENSG00000140093.10	SERPINA10	51156	3.238658506	2.59E-19	5.09E-18
926	ENSG00000188483.8	IER5L	389792	1.231103691	2.62E-19	5.14E-18
927	ENSG00000112877.8	CEP72	55722	1.061147355	2.65E-19	5.20E-18
928	ENSG00000259240.1	MIR4713HG	109729174	1.639604195	2.73E-19	5.36E-18
929	ENSG00000245522.2	LINC02709	440028	1.360289191	2.79E-19	5.47E-18
930	ENSG00000240990.10	HOXA11-AS	221883	4.13055321	2.81E-19	5.50E-18
931	ENSG00000205704.7	SMIM45	339674	2.72138335	2.96E-19	5.78E-18
932	ENSG00000240476.1	LINC00973	105374003	4.603452919	3.00E-19	5.86E-18
933	ENSG00000137474.22	MYO7A	4647	1.389506526	3.11E-19	6.06E-18
934	ENSG00000250509.2			3.383290681	3.18E-19	6.19E-18
935	ENSG00000261649.6	GOLGA6L7	728310	5.632196815	3.60E-19	6.99E-18
936	ENSG00000176208.9	ATAD5	79915	1.028859487	3.71E-19	7.19E-18
937	ENSG00000267978.5	MAGEA9B	4108	7.607674705	3.94E-19	7.62E-18
938	ENSG00000232508.1	MRPL45P1		4.386217754	4.03E-19	7.79E-18
939	ENSG00000286966.1			3.842178919	4.19E-19	8.08E-18
940	ENSG00000174827.14	PDZK1	5174	2.56662636	4.20E-19	8.10E-18
941	ENSG00000073146.16	MOV10L1	54456	2.132168763	4.40E-19	8.46E-18
942	ENSG00000284948.1		254896	3.71351868	4.40E-19	8.46E-18
943	ENSG00000137812.20	KNL1	57082	1.133813324	4.46E-19	8.58E-18
944	ENSG00000196074.13	SYCP2	10388	3.100863083	4.50E-19	8.64E-18
945	ENSG00000225383.8	SFTA1P	207107	3.054180994	4.51E-19	8.66E-18
946	ENSG00000238113.7	LINC01410	442421	1.326118978	4.78E-19	9.17E-18
947	ENSG00000066279.18	ASPM	259266	1.196693487	4.84E-19	9.27E-18
948	ENSG00000236216.5	PPP1R11P1		4.131505405	4.98E-19	9.52E-18
949	ENSG00000165553.4	NGB	58157	4.216876941	5.44E-19	1.04E-17
950	ENSG00000188343.13	CIBAR1	137392	1.001230214	5.52E-19	1.05E-17
951	ENSG00000125885.13	MCM8	84515	1.023339578	5.53E-19	1.05E-17
952	ENSG00000288060.1			3.760980885	5.61E-19	1.07E-17
953	ENSG00000243321.3	LINC01998	107986046	4.120413564	5.77E-19	1.10E-17
954	ENSG00000136193.17	SCRN1	9805	1.049080828	6.32E-19	1.20E-17
955	ENSG00000251361.1			3.672107603	6.32E-19	1.20E-17
956	ENSG00000255026.1			2.218466567	6.37E-19	1.21E-17
957	ENSG00000245614.4	DDX11-AS1	100506660	1.728660801	6.38E-19	1.21E-17
958	ENSG00000087303.18	NID2	22795	2.077530295	6.48E-19	1.23E-17
959	ENSG00000223511.7	LINC02968		2.35596168	6.55E-19	1.24E-17
960	ENSG00000163599.17	CTLA4	1493	1.795765878	7.05E-19	1.33E-17
961	ENSG00000161653.11	NAGS	162417	1.39240255	7.10E-19	1.34E-17
962	ENSG00000251550.1			4.230050838	7.16E-19	1.35E-17
963	ENSG00000146216.13	TTBK1	84630	1.91654591	7.32E-19	1.38E-17
964	ENSG00000231298.7	MANCR	100216001	2.453446335	7.59E-19	1.43E-17
965	ENSG00000163463.13	KRTCAP2	200185	1.459370954	7.78E-19	1.46E-17
966	ENSG00000260289.2			5.050737244	7.80E-19	1.47E-17
967	ENSG00000119917.14	IFIT3	3437	2.093566013	8.43E-19	1.58E-17
968	ENSG00000109511.12	ANXA10	11199	3.501098773	8.44E-19	1.58E-17
969	ENSG00000237978.6	KCNMB2-AS1		2.233038354	8.60E-19	1.61E-17
970	ENSG00000182022.18	CHST15	51363	1.1295654	9.54E-19	1.78E-17
971	ENSG00000227992.1			1.659606838	9.67E-19	1.80E-17
972	ENSG00000278266.1			2.469553475	1.01E-18	1.88E-17
973	ENSG00000249641.2	HOXC13-AS	100874366	2.429490291	1.02E-18	1.90E-17
974	ENSG00000089327.15	FXYD5	53827	1.5479284	1.05E-18	1.95E-17
975	ENSG00000137959.16	IFI44L	10964	2.187085755	1.07E-18	1.98E-17
976	ENSG00000285831.1			2.567688008	1.12E-18	2.08E-17
977	ENSG00000144810.16	COL8A1	1295	2.180191526	1.14E-18	2.10E-17
978	ENSG00000248370.2	LINC02434		5.118934186	1.14E-18	2.10E-17
979	ENSG00000118473.22	SGIP1	84251	1.657151437	1.23E-18	2.26E-17
980	ENSG00000228697.4		101928565	3.256907771	1.34E-18	2.46E-17
981	ENSG00000119922.10	IFIT2	3433	2.053195081	1.36E-18	2.49E-17
982	ENSG00000248898.3	PELO-AS1	125775243	2.520577596	1.39E-18	2.56E-17
983	ENSG00000275713.2	H2BC9	8345	1.622629661	1.41E-18	2.59E-17
984	ENSG00000171608.16	PIK3CD	5293	1.178843748	1.45E-18	2.65E-17
985	ENSG00000109705.8	NKX3-2	579	2.476677126	1.50E-18	2.74E-17
986	ENSG00000253304.2	TMEM200B	399474	1.538141942	1.52E-18	2.79E-17
987	ENSG00000196979.1	GPRACR	401554	3.081735963	1.57E-18	2.87E-17
988	ENSG00000160207.9	HSF2BP	11077	1.488983918	1.58E-18	2.88E-17
989	ENSG00000266602.2			5.144448181	1.61E-18	2.93E-17
990	ENSG00000154451.14	GBP5	115362	2.614777859	1.65E-18	3.01E-17
991	ENSG00000219375.1			2.419104964	1.77E-18	3.22E-17
992	ENSG00000288611.1	NPBWR1	2831	2.955165941	1.79E-18	3.26E-17
993	ENSG00000168040.5	FADD	8772	1.766316609	1.80E-18	3.27E-17
994	ENSG00000120068.7	HOXB8	3218	3.325906163	1.80E-18	3.27E-17
995	ENSG00000100867.15	DHRS2	10202	2.81530234	1.85E-18	3.35E-17
996	ENSG00000257844.2	KRT90P		4.051544006	1.95E-18	3.53E-17
997	ENSG00000231240.1	KLF2P1		5.636231848	1.95E-18	3.53E-17
998	ENSG00000248268.1			3.786841548	2.08E-18	3.76E-17
999	ENSG00000272872.1			2.835282984	2.11E-18	3.81E-17
1000	ENSG00000136280.17	CCM2	83605	1.115099995	2.15E-18	3.88E-17
1001	ENSG00000251151.2	HOXC-AS3		4.030038307	2.22E-18	4.01E-17
1002	ENSG00000147536.12	GINS4	84296	1.343719723	2.23E-18	4.01E-17
1003	ENSG00000176406.23	RIMS2	9699	2.313680096	2.25E-18	4.06E-17
1004	ENSG00000106588.12	PSMA2	5683	1.537084997	2.34E-18	4.22E-17
1005	ENSG00000140937.14	CDH11	1009	2.007208249	2.42E-18	4.34E-17
1006	ENSG00000171587.15	DSCAM	1826	3.227420954	2.44E-18	4.38E-17
1007	ENSG00000116745.7	RPE65	6121	3.757959297	2.60E-18	4.65E-17
1008	ENSG00000287424.1			5.021376953	2.60E-18	4.65E-17
1009	ENSG00000168461.13	RAB31	11031	1.162750543	2.71E-18	4.84E-17
1010	ENSG00000225146.1			3.904002486	2.80E-18	4.99E-17
1011	ENSG00000255282.6	WTAPP1		2.369010204	2.85E-18	5.08E-17
1012	ENSG00000171388.12	APLN	8862	1.806911759	2.98E-18	5.30E-17
1013	ENSG00000185904.12	LINC00839	84856	2.54841415	3.08E-18	5.47E-17
1014	ENSG00000136114.17	THSD1	55901	1.694796055	3.11E-18	5.53E-17
1015	ENSG00000275437.1			1.453962189	3.12E-18	5.54E-17
1016	ENSG00000182631.7	RXFP3	51289	3.588193692	3.14E-18	5.58E-17
1017	ENSG00000284194.3	SCO2	9997	2.285255044	3.28E-18	5.81E-17
1018	ENSG00000179750.16	APOBEC3B	9582	1.793339941	3.35E-18	5.92E-17
1019	ENSG00000122136.14	OBP2A	29991	3.291100598	3.37E-18	5.97E-17
1020	ENSG00000235890.2	TSPEAR-AS1		3.386957339	3.82E-18	6.73E-17
1021	ENSG00000074219.14	TEAD2	8463	1.083355443	4.18E-18	7.34E-17
1022	ENSG00000148773.14	MKI67	4288	1.140970926	4.22E-18	7.41E-17
1023	ENSG00000249199.2			4.672590722	4.23E-18	7.41E-17
1024	ENSG00000172322.14	CLEC12A	160364	1.972765922	4.38E-18	7.66E-17
1025	ENSG00000109861.17	CTSC	1075	1.293739231	4.40E-18	7.69E-17
1026	ENSG00000198915.12	RASGEF1A	221002	2.192824891	4.78E-18	8.34E-17
1027	ENSG00000231638.1	LUARIS	100506895	2.485172983	5.06E-18	8.81E-17
1028	ENSG00000258938.1			1.479603937	5.07E-18	8.82E-17
1029	ENSG00000145014.18	TMEM44	93109	1.229412125	5.13E-18	8.92E-17
1030	ENSG00000128595.17	CALU	813	1.004567918	5.30E-18	9.19E-17
1031	ENSG00000225964.6	NRIR		2.403611911	5.34E-18	9.27E-17
1032	ENSG00000234155.1	LINC02535	101928820	2.569738917	5.59E-18	9.68E-17
1033	ENSG00000279712.1			2.739460997	5.65E-18	9.78E-17
1034	ENSG00000006327.14	TNFRSF12A	51330	1.421963522	5.96E-18	1.03E-16
1035	ENSG00000229967.1	MAGEA4-AS1		6.053070986	6.27E-18	1.08E-16
1036	ENSG00000124575.7	H1-3	3007	2.256731241	6.52E-18	1.12E-16
1037	ENSG00000115844.11	DLX2	1746	2.366283786	6.55E-18	1.13E-16
1038	ENSG00000232233.1	LINC02043	102724699	2.681218586	6.57E-18	1.13E-16
1039	ENSG00000120659.15	TNFSF11	8600	2.09101066	6.71E-18	1.15E-16
1040	ENSG00000196421.8	C20orf204	284739	1.954558553	7.56E-18	1.29E-16
1041	ENSG00000271216.2	LINC01050		3.006776203	7.63E-18	1.30E-16
1042	ENSG00000175879.9	HOXD8	3234	1.292554296	7.76E-18	1.33E-16
1043	ENSG00000132330.18	SCLY	51540	1.03752	7.90E-18	1.35E-16
1044	ENSG00000279495.1			1.311168634	8.21E-18	1.40E-16
1045	ENSG00000263513.5	FAM72C	554282	1.498769611	8.31E-18	1.42E-16
1046	ENSG00000259306.2			1.886815256	8.60E-18	1.46E-16
1047	ENSG00000189212.12	DPY19L2P1		4.193117904	8.72E-18	1.48E-16
1048	ENSG00000160957.14	RECQL4	9401	1.23931615	9.12E-18	1.55E-16
1049	ENSG00000089692.9	LAG3	3902	2.064728844	9.20E-18	1.56E-16
1050	ENSG00000011083.9	SLC6A7	6534	2.317180289	9.21E-18	1.56E-16
1051	ENSG00000164930.12	FZD6	8323	1.02681083	9.38E-18	1.59E-16
1052	ENSG00000106348.18	IMPDH1	3614	1.073654343	9.46E-18	1.60E-16
1053	ENSG00000284753.2	EEF1AKMT4	110599564	1.223335129	9.81E-18	1.66E-16
1054	ENSG00000197299.12	BLM	641	1.021927235	9.84E-18	1.66E-16
1055	ENSG00000165480.16	SKA3	221150	1.020623244	1.04E-17	1.75E-16
1056	ENSG00000262874.2	C19orf84	147646	2.659829024	1.04E-17	1.76E-16
1057	ENSG00000246130.1	TNFRSF10B-AS1	286059	2.288202714	1.10E-17	1.85E-16
1058	ENSG00000147324.11	MFHAS1	9258	1.052295507	1.10E-17	1.86E-16
1059	ENSG00000258053.1			3.784768537	1.12E-17	1.88E-16
1060	ENSG00000255921.1	BCAT1-DT		3.636731738	1.13E-17	1.90E-16
1061	ENSG00000182632.15	CCNYL2		4.515271144	1.14E-17	1.92E-16
1062	ENSG00000204296.11	TSBP1	10665	2.883794028	1.17E-17	1.96E-16
1063	ENSG00000111331.13	OAS3	4940	1.552736815	1.20E-17	2.01E-16
1064	ENSG00000182393.3	IFNL1	282618	3.365279888	1.20E-17	2.02E-16
1065	ENSG00000129990.15	SYT5	6861	2.880665949	1.31E-17	2.19E-16
1066	ENSG00000228649.9	SNHG26		1.439691057	1.31E-17	2.20E-16
1067	ENSG00000287349.1			2.347786825	1.34E-17	2.23E-16
1068	ENSG00000169427.8	KCNK9	51305	2.445679833	1.40E-17	2.34E-16
1069	ENSG00000268902.3	CSAG2	389903	5.058087053	1.42E-17	2.37E-16
1070	ENSG00000224897.8	POT1-AS1	401398	1.980599973	1.45E-17	2.42E-16
1071	ENSG00000139734.19	DIAPH3	81624	1.049043923	1.45E-17	2.43E-16
1072	ENSG00000259672.3			3.624083858	1.47E-17	2.45E-16
1073	ENSG00000185347.18	TEDC1	283643	1.263132343	1.48E-17	2.47E-16
1074	ENSG00000058335.16	RASGRF1	5923	2.020556064	1.51E-17	2.51E-16
1075	ENSG00000257702.3	LBX2-AS1	151534	1.242555125	1.58E-17	2.64E-16
1076	ENSG00000054967.13	RELT	84957	1.045923839	1.62E-17	2.69E-16
1077	ENSG00000286544.1			5.064108821	1.64E-17	2.72E-16
1078	ENSG00000151917.18	BEND6	221336	1.531721637	1.66E-17	2.76E-16
1079	ENSG00000101680.15	LAMA1	284217	2.562648551	1.69E-17	2.80E-16
1080	ENSG00000205436.7	EXOC3L4	91828	2.605570949	1.69E-17	2.81E-16
1081	ENSG00000160932.11	LY6E	4061	1.430767316	1.72E-17	2.84E-16
1082	ENSG00000286522.2	H3C2	8358	2.352528634	1.88E-17	3.10E-16
1083	ENSG00000287670.1			1.755541462	1.91E-17	3.15E-16
1084	ENSG00000224034.1	LINC02561		3.112898749	2.02E-17	3.32E-16
1085	ENSG00000220154.2			4.017886621	2.04E-17	3.36E-16
1086	ENSG00000144355.15	DLX1	1745	1.956266685	2.07E-17	3.39E-16
1087	ENSG00000185745.10	IFIT1	3434	2.128038675	2.08E-17	3.40E-16
1088	ENSG00000283959.1			1.669481726	2.16E-17	3.54E-16
1089	ENSG00000132530.17	XAF1	54739	1.723039036	2.19E-17	3.58E-16
1090	ENSG00000166349.10	RAG1	5896	1.833325464	2.22E-17	3.62E-16
1091	ENSG00000236200.6	KDM4A-AS1		1.481613074	2.24E-17	3.65E-16
1092	ENSG00000131203.13	IDO1	3620	2.745915831	2.27E-17	3.71E-16
1093	ENSG00000105974.13	CAV1	857	1.748661606	2.31E-17	3.76E-16
1094	ENSG00000180611.7	MB21D2	151963	1.077557831	2.32E-17	3.78E-16
1095	ENSG00000253394.6	LINC00534		3.17974524	2.38E-17	3.87E-16
1096	ENSG00000135248.15	GARIN1B	84691	3.872931	2.38E-17	3.88E-16
1097	ENSG00000224750.6			3.839943231	2.44E-17	3.97E-16
1098	ENSG00000229720.1		101929460	3.821794186	2.45E-17	3.98E-16
1099	ENSG00000104537.17	ANXA13	312	2.69974256	2.47E-17	4.02E-16
1100	ENSG00000280002.1			5.065374699	2.53E-17	4.10E-16
1101	ENSG00000105278.12	ZFR2	23217	4.179338007	2.60E-17	4.22E-16
1102	ENSG00000256663.1			1.418165425	2.67E-17	4.33E-16
1103	ENSG00000286614.1			3.55059359	2.98E-17	4.82E-16
1104	ENSG00000196876.18	SCN8A	6334	1.846424592	3.02E-17	4.88E-16
1105	ENSG00000157168.20	NRG1	3084	1.983667712	3.03E-17	4.90E-16
1106	ENSG00000119630.14	PGF	5228	1.749999808	3.13E-17	5.05E-16
1107	ENSG00000100479.13	POLE2	5427	1.012794091	3.15E-17	5.09E-16
1108	ENSG00000286015.1			5.157285017	3.25E-17	5.24E-16
1109	ENSG00000143147.14	GPR161	23432	1.278103829	3.35E-17	5.41E-16
1110	ENSG00000250548.7	LINC01303	101927780	1.907275551	3.47E-17	5.57E-16
1111	ENSG00000230750.1	SDAD1P2		4.268108691	3.50E-17	5.62E-16
1112	ENSG00000231607.12	DLEU2	8847	1.083309015	3.51E-17	5.64E-16
1113	ENSG00000267694.1		124904256	3.860886906	3.52E-17	5.65E-16
1114	ENSG00000197472.15	ZNF695	57116	2.418377525	3.80E-17	6.09E-16
1115	ENSG00000254560.6	BBOX1-AS1	103695435	1.608272404	3.83E-17	6.13E-16
1116	ENSG00000169994.18	MYO7B	4648	2.688499245	3.90E-17	6.24E-16
1117	ENSG00000092068.20	SLC7A8	23428	1.668542713	3.92E-17	6.27E-16
1118	ENSG00000082684.16	SEMA5B	54437	1.873528036	4.14E-17	6.62E-16
1119	ENSG00000268964.3	ERVV-2	100271846	5.149246084	4.34E-17	6.93E-16
1120	ENSG00000185742.7	C11orf87	399947	3.908327979	4.37E-17	6.97E-16
1121	ENSG00000249815.1	LINC02945	123464519	5.378744892	4.45E-17	7.09E-16
1122	ENSG00000235162.9	C12orf75	387882	1.424072393	4.53E-17	7.21E-16
1123	ENSG00000173894.11	CBX2	84733	1.434858308	4.55E-17	7.23E-16
1124	ENSG00000171121.16	KCNMB3	27094	1.675793801	4.59E-17	7.30E-16
1125	ENSG00000177674.16	AGTRAP	57085	1.102915019	4.86E-17	7.71E-16
1126	ENSG00000100162.15	CENPM	79019	1.279139345	4.96E-17	7.87E-16
1127	ENSG00000260589.1	STAM-DT	102723166	1.760056091	5.32E-17	8.42E-16
1128	ENSG00000164438.6	TLX3	30012	6.489684548	5.46E-17	8.63E-16
1129	ENSG00000259178.1			2.155442835	5.49E-17	8.68E-16
1130	ENSG00000217930.8	PAM16	51025	1.060139342	5.72E-17	9.02E-16
1131	ENSG00000255860.3			4.251614764	5.79E-17	9.11E-16
1132	ENSG00000283599.2		101059915	3.929097612	5.90E-17	9.29E-16
1133	ENSG00000132026.14	RTBDN	83546	3.196547783	5.94E-17	9.33E-16
1134	ENSG00000277342.1			1.939747251	6.12E-17	9.61E-16
1135	ENSG00000179598.6	PLD6	201164	1.214977197	6.19E-17	9.71E-16
1136	ENSG00000283737.1			3.967634297	6.28E-17	9.84E-16
1137	ENSG00000222022.1			3.393093314	6.34E-17	9.92E-16
1138	ENSG00000261143.1	ADAMTS7P3		2.203155204	6.43E-17	1.01E-15
1139	ENSG00000275180.1			1.988168727	6.51E-17	1.02E-15
1140	ENSG00000078081.8	LAMP3	27074	1.568132287	6.56E-17	1.03E-15
1141	ENSG00000138675.16	FGF5	2250	3.21059915	6.64E-17	1.04E-15
1142	ENSG00000149609.6	C20orf144	128864	1.537724681	6.95E-17	1.08E-15
1143	ENSG00000126790.12	L3HYPDH	112849	1.003427482	7.16E-17	1.11E-15
1144	ENSG00000204044.6	SLC12A5-AS1	109729184	3.418841448	7.42E-17	1.15E-15
1145	ENSG00000203799.13	CCDC162P		2.176779854	7.46E-17	1.16E-15
1146	ENSG00000119969.15	HELLS	3070	1.095186961	7.55E-17	1.17E-15
1147	ENSG00000174450.12	GOLGA6L2	283685	2.648597971	7.70E-17	1.20E-15
1148	ENSG00000171084.15	FAM86JP		1.076730853	7.83E-17	1.21E-15
1149	ENSG00000130475.14	FCHO1	23149	1.668175576	8.08E-17	1.25E-15
1150	ENSG00000232043.1			2.023841371	8.19E-17	1.27E-15
1151	ENSG00000267151.5	MIR2117HG	106660605	2.54018333	8.43E-17	1.30E-15
1152	ENSG00000206262.9	FOXL2NB	401089	2.867590061	8.61E-17	1.33E-15
1153	ENSG00000237870.6		102724434	3.251834639	8.63E-17	1.33E-15
1154	ENSG00000141756.19	FKBP10	60681	1.779998797	8.76E-17	1.35E-15
1155	ENSG00000227906.8	SNAP25-AS1		3.155528607	8.86E-17	1.37E-15
1156	ENSG00000107819.13	SFXN3	81855	1.052153125	8.88E-17	1.37E-15
1157	ENSG00000257924.1	LINC02416		2.200246432	8.92E-17	1.37E-15
1158	ENSG00000274460.1			2.00017544	8.95E-17	1.38E-15
1159	ENSG00000225205.5			1.159205544	9.08E-17	1.40E-15
1160	ENSG00000284946.1			1.579693989	9.11E-17	1.40E-15
1161	ENSG00000250081.1			2.8136915	9.18E-17	1.41E-15
1162	ENSG00000181097.5	LINC02878		1.531850939	9.37E-17	1.44E-15
1163	ENSG00000277156.1	TOMM40P1		2.835476042	9.39E-17	1.44E-15
1164	ENSG00000248740.6	LINC02428		3.037391661	9.73E-17	1.49E-15
1165	ENSG00000223722.3			2.727669599	9.80E-17	1.50E-15
1166	ENSG00000257253.2			2.450584674	1.04E-16	1.59E-15
1167	ENSG00000167895.15	TMC8	147138	1.40291935	1.04E-16	1.59E-15
1168	ENSG00000158373.8	H2BC5	3017	1.401112426	1.05E-16	1.60E-15
1169	ENSG00000250829.2			4.228274451	1.06E-16	1.62E-15
1170	ENSG00000259107.2	LINC00911		3.626453735	1.15E-16	1.76E-15
1171	ENSG00000167771.6	RCOR2	283248	1.953903672	1.17E-16	1.78E-15
1172	ENSG00000139329.5	LUM	4060	1.848486123	1.18E-16	1.79E-15
1173	ENSG00000169495.5	HTRA4	203100	2.409552535	1.18E-16	1.79E-15
1174	ENSG00000230061.2	TRPM2-AS		2.687371583	1.18E-16	1.80E-15
1175	ENSG00000166803.14	PCLAF	9768	1.235794931	1.19E-16	1.80E-15
1176	ENSG00000174837.15	ADGRE1	2015	2.377172971	1.22E-16	1.85E-15
1177	ENSG00000187689.10	AMTN	401138	3.463685735	1.24E-16	1.89E-15
1178	ENSG00000196368.5	NUDT11	55190	2.016289285	1.25E-16	1.89E-15
1179	ENSG00000231431.7	FAR2P4		5.638290549	1.33E-16	2.01E-15
1180	ENSG00000286555.1			1.761352394	1.33E-16	2.01E-15
1181	ENSG00000160233.8	LRRC3	81543	1.292750136	1.34E-16	2.02E-15
1182	ENSG00000140323.6	DISP2	85455	2.122314697	1.36E-16	2.05E-15
1183	ENSG00000101842.14	VSIG1	340547	1.453068044	1.38E-16	2.08E-15
1184	ENSG00000227154.4	MKRN6P		2.971423844	1.39E-16	2.09E-15
1185	ENSG00000242147.3	LASTR	102723629	2.06700019	1.39E-16	2.09E-15
1186	ENSG00000169902.15	TPST1	8460	1.125460958	1.50E-16	2.25E-15
1187	ENSG00000080511.5	RDH8	50700	3.322735804	1.54E-16	2.31E-15
1188	ENSG00000115318.12	LOXL3	84695	1.204026457	1.56E-16	2.34E-15
1189	ENSG00000176225.15	RTTN	25914	1.18467389	1.65E-16	2.48E-15
1190	ENSG00000184351.8	KRTAP19-1	337882	5.37406039	1.81E-16	2.70E-15
1191	ENSG00000182667.14	NTM	50863	1.684855067	1.82E-16	2.71E-15
1192	ENSG00000147896.3	IFNK	56832	4.549000377	1.82E-16	2.72E-15
1193	ENSG00000256955.2			3.261543149	1.83E-16	2.73E-15
1194	ENSG00000101104.13	PABPC1L	80336	1.555858469	1.88E-16	2.79E-15
1195	ENSG00000255345.1	TENM4-AS1	139281643	2.924917019	1.90E-16	2.82E-15
1196	ENSG00000240065.8	PSMB9	5698	1.53300009	1.90E-16	2.82E-15
1197	ENSG00000184106.8	TREML3P		3.0030858	1.97E-16	2.92E-15
1198	ENSG00000281327.2	LINC01338		2.213338363	2.07E-16	3.06E-15
1199	ENSG00000234352.9		349160	3.808194102	2.09E-16	3.09E-15
1200	ENSG00000178752.16	ERFE	151176	1.735001648	2.11E-16	3.12E-15
1201	ENSG00000277128.2			3.729977304	2.16E-16	3.19E-15
1202	ENSG00000222047.9	C10orf55	414236	1.399962209	2.16E-16	3.19E-15
1203	ENSG00000143184.5	XCL1	6375	1.781892655	2.21E-16	3.25E-15
1204	ENSG00000223725.6	MYOSLID-AS1		1.929518182	2.25E-16	3.31E-15
1205	ENSG00000229953.1	BCAN-AS2	126568844	2.3594286	2.27E-16	3.34E-15
1206	ENSG00000269993.1			4.914511033	2.30E-16	3.37E-15
1207	ENSG00000242622.2	GSK3B-DT	107986119	1.126893309	2.30E-16	3.38E-15
1208	ENSG00000130675.15	MNX1	3110	2.660533361	2.35E-16	3.45E-15
1209	ENSG00000125872.8	LRRN4	164312	2.665655398	2.39E-16	3.51E-15
1210	ENSG00000284052.1			1.290460111	2.51E-16	3.67E-15
1211	ENSG00000130055.14	GDPD2	54857	2.737765202	2.53E-16	3.69E-15
1212	ENSG00000147650.11	LRP12	29967	1.269888688	2.53E-16	3.69E-15
1213	ENSG00000166923.12	GREM1	26585	1.958180481	2.72E-16	3.96E-15
1214	ENSG00000262001.1	DLGAP1-AS2		1.70311815	2.73E-16	3.98E-15
1215	ENSG00000287346.1			1.296737419	2.74E-16	3.99E-15
1216	ENSG00000239392.2			2.786879029	2.83E-16	4.12E-15
1217	ENSG00000118508.5	RAB32	10981	1.126123863	2.94E-16	4.27E-15
1218	ENSG00000146857.4	STRA8	346673	3.918933464	3.02E-16	4.38E-15
1219	ENSG00000169676.6	DRD5	1816	3.064693278	3.03E-16	4.40E-15
1220	ENSG00000264464.1	LINC02837	101928144	3.880156272	3.03E-16	4.40E-15
1221	ENSG00000246095.2	LINC01096		2.406984	3.07E-16	4.45E-15
1222	ENSG00000251381.8	LINC00958	100506305	1.595932979	3.09E-16	4.47E-15
1223	ENSG00000258732.1			3.033096022	3.24E-16	4.68E-15
1224	ENSG00000196337.12	CGB7	94027	1.776968994	3.32E-16	4.79E-15
1225	ENSG00000114124.2	GRK7	131890	2.576214395	3.34E-16	4.82E-15
1226	ENSG00000166278.15	C2	717	1.338002088	3.34E-16	4.82E-15
1227	ENSG00000213949.10	ITGA1	3672	1.22337834	3.35E-16	4.83E-15
1228	ENSG00000105717.14	PBX4	80714	1.44629757	3.38E-16	4.87E-15
1229	ENSG00000172061.9	LRRC15	131578	2.471593715	3.39E-16	4.88E-15
1230	ENSG00000257298.1			1.677391268	3.49E-16	5.02E-15
1231	ENSG00000122733.12	PHF24	23349	2.154419274	3.49E-16	5.03E-15
1232	ENSG00000107201.10	RIGI	23586	1.523989058	3.62E-16	5.20E-15
1233	ENSG00000213730.3	POLD2P1		3.136851795	3.72E-16	5.35E-15
1234	ENSG00000169245.6	CXCL10	3627	2.714121811	3.81E-16	5.47E-15
1235	ENSG00000135862.6	LAMC1	3915	1.007335918	4.05E-16	5.80E-15
1236	ENSG00000205268.11	PDE7A	5150	1.035319564	4.10E-16	5.88E-15
1237	ENSG00000234828.9	IQCM	285423	3.865178	4.17E-16	5.96E-15
1238	ENSG00000178773.15	CPNE7	27132	1.930848322	4.18E-16	5.96E-15
1239	ENSG00000106689.11	LHX2	9355	2.53157269	4.23E-16	6.04E-15
1240	ENSG00000116701.15	NCF2	4688	1.162964233	4.23E-16	6.04E-15
1241	ENSG00000246889.2	CTTN-DT		1.906590321	4.26E-16	6.07E-15
1242	ENSG00000164294.14	GPX8	493869	1.200096071	4.46E-16	6.34E-15
1243	ENSG00000214336.5	FOXI3	344167	3.88913906	4.49E-16	6.38E-15
1244	ENSG00000204941.14	PSG5	5673	3.279538454	4.75E-16	6.73E-15
1245	ENSG00000182366.10	FAM87A	157693	1.801459653	4.84E-16	6.85E-15
1246	ENSG00000228189.5	LINC02843		4.923158015	5.09E-16	7.19E-15
1247	ENSG00000204791.10	SMPD5		1.938344838	5.24E-16	7.38E-15
1248	ENSG00000091879.14	ANGPT2	285	1.265842209	5.29E-16	7.46E-15
1249	ENSG00000164796.18	CSMD3	114788	3.503456298	5.35E-16	7.53E-15
1250	ENSG00000197646.8	PDCD1LG2	80380	1.714741175	5.56E-16	7.83E-15
1251	ENSG00000255262.3	ELOBP2		2.741795105	5.57E-16	7.84E-15
1252	ENSG00000258910.3	LINC01956		3.537197786	5.62E-16	7.91E-15
1253	ENSG00000250390.2			3.138687245	5.67E-16	7.98E-15
1254	ENSG00000171848.16	RRM2	6241	1.011613621	5.68E-16	7.98E-15
1255	ENSG00000025708.14	TYMP	1890	1.43395288	5.72E-16	8.04E-15
1256	ENSG00000100342.21	APOL1	8542	1.686755711	5.80E-16	8.14E-15
1257	ENSG00000134326.11	CMPK2	129607	1.737644165	5.96E-16	8.36E-15
1258	ENSG00000287329.1		105379444	4.18053736	6.02E-16	8.44E-15
1259	ENSG00000147804.10	SLC39A4	55630	1.251285076	6.03E-16	8.45E-15
1260	ENSG00000117148.8	ACTL8	81569	3.463098518	6.03E-16	8.45E-15
1261	ENSG00000179761.12	PIPOX	51268	2.006271917	6.25E-16	8.76E-15
1262	ENSG00000198901.14	PRC1	9055	1.267567322	6.30E-16	8.81E-15
1263	ENSG00000256151.1	ADGRD1-AS1		5.064845784	6.72E-16	9.38E-15
1264	ENSG00000204540.11	PSORS1C1	170679	1.8132693	6.73E-16	9.39E-15
1265	ENSG00000283128.2			2.497820034	6.78E-16	9.45E-15
1266	ENSG00000137628.17	DDX60	55601	1.609089814	7.08E-16	9.87E-15
1267	ENSG00000268941.2	LINC01711	79160	2.514446289	7.12E-16	9.91E-15
1268	ENSG00000173486.14	FKBP2	2286	1.772950331	7.40E-16	1.03E-14
1269	ENSG00000174325.6	DIRC1		2.896795403	7.64E-16	1.06E-14
1270	ENSG00000187772.8	LIN28B	389421	6.362689346	7.76E-16	1.08E-14
1271	ENSG00000132517.15	SLC52A1	55065	1.654372443	7.94E-16	1.10E-14
1272	ENSG00000253931.1			3.142545865	7.95E-16	1.10E-14
1273	ENSG00000083782.8	EPYC	1833	3.48639317	8.36E-16	1.15E-14
1274	ENSG00000256915.2			3.522674068	8.75E-16	1.21E-14
1275	ENSG00000165490.13	DDIAS	220042	1.039495993	8.78E-16	1.21E-14
1276	ENSG00000230863.3			2.602881399	8.86E-16	1.22E-14
1277	ENSG00000213344.2	PCNPP3		3.29793768	9.16E-16	1.26E-14
1278	ENSG00000231066.3	NPM1P9		1.570435954	9.20E-16	1.27E-14
1279	ENSG00000256001.2			2.166338814	9.24E-16	1.27E-14
1280	ENSG00000049768.16	FOXP3	50943	1.504319846	9.29E-16	1.28E-14
1281	ENSG00000206820.1	RNU1-138P		3.305381261	9.43E-16	1.30E-14
1282	ENSG00000011590.14	ZBTB32	27033	1.766871732	9.82E-16	1.35E-14
1283	ENSG00000180066.10	LINC02870		1.841488536	1.00E-15	1.37E-14
1284	ENSG00000129810.15	SGO1	151648	1.014289952	1.01E-15	1.38E-14
1285	ENSG00000230002.3	ALMS1-IT1		1.376176801	1.04E-15	1.42E-14
1286	ENSG00000168298.7	H1-4	3008	1.742704586	1.04E-15	1.43E-14
1287	ENSG00000136235.17	GPNMB	10457	1.549652797	1.06E-15	1.45E-14
1288	ENSG00000198108.4	CHSY3	337876	1.489605554	1.10E-15	1.50E-14
1289	ENSG00000124140.14	SLC12A5	57468	2.02062424	1.12E-15	1.53E-14
1290	ENSG00000286791.1			2.365055552	1.13E-15	1.54E-14
1291	ENSG00000261742.6	LINC00922	283867	3.339242653	1.14E-15	1.55E-14
1292	ENSG00000249413.2			3.774349731	1.15E-15	1.57E-14
1293	ENSG00000220472.1	RPS3P4		1.326537662	1.17E-15	1.59E-14
1294	ENSG00000232053.7		105375523	2.225801022	1.23E-15	1.67E-14
1295	ENSG00000268621.6	IGFL2-AS1	645553	2.657290698	1.25E-15	1.70E-14
1296	ENSG00000228933.8			3.90074261	1.27E-15	1.72E-14
1297	ENSG00000104889.7	RNASEH2A	10535	2.046362516	1.30E-15	1.77E-14
1298	ENSG00000188624.3	IGFL3	388555	3.499116569	1.43E-15	1.93E-14
1299	ENSG00000258763.5		124902940	3.988008648	1.44E-15	1.94E-14
1300	ENSG00000143512.13	HHIPL2	79802	2.801347465	1.44E-15	1.95E-14
1301	ENSG00000234695.1	TFPI2-DT		2.781239571	1.46E-15	1.96E-14
1302	ENSG00000236548.2	RNF217-AS1		1.690586904	1.46E-15	1.97E-14
1303	ENSG00000258384.1	RCCD1-AS1		1.664753967	1.47E-15	1.98E-14
1304	ENSG00000188501.11	LCTL	197021	1.711673378	1.48E-15	1.99E-14
1305	ENSG00000133101.10	CCNA1	8900	2.816321254	1.52E-15	2.04E-14
1306	ENSG00000165181.16	SHOC1	158401	1.497916132	1.53E-15	2.06E-14
1307	ENSG00000062038.14	CDH3	1001	1.329779389	1.56E-15	2.09E-14
1308	ENSG00000188064.10	WNT7B	7477	1.23928725	1.57E-15	2.10E-14
1309	ENSG00000258168.6	PTPRB-AS1		2.638296444	1.65E-15	2.22E-14
1310	ENSG00000131620.17	ANO1	55107	2.069481087	1.69E-15	2.27E-14
1311	ENSG00000124635.9	H2BC11	8970	1.609475635	1.70E-15	2.27E-14
1312	ENSG00000143469.20	SYT14	255928	2.698898899	1.72E-15	2.30E-14
1313	ENSG00000285600.1			3.290483132	1.76E-15	2.36E-14
1314	ENSG00000115415.20	STAT1	6772	1.327484883	1.82E-15	2.43E-14
1315	ENSG00000247402.2			2.465159001	1.82E-15	2.44E-14
1316	ENSG00000271830.1			2.989936465	1.84E-15	2.46E-14
1317	ENSG00000125864.14	BFSP1	631	1.332870418	1.86E-15	2.48E-14
1318	ENSG00000258839.4	MC1R	4157	1.127207346	1.88E-15	2.50E-14
1319	ENSG00000111981.5	ULBP1	80329	2.23499104	1.91E-15	2.54E-14
1320	ENSG00000197153.5	H3C12	8356	2.053816274	1.94E-15	2.58E-14
1321	ENSG00000280356.1			3.437800368	1.99E-15	2.64E-14
1322	ENSG00000140022.13	STON2	85439	1.175172019	1.99E-15	2.64E-14
1323	ENSG00000233885.7	YEATS2-AS1		1.422849491	2.00E-15	2.65E-14
1324	ENSG00000180061.10	TMEM150B	284417	1.616140006	2.03E-15	2.69E-14
1325	ENSG00000283341.2			1.644698145	2.03E-15	2.70E-14
1326	ENSG00000213729.3	RPS16P2		3.280338899	2.12E-15	2.80E-14
1327	ENSG00000233542.2			2.362647411	2.16E-15	2.85E-14
1328	ENSG00000279331.1	RBM12B-AS1	55472	1.334235848	2.17E-15	2.87E-14
1329	ENSG00000226145.7	KRT16P6		2.873083148	2.35E-15	3.10E-14
1330	ENSG00000251179.1	TMEM92-AS1	103752589	1.924918005	2.39E-15	3.14E-14
1331	ENSG00000204610.13	TRIM15	89870	2.93374986	2.41E-15	3.18E-14
1332	ENSG00000187730.9	GABRD	2563	1.859951487	2.56E-15	3.36E-14
1333	ENSG00000256540.1	IQSEC3-AS1		1.765318152	2.78E-15	3.64E-14
1334	ENSG00000100097.12	LGALS1	3956	1.49257494	2.80E-15	3.67E-14
1335	ENSG00000173546.7	CSPG4	1464	1.904920679	2.81E-15	3.68E-14
1336	ENSG00000243742.5	RPLP0P2		1.710747962	2.81E-15	3.68E-14
1337	ENSG00000265182.1	SRP72P1		3.336507253	2.89E-15	3.79E-14
1338	ENSG00000130589.16	HELZ2	85441	1.180927369	2.90E-15	3.79E-14
1339	ENSG00000287229.1			3.505652271	2.97E-15	3.87E-14
1340	ENSG00000147434.9	CHRNA6	8973	2.313782609	3.19E-15	4.16E-14
1341	ENSG00000160117.16	ANKLE1	126549	1.988973743	3.21E-15	4.17E-14
1342	ENSG00000269821.1	KCNQ1OT1	10984	1.269513376	3.31E-15	4.31E-14
1343	ENSG00000239213.6	NCK1-DT	101927597	1.045868642	3.59E-15	4.65E-14
1344	ENSG00000284649.1			2.869470331	3.90E-15	5.04E-14
1345	ENSG00000276903.2	H2AC16	8332	2.111758055	3.90E-15	5.04E-14
1346	ENSG00000007372.24	PAX6	5080	2.738138414	4.08E-15	5.27E-14
1347	ENSG00000104899.8	AMH	268	2.512214303	4.13E-15	5.33E-14
1348	ENSG00000072274.13	TFRC	7037	1.304272779	4.14E-15	5.35E-14
1349	ENSG00000234800.2	PCMTD1P3		2.914354025	4.33E-15	5.58E-14
1350	ENSG00000204642.14	HLA-F	3134	1.382757873	4.45E-15	5.73E-14
1351	ENSG00000196092.14	PAX5	5079	2.125768354	4.55E-15	5.85E-14
1352	ENSG00000273002.1	ARHGEF2-AS2	107985209	1.57357702	4.61E-15	5.92E-14
1353	ENSG00000271503.6	CCL5	6352	1.830206307	4.64E-15	5.95E-14
1354	ENSG00000172244.9	C5orf34	375444	1.122019272	4.79E-15	6.15E-14
1355	ENSG00000271996.1			3.327501598	4.82E-15	6.19E-14
1356	ENSG00000136122.18	BORA	79866	1.075138744	4.93E-15	6.33E-14
1357	ENSG00000104951.16	IL4I1	259307	1.633846218	4.94E-15	6.33E-14
1358	ENSG00000180739.14	S1PR5	53637	1.147800116	5.11E-15	6.54E-14
1359	ENSG00000233384.3	CNIH3-AS2	102723817	2.855972589	5.18E-15	6.62E-14
1360	ENSG00000168454.12	TXNDC2	84203	1.718571425	5.23E-15	6.69E-14
1361	ENSG00000217236.2	SP9	100131390	2.922848479	5.34E-15	6.81E-14
1362	ENSG00000142619.4	PADI3	51702	2.550012233	5.43E-15	6.93E-14
1363	ENSG00000165935.9	SMCO2	341346	1.339386235	5.51E-15	7.02E-14
1364	ENSG00000106410.15	NOBOX	135935	7.591056892	5.52E-15	7.03E-14
1365	ENSG00000183853.18	KIRREL1	55243	1.198865273	5.57E-15	7.09E-14
1366	ENSG00000121905.10	HPCA	3208	1.704224924	5.64E-15	7.19E-14
1367	ENSG00000206337.12	HCP5	10866	1.176117359	5.69E-15	7.25E-14
1368	ENSG00000249992.2	TMEM158	25907	1.568297054	5.82E-15	7.40E-14
1369	ENSG00000196924.19	FLNA	2316	1.026821875	5.87E-15	7.46E-14
1370	ENSG00000176788.9	BASP1	10409	1.522836504	5.89E-15	7.49E-14
1371	ENSG00000225548.7	LINC01980	105377007	2.961213547	5.90E-15	7.49E-14
1372	ENSG00000102575.13	ACP5	54	1.169708833	6.10E-15	7.74E-14
1373	ENSG00000235531.11	MSC-AS1	100132891	1.555323231	6.25E-15	7.93E-14
1374	ENSG00000225907.1	RPS27P16		2.350730418	6.26E-15	7.94E-14
1375	ENSG00000200879.1	SNORD14E	85391	2.400294532	6.36E-15	8.06E-14
1376	ENSG00000280383.1			1.386400712	6.42E-15	8.13E-14
1377	ENSG00000286540.1			3.069808671	6.55E-15	8.28E-14
1378	ENSG00000124614.16	RPS10	6204	1.437534894	6.68E-15	8.44E-14
1379	ENSG00000102794.10	ACOD1	730249	3.32336432	6.81E-15	8.61E-14
1380	ENSG00000261039.3			2.184299763	6.87E-15	8.68E-14
1381	ENSG00000231806.3	PCAT7	101928099	2.058768254	6.93E-15	8.74E-14
1382	ENSG00000236540.7			1.070407111	6.94E-15	8.76E-14
1383	ENSG00000106268.15	NUDT1	4521	1.126790692	7.03E-15	8.86E-14
1384	ENSG00000266088.6		105371773	1.636880926	7.43E-15	9.35E-14
1385	ENSG00000211896.7	IGHG1		2.695283475	7.62E-15	9.57E-14
1386	ENSG00000213786.3	NHP2P2		3.140796055	8.03E-15	1.01E-13
1387	ENSG00000286828.1			1.899228992	8.03E-15	1.01E-13
1388	ENSG00000135074.16	ADAM19	8728	1.501889269	8.17E-15	1.02E-13
1389	ENSG00000139547.7	RDH16	8608	1.861244817	8.34E-15	1.05E-13
1390	ENSG00000187951.11		100288637	1.084979893	8.37E-15	1.05E-13
1391	ENSG00000142185.16	TRPM2	7226	1.487802877	8.49E-15	1.06E-13
1392	ENSG00000277075.2	H2AC8	3012	1.690900229	8.79E-15	1.10E-13
1393	ENSG00000230732.4			1.470551331	8.89E-15	1.11E-13
1394	ENSG00000253553.7			2.948550194	9.10E-15	1.14E-13
1395	ENSG00000272468.1			1.819835017	9.26E-15	1.16E-13
1396	ENSG00000156466.10	GDF6	392255	2.474022513	9.50E-15	1.18E-13
1397	ENSG00000205562.3	FLRT2-AS1	100506731	2.159161756	1.01E-14	1.26E-13
1398	ENSG00000169129.15	AFAP1L2	84632	1.142055566	1.04E-14	1.29E-13
1399	ENSG00000101188.5	NTSR1	4923	3.160835435	1.05E-14	1.31E-13
1400	ENSG00000160973.8	FOXH1	8928	3.479039688	1.07E-14	1.33E-13
1401	ENSG00000177294.7	FBXO39	162517	1.781859523	1.08E-14	1.34E-13
1402	ENSG00000227964.1	LINC01429	101927678	3.401580341	1.09E-14	1.35E-13
1403	ENSG00000284634.1		374443	1.689601418	1.11E-14	1.37E-13
1404	ENSG00000186790.6	FOXE3	2301	3.315200062	1.11E-14	1.37E-13
1405	ENSG00000233966.1	UBE2SP1		1.579000941	1.11E-14	1.38E-13
1406	ENSG00000230936.1			4.704678571	1.13E-14	1.40E-13
1407	ENSG00000267048.1			1.600325343	1.13E-14	1.40E-13
1408	ENSG00000258073.1			4.015068154	1.15E-14	1.42E-13
1409	ENSG00000182791.5	CCDC87	55231	1.308893786	1.16E-14	1.43E-13
1410	ENSG00000277945.1			3.505111364	1.18E-14	1.46E-13
1411	ENSG00000170044.8	ZPLD1	131368	2.453175191	1.20E-14	1.48E-13
1412	ENSG00000168685.15	IL7R	3575	1.524974672	1.27E-14	1.56E-13
1413	ENSG00000188816.3	HMX2	3167	4.748997103	1.30E-14	1.60E-13
1414	ENSG00000206878.1			3.280507457	1.32E-14	1.63E-13
1415	ENSG00000170054.16	SERPINA9	327657	3.082745753	1.33E-14	1.64E-13
1416	ENSG00000251692.8	PTX4	390667	4.032044176	1.33E-14	1.64E-13
1417	ENSG00000139973.16	SYT16	83851	2.391567878	1.34E-14	1.65E-13
1418	ENSG00000273297.2	LINC03060	105375517	1.953734681	1.38E-14	1.69E-13
1419	ENSG00000251273.4	LINC02228		4.292568185	1.48E-14	1.80E-13
1420	ENSG00000134028.14	ADAMDEC1	27299	2.130177067	1.48E-14	1.81E-13
1421	ENSG00000170049.9	KCNAB3	9196	1.458929828	1.51E-14	1.84E-13
1422	ENSG00000142102.16	PGGHG	80162	1.408645703	1.52E-14	1.85E-13
1423	ENSG00000234745.12	HLA-B	3106	1.214728246	1.54E-14	1.87E-13
1424	ENSG00000089356.18	FXYD3	5349	1.407651496	1.60E-14	1.94E-13
1425	ENSG00000118156.12	ZNF541	84215	3.091866628	1.65E-14	2.00E-13
1426	ENSG00000069188.17	SDK2	54549	1.715399352	1.68E-14	2.04E-13
1427	ENSG00000281406.2	BLACAT1	101669762	1.359673225	1.68E-14	2.04E-13
1428	ENSG00000281706.3	LINC01012	100507173	1.521643116	1.70E-14	2.06E-13
1429	ENSG00000221829.10	FANCG	2189	1.524086067	1.70E-14	2.06E-13
1430	ENSG00000092470.12	WDR76	79968	1.038383582	1.73E-14	2.09E-13
1431	ENSG00000231346.5	LINC01160		2.548749627	1.73E-14	2.10E-13
1432	ENSG00000262703.1			2.236497578	1.78E-14	2.15E-13
1433	ENSG00000103546.18	SLC6A2	6530	2.33948567	1.81E-14	2.18E-13
1434	ENSG00000269720.2	CCDC194	110806280	2.790650859	1.86E-14	2.24E-13
1435	ENSG00000150630.4	VEGFC	7424	1.840309131	1.86E-14	2.25E-13
1436	ENSG00000165685.8	TMEM52B	120939	2.354145191	1.89E-14	2.28E-13
1437	ENSG00000167414.4	GNG8	94235	2.426659107	1.95E-14	2.35E-13
1438	ENSG00000171867.17	PRNP	5621	1.062499835	1.98E-14	2.38E-13
1439	ENSG00000123989.14	CHPF	79586	1.071787014	2.14E-14	2.57E-13
1440	ENSG00000184524.6	CEND1	51286	1.654426778	2.15E-14	2.58E-13
1441	ENSG00000243449.6	NICOL1	401115	1.633874816	2.16E-14	2.59E-13
1442	ENSG00000140853.16	NLRC5	84166	1.094966796	2.19E-14	2.63E-13
1443	ENSG00000112238.12	PRDM13	59336	3.297900685	2.23E-14	2.67E-13
1444	ENSG00000149716.13	LTO1	220064	1.511598669	2.24E-14	2.68E-13
1445	ENSG00000266289.1	LINC02933		2.006816799	2.24E-14	2.68E-13
1446	ENSG00000179431.7	FJX1	24147	1.196856427	2.39E-14	2.86E-13
1447	ENSG00000154978.13	VOPP1	81552	1.212730773	2.40E-14	2.87E-13
1448	ENSG00000128645.15	HOXD1	3231	2.213673816	2.40E-14	2.87E-13
1449	ENSG00000260853.2	NFATC2IP-AS1	123706545	1.289936893	2.41E-14	2.88E-13
1450	ENSG00000228519.3	RANBP1P1		1.203374954	2.43E-14	2.90E-13
1451	ENSG00000136492.9	BRIP1	83990	1.021090045	2.50E-14	2.99E-13
1452	ENSG00000162639.16	HENMT1	113802	1.179652581	2.54E-14	3.03E-13
1453	ENSG00000184786.6	DYNLT2	6991	1.142035737	2.55E-14	3.04E-13
1454	ENSG00000227533.6	SLC2A1-DT	440584	1.445734502	2.61E-14	3.10E-13
1455	ENSG00000187258.14	NPSR1	387129	2.587712946	2.62E-14	3.12E-13
1456	ENSG00000282950.1			3.005775447	2.64E-14	3.14E-13
1457	ENSG00000158292.7	GPR153	387509	1.231459157	2.67E-14	3.17E-13
1458	ENSG00000263961.8	RHEX	440712	2.37757283	2.68E-14	3.19E-13
1459	ENSG00000254338.1	MAFA-AS1		2.941879685	2.76E-14	3.27E-13
1460	ENSG00000164266.11	SPINK1	6690	2.832271709	2.78E-14	3.29E-13
1461	ENSG00000236481.1	LINC02195	105371152	1.898496287	2.84E-14	3.35E-13
1462	ENSG00000224067.2			2.009952273	3.03E-14	3.57E-13
1463	ENSG00000105472.13	CLEC11A	6320	1.522612285	3.10E-14	3.65E-13
1464	ENSG00000151006.7	PRSS53	339105	1.66061395	3.10E-14	3.65E-13
1465	ENSG00000255856.2			1.707580759	3.25E-14	3.82E-13
1466	ENSG00000180176.15	TH	7054	3.037051534	3.33E-14	3.92E-13
1467	ENSG00000125675.18	GRIA3	2892	2.119777006	3.43E-14	4.02E-13
1468	ENSG00000160883.11	HK3	3101	1.768110216	3.47E-14	4.07E-13
1469	ENSG00000144488.15	ESPNL	339768	2.320321269	3.54E-14	4.15E-13
1470	ENSG00000274290.3	H2BC6	8344	1.371237355	3.55E-14	4.16E-13
1471	ENSG00000137868.19	STRA6	64220	1.951572848	3.60E-14	4.21E-13
1472	ENSG00000170214.5	ADRA1B	147	2.302344844	3.64E-14	4.26E-13
1473	ENSG00000258654.1			3.373923998	3.67E-14	4.29E-13
1474	ENSG00000205837.7	LINC00487	400941	2.869300454	3.71E-14	4.34E-13
1475	ENSG00000230316.7	FEZF1-AS1	154860	3.1486103	3.72E-14	4.35E-13
1476	ENSG00000231881.2			2.017788922	3.83E-14	4.48E-13
1477	ENSG00000143199.18	ADCY10	55811	2.336259648	3.91E-14	4.57E-13
1478	ENSG00000248265.2			2.314506276	3.99E-14	4.66E-13
1479	ENSG00000157833.13	GAREM2	150946	1.256194614	4.02E-14	4.69E-13
1480	ENSG00000134538.3	SLCO1B1	10599	3.83073666	4.03E-14	4.70E-13
1481	ENSG00000198400.12	NTRK1	4914	1.432166225	4.04E-14	4.71E-13
1482	ENSG00000152592.14	DMP1	1758	2.607257913	4.04E-14	4.71E-13
1483	ENSG00000271151.1			1.835702135	4.09E-14	4.76E-13
1484	ENSG00000110427.17	KIAA1549L	25758	1.81436592	4.10E-14	4.77E-13
1485	ENSG00000176890.16	TYMS	7298	1.101448301	4.11E-14	4.77E-13
1486	ENSG00000156587.16	UBE2L6	9246	1.1551451	4.16E-14	4.83E-13
1487	ENSG00000164611.13	PTTG1	9232	1.131083777	4.19E-14	4.87E-13
1488	ENSG00000183688.4	RFLNB	359845	1.136294346	4.23E-14	4.90E-13
1489	ENSG00000263711.6	LINC02864	400655	5.121147537	4.39E-14	5.09E-13
1490	ENSG00000116544.12	DLGAP3	58512	1.870199362	4.42E-14	5.11E-13
1491	ENSG00000140105.18	WARS1	7453	1.781113837	4.62E-14	5.35E-13
1492	ENSG00000014138.9	POLA2	23649	1.126854211	4.73E-14	5.47E-13
1493	ENSG00000101463.6	SYNDIG1	79953	2.245273421	4.79E-14	5.52E-13
1494	ENSG00000288555.1			3.958772108	4.83E-14	5.57E-13
1495	ENSG00000268403.2	ZNF143-AS1	644656	1.642134318	4.83E-14	5.57E-13
1496	ENSG00000213997.3	PGAM1P7		2.344258899	4.84E-14	5.58E-13
1497	ENSG00000183186.8	C2CD4C	126567	1.771363403	4.89E-14	5.63E-13
1498	ENSG00000231725.1	VN1R110P		2.819815446	4.91E-14	5.65E-13
1499	ENSG00000284138.1	ATP6V0CP4		2.525562467	5.02E-14	5.78E-13
1500	ENSG00000198598.6	MMP17	4326	1.375941301	5.09E-14	5.85E-13
1501	ENSG00000229334.1	TMEM44-AS2		1.232463783	5.12E-14	5.88E-13
1502	ENSG00000146666.5	LINC00525	84847	2.701252322	5.18E-14	5.95E-13
1503	ENSG00000248329.6	APELA	100506013	2.368663336	5.35E-14	6.14E-13
1504	ENSG00000136490.9	LIMD2	80774	1.204219494	5.46E-14	6.27E-13
1505	ENSG00000287080.2	H3C3	8352	1.912919116	5.49E-14	6.29E-13
1506	ENSG00000214243.3			1.373288339	5.69E-14	6.52E-13
1507	ENSG00000215267.8	AKR1C7P		2.468303703	5.71E-14	6.54E-13
1508	ENSG00000100167.20	SEPTIN3	55964	1.886590927	5.76E-14	6.58E-13
1509	ENSG00000221887.6	HMSD	284293	2.236800416	6.01E-14	6.87E-13
1510	ENSG00000250682.6	LINC00491	285708	2.118141889	6.10E-14	6.97E-13
1511	ENSG00000259692.6	LINC01418		2.19897447	6.13E-14	7.00E-13
1512	ENSG00000203747.12	FCGR3A	2214	1.627412797	6.17E-14	7.04E-13
1513	ENSG00000260167.1			2.118499209	6.25E-14	7.13E-13
1514	ENSG00000146197.9	SCUBE3	222663	2.62938115	6.25E-14	7.13E-13
1515	ENSG00000256802.2	LCIIAR	100130111	2.099570593	6.29E-14	7.17E-13
1516	ENSG00000174469.23	CNTNAP2	26047	2.49038822	6.36E-14	7.24E-13
1517	ENSG00000168394.11	TAP1	6890	1.25960703	6.55E-14	7.46E-13
1518	ENSG00000213185.7	FAM24B	196792	1.480728144	6.55E-14	7.46E-13
1519	ENSG00000260005.6	TEPSIN-AS1		1.319443013	6.79E-14	7.72E-13
1520	ENSG00000170166.6	HOXD4	3233	2.030200465	6.83E-14	7.77E-13
1521	ENSG00000287853.1			2.384766008	6.84E-14	7.77E-13
1522	ENSG00000113327.17	GABRG2	2566	4.751897216	7.06E-14	8.03E-13
1523	ENSG00000126467.11	TSKS	60385	2.559104268	7.21E-14	8.18E-13
1524	ENSG00000105976.16	MET	4233	1.089123547	7.33E-14	8.31E-13
1525	ENSG00000228393.4			1.225752522	7.33E-14	8.31E-13
1526	ENSG00000136574.18	GATA4	2626	4.316525744	7.39E-14	8.37E-13
1527	ENSG00000259594.6			3.041774812	7.44E-14	8.43E-13
1528	ENSG00000124256.15	ZBP1	81030	1.788690486	7.50E-14	8.49E-13
1529	ENSG00000239382.10	ALKBH6	84964	1.295088878	7.58E-14	8.57E-13
1530	ENSG00000115758.13	ODC1	4953	1.737607063	7.66E-14	8.66E-13
1531	ENSG00000252690.3			1.849387739	7.68E-14	8.68E-13
1532	ENSG00000123219.13	CENPK	64105	1.113264705	7.69E-14	8.68E-13
1533	ENSG00000177839.7	PCDHB9	56127	1.393161163	7.69E-14	8.68E-13
1534	ENSG00000212232.1	SNORD17	692086	1.344990809	7.88E-14	8.90E-13
1535	ENSG00000207296.1	RNU6-140P		1.514366375	7.99E-14	9.02E-13
1536	ENSG00000269706.1			4.203863868	8.26E-14	9.31E-13
1537	ENSG00000271781.1			1.570725896	8.26E-14	9.31E-13
1538	ENSG00000134986.14	NREP	9315	1.170727245	8.59E-14	9.66E-13
1539	ENSG00000267073.1			2.530655647	8.61E-14	9.68E-13
1540	ENSG00000225916.1			2.825792862	8.75E-14	9.83E-13
1541	ENSG00000233975.1	LINC02574		2.764882888	8.79E-14	9.87E-13
1542	ENSG00000263655.1			3.726131641	8.85E-14	9.94E-13
1543	ENSG00000254503.1			1.490692727	9.18E-14	1.03E-12
1544	ENSG00000229162.1	RUNX3-AS1		1.878251477	9.23E-14	1.03E-12
1545	ENSG00000075340.23	ADD2	119	2.538264317	9.28E-14	1.04E-12
1546	ENSG00000261559.1	FSCN1P1		1.745924037	9.30E-14	1.04E-12
1547	ENSG00000250337.7	PURPL	643401	3.409224039	9.30E-14	1.04E-12
1548	ENSG00000236449.2			3.101793565	9.50E-14	1.06E-12
1549	ENSG00000226887.8	ERVMER34-1	100288413	1.398850191	9.60E-14	1.07E-12
1550	ENSG00000136231.14	IGF2BP3	10643	2.046441783	9.92E-14	1.11E-12
1551	ENSG00000171320.15	ESCO2	157570	1.076844365	1.00E-13	1.12E-12
1552	ENSG00000236015.1	TMX1P1		1.610391971	1.01E-13	1.12E-12
1553	ENSG00000263201.1	DPEP2NB	100131303	3.675734813	1.02E-13	1.14E-12
1554	ENSG00000276966.2	H4C5	8367	1.638330956	1.03E-13	1.15E-12
1555	ENSG00000165568.18	AKR1E2	83592	1.572887096	1.05E-13	1.17E-12
1556	ENSG00000224194.1			3.592920018	1.07E-13	1.19E-12
1557	ENSG00000109089.7	CDR2L	30850	1.101610017	1.08E-13	1.20E-12
1558	ENSG00000134940.14	ACRV1	56	1.352458111	1.11E-13	1.24E-12
1559	ENSG00000225880.5	LINC00115		1.210312252	1.13E-13	1.26E-12
1560	ENSG00000128610.12	FEZF1	389549	3.51623971	1.14E-13	1.27E-12
1561	ENSG00000274925.1	ZKSCAN2-DT		1.384827829	1.16E-13	1.29E-12
1562	ENSG00000261236.8	BOP1	23246	1.019732398	1.19E-13	1.32E-12
1563	ENSG00000244578.1	LINC01391	103344930	3.391699381	1.21E-13	1.34E-12
1564	ENSG00000142173.16	COL6A2	1292	1.547374645	1.22E-13	1.35E-12
1565	ENSG00000185130.5	H2BC13	8340	1.557662305	1.23E-13	1.36E-12
1566	ENSG00000185585.20	OLFML2A	169611	1.300375209	1.25E-13	1.38E-12
1567	ENSG00000225313.5			1.183084773	1.25E-13	1.38E-12
1568	ENSG00000179111.9	HES7	84667	2.203806475	1.25E-13	1.38E-12
1569	ENSG00000272455.1	MRPL20-DT		1.037303967	1.32E-13	1.46E-12
1570	ENSG00000203814.6	H2BC18	440689	1.600975601	1.36E-13	1.51E-12
1571	ENSG00000258521.1			2.328691856	1.41E-13	1.55E-12
1572	ENSG00000266278.1	LINC01910	101060542	2.504924752	1.41E-13	1.56E-12
1573	ENSG00000266401.2	DLGAP1-AS6	105371967	3.533923935	1.43E-13	1.58E-12
1574	ENSG00000160867.15	FGFR4	2264	1.47645682	1.44E-13	1.58E-12
1575	ENSG00000166961.15	MS4A15	219995	3.147468595	1.45E-13	1.59E-12
1576	ENSG00000173077.16	DELEC1	50514	2.166920892	1.48E-13	1.62E-12
1577	ENSG00000235790.7	PEF1-AS1		1.576498327	1.51E-13	1.66E-12
1578	ENSG00000105467.9	SYNGR4	23546	2.386006173	1.51E-13	1.66E-12
1579	ENSG00000137090.12	DMRT1	1761	3.427399561	1.52E-13	1.66E-12
1580	ENSG00000237636.3	ANKRD26P3		3.70302582	1.53E-13	1.68E-12
1581	ENSG00000270195.2			1.838469822	1.54E-13	1.68E-12
1582	ENSG00000179934.7	CCR8	1237	1.761623701	1.54E-13	1.69E-12
1583	ENSG00000273542.2	H4C12	8362	1.718123335	1.63E-13	1.78E-12
1584	ENSG00000177822.8	TENM3-AS1		1.959988259	1.67E-13	1.82E-12
1585	ENSG00000226539.1	MLXP1		3.788641016	1.70E-13	1.86E-12
1586	ENSG00000233429.9	HOTAIRM1		1.556088638	1.71E-13	1.86E-12
1587	ENSG00000115267.8	IFIH1	64135	1.297689753	1.80E-13	1.97E-12
1588	ENSG00000229227.8		105378289	3.430482574	1.81E-13	1.97E-12
1589	ENSG00000181847.12	TIGIT	201633	1.548735263	1.84E-13	2.00E-12
1590	ENSG00000157680.15	DGKI	9162	1.874560096	1.90E-13	2.06E-12
1591	ENSG00000103253.19	HAGHL	84264	1.565131123	1.93E-13	2.09E-12
1592	ENSG00000197696.10	NMB	4828	1.409262445	1.99E-13	2.16E-12
1593	ENSG00000187475.6	H1-6	3010	3.17504629	1.99E-13	2.16E-12
1594	ENSG00000184357.5	H1-5	3009	1.791514477	2.05E-13	2.22E-12
1595	ENSG00000088340.17	FER1L4		1.704316158	2.06E-13	2.23E-12
1596	ENSG00000188766.12	SPRED3	399473	1.156008056	2.06E-13	2.24E-12
1597	ENSG00000235121.1			2.495435482	2.12E-13	2.30E-12
1598	ENSG00000248787.2			1.729302291	2.15E-13	2.33E-12
1599	ENSG00000186891.14	TNFRSF18	8784	1.631482436	2.17E-13	2.35E-12
1600	ENSG00000259518.2	LINC01583	101929690	3.087425515	2.22E-13	2.39E-12
1601	ENSG00000285697.1			1.406668908	2.29E-13	2.47E-12
1602	ENSG00000229876.1	CASC20	101929244	4.066714064	2.37E-13	2.56E-12
1603	ENSG00000224566.2	CIAO2AP2		2.545065421	2.39E-13	2.58E-12
1604	ENSG00000225637.1			2.506818645	2.42E-13	2.60E-12
1605	ENSG00000280890.1	ELDR		2.883477052	2.54E-13	2.73E-12
1606	ENSG00000180210.15	F2	2147	3.660549947	2.73E-13	2.92E-12
1607	ENSG00000278594.1			2.961877864	2.76E-13	2.96E-12
1608	ENSG00000272941.1			1.277267714	2.76E-13	2.96E-12
1609	ENSG00000138623.10	SEMA7A	8482	1.170509161	2.78E-13	2.97E-12
1610	ENSG00000235126.1			2.769992382	2.79E-13	2.99E-12
1611	ENSG00000223478.1	ZDHHC12-DT		1.012730883	2.89E-13	3.09E-12
1612	ENSG00000269220.1	LINC00528	200298	1.735506207	2.89E-13	3.09E-12
1613	ENSG00000279765.3			1.741238849	2.91E-13	3.11E-12
1614	ENSG00000165682.14	CLEC1B	51266	2.272067771	2.91E-13	3.11E-12
1615	ENSG00000165078.13	CPA6	57094	2.153308227	2.94E-13	3.14E-12
1616	ENSG00000286984.1			3.563770849	3.03E-13	3.23E-12
1617	ENSG00000282122.1	IGHV7-4-1		3.761349871	3.05E-13	3.24E-12
1618	ENSG00000076641.4	PAG1	55824	1.117222327	3.06E-13	3.26E-12
1619	ENSG00000197110.9	IFNL3	282617	3.560505725	3.16E-13	3.36E-12
1620	ENSG00000163499.12	CRYBA2	1412	2.751033415	3.31E-13	3.50E-12
1621	ENSG00000210741.1	MIR196A1	406972	3.574768597	3.46E-13	3.66E-12
1622	ENSG00000197046.12	SIGLEC15	284266	1.934671736	3.50E-13	3.70E-12
1623	ENSG00000182492.16	BGN	633	1.534270902	3.62E-13	3.82E-12
1624	ENSG00000276923.1	LINC03079		2.633055483	3.63E-13	3.84E-12
1625	ENSG00000197461.13	PDGFA	5154	1.021319837	3.63E-13	3.84E-12
1626	ENSG00000164935.6	DCSTAMP	81501	2.577946382	3.63E-13	3.84E-12
1627	ENSG00000276368.2	H2AC14	8331	1.993570651	3.66E-13	3.86E-12
1628	ENSG00000257913.2	DDN-AS1	105369758	1.376653281	3.69E-13	3.89E-12
1629	ENSG00000105613.10	MAST1	22983	1.700207051	3.72E-13	3.92E-12
1630	ENSG00000143226.15	FCGR2A	2212	1.242760393	3.75E-13	3.95E-12
1631	ENSG00000235350.1			3.208954984	3.80E-13	4.00E-12
1632	ENSG00000224963.3	ECMXP		2.614675176	3.81E-13	4.01E-12
1633	ENSG00000268030.1			1.028047936	3.92E-13	4.12E-12
1634	ENSG00000267523.1			1.4805115	4.06E-13	4.26E-12
1635	ENSG00000273415.3	LINC02725	105369563	3.665974447	4.11E-13	4.32E-12
1636	ENSG00000134460.18	IL2RA	3559	1.379976144	4.13E-13	4.33E-12
1637	ENSG00000271788.1			1.79619031	4.14E-13	4.34E-12
1638	ENSG00000232598.2			3.716867663	4.17E-13	4.37E-12
1639	ENSG00000273129.1	PACERR		2.565087044	4.20E-13	4.40E-12
1640	ENSG00000258101.2			1.231019033	4.37E-13	4.57E-12
1641	ENSG00000118946.12	PCDH17	27253	1.259487684	4.40E-13	4.61E-12
1642	ENSG00000212237.1	RNA5SP18		2.502213786	4.41E-13	4.61E-12
1643	ENSG00000070886.12	EPHA8	2046	2.989999445	4.41E-13	4.61E-12
1644	ENSG00000250122.1			3.401570616	4.56E-13	4.76E-12
1645	ENSG00000262668.2			2.91982626	4.60E-13	4.80E-12
1646	ENSG00000082126.18	MPP4	58538	1.985437527	4.81E-13	5.01E-12
1647	ENSG00000131626.19	PPFIA1	8500	1.385041097	4.82E-13	5.02E-12
1648	ENSG00000173391.9	OLR1	4973	1.983997886	4.83E-13	5.02E-12
1649	ENSG00000117598.13	PLPPR5	163404	2.677555308	4.85E-13	5.04E-12
1650	ENSG00000172789.3	HOXC5	3222	2.784684214	4.95E-13	5.13E-12
1651	ENSG00000155495.9	MAGEC1	9947	6.842069369	4.97E-13	5.15E-12
1652	ENSG00000287747.1		124901872	2.905162317	5.03E-13	5.21E-12
1653	ENSG00000130950.14	NUTM2F	54754	2.937499021	5.03E-13	5.21E-12
1654	ENSG00000184937.16	WT1	7490	3.231659254	5.05E-13	5.23E-12
1655	ENSG00000171403.10	KRT9	3857	1.906171563	5.06E-13	5.24E-12
1656	ENSG00000251350.2	LINC02475	100506827	4.01202207	5.50E-13	5.67E-12
1657	ENSG00000174130.12	TLR6	10333	1.174585319	5.68E-13	5.85E-12
1658	ENSG00000232775.6	BMS1P22		3.270733887	5.71E-13	5.87E-12
1659	ENSG00000137142.5	IGFBPL1	347252	2.515694688	5.85E-13	6.01E-12
1660	ENSG00000034510.6	TMSB10	9168	1.182189116	5.89E-13	6.05E-12
1661	ENSG00000090971.5	NAT14	57106	1.178461112	5.95E-13	6.11E-12
1662	ENSG00000230067.3	HSPD1P6		1.312688081	5.99E-13	6.14E-12
1663	ENSG00000176692.8	FOXC2	2303	1.90562932	5.99E-13	6.14E-12
1664	ENSG00000233822.4	H2BC15	8341	1.32292142	6.10E-13	6.25E-12
1665	ENSG00000204632.11	HLA-G	3135	1.718748698	6.16E-13	6.30E-12
1666	ENSG00000224126.2	UBE2SP2		1.753345377	6.16E-13	6.31E-12
1667	ENSG00000253477.5			2.612818656	6.28E-13	6.42E-12
1668	ENSG00000231702.2			3.90313277	6.29E-13	6.44E-12
1669	ENSG00000183615.6	FAM167B	84734	1.025740703	6.34E-13	6.48E-12
1670	ENSG00000123612.16	ACVR1C	130399	1.252478047	6.44E-13	6.58E-12
1671	ENSG00000227066.2		117779438	2.480171288	6.57E-13	6.71E-12
1672	ENSG00000233080.3	LINC01399		3.252461649	6.58E-13	6.72E-12
1673	ENSG00000134369.15	NAV1	89796	1.031346823	6.63E-13	6.76E-12
1674	ENSG00000225096.3		101927293	2.181106712	6.83E-13	6.96E-12
1675	ENSG00000205334.2	LINC01460	100129995	2.634935333	7.02E-13	7.15E-12
1676	ENSG00000127507.18	ADGRE2	30817	1.277849173	7.04E-13	7.16E-12
1677	ENSG00000169594.13	BNC1	646	1.286234644	7.07E-13	7.20E-12
1678	ENSG00000230533.4	LINC03004		2.144486339	7.19E-13	7.31E-12
1679	ENSG00000286062.1		105379003	3.426407727	7.31E-13	7.41E-12
1680	ENSG00000246859.3	STARD4-AS1	100505678	1.395345826	7.31E-13	7.41E-12
1681	ENSG00000169682.18	SPNS1	83985	1.291410515	7.37E-13	7.47E-12
1682	ENSG00000236047.1	RPL13AP12		2.395711667	7.44E-13	7.54E-12
1683	ENSG00000198088.10	NUP62CL	54830	1.083282164	7.47E-13	7.56E-12
1684	ENSG00000172243.18	CLEC7A	64581	1.392148544	7.49E-13	7.58E-12
1685	ENSG00000005961.19	ITGA2B	3674	1.463696394	7.49E-13	7.59E-12
1686	ENSG00000287544.1		105377557	3.657695795	7.58E-13	7.67E-12
1687	ENSG00000231407.6	GORAB-AS1	101928650	1.434562586	7.76E-13	7.85E-12
1688	ENSG00000286351.1			3.482529681	7.92E-13	8.00E-12
1689	ENSG00000242294.6	STAG3L5P		1.272331522	7.93E-13	8.01E-12
1690	ENSG00000140678.17	ITGAX	3687	1.305529766	7.98E-13	8.05E-12
1691	ENSG00000249092.1	PPIAP77		2.239200133	8.23E-13	8.30E-12
1692	ENSG00000163909.8	HEYL	26508	1.229989362	8.24E-13	8.32E-12
1693	ENSG00000138448.12	ITGAV	3685	1.014311639	8.30E-13	8.37E-12
1694	ENSG00000105810.10	CDK6	1021	1.223411129	8.32E-13	8.38E-12
1695	ENSG00000117009.12	KMO	8564	1.385402242	8.36E-13	8.42E-12
1696	ENSG00000206503.13	HLA-A	3105	1.076786308	8.45E-13	8.50E-12
1697	ENSG00000152207.7	CYSLTR2	57105	1.428183139	8.64E-13	8.68E-12
1698	ENSG00000272979.1			1.383205581	8.81E-13	8.85E-12
1699	ENSG00000237609.1	LINC02943		2.338680536	8.82E-13	8.85E-12
1700	ENSG00000257732.1	TXNRD1-AS1	124903002	3.005373669	9.14E-13	9.16E-12
1701	ENSG00000261706.1	LINC00165	727701	3.789288785	9.19E-13	9.20E-12
1702	ENSG00000181104.7	F2R	2149	1.123959993	9.29E-13	9.29E-12
1703	ENSG00000253163.1		107986494	2.314146335	9.33E-13	9.33E-12
1704	ENSG00000232230.1	TPM4P1		3.374086408	9.34E-13	9.34E-12
1705	ENSG00000116819.8	TFAP2E	339488	1.491105861	9.50E-13	9.50E-12
1706	ENSG00000260051.1			1.655432633	9.73E-13	9.72E-12
1707	ENSG00000228705.2	LINC00659		2.629333351	1.02E-12	1.01E-11
1708	ENSG00000047936.11	ROS1	6098	2.82720695	1.02E-12	1.02E-11
1709	ENSG00000102287.19	GABRE	2564	1.500799287	1.03E-12	1.03E-11
1710	ENSG00000224817.2	LINC03046	125775240	3.826832494	1.05E-12	1.05E-11
1711	ENSG00000167889.13	MGAT5B	146664	1.876684471	1.07E-12	1.07E-11
1712	ENSG00000287848.1			3.688937844	1.08E-12	1.08E-11
1713	ENSG00000100314.4	CABP7	164633	2.048049921	1.09E-12	1.08E-11
1714	ENSG00000020633.19	RUNX3	864	1.099721772	1.09E-12	1.08E-11
1715	ENSG00000223872.2		105375610	3.560446764	1.14E-12	1.13E-11
1716	ENSG00000255276.1	RRM1-AS1		2.538515184	1.21E-12	1.20E-11
1717	ENSG00000267172.1			3.053329537	1.22E-12	1.21E-11
1718	ENSG00000253706.5			3.623015638	1.23E-12	1.21E-11
1719	ENSG00000153233.13	PTPRR	5801	1.851055717	1.23E-12	1.22E-11
1720	ENSG00000235643.2	LINC01647	101929181	3.428991475	1.25E-12	1.23E-11
1721	ENSG00000204188.7	GGNBP1		2.242499232	1.33E-12	1.32E-11
1722	ENSG00000233854.2	POU6F2-AS2		3.590397944	1.35E-12	1.33E-11
1723	ENSG00000081985.13	IL12RB2	3595	1.795092942	1.39E-12	1.36E-11
1724	ENSG00000231683.6	KILH	101927136	4.143501372	1.41E-12	1.38E-11
1725	ENSG00000060982.15	BCAT1	586	1.628538726	1.45E-12	1.43E-11
1726	ENSG00000130208.9	APOC1	341	1.928361344	1.47E-12	1.44E-11
1727	ENSG00000283973.2			2.563322085	1.48E-12	1.45E-11
1728	ENSG00000213495.3	RPL26P13		2.022165537	1.54E-12	1.50E-11
1729	ENSG00000273723.1	SUGT1-DT	116435302	1.341449383	1.54E-12	1.51E-11
1730	ENSG00000154118.13	JPH3	57338	2.28075201	1.54E-12	1.51E-11
1731	ENSG00000203760.9	CENPW	387103	1.06895138	1.56E-12	1.52E-11
1732	ENSG00000115009.13	CCL20	6364	2.236762506	1.58E-12	1.54E-11
1733	ENSG00000285904.1			3.156731315	1.61E-12	1.57E-11
1734	ENSG00000225826.2	LINC00626	79100	3.24201788	1.67E-12	1.63E-11
1735	ENSG00000180801.14	ARSJ	79642	1.322900839	1.68E-12	1.64E-11
1736	ENSG00000205359.10	SLCO6A1	133482	2.424330921	1.70E-12	1.66E-11
1737	ENSG00000228793.2		100507336	2.357213048	1.72E-12	1.68E-11
1738	ENSG00000236670.1	KRT18P5		2.091573554	1.74E-12	1.69E-11
1739	ENSG00000241220.1	LINC03109		2.743250237	1.75E-12	1.71E-11
1740	ENSG00000182261.4	NLRP10	338322	2.669986217	1.75E-12	1.71E-11
1741	ENSG00000229970.3			2.686612876	1.79E-12	1.74E-11
1742	ENSG00000135362.14	PRR5L	79899	1.129965989	1.80E-12	1.75E-11
1743	ENSG00000198358.4		101928372	3.290972939	1.81E-12	1.75E-11
1744	ENSG00000182459.5	TEX19	400629	3.008683974	1.86E-12	1.80E-11
1745	ENSG00000256417.1			1.600039268	1.87E-12	1.81E-11
1746	ENSG00000239542.3	RN7SL399P		3.330790581	1.88E-12	1.82E-11
1747	ENSG00000203896.10	LIME1	54923	1.211170006	1.91E-12	1.85E-11
1748	ENSG00000183709.8	IFNL2	282616	3.516928034	1.92E-12	1.85E-11
1749	ENSG00000261587.2	TMEM249	340393	2.682609694	1.95E-12	1.89E-11
1750	ENSG00000112273.7	HDGFL1	154150	4.19287503	2.00E-12	1.93E-11
1751	ENSG00000107187.17	LHX3	8022	3.142004306	2.01E-12	1.94E-11
1752	ENSG00000233435.2	AGGF1P2		2.091911569	2.01E-12	1.94E-11
1753	ENSG00000229020.4	AKR7A2P1		1.854492931	2.02E-12	1.95E-11
1754	ENSG00000170927.15	PKHD1	5314	2.690274859	2.03E-12	1.96E-11
1755	ENSG00000230400.3	LINC01747		2.903606692	2.06E-12	1.99E-11
1756	ENSG00000249069.8			3.204383595	2.07E-12	1.99E-11
1757	ENSG00000095932.7	SMIM24	284422	2.258705897	2.09E-12	2.02E-11
1758	ENSG00000115008.6	IL1A	3552	2.003286037	2.13E-12	2.05E-11
1759	ENSG00000226674.11	TEX41	401014	1.998729583	2.23E-12	2.14E-11
1760	ENSG00000257954.1			1.80808101	2.23E-12	2.14E-11
1761	ENSG00000257219.6	LNCOG	105369848	2.186764439	2.25E-12	2.16E-11
1762	ENSG00000163825.4	RTP3	83597	3.398559323	2.28E-12	2.18E-11
1763	ENSG00000067715.14	SYT1	6857	1.978407422	2.34E-12	2.25E-11
1764	ENSG00000174015.10	CBY2	220082	1.901947115	2.35E-12	2.26E-11
1765	ENSG00000231568.1	S100A11P7		3.508549473	2.37E-12	2.27E-11
1766	ENSG00000176746.6	MAGEB6	158809	5.040804487	2.39E-12	2.29E-11
1767	ENSG00000261326.4	LINC01355		1.334632208	2.40E-12	2.30E-11
1768	ENSG00000272168.8	CASC15	105374971	1.357118554	2.44E-12	2.33E-11
1769	ENSG00000255031.5			1.357693926	2.46E-12	2.35E-11
1770	ENSG00000244124.1	ATP1B3-AS1		1.674875507	2.47E-12	2.36E-11
1771	ENSG00000276931.1			1.037501351	2.52E-12	2.40E-11
1772	ENSG00000123342.16	MMP19	4327	1.260081922	2.54E-12	2.42E-11
1773	ENSG00000135094.11	SDS	10993	1.553355341	2.60E-12	2.48E-11
1774	ENSG00000230773.9		105374593	1.144824243	2.65E-12	2.53E-11
1775	ENSG00000287088.1			4.204717955	2.67E-12	2.54E-11
1776	ENSG00000287937.1		124907871	2.782798258	2.68E-12	2.55E-11
1777	ENSG00000182472.9	CAPN12	147968	1.227862348	2.70E-12	2.57E-11
1778	ENSG00000228709.1	LINC02575		3.39055003	2.71E-12	2.57E-11
1779	ENSG00000253667.2			1.234418655	2.71E-12	2.58E-11
1780	ENSG00000259439.2	LINC01833	107985879	3.765785784	2.72E-12	2.59E-11
1781	ENSG00000135747.11	ZNF670-ZNF695		2.864540204	2.73E-12	2.59E-11
1782	ENSG00000106624.11	AEBP1	165	1.506349109	2.74E-12	2.60E-11
1783	ENSG00000135740.17	SLC9A5	6553	1.073407311	2.75E-12	2.61E-11
1784	ENSG00000157021.9	CIBAR1P1		1.158055147	2.78E-12	2.64E-11
1785	ENSG00000275793.1	RIMBP3	85376	1.511470051	2.79E-12	2.64E-11
1786	ENSG00000256633.1	PDE2A-AS2		2.002610206	2.79E-12	2.65E-11
1787	ENSG00000280179.1			2.259054835	2.80E-12	2.65E-11
1788	ENSG00000273243.1			1.284893837	2.83E-12	2.69E-11
1789	ENSG00000229964.1			3.539585489	2.87E-12	2.72E-11
1790	ENSG00000211897.9	IGHG3		2.405393187	2.90E-12	2.75E-11
1791	ENSG00000242041.1	RPL35AP28		2.018310847	2.92E-12	2.76E-11
1792	ENSG00000238222.3			2.832911963	2.93E-12	2.77E-11
1793	ENSG00000126890.13	CTAG2	30848	6.613004923	2.97E-12	2.81E-11
1794	ENSG00000287511.1			1.56349028	2.97E-12	2.81E-11
1795	ENSG00000244268.1			1.428316705	3.01E-12	2.85E-11
1796	ENSG00000238260.1			1.727218395	3.01E-12	2.85E-11
1797	ENSG00000270557.1			1.68410216	3.02E-12	2.85E-11
1798	ENSG00000275874.1	PICSAR		2.498672552	3.03E-12	2.87E-11
1799	ENSG00000008118.10	CAMK1G	57172	1.329968672	3.11E-12	2.93E-11
1800	ENSG00000233098.9	CCDC144NL-AS1	339260	2.438011186	3.14E-12	2.96E-11
1801	ENSG00000250748.6	MSRB3-AS1	105369187	2.064083671	3.20E-12	3.01E-11
1802	ENSG00000146094.15	DOK3	79930	1.0521445	3.22E-12	3.03E-11
1803	ENSG00000215788.11	TNFRSF25	8718	1.052741153	3.23E-12	3.03E-11
1804	ENSG00000257242.8	LINC01619	256021	1.336055631	3.24E-12	3.05E-11
1805	ENSG00000213556.3			3.180869698	3.25E-12	3.06E-11
1806	ENSG00000121716.20	PILRB	29990	1.547273201	3.32E-12	3.12E-11
1807	ENSG00000121753.12	ADGRB2	576	1.383960952	3.33E-12	3.13E-11
1808	ENSG00000163530.4	DPPA2	151871	5.515156928	3.36E-12	3.15E-11
1809	ENSG00000166446.15	CDYL2	124359	1.096898221	3.38E-12	3.17E-11
1810	ENSG00000280132.1			1.449110491	3.40E-12	3.19E-11
1811	ENSG00000203721.7	LINC00862	554279	1.874389492	3.49E-12	3.26E-11
1812	ENSG00000285637.2			2.999895131	3.49E-12	3.27E-11
1813	ENSG00000262712.1			1.113370452	3.57E-12	3.34E-11
1814	ENSG00000196366.3	C9orf163	158055	1.384495986	3.61E-12	3.38E-11
1815	ENSG00000203772.7	SPRN	503542	1.815984936	3.64E-12	3.40E-11
1816	ENSG00000253187.2	HOXA10-AS	100874323	3.052106071	3.70E-12	3.45E-11
1817	ENSG00000276763.1			2.741130831	3.71E-12	3.46E-11
1818	ENSG00000236045.1			2.989009242	3.78E-12	3.53E-11
1819	ENSG00000231513.3	E2F6P4		4.32523397	3.95E-12	3.68E-11
1820	ENSG00000175841.8	ARB2BP		2.136099064	4.19E-12	3.89E-11
1821	ENSG00000115363.14	EVA1A	84141	1.631530571	4.23E-12	3.94E-11
1822	ENSG00000273703.2	H2BC14	8342	2.749103584	4.30E-12	4.00E-11
1823	ENSG00000273551.1			3.022566082	4.47E-12	4.15E-11
1824	ENSG00000275582.1			1.376929648	4.51E-12	4.18E-11
1825	ENSG00000228107.1			1.444766557	4.53E-12	4.20E-11
1826	ENSG00000287384.1			1.417190478	4.60E-12	4.26E-11
1827	ENSG00000234264.1	DEPDC1-AS1	101927220	1.693173226	4.76E-12	4.40E-11
1828	ENSG00000260052.1			2.129590526	4.80E-12	4.44E-11
1829	ENSG00000178015.5	GPR150	285601	1.546833456	4.80E-12	4.44E-11
1830	ENSG00000115919.15	KYNU	8942	1.705782758	4.83E-12	4.47E-11
1831	ENSG00000127831.11	VIL1	7429	3.018754535	4.84E-12	4.47E-11
1832	ENSG00000251185.1			3.01258039	4.86E-12	4.49E-11
1833	ENSG00000183019.7	MCEMP1	199675	1.86052901	4.87E-12	4.50E-11
1834	ENSG00000231317.1			3.554848641	4.96E-12	4.58E-11
1835	ENSG00000263080.1		105371083	2.046766384	5.03E-12	4.63E-11
1836	ENSG00000245156.1			1.305158355	5.05E-12	4.65E-11
1837	ENSG00000260924.2	LINC01311		1.267735007	5.09E-12	4.68E-11
1838	ENSG00000244300.3	GATA2-AS1		1.625327823	5.23E-12	4.81E-11
1839	ENSG00000171509.16	RXFP1	59350	1.933948952	5.26E-12	4.83E-11
1840	ENSG00000089505.18	CMTM1	113540	1.130468914	5.26E-12	4.83E-11
1841	ENSG00000278987.1			1.296950737	5.28E-12	4.85E-11
1842	ENSG00000261716.2	H2BC20P		1.010860545	5.38E-12	4.93E-11
1843	ENSG00000039560.14	RAI14	26064	1.073613859	5.41E-12	4.96E-11
1844	ENSG00000167912.6	TOX-DT	100505501	1.478039216	5.44E-12	4.99E-11
1845	ENSG00000279369.1			1.468937788	5.58E-12	5.12E-11
1846	ENSG00000204960.7	BLACE	338436	3.019225095	5.60E-12	5.13E-11
1847	ENSG00000197670.6	ZNF217-AS1		1.120093889	5.62E-12	5.15E-11
1848	ENSG00000258902.1			2.760797617	5.66E-12	5.19E-11
1849	ENSG00000167077.13	MEI1	150365	2.083443962	5.77E-12	5.28E-11
1850	ENSG00000253167.1	WASHC5-AS1	106479020	1.880510016	5.77E-12	5.28E-11
1851	ENSG00000026036.22	RTEL1-TNFRSF6B		1.148382701	5.80E-12	5.30E-11
1852	ENSG00000253746.1			1.628258777	5.90E-12	5.39E-11
1853	ENSG00000128045.7	RASL11B	65997	2.012286748	5.93E-12	5.41E-11
1854	ENSG00000227932.2	SELENOOLP		4.277999224	6.06E-12	5.53E-11
1855	ENSG00000286060.1		124905218	3.269374596	6.07E-12	5.53E-11
1856	ENSG00000168078.10	PBK	55872	1.02388649	6.07E-12	5.53E-11
1857	ENSG00000259868.3		102723670	2.604277515	6.14E-12	5.59E-11
1858	ENSG00000267710.9	EDDM13	100506374	1.334169126	6.15E-12	5.60E-11
1859	ENSG00000165905.18	LARGE2	120071	1.16579315	6.17E-12	5.62E-11
1860	ENSG00000229140.11	CCDC26	137196	1.782351564	6.20E-12	5.64E-11
1861	ENSG00000169548.3	ZNF280A	129025	4.192454475	6.33E-12	5.75E-11
1862	ENSG00000224854.3	CDKN2A-AS1		3.161764281	6.41E-12	5.82E-11
1863	ENSG00000261207.1			1.562140062	6.43E-12	5.84E-11
1864	ENSG00000273373.1			1.409993952	6.51E-12	5.91E-11
1865	ENSG00000256083.1	HMGA2-AS2		3.040774345	6.54E-12	5.94E-11
1866	ENSG00000186074.19	CD300LF	146722	1.343039527	6.54E-12	5.94E-11
1867	ENSG00000204531.20	POU5F1	5460	1.305472438	6.56E-12	5.95E-11
1868	ENSG00000158406.5	H4C8	8365	1.368179904	6.67E-12	6.05E-11
1869	ENSG00000242837.1	RPL21P13		3.715806585	6.69E-12	6.06E-11
1870	ENSG00000258733.6	LINC02328		1.483034603	6.76E-12	6.13E-11
1871	ENSG00000273064.1			1.728520832	7.10E-12	6.43E-11
1872	ENSG00000182968.5	SOX1	6656	3.559836292	7.11E-12	6.43E-11
1873	ENSG00000163600.13	ICOS	29851	1.422372198	7.18E-12	6.49E-11
1874	ENSG00000288271.1			3.634841362	7.18E-12	6.50E-11
1875	ENSG00000259158.4	ADAM20P1		1.787876823	7.29E-12	6.58E-11
1876	ENSG00000286502.1		124902301	2.765847159	7.32E-12	6.61E-11
1877	ENSG00000250569.1	NTAN1P2		1.074852361	7.35E-12	6.64E-11
1878	ENSG00000280378.1			1.219409459	7.45E-12	6.73E-11
1879	ENSG00000171877.21	FRMD5	84978	1.788855427	7.56E-12	6.82E-11
1880	ENSG00000184492.6	FOXD4L1	200350	1.594555739	7.59E-12	6.84E-11
1881	ENSG00000253898.1	LINC01419	103352670	5.29474685	7.62E-12	6.86E-11
1882	ENSG00000138472.11	GUCA1C	9626	3.385907133	7.67E-12	6.91E-11
1883	ENSG00000235008.1			2.642300768	7.73E-12	6.96E-11
1884	ENSG00000259590.1	LINC02244	105371018	3.25040519	7.83E-12	7.05E-11
1885	ENSG00000153495.11	TEX29	121793	1.827371008	8.02E-12	7.21E-11
1886	ENSG00000142621.19	FHAD1	114827	1.721809074	8.16E-12	7.34E-11
1887	ENSG00000100767.17	PAPLN	89932	1.225562761	8.25E-12	7.41E-11
1888	ENSG00000254885.1			3.668880664	8.28E-12	7.44E-11
1889	ENSG00000276758.1			2.767640338	8.37E-12	7.51E-11
1890	ENSG00000205560.13	CPT1B	1375	1.432818904	8.72E-12	7.81E-11
1891	ENSG00000243479.3	MNX1-AS1		2.915386747	8.73E-12	7.81E-11
1892	ENSG00000286460.1			3.23496881	9.02E-12	8.07E-11
1893	ENSG00000268230.5			1.54504022	9.04E-12	8.09E-11
1894	ENSG00000085514.16	PILRA	29992	1.264072102	9.11E-12	8.14E-11
1895	ENSG00000162458.13	FBLIM1	54751	1.043100214	9.12E-12	8.15E-11
1896	ENSG00000124092.12	CTCFL	140690	4.694364796	9.23E-12	8.24E-11
1897	ENSG00000263033.2			2.09303648	9.31E-12	8.30E-11
1898	ENSG00000121207.12	LRAT	9227	2.606038761	9.37E-12	8.36E-11
1899	ENSG00000189127.8	ANKRD34B	340120	3.114925464	9.49E-12	8.46E-11
1900	ENSG00000079263.19	SP140	11262	1.495940806	9.54E-12	8.50E-11
1901	ENSG00000164411.12	GJB7	375519	2.382289563	9.63E-12	8.57E-11
1902	ENSG00000136514.3	RTP4	64108	1.359052286	9.84E-12	8.76E-11
1903	ENSG00000101255.11	TRIB3	57761	1.170048219	9.94E-12	8.84E-11
1904	ENSG00000232021.7	LEF1-AS1	641518	1.471483483	1.01E-11	9.00E-11
1905	ENSG00000074276.10	CDHR2	54825	1.148827427	1.03E-11	9.12E-11
1906	ENSG00000174948.6	GPR149	344758	4.744223474	1.03E-11	9.14E-11
1907	ENSG00000224570.1			3.060041503	1.03E-11	9.18E-11
1908	ENSG00000287593.1			1.894964332	1.05E-11	9.29E-11
1909	ENSG00000222041.11	CYTOR	112597	1.111721886	1.05E-11	9.33E-11
1910	ENSG00000171502.15	COL24A1	255631	1.52878827	1.05E-11	9.33E-11
1911	ENSG00000278626.1			3.125472402	1.06E-11	9.35E-11
1912	ENSG00000258713.3	C20orf141	128653	3.521831486	1.06E-11	9.38E-11
1913	ENSG00000226856.7	THORLNC	124907879	1.907919892	1.07E-11	9.46E-11
1914	ENSG00000204866.8	IGFL2	147920	2.294947704	1.07E-11	9.47E-11
1915	ENSG00000169684.13	CHRNA5	1138	1.069082596	1.07E-11	9.47E-11
1916	ENSG00000250274.2		100128059	1.834245893	1.11E-11	9.82E-11
1917	ENSG00000224367.6	OACYLP		1.570404466	1.13E-11	9.97E-11
1918	ENSG00000142227.11	EMP3	2014	1.093858423	1.13E-11	9.97E-11
1919	ENSG00000257556.2	LINC02298	124903398	1.367428362	1.13E-11	9.99E-11
1920	ENSG00000176659.9			2.382011322	1.13E-11	1.00E-10
1921	ENSG00000189057.11	FAM111B	374393	1.090940351	1.14E-11	1.01E-10
1922	ENSG00000050344.9	NFE2L3	9603	1.278103608	1.16E-11	1.02E-10
1923	ENSG00000107105.15	ELAVL2	1993	2.208855414	1.16E-11	1.02E-10
1924	ENSG00000157193.18	LRP8	7804	1.065676266	1.17E-11	1.03E-10
1925	ENSG00000272788.1			2.328609852	1.19E-11	1.05E-10
1926	ENSG00000229754.1	CXCR2P1		2.305597781	1.20E-11	1.05E-10
1927	ENSG00000135318.12	NT5E	4907	1.602323662	1.20E-11	1.06E-10
1928	ENSG00000260941.1	LINC00622	644242	1.609923015	1.20E-11	1.06E-10
1929	ENSG00000179088.15	C12orf42	374470	1.575191215	1.22E-11	1.07E-10
1930	ENSG00000225206.10	MIR137HG	400765	3.31812161	1.22E-11	1.07E-10
1931	ENSG00000287941.1			3.080770236	1.22E-11	1.07E-10
1932	ENSG00000185155.12	MIXL1	83881	1.892739306	1.22E-11	1.07E-10
1933	ENSG00000154898.15	CCDC144CP		2.056255104	1.24E-11	1.08E-10
1934	ENSG00000254403.1	OR10Y1P		3.342105203	1.24E-11	1.09E-10
1935	ENSG00000103569.10	AQP9	366	1.713651759	1.32E-11	1.15E-10
1936	ENSG00000268879.1	IGFL1P1		2.168581013	1.33E-11	1.16E-10
1937	ENSG00000277290.1			1.483291019	1.34E-11	1.17E-10
1938	ENSG00000229835.2	KHSRPP1		2.08797669	1.34E-11	1.17E-10
1939	ENSG00000227748.2			3.358336356	1.37E-11	1.20E-10
1940	ENSG00000231574.6	LINC02015	102724550	2.352983925	1.38E-11	1.20E-10
1941	ENSG00000137825.11	ITPKA	3706	1.417338696	1.38E-11	1.20E-10
1942	ENSG00000213996.13	TM6SF2	53345	1.786969748	1.39E-11	1.21E-10
1943	ENSG00000163206.6	SMCP	4184	3.974544712	1.41E-11	1.23E-10
1944	ENSG00000204681.11	GABBR1	2550	1.005098749	1.41E-11	1.23E-10
1945	ENSG00000135899.19	SP110	3431	1.043046	1.42E-11	1.24E-10
1946	ENSG00000276851.1			2.191770082	1.43E-11	1.25E-10
1947	ENSG00000246876.7	LINC02466	101927282	3.611322377	1.45E-11	1.26E-10
1948	ENSG00000225611.1	CCDC13-AS2		2.633716807	1.46E-11	1.27E-10
1949	ENSG00000163515.6	RETNLB	84666	2.776940902	1.46E-11	1.27E-10
1950	ENSG00000196562.14	SULF2	55959	1.172329021	1.46E-11	1.27E-10
1951	ENSG00000268223.6	ARL14EPL	644100	3.420210175	1.47E-11	1.28E-10
1952	ENSG00000138755.6	CXCL9	4283	2.10410951	1.50E-11	1.30E-10
1953	ENSG00000232555.2			1.869066347	1.52E-11	1.32E-10
1954	ENSG00000171224.9	FAM241B	219738	1.094094636	1.57E-11	1.36E-10
1955	ENSG00000238266.2			2.216963584	1.57E-11	1.36E-10
1956	ENSG00000276272.1			1.665144824	1.59E-11	1.37E-10
1957	ENSG00000225968.7	ELFN1	392617	1.518166875	1.59E-11	1.38E-10
1958	ENSG00000196604.13	POTEF	728378	2.03245983	1.61E-11	1.39E-10
1959	ENSG00000185885.16	IFITM1	8519	1.223058349	1.62E-11	1.40E-10
1960	ENSG00000141668.10	CBLN2	147381	2.862988309	1.63E-11	1.41E-10
1961	ENSG00000186340.16	THBS2	7058	1.80144483	1.66E-11	1.43E-10
1962	ENSG00000163794.6	UCN	7349	1.436704389	1.68E-11	1.45E-10
1963	ENSG00000287743.1			2.309685271	1.68E-11	1.45E-10
1964	ENSG00000246640.1	PICART1	284080	1.208864494	1.70E-11	1.46E-10
1965	ENSG00000286133.2			2.666289727	1.72E-11	1.48E-10
1966	ENSG00000287535.1			2.167983457	1.72E-11	1.48E-10
1967	ENSG00000287591.1			3.105065697	1.75E-11	1.50E-10
1968	ENSG00000207547.1	MIR25	407014	1.587497633	1.76E-11	1.51E-10
1969	ENSG00000039600.11	SOX30	11063	2.226982758	1.82E-11	1.57E-10
1970	ENSG00000205116.3	TMEM88B	643965	2.562012151	1.88E-11	1.61E-10
1971	ENSG00000103522.16	IL21R	50615	1.484121489	1.89E-11	1.62E-10
1972	ENSG00000237950.3	LINC02918	101929609	1.139927476	1.91E-11	1.63E-10
1973	ENSG00000279821.1			1.379402804	1.92E-11	1.64E-10
1974	ENSG00000280414.1			3.076345325	1.95E-11	1.67E-10
1975	ENSG00000273568.1			1.262711301	1.98E-11	1.69E-10
1976	ENSG00000255837.1	TAS2R20	259295	1.436882125	1.99E-11	1.70E-10
1977	ENSG00000255992.1	PUS1-AS1		1.211343995	2.03E-11	1.73E-10
1978	ENSG00000238098.9	ABCA17P		2.587974634	2.08E-11	1.77E-10
1979	ENSG00000183486.13	MX2	4600	1.385070661	2.09E-11	1.78E-10
1980	ENSG00000237813.4	CAV2-DT	107986838	1.582078768	2.17E-11	1.84E-10
1981	ENSG00000204316.13	MRPL38	64978	1.053649597	2.21E-11	1.88E-10
1982	ENSG00000277157.2	H4C4	8360	1.526196467	2.24E-11	1.90E-10
1983	ENSG00000224409.1			1.64216237	2.26E-11	1.92E-10
1984	ENSG00000127533.4	F2RL3	9002	1.132208844	2.29E-11	1.95E-10
1985	ENSG00000184368.16	MAP7D2	256714	2.591001973	2.30E-11	1.95E-10
1986	ENSG00000162745.10	OLFML2B	25903	1.405241904	2.34E-11	1.99E-10
1987	ENSG00000180279.7	TMEM277P		1.326419004	2.34E-11	1.99E-10
1988	ENSG00000249685.1			1.165576071	2.40E-11	2.04E-10
1989	ENSG00000075429.9	CACNG5	27091	2.8351613	2.40E-11	2.04E-10
1990	ENSG00000227373.6	RABGAP1L-DT		1.155063646	2.45E-11	2.07E-10
1991	ENSG00000249196.6	TMEM132D-AS1	283352	5.360684081	2.45E-11	2.08E-10
1992	ENSG00000250771.2			2.140468524	2.48E-11	2.10E-10
1993	ENSG00000279844.1			3.117792324	2.49E-11	2.11E-10
1994	ENSG00000278588.2	H2BC10	8346	2.415855179	2.57E-11	2.17E-10
1995	ENSG00000267287.1			2.252213664	2.58E-11	2.18E-10
1996	ENSG00000187997.11	C17orf99	100141515	1.423727457	2.59E-11	2.19E-10
1997	ENSG00000275560.1			1.176655966	2.59E-11	2.19E-10
1998	ENSG00000188290.11	HES4	57801	1.108505868	2.60E-11	2.19E-10
1999	ENSG00000286194.1			1.121854191	2.60E-11	2.20E-10
2000	ENSG00000255462.1		124902632	1.781415029	2.62E-11	2.21E-10
2001	ENSG00000136286.16	MYO1G	64005	1.257427963	2.63E-11	2.22E-10
2002	ENSG00000234235.1	BOK-AS1		4.701869829	2.80E-11	2.36E-10
2003	ENSG00000108688.11	CCL7	6354	1.961857599	2.84E-11	2.39E-10
2004	ENSG00000258590.5	NBEAP1		2.624896435	2.91E-11	2.45E-10
2005	ENSG00000279347.1			1.840986039	2.95E-11	2.48E-10
2006	ENSG00000132432.14	SEC61G	23480	1.340994295	3.01E-11	2.52E-10
2007	ENSG00000205300.3			3.019976144	3.08E-11	2.59E-10
2008	ENSG00000229808.1			1.16102114	3.16E-11	2.65E-10
2009	ENSG00000285103.2	EMICERI		1.540196235	3.23E-11	2.71E-10
2010	ENSG00000166033.13	HTRA1	5654	1.217161528	3.26E-11	2.73E-10
2011	ENSG00000279611.1			2.194885835	3.27E-11	2.74E-10
2012	ENSG00000229373.9	LINC00452	643365	3.483271298	3.42E-11	2.86E-10
2013	ENSG00000237015.1			1.40428587	3.44E-11	2.88E-10
2014	ENSG00000277586.4	NEFL	4747	2.111127049	3.44E-11	2.88E-10
2015	ENSG00000225872.4	TEX14BP		1.978043014	3.49E-11	2.92E-10
2016	ENSG00000066923.17	STAG3	10734	2.199403058	3.56E-11	2.97E-10
2017	ENSG00000100302.7	RASD2	23551	1.749523669	3.63E-11	3.03E-10
2018	ENSG00000262904.1	TMPOP2		1.494412836	3.64E-11	3.03E-10
2019	ENSG00000249695.6		574538	2.241775317	3.68E-11	3.07E-10
2020	ENSG00000275888.1			1.195089542	3.70E-11	3.08E-10
2021	ENSG00000160282.14	FTCD	10841	2.525784673	3.72E-11	3.09E-10
2022	ENSG00000221947.8	XKR9	389668	1.578756985	3.74E-11	3.11E-10
2023	ENSG00000121075.11	TBX4	9496	2.300012273	3.81E-11	3.16E-10
2024	ENSG00000260862.1		101928446	2.54844167	3.81E-11	3.16E-10
2025	ENSG00000123569.8	H2BW1	158983	3.479734956	3.86E-11	3.20E-10
2026	ENSG00000270882.2	H4C14	8370	1.448263428	3.90E-11	3.23E-10
2027	ENSG00000204410.16	MSH5	4439	1.223598546	3.93E-11	3.26E-10
2028	ENSG00000233441.6	CYP2AB1P		3.320206626	4.02E-11	3.33E-10
2029	ENSG00000066405.13	CLDN18	51208	1.864309243	4.04E-11	3.35E-10
2030	ENSG00000185070.11	FLRT2	23768	1.525435677	4.05E-11	3.35E-10
2031	ENSG00000165972.13	CCDC38	120935	2.236347955	4.16E-11	3.44E-10
2032	ENSG00000152192.8	POU4F1	5457	2.926882961	4.20E-11	3.47E-10
2033	ENSG00000253838.1			3.257642712	4.22E-11	3.48E-10
2034	ENSG00000167105.8	TMEM92	162461	1.458301862	4.22E-11	3.49E-10
2035	ENSG00000275481.1			1.534364312	4.26E-11	3.52E-10
2036	ENSG00000241679.2			2.19531872	4.29E-11	3.54E-10
2037	ENSG00000273343.1			1.159015614	4.29E-11	3.54E-10
2038	ENSG00000132297.12	HHLA1	10086	2.601707198	4.30E-11	3.54E-10
2039	ENSG00000271287.1	BCRP9		5.225600531	4.35E-11	3.59E-10
2040	ENSG00000160200.18	CBS	875	2.214233634	4.42E-11	3.64E-10
2041	ENSG00000134365.13	CFHR4	10877	3.849368689	4.45E-11	3.66E-10
2042	ENSG00000154277.13	UCHL1	7345	2.145514846	4.54E-11	3.74E-10
2043	ENSG00000219039.2			2.784671621	4.66E-11	3.83E-10
2044	ENSG00000149243.16	KLHL35	283212	1.921492697	4.67E-11	3.84E-10
2045	ENSG00000115194.11	SLC30A3	7781	1.844616485	4.67E-11	3.84E-10
2046	ENSG00000280113.2			2.081899751	4.68E-11	3.85E-10
2047	ENSG00000244573.3	RPL30P11		2.800084197	4.72E-11	3.87E-10
2048	ENSG00000268186.1	ZNF114-AS1		2.851385687	4.75E-11	3.90E-10
2049	ENSG00000259527.2	LINC00052	145978	3.991808039	4.84E-11	3.97E-10
2050	ENSG00000284700.1			4.08213365	4.92E-11	4.03E-10
2051	ENSG00000259341.2	LINC03080		1.542433798	5.11E-11	4.18E-10
2052	ENSG00000128422.17	KRT17	3872	1.507237655	5.31E-11	4.34E-10
2053	ENSG00000139344.8	AMDHD1	144193	2.220782977	5.32E-11	4.34E-10
2054	ENSG00000269758.1			2.360843116	5.46E-11	4.45E-10
2055	ENSG00000270061.1			2.019159289	5.48E-11	4.47E-10
2056	ENSG00000259760.1			3.33224623	5.52E-11	4.49E-10
2057	ENSG00000278041.1			3.970587564	5.53E-11	4.51E-10
2058	ENSG00000254349.6	MIR2052HG	441355	2.246185548	5.56E-11	4.53E-10
2059	ENSG00000215369.3	RPL37P3		2.727064647	5.57E-11	4.53E-10
2060	ENSG00000288610.1			2.67897672	5.67E-11	4.61E-10
2061	ENSG00000241343.10	RPL36A	6173	1.004108697	5.68E-11	4.62E-10
2062	ENSG00000255893.1			1.526380885	5.75E-11	4.67E-10
2063	ENSG00000188372.15	ZP3	7784	1.107019394	5.88E-11	4.77E-10
2064	ENSG00000234146.1	AKAP8P1		2.954065789	5.90E-11	4.79E-10
2065	ENSG00000229912.1			2.513950499	5.99E-11	4.85E-10
2066	ENSG00000198883.12	PNMA5	114824	3.249355104	6.04E-11	4.89E-10
2067	ENSG00000273367.1			2.121909941	6.08E-11	4.92E-10
2068	ENSG00000184984.10	CHRM5	1133	2.103526965	6.14E-11	4.96E-10
2069	ENSG00000123407.4	HOXC12	3228	4.421369704	6.17E-11	4.98E-10
2070	ENSG00000187144.11	SPATA21	374955	3.506275569	6.26E-11	5.05E-10
2071	ENSG00000261051.1			1.333480379	6.37E-11	5.13E-10
2072	ENSG00000239093.1		124905594	2.861867698	6.43E-11	5.17E-10
2073	ENSG00000173727.12	FAUP4		1.171059179	6.47E-11	5.20E-10
2074	ENSG00000287299.1			1.113813367	6.47E-11	5.20E-10
2075	ENSG00000072182.13	ASIC4	55515	2.174484698	6.51E-11	5.23E-10
2076	ENSG00000169432.18	SCN9A	6335	2.330261632	6.72E-11	5.40E-10
2077	ENSG00000255400.1	LINC02989		3.1397391	6.75E-11	5.42E-10
2078	ENSG00000283686.2			3.387223962	6.77E-11	5.44E-10
2079	ENSG00000181433.9	SAGE1	55511	5.248257444	6.80E-11	5.45E-10
2080	ENSG00000229757.2	RPL7AP21		2.191514037	6.86E-11	5.50E-10
2081	ENSG00000204778.4	ZNG1DP		1.364779977	6.97E-11	5.58E-10
2082	ENSG00000113430.10	IRX4	50805	1.600490706	7.09E-11	5.68E-10
2083	ENSG00000011422.12	PLAUR	5329	1.044553207	7.12E-11	5.70E-10
2084	ENSG00000170909.13	OSCAR	126014	1.091938084	7.23E-11	5.78E-10
2085	ENSG00000238840.1	U8		3.982529796	7.27E-11	5.81E-10
2086	ENSG00000266560.5		100533997	3.239575781	7.28E-11	5.81E-10
2087	ENSG00000176510.5	OR10AC1		2.304976847	7.35E-11	5.87E-10
2088	ENSG00000017483.15	SLC38A5	92745	1.550892629	7.45E-11	5.94E-10
2089	ENSG00000234838.3	PPIAP78		2.763963249	7.48E-11	5.96E-10
2090	ENSG00000100350.15	FOXRED2	80020	1.048184664	7.49E-11	5.96E-10
2091	ENSG00000158714.11	SLAMF8	56833	1.320399917	7.50E-11	5.97E-10
2092	ENSG00000229962.2		105372753	3.586868953	7.54E-11	6.00E-10
2093	ENSG00000228318.3	MX1-AS1		1.962913445	7.54E-11	6.00E-10
2094	ENSG00000133026.13	MYH10	4628	1.006716147	7.58E-11	6.02E-10
2095	ENSG00000201772.1	SNORA5C	677796	1.087154049	7.60E-11	6.04E-10
2096	ENSG00000270977.2			1.844038423	7.67E-11	6.09E-10
2097	ENSG00000185933.7	CALHM1	255022	2.775204996	7.77E-11	6.17E-10
2098	ENSG00000186818.12	LILRB4	11006	1.524671929	7.86E-11	6.23E-10
2099	ENSG00000287895.1			2.197231734	7.95E-11	6.30E-10
2100	ENSG00000158865.12	SLC5A11	115584	2.316066692	8.30E-11	6.56E-10
2101	ENSG00000231625.4	SLC47A1P2		2.693768728	8.39E-11	6.64E-10
2102	ENSG00000253669.4	GASAL1		1.421602768	8.46E-11	6.69E-10
2103	ENSG00000187902.11	SHISA7	729956	2.181249389	8.51E-11	6.73E-10
2104	ENSG00000261762.1			1.058540699	8.55E-11	6.75E-10
2105	ENSG00000219410.6			1.446167702	8.78E-11	6.93E-10
2106	ENSG00000259820.1			1.071720461	8.79E-11	6.93E-10
2107	ENSG00000103269.14	RHBDL1	9028	1.519242288	8.86E-11	6.98E-10
2108	ENSG00000163286.9	ALPG	251	3.249475574	8.89E-11	7.01E-10
2109	ENSG00000182118.8	FAM89A	375061	1.034177218	8.96E-11	7.06E-10
2110	ENSG00000139515.6	PDX1	3651	3.499037856	9.13E-11	7.18E-10
2111	ENSG00000285201.1			3.795688873	9.31E-11	7.32E-10
2112	ENSG00000146678.10	IGFBP1	3484	2.462133594	9.36E-11	7.36E-10
2113	ENSG00000280239.1			1.29692807	9.47E-11	7.44E-10
2114	ENSG00000279452.1			1.340491165	9.51E-11	7.47E-10
2115	ENSG00000224057.1	EGFR-AS1	100507500	1.849776551	9.62E-11	7.55E-10
2116	ENSG00000226954.1			2.954470818	9.69E-11	7.61E-10
2117	ENSG00000237452.3	MEIOSIN	388553	2.160627372	9.77E-11	7.66E-10
2118	ENSG00000254413.8	CHKB-CPT1B		1.857937171	9.86E-11	7.73E-10
2119	ENSG00000270903.1	HNRNPA3P9		1.72615425	9.88E-11	7.75E-10
2120	ENSG00000170848.16	PSG6	5675	3.523869212	1.00E-10	7.85E-10
2121	ENSG00000106633.17	GCK	2645	1.848855717	1.00E-10	7.87E-10
2122	ENSG00000183876.9	ARSI	340075	1.546600745	1.01E-10	7.90E-10
2123	ENSG00000028277.21	POU2F2	5452	1.14959832	1.01E-10	7.94E-10
2124	ENSG00000273162.1		107984262	1.150786933	1.03E-10	8.08E-10
2125	ENSG00000242242.6	NECTIN3-AS1		2.007940903	1.04E-10	8.13E-10
2126	ENSG00000161609.10	KASH5	147872	3.429790811	1.04E-10	8.16E-10
2127	ENSG00000124493.14	GRM4	2914	1.984706973	1.07E-10	8.34E-10
2128	ENSG00000162572.21	SCNN1D	6339	1.827185542	1.07E-10	8.36E-10
2129	ENSG00000220323.4	H2BC19P		1.053547251	1.07E-10	8.38E-10
2130	ENSG00000213700.3	RPL17P50		1.059019534	1.07E-10	8.39E-10
2131	ENSG00000231666.7	LINC01704	646268	1.974915733	1.08E-10	8.42E-10
2132	ENSG00000038427.16	VCAN	1462	1.632128892	1.09E-10	8.52E-10
2133	ENSG00000185842.15	DNAH14	127602	1.60699533	1.11E-10	8.62E-10
2134	ENSG00000232000.3	CLCN3P1		1.277214597	1.11E-10	8.64E-10
2135	ENSG00000188984.12	AADACL3	126767	4.550192712	1.11E-10	8.68E-10
2136	ENSG00000258927.2			4.34495036	1.16E-10	9.04E-10
2137	ENSG00000267750.6	RUNDC3A-AS1		1.56751737	1.16E-10	9.04E-10
2138	ENSG00000288074.1			3.539236917	1.16E-10	9.04E-10
2139	ENSG00000250067.12	YJEFN3	374887	1.613494931	1.18E-10	9.18E-10
2140	ENSG00000188242.4	SLC9A3-OT1	25845	1.609062625	1.19E-10	9.28E-10
2141	ENSG00000160471.13	COX6B2	125965	1.688855567	1.21E-10	9.38E-10
2142	ENSG00000071909.19	MYO3B	140469	1.816073134	1.22E-10	9.48E-10
2143	ENSG00000125820.6	NKX2-2	4821	4.743150238	1.22E-10	9.50E-10
2144	ENSG00000222017.1			1.480625995	1.24E-10	9.63E-10
2145	ENSG00000261798.1		101929536	2.283917026	1.25E-10	9.65E-10
2146	ENSG00000265800.1			1.167717828	1.26E-10	9.79E-10
2147	ENSG00000269924.1			1.770329032	1.27E-10	9.80E-10
2148	ENSG00000241634.1	RPL13P8		1.479697886	1.27E-10	9.86E-10
2149	ENSG00000253161.5	LINC01605	100507420	1.432544668	1.28E-10	9.92E-10
2150	ENSG00000225684.4	FAM225B	100128385	1.541902244	1.32E-10	1.02268E-09
2151	ENSG00000135097.7	MSI1	4440	2.102084902	1.38E-10	1.06067E-09
2152	ENSG00000154096.14	THY1	7070	1.309127835	1.38E-10	1.06774E-09
2153	ENSG00000177807.10	KCNJ10	3766	1.91877339	1.39E-10	1.07256E-09
2154	ENSG00000250735.5			4.084201648	1.40E-10	1.07655E-09
2155	ENSG00000277241.1	FGFR3P4		2.985491887	1.41E-10	1.08762E-09
2156	ENSG00000101311.16	FERMT1	55612	1.053694805	1.43E-10	1.09919E-09
2157	ENSG00000251629.7	LINC02241	105374676	3.830599884	1.45E-10	1.11814E-09
2158	ENSG00000257869.1			4.778737721	1.46E-10	1.12656E-09
2159	ENSG00000287530.1			2.504985207	1.47E-10	1.12967E-09
2160	ENSG00000184995.7	IFNE	338376	1.902167529	1.49E-10	1.14439E-09
2161	ENSG00000234883.6	MIR155HG		1.388429157	1.51E-10	1.16091E-09
2162	ENSG00000162975.5	KCNF1	3754	1.85525188	1.52E-10	1.16448E-09
2163	ENSG00000224251.6			2.455785067	1.54E-10	1.1836E-09
2164	ENSG00000108465.15	CDK5RAP3	80279	1.2892668	1.54E-10	1.18453E-09
2165	ENSG00000169750.9	RAC3	5881	1.345513241	1.54E-10	1.18491E-09
2166	ENSG00000224431.1			1.679544893	1.55E-10	1.18941E-09
2167	ENSG00000180953.11	ST20		1.221696434	1.55E-10	1.19083E-09
2168	ENSG00000278000.1			1.345874589	1.58E-10	1.21119E-09
2169	ENSG00000221539.1	SNORD99		1.60840474	1.61E-10	1.23658E-09
2170	ENSG00000105370.8	LIM2	3982	3.646924674	1.62E-10	1.23975E-09
2171	ENSG00000286919.1			2.835722186	1.62E-10	1.24155E-09
2172	ENSG00000111886.11	GABRR2	2570	1.228248011	1.62E-10	1.24174E-09
2173	ENSG00000268895.6	A1BG-AS1	503538	1.21390383	1.69E-10	1.29119E-09
2174	ENSG00000089225.20	TBX5	6910	3.130025873	1.72E-10	1.31594E-09
2175	ENSG00000279277.2			1.300586806	1.75E-10	1.34097E-09
2176	ENSG00000261170.2		107984827	1.04679232	1.76E-10	1.34296E-09
2177	ENSG00000160963.14	COL26A1	136227	1.835582597	1.79E-10	1.37089E-09
2178	ENSG00000111087.10	GLI1	2735	1.731196177	1.82E-10	1.3932E-09
2179	ENSG00000166448.15	TMEM130	222865	1.504178737	1.83E-10	1.39572E-09
2180	ENSG00000106536.21	POU6F2	11281	2.921008766	1.83E-10	1.39744E-09
2181	ENSG00000113083.15	LOX	4015	1.349397782	1.86E-10	1.41437E-09
2182	ENSG00000225506.2	CYP4A22-AS1		1.292865502	1.90E-10	1.44408E-09
2183	ENSG00000274997.2	H2AC12	85235	1.831133345	1.90E-10	1.44702E-09
2184	ENSG00000259471.1	LINC01169	102723165	3.818473747	1.91E-10	1.45237E-09
2185	ENSG00000244486.9	SCARF2	91179	1.356086553	1.92E-10	1.46223E-09
2186	ENSG00000221598.3	MIR1249	100302149	2.422759591	1.93E-10	1.4694E-09
2187	ENSG00000069696.7	DRD4	1815	1.470558315	1.95E-10	1.4845E-09
2188	ENSG00000100341.12	PNPLA5	150379	3.651595071	1.99E-10	1.51315E-09
2189	ENSG00000137496.18	IL18BP	10068	1.142171907	2.01E-10	1.52831E-09
2190	ENSG00000164283.13	ESM1	11082	1.348322222	2.03E-10	1.53966E-09
2191	ENSG00000244215.2	LINC02016	101927123	3.904798249	2.06E-10	1.56114E-09
2192	ENSG00000272800.3			1.007983994	2.07E-10	1.56808E-09
2193	ENSG00000118113.12	MMP8	4317	2.299499537	2.07E-10	1.56809E-09
2194	ENSG00000227722.2			2.440398447	2.08E-10	1.57907E-09
2195	ENSG00000269526.1	ERVV-1	147664	3.48465133	2.10E-10	1.59394E-09
2196	ENSG00000186831.11	KRT17P2		1.587556632	2.15E-10	1.62953E-09
2197	ENSG00000238045.9	MVP-DT		1.047999008	2.17E-10	1.6391E-09
2198	ENSG00000232006.9			1.195002838	2.18E-10	1.64703E-09
2199	ENSG00000261122.6	LINC02167	400533	4.203750944	2.20E-10	1.66163E-09
2200	ENSG00000286868.1			3.747409242	2.24E-10	1.69021E-09
2201	ENSG00000113721.14	PDGFRB	5159	1.209623293	2.26E-10	1.70017E-09
2202	ENSG00000103313.13	MEFV	4210	1.472352141	2.29E-10	1.72255E-09
2203	ENSG00000248103.1			3.942716759	2.32E-10	1.74837E-09
2204	ENSG00000270480.1			1.965314438	2.38E-10	1.79121E-09
2205	ENSG00000249406.3	TILAM		2.594899969	2.39E-10	1.7934E-09
2206	ENSG00000253755.1	IGHGP		2.22620203	2.39E-10	1.79919E-09
2207	ENSG00000196581.11	AJAP1	55966	2.020904432	2.40E-10	1.80022E-09
2208	ENSG00000285972.1	CERNA2	642934	1.279475216	2.44E-10	1.83212E-09
2209	ENSG00000142920.17	AZIN2	113451	1.094701833	2.44E-10	1.83221E-09
2210	ENSG00000287453.1			5.296622933	2.48E-10	1.86196E-09
2211	ENSG00000231870.4	KRT17P3		2.078076707	2.48E-10	1.8627E-09
2212	ENSG00000278875.1			2.206896429	2.58E-10	1.9333E-09
2213	ENSG00000125538.12	IL1B	3553	1.58873187	2.58E-10	1.9333E-09
2214	ENSG00000280069.1			1.604961514	2.59E-10	1.93842E-09
2215	ENSG00000286538.1			2.93216279	2.62E-10	1.95521E-09
2216	ENSG00000116014.10	KISS1R	84634	2.08965989	2.62E-10	1.95776E-09
2217	ENSG00000243635.1	EZRP1		3.023532675	2.66E-10	1.98373E-09
2218	ENSG00000261211.1			1.250562883	2.66E-10	1.98792E-09
2219	ENSG00000253317.1			2.639337102	2.69E-10	2.00557E-09
2220	ENSG00000179841.8	AKAP5	9495	1.090830303	2.78E-10	2.06813E-09
2221	ENSG00000102387.16	TAF7L	54457	2.505091278	2.83E-10	2.10648E-09
2222	ENSG00000155966.14	AFF2	2334	2.018102534	2.83E-10	2.11041E-09
2223	ENSG00000184697.7	CLDN6	9074	2.383711282	2.88E-10	2.14438E-09
2224	ENSG00000261732.1			1.387732975	2.92E-10	2.17079E-09
2225	ENSG00000227234.2	SPANXB1	728695	4.543943876	2.92E-10	2.17141E-09
2226	ENSG00000249378.6	LINC01060		1.71647067	2.92E-10	2.17172E-09
2227	ENSG00000184163.3	C1QTNF12	388581	2.014611882	2.95E-10	2.19362E-09
2228	ENSG00000243766.8	HOTTIP		3.081614069	2.97E-10	2.20896E-09
2229	ENSG00000135919.14	SERPINE2	5270	1.435054064	2.98E-10	2.2159E-09
2230	ENSG00000203663.4	OR2L2	26246	3.393356662	2.99E-10	2.22169E-09
2231	ENSG00000235180.1	LINC00601	101101772	2.599382801	3.01E-10	2.23579E-09
2232	ENSG00000111863.13	ADTRP	84830	1.502408741	3.06E-10	2.26911E-09
2233	ENSG00000124196.6	GTSF1L	149699	2.05041707	3.10E-10	2.30164E-09
2234	ENSG00000186854.11	TRABD2A	129293	1.404333548	3.14E-10	2.32634E-09
2235	ENSG00000178860.8	MSC	9242	1.353288986	3.25E-10	2.40393E-09
2236	ENSG00000224875.2	EML4-AS1	102723824	1.89098529	3.29E-10	2.43292E-09
2237	ENSG00000242085.1	RPS20P33		1.257224801	3.31E-10	2.44758E-09
2238	ENSG00000286552.1			3.165223947	3.34E-10	2.47321E-09
2239	ENSG00000256694.1			1.39193931	3.42E-10	2.52707E-09
2240	ENSG00000227934.1			1.595563678	3.50E-10	2.58362E-09
2241	ENSG00000163631.17	ALB	213	2.553079257	3.50E-10	2.58362E-09
2242	ENSG00000278635.1			2.037053158	3.58E-10	2.64199E-09
2243	ENSG00000231482.3			2.108732395	3.59E-10	2.64375E-09
2244	ENSG00000157601.14	MX1	4599	1.28752493	3.59E-10	2.64504E-09
2245	ENSG00000267698.1		101927572	1.243798765	3.60E-10	2.65271E-09
2246	ENSG00000106631.8	MYL7	58498	2.756503368	3.64E-10	2.6798E-09
2247	ENSG00000257270.1			1.179327129	3.67E-10	2.6963E-09
2248	ENSG00000158164.7	TMSB15A	11013	1.94488275	3.75E-10	2.75916E-09
2249	ENSG00000250420.8	AACSP1		2.238523981	3.85E-10	2.82167E-09
2250	ENSG00000203585.4	LINC02408	100507175	1.521516916	3.89E-10	2.85066E-09
2251	ENSG00000268942.2	CKS1BP3		2.6156795	3.90E-10	2.86159E-09
2252	ENSG00000104419.17	NDRG1	10397	1.117545377	3.95E-10	2.89392E-09
2253	ENSG00000104998.4	IL27RA	9466	1.127658187	3.99E-10	2.92015E-09
2254	ENSG00000140368.13	PSTPIP1	9051	1.050364049	4.05E-10	2.96223E-09
2255	ENSG00000100429.18	HDAC10	83933	1.095833919	4.09E-10	2.99058E-09
2256	ENSG00000236499.2	LINC00896	150197	1.921624061	4.12E-10	3.00795E-09
2257	ENSG00000167723.15	TRPV3	162514	1.568209965	4.18E-10	3.0528E-09
2258	ENSG00000121068.14	TBX2	6909	1.212189243	4.23E-10	3.08499E-09
2259	ENSG00000111181.12	SLC6A12	6539	1.394163434	4.40E-10	3.2064E-09
2260	ENSG00000287352.1		102724858	3.182716973	4.47E-10	3.25981E-09
2261	ENSG00000231476.1		105375303	5.876952479	4.49E-10	3.26704E-09
2262	ENSG00000120949.15	TNFRSF8	943	1.32635207	4.51E-10	3.28484E-09
2263	ENSG00000157060.16	SHCBP1L	81626	4.715769625	4.60E-10	3.34311E-09
2264	ENSG00000224141.6	MIR548XHG		5.508540034	4.60E-10	3.34586E-09
2265	ENSG00000228109.1	MELTF-AS1	100507057	1.375711413	4.64E-10	3.36807E-09
2266	ENSG00000279862.1			1.890754781	4.66E-10	3.38642E-09
2267	ENSG00000168124.2	OR1F1	4992	2.840103604	4.81E-10	3.49386E-09
2268	ENSG00000175305.17	CCNE2	9134	1.004205312	4.95E-10	3.58939E-09
2269	ENSG00000260996.1			1.167407217	4.97E-10	3.59789E-09
2270	ENSG00000285077.2	ARHGAP11B	89839	1.163492209	5.01E-10	3.62539E-09
2271	ENSG00000114770.17	ABCC5	10057	1.179039009	5.08E-10	3.66993E-09
2272	ENSG00000205277.9	MUC12	10071	1.506859947	5.13E-10	3.7054E-09
2273	ENSG00000260084.1			2.247621819	5.21E-10	3.76234E-09
2274	ENSG00000279786.1			1.787607181	5.36E-10	3.86602E-09
2275	ENSG00000114948.13	ADAM23	8745	1.726120508	5.36E-10	3.86602E-09
2276	ENSG00000270012.1			1.100065022	5.40E-10	3.88878E-09
2277	ENSG00000083857.14	FAT1	2195	1.125526096	5.47E-10	3.94283E-09
2278	ENSG00000237491.10	LINC01409		1.028150295	5.50E-10	3.96297E-09
2279	ENSG00000137251.16	TINAG	27283	2.976371987	5.56E-10	4.00322E-09
2280	ENSG00000225678.2			1.917197752	5.76E-10	4.13585E-09
2281	ENSG00000171956.7	FOXB1	27023	2.091052937	5.80E-10	4.16185E-09
2282	ENSG00000178199.14	ZC3H12D	340152	1.157875705	5.83E-10	4.18653E-09
2283	ENSG00000287326.1		728158	2.617341958	5.91E-10	4.23716E-09
2284	ENSG00000275092.1			1.611420145	5.97E-10	4.27956E-09
2285	ENSG00000254545.1			2.564037199	5.97E-10	4.27956E-09
2286	ENSG00000138152.10	BTBD16	118663	2.027863089	5.99E-10	4.29379E-09
2287	ENSG00000250102.6	LINC02377	105377424	3.473258469	6.03E-10	4.31931E-09
2288	ENSG00000105290.13	APLP1	333	1.36595525	6.09E-10	4.35654E-09
2289	ENSG00000286403.1			2.330466534	6.12E-10	4.37942E-09
2290	ENSG00000006606.9	CCL26	10344	1.894316972	6.18E-10	4.41944E-09
2291	ENSG00000168065.16	SLC22A11	55867	2.28619956	6.19E-10	4.42482E-09
2292	ENSG00000243753.5	HLA-L		1.191498622	6.20E-10	4.43163E-09
2293	ENSG00000235277.1			2.088874287	6.28E-10	4.48321E-09
2294	ENSG00000152661.9	GJA1	2697	1.201001578	6.35E-10	4.53471E-09
2295	ENSG00000271447.6	MMP28	79148	1.527740419	6.61E-10	4.71473E-09
2296	ENSG00000224816.1	NAP1L4P2		3.120641124	6.64E-10	4.72528E-09
2297	ENSG00000227834.1			2.608195036	6.65E-10	4.73752E-09
2298	ENSG00000248973.3		107986400	2.004342932	6.68E-10	4.75768E-09
2299	ENSG00000233056.3	ERVH48-1	90625	2.282905169	6.69E-10	4.76287E-09
2300	ENSG00000272211.1			1.359291615	6.75E-10	4.80116E-09
2301	ENSG00000246366.6	LACTB2-AS1	286190	1.331863982	6.77E-10	4.81114E-09
2302	ENSG00000249502.3	MED28-DT	121232375	1.302201941	6.87E-10	4.8795E-09
2303	ENSG00000263551.6			3.431404196	6.88E-10	4.88369E-09
2304	ENSG00000176049.16	JAKMIP2	9832	1.876021921	6.98E-10	4.95216E-09
2305	ENSG00000225578.1	NCBP2-AS1	100874001	1.553039235	7.02E-10	4.97838E-09
2306	ENSG00000275191.1			1.613125443	7.02E-10	4.98156E-09
2307	ENSG00000226526.1			2.388143609	7.06E-10	5.00861E-09
2308	ENSG00000056558.11	TRAF1	7185	1.041730127	7.23E-10	5.12367E-09
2309	ENSG00000276707.1			3.2033548	7.31E-10	5.17596E-09
2310	ENSG00000268108.1			2.213179525	7.33E-10	5.186E-09
2311	ENSG00000245904.4	BTG1-DT		1.214964915	7.37E-10	5.21458E-09
2312	ENSG00000217241.1	CBX3P9		1.198196692	7.47E-10	5.282E-09
2313	ENSG00000241912.1			2.541991109	7.56E-10	5.34245E-09
2314	ENSG00000286802.1		105378842	2.302674442	7.64E-10	5.39005E-09
2315	ENSG00000198729.5	PPP1R14C	81706	1.01266113	7.72E-10	5.44669E-09
2316	ENSG00000221957.8	KIR2DS4	3809	2.642083576	7.79E-10	5.49331E-09
2317	ENSG00000233101.10	HOXB-AS3	404266	2.392167205	7.90E-10	5.56659E-09
2318	ENSG00000249894.1			2.115847684	7.90E-10	5.56862E-09
2319	ENSG00000078399.19	HOXA9	3205	2.147062454	7.94E-10	5.58707E-09
2320	ENSG00000286067.1			1.146844349	7.98E-10	5.61925E-09
2321	ENSG00000162779.22	AXDND1	126859	2.059292275	8.12E-10	5.71678E-09
2322	ENSG00000175197.12	DDIT3	1649	1.112947716	8.17E-10	5.74373E-09
2323	ENSG00000285407.1			2.214666948	8.24E-10	5.78919E-09
2324	ENSG00000271013.1	LRRC37A9P		2.022401975	8.25E-10	5.79318E-09
2325	ENSG00000144583.5	MARCHF4	57574	1.725252419	8.28E-10	5.81797E-09
2326	ENSG00000257900.2			2.190625653	8.31E-10	5.8348E-09
2327	ENSG00000247081.8	BAALC-AS1	105369147	1.071589615	8.33E-10	5.8473E-09
2328	ENSG00000225762.1	LINC01389		1.45698594	8.34E-10	5.85213E-09
2329	ENSG00000163806.16	SPDYA	245711	1.10499277	8.58E-10	6.01343E-09
2330	ENSG00000263412.1	NFE2L1-DT		1.098860648	8.63E-10	6.04408E-09
2331	ENSG00000240032.1	LNCSRLR	109729161	1.518461412	8.71E-10	6.09751E-09
2332	ENSG00000165046.13	LETM2	137994	1.060505768	8.74E-10	6.11844E-09
2333	ENSG00000227088.2			3.148515141	8.74E-10	6.12002E-09
2334	ENSG00000272218.1			3.317094075	8.82E-10	6.17211E-09
2335	ENSG00000061656.10	SPAG4	6676	1.117096552	8.84E-10	6.17865E-09
2336	ENSG00000231802.1	DNAJB6P3		2.934106702	8.85E-10	6.19077E-09
2337	ENSG00000258535.1			2.083322816	8.86E-10	6.19603E-09
2338	ENSG00000272953.1			1.574256459	8.92E-10	6.2345E-09
2339	ENSG00000105991.10	HOXA1	3198	1.032249094	8.96E-10	6.25411E-09
2340	ENSG00000247151.7	CSTF3-DT	338739	2.224322467	9.22E-10	6.42693E-09
2341	ENSG00000267922.1			1.939469887	9.43E-10	6.57103E-09
2342	ENSG00000133863.9	TEX15	56154	3.345769415	9.58E-10	6.67143E-09
2343	ENSG00000287806.1			3.636797889	9.62E-10	6.69414E-09
2344	ENSG00000214897.5	PNMA6E	649238	4.064943731	9.62E-10	6.69847E-09
2345	ENSG00000242082.2	SLC5A4-AS1		2.266162005	9.63E-10	6.69995E-09
2346	ENSG00000279459.1			2.39076669	9.63E-10	6.70177E-09
2347	ENSG00000227076.1			1.623274247	9.77E-10	6.7894E-09
2348	ENSG00000121933.19	TMIGD3	57413	1.118789942	9.82E-10	6.82601E-09
2349	ENSG00000257135.7	ODC1-DT		1.79036503	9.86E-10	6.85269E-09
2350	ENSG00000180785.10	OR51E1	143503	1.095103802	1.00185E-09	6.95782E-09
2351	ENSG00000218690.2	H2AC10P		1.838693976	1.02247E-09	7.09237E-09
2352	ENSG00000232258.6	TMEM114	283953	3.454541898	1.02838E-09	7.13088E-09
2353	ENSG00000106976.21	DNM1	1759	1.112523932	1.02872E-09	7.13197E-09
2354	ENSG00000144214.10	LYG1	129530	1.280917945	1.04574E-09	7.23858E-09
2355	ENSG00000089820.16	ARHGAP4	393	1.239647179	1.05857E-09	7.3225E-09
2356	ENSG00000248405.10	PRR5-ARHGAP8	553158	2.008394613	1.06743E-09	7.38099E-09
2357	ENSG00000272807.1			1.092627818	1.07192E-09	7.41074E-09
2358	ENSG00000171126.8	KCNG3	170850	2.000832928	1.07241E-09	7.41221E-09
2359	ENSG00000287426.1			2.546487824	1.07417E-09	7.42114E-09
2360	ENSG00000238279.1			1.255475662	1.07975E-09	7.45709E-09
2361	ENSG00000233828.5	MIR4280HG	117751736	1.742160436	1.08321E-09	7.47833E-09
2362	ENSG00000115221.12	ITGB6	3694	1.269330915	1.10018E-09	7.59287E-09
2363	ENSG00000103257.9	SLC7A5	8140	1.062180168	1.11468E-09	7.69026E-09
2364	ENSG00000043143.20	JADE2	23338	1.077768796	1.12643E-09	7.76533E-09
2365	ENSG00000207146.1	Y_RNA		2.087379971	1.13188E-09	7.79942E-09
2366	ENSG00000263786.1			1.268721449	1.15279E-09	7.93661E-09
2367	ENSG00000255622.3	PCDHB17P		2.624234705	1.15907E-09	7.97711E-09
2368	ENSG00000272449.2			1.270297323	1.16382E-09	8.00841E-09
2369	ENSG00000150551.11	LYPD1	116372	1.534916868	1.18637E-09	8.15644E-09
2370	ENSG00000251604.1	LINC01385		3.041875206	1.19055E-09	8.18234E-09
2371	ENSG00000287054.1			1.462264739	1.19424E-09	8.20628E-09
2372	ENSG00000120162.10	MOB3B	79817	1.053508822	1.22789E-09	8.42268E-09
2373	ENSG00000229867.2	STEAP3-AS1	100874111	1.547618704	1.22806E-09	8.42268E-09
2374	ENSG00000271930.1			1.674679866	1.24791E-09	8.55582E-09
2375	ENSG00000260476.1			3.433688095	1.2575E-09	8.61415E-09
2376	ENSG00000204682.8	MIR1915HG	399726	1.074349054	1.26109E-09	8.63577E-09
2377	ENSG00000258881.6			1.944952909	1.27319E-09	8.71255E-09
2378	ENSG00000233746.2	LINC00656	200261	2.462252267	1.27418E-09	8.71786E-09
2379	ENSG00000168062.10	BATF2	116071	1.699090264	1.27754E-09	8.7363E-09
2380	ENSG00000228216.1			2.094296401	1.29387E-09	8.8434E-09
2381	ENSG00000227482.1			1.844280748	1.29756E-09	8.86714E-09
2382	ENSG00000221740.1	SNORD93	692210	2.166278587	1.30471E-09	8.91291E-09
2383	ENSG00000214222.2	TUBBP2		1.646964515	1.30786E-09	8.93289E-09
2384	ENSG00000173262.12	SLC2A14	144195	2.026809847	1.30845E-09	8.93537E-09
2385	ENSG00000287815.1			1.748876325	1.31191E-09	8.95439E-09
2386	ENSG00000105825.14	TFPI2	7980	2.189700205	1.31943E-09	9.00105E-09
2387	ENSG00000273449.1			1.27497201	1.37292E-09	9.34989E-09
2388	ENSG00000248238.2	LINC02438		3.687039148	1.38954E-09	9.45654E-09
2389	ENSG00000230647.2			2.657274035	1.38984E-09	9.457E-09
2390	ENSG00000260913.1	LINC01254	101927350	2.684402044	1.40753E-09	9.57244E-09
2391	ENSG00000285875.1			2.319784392	1.42714E-09	9.70079E-09
2392	ENSG00000230257.5	NFE4	58160	3.018483677	1.4372E-09	9.76416E-09
2393	ENSG00000106484.16	MEST	4232	1.046082559	1.44326E-09	9.80028E-09
2394	ENSG00000112837.17	TBX18	9096	1.53638709	1.46465E-09	9.93705E-09
2395	ENSG00000249628.3	LINC00942	100292680	2.06748028	1.46813E-09	9.95889E-09
2396	ENSG00000139629.16	GALNT6	11226	1.327979204	1.48011E-09	1.0035E-08
2397	ENSG00000204381.12	LAYN	143903	1.148958715	1.51565E-09	1.02655E-08
2398	ENSG00000166928.11	MS4A14	84689	1.108322557	1.54012E-09	1.04241E-08
2399	ENSG00000270933.1			1.663770915	1.54391E-09	1.0448E-08
2400	ENSG00000283421.1			2.568714771	1.54665E-09	1.04638E-08
2401	ENSG00000262136.1	NQO1-DT	119139903	1.499156028	1.54704E-09	1.04638E-08
2402	ENSG00000225693.1	LAGE3P1		1.847375188	1.55599E-09	1.05225E-08
2403	ENSG00000196139.14	AKR1C3	8644	1.956221584	1.59433E-09	1.07708E-08
2404	ENSG00000263834.1	MIR4635	100616479	2.181977049	1.61527E-09	1.09085E-08
2405	ENSG00000179083.6	FAM133A	286499	3.540206754	1.63293E-09	1.10203E-08
2406	ENSG00000229961.3			2.304684124	1.64863E-09	1.11241E-08
2407	ENSG00000285887.1			2.450877035	1.6684E-09	1.12492E-08
2408	ENSG00000273340.1	MICE		1.254295839	1.70683E-09	1.14975E-08
2409	ENSG00000258592.1	LINC03059		1.753700579	1.71412E-09	1.15407E-08
2410	ENSG00000118160.14	SLC8A2	6543	2.052726266	1.72895E-09	1.16366E-08
2411	ENSG00000204278.12	TMEM235	283999	3.543189089	1.74225E-09	1.17201E-08
2412	ENSG00000276166.1			1.907829217	1.75212E-09	1.17788E-08
2413	ENSG00000103449.12	SALL1	6299	1.880356991	1.75216E-09	1.17788E-08
2414	ENSG00000231532.6	LINC01249	727982	4.578122427	1.76762E-09	1.18787E-08
2415	ENSG00000142700.12	DMRTA2	63950	2.921543991	1.8122E-09	1.2168E-08
2416	ENSG00000142910.16	TINAGL1	64129	1.182564908	1.81614E-09	1.21903E-08
2417	ENSG00000163810.12	TGM4	7047	1.368678046	1.82304E-09	1.22346E-08
2418	ENSG00000245008.4		101929538	1.700147893	1.83293E-09	1.22989E-08
2419	ENSG00000223695.1	MYH9-DT		1.369776027	1.83787E-09	1.23278E-08
2420	ENSG00000108417.4	KRT37	8688	2.310425399	1.85439E-09	1.24323E-08
2421	ENSG00000164694.17	FNDC1	84624	1.720034951	1.87775E-09	1.25826E-08
2422	ENSG00000162341.18	TPCN2	219931	1.044271237	1.88484E-09	1.26258E-08
2423	ENSG00000132000.13	PODNL1	79883	1.151666068	1.90364E-09	1.27453E-08
2424	ENSG00000265728.1			2.556294512	1.91287E-09	1.28005E-08
2425	ENSG00000070748.19	CHAT	1103	2.891043124	1.9133E-09	1.28013E-08
2426	ENSG00000163464.8	CXCR1	3577	1.753774194	1.93849E-09	1.29633E-08
2427	ENSG00000186867.11	QRFPR	84109	3.115854015	1.94822E-09	1.3024E-08
2428	ENSG00000242258.1	LINC00996	285972	1.395429313	1.95134E-09	1.30426E-08
2429	ENSG00000271218.1			1.389494156	1.99675E-09	1.33416E-08
2430	ENSG00000229816.1	DDX50P1		1.087040326	2.0016E-09	1.33718E-08
2431	ENSG00000203588.3	IGBP1-AS1		1.480998382	2.00769E-09	1.34079E-08
2432	ENSG00000139572.4	GPR84	53831	1.141258211	2.0176E-09	1.3465E-08
2433	ENSG00000146006.8	LRRTM2	26045	1.430327101	2.02506E-09	1.35103E-08
2434	ENSG00000259701.1			2.001692596	2.02889E-09	1.35336E-08
2435	ENSG00000236700.6	LINC01010	154092	1.407393065	2.05919E-09	1.37287E-08
2436	ENSG00000250657.1			4.187670324	2.07573E-09	1.38367E-08
2437	ENSG00000253408.6	IKBKB-DT	101929897	1.097055251	2.09034E-09	1.39294E-08
2438	ENSG00000237686.7	SCIRT	101929705	1.513894216	2.12279E-09	1.41385E-08
2439	ENSG00000287306.1			1.528516257	2.12971E-09	1.41822E-08
2440	ENSG00000139714.12	MORN3	283385	1.608787748	2.13274E-09	1.41994E-08
2441	ENSG00000177710.6	SLC35G5	83650	2.310668595	2.133E-09	1.41994E-08
2442	ENSG00000286940.1	MICB-DT	102725068	1.22357898	2.13936E-09	1.42381E-08
2443	ENSG00000273232.1			2.2111769	2.15385E-09	1.43309E-08
2444	ENSG00000261379.1			2.542770978	2.16433E-09	1.43983E-08
2445	ENSG00000231528.3	FAM225A	286333	1.236485776	2.20384E-09	1.4639E-08
2446	ENSG00000266105.1			4.075449683	2.20459E-09	1.46415E-08
2447	ENSG00000236501.6			1.689427184	2.21096E-09	1.46813E-08
2448	ENSG00000251593.1	MSNP1		1.282311541	2.21847E-09	1.47263E-08
2449	ENSG00000240399.1			1.281432065	2.27532E-09	1.50844E-08
2450	ENSG00000248599.2	SLEAR	441374	1.727806066	2.27547E-09	1.50844E-08
2451	ENSG00000215252.11	GOLGA8B	23015	1.229720201	2.31441E-09	1.53247E-08
2452	ENSG00000148704.13	VAX1	11023	2.344274549	2.32176E-09	1.53661E-08
2453	ENSG00000236591.1	UST-AS2		1.958479292	2.35084E-09	1.55503E-08
2454	ENSG00000196593.9	ANKRD20A19P		3.232942073	2.3887E-09	1.57797E-08
2455	ENSG00000220702.1			2.853967856	2.39768E-09	1.58337E-08
2456	ENSG00000235207.1	TUBBP6		2.50063699	2.41922E-09	1.59653E-08
2457	ENSG00000188660.4	LINC00319	102724398	2.018579561	2.43247E-09	1.60474E-08
2458	ENSG00000248388.5	HAFML	105377555	2.46647648	2.44893E-09	1.61506E-08
2459	ENSG00000286913.1			1.828719464	2.45172E-09	1.61637E-08
2460	ENSG00000225882.1	LINC01456	105373144	4.344586498	2.52375E-09	1.66109E-08
2461	ENSG00000286069.2		105378250	2.536190078	2.54172E-09	1.67208E-08
2462	ENSG00000225077.3	ICMT-DT	148645	1.100711532	2.56239E-09	1.68414E-08
2463	ENSG00000186038.9	HTR3E	285242	2.726190837	2.57072E-09	1.68892E-08
2464	ENSG00000213397.10	HAUS7	55559	1.158719942	2.57395E-09	1.69076E-08
2465	ENSG00000280604.1			1.84315149	2.59682E-09	1.70522E-08
2466	ENSG00000258949.1			2.628323031	2.60132E-09	1.70789E-08
2467	ENSG00000177699.4		100129540	1.812713094	2.60824E-09	1.71215E-08
2468	ENSG00000286021.2	LINC02822		3.853581402	2.72498E-09	1.78612E-08
2469	ENSG00000111537.5	IFNG	3458	1.852343734	2.75956E-09	1.8079E-08
2470	ENSG00000241104.5	CEACAMP10		2.532904209	2.76876E-09	1.81332E-08
2471	ENSG00000272037.1			1.192603265	2.77142E-09	1.81448E-08
2472	ENSG00000155465.20	SLC7A7	9056	1.064966471	2.77145E-09	1.81448E-08
2473	ENSG00000284540.1			2.553845911	2.78022E-09	1.81992E-08
2474	ENSG00000281756.1	C2-AS1	102060414	2.375072295	2.78529E-09	1.82264E-08
2475	ENSG00000267201.1	LINC01775	101928602	1.357904372	2.80047E-09	1.83197E-08
2476	ENSG00000235304.2	LINC01281	286442	1.760679587	2.81494E-09	1.84114E-08
2477	ENSG00000241106.8	HLA-DOB	3112	1.069316308	2.81955E-09	1.84384E-08
2478	ENSG00000262903.1	CTNS-AS1		1.198320317	2.91504E-09	1.90409E-08
2479	ENSG00000269997.1			1.477819218	2.92852E-09	1.91195E-08
2480	ENSG00000167619.13	TMEM145	284339	1.619511808	2.93635E-09	1.91675E-08
2481	ENSG00000240922.1	LSAMP-AS1	101926903	2.690111819	2.96256E-09	1.93227E-08
2482	ENSG00000231752.6	EMBP1		1.372945054	2.98907E-09	1.94924E-08
2483	ENSG00000198573.7	SPANXC	64663	4.680486871	3.03447E-09	1.97754E-08
2484	ENSG00000230392.1	LINC03098	101928336	2.95326541	3.06544E-09	1.99609E-08
2485	ENSG00000204876.4			2.158523486	3.11624E-09	2.02783E-08
2486	ENSG00000254057.1			2.446553492	3.13159E-09	2.03709E-08
2487	ENSG00000107984.10	DKK1	22943	1.833519613	3.13607E-09	2.03906E-08
2488	ENSG00000255537.1		403312	2.054846818	3.16937E-09	2.0597E-08
2489	ENSG00000287735.1			1.259158993	3.17427E-09	2.06255E-08
2490	ENSG00000108511.10	HOXB6	3216	1.586536683	3.18087E-09	2.0665E-08
2491	ENSG00000112195.9	TREML2	79865	1.479199319	3.18509E-09	2.0689E-08
2492	ENSG00000286642.1			2.940379148	3.20547E-09	2.08146E-08
2493	ENSG00000185955.5	SPACDR	402573	1.358465313	3.2136E-09	2.08605E-08
2494	ENSG00000279789.1			1.059625561	3.22532E-09	2.09332E-08
2495	ENSG00000226706.1		101928525	1.84379602	3.25779E-09	2.11336E-08
2496	ENSG00000287031.1			2.124372109	3.2821E-09	2.12843E-08
2497	ENSG00000166897.16	ELFN2	114794	1.996148915	3.30826E-09	2.14434E-08
2498	ENSG00000160856.21	FCRL3	115352	1.722294526	3.3326E-09	2.15976E-08
2499	ENSG00000228376.3	GAS2L1P2		2.371705485	3.35956E-09	2.17582E-08
2500	ENSG00000253554.7	LINC01414	102724623	3.896112607	3.36056E-09	2.17611E-08
2501	ENSG00000255558.1			2.769318164	3.39224E-09	2.19554E-08
2502	ENSG00000248597.2			1.857891395	3.45977E-09	2.23633E-08
2503	ENSG00000248773.1			1.82316639	3.46358E-09	2.23843E-08
2504	ENSG00000256443.1			1.471445166	3.48406E-09	2.25056E-08
2505	ENSG00000121297.8	TSHZ3	57616	1.004923445	3.49408E-09	2.25667E-08
2506	ENSG00000283627.1			3.239729293	3.5023E-09	2.26087E-08
2507	ENSG00000258128.2	MKRN9P		3.008434887	3.54915E-09	2.29E-08
2508	ENSG00000175728.4	LINC02873	283171	1.984978242	3.54992E-09	2.29012E-08
2509	ENSG00000269984.1			1.230679083	3.57151E-09	2.3033E-08
2510	ENSG00000260145.1			1.92463862	3.68525E-09	2.37296E-08
2511	ENSG00000258183.6	LINC02392		2.776878272	3.68809E-09	2.37423E-08
2512	ENSG00000146674.15	IGFBP3	3486	1.290414293	3.70674E-09	2.38508E-08
2513	ENSG00000266651.1			1.242605739	3.72451E-09	2.39574E-08
2514	ENSG00000230033.1			2.985005873	3.72582E-09	2.39619E-08
2515	ENSG00000253658.6	LINC01592	100505718	2.718831669	3.76534E-09	2.42082E-08
2516	ENSG00000232725.1	PLXNB3-AS1		1.161509482	3.77446E-09	2.42629E-08
2517	ENSG00000167281.19	RBFOX3	146713	1.880085124	3.81622E-09	2.45194E-08
2518	ENSG00000224899.1	LINC02830		3.406411159	3.81916E-09	2.45343E-08
2519	ENSG00000260186.5	LINC02137		1.669740148	3.82728E-09	2.45785E-08
2520	ENSG00000214184.3	GCC2-AS1	644903	1.080549485	3.83876E-09	2.46443E-08
2521	ENSG00000278341.1			1.194245939	3.84543E-09	2.46791E-08
2522	ENSG00000187045.19	TMPRSS6	164656	1.241857876	3.84769E-09	2.46896E-08
2523	ENSG00000259408.2			1.310469822	3.91708E-09	2.51226E-08
2524	ENSG00000065618.21	COL17A1	1308	1.339538483	3.92551E-09	2.51726E-08
2525	ENSG00000269053.1			1.391612433	3.93152E-09	2.52071E-08
2526	ENSG00000269929.3	MIRLET7A1HG	112903833	1.085650776	3.93302E-09	2.52127E-08
2527	ENSG00000125462.19	MIR9-1HG	10485	1.743799503	3.94063E-09	2.52546E-08
2528	ENSG00000114473.14	IQCG	84223	1.239705389	3.94084E-09	2.52546E-08
2529	ENSG00000157399.16	ARSL	415	1.421337662	3.96252E-09	2.53812E-08
2530	ENSG00000258904.1			1.470918752	4.02627E-09	2.57563E-08
2531	ENSG00000241475.1			2.163988918	4.07105E-09	2.60344E-08
2532	ENSG00000265917.1	MIR3685	100500802	1.39242281	4.08218E-09	2.60971E-08
2533	ENSG00000184954.4	OR6C70	390327	3.891243969	4.16744E-09	2.66164E-08
2534	ENSG00000271156.1			3.088511648	4.16937E-09	2.66245E-08
2535	ENSG00000213658.12	LAT	27040	1.317968993	4.22098E-09	2.6941E-08
2536	ENSG00000250038.7			3.811587067	4.22677E-09	2.69736E-08
2537	ENSG00000197753.11	LHFPL5	222662	1.019486751	4.34163E-09	2.76932E-08
2538	ENSG00000259727.1			2.081480703	4.3516E-09	2.77492E-08
2539	ENSG00000254369.6	HOXA-AS3	100133311	1.755314769	4.35278E-09	2.7751E-08
2540	ENSG00000287022.1			2.564575611	4.36402E-09	2.78137E-08
2541	ENSG00000236961.1			2.937781708	4.43068E-09	2.82159E-08
2542	ENSG00000152503.10	TRIM36	55521	1.010746741	4.44915E-09	2.83198E-08
2543	ENSG00000205846.4	CLEC6A	93978	2.022847013	4.49528E-09	2.85951E-08
2544	ENSG00000286546.1		613266	2.970004465	4.61805E-09	2.93384E-08
2545	ENSG00000039987.7	BEST2	54831	2.26434918	4.64459E-09	2.95023E-08
2546	ENSG00000262152.7	GREP1	283875	1.91612885	4.68743E-09	2.97506E-08
2547	ENSG00000250708.2	LINC02269	101928478	3.579502101	4.7008E-09	2.98259E-08
2548	ENSG00000196335.13	STK31	56164	1.515963302	4.71909E-09	2.99371E-08
2549	ENSG00000244482.10	LILRA6	79168	1.178255661	4.7311E-09	3.00037E-08
2550	ENSG00000277693.2			4.197953908	4.73402E-09	3.00175E-08
2551	ENSG00000112761.21	CCN6	8838	2.123469612	4.73514E-09	3.00197E-08
2552	ENSG00000170858.10	LILRP2	79166	1.722311919	4.79318E-09	3.03634E-08
2553	ENSG00000275379.2	H3C11	8354	2.121129329	4.82564E-09	3.05593E-08
2554	ENSG00000233708.1			2.467458074	4.95042E-09	3.13095E-08
2555	ENSG00000229021.2			1.397950373	4.98901E-09	3.15435E-08
2556	ENSG00000109193.12	SULT1E1	6783	2.254961166	4.99553E-09	3.15797E-08
2557	ENSG00000061918.14	GUCY1B1	2983	1.036380387	4.99894E-09	3.15911E-08
2558	ENSG00000225855.7	RUSC1-AS1	284618	1.02746321	5.01402E-09	3.16814E-08
2559	ENSG00000198917.13	SPOUT1	51490	1.664953164	5.06043E-09	3.19645E-08
2560	ENSG00000224321.1	RPL12P14		1.213781557	5.07265E-09	3.20314E-08
2561	ENSG00000232884.9		105372738	1.34702885	5.13614E-09	3.24066E-08
2562	ENSG00000279926.1			1.342132776	5.30698E-09	3.3426E-08
2563	ENSG00000070087.14	PFN2	5217	1.101324733	5.34161E-09	3.36387E-08
2564	ENSG00000280063.1			1.111621808	5.39487E-09	3.39472E-08
2565	ENSG00000164112.13	SMIM43	132332	1.591620532	5.41138E-09	3.40457E-08
2566	ENSG00000231107.2	LINC01508		2.26027419	5.4203E-09	3.40933E-08
2567	ENSG00000259349.2	APPBP2-DT		1.423869277	5.42067E-09	3.40933E-08
2568	ENSG00000204661.9	SPATA31J1		2.011484193	5.429E-09	3.41403E-08
2569	ENSG00000182183.15	SHISAL2A	348378	1.3921601	5.64448E-09	3.54335E-08
2570	ENSG00000198846.6	TOX	9760	1.217378331	5.6678E-09	3.55686E-08
2571	ENSG00000230876.8	LINC00486	285045	3.01140449	5.68263E-09	3.56447E-08
2572	ENSG00000224003.1	YES1P1		1.3894767	5.68456E-09	3.56512E-08
2573	ENSG00000113600.11	C9	735	1.689838924	5.69997E-09	3.57366E-08
2574	ENSG00000231648.2	LINC01698		1.372138359	5.70179E-09	3.57423E-08
2575	ENSG00000232453.7	LINC02777	105378753	1.268985625	5.71875E-09	3.5843E-08
2576	ENSG00000105419.18	MEIS3	56917	1.159712484	5.77872E-09	3.62074E-08
2577	ENSG00000230088.1	KRT16P5		1.811938597	5.8372E-09	3.6568E-08
2578	ENSG00000188056.12	TREML4	285852	2.497618918	5.86935E-09	3.67462E-08
2579	ENSG00000278954.1			1.330057147	6.00174E-09	3.75158E-08
2580	ENSG00000263893.2		105371887	1.46975478	6.00704E-09	3.75431E-08
2581	ENSG00000211892.4	IGHG4		2.213216973	6.10077E-09	3.80988E-08
2582	ENSG00000230798.5	FOXD3-AS1	100996301	1.7008729	6.15243E-09	3.84093E-08
2583	ENSG00000254854.1	NECTIN1-DT		1.038810667	6.19099E-09	3.86318E-08
2584	ENSG00000216490.4	IFI30	10437	1.237221559	6.25381E-09	3.89985E-08
2585	ENSG00000182218.9	HHIPL1	84439	1.174497928	6.27082E-09	3.90869E-08
2586	ENSG00000213706.2	RHOG2P		2.153629219	6.27499E-09	3.91067E-08
2587	ENSG00000279716.1			1.182510624	6.31785E-09	3.93614E-08
2588	ENSG00000244349.1			1.250826102	6.3682E-09	3.96502E-08
2589	ENSG00000259786.7	LINC02109		3.312549946	6.42593E-09	4.00034E-08
2590	ENSG00000197506.8	SLC28A3	64078	1.267167811	6.44559E-09	4.01069E-08
2591	ENSG00000255346.10	NOX5	79400	1.44943102	6.48675E-09	4.03504E-08
2592	ENSG00000232458.1	LINC01450	101928744	2.59290924	6.50037E-09	4.04267E-08
2593	ENSG00000254287.1			2.878396144	6.50106E-09	4.04267E-08
2594	ENSG00000146555.19	SDK1	221935	1.209093157	6.58967E-09	4.09392E-08
2595	ENSG00000260456.7	C16orf95	100506581	1.058109419	6.70752E-09	4.16322E-08
2596	ENSG00000254991.1			1.902921506	6.75052E-09	4.18794E-08
2597	ENSG00000253831.3	ETV3L	440695	2.094863486	6.78377E-09	4.20725E-08
2598	ENSG00000286959.1			3.591910227	6.83446E-09	4.23803E-08
2599	ENSG00000268686.2		101928295	2.060779423	6.85847E-09	4.25159E-08
2600	ENSG00000205045.11	SLFN12L	100506736	1.08949146	6.98748E-09	4.32615E-08
2601	ENSG00000116996.9	ZP4	57829	2.926660178	7.14008E-09	4.41649E-08
2602	ENSG00000117228.10	GBP1	2633	1.26174162	7.14935E-09	4.42153E-08
2603	ENSG00000274372.5			1.40749601	7.25554E-09	4.48293E-08
2604	ENSG00000154864.13	PIEZO2	63895	1.311567605	7.25654E-09	4.48293E-08
2605	ENSG00000182685.7	BRICD5	283870	1.208883678	7.30289E-09	4.51016E-08
2606	ENSG00000171483.13	SSX6P		3.505293207	7.32728E-09	4.52452E-08
2607	ENSG00000172139.15	SLC9C1	285335	1.31523986	7.3718E-09	4.54898E-08
2608	ENSG00000280335.1			1.984209556	7.50136E-09	4.62193E-08
2609	ENSG00000177688.7	SUMO4	387082	1.262441808	7.54816E-09	4.6486E-08
2610	ENSG00000175877.4	TMEM270	135886	2.305171412	7.61441E-09	4.68649E-08
2611	ENSG00000219700.1	PTCHD3P3		2.502788831	7.77796E-09	4.78196E-08
2612	ENSG00000132932.17	ATP8A2	51761	1.384548039	7.83173E-09	4.81203E-08
2613	ENSG00000254639.1			2.130769608	7.89001E-09	4.84483E-08
2614	ENSG00000162039.17	MEIOB	254528	2.3797388	8.08485E-09	4.95862E-08
2615	ENSG00000271550.1	BNIP3P11		1.03825963	8.10588E-09	4.96893E-08
2616	ENSG00000224017.2	LINC01449	101928773	2.113975391	8.38032E-09	5.12844E-08
2617	ENSG00000138080.14	EMILIN1	11117	1.2803494	8.43179E-09	5.15834E-08
2618	ENSG00000275576.1			1.716555037	8.46093E-09	5.17537E-08
2619	ENSG00000263934.5	SNORD3A		1.606122452	8.4984E-09	5.19669E-08
2620	ENSG00000187912.12	CLEC17A	388512	1.718033394	8.51101E-09	5.20359E-08
2621	ENSG00000170231.16	FABP6	2172	1.658021342	8.62582E-09	5.27054E-08
2622	ENSG00000256209.2			2.100123496	8.66206E-09	5.29105E-08
2623	ENSG00000225489.7			1.509167577	8.74239E-09	5.33765E-08
2624	ENSG00000274461.2	LINC02930	105378516	1.793433622	8.79508E-09	5.36734E-08
2625	ENSG00000100298.16	APOBEC3H	164668	1.216119452	9.02872E-09	5.50081E-08
2626	ENSG00000218281.1	H2AC9P		2.519998365	9.02905E-09	5.50081E-08
2627	ENSG00000169429.11	CXCL8	3576	1.655712439	9.15304E-09	5.57464E-08
2628	ENSG00000284523.2		105375421	1.472646042	9.38676E-09	5.71435E-08
2629	ENSG00000288096.1			1.117013096	9.41479E-09	5.73054E-08
2630	ENSG00000287781.1			2.172522824	9.50447E-09	5.77979E-08
2631	ENSG00000279032.1			2.486282453	9.51705E-09	5.78656E-08
2632	ENSG00000273388.1			2.470246072	9.53329E-09	5.79554E-08
2633	ENSG00000285769.2			2.531039272	9.55299E-09	5.80574E-08
2634	ENSG00000231971.6	CT69		1.648578	9.57446E-09	5.817E-08
2635	ENSG00000253686.2	LINC01484	101928136	1.781395306	9.5835E-09	5.8216E-08
2636	ENSG00000277050.1			1.216226029	9.60764E-09	5.83537E-08
2637	ENSG00000255438.2			1.683971889	9.6742E-09	5.8749E-08
2638	ENSG00000126259.20	KIRREL2	84063	2.004693221	9.76837E-09	5.92914E-08
2639	ENSG00000225163.4			1.701527559	9.7695E-09	5.92914E-08
2640	ENSG00000131096.10	PYY	5697	1.313827795	9.90929E-09	6.00754E-08
2641	ENSG00000187134.14	AKR1C1	1645	1.992339741	9.96921E-09	6.04295E-08
2642	ENSG00000255559.1	ZNF252P-AS1	286103	1.296042123	9.97545E-09	6.04581E-08
2643	ENSG00000235703.6	EOLA2-DT		1.076017581	1.00452E-08	6.08621E-08
2644	ENSG00000198759.12	EGFL6	25975	1.148474933	1.00507E-08	6.08768E-08
2645	ENSG00000259792.1			1.147083644	1.01289E-08	6.1332E-08
2646	ENSG00000069812.11	HES2	54626	1.101203596	1.01337E-08	6.13516E-08
2647	ENSG00000260997.1			1.319364367	1.02038E-08	6.17664E-08
2648	ENSG00000143851.15	PTPN7	5778	1.154904948	1.02435E-08	6.19737E-08
2649	ENSG00000273008.1	CSGALNACT2-DT	118568825	1.005076179	1.03484E-08	6.25559E-08
2650	ENSG00000287277.1		105376387	1.568523884	1.03667E-08	6.26476E-08
2651	ENSG00000180644.8	PRF1	5551	1.240562484	1.04284E-08	6.30013E-08
2652	ENSG00000179331.3	RAB39A	54734	1.494212996	1.04936E-08	6.33661E-08
2653	ENSG00000286507.1			2.100304971	1.05498E-08	6.36953E-08
2654	ENSG00000269534.5			1.837766123	1.06064E-08	6.40084E-08
2655	ENSG00000275632.1			1.253563346	1.06445E-08	6.42182E-08
2656	ENSG00000260022.1			1.085358644	1.07916E-08	6.50467E-08
2657	ENSG00000237017.1	PRPF31-AS1		1.188422301	1.08776E-08	6.55549E-08
2658	ENSG00000151631.8	AKR1C6P		2.491563181	1.10035E-08	6.62736E-08
2659	ENSG00000230499.1			1.698662376	1.10069E-08	6.62836E-08
2660	ENSG00000287097.1		107986639	2.444107353	1.11715E-08	6.72241E-08
2661	ENSG00000267279.2			1.579841712	1.12793E-08	6.78211E-08
2662	ENSG00000287697.1		105371622	1.725677569	1.13842E-08	6.84209E-08
2663	ENSG00000116035.4	VAX2	25806	1.53826697	1.1387E-08	6.84272E-08
2664	ENSG00000170801.10	HTRA3	94031	1.285241112	1.18165E-08	7.09225E-08
2665	ENSG00000096996.16	IL12RB1	3594	1.203160779	1.18189E-08	7.09256E-08
2666	ENSG00000226985.7	LINC01203	100133123	1.617648202	1.19074E-08	7.14355E-08
2667	ENSG00000240350.3	SOCAR		1.373190607	1.19167E-08	7.14804E-08
2668	ENSG00000272328.1			2.460109944	1.19345E-08	7.15763E-08
2669	ENSG00000231605.6	LINC01363	101928484	2.602546968	1.19963E-08	7.19359E-08
2670	ENSG00000185507.21	IRF7	3665	1.01418053	1.21239E-08	7.26354E-08
2671	ENSG00000269994.3	LINC02893	440173	1.900779397	1.23993E-08	7.42292E-08
2672	ENSG00000123201.14	GUCY1B2		1.633194258	1.24514E-08	7.45185E-08
2673	ENSG00000285881.1			1.632104603	1.25828E-08	7.52823E-08
2674	ENSG00000266967.7	AARSD1	80755	1.028719351	1.26107E-08	7.54379E-08
2675	ENSG00000199572.1	RNA5SP174		3.042528842	1.29919E-08	7.76011E-08
2676	ENSG00000197599.12	CCDC154	645811	1.637951714	1.30165E-08	7.77363E-08
2677	ENSG00000164176.13	EDIL3	10085	1.320373779	1.3042E-08	7.78767E-08
2678	ENSG00000180113.16	TDRD6	221400	1.061509365	1.30916E-08	7.81496E-08
2679	ENSG00000267199.1			1.37755801	1.31013E-08	7.81957E-08
2680	ENSG00000225107.1			3.861261351	1.32536E-08	7.90451E-08
2681	ENSG00000272100.1			2.01345534	1.33591E-08	7.96267E-08
2682	ENSG00000270343.1	UNGP3		1.210229782	1.35369E-08	8.06498E-08
2683	ENSG00000226004.1	SIMALR		2.460699015	1.35893E-08	8.09499E-08
2684	ENSG00000249642.2		105377603	4.353957173	1.36245E-08	8.11232E-08
2685	ENSG00000212961.4	HNRNPA1P40		2.043273768	1.37493E-08	8.18169E-08
2686	ENSG00000188060.7	RAB42	115273	1.273814757	1.37646E-08	8.18953E-08
2687	ENSG00000275022.1	MIR6753	102465451	1.913084809	1.37666E-08	8.18953E-08
2688	ENSG00000270562.1	FOXP1-DT		1.55081742	1.37897E-08	8.20206E-08
2689	ENSG00000233611.3	GBX2-AS1	121853074	2.377238393	1.38478E-08	8.23536E-08
2690	ENSG00000258657.5	GZMH-AS1		1.63275072	1.38541E-08	8.23787E-08
2691	ENSG00000235651.1	G3BP1P1		1.688416671	1.38804E-08	8.25226E-08
2692	ENSG00000264015.2			2.987315133	1.40132E-08	8.32749E-08
2693	ENSG00000228499.1	TMSB10P1		1.42242983	1.40388E-08	8.34017E-08
2694	ENSG00000287987.1			2.47064322	1.40816E-08	8.36314E-08
2695	ENSG00000228028.2			2.035934475	1.42552E-08	8.46241E-08
2696	ENSG00000267554.1			1.101060919	1.43062E-08	8.49016E-08
2697	ENSG00000239713.9	APOBEC3G	60489	1.044731511	1.43412E-08	8.50961E-08
2698	ENSG00000158427.15	TMSB15B	286527	1.412616676	1.43816E-08	8.52978E-08
2699	ENSG00000267452.3	LINC02073	440446	1.570904554	1.4393E-08	8.53397E-08
2700	ENSG00000274150.1	FADS2B		2.996201766	1.46648E-08	8.68672E-08
2701	ENSG00000152495.11	CAMK4	814	1.061540053	1.46788E-08	8.69307E-08
2702	ENSG00000267736.2	HMGB2P1		2.198546967	1.47524E-08	8.73533E-08
2703	ENSG00000261949.6	GFY	100507003	2.645916412	1.49365E-08	8.83906E-08
2704	ENSG00000227542.1	MYO1B-AS1	122152331	1.010092551	1.5127E-08	8.94512E-08
2705	ENSG00000158786.5	PLA2G2F	64600	2.034107778	1.52213E-08	8.99553E-08
2706	ENSG00000128253.15	RFPL2	10739	1.950723812	1.52372E-08	9.00359E-08
2707	ENSG00000227579.6			2.418683723	1.53565E-08	9.06865E-08
2708	ENSG00000253837.1	LOXL2-AS1	100507156	1.309456677	1.58525E-08	9.3435E-08
2709	ENSG00000236713.1			2.603328799	1.58686E-08	9.3516E-08
2710	ENSG00000214617.9	SLC6A10P		2.808808105	1.59594E-08	9.40178E-08
2711	ENSG00000237290.2	LINC01343	339442	2.338215271	1.62111E-08	9.54063E-08
2712	ENSG00000253363.7		124906685	3.689920121	1.64889E-08	9.69695E-08
2713	ENSG00000095059.16	DHPS	1725	1.129723097	1.65394E-08	9.72087E-08
2714	ENSG00000287134.1			1.488166102	1.65931E-08	9.75101E-08
2715	ENSG00000250302.3	LINC01618	107986281	1.024351243	1.67597E-08	9.84453E-08
2716	ENSG00000177173.5	NAP1L4P1		1.001780221	1.68296E-08	9.88413E-08
2717	ENSG00000171872.5	KLF17	128209	2.239053389	1.69171E-08	9.93258E-08
2718	ENSG00000273350.1			1.351145022	1.70234E-08	9.992E-08
2719	ENSG00000162782.16	TDRD5	163589	2.32808766	1.70431E-08	1.00021E-07
2720	ENSG00000174343.6	CHRNA9	55584	1.977839668	1.71631E-08	1.0068E-07
2721	ENSG00000239605.11	STPG4	285051	2.485827087	1.73379E-08	1.01635E-07
2722	ENSG00000136535.15	TBR1	10716	3.158269879	1.75205E-08	1.02625E-07
2723	ENSG00000284994.1			2.03211535	1.7614E-08	1.03142E-07
2724	ENSG00000259831.1	LINC00567	283486	1.990548302	1.77976E-08	1.04156E-07
2725	ENSG00000244932.2	IFT122P2		1.094284427	1.81062E-08	1.05853E-07
2726	ENSG00000262445.3			2.338500313	1.86241E-08	1.08704E-07
2727	ENSG00000180318.4	ALX1	8092	2.35327798	1.90964E-08	1.11346E-07
2728	ENSG00000165521.15	EML5	161436	1.6030295	1.91148E-08	1.11437E-07
2729	ENSG00000224082.1	UBTFL8		3.229682955	1.93779E-08	1.12888E-07
2730	ENSG00000250979.1	TRPA2P		2.04335348	1.94278E-08	1.13162E-07
2731	ENSG00000262979.1			1.285458418	1.97206E-08	1.14817E-07
2732	ENSG00000189430.13	NCR1	9437	1.452855465	2.00434E-08	1.16628E-07
2733	ENSG00000205628.4	LINC01446	401337	3.845900497	2.05998E-08	1.1976E-07
2734	ENSG00000287195.1			1.863525713	2.07394E-08	1.2048E-07
2735	ENSG00000234945.8	GTF3C2-AS1	100505624	1.600958122	2.07419E-08	1.2048E-07
2736	ENSG00000260177.1			2.060860474	2.07579E-08	1.20537E-07
2737	ENSG00000249375.8	CASC11	100270680	1.159098897	2.08716E-08	1.21153E-07
2738	ENSG00000226053.1	LINC01776	729987	3.554383116	2.10013E-08	1.21862E-07
2739	ENSG00000237265.6			1.957035805	2.11184E-08	1.22505E-07
2740	ENSG00000227492.2	CHUK-DT		1.095716085	2.13324E-08	1.23692E-07
2741	ENSG00000118004.18	COLEC11	78989	1.597218786	2.16486E-08	1.25452E-07
2742	ENSG00000271969.1			2.157550758	2.19033E-08	1.26854E-07
2743	ENSG00000187474.5	FPR3	2359	1.101992123	2.20943E-08	1.27867E-07
2744	ENSG00000248131.6	LINC01194	404663	5.344919957	2.22415E-08	1.28662E-07
2745	ENSG00000222012.1		105375614	1.814058148	2.22593E-08	1.28747E-07
2746	ENSG00000227214.2	HCG15		1.092184695	2.23357E-08	1.2917E-07
2747	ENSG00000274528.1			1.066005782	2.23534E-08	1.29253E-07
2748	ENSG00000128262.8	POM121L9P		1.191707141	2.24336E-08	1.2966E-07
2749	ENSG00000269653.1			1.452925518	2.25016E-08	1.30035E-07
2750	ENSG00000214888.3	NPAP1P9		3.138438709	2.27672E-08	1.31491E-07
2751	ENSG00000271833.1			1.052606799	2.27702E-08	1.31491E-07
2752	ENSG00000260810.1			1.177075666	2.28529E-08	1.31892E-07
2753	ENSG00000185433.10	LINC00158	54072	2.082845621	2.28865E-08	1.32066E-07
2754	ENSG00000272551.1			1.962105458	2.348E-08	1.35353E-07
2755	ENSG00000253496.3			2.360324041	2.36096E-08	1.36061E-07
2756	ENSG00000272374.1	SCUBE3-AS1		1.158764213	2.36218E-08	1.36111E-07
2757	ENSG00000225087.2		105378798	2.532943353	2.3704E-08	1.36565E-07
2758	ENSG00000205702.11	CYP2D7	105377203	1.120076011	2.37304E-08	1.36697E-07
2759	ENSG00000100288.20	CHKB	1120	1.127310628	2.37393E-08	1.36729E-07
2760	ENSG00000198133.8	TMEM229B	161145	1.188605694	2.38966E-08	1.37575E-07
2761	ENSG00000269846.1			1.876497817	2.39421E-08	1.37797E-07
2762	ENSG00000261360.1	OTULIN-DT	728178	1.027757211	2.39603E-08	1.37861E-07
2763	ENSG00000268785.1	RPL7P50		1.565733017	2.40495E-08	1.38354E-07
2764	ENSG00000164484.12	TMEM200A	114801	1.297719148	2.40663E-08	1.38431E-07
2765	ENSG00000286587.1			1.841246666	2.41783E-08	1.39035E-07
2766	ENSG00000273989.1			1.693097265	2.42661E-08	1.3952E-07
2767	ENSG00000265206.5	MIR142HG	123466215	1.512061564	2.45189E-08	1.40932E-07
2768	ENSG00000271924.1	RNA5SP108		1.940239848	2.48961E-08	1.42934E-07
2769	ENSG00000143595.13	AQP10	89872	1.722309262	2.50361E-08	1.43697E-07
2770	ENSG00000236453.5			2.614165523	2.51125E-08	1.44093E-07
2771	ENSG00000270802.2			2.101927874	2.54367E-08	1.45924E-07
2772	ENSG00000262681.2			1.456662741	2.5473E-08	1.46099E-07
2773	ENSG00000287861.1		107984132	4.26034652	2.55762E-08	1.46648E-07
2774	ENSG00000232850.3	PTGES2-AS1	389791	1.407535022	2.56982E-08	1.47305E-07
2775	ENSG00000270038.1			3.979496926	2.58199E-08	1.4796E-07
2776	ENSG00000224789.1			3.324064823	2.58546E-08	1.48137E-07
2777	ENSG00000188820.13	CALHM6	441168	1.522639913	2.61723E-08	1.49914E-07
2778	ENSG00000164850.15	GPER1	2852	1.114146013	2.64798E-08	1.51588E-07
2779	ENSG00000230013.2	CT70		1.377268293	2.66683E-08	1.52623E-07
2780	ENSG00000223023.1	Y_RNA		2.220119987	2.6689E-08	1.52719E-07
2781	ENSG00000185038.14	MROH2A	339766	2.471512494	2.71459E-08	1.552E-07
2782	ENSG00000196534.5	OR9K1P		3.027980099	2.72204E-08	1.55581E-07
2783	ENSG00000071794.16	HLTF	6596	1.08469923	2.72561E-08	1.55756E-07
2784	ENSG00000260139.6	CSPG4P13		1.62395883	2.7263E-08	1.55756E-07
2785	ENSG00000196660.11	SLC30A10	55532	2.137358854	2.73305E-08	1.56052E-07
2786	ENSG00000287923.1			2.52899454	2.76189E-08	1.57608E-07
2787	ENSG00000081051.8	AFP	174	2.202233938	2.76445E-08	1.57732E-07
2788	ENSG00000274840.4	BALR6		4.475050429	2.77889E-08	1.58533E-07
2789	ENSG00000203523.3	TAS2R2		2.418343979	2.79643E-08	1.59465E-07
2790	ENSG00000234584.1			1.138281338	2.82018E-08	1.60796E-07
2791	ENSG00000277322.1	GOLGA6L6	727832	3.10629482	2.82669E-08	1.61143E-07
2792	ENSG00000086506.3	HBQ1	3049	3.031141933	2.90161E-08	1.65248E-07
2793	ENSG00000235748.1	SEPTIN14P12		1.917907198	2.91701E-08	1.66077E-07
2794	ENSG00000280214.1			1.325732534	2.91959E-08	1.66201E-07
2795	ENSG00000139725.8	RHOF	54509	1.118450518	2.94771E-08	1.67657E-07
2796	ENSG00000104808.8	DHDH	27294	1.183144487	2.98919E-08	1.6987E-07
2797	ENSG00000272622.2			1.47896024	3.01792E-08	1.71454E-07
2798	ENSG00000062582.14	MRPS24	64951	1.004412176	3.04021E-08	1.72621E-07
2799	ENSG00000286654.1			1.818309372	3.06419E-08	1.73933E-07
2800	ENSG00000233682.3	FNDC1-AS1		1.578614308	3.0684E-08	1.74147E-07
2801	ENSG00000027644.5	INSRR	3645	1.453439266	3.07287E-08	1.74376E-07
2802	ENSG00000151962.8	RBM46	166863	2.829950198	3.12519E-08	1.77142E-07
2803	ENSG00000279479.1			3.026731108	3.14412E-08	1.78088E-07
2804	ENSG00000270277.1			1.356481895	3.15098E-08	1.78374E-07
2805	ENSG00000151012.13	SLC7A11	23657	1.589215097	3.25177E-08	1.83791E-07
2806	ENSG00000161180.11	CCDC116	164592	1.089232651	3.27351E-08	1.84941E-07
2807	ENSG00000272512.1			1.311020888	3.29008E-08	1.85824E-07
2808	ENSG00000073756.12	PTGS2	5743	1.58994067	3.3495E-08	1.89099E-07
2809	ENSG00000267114.1			1.654149321	3.35597E-08	1.89437E-07
2810	ENSG00000228577.1			2.551333385	3.39456E-08	1.91398E-07
2811	ENSG00000275468.1			1.553421641	3.41916E-08	1.92663E-07
2812	ENSG00000286886.1			2.4890459	3.41943E-08	1.92663E-07
2813	ENSG00000137707.13	BTG4	54766	1.726913021	3.43739E-08	1.93537E-07
2814	ENSG00000105967.16	TFEC	22797	1.136198569	3.44946E-08	1.94162E-07
2815	ENSG00000234722.5	LINC01287	103724390	2.944957902	3.45994E-08	1.94724E-07
2816	ENSG00000225101.6	OR52K3P		1.395728779	3.46824E-08	1.95164E-07
2817	ENSG00000260495.1			1.460608169	3.49094E-08	1.96329E-07
2818	ENSG00000182521.5	TBPL2	387332	2.192854466	3.54949E-08	1.99481E-07
2819	ENSG00000272097.2	TMEM14B-DT	118597831	1.000597698	3.60446E-08	2.02323E-07
2820	ENSG00000286244.1		112268419	1.254393503	3.60465E-08	2.02323E-07
2821	ENSG00000222881.1	Y_RNA		2.231991896	3.62441E-08	2.03374E-07
2822	ENSG00000282849.1		124900414	2.531032892	3.64653E-08	2.04529E-07
2823	ENSG00000230795.3	HLA-K		1.045490859	3.66113E-08	2.05177E-07
2824	ENSG00000157542.11	KCNJ6	3763	1.555871968	3.6612E-08	2.05177E-07
2825	ENSG00000287643.1			1.686308652	3.68794E-08	2.0653E-07
2826	ENSG00000126752.8	SSX1	6756	3.090513625	3.69813E-08	2.07042E-07
2827	ENSG00000070669.17	ASNS	440	1.084835472	3.72375E-08	2.08418E-07
2828	ENSG00000266340.1			1.458396287	3.73603E-08	2.09046E-07
2829	ENSG00000160469.17	BRSK1	84446	1.059239127	3.77514E-08	2.11204E-07
2830	ENSG00000042062.13	RIPOR3	140876	1.205854644	3.79157E-08	2.11974E-07
2831	ENSG00000189013.14	KIR2DL4	3805	1.504045707	3.80242E-08	2.1255E-07
2832	ENSG00000170558.10	CDH2	1000	1.692461573	3.82461E-08	2.13731E-07
2833	ENSG00000161149.12			1.580020845	3.83925E-08	2.14458E-07
2834	ENSG00000119714.11	GPR68	8111	1.04844742	3.88664E-08	2.16953E-07
2835	ENSG00000106327.13	TFR2	7036	1.236111831	3.89375E-08	2.17274E-07
2836	ENSG00000158163.15	DZIP1L	199221	1.009412468	3.92552E-08	2.18999E-07
2837	ENSG00000100285.10	NEFH	4744	2.102891532	3.95251E-08	2.20412E-07
2838	ENSG00000279138.1			1.17037661	3.99837E-08	2.22782E-07
2839	ENSG00000287406.1			2.888256842	4.0236E-08	2.24061E-07
2840	ENSG00000286210.1			1.405769529	4.03537E-08	2.24685E-07
2841	ENSG00000248996.1	PDLIM7-AS1		1.048263665	4.04752E-08	2.25298E-07
2842	ENSG00000161850.3	KRT82	3888	1.761944238	4.05473E-08	2.25668E-07
2843	ENSG00000231672.8	DIRC3	729582	1.486648131	4.10717E-08	2.28491E-07
2844	ENSG00000224287.2			1.409118343	4.20686E-08	2.3384E-07
2845	ENSG00000228818.1			1.011343349	4.2132E-08	2.34159E-07
2846	ENSG00000183674.12	LINC00518		1.886373282	4.24903E-08	2.36085E-07
2847	ENSG00000176349.11			2.498785922	4.25338E-08	2.36293E-07
2848	ENSG00000100053.10	CRYBB3	1417	1.297232783	4.27038E-08	2.37204E-07
2849	ENSG00000253925.1			2.360612727	4.2916E-08	2.3835E-07
2850	ENSG00000244151.1			1.237214002	4.33068E-08	2.40486E-07
2851	ENSG00000287023.1			1.965958416	4.33189E-08	2.4052E-07
2852	ENSG00000231443.2	PPP4R2P2		1.342151653	4.54122E-08	2.51719E-07
2853	ENSG00000187690.4	EZHIP	340602	4.599842286	4.54584E-08	2.5194E-07
2854	ENSG00000235681.1			1.453610146	4.57549E-08	2.53442E-07
2855	ENSG00000101076.17	HNF4A	3172	1.784724316	4.5842E-08	2.53853E-07
2856	ENSG00000173227.14	SYT12	91683	1.342977464	4.6316E-08	2.56407E-07
2857	ENSG00000235052.1			1.56812027	4.69167E-08	2.59514E-07
2858	ENSG00000262692.1			1.099602111	4.70092E-08	2.5999E-07
2859	ENSG00000228470.1			1.431182093	4.70759E-08	2.60286E-07
2860	ENSG00000236562.4	IFITM3P1		1.192483985	4.72971E-08	2.61436E-07
2861	ENSG00000226609.2	SNX30-DT	122455334	1.403153177	4.80256E-08	2.65278E-07
2862	ENSG00000279058.2	AGAP14P		1.702398877	4.83264E-08	2.66828E-07
2863	ENSG00000182272.12	B4GALNT4	338707	1.600836749	4.86704E-08	2.68653E-07
2864	ENSG00000255843.1			2.101260758	4.87601E-08	2.69073E-07
2865	ENSG00000251574.8		105379109	3.278953482	4.90652E-08	2.70643E-07
2866	ENSG00000261807.2	LINC02141	101927580	3.127014255	4.9131E-08	2.70931E-07
2867	ENSG00000270661.1			2.137624853	4.96672E-08	2.73812E-07
2868	ENSG00000128011.5	LRFN1	57622	1.117656722	4.97076E-08	2.73996E-07
2869	ENSG00000254337.2		107986951	2.866643206	4.97261E-08	2.7406E-07
2870	ENSG00000105143.12	SLC1A6	6511	2.607556143	4.97958E-08	2.7433E-07
2871	ENSG00000229780.1	UBE2Q1-AS1	100874097	1.505851558	5.01467E-08	2.76187E-07
2872	ENSG00000185565.12	LSAMP	4045	1.39851787	5.04226E-08	2.77629E-07
2873	ENSG00000270137.1			2.014015405	5.07675E-08	2.79412E-07
2874	ENSG00000186007.10	LEMD1	93273	1.574913541	5.13195E-08	2.82371E-07
2875	ENSG00000253859.2			2.488446325	5.13713E-08	2.82578E-07
2876	ENSG00000286607.1			1.541798904	5.21161E-08	2.86422E-07
2877	ENSG00000180610.10	ZBTB12BP		1.255434799	5.36896E-08	2.94566E-07
2878	ENSG00000259488.2	DUT-AS1		1.175705887	5.36918E-08	2.94566E-07
2879	ENSG00000214776.12			2.058475156	5.39207E-08	2.95658E-07
2880	ENSG00000267197.1			1.337589216	5.40257E-08	2.96192E-07
2881	ENSG00000258912.2	LINC02316	102724380	2.001233749	5.40499E-08	2.96285E-07
2882	ENSG00000286393.1			1.788209735	5.44508E-08	2.98276E-07
2883	ENSG00000128564.7	VGF	7425	1.376366308	5.47372E-08	2.99762E-07
2884	ENSG00000111218.12	PRMT8	56341	2.407380208	5.47484E-08	2.99782E-07
2885	ENSG00000267369.1	TAF5LP1		1.28416372	5.53111E-08	3.02779E-07
2886	ENSG00000253508.1			2.683095626	5.59272E-08	3.05814E-07
2887	ENSG00000261067.7			1.3813689	5.60338E-08	3.06355E-07
2888	ENSG00000282951.1			1.277371932	5.60679E-08	3.06499E-07
2889	ENSG00000257138.1	TAS2R38	5726	2.356114585	5.61839E-08	3.07091E-07
2890	ENSG00000212396.1	RNA5SP323		2.12249419	5.61946E-08	3.07107E-07
2891	ENSG00000120341.18	SEC16B	89866	1.206258114	5.63026E-08	3.07655E-07
2892	ENSG00000231056.1	LINC02522		3.035470007	5.63333E-08	3.07737E-07
2893	ENSG00000165556.10	CDX2	1045	3.069161223	5.64081E-08	3.08104E-07
2894	ENSG00000140107.12	SLC25A47	283600	1.836312618	5.65394E-08	3.08779E-07
2895	ENSG00000187838.17	PLSCR3	57048	1.451390704	5.65618E-08	3.08858E-07
2896	ENSG00000236120.7	LINC03070	101928228	1.911636269	5.66236E-08	3.09068E-07
2897	ENSG00000152595.16	MEPE	56955	2.410459903	5.75587E-08	3.13783E-07
2898	ENSG00000272599.2			1.074777334	5.78799E-08	3.15404E-07
2899	ENSG00000115386.6	REG1A	5967	3.70333624	5.80252E-08	3.16066E-07
2900	ENSG00000108309.14	RUNDC3A	10900	1.375410648	5.84903E-08	3.18468E-07
2901	ENSG00000198286.10	CARD11	84433	1.189204278	6.07686E-08	3.30192E-07
2902	ENSG00000207181.1	SNORA14B	677802	1.118668956	6.10905E-08	3.3185E-07
2903	ENSG00000074317.11	SNCB	6620	1.60470656	6.11151E-08	3.31939E-07
2904	ENSG00000173825.7	TIGD3	220359	1.01292942	6.14048E-08	3.33375E-07
2905	ENSG00000172476.4	RAB40A	142684	1.212136431	6.16353E-08	3.34489E-07
2906	ENSG00000248968.1			2.154150206	6.24208E-08	3.3852E-07
2907	ENSG00000141314.13	RHBDL3	162494	1.576493042	6.27677E-08	3.40355E-07
2908	ENSG00000223648.4	IGHV3-64		2.36571904	6.32598E-08	3.42742E-07
2909	ENSG00000242540.3			2.694640918	6.35342E-08	3.44041E-07
2910	ENSG00000164556.7	CFAP144P1		1.99423278	6.36975E-08	3.44784E-07
2911	ENSG00000277865.4	GOLGA6L22	440243	2.75159049	6.39512E-08	3.4611E-07
2912	ENSG00000267919.1			2.064735875	6.41935E-08	3.47326E-07
2913	ENSG00000201207.1	Y_RNA		1.554042486	6.48456E-08	3.50759E-07
2914	ENSG00000161681.16	SHANK1	50944	1.298016297	6.63545E-08	3.58627E-07
2915	ENSG00000255983.1			1.692305704	6.68795E-08	3.61268E-07
2916	ENSG00000164485.15	IL22RA2	116379	1.495516157	6.69727E-08	3.61672E-07
2917	ENSG00000226953.8	NCKAP5-AS2	101928161	1.398593666	6.74143E-08	3.63908E-07
2918	ENSG00000259485.2	LINC02253	107984764	2.948873626	6.74542E-08	3.64075E-07
2919	ENSG00000261286.1	ATP2C2-AS1	105371374	1.130551256	6.76114E-08	3.64824E-07
2920	ENSG00000230623.6			2.170774202	6.87622E-08	3.70832E-07
2921	ENSG00000135119.15	RNFT2	84900	1.40559801	6.9169E-08	3.72876E-07
2922	ENSG00000279220.4	CMKLR2-AS		2.496916093	6.91695E-08	3.72876E-07
2923	ENSG00000272259.5	LINC01749		2.688956957	7.05214E-08	3.79957E-07
2924	ENSG00000271032.1			1.313050617	7.08029E-08	3.81422E-07
2925	ENSG00000162188.6	GNG3	2785	1.485408715	7.09154E-08	3.81924E-07
2926	ENSG00000225857.5	LINC02816		1.68213153	7.12423E-08	3.83633E-07
2927	ENSG00000163508.13	EOMES	8320	1.251787233	7.17511E-08	3.86162E-07
2928	ENSG00000278928.1			1.770263662	7.1991E-08	3.87401E-07
2929	ENSG00000164638.10	SLC29A4	222962	1.315628673	7.21021E-08	3.87946E-07
2930	ENSG00000174672.16	BRSK2	9024	1.54554031	7.28976E-08	3.9196E-07
2931	ENSG00000211950.2	IGHV1-24		2.172706221	7.32717E-08	3.93812E-07
2932	ENSG00000183734.4	ASCL2	430	1.162825955	7.35956E-08	3.95392E-07
2933	ENSG00000283047.1	FRG1FP		2.625075169	7.47318E-08	4.01279E-07
2934	ENSG00000286195.1			2.063654492	7.49564E-08	4.02376E-07
2935	ENSG00000280083.1			1.742836718	7.52794E-08	4.03945E-07
2936	ENSG00000264876.2			2.547991346	7.60779E-08	4.08065E-07
2937	ENSG00000287899.1			1.971219614	7.61644E-08	4.08473E-07
2938	ENSG00000226650.6	KIF4B	285643	1.41001951	7.66108E-08	4.10756E-07
2939	ENSG00000243094.1	RPL32P2		1.335728351	7.79623E-08	4.17551E-07
2940	ENSG00000212296.1	SNORD72	619564	1.954451889	7.81527E-08	4.18458E-07
2941	ENSG00000089012.14	SIRPG	55423	1.264098051	7.85357E-08	4.20338E-07
2942	ENSG00000233033.1	CASK-AS1		1.961177784	7.98705E-08	4.26906E-07
2943	ENSG00000125207.7	PIWIL1	9271	2.257433793	8.24099E-08	4.39688E-07
2944	ENSG00000256975.1			2.232690764	8.24173E-08	4.39688E-07
2945	ENSG00000180616.9	SSTR2	6752	1.309086854	8.28986E-08	4.41984E-07
2946	ENSG00000120498.14	TEX11	56159	1.352364699	8.29034E-08	4.41984E-07
2947	ENSG00000236516.1	KLF2P4		4.145006708	8.36447E-08	4.45637E-07
2948	ENSG00000235027.1	PRADX	126568849	1.259586091	8.42771E-08	4.48885E-07
2949	ENSG00000243650.3	RN7SL834P		1.272453523	8.45838E-08	4.50397E-07
2950	ENSG00000279196.1			1.296268019	8.4595E-08	4.50397E-07
2951	ENSG00000260001.6	TGFBR3L	100507588	1.573166536	8.59429E-08	4.57021E-07
2952	ENSG00000264290.1			1.224364452	8.64448E-08	4.59567E-07
2953	ENSG00000271889.1	C2orf74-AS1	339803	1.943689989	8.64736E-08	4.59658E-07
2954	ENSG00000250033.5	SLC7A11-AS1	641364	2.0611946	8.66908E-08	4.6069E-07
2955	ENSG00000225756.1	DBH-AS1	138948	1.45634138	8.67153E-08	4.60758E-07
2956	ENSG00000138741.11	TRPC3	7222	1.102139838	8.67976E-08	4.61133E-07
2957	ENSG00000155893.13	PXYLP1	92370	1.028983549	8.69072E-08	4.61654E-07
2958	ENSG00000241544.2	LINC02029	105374177	1.832539274	8.76331E-08	4.65385E-07
2959	ENSG00000170323.9	FABP4	2167	2.501582449	8.77476E-08	4.65931E-07
2960	ENSG00000132854.19	KANK4	163782	1.743413707	8.96421E-08	4.75736E-07
2961	ENSG00000280388.1			1.075419764	9.01564E-08	4.78401E-07
2962	ENSG00000283071.1	LBHD2	107984640	2.258305764	9.02589E-08	4.78817E-07
2963	ENSG00000226025.9			1.98682515	9.04341E-08	4.79575E-07
2964	ENSG00000227115.8	LINC01630	100287225	2.122995983	9.06338E-08	4.80484E-07
2965	ENSG00000146648.19	EGFR	1956	1.076276844	9.13707E-08	4.83809E-07
2966	ENSG00000063515.3	GSC2	2928	1.812825368	9.17588E-08	4.85734E-07
2967	ENSG00000261761.5	LINC02616	100508631	4.700345798	9.20978E-08	4.87398E-07
2968	ENSG00000124134.9	KCNS1	3787	1.958138094	9.22401E-08	4.88086E-07
2969	ENSG00000226476.6	LINC01748	105378763	1.863582051	9.29622E-08	4.91579E-07
2970	ENSG00000270427.1	NRBF2P5		1.229782204	9.37949E-08	4.95718E-07
2971	ENSG00000243144.7	LINC02932	105375392	2.029668904	9.38078E-08	4.9572E-07
2972	ENSG00000272849.1			1.115774986	9.39137E-08	4.96147E-07
2973	ENSG00000211893.4	IGHG2		1.89330497	9.46979E-08	4.99957E-07
2974	ENSG00000186075.13	ZPBP2	124626	4.380660935	9.4739E-08	5.00108E-07
2975	ENSG00000270019.1			1.096403124	9.49821E-08	5.01257E-07
2976	ENSG00000228496.1		100506405	4.168724956	9.52797E-08	5.0256E-07
2977	ENSG00000136531.18	SCN2A	6326	2.107626398	9.54087E-08	5.03174E-07
2978	ENSG00000256084.1			2.047652389	9.60797E-08	5.06577E-07
2979	ENSG00000125571.9	IL37	27178	1.892594629	9.64612E-08	5.08522E-07
2980	ENSG00000254743.1	OR10V3P		2.6757627	9.6577E-08	5.09064E-07
2981	ENSG00000253603.1	CERNA3		1.944973473	9.75331E-08	5.13899E-07
2982	ENSG00000183242.12	WT1-AS	51352	3.047653144	9.76297E-08	5.14339E-07
2983	ENSG00000232065.1	LINC01063		1.329455748	9.84182E-08	5.18012E-07
2984	ENSG00000154764.6	WNT7A	7476	1.501420289	9.8847E-08	5.2013E-07
2985	ENSG00000009694.13	TENM1	10178	1.97934396	9.91417E-08	5.21474E-07
2986	ENSG00000257222.1			1.170541627	1.00296E-07	5.27128E-07
2987	ENSG00000287630.1			3.160504214	1.00422E-07	5.27719E-07
2988	ENSG00000234806.1	RPL26P29		2.154072649	1.01881E-07	5.34888E-07
2989	ENSG00000226535.1			2.166956748	1.01924E-07	5.35045E-07
2990	ENSG00000227012.3	LINC02527	105377946	2.933779743	1.02186E-07	5.36277E-07
2991	ENSG00000248870.1			2.347792239	1.02251E-07	5.36546E-07
2992	ENSG00000260763.1		124901168	2.000078988	1.03156E-07	5.41083E-07
2993	ENSG00000248161.5	NFKB1-AS1		1.047011914	1.03579E-07	5.43084E-07
2994	ENSG00000226174.7	TEX22	647310	1.147552835	1.05374E-07	5.52129E-07
2995	ENSG00000164509.15	IL31RA	133396	1.212394779	1.05805E-07	5.54167E-07
2996	ENSG00000274184.1			1.570705683	1.06096E-07	5.55545E-07
2997	ENSG00000155265.11	GOLGA7B	401647	1.311346857	1.06405E-07	5.57051E-07
2998	ENSG00000100206.10	DMC1	11144	1.066016789	1.06411E-07	5.57051E-07
2999	ENSG00000175697.11	GPR156	165829	1.369780232	1.06556E-07	5.57736E-07
3000	ENSG00000261873.2	SMIM36	101927367	2.219109721	1.06713E-07	5.58484E-07
3001	ENSG00000274099.1	ABCB10P1		3.474502462	1.06927E-07	5.5953E-07
3002	ENSG00000236799.1	LINC02626	102724589	1.9873811	1.07702E-07	5.63288E-07
3003	ENSG00000171450.6	CDK5R2	8941	2.139811206	1.07783E-07	5.63559E-07
3004	ENSG00000280411.1	IGHV1-69D		2.097901752	1.08571E-07	5.67459E-07
3005	ENSG00000277978.1			1.098380889	1.08674E-07	5.67922E-07
3006	ENSG00000204539.4	CDSN	1041	2.30881062	1.08726E-07	5.68119E-07
3007	ENSG00000253307.1			1.364554427	1.09521E-07	5.72044E-07
3008	ENSG00000204362.6	LINC02783	400743	1.552634601	1.1076E-07	5.78213E-07
3009	ENSG00000135100.18	HNF1A	6927	2.437605324	1.11746E-07	5.83128E-07
3010	ENSG00000125531.7	FNDC11	79025	1.132653834	1.12536E-07	5.8702E-07
3011	ENSG00000268756.1			1.484493868	1.12866E-07	5.88662E-07
3012	ENSG00000224853.3	LINC00393		2.065364898	1.15433E-07	6.01026E-07
3013	ENSG00000286147.1			2.293145856	1.15472E-07	6.0115E-07
3014	ENSG00000261460.1	CELF6-AS1	105370887	1.778549932	1.15847E-07	6.02863E-07
3015	ENSG00000263990.1			1.18006223	1.17268E-07	6.10098E-07
3016	ENSG00000206633.1	SNORA80B	100302743	2.126401337	1.17526E-07	6.11359E-07
3017	ENSG00000250116.2	RHOQ-AS1	100506142	1.183624457	1.17777E-07	6.12503E-07
3018	ENSG00000269191.1			1.394392906	1.18636E-07	6.1673E-07
3019	ENSG00000267121.6	FMNL1-DT		1.133174034	1.19115E-07	6.1914E-07
3020	ENSG00000264859.6	DSG2-AS1		1.133377564	1.19176E-07	6.19295E-07
3021	ENSG00000233191.2			2.244782124	1.19529E-07	6.20967E-07
3022	ENSG00000287788.1			1.266113715	1.19579E-07	6.21144E-07
3023	ENSG00000273100.1			1.25515115	1.19892E-07	6.22604E-07
3024	ENSG00000214770.3			1.116269055	1.20534E-07	6.25775E-07
3025	ENSG00000258769.1	RAP1AP		1.878353023	1.21207E-07	6.29024E-07
3026	ENSG00000273179.1	SPON2-AS1		1.102826597	1.23505E-07	6.40444E-07
3027	ENSG00000196990.9	FAM163B	642968	1.90220279	1.24029E-07	6.42995E-07
3028	ENSG00000260777.1			2.807561788	1.25091E-07	6.48077E-07
3029	ENSG00000260121.1			1.252986616	1.25688E-07	6.51085E-07
3030	ENSG00000120669.16	SOHLH2	54937	2.154255917	1.26027E-07	6.52758E-07
3031	ENSG00000240893.1	LINC02042	101929694	2.951453349	1.27408E-07	6.59218E-07
3032	ENSG00000229648.1	RPSAP22		2.278697338	1.28906E-07	6.66535E-07
3033	ENSG00000255146.1			2.586343202	1.2936E-07	6.68795E-07
3034	ENSG00000277775.2	H3C7	8968	1.213052949	1.29571E-07	6.6971E-07
3035	ENSG00000175265.17	GOLGA8A	23015	1.219150168	1.29742E-07	6.7051E-07
3036	ENSG00000227204.1	RBMY2JP		3.300272639	1.30565E-07	6.74496E-07
3037	ENSG00000232109.1	VN1R54P		3.301466181	1.3079E-07	6.75572E-07
3038	ENSG00000114646.10	CSPG5	10675	1.18379878	1.3124E-07	6.7772E-07
3039	ENSG00000208772.1	SNORD94	692225	1.086459674	1.32755E-07	6.84583E-07
3040	ENSG00000154646.9	TMPRSS15	5651	2.850988392	1.32759E-07	6.84583E-07
3041	ENSG00000245322.7	H2AZ1-DT		1.055068774	1.34603E-07	6.93639E-07
3042	ENSG00000135925.9	WNT10A	80326	1.255050916	1.34726E-07	6.94187E-07
3043	ENSG00000159753.14	CARMIL2	146206	1.024088886	1.35087E-07	6.95953E-07
3044	ENSG00000237819.5	CDK6-AS1		1.68352376	1.35447E-07	6.97717E-07
3045	ENSG00000126583.11	PRKCG	5582	1.884051171	1.36087E-07	7.00926E-07
3046	ENSG00000223922.1	ASS1P2		1.231353994	1.3806E-07	7.10808E-07
3047	ENSG00000225807.2			2.265137851	1.38308E-07	7.11994E-07
3048	ENSG00000230836.1			1.65929058	1.3962E-07	7.18283E-07
3049	ENSG00000251405.3	NIPAL4-DT		1.828891971	1.39752E-07	7.18867E-07
3050	ENSG00000235290.1	HLA-W		1.269318932	1.40092E-07	7.20523E-07
3051	ENSG00000260593.1			2.098810415	1.40309E-07	7.21543E-07
3052	ENSG00000140675.13	SLC5A2	6524	1.623184917	1.41609E-07	7.27761E-07
3053	ENSG00000157322.17	CLEC18A	348174	1.605881158	1.42433E-07	7.31803E-07
3054	ENSG00000147437.10	GNRH1	2796	1.022310048	1.43172E-07	7.35315E-07
3055	ENSG00000126231.15	PROZ	8858	1.400136662	1.43461E-07	7.36607E-07
3056	ENSG00000256690.1	STX5-DT	105369332	1.376278335	1.43632E-07	7.3739E-07
3057	ENSG00000259709.1			1.815066735	1.45165E-07	7.44777E-07
3058	ENSG00000252892.1	RNU6-548P		2.025713661	1.45426E-07	7.46019E-07
3059	ENSG00000235979.9		105371574	2.866664344	1.45832E-07	7.48007E-07
3060	ENSG00000235086.1	FNDC1-IT1	100874312	2.394750081	1.46431E-07	7.50791E-07
3061	ENSG00000228049.7	POLR2J2	246721	1.366117475	1.48654E-07	7.61399E-07
3062	ENSG00000260135.7	MMP2-AS1	105371279	1.771827056	1.50421E-07	7.69654E-07
3063	ENSG00000261594.4	TPBGL	100507050	1.024962477	1.50444E-07	7.69671E-07
3064	ENSG00000261342.1			1.05276491	1.50777E-07	7.71276E-07
3065	ENSG00000161911.11	TREML1	340205	1.024046883	1.51892E-07	7.76477E-07
3066	ENSG00000261997.1			1.182132249	1.528E-07	7.80718E-07
3067	ENSG00000118017.4	A4GNT	51146	2.212948858	1.53428E-07	7.83625E-07
3068	ENSG00000176912.5	TYMSOS		1.202451246	1.53609E-07	7.84449E-07
3069	ENSG00000164683.18	HEY1	23462	1.251858063	1.53711E-07	7.84864E-07
3070	ENSG00000286859.1			1.214088805	1.54008E-07	7.86182E-07
3071	ENSG00000225411.3			1.236973924	1.54232E-07	7.87124E-07
3072	ENSG00000237693.4	IRGM	345611	1.644035453	1.54857E-07	7.90109E-07
3073	ENSG00000269938.1			1.583570407	1.55001E-07	7.90742E-07
3074	ENSG00000260103.2			2.00532418	1.55439E-07	7.92771E-07
3075	ENSG00000200502.1	Y_RNA		1.132783361	1.56576E-07	7.98363E-07
3076	ENSG00000140557.12	ST8SIA2	8128	1.443600592	1.58082E-07	8.05318E-07
3077	ENSG00000228915.3	OR7E128P		1.331118155	1.58859E-07	8.09175E-07
3078	ENSG00000178233.18	TMEM151B	441151	2.198161896	1.62299E-07	8.2574E-07
3079	ENSG00000286536.1			1.368909456	1.63397E-07	8.30845E-07
3080	ENSG00000232479.2	KRT8P52		1.415961756	1.64261E-07	8.34815E-07
3081	ENSG00000225535.7	LINC01393	102724386	1.119643427	1.64537E-07	8.36052E-07
3082	ENSG00000253892.1			2.395008823	1.64701E-07	8.36675E-07
3083	ENSG00000253712.1	LINC02886		2.154228736	1.66552E-07	8.45573E-07
3084	ENSG00000280117.1			2.666492329	1.66593E-07	8.45635E-07
3085	ENSG00000179097.6	HTR1F	3355	1.351851892	1.66988E-07	8.47363E-07
3086	ENSG00000280208.1	GGT4P		2.296998823	1.67124E-07	8.47788E-07
3087	ENSG00000183773.15	AIFM3	150209	1.159978396	1.68759E-07	8.5586E-07
3088	ENSG00000269210.2		375196	2.924349077	1.69024E-07	8.57094E-07
3089	ENSG00000225064.1	LINC01802		2.187703987	1.69818E-07	8.60682E-07
3090	ENSG00000178033.6	CALHM5	254228	1.020047741	1.72657E-07	8.73953E-07
3091	ENSG00000258450.1			1.749836241	1.7316E-07	8.76142E-07
3092	ENSG00000224903.2	RNF32-AS1		1.022614374	1.73583E-07	8.77966E-07
3093	ENSG00000242021.2			3.191724138	1.74504E-07	8.82514E-07
3094	ENSG00000259804.1	CTCF-DT	100505942	1.104994864	1.75813E-07	8.88909E-07
3095	ENSG00000232774.8	LINC03033		1.067863866	1.76028E-07	8.89766E-07
3096	ENSG00000267546.2			1.176516511	1.77464E-07	8.96914E-07
3097	ENSG00000182264.9	IZUMO1	284359	1.2303834	1.77835E-07	8.98328E-07
3098	ENSG00000259668.6			1.013634377	1.78375E-07	9.00939E-07
3099	ENSG00000136883.14	KIF12	113220	1.505323831	1.78617E-07	9.02048E-07
3100	ENSG00000242798.1			1.128156626	1.79118E-07	9.0435E-07
3101	ENSG00000280097.1			2.297635631	1.79307E-07	9.05187E-07
3102	ENSG00000185186.10	LINC00313		1.597863894	1.80095E-07	9.08817E-07
3103	ENSG00000228573.1			2.609304977	1.80263E-07	9.09552E-07
3104	ENSG00000234362.6	LINC01914		1.612707438	1.8092E-07	9.12516E-07
3105	ENSG00000258451.1	EGILA		2.742884237	1.82448E-07	9.19756E-07
3106	ENSG00000145649.8	GZMA	3001	1.316780855	1.8253E-07	9.19934E-07
3107	ENSG00000278463.2	H2AC4	8335	1.611448956	1.83033E-07	9.22234E-07
3108	ENSG00000161133.16	USP41P		1.837882878	1.8483E-07	9.30818E-07
3109	ENSG00000230666.5	CEACAM22P		2.053012798	1.87152E-07	9.41915E-07
3110	ENSG00000116661.11	FBXO2	26232	1.259367433	1.87487E-07	9.4348E-07
3111	ENSG00000237594.2	TIAM1-AS1	150051	1.300853219	1.88033E-07	9.45986E-07
3112	ENSG00000267313.7	KC6	641516	2.024189761	1.88088E-07	9.46141E-07
3113	ENSG00000249755.2			1.921413724	1.88162E-07	9.46396E-07
3114	ENSG00000267078.1			1.744763709	1.90835E-07	9.59598E-07
3115	ENSG00000260944.1	FOXC2-AS1		2.610564213	1.92225E-07	9.65974E-07
3116	ENSG00000185903.10	OR11N1P		2.31739105	1.93048E-07	9.69861E-07
3117	ENSG00000234199.3	LINC01191	440900	1.323921741	1.94697E-07	9.77483E-07
3118	ENSG00000264644.1	KRT18P8		1.641690499	1.94713E-07	9.77483E-07
3119	ENSG00000279845.1			1.711720723	1.95302E-07	9.79947E-07
3120	ENSG00000233473.2	RAD1P2		1.820532947	1.95795E-07	9.81921E-07
3121	ENSG00000211662.2	IGLV3-21		1.950858408	1.98591E-07	9.94938E-07
3122	ENSG00000186583.12	SPATC1	375686	1.078902568	2.02718E-07	1.01497E-06
3123	ENSG00000196132.14	MYT1	4661	1.476598353	2.03064E-07	1.01657E-06
3124	ENSG00000250198.3	LINC02199	101929284	2.383179531	2.05081E-07	1.02602E-06
3125	ENSG00000261548.1	HLA-P		2.158474892	2.05727E-07	1.02913E-06
3126	ENSG00000250490.1	LINC02145	401172	1.624652253	2.07678E-07	1.03836E-06
3127	ENSG00000286534.1			2.962356081	2.09752E-07	1.04794E-06
3128	ENSG00000251410.2			2.692023377	2.1281E-07	1.06268E-06
3129	ENSG00000228561.2			1.901472596	2.17187E-07	1.08345E-06
3130	ENSG00000235672.1			1.676641846	2.17388E-07	1.08431E-06
3131	ENSG00000266835.6	GAPLINC	100505592	1.093072977	2.19474E-07	1.09403E-06
3132	ENSG00000285520.1			1.321429491	2.19881E-07	1.09592E-06
3133	ENSG00000168348.4	INSM2	84684	2.082288742	2.22394E-07	1.10761E-06
3134	ENSG00000171049.9	FPR2	2358	1.415924314	2.23329E-07	1.11199E-06
3135	ENSG00000256039.2	LINC02446	101060038	1.642268075	2.24724E-07	1.11866E-06
3136	ENSG00000134245.18	WNT2B	7482	1.142386011	2.27475E-07	1.13178E-06
3137	ENSG00000227094.1			1.949832127	2.27955E-07	1.13374E-06
3138	ENSG00000167355.8	OR51B5	282763	3.270731516	2.29203E-07	1.13931E-06
3139	ENSG00000145692.15	BHMT	635	1.974167238	2.31497E-07	1.14977E-06
3140	ENSG00000130540.14	SULT4A1	25830	2.159452499	2.32166E-07	1.15295E-06
3141	ENSG00000234460.2			3.269758528	2.3237E-07	1.15382E-06
3142	ENSG00000287858.1			2.333586466	2.33933E-07	1.16114E-06
3143	ENSG00000198691.13	ABCA4	24	1.623596522	2.34297E-07	1.1628E-06
3144	ENSG00000245148.2	ARAP1-AS2	100506020	1.352815373	2.35995E-07	1.17094E-06
3145	ENSG00000233783.8			1.461201944	2.3699E-07	1.17558E-06
3146	ENSG00000277173.1			1.9281345	2.37666E-07	1.17879E-06
3147	ENSG00000285748.2			1.662524039	2.39807E-07	1.18896E-06
3148	ENSG00000227953.7	LINC01341		1.607706206	2.40397E-07	1.19173E-06
3149	ENSG00000260293.2			1.532653782	2.4128E-07	1.19596E-06
3150	ENSG00000154040.21	CABYR	26256	1.229483852	2.50393E-07	1.23974E-06
3151	ENSG00000120322.4	PCDHB8	56128	1.496740618	2.50969E-07	1.24243E-06
3152	ENSG00000286017.1			1.135660016	2.52009E-07	1.24743E-06
3153	ENSG00000101825.8	MXRA5	25878	1.170967393	2.53902E-07	1.25633E-06
3154	ENSG00000117971.12	CHRNB4	1143	1.538785128	2.55018E-07	1.26169E-06
3155	ENSG00000285851.1			1.100417298	2.57218E-07	1.27194E-06
3156	ENSG00000049247.14	UTS2	10911	1.631788675	2.58015E-07	1.27557E-06
3157	ENSG00000229243.2	LINC01981	100996624	2.227208418	2.60146E-07	1.28528E-06
3158	ENSG00000234226.5	NDUFS5P2		2.690012203	2.60175E-07	1.28528E-06
3159	ENSG00000267372.2			1.430924539	2.61258E-07	1.29031E-06
3160	ENSG00000267943.1			2.277623365	2.61562E-07	1.29165E-06
3161	ENSG00000120075.5	HOXB5	3215	1.376767578	2.62921E-07	1.29788E-06
3162	ENSG00000181690.8	PLAG1	5324	1.21177397	2.64557E-07	1.30563E-06
3163	ENSG00000286122.1	LINC02964		1.610793051	2.66027E-07	1.31207E-06
3164	ENSG00000132749.11	TESMIN	9633	1.111088131	2.66744E-07	1.31544E-06
3165	ENSG00000279467.1			1.432899698	2.67217E-07	1.31728E-06
3166	ENSG00000214725.9	CDIPTOSP		1.737639942	2.68453E-07	1.32321E-06
3167	ENSG00000169896.18	ITGAM	3684	1.103620233	2.74961E-07	1.35327E-06
3168	ENSG00000225075.1			1.390375982	2.75961E-07	1.35802E-06
3169	ENSG00000137558.9	PI15	51050	1.234239348	2.77683E-07	1.36616E-06
3170	ENSG00000266995.1			1.27869213	2.78445E-07	1.36973E-06
3171	ENSG00000203756.8	TMEM244	253582	2.008774518	2.80017E-07	1.37712E-06
3172	ENSG00000104901.7	DKKL1	27120	1.382430516	2.81413E-07	1.38348E-06
3173	ENSG00000215612.8	HMX1	3166	2.095996557	2.85635E-07	1.40371E-06
3174	ENSG00000100453.13	GZMB	3002	1.242869574	2.86646E-07	1.40815E-06
3175	ENSG00000232732.11		101927687	1.652424048	2.87593E-07	1.41263E-06
3176	ENSG00000231381.2	RNF2P1		2.490213146	2.90626E-07	1.42701E-06
3177	ENSG00000286742.1			1.232785125	2.93693E-07	1.44099E-06
3178	ENSG00000273443.1			1.554558705	2.96945E-07	1.45606E-06
3179	ENSG00000153707.17	PTPRD	5789	1.516910306	2.97174E-07	1.45699E-06
3180	ENSG00000262482.1	FLYWCH1-AS1	105371055	1.560565848	2.97474E-07	1.4581E-06
3181	ENSG00000234068.7	PAGE2	203569	4.744716892	2.98048E-07	1.46055E-06
3182	ENSG00000184486.10	POU3F2	5454	1.716914832	3.01732E-07	1.47787E-06
3183	ENSG00000264265.1	LINC01925	105376854	3.175518495	3.04721E-07	1.49141E-06
3184	ENSG00000274653.1			1.202011517	3.06196E-07	1.49826E-06
3185	ENSG00000187173.4	LCE2A	353139	3.000163444	3.06775E-07	1.5009E-06
3186	ENSG00000255267.4	LINC02687	399886	1.669224477	3.07087E-07	1.50225E-06
3187	ENSG00000284610.1			1.299886963	3.1228E-07	1.52633E-06
3188	ENSG00000265112.1	MIR3153	100422936	1.95532198	3.12773E-07	1.52855E-06
3189	ENSG00000234703.1	RUNX1-AS2		2.467679731	3.13743E-07	1.5331E-06
3190	ENSG00000105880.7	DLX5	1749	1.079256151	3.14173E-07	1.53501E-06
3191	ENSG00000285948.1			1.896434557	3.16082E-07	1.54377E-06
3192	ENSG00000242078.1			1.762440392	3.16837E-07	1.54708E-06
3193	ENSG00000179219.5	LINC00311		1.8892455	3.20722E-07	1.56508E-06
3194	ENSG00000107593.17	PKD2L1	9033	1.240049867	3.22164E-07	1.57154E-06
3195	ENSG00000130477.15	UNC13A	23025	1.141834841	3.23104E-07	1.57574E-06
3196	ENSG00000141485.17	SLC13A5	284111	1.984625629	3.23276E-07	1.57638E-06
3197	ENSG00000213130.3	EEF1DP5		2.539645111	3.2336E-07	1.5766E-06
3198	ENSG00000228863.8			1.347731055	3.2494E-07	1.5835E-06
3199	ENSG00000268460.2		93429	1.376640898	3.24976E-07	1.5835E-06
3200	ENSG00000270980.1			2.864217529	3.25888E-07	1.58736E-06
3201	ENSG00000279518.1			1.132719846	3.26357E-07	1.58936E-06
3202	ENSG00000287742.1			1.224842868	3.26377E-07	1.58936E-06
3203	ENSG00000283525.1			1.65707634	3.26539E-07	1.58995E-06
3204	ENSG00000256463.8	SALL3	27164	2.977866554	3.29414E-07	1.60335E-06
3205	ENSG00000152049.6	KCNE4	23704	1.045721211	3.29749E-07	1.60479E-06
3206	ENSG00000147509.14	RGS20	8601	1.172196881	3.31024E-07	1.61044E-06
3207	ENSG00000249425.1	LINC02477		2.846758444	3.32139E-07	1.61523E-06
3208	ENSG00000182870.13	GALNT9	50614	1.770658754	3.3271E-07	1.61781E-06
3209	ENSG00000196289.7	BECN2	441925	2.501176633	3.33247E-07	1.62022E-06
3210	ENSG00000255753.1			2.172042831	3.40225E-07	1.65192E-06
3211	ENSG00000259353.1			2.555469628	3.40853E-07	1.65477E-06
3212	ENSG00000248964.7		101929470	1.437366192	3.42025E-07	1.66005E-06
3213	ENSG00000273032.3	DGCR5	26220	1.142930486	3.43125E-07	1.66519E-06
3214	ENSG00000233924.1	RPSAP13		1.829213653	3.44594E-07	1.6715E-06
3215	ENSG00000272595.2	OR10AH1P		2.107871913	3.46046E-07	1.67833E-06
3216	ENSG00000168703.5	WFDC12	128488	2.173686623	3.48182E-07	1.6881E-06
3217	ENSG00000158516.12	CPA2	1358	1.828825951	3.50297E-07	1.69812E-06
3218	ENSG00000265702.2	MARCHF10-AS1	105371855	1.583583194	3.50915E-07	1.7007E-06
3219	ENSG00000228917.1	NECTIN4-AS1		1.196958962	3.58664E-07	1.73656E-06
3220	ENSG00000240440.1			1.91847124	3.5875E-07	1.73676E-06
3221	ENSG00000215515.2	IFIT1P1		1.789504031	3.60122E-07	1.74255E-06
3222	ENSG00000178172.7	SPINK6	404203	2.341609805	3.62463E-07	1.75367E-06
3223	ENSG00000186704.9	DTX2P1		1.541335276	3.64505E-07	1.76311E-06
3224	ENSG00000179455.10	MKRN3	7681	1.097087587	3.65317E-07	1.76683E-06
3225	ENSG00000261392.1			2.062397103	3.70615E-07	1.79201E-06
3226	ENSG00000286186.1			1.265761417	3.71838E-07	1.79749E-06
3227	ENSG00000134443.10	GRP	2922	1.865629804	3.74655E-07	1.81071E-06
3228	ENSG00000256843.1			2.109793759	3.77892E-07	1.82542E-06
3229	ENSG00000241560.7	ZBTB20-AS1	100131117	1.456331703	3.78208E-07	1.8265E-06
3230	ENSG00000279283.1			1.295689073	3.78664E-07	1.82848E-06
3231	ENSG00000115602.17	IL1RL1	9173	1.486179282	3.79191E-07	1.8308E-06
3232	ENSG00000237988.5	OR2I1	442197	1.583489422	3.805E-07	1.83667E-06
3233	ENSG00000280445.1			2.49898502	3.89292E-07	1.87729E-06
3234	ENSG00000263916.1			1.365032629	3.89523E-07	1.87794E-06
3235	ENSG00000218561.1			3.520787372	3.90992E-07	1.8848E-06
3236	ENSG00000258602.1	LINC01629	105370578	2.24085046	3.94314E-07	1.89919E-06
3237	ENSG00000258777.1			1.262247541	3.94764E-07	1.9009E-06
3238	ENSG00000277744.1			1.325132414	3.95265E-07	1.90262E-06
3239	ENSG00000282021.1			1.044781454	3.95523E-07	1.90363E-06
3240	ENSG00000260328.1			1.2992207	3.95821E-07	1.90437E-06
3241	ENSG00000171532.5	NEUROD2	4761	1.936651769	3.9812E-07	1.91427E-06
3242	ENSG00000225269.3	LINC00705		1.341087052	3.98264E-07	1.91473E-06
3243	ENSG00000145824.13	CXCL14	9547	1.439537318	4.06767E-07	1.95465E-06
3244	ENSG00000179698.14	WDR97	340390	1.264813472	4.06814E-07	1.95465E-06
3245	ENSG00000243620.2		112268449	3.386451747	4.06964E-07	1.95513E-06
3246	ENSG00000246214.2	RETREG1-AS1	101929524	1.153601065	4.07976E-07	1.95952E-06
3247	ENSG00000232729.8	GTF2I-AS1		1.326566441	4.08274E-07	1.96071E-06
3248	ENSG00000278909.1			2.331908849	4.10873E-07	1.97272E-06
3249	ENSG00000236009.1			2.937975575	4.14081E-07	1.98643E-06
3250	ENSG00000134765.10	DSC1	1823	2.087848178	4.16645E-07	1.99777E-06
3251	ENSG00000256008.3	CABP1-DT		2.620827109	4.21354E-07	2.01816E-06
3252	ENSG00000196155.13	PLEKHG4	25894	1.005810009	4.21356E-07	2.01816E-06
3253	ENSG00000253214.2		105375924	1.213247787	4.22334E-07	2.02187E-06
3254	ENSG00000242551.2	POU5F1P6		2.059501982	4.22417E-07	2.02202E-06
3255	ENSG00000271590.1			1.455383717	4.2408E-07	2.02925E-06
3256	ENSG00000236358.1			1.887053678	4.25069E-07	2.03349E-06
3257	ENSG00000280157.1			1.264256679	4.25788E-07	2.03644E-06
3258	ENSG00000137101.13	CD72	971	1.125983195	4.28261E-07	2.04777E-06
3259	ENSG00000146453.13	PNLDC1	154197	2.24816238	4.29714E-07	2.05422E-06
3260	ENSG00000232586.1	KIAA1614-AS1	103344928	1.731705008	4.30067E-07	2.05517E-06
3261	ENSG00000265539.1	MIR3164	100422846	2.2427669	4.31393E-07	2.06126E-06
3262	ENSG00000278195.2	SSTR3	6753	1.293014505	4.32553E-07	2.06655E-06
3263	ENSG00000274356.1			1.422819257	4.36566E-07	2.08472E-06
3264	ENSG00000213999.17	MEF2B	4207	1.566252852	4.40045E-07	2.10082E-06
3265	ENSG00000179603.18	GRM8	2918	1.087786734	4.40585E-07	2.10315E-06
3266	ENSG00000222764.1	RN7SKP106		2.291568438	4.43302E-07	2.11586E-06
3267	ENSG00000058866.15	DGKG	1608	1.232763796	4.50059E-07	2.14578E-06
3268	ENSG00000214514.8	KRT42P		1.425430093	4.50629E-07	2.14799E-06
3269	ENSG00000287445.1		105372906	1.109538187	4.57329E-07	2.17862E-06
3270	ENSG00000248578.1	NPM1P21		1.5646208	4.57559E-07	2.17921E-06
3271	ENSG00000215277.8	RNF212B	100507650	1.224153686	4.57619E-07	2.17921E-06
3272	ENSG00000273736.1			1.990459848	4.58812E-07	2.18411E-06
3273	ENSG00000199804.1	RNA5SP383		1.174755872	4.59406E-07	2.18667E-06
3274	ENSG00000287921.1			2.407303807	4.6949E-07	2.23065E-06
3275	ENSG00000232671.6	ZNF687-AS1		1.046776126	4.69966E-07	2.23265E-06
3276	ENSG00000251521.2	IMPA1P1		2.230666541	4.72368E-07	2.24379E-06
3277	ENSG00000214894.6	LINC00243	401247	1.08933985	4.76843E-07	2.26396E-06
3278	ENSG00000267387.1			1.833491843	4.77702E-07	2.2675E-06
3279	ENSG00000246877.1	DNM1P35		1.03014652	4.80215E-07	2.27806E-06
3280	ENSG00000122687.19	MRM2	29960	1.122810322	4.84798E-07	2.29843E-06
3281	ENSG00000277218.1			1.409041457	4.89972E-07	2.32101E-06
3282	ENSG00000266126.1			1.479564977	4.95952E-07	2.3471E-06
3283	ENSG00000249326.1	CTD-2194D22.4	101929081	1.675651043	4.96287E-07	2.34829E-06
3284	ENSG00000178297.14	TMPRSS9	360200	1.040939203	5.05662E-07	2.38992E-06
3285	ENSG00000268401.2			1.442039073	5.06193E-07	2.39214E-06
3286	ENSG00000228727.9	SAPCD1	401251	1.418560213	5.06569E-07	2.39363E-06
3287	ENSG00000258609.3	LINC-ROR	100885779	1.939318525	5.09754E-07	2.40811E-06
3288	ENSG00000217644.5			1.28027954	5.15862E-07	2.43551E-06
3289	ENSG00000213221.5	DNLZ	728489	1.084941834	5.19858E-07	2.45262E-06
3290	ENSG00000159164.10	SV2A	9900	1.145379281	5.22441E-07	2.46393E-06
3291	ENSG00000167080.9	B4GALNT2	124872	1.980697757	5.26277E-07	2.48143E-06
3292	ENSG00000174992.8	ZG16	653808	2.502458194	5.32607E-07	2.51038E-06
3293	ENSG00000279080.1			1.093853427	5.33858E-07	2.51598E-06
3294	ENSG00000149927.18	DOC2A	8448	1.529051213	5.35311E-07	2.52223E-06
3295	ENSG00000233355.7	CHRM3-AS2	100506915	1.254389698	5.3658E-07	2.5279E-06
3296	ENSG00000237886.1	NALT1		1.401443657	5.41716E-07	2.55029E-06
3297	ENSG00000270257.1			1.320923987	5.43405E-07	2.55702E-06
3298	ENSG00000286563.1			1.53810859	5.49005E-07	2.58123E-06
3299	ENSG00000243368.2	MCCC1-AS1		1.365035616	5.51516E-07	2.59205E-06
3300	ENSG00000226521.7			2.152347285	5.61411E-07	2.63674E-06
3301	ENSG00000243276.6		105374059	2.466514385	5.6341E-07	2.64488E-06
3302	ENSG00000237977.1	EIF4HP2		1.026682045	5.66498E-07	2.65874E-06
3303	ENSG00000224167.2			2.250050094	5.70226E-07	2.67498E-06
3304	ENSG00000260958.3		105371200	3.275636645	5.84028E-07	2.73778E-06
3305	ENSG00000174482.10	LINGO2	158038	2.085965817	5.84817E-07	2.74115E-06
3306	ENSG00000233072.1	RPS15AP6		1.885059142	5.86061E-07	2.74601E-06
3307	ENSG00000285730.1			1.643423623	5.87226E-07	2.75114E-06
3308	ENSG00000287963.1			2.247013383	5.88616E-07	2.75733E-06
3309	ENSG00000265678.1			1.101967061	5.96323E-07	2.79013E-06
3310	ENSG00000188525.4			2.490383579	5.98791E-07	2.80102E-06
3311	ENSG00000093100.13			1.47610333	6.01591E-07	2.81353E-06
3312	ENSG00000089199.10	CHGB	1114	2.12528805	6.04072E-07	2.82473E-06
3313	ENSG00000256282.1	MTCH1P2		1.079573296	6.06528E-07	2.83554E-06
3314	ENSG00000286045.1			1.071424495	6.0792E-07	2.84105E-06
3315	ENSG00000254221.2	PCDHGB1	56104	1.580288355	6.15662E-07	2.87587E-06
3316	ENSG00000285518.1		124903342	1.595892868	6.16152E-07	2.87782E-06
3317	ENSG00000233360.4	PDXP-DT	101927051	1.353770419	6.18161E-07	2.88686E-06
3318	ENSG00000272243.6		105377858	2.566498278	6.18532E-07	2.88791E-06
3319	ENSG00000224308.1	LINC01527	101927988	1.733099097	6.20034E-07	2.89425E-06
3320	ENSG00000249816.7	LINC00964	157381	1.128324467	6.21971E-07	2.9026E-06
3321	ENSG00000227945.1	PTPRK-AS1		1.84447271	6.22254E-07	2.90358E-06
3322	ENSG00000173237.4	C11orf86	254439	2.575326004	6.23319E-07	2.90821E-06
3323	ENSG00000109072.14	VTN	7448	1.28236813	6.25469E-07	2.9179E-06
3324	ENSG00000274835.1			2.653826104	6.27994E-07	2.9283E-06
3325	ENSG00000123584.7	MAGEA9	4108	5.29565644	6.32239E-07	2.9474E-06
3326	ENSG00000282933.2	RHOXF1P3		1.721066235	6.33969E-07	2.95512E-06
3327	ENSG00000224020.1	MIR181A2HG	100379345	1.203365228	6.36132E-07	2.9638E-06
3328	ENSG00000275714.2	H3C1	8350	1.227998675	6.4365E-07	2.99778E-06
3329	ENSG00000249626.1	PPP4R2P4		1.145999783	6.4589E-07	3.00715E-06
3330	ENSG00000171540.8	OTP	23440	2.462396136	6.47221E-07	3.01299E-06
3331	ENSG00000254726.3	MEX3A	92312	1.026490396	6.49583E-07	3.023E-06
3332	ENSG00000249345.8	LINC02405	101927592	2.209103695	6.496E-07	3.023E-06
3333	ENSG00000270240.2		105371745	1.985137773	6.54019E-07	3.04286E-06
3334	ENSG00000238133.6	MAP3K20-AS1	339751	1.03366728	6.56126E-07	3.05158E-06
3335	ENSG00000145451.13	GLRA3	8001	1.715319845	6.58265E-07	3.06046E-06
3336	ENSG00000248475.5			4.233848269	6.58491E-07	3.06114E-06
3337	ENSG00000253868.4	FER1L6-AS2	157376	3.505431013	6.6009E-07	3.06786E-06
3338	ENSG00000241360.2	PDXP	57026	1.155654032	6.72949E-07	3.12506E-06
3339	ENSG00000235683.1			1.869422778	6.75234E-07	3.1353E-06
3340	ENSG00000277247.1			2.345324036	6.85632E-07	3.18135E-06
3341	ENSG00000234861.2	ATP5F1AP1		2.716207226	6.90929E-07	3.2048E-06
3342	ENSG00000266473.1	HELZ-AS1		1.3776057	6.98936E-07	3.23967E-06
3343	ENSG00000236279.7	CLEC2L	154790	2.265501844	7.03576E-07	3.25927E-06
3344	ENSG00000280311.1			1.536103762	7.0424E-07	3.2612E-06
3345	ENSG00000212421.1			2.119115973	7.06194E-07	3.2691E-06
3346	ENSG00000158525.16	CPA5	93979	1.149029415	7.15416E-07	3.30832E-06
3347	ENSG00000100344.11	PNPLA3	80339	1.171207601	7.21603E-07	3.33576E-06
3348	ENSG00000215156.5		124906495	1.450391382	7.22329E-07	3.33857E-06
3349	ENSG00000214513.4	NOTO	344022	2.423928052	7.24085E-07	3.34607E-06
3350	ENSG00000223662.1	SAMSN1-AS1	100874190	2.054980391	7.26021E-07	3.35384E-06
3351	ENSG00000115155.19	OTOF	9381	1.349815592	7.28452E-07	3.36468E-06
3352	ENSG00000225231.2	LINC02470		2.476924232	7.38939E-07	3.41113E-06
3353	ENSG00000091583.11	APOH	350	3.10910942	7.41958E-07	3.42307E-06
3354	ENSG00000243810.1	CFAP61-AS1		2.388102115	7.45592E-07	3.43824E-06
3355	ENSG00000181577.16	LINC03040		1.554333648	7.47367E-07	3.44602E-06
3356	ENSG00000267665.2			2.273032441	7.57977E-07	3.4921E-06
3357	ENSG00000221852.5	KRTAP1-5	83895	1.870256597	7.64758E-07	3.5217E-06
3358	ENSG00000259015.1			1.006882923	7.67588E-07	3.53392E-06
3359	ENSG00000264067.1			2.539053518	7.723E-07	3.55437E-06
3360	ENSG00000198754.5	OXCT2	64064	1.22482485	7.72465E-07	3.55472E-06
3361	ENSG00000112333.12	NR2E1	7101	1.816362934	7.78042E-07	3.57872E-06
3362	ENSG00000285897.1			1.398297719	7.79245E-07	3.58384E-06
3363	ENSG00000175513.9	TSGA10IP	254187	1.890134251	7.81152E-07	3.59136E-06
3364	ENSG00000254206.5	NPIPB11	728888	1.045082129	7.91483E-07	3.63717E-06
3365	ENSG00000257642.1	C12orf75-AS1	105369954	2.155144047	7.92918E-07	3.64334E-06
3366	ENSG00000266718.2			1.380594173	7.95487E-07	3.6543E-06
3367	ENSG00000283761.1			1.067084182	7.96497E-07	3.65851E-06
3368	ENSG00000168843.14	FSTL5	56884	2.747959843	7.97076E-07	3.66075E-06
3369	ENSG00000286418.1			1.693782122	8.01565E-07	3.67881E-06
3370	ENSG00000243225.2			1.906879468	8.05511E-07	3.69521E-06
3371	ENSG00000178997.11	EXD1	161829	1.576208396	8.11556E-07	3.72036E-06
3372	ENSG00000237605.2	DYRK3-AS1	105372876	1.046314581	8.12993E-07	3.72608E-06
3373	ENSG00000228948.2	SLC25A6P5		2.153426528	8.13735E-07	3.72837E-06
3374	ENSG00000223760.4	MED15P9		3.86300453	8.19099E-07	3.75103E-06
3375	ENSG00000236039.3	LINC02889	101927630	1.362747761	8.23941E-07	3.77103E-06
3376	ENSG00000257231.1	DYNLL1P4		2.019169222	8.26562E-07	3.78259E-06
3377	ENSG00000227879.2	PSPC1P1		1.261068924	8.27285E-07	3.78502E-06
3378	ENSG00000237775.1	DDR1-DT		1.930046817	8.41567E-07	3.84593E-06
3379	ENSG00000233190.1	RPS24P13		2.093434966	8.44732E-07	3.85906E-06
3380	ENSG00000275132.1	RN7SL663P		1.653653578	8.49234E-07	3.8765E-06
3381	ENSG00000239941.1	LINC02917		1.887821842	8.5024E-07	3.8802E-06
3382	ENSG00000164933.12	SLC25A32	81034	1.162703393	8.70993E-07	3.97034E-06
3383	ENSG00000180638.19	SLC47A2	146802	1.283202263	8.73567E-07	3.98116E-06
3384	ENSG00000091129.21	NRCAM	4897	1.391314373	8.84299E-07	4.02683E-06
3385	ENSG00000105605.7	CACNG7	59284	2.345011575	8.85611E-07	4.03234E-06
3386	ENSG00000228278.5	ORM2	5005	2.005943666	8.88158E-07	4.04254E-06
3387	ENSG00000271581.1			1.282511507	8.96435E-07	4.07834E-06
3388	ENSG00000121895.8	TMEM156	80008	1.096523751	9.10866E-07	4.13972E-06
3389	ENSG00000228663.1	PSMD10P1		1.320407253	9.19073E-07	4.17629E-06
3390	ENSG00000229190.1			1.813886977	9.19123E-07	4.17629E-06
3391	ENSG00000270000.1			1.088729549	9.24406E-07	4.19911E-06
3392	ENSG00000242221.9	PSG2	5670	2.414878301	9.25358E-07	4.20269E-06
3393	ENSG00000224387.1			1.405560948	9.33241E-07	4.23704E-06
3394	ENSG00000259444.1			2.145777518	9.40769E-07	4.26877E-06
3395	ENSG00000288560.1			2.241136192	9.44186E-07	4.28232E-06
3396	ENSG00000287731.1			2.238913668	9.47849E-07	4.29795E-06
3397	ENSG00000187556.7	NANOS3	342977	1.034127259	9.75493E-07	4.41623E-06
3398	ENSG00000247193.3		439933	2.14675228	9.75743E-07	4.41686E-06
3399	ENSG00000272669.1			1.078582756	9.78578E-07	4.42817E-06
3400	ENSG00000288538.1			1.11315216	9.89417E-07	4.47466E-06
3401	ENSG00000218996.1	ARL4AP5		1.307630035	9.90116E-07	4.47732E-06
3402	ENSG00000276337.1			1.088514339	9.93084E-07	4.49022E-06
3403	ENSG00000234753.5	FOXP4-AS1		1.406870069	1.00486E-06	4.5419E-06
3404	ENSG00000227262.3	HCG4B		1.188079093	1.03008E-06	4.65114E-06
3405	ENSG00000258301.3			1.412474735	1.03304E-06	4.66231E-06
3406	ENSG00000225603.3			1.823712833	1.03314E-06	4.66231E-06
3407	ENSG00000268746.1			1.277609598	1.03677E-06	4.67614E-06
3408	ENSG00000242880.2			1.61140761	1.04186E-06	4.69791E-06
3409	ENSG00000269009.1	SLC6A21P		3.636445507	1.04744E-06	4.72199E-06
3410	ENSG00000241743.4	XACT		1.571205564	1.04943E-06	4.72994E-06
3411	ENSG00000275491.1	LINC01730		1.253514639	1.05608E-06	4.75826E-06
3412	ENSG00000285987.1			2.471649361	1.05924E-06	4.7714E-06
3413	ENSG00000236053.1	LINC01067		2.047620952	1.06128E-06	4.7795E-06
3414	ENSG00000185640.6	KRT79	338785	1.802071826	1.06218E-06	4.78302E-06
3415	ENSG00000204149.13	AGAP6	414189	1.064804662	1.06617E-06	4.80043E-06
3416	ENSG00000287470.1		105372802	1.409987812	1.07028E-06	4.81839E-06
3417	ENSG00000286850.1			2.05754553	1.07288E-06	4.82788E-06
3418	ENSG00000265612.1	MIR4539	100616374	2.342084349	1.08454E-06	4.87647E-06
3419	ENSG00000156269.5	NAA11	84779	3.832417265	1.08613E-06	4.88305E-06
3420	ENSG00000275038.2			3.148070368	1.08624E-06	4.88305E-06
3421	ENSG00000273802.2	H2BC8	8339	1.027605812	1.11047E-06	4.988E-06
3422	ENSG00000230006.7	ANKRD36BP2		1.465687627	1.11675E-06	5.0145E-06
3423	ENSG00000259531.2			1.536039627	1.1191E-06	5.02392E-06
3424	ENSG00000119608.14	PROX2	283571	1.028857765	1.12127E-06	5.03309E-06
3425	ENSG00000272002.1	LINC02973		1.518344687	1.12219E-06	5.03605E-06
3426	ENSG00000272702.1			1.141774549	1.12707E-06	5.05682E-06
3427	ENSG00000240996.1			1.29120219	1.13112E-06	5.07386E-06
3428	ENSG00000258483.4	LINC02251	105371008	2.310277076	1.13943E-06	5.10938E-06
3429	ENSG00000224245.1			2.138171317	1.14956E-06	5.15249E-06
3430	ENSG00000272834.1			1.263337691	1.15284E-06	5.16299E-06
3431	ENSG00000131885.17	KRT17P1		1.163938632	1.15293E-06	5.16299E-06
3432	ENSG00000259611.2	LINC01582	101927332	2.7303429	1.1574E-06	5.18175E-06
3433	ENSG00000249047.2	COX6B1P5		1.696163201	1.16091E-06	5.19692E-06
3434	ENSG00000104267.10	CA2	760	1.211688625	1.187E-06	5.30829E-06
3435	ENSG00000277382.1			1.920904369	1.19095E-06	5.32416E-06
3436	ENSG00000234261.4			1.345456278	1.19787E-06	5.35149E-06
3437	ENSG00000238040.1	SALL4P2		3.556075242	1.20828E-06	5.39493E-06
3438	ENSG00000185332.8	TMEM105	284186	1.093861461	1.21154E-06	5.40828E-06
3439	ENSG00000251455.1			1.060588788	1.21652E-06	5.42928E-06
3440	ENSG00000197249.14	SERPINA1	5265	1.041093722	1.22879E-06	5.48156E-06
3441	ENSG00000231613.1			1.749603979	1.23441E-06	5.50388E-06
3442	ENSG00000174871.11	CNIH2	254263	1.080730158	1.23521E-06	5.50588E-06
3443	ENSG00000279365.1			1.453370613	1.237E-06	5.51263E-06
3444	ENSG00000215866.8	LINC01356	100996702	1.541764809	1.24296E-06	5.53793E-06
3445	ENSG00000266976.3	ERVE-5	102724908	2.824445177	1.24514E-06	5.54579E-06
3446	ENSG00000251138.7	LINC02882	100507377	3.343707383	1.24536E-06	5.54612E-06
3447	ENSG00000255050.1			1.229996527	1.24845E-06	5.55927E-06
3448	ENSG00000211818.1	TRAV39		1.592770643	1.25948E-06	5.60649E-06
3449	ENSG00000234537.1			2.276370102	1.26079E-06	5.61172E-06
3450	ENSG00000189229.11		105376944	1.44487296	1.26166E-06	5.61494E-06
3451	ENSG00000258646.1			1.317797129	1.26692E-06	5.63646E-06
3452	ENSG00000255129.6	TTC12-DT		1.311313122	1.27381E-06	5.6633E-06
3453	ENSG00000249771.2			1.475298512	1.27516E-06	5.66865E-06
3454	ENSG00000232678.2	SPTLC1P4		2.130077697	1.27817E-06	5.67951E-06
3455	ENSG00000104941.8	RSPH6A	81492	2.602932251	1.29096E-06	5.73375E-06
3456	ENSG00000253476.1			1.075096376	1.3091E-06	5.80979E-06
3457	ENSG00000276399.1	FLJ36000	284124	4.257475174	1.31982E-06	5.85474E-06
3458	ENSG00000233077.1	LINC01271	101927586	1.737753752	1.32241E-06	5.86555E-06
3459	ENSG00000182196.13	ARL6IP4	51329	1.045712621	1.34684E-06	5.96592E-06
3460	ENSG00000285822.1			4.99949073	1.34888E-06	5.97361E-06
3461	ENSG00000254207.1			1.837753184	1.3614E-06	6.02706E-06
3462	ENSG00000199691.1	RN7SKP173		1.657651208	1.36612E-06	6.04591E-06
3463	ENSG00000247134.7			1.361711655	1.36729E-06	6.04972E-06
3464	ENSG00000272382.1			1.000450457	1.37088E-06	6.06493E-06
3465	ENSG00000164076.17	CAMKV	79012	1.541793369	1.37524E-06	6.08055E-06
3466	ENSG00000285888.1			1.628518294	1.37694E-06	6.08565E-06
3467	ENSG00000143627.19	PKLR	5313	1.694247989	1.37967E-06	6.09497E-06
3468	ENSG00000164299.7	SPZ1	84654	3.558134679	1.38852E-06	6.13136E-06
3469	ENSG00000201820.1	Y_RNA		1.527557996	1.39438E-06	6.1538E-06
3470	ENSG00000255051.1	BCAS2P1		2.74415946	1.39718E-06	6.16536E-06
3471	ENSG00000237398.1	HLA-DPA3		1.755629251	1.39759E-06	6.16589E-06
3472	ENSG00000120217.14	CD274	29126	1.118125275	1.40081E-06	6.17876E-06
3473	ENSG00000184774.10	MGAT4EP		1.552938755	1.41265E-06	6.22822E-06
3474	ENSG00000267374.2	MIR924HG	647946	1.118436075	1.41364E-06	6.23187E-06
3475	ENSG00000254593.1	OR7E126P		1.248694819	1.42316E-06	6.27106E-06
3476	ENSG00000147231.14	RADX	55086	1.011738709	1.42851E-06	6.29322E-06
3477	ENSG00000258344.1			1.549812891	1.42885E-06	6.294E-06
3478	ENSG00000237661.1	TNIP2P1		2.350322445	1.43539E-06	6.32142E-06
3479	ENSG00000211956.2	IGHV4-34		1.745248714	1.43565E-06	6.32189E-06
3480	ENSG00000172031.7	EPHX4	253152	1.056912889	1.46013E-06	6.42166E-06
3481	ENSG00000142513.6	ACP4	93650	1.213859479	1.46026E-06	6.42166E-06
3482	ENSG00000253499.2			3.283843994	1.47E-06	6.46379E-06
3483	ENSG00000237716.2			3.345738514	1.48037E-06	6.5058E-06
3484	ENSG00000223969.6			1.395609766	1.48714E-06	6.53408E-06
3485	ENSG00000233397.3			1.998581493	1.50479E-06	6.60722E-06
3486	ENSG00000203877.8	RIPPLY2	134701	2.998307374	1.50625E-06	6.61218E-06
3487	ENSG00000287532.1			1.874283171	1.5154E-06	6.64614E-06
3488	ENSG00000276691.1			1.651994413	1.5208E-06	6.66794E-06
3489	ENSG00000163736.4	PPBP	5473	1.732133743	1.5218E-06	6.6716E-06
3490	ENSG00000231924.10	PSG1	5669	2.268005873	1.53479E-06	6.72555E-06
3491	ENSG00000229727.7		100506274	1.439564455	1.53993E-06	6.74586E-06
3492	ENSG00000164929.17	BAALC	79870	1.426179067	1.54067E-06	6.74833E-06
3493	ENSG00000268938.2			1.231663141	1.54935E-06	6.78412E-06
3494	ENSG00000267662.1	TSHZ3-AS1		1.823374109	1.5796E-06	6.91124E-06
3495	ENSG00000242599.7	CSAG4		4.042127479	1.59304E-06	6.9654E-06
3496	ENSG00000178795.9	GDPD4	220032	1.493041921	1.59646E-06	6.97757E-06
3497	ENSG00000152377.14	SPOCK1	6695	1.304413362	1.60885E-06	7.02759E-06
3498	ENSG00000285737.1	LINC02680		1.182234418	1.61085E-06	7.03553E-06
3499	ENSG00000230356.1	NCAPD2P1		1.976519992	1.61441E-06	7.04876E-06
3500	ENSG00000287181.1		124900731	2.121399488	1.61677E-06	7.0583E-06
3501	ENSG00000279807.1			1.574217173	1.64984E-06	7.19791E-06
3502	ENSG00000224794.2			2.38177463	1.65189E-06	7.20603E-06
3503	ENSG00000169851.15	PCDH7	5099	1.097678181	1.68468E-06	7.34263E-06
3504	ENSG00000272282.1	LINC02084		1.05909876	1.70773E-06	7.43898E-06
3505	ENSG00000259895.1	TEDC2-AS1	729652	1.775773539	1.71128E-06	7.45201E-06
3506	ENSG00000099869.8	IGF2-AS	51214	1.457394958	1.7115E-06	7.45215E-06
3507	ENSG00000283232.1	CYP2C23P		2.360388113	1.71598E-06	7.47084E-06
3508	ENSG00000236973.2	GAPDHP51		2.335576567	1.73896E-06	7.56671E-06
3509	ENSG00000180730.5	SHISA2	387914	1.388483091	1.74615E-06	7.59553E-06
3510	ENSG00000284512.1			1.128212491	1.76542E-06	7.67427E-06
3511	ENSG00000258815.1	LINC02820		2.033187405	1.77559E-06	7.71597E-06
3512	ENSG00000223387.6	LINC02068	105374219	1.284389973	1.78907E-06	7.76689E-06
3513	ENSG00000099985.4	OSM	5008	1.151195043	1.79059E-06	7.7726E-06
3514	ENSG00000202441.2	RNY4P10		1.037126799	1.81685E-06	7.87713E-06
3515	ENSG00000166523.8	CLEC4E	26253	1.078387464	1.81836E-06	7.88196E-06
3516	ENSG00000229637.4	PRAC2	360205	3.305258325	1.83362E-06	7.9446E-06
3517	ENSG00000188282.12	RUFY4	285180	1.229782332	1.84872E-06	8.0048E-06
3518	ENSG00000225106.1	CDH4-AS1	139355210	2.104150312	1.8585E-06	8.04277E-06
3519	ENSG00000125816.5	NKX2-4	644524	4.929403551	1.86899E-06	8.0855E-06
3520	ENSG00000155754.15	C2CD6	151254	1.446662948	1.89367E-06	8.17978E-06
3521	ENSG00000225913.2			2.010591223	1.90043E-06	8.20719E-06
3522	ENSG00000244157.1	EIF4E2P2		2.687600082	1.90106E-06	8.20903E-06
3523	ENSG00000218809.1			2.206207128	1.90746E-06	8.23576E-06
3524	ENSG00000255666.7	LINC02700		1.962212686	1.92042E-06	8.28721E-06
3525	ENSG00000229298.1	TUBB8P1		1.310425745	1.94247E-06	8.37959E-06
3526	ENSG00000227589.1	TP73-AS3		2.13924604	1.94568E-06	8.39255E-06
3527	ENSG00000256115.6	LINC02443		1.579706685	1.94779E-06	8.40074E-06
3528	ENSG00000228235.1			2.845369814	1.96059E-06	8.45225E-06
3529	ENSG00000279379.1			3.624919396	1.96649E-06	8.47491E-06
3530	ENSG00000213197.3	NUDCP1		1.399186783	1.96767E-06	8.4791E-06
3531	ENSG00000167371.21	PRRT2	112476	1.013895071	1.97199E-06	8.4968E-06
3532	ENSG00000170961.7	HAS2	3037	1.168681051	1.97423E-06	8.50551E-06
3533	ENSG00000260504.1			2.534326533	1.97645E-06	8.51416E-06
3534	ENSG00000273287.2			1.624446372	2.01723E-06	8.6804E-06
3535	ENSG00000206557.6	TRIM71	131405	1.775831941	2.01817E-06	8.68348E-06
3536	ENSG00000268940.5	CT45A1	541466	4.847261445	2.01932E-06	8.68748E-06
3537	ENSG00000233122.2	CTAGE7P		1.013315891	2.06082E-06	8.86125E-06
3538	ENSG00000267892.1			1.112250711	2.06328E-06	8.86987E-06
3539	ENSG00000232197.3			2.864440127	2.06861E-06	8.89184E-06
3540	ENSG00000226806.1	LCT-AS1	100507600	1.518441644	2.07843E-06	8.93018E-06
3541	ENSG00000272192.1	PDE7A-DT		1.127075532	2.09011E-06	8.97937E-06
3542	ENSG00000271267.1			1.262682468	2.11431E-06	9.07943E-06
3543	ENSG00000114200.10	BCHE	590	1.697464615	2.11678E-06	9.08904E-06
3544	ENSG00000113249.13	HAVCR1	26762	1.835448498	2.12105E-06	9.10639E-06
3545	ENSG00000226043.2			2.998616105	2.12468E-06	9.12099E-06
3546	ENSG00000241678.1	RPL21P94		1.812155826	2.12684E-06	9.12926E-06
3547	ENSG00000115850.10	LCT	3938	1.811219652	2.13133E-06	9.14757E-06
3548	ENSG00000281566.3	LINC03077		4.221021209	2.13374E-06	9.15689E-06
3549	ENSG00000274471.2			1.015459484	2.15742E-06	9.25753E-06
3550	ENSG00000255079.1	LINC02704	105376647	3.116258308	2.16668E-06	9.29426E-06
3551	ENSG00000152034.11	MCHR2	84539	2.828028003	2.16928E-06	9.30239E-06
3552	ENSG00000217646.1	H2BC16P		2.053752139	2.16964E-06	9.30264E-06
3553	ENSG00000285473.1			2.371079022	2.17649E-06	9.33027E-06
3554	ENSG00000286004.1			1.018780373	2.17704E-06	9.33164E-06
3555	ENSG00000168754.15	FAM178B	51252	1.495865008	2.20219E-06	9.43228E-06
3556	ENSG00000279516.2	FAM230C	26080	4.418568405	2.20256E-06	9.43284E-06
3557	ENSG00000271615.1	ACTG1P22		2.020146875	2.21003E-06	9.46176E-06
3558	ENSG00000256037.1	MRPL40P1		1.515226027	2.30137E-06	9.83902E-06
3559	ENSG00000196406.4	SPANXD	64648	4.415385818	2.31322E-06	9.88646E-06
3560	ENSG00000257894.2	SYT1-AS1		1.187889335	2.31649E-06	9.8983E-06
3561	ENSG00000278705.1	H4C2	8366	1.694582929	2.33483E-06	9.97346E-06
3562	ENSG00000231291.3			1.594484908	2.33993E-06	9.99307E-06
3563	ENSG00000154545.16	MAGED4	728239	1.988900666	2.35971E-06	1.00721E-05
3564	ENSG00000255143.1			2.602830468	2.36826E-06	1.01065E-05
3565	ENSG00000270130.1			1.292635354	2.37042E-06	1.01146E-05
3566	ENSG00000175894.18	TSPEAR	54084	1.739790816	2.37409E-06	1.01292E-05
3567	ENSG00000263235.1			1.041503243	2.39377E-06	1.02043E-05
3568	ENSG00000228784.9	LINC00954	400946	1.16330634	2.42917E-06	1.03497E-05
3569	ENSG00000279161.1			1.837171512	2.43452E-06	1.03691E-05
3570	ENSG00000151632.17	AKR1C2	1646	1.552361715	2.45806E-06	1.04649E-05
3571	ENSG00000260088.1	DDX59-AS1	101929224	1.164933762	2.47259E-06	1.05256E-05
3572	ENSG00000228040.1			1.936800873	2.481E-06	1.05603E-05
3573	ENSG00000266171.1			1.241344132	2.50798E-06	1.06661E-05
3574	ENSG00000267650.1			1.974314514	2.51243E-06	1.06826E-05
3575	ENSG00000288056.1			1.302127425	2.55426E-06	1.08512E-05
3576	ENSG00000260953.1			1.845449035	2.55838E-06	1.08675E-05
3577	ENSG00000260394.2	STUB1-DT		1.683700047	2.57836E-06	1.09477E-05
3578	ENSG00000286240.1			1.425484373	2.58117E-06	1.09572E-05
3579	ENSG00000263923.1			2.480548661	2.58882E-06	1.09838E-05
3580	ENSG00000055732.13	MCOLN3	55283	1.166117925	2.60332E-06	1.10419E-05
3581	ENSG00000238390.1		124900469	1.470580782	2.6086E-06	1.10631E-05
3582	ENSG00000235034.7	C19orf81	342918	2.193983386	2.61511E-06	1.10895E-05
3583	ENSG00000234405.2			1.249018373	2.61747E-06	1.10971E-05
3584	ENSG00000286362.1			1.459578063	2.6196E-06	1.11049E-05
3585	ENSG00000234115.2	IDH1P1		2.21735758	2.62454E-06	1.11247E-05
3586	ENSG00000186844.6	LCE1A	353131	2.69876656	2.63388E-06	1.11631E-05
3587	ENSG00000255477.1			2.95453756	2.64454E-06	1.12047E-05
3588	ENSG00000229563.7	LINC01204	101927528	1.922817441	2.65335E-06	1.12396E-05
3589	ENSG00000277957.1	SENP3-EIF4A1		1.299795075	2.65632E-06	1.1251E-05
3590	ENSG00000280543.1	ASAP1-IT2	100507117	1.132794838	2.65843E-06	1.12575E-05
3591	ENSG00000166473.17	PKD1L2	114780	1.471703992	2.66986E-06	1.13023E-05
3592	ENSG00000186543.7	CROCCP5		2.265127929	2.67839E-06	1.13323E-05
3593	ENSG00000250770.3			1.43042868	2.6968E-06	1.1403E-05
3594	ENSG00000226935.6	LINC00161	118421	2.221610541	2.71619E-06	1.14813E-05
3595	ENSG00000023839.12	ABCC2	1244	1.10733038	2.74897E-06	1.16136E-05
3596	ENSG00000271021.1			1.844538483	2.76292E-06	1.16688E-05
3597	ENSG00000272662.1	TIMMDC1-DT		1.318797224	2.76375E-06	1.16711E-05
3598	ENSG00000254632.2			2.064137003	2.77607E-06	1.17151E-05
3599	ENSG00000278811.4	LINC00624	100289211	1.0209919	2.78062E-06	1.17311E-05
3600	ENSG00000178772.7	CPN2	1370	1.581929456	2.78253E-06	1.17366E-05
3601	ENSG00000184985.16	SORCS2	57537	1.091589702	2.79687E-06	1.17896E-05
3602	ENSG00000169894.18	MUC3A	4584	1.344151689	2.80565E-06	1.18241E-05
3603	ENSG00000265417.1			2.792706826	2.82113E-06	1.18856E-05
3604	ENSG00000267108.1			1.861012995	2.85052E-06	1.2003E-05
3605	ENSG00000273313.1	RBAKDN		2.035969115	2.89237E-06	1.21689E-05
3606	ENSG00000230262.8	LINC02603	158257	1.251388172	2.922E-06	1.22818E-05
3607	ENSG00000228221.6	LINC00578	100505566	1.038949147	2.93475E-06	1.23315E-05
3608	ENSG00000224426.1	SLC31A1P1		1.77830639	2.98688E-06	1.25372E-05
3609	ENSG00000279357.1			1.744131044	2.99312E-06	1.25608E-05
3610	ENSG00000231310.3	TBL1XR1-AS1		1.903010303	3.0053E-06	1.26092E-05
3611	ENSG00000172650.14	AGAP5	729092	1.693602787	3.03825E-06	1.27407E-05
3612	ENSG00000248544.2			1.433232881	3.05912E-06	1.28228E-05
3613	ENSG00000259404.5	EFL1P1		1.49943355	3.0751E-06	1.2885E-05
3614	ENSG00000278861.1			1.17228952	3.1129E-06	1.30358E-05
3615	ENSG00000265194.1			1.15033526	3.11752E-06	1.30538E-05
3616	ENSG00000261357.1			1.271781632	3.14969E-06	1.3176E-05
3617	ENSG00000283876.1	MIR4260	100422894	2.050399027	3.18266E-06	1.33083E-05
3618	ENSG00000227619.3		105376292	1.396853014	3.19078E-06	1.3338E-05
3619	ENSG00000277310.1			1.084882469	3.2082E-06	1.34094E-05
3620	ENSG00000237525.7			2.756040386	3.21009E-06	1.34159E-05
3621	ENSG00000237025.1	PARD6BP1		1.699614213	3.23282E-06	1.35052E-05
3622	ENSG00000268287.1			1.321089695	3.25594E-06	1.35989E-05
3623	ENSG00000215417.13	MIR17HG	407975	1.006996185	3.28041E-06	1.36954E-05
3624	ENSG00000249505.1	JMJD4P1		2.921512342	3.3267E-06	1.38755E-05
3625	ENSG00000280129.1			1.023060935	3.34413E-06	1.39453E-05
3626	ENSG00000274191.1			1.262773753	3.35737E-06	1.39946E-05
3627	ENSG00000244712.1	RPL29P27		1.220442477	3.35793E-06	1.39954E-05
3628	ENSG00000168126.3	OR2W6P		1.994229646	3.38567E-06	1.41081E-05
3629	ENSG00000125848.10	FLRT3	23767	1.414378176	3.39849E-06	1.41585E-05
3630	ENSG00000237136.8	C4orf51	646603	2.260089548	3.40529E-06	1.41839E-05
3631	ENSG00000279781.1			1.074252127	3.40723E-06	1.41905E-05
3632	ENSG00000236124.1	HMGN2P23		1.841438709	3.41829E-06	1.4235E-05
3633	ENSG00000187243.16	MAGED4B	81557	1.716150844	3.42436E-06	1.42588E-05
3634	ENSG00000248360.7	LINC00504	201853	1.474836871	3.44861E-06	1.43538E-05
3635	ENSG00000224960.5	PPP4R3C	139420	4.944644132	3.45058E-06	1.4359E-05
3636	ENSG00000257191.1			2.031245924	3.46112E-06	1.43953E-05
3637	ENSG00000228061.7	ELP4-AS1		1.462151239	3.4783E-06	1.44637E-05
3638	ENSG00000233069.1			1.930612587	3.48123E-06	1.44743E-05
3639	ENSG00000184507.16	NUTM1	256646	1.872259233	3.48621E-06	1.44935E-05
3640	ENSG00000163958.14	ZDHHC19	131540	1.353678802	3.48729E-06	1.44965E-05
3641	ENSG00000288033.1			1.147110086	3.53494E-06	1.46823E-05
3642	ENSG00000264548.1			1.294890295	3.56141E-06	1.4786E-05
3643	ENSG00000211899.10	IGHM		1.73848991	3.56342E-06	1.47928E-05
3644	ENSG00000082074.19	FYB1	2533	1.023000097	3.5669E-06	1.48057E-05
3645	ENSG00000255399.4	TBX5-AS1	255480	2.413151582	3.5888E-06	1.48935E-05
3646	ENSG00000286271.2			1.62941109	3.58977E-06	1.48959E-05
3647	ENSG00000259438.1	MAPK6-DT	112543478	1.00254995	3.61581E-06	1.4993E-05
3648	ENSG00000112139.16	MDGA1	266727	1.034707486	3.62039E-06	1.50104E-05
3649	ENSG00000252680.1	RNA5SP449		1.77180576	3.64233E-06	1.50983E-05
3650	ENSG00000242876.3	RN7SL812P		1.891718096	3.64338E-06	1.5101E-05
3651	ENSG00000249790.3	LINC02972		2.076003602	3.65468E-06	1.51463E-05
3652	ENSG00000137261.14	KIAA0319	9856	1.620237456	3.66519E-06	1.51882E-05
3653	ENSG00000176273.15	SLC35G1	159371	1.051269061	3.67678E-06	1.52347E-05
3654	ENSG00000101307.16	SIRPB1	10326	1.113895741	3.68338E-06	1.52604E-05
3655	ENSG00000197238.5	H4C11	8363	1.037616941	3.6902E-06	1.52823E-05
3656	ENSG00000232727.2	YWHAEP1		1.605914368	3.69942E-06	1.53189E-05
3657	ENSG00000228664.1			2.689133258	3.71174E-06	1.53683E-05
3658	ENSG00000258240.6	LINC01404	105369980	2.626743135	3.71385E-06	1.53738E-05
3659	ENSG00000214376.6	VSTM5	387804	1.240911628	3.74578E-06	1.54914E-05
3660	ENSG00000272205.1	POU2F1-DT		1.06155077	3.77416E-06	1.56023E-05
3661	ENSG00000261218.5			1.357893059	3.7818E-06	1.5629E-05
3662	ENSG00000224713.4			1.56529195	3.79544E-06	1.56795E-05
3663	ENSG00000211673.2	IGLV3-1		1.663116434	3.79806E-06	1.5688E-05
3664	ENSG00000166106.3	ADAMTS15	170689	1.16932016	3.80485E-06	1.57115E-05
3665	ENSG00000156886.12	ITGAD	3681	1.186058919	3.82962E-06	1.58068E-05
3666	ENSG00000272235.1			1.297166341	3.85612E-06	1.59054E-05
3667	ENSG00000278881.2			1.494660435	3.87435E-06	1.59732E-05
3668	ENSG00000179774.9	ATOH7	220202	1.585808929	3.87776E-06	1.59856E-05
3669	ENSG00000123999.5	INHA	3623	1.168695318	3.94163E-06	1.62404E-05
3670	ENSG00000287421.1			1.839706686	3.94901E-06	1.62671E-05
3671	ENSG00000256312.1			1.828906368	4.01307E-06	1.65021E-05
3672	ENSG00000159224.4	GIP	2695	2.697067379	4.01346E-06	1.65021E-05
3673	ENSG00000258700.6	LINC00871	100506412	2.760964311	4.04819E-06	1.6632E-05
3674	ENSG00000197272.2	IL27	246778	1.226480641	4.05516E-06	1.66545E-05
3675	ENSG00000269978.1			1.765959443	4.05791E-06	1.66624E-05
3676	ENSG00000184148.4	SPRR4	163778	1.799281074	4.06021E-06	1.66701E-05
3677	ENSG00000258593.2			1.059081693	4.06111E-06	1.66721E-05
3678	ENSG00000237914.5	SIRPG-AS1	101929010	1.779986955	4.06715E-06	1.66934E-05
3679	ENSG00000241120.1	HMGN1P8		1.494075386	4.08912E-06	1.67819E-05
3680	ENSG00000261730.1	FOXF2-DT		1.123341442	4.0922E-06	1.6791E-05
3681	ENSG00000230450.1	NEK2P4		2.354091415	4.13106E-06	1.69417E-05
3682	ENSG00000237073.1			1.655832601	4.16465E-06	1.70671E-05
3683	ENSG00000179695.3	OR6C2	341416	2.82301875	4.18384E-06	1.71422E-05
3684	ENSG00000156103.16	MMP16	4325	1.08777069	4.19631E-06	1.71915E-05
3685	ENSG00000211973.2	IGHV1-69		1.941098012	4.22647E-06	1.73061E-05
3686	ENSG00000267258.1	PPIAP58		1.399298073	4.24339E-06	1.73701E-05
3687	ENSG00000213212.3	NCLP1		1.22931007	4.26172E-06	1.74379E-05
3688	ENSG00000261245.2			1.95053245	4.29752E-06	1.75771E-05
3689	ENSG00000274363.1			1.450708475	4.33419E-06	1.77161E-05
3690	ENSG00000244218.3	RN7SL81P		1.350632248	4.34003E-06	1.77363E-05
3691	ENSG00000262358.1			1.654454009	4.36165E-06	1.7821E-05
3692	ENSG00000136834.3	OR1J1	347168	1.921860511	4.3789E-06	1.7886E-05
3693	ENSG00000168676.11	KCTD19	146212	1.267578727	4.43579E-06	1.81108E-05
3694	ENSG00000175264.8	CHST1	8534	1.057041621	4.4378E-06	1.81172E-05
3695	ENSG00000038295.8	TLL1	7092	1.280424652	4.45536E-06	1.81814E-05
3696	ENSG00000244617.2	ASPRV1	151516	1.745257688	4.5465E-06	1.85285E-05
3697	ENSG00000240086.7		102724019	1.834172034	4.58637E-06	1.86718E-05
3698	ENSG00000257674.1			1.940604654	4.60245E-06	1.87315E-05
3699	ENSG00000205445.3	KRTAP10-2	386679	2.213596083	4.62074E-06	1.88021E-05
3700	ENSG00000182352.8	CD300LD-AS1	146723	2.807445931	4.66047E-06	1.8956E-05
3701	ENSG00000205238.9	SPDYE2	441273	1.262560641	4.66621E-06	1.89774E-05
3702	ENSG00000228016.1	RAPGEF4-AS1	91149	1.777007226	4.67928E-06	1.90247E-05
3703	ENSG00000253174.2			1.846186118	4.70772E-06	1.91325E-05
3704	ENSG00000254987.1	PDGFDDN		1.819471971	4.74702E-06	1.92863E-05
3705	ENSG00000286975.1			1.124327172	4.76592E-06	1.93611E-05
3706	ENSG00000243483.1	DNAJB1P2		2.673098438	4.77634E-06	1.94014E-05
3707	ENSG00000257452.1			1.763651377	4.78646E-06	1.94385E-05
3708	ENSG00000276509.1			1.729986187	4.80373E-06	1.95027E-05
3709	ENSG00000269959.1	SPACA6-AS1	102238594	1.533399518	4.8408E-06	1.96411E-05
3710	ENSG00000225720.6			1.137916088	4.8748E-06	1.9771E-05
3711	ENSG00000267838.2			1.032137226	4.90217E-06	1.98726E-05
3712	ENSG00000121743.4	GJA3	2700	1.081983419	4.92077E-06	1.99431E-05
3713	ENSG00000272973.1			2.346422315	4.94536E-06	2.00387E-05
3714	ENSG00000280408.1			1.618958476	4.98286E-06	2.01824E-05
3715	ENSG00000287600.1			1.128829306	5.01423E-06	2.03032E-05
3716	ENSG00000267056.2			1.870327974	5.03043E-06	2.03605E-05
3717	ENSG00000234191.1	LINC01283	107985711	1.883825581	5.04262E-06	2.04078E-05
3718	ENSG00000249619.1	HMGN1P13		1.889036409	5.06903E-06	2.05063E-05
3719	ENSG00000176998.4	HCG4		1.27826623	5.08987E-06	2.05801E-05
3720	ENSG00000272720.1			1.169292373	5.11123E-06	2.06601E-05
3721	ENSG00000256234.1	SSPN-AS1		1.600424922	5.11623E-06	2.06782E-05
3722	ENSG00000260498.6			1.622282988	5.11692E-06	2.06789E-05
3723	ENSG00000274505.1	Metazoa_SRP		1.742404851	5.12352E-06	2.07035E-05
3724	ENSG00000279488.1			1.500115693	5.1926E-06	2.09595E-05
3725	ENSG00000236337.1	FMR1-IT1		1.036355053	5.21415E-06	2.10439E-05
3726	ENSG00000262768.2			1.93268178	5.24276E-06	2.11551E-05
3727	ENSG00000272507.1	RNU6-88P		1.70974394	5.26004E-06	2.12227E-05
3728	ENSG00000233985.2	LINC01681		2.531341431	5.32099E-06	2.14642E-05
3729	ENSG00000283788.1	MIR936	100126326	2.032642486	5.33058E-06	2.15007E-05
3730	ENSG00000259717.1	LINC00677		1.886690745	5.33159E-06	2.15026E-05
3731	ENSG00000113889.14	KNG1	3827	2.246303238	5.35954E-06	2.16089E-05
3732	ENSG00000278342.1			2.476797353	5.35958E-06	2.16089E-05
3733	ENSG00000225173.1			1.511246591	5.45257E-06	2.19636E-05
3734	ENSG00000225465.9	RFPL1S	102723305	1.49325309	5.4534E-06	2.19636E-05
3735	ENSG00000271860.7		101927314	1.917242241	5.45366E-06	2.19636E-05
3736	ENSG00000276462.2	LINC03025	440896	2.101107363	5.47254E-06	2.20307E-05
3737	ENSG00000244184.1	PELP1-DT		1.165186449	5.49363E-06	2.21111E-05
3738	ENSG00000288086.1			2.409199579	5.50021E-06	2.21354E-05
3739	ENSG00000239311.1			5.02760664	5.52319E-06	2.22166E-05
3740	ENSG00000171855.7	IFNB1	3456	2.211744243	5.60433E-06	2.25247E-05
3741	ENSG00000228886.1			1.634246277	5.60645E-06	2.25309E-05
3742	ENSG00000258759.1			1.713653454	5.65241E-06	2.27039E-05
3743	ENSG00000187172.15	BAGE2		3.917829128	5.7021E-06	2.28852E-05
3744	ENSG00000259921.1			1.021467249	5.71827E-06	2.29454E-05
3745	ENSG00000146938.16	NLGN4X	57502	1.517602696	5.72503E-06	2.29679E-05
3746	ENSG00000228135.1			1.920878924	5.74246E-06	2.30331E-05
3747	ENSG00000285658.1			1.735826731	5.78167E-06	2.31717E-05
3748	ENSG00000255987.1	TOMM20P2		1.265023279	5.82442E-06	2.33335E-05
3749	ENSG00000248610.1	HSPA8P4		1.044457224	5.84629E-06	2.34094E-05
3750	ENSG00000287896.1			1.316807939	5.92908E-06	2.37265E-05
3751	ENSG00000274294.1			1.607656629	5.93652E-06	2.37515E-05
3752	ENSG00000225536.2	STIP1P3		1.300921678	5.95313E-06	2.38131E-05
3753	ENSG00000249388.3	FLJ12825	440101	1.796443031	5.96801E-06	2.3863E-05
3754	ENSG00000122592.8	HOXA7	3204	1.333613939	6.02207E-06	2.4067E-05
3755	ENSG00000232564.3	MRTFA-AS1	101927257	1.463397139	6.04507E-06	2.41541E-05
3756	ENSG00000197977.4	ELOVL2	54898	1.159617235	6.0696E-06	2.42448E-05
3757	ENSG00000286180.1			1.671523149	6.09592E-06	2.43474E-05
3758	ENSG00000160781.17	PAQR6	79957	1.000273312	6.13315E-06	2.44887E-05
3759	ENSG00000254585.5	MAGEL2	54551	1.312914639	6.16984E-06	2.46228E-05
3760	ENSG00000285437.1	POLR2J3	548644	1.022840657	6.19426E-06	2.47103E-05
3761	ENSG00000242520.5	MAGEA5P		2.52073821	6.21165E-06	2.47682E-05
3762	ENSG00000220256.4			2.061476226	6.22059E-06	2.47954E-05
3763	ENSG00000138696.11	BMPR1B	658	1.038981492	6.28151E-06	2.50332E-05
3764	ENSG00000259755.1	LRRK1-AS1		2.05864686	6.34555E-06	2.52707E-05
3765	ENSG00000256597.3	LINC02393	400087	2.762143149	6.3927E-06	2.54405E-05
3766	ENSG00000281880.2	PAUPAR	440034	2.517608053	6.40676E-06	2.54888E-05
3767	ENSG00000276916.1			1.065689438	6.4339E-06	2.55917E-05
3768	ENSG00000125878.6	TCF15	6939	1.477157474	6.49807E-06	2.58314E-05
3769	ENSG00000236871.7	LINC00106	751580	1.01711672	6.57437E-06	2.61268E-05
3770	ENSG00000124232.11	RBPJL	11317	1.579152993	6.58127E-06	2.61516E-05
3771	ENSG00000235892.1	PKMP2		1.671369007	6.60665E-06	2.62498E-05
3772	ENSG00000254283.1			3.403760106	6.63117E-06	2.63393E-05
3773	ENSG00000152128.13	TMEM163	81615	1.119333038	6.68023E-06	2.65209E-05
3774	ENSG00000230987.2			1.994769447	6.73226E-06	2.67034E-05
3775	ENSG00000254484.1			2.071505364	6.74698E-06	2.67591E-05
3776	ENSG00000170516.17	COX7B2	170712	5.448436911	6.76734E-06	2.68318E-05
3777	ENSG00000242522.1	KLHL6-AS1		2.259937055	6.8092E-06	2.69789E-05
3778	ENSG00000238168.5	IFITM3P5		1.594252233	6.83343E-06	2.70722E-05
3779	ENSG00000272226.1			1.074043071	6.87258E-06	2.72219E-05
3780	ENSG00000101198.15	NKAIN4	128414	1.518356687	6.91784E-06	2.73902E-05
3781	ENSG00000155428.12	TRIM74	378108	1.256919626	6.92475E-06	2.74148E-05
3782	ENSG00000175065.12	DSG4	147409	1.485623538	6.94094E-06	2.74734E-05
3783	ENSG00000271161.1	BOLA2P2		1.307973487	6.9685E-06	2.75687E-05
3784	ENSG00000189068.11	VSTM1	284415	1.563172636	7.0124E-06	2.77307E-05
3785	ENSG00000287322.1			1.659007007	7.05978E-06	2.79104E-05
3786	ENSG00000265683.1	SYPL1P2		1.396794643	7.15268E-06	2.82664E-05
3787	ENSG00000183822.3	NCF4-AS1	107985578	1.730044763	7.18151E-06	2.83718E-05
3788	ENSG00000231123.1	SPATA20P1		1.060664671	7.18389E-06	2.83784E-05
3789	ENSG00000215007.3	DNAJA1P3		1.12364813	7.24264E-06	2.85962E-05
3790	ENSG00000123496.8	IL13RA2	3598	1.510886308	7.26001E-06	2.86591E-05
3791	ENSG00000233081.1			1.040604128	7.26534E-06	2.86773E-05
3792	ENSG00000135519.8	KCNH3	23416	1.153136705	7.32937E-06	2.8907E-05
3793	ENSG00000270641.1	TSIX	9383	1.581350336	7.39158E-06	2.91408E-05
3794	ENSG00000259023.3	LINC00524		2.190239858	7.40502E-06	2.9185E-05
3795	ENSG00000279197.1			1.530627412	7.43779E-06	2.92996E-05
3796	ENSG00000230426.4	LINC01036	104169671	1.835788822	7.45111E-06	2.93463E-05
3797	ENSG00000159167.12	STC1	6781	1.020980906	7.50842E-06	2.95603E-05
3798	ENSG00000232334.1			1.227171346	7.53759E-06	2.96692E-05
3799	ENSG00000286179.1			1.618785408	7.65772E-06	3.01181E-05
3800	ENSG00000261089.1	CDC37P2		1.58885232	7.68269E-06	3.02074E-05
3801	ENSG00000226235.1	LEMD1-AS1	284576	1.575406544	7.69465E-06	3.02454E-05
3802	ENSG00000197084.5	LCE1C	353133	1.566491167	7.70234E-06	3.02666E-05
3803	ENSG00000253910.2	PCDHGB2	56103	1.17257751	7.70312E-06	3.02667E-05
3804	ENSG00000275936.1			1.583617068	7.70715E-06	3.02795E-05
3805	ENSG00000240265.1	H1-8P1		1.875839371	7.72459E-06	3.0342E-05
3806	ENSG00000147168.13	IL2RG	3561	1.013064728	7.72698E-06	3.03484E-05
3807	ENSG00000166707.12	ZCCHC18	644353	1.012768748	7.73295E-06	3.03688E-05
3808	ENSG00000130368.7	MAS1	4142	1.737463751	7.75621E-06	3.04565E-05
3809	ENSG00000200403.1	RNU6-1099P		1.625374492	7.80298E-06	3.06196E-05
3810	ENSG00000198910.14	L1CAM	3897	1.318749627	7.81834E-06	3.06768E-05
3811	ENSG00000254780.1			1.942254947	7.85269E-06	3.08024E-05
3812	ENSG00000224132.2			1.732590385	7.92202E-06	3.10652E-05
3813	ENSG00000180767.10	CHST13	166012	1.004791357	7.92988E-06	3.10929E-05
3814	ENSG00000257896.1			1.647153822	8.00954E-06	3.13929E-05
3815	ENSG00000279114.1			1.526858137	8.08001E-06	3.16628E-05
3816	ENSG00000157570.12	TSPAN18	90139	1.079494562	8.09971E-06	3.17306E-05
3817	ENSG00000267778.1	CBARP-DT		1.764149031	8.11148E-06	3.17736E-05
3818	ENSG00000271392.1			1.793163399	8.1542E-06	3.19283E-05
3819	ENSG00000004468.13	CD38	952	1.002243202	8.17804E-06	3.20185E-05
3820	ENSG00000206647.1		124906144	1.848595497	8.19676E-06	3.20886E-05
3821	ENSG00000231982.1	MCF2L2P1		1.25148791	8.21928E-06	3.21736E-05
3822	ENSG00000255152.8	MSH5-SAPCD1		1.139760673	8.22478E-06	3.21919E-05
3823	ENSG00000259129.6	LINC00648	100506433	2.637773913	8.22622E-06	3.21944E-05
3824	ENSG00000236559.1	BCL2L1-AS1	105372589	1.36624915	8.27213E-06	3.23549E-05
3825	ENSG00000253357.1			2.317484159	8.34549E-06	3.26161E-05
3826	ENSG00000228956.8	SATB1-AS1		1.025803413	8.3558E-06	3.26522E-05
3827	ENSG00000225728.2	TMEM230P1		1.739308675	8.36389E-06	3.26783E-05
3828	ENSG00000286112.1	KYAT1	883	1.46786663	8.38173E-06	3.27416E-05
3829	ENSG00000226194.5	LINC02519	105378147	1.383146438	8.38795E-06	3.27627E-05
3830	ENSG00000260423.2	LINC02367	101930452	1.113353076	8.39752E-06	3.27936E-05
3831	ENSG00000279339.1			1.21471191	8.40701E-06	3.28242E-05
3832	ENSG00000280081.4	LINC01667		3.774572231	8.4139E-06	3.28446E-05
3833	ENSG00000270781.1			1.007201368	8.43125E-06	3.29026E-05
3834	ENSG00000282317.1			1.194223716	8.50107E-06	3.31653E-05
3835	ENSG00000227438.1	COL6A2-DT		1.433997813	8.5321E-06	3.32765E-05
3836	ENSG00000129521.15	EGLN3	112399	1.007230506	8.55312E-06	3.33552E-05
3837	ENSG00000074047.22	GLI2	2736	1.218423292	8.57315E-06	3.34268E-05
3838	ENSG00000228415.3	PTMAP1		1.392423675	8.66888E-06	3.37768E-05
3839	ENSG00000264705.1			1.776488555	8.74545E-06	3.40684E-05
3840	ENSG00000188778.6	ADRB3	155	1.567280124	8.85761E-06	3.44816E-05
3841	ENSG00000198854.5	KPLCE	100129271	1.810598369	8.86384E-06	3.45025E-05
3842	ENSG00000187893.10	CXXC1P1		2.187254402	8.88011E-06	3.45557E-05
3843	ENSG00000232627.1			2.038148037	8.88582E-06	3.45745E-05
3844	ENSG00000227560.1	RPS15AP30		1.610523975	8.9086E-06	3.46529E-05
3845	ENSG00000227666.1	CYCSP24		1.339844134	8.93729E-06	3.47577E-05
3846	ENSG00000228869.1	COX4I1P2		1.783025923	8.99095E-06	3.49492E-05
3847	ENSG00000240720.9	LRRD1	401387	1.649361318	8.99515E-06	3.49587E-05
3848	ENSG00000230269.7	LINC02525		3.718230558	9.02445E-06	3.50691E-05
3849	ENSG00000274767.1			1.529237615	9.05536E-06	3.51755E-05
3850	ENSG00000235885.8	LINC01828	101927661	1.358773574	9.06342E-06	3.51999E-05
3851	ENSG00000271749.1			1.618113967	9.06887E-06	3.52105E-05
3852	ENSG00000229719.5	MIR194-2HG		1.580001863	9.11054E-06	3.53552E-05
3853	ENSG00000258086.1	GPR84-AS1		1.359518558	9.11999E-06	3.53884E-05
3854	ENSG00000229407.5			2.273126752	9.12284E-06	3.5396E-05
3855	ENSG00000266708.1	RAB12-AS1		1.084186948	9.15215E-06	3.54993E-05
3856	ENSG00000242252.2	BGLAP	632	1.15150733	9.17977E-06	3.55925E-05
3857	ENSG00000263126.1			1.051333621	9.21943E-06	3.57428E-05
3858	ENSG00000205086.8	LINC02898	400950	1.805419854	9.22567E-06	3.576E-05
3859	ENSG00000265055.2		124904048	1.026084955	9.27126E-06	3.59262E-05
3860	ENSG00000152977.10	ZIC1	7545	2.067926519	9.30414E-06	3.60359E-05
3861	ENSG00000287812.1			1.649604422	9.3249E-06	3.61107E-05
3862	ENSG00000275833.1			1.44292124	9.32527E-06	3.61107E-05
3863	ENSG00000236583.1			1.683464575	9.35962E-06	3.62371E-05
3864	ENSG00000101812.14	H2BW2	286436	2.563637395	9.36338E-06	3.62441E-05
3865	ENSG00000205955.4	HSP90AA5P		1.097813991	9.43094E-06	3.64843E-05
3866	ENSG00000132639.13	SNAP25	6616	1.058905129	9.46773E-06	3.6623E-05
3867	ENSG00000269898.1			1.679289471	9.49936E-06	3.67346E-05
3868	ENSG00000287616.1		105377918	1.809373001	9.51695E-06	3.67955E-05
3869	ENSG00000205754.12	SLCO1B7		2.31197547	9.5773E-06	3.70144E-05
3870	ENSG00000267033.1			1.529089305	9.58833E-06	3.70534E-05
3871	ENSG00000264717.5	NPY4R2	100996758	1.551819339	9.59554E-06	3.70776E-05
3872	ENSG00000163518.11	FCRL4	83417	1.74931435	9.59857E-06	3.70857E-05
3873	ENSG00000184478.7	OR56A3	390083	3.226629159	9.7509E-06	3.76449E-05
3874	ENSG00000259381.2			1.015277843	9.79216E-06	3.77821E-05
3875	ENSG00000215808.4	LINC01139	339535	1.674323816	9.82302E-06	3.78917E-05
3876	ENSG00000199944.1	RNU6-653P		2.08361302	9.82343E-06	3.78917E-05
3877	ENSG00000277332.1			3.668232753	9.85268E-06	3.80009E-05
3878	ENSG00000286489.1			2.171473785	9.87137E-06	3.80656E-05
3879	ENSG00000284624.1			1.240418034	1.00139E-05	3.85628E-05
3880	ENSG00000164616.16	FBXL21P		3.76617373	1.0018E-05	3.85726E-05
3881	ENSG00000283020.1	DUX4L37		1.762530425	1.00184E-05	3.85726E-05
3882	ENSG00000272163.1			1.88859397	1.00398E-05	3.86474E-05
3883	ENSG00000211665.3	IGLV3-16		2.059832445	1.00486E-05	3.8676E-05
3884	ENSG00000281420.1			1.106147849	1.00685E-05	3.87465E-05
3885	ENSG00000234156.3			1.148119086	1.01351E-05	3.89953E-05
3886	ENSG00000244036.3	UBE2H-DT		1.725456175	1.02006E-05	3.92269E-05
3887	ENSG00000167749.11	KLK4	9622	1.37502939	1.02626E-05	3.94477E-05
3888	ENSG00000129221.16	AIPL1	23746	1.746434578	1.02695E-05	3.94664E-05
3889	ENSG00000229781.1	RRN3P4		1.773370723	1.03774E-05	3.9858E-05
3890	ENSG00000276633.1			1.943747972	1.04415E-05	4.00732E-05
3891	ENSG00000230799.1	RPS2P16		1.220507051	1.04892E-05	4.02327E-05
3892	ENSG00000277901.1			1.715807779	1.04948E-05	4.02505E-05
3893	ENSG00000269072.1			2.840034798	1.04966E-05	4.02533E-05
3894	ENSG00000187066.8	TMEM262	100130348	1.097337778	1.05833E-05	4.05504E-05
3895	ENSG00000255525.1			2.011473318	1.0638E-05	4.07403E-05
3896	ENSG00000156009.10	MAGEA8	4107	1.915349382	1.06407E-05	4.07467E-05
3897	ENSG00000255583.2			2.765167885	1.06652E-05	4.08328E-05
3898	ENSG00000254044.5			2.963921324	1.07967E-05	4.13002E-05
3899	ENSG00000252297.1	RNU6-875P		1.644378168	1.08003E-05	4.13103E-05
3900	ENSG00000279573.1			1.305304011	1.08308E-05	4.14227E-05
3901	ENSG00000250974.4	LINC02196	105374642	2.376203216	1.08409E-05	4.14576E-05
3902	ENSG00000188385.12	JAKMIP3	282973	1.436633823	1.08843E-05	4.16075E-05
3903	ENSG00000286036.1			1.237731609	1.09082E-05	4.16905E-05
3904	ENSG00000170409.7			1.705145031	1.0993E-05	4.19863E-05
3905	ENSG00000245080.7	MIR3150BHG	105375650	1.056079181	1.10091E-05	4.20441E-05
3906	ENSG00000244159.1	RPS27AP13		1.787901997	1.1013E-05	4.20548E-05
3907	ENSG00000286123.1			3.622486527	1.1055E-05	4.21947E-05
3908	ENSG00000258976.1			1.353426904	1.1078E-05	4.22745E-05
3909	ENSG00000171564.12	FGB	2244	2.731154892	1.11481E-05	4.25215E-05
3910	ENSG00000121410.12	A1BG	1	1.087570747	1.11693E-05	4.2594E-05
3911	ENSG00000142233.14	NTN5	126147	1.065511717	1.11923E-05	4.26695E-05
3912	ENSG00000260661.1			1.526177475	1.11988E-05	4.26901E-05
3913	ENSG00000153157.13	SYCP2L	221711	1.539887067	1.1249E-05	4.28735E-05
3914	ENSG00000287448.1			2.85706576	1.12651E-05	4.29266E-05
3915	ENSG00000212163.6	SNORD91A		1.592811691	1.13076E-05	4.30759E-05
3916	ENSG00000185674.9	LYG2	254773	1.280820444	1.13124E-05	4.30902E-05
3917	ENSG00000258674.5			1.138023602	1.13691E-05	4.32782E-05
3918	ENSG00000287093.1			1.518835208	1.13694E-05	4.32782E-05
3919	ENSG00000204174.8	NPY4R	5540	1.370127837	1.13957E-05	4.33701E-05
3920	ENSG00000276404.1	MIR6835	102465502	1.448324494	1.14244E-05	4.34708E-05
3921	ENSG00000206014.7	OR7E161P		1.717861273	1.14594E-05	4.35875E-05
3922	ENSG00000254750.1	CASP1P2		1.09071464	1.14925E-05	4.37088E-05
3923	ENSG00000225546.6	LINC02476	105375475	3.494774973	1.14987E-05	4.37244E-05
3924	ENSG00000270115.1			1.383186293	1.15273E-05	4.38288E-05
3925	ENSG00000266017.1	MIR4477B	100616194	1.014323123	1.16209E-05	4.41634E-05
3926	ENSG00000227868.6	TEX46	729059	1.240120417	1.16425E-05	4.42326E-05
3927	ENSG00000198555.7			1.832565568	1.16692E-05	4.43257E-05
3928	ENSG00000231858.5	STAT4-AS1	105373805	1.546378785	1.17636E-05	4.4646E-05
3929	ENSG00000258317.1			1.008526758	1.17706E-05	4.46682E-05
3930	ENSG00000228252.9	COL6A4P2		1.408401768	1.17779E-05	4.46874E-05
3931	ENSG00000230077.1	MTAPP2		1.909157472	1.18207E-05	4.48411E-05
3932	ENSG00000184735.7	DDX53	168400	4.418779532	1.18453E-05	4.493E-05
3933	ENSG00000255605.1		107984377	2.065214506	1.18687E-05	4.50144E-05
3934	ENSG00000254339.5			2.157231606	1.18779E-05	4.50451E-05
3935	ENSG00000240386.3	LCE1F	353137	1.948654881	1.18798E-05	4.50463E-05
3936	ENSG00000275591.5	XKR5	389610	1.444579973	1.19314E-05	4.52219E-05
3937	ENSG00000234832.1	NXT1-AS1		1.099235163	1.2005E-05	4.54923E-05
3938	ENSG00000227098.2		105373757	1.781416544	1.20663E-05	4.5707E-05
3939	ENSG00000276968.1			1.103237731	1.21234E-05	4.59104E-05
3940	ENSG00000254208.1			1.870730766	1.21697E-05	4.60723E-05
3941	ENSG00000145536.15	ADAMTS16	170690	1.484170281	1.21755E-05	4.60898E-05
3942	ENSG00000253891.2			1.930577037	1.22339E-05	4.62979E-05
3943	ENSG00000220891.1	LL22NC03-63E9.3	648691	2.56961186	1.22453E-05	4.63363E-05
3944	ENSG00000260454.2	LINC02957	105376144	1.590986809	1.22629E-05	4.63988E-05
3945	ENSG00000202314.1	SNORD6	692075	1.00599248	1.22732E-05	4.64333E-05
3946	ENSG00000230526.1			1.269400006	1.23383E-05	4.66707E-05
3947	ENSG00000286636.1			2.224971956	1.23463E-05	4.66964E-05
3948	ENSG00000257890.1			1.815488114	1.2413E-05	4.69397E-05
3949	ENSG00000256108.1			1.701654765	1.24402E-05	4.70271E-05
3950	ENSG00000272563.1			1.700262536	1.24416E-05	4.70271E-05
3951	ENSG00000248367.1		124901151	1.03327274	1.24605E-05	4.70923E-05
3952	ENSG00000267409.1			1.452130288	1.24643E-05	4.71022E-05
3953	ENSG00000267074.1			1.111089039	1.25452E-05	4.74036E-05
3954	ENSG00000271993.1			1.272950512	1.25823E-05	4.75301E-05
3955	ENSG00000267293.1			1.214245159	1.26247E-05	4.7672E-05
3956	ENSG00000233455.2			3.200660658	1.27071E-05	4.79693E-05
3957	ENSG00000225255.6	PSLNR		3.809544677	1.27544E-05	4.81389E-05
3958	ENSG00000183287.14	CCBE1	147372	1.254802075	1.28321E-05	4.84139E-05
3959	ENSG00000258314.3			3.501179569	1.28796E-05	4.85744E-05
3960	ENSG00000229901.1	FOXO6-AS1	101929901	1.769590623	1.29345E-05	4.87629E-05
3961	ENSG00000185304.15	RGPD2	729857	1.265449009	1.3006E-05	4.90185E-05
3962	ENSG00000228008.1			1.324198861	1.30868E-05	4.93044E-05
3963	ENSG00000124678.20	TCP11	6954	1.844728153	1.31223E-05	4.94241E-05
3964	ENSG00000238024.1	DDX39BP2		2.058468829	1.31403E-05	4.9487E-05
3965	ENSG00000258919.1			1.315840437	1.31598E-05	4.95557E-05
3966	ENSG00000180869.4	LINC01555	439927	1.983053591	1.31952E-05	4.96795E-05
3967	ENSG00000226758.1	DISC1-IT1	104472714	2.167563562	1.32145E-05	4.9743E-05
3968	ENSG00000281162.2	LINC01127		1.27902963	1.3233E-05	4.98078E-05
3969	ENSG00000255836.1			1.823970729	1.33035E-05	5.00637E-05
3970	ENSG00000104760.17	FGL1	2267	1.823366097	1.33286E-05	5.01532E-05
3971	ENSG00000266852.2	MIR4482	100616323	1.341264985	1.33981E-05	5.03957E-05
3972	ENSG00000274051.1			1.47159245	1.35132E-05	5.08044E-05
3973	ENSG00000274549.1			1.832296046	1.35784E-05	5.10351E-05
3974	ENSG00000102109.9	PCSK1N	27344	1.531649285	1.35994E-05	5.11093E-05
3975	ENSG00000105374.10	NKG7	4818	1.105255664	1.36657E-05	5.13422E-05
3976	ENSG00000178403.4	NEUROG2	63973	2.055690785	1.36666E-05	5.13422E-05
3977	ENSG00000236264.5	RPL26P30		1.255443655	1.36884E-05	5.14192E-05
3978	ENSG00000233309.1	RPS6P12		2.419692697	1.3699E-05	5.14541E-05
3979	ENSG00000287243.2	THCAT155	100129223	2.687203293	1.37615E-05	5.16842E-05
3980	ENSG00000145192.13	AHSG	197	1.583260138	1.37834E-05	5.17516E-05
3981	ENSG00000241278.1	ENPP7P4		1.367862282	1.37983E-05	5.18028E-05
3982	ENSG00000255422.3			1.18384904	1.38343E-05	5.19233E-05
3983	ENSG00000228352.2	DCAF12-AS1		1.030329059	1.38574E-05	5.20048E-05
3984	ENSG00000248837.7		105374524	2.318972108	1.38765E-05	5.20667E-05
3985	ENSG00000269161.1			1.09837796	1.39146E-05	5.21851E-05
3986	ENSG00000278902.1			2.353446737	1.39552E-05	5.23223E-05
3987	ENSG00000243710.8	CFAP57	149465	1.213635592	1.4127E-05	5.29215E-05
3988	ENSG00000128606.13	LRRC17	10234	1.227146672	1.41527E-05	5.30026E-05
3989	ENSG00000224079.1	CHLSN-AS1		1.17199542	1.42829E-05	5.34501E-05
3990	ENSG00000218819.6	TDRD15	100129278	3.735162714	1.4351E-05	5.36896E-05
3991	ENSG00000175093.5	SPSB4	92369	1.318434059	1.43553E-05	5.37006E-05
3992	ENSG00000132185.18	FCRLA	84824	1.214374478	1.43709E-05	5.37539E-05
3993	ENSG00000104970.11	KIR3DX1		1.321106171	1.43807E-05	5.37855E-05
3994	ENSG00000224373.3	IGHV4-59		1.62231791	1.43832E-05	5.37897E-05
3995	ENSG00000230990.2			1.603596187	1.44052E-05	5.3867E-05
3996	ENSG00000230010.2			1.081715098	1.4433E-05	5.3966E-05
3997	ENSG00000271778.1			1.073598956	1.44745E-05	5.41057E-05
3998	ENSG00000271555.1			1.15230502	1.45818E-05	5.44811E-05
3999	ENSG00000261298.1			2.414754717	1.45963E-05	5.45249E-05
4000	ENSG00000227496.3		102723834	1.113721205	1.46658E-05	5.47691E-05
4001	ENSG00000105369.10	CD79A	973	1.418290658	1.46683E-05	5.47735E-05
4002	ENSG00000248498.4	ASNSP1		2.521671117	1.47171E-05	5.49401E-05
4003	ENSG00000244378.1	RPS2P45		1.516277365	1.47811E-05	5.51685E-05
4004	ENSG00000271428.1	MPHOSPH6P1		1.599981742	1.47837E-05	5.51714E-05
4005	ENSG00000230387.4	LINC03125	100505664	1.38380816	1.47846E-05	5.51714E-05
4006	ENSG00000287798.1			1.897594604	1.47954E-05	5.52011E-05
4007	ENSG00000226965.2			1.391080151	1.47983E-05	5.52069E-05
4008	ENSG00000260288.4		101929408	1.107682493	1.4817E-05	5.52715E-05
4009	ENSG00000205809.9	KLRC2	3822	1.441159857	1.49698E-05	5.5815E-05
4010	ENSG00000234967.2			2.215763305	1.49965E-05	5.59043E-05
4011	ENSG00000274259.2	SYNGAP1-AS1		1.249949413	1.50112E-05	5.59538E-05
4012	ENSG00000254815.6	LMNTD2-AS1	692247	1.223793091	1.50581E-05	5.61233E-05
4013	ENSG00000254495.1			1.922872503	1.51167E-05	5.63205E-05
4014	ENSG00000128438.10	TBC1D27P		1.549869942	1.52303E-05	5.67118E-05
4015	ENSG00000244247.2			1.926731586	1.52772E-05	5.68649E-05
4016	ENSG00000244230.3	RN7SL151P		1.82892973	1.52905E-05	5.69049E-05
4017	ENSG00000250284.3			3.103516678	1.52908E-05	5.69049E-05
4018	ENSG00000212607.1	SNORA3B	677826	1.00310554	1.53118E-05	5.69778E-05
4019	ENSG00000244791.3		101927657	1.645215682	1.54043E-05	5.73111E-05
4020	ENSG00000282418.1			1.952382354	1.55169E-05	5.76869E-05
4021	ENSG00000241135.6	LINC00881		1.394939103	1.57257E-05	5.84191E-05
4022	ENSG00000236095.1			1.057270099	1.57741E-05	5.85827E-05
4023	ENSG00000224893.7	LINC02840	105378065	2.108534439	1.59913E-05	5.93337E-05
4024	ENSG00000183598.4	H3C13	653604	1.105404497	1.60011E-05	5.93643E-05
4025	ENSG00000252835.1	SCARNA21	677763	1.475954335	1.605E-05	5.95346E-05
4026	ENSG00000254331.1	CKS1BP7		1.235954965	1.61747E-05	5.99803E-05
4027	ENSG00000279000.3	OR10A6	390093	1.821638572	1.63189E-05	6.04868E-05
4028	ENSG00000215529.12	EFCAB8	388795	1.308089888	1.63912E-05	6.07378E-05
4029	ENSG00000113905.5	HRG	3273	1.58565664	1.6399E-05	6.07555E-05
4030	ENSG00000272540.1			1.009780481	1.64639E-05	6.09785E-05
4031	ENSG00000236498.1			1.020901738	1.65029E-05	6.11118E-05
4032	ENSG00000273521.1			1.155624615	1.65492E-05	6.12605E-05
4033	ENSG00000278924.1			1.06639473	1.65561E-05	6.128E-05
4034	ENSG00000178222.13	RNF212	285498	1.508526108	1.6576E-05	6.13479E-05
4035	ENSG00000231439.4	WASIR2		1.617259836	1.66222E-05	6.15133E-05
4036	ENSG00000171889.5	MIR31HG	554202	1.235094827	1.66591E-05	6.16326E-05
4037	ENSG00000179873.14	NLRP11	204801	1.88404798	1.67266E-05	6.18593E-05
4038	ENSG00000183251.4	OR51B4	79339	3.53363542	1.67366E-05	6.18847E-05
4039	ENSG00000171246.6	NPTX1	4884	1.341008268	1.6752E-05	6.19357E-05
4040	ENSG00000259242.2			1.973541918	1.69071E-05	6.25035E-05
4041	ENSG00000258581.2			1.332806724	1.70235E-05	6.29221E-05
4042	ENSG00000270919.1			1.716471961	1.70776E-05	6.31103E-05
4043	ENSG00000176198.3	OR11H4	390442	2.739285776	1.7129E-05	6.32826E-05
4044	ENSG00000233928.6			1.843027352	1.71863E-05	6.34764E-05
4045	ENSG00000266102.1			2.328480713	1.7221E-05	6.35989E-05
4046	ENSG00000284685.1			1.8572065	1.73679E-05	6.41052E-05
4047	ENSG00000139193.4	CD27	939	1.05137806	1.74142E-05	6.42584E-05
4048	ENSG00000265480.5	KRT18P55		1.274902584	1.74555E-05	6.43986E-05
4049	ENSG00000152969.20	JAKMIP1	152789	1.374965433	1.74827E-05	6.4481E-05
4050	ENSG00000250563.1	KNOP1P5		2.665064045	1.74849E-05	6.44832E-05
4051	ENSG00000211663.2	IGLV3-19		1.657763989	1.75549E-05	6.46992E-05
4052	ENSG00000270816.6	LINC00221	338005	3.271472942	1.75702E-05	6.47438E-05
4053	ENSG00000176177.10	ENTHD1	150350	1.180444195	1.75805E-05	6.47754E-05
4054	ENSG00000258038.6	LINC02327	102724917	3.360209484	1.77476E-05	6.53588E-05
4055	ENSG00000224728.1	IMPDH1P8		1.160476542	1.7768E-05	6.54237E-05
4056	ENSG00000255750.6			1.425172574	1.77713E-05	6.54299E-05
4057	ENSG00000108684.15	ASIC2	40	1.416560999	1.80119E-05	6.62789E-05
4058	ENSG00000100156.10	SLC16A8	23539	1.120723082	1.80239E-05	6.63166E-05
4059	ENSG00000270048.1			1.504021787	1.80324E-05	6.6342E-05
4060	ENSG00000252202.1	Y_RNA		1.426285312	1.81332E-05	6.67004E-05
4061	ENSG00000204011.5	COL5A1-AS1		1.913853056	1.82051E-05	6.69525E-05
4062	ENSG00000169876.14	MUC17	140453	2.118912173	1.82379E-05	6.70667E-05
4063	ENSG00000262248.1			1.312127358	1.82613E-05	6.71466E-05
4064	ENSG00000279887.1			1.86538474	1.82742E-05	6.71834E-05
4065	ENSG00000268333.1			2.175887003	1.84922E-05	6.79578E-05
4066	ENSG00000263146.2	LINC01896		3.184363241	1.8505E-05	6.79859E-05
4067	ENSG00000260646.1			1.200927125	1.85225E-05	6.8044E-05
4068	ENSG00000264006.8	AKR1C8	340811	1.963311179	1.85346E-05	6.8082E-05
4069	ENSG00000287021.1			2.081603295	1.85576E-05	6.81601E-05
4070	ENSG00000280062.1			1.229320876	1.85707E-05	6.8202E-05
4071	ENSG00000250292.1			1.688120857	1.8583E-05	6.82408E-05
4072	ENSG00000258072.1			1.841118602	1.8665E-05	6.85192E-05
4073	ENSG00000265282.1			1.012373579	1.87322E-05	6.87444E-05
4074	ENSG00000174498.14	IGDCC3	9543	1.406784062	1.87765E-05	6.89005E-05
4075	ENSG00000286089.1			1.190757025	1.88183E-05	6.90409E-05
4076	ENSG00000236658.1	FIBCD1-AS1		2.627608397	1.88485E-05	6.91454E-05
4077	ENSG00000259203.2		101928499	3.410065891	1.90972E-05	7.00124E-05
4078	ENSG00000183246.7	RIMBP3C	150221	1.560147835	1.92376E-05	7.05013E-05
4079	ENSG00000261243.1			1.23375342	1.92797E-05	7.0636E-05
4080	ENSG00000235419.5			1.099178265	1.93142E-05	7.07493E-05
4081	ENSG00000260229.1	PPIAP51		1.007738892	1.93307E-05	7.07966E-05
4082	ENSG00000239590.1	OR1J4	26219	1.743959676	1.93973E-05	7.10274E-05
4083	ENSG00000232259.1			1.353393689	1.95069E-05	7.13822E-05
4084	ENSG00000225329.3			1.515324064	1.96395E-05	7.18413E-05
4085	ENSG00000158874.11	APOA2	336	2.600602984	1.96505E-05	7.18747E-05
4086	ENSG00000253998.3	IGKV2-29		2.151282411	1.97929E-05	7.23821E-05
4087	ENSG00000231868.1			1.305614855	1.98079E-05	7.24185E-05
4088	ENSG00000274721.1			1.644798624	1.98388E-05	7.25235E-05
4089	ENSG00000214694.12	ARHGEF33	100271715	1.240410243	2.00407E-05	7.32088E-05
4090	ENSG00000211945.2	IGHV1-18		1.569790963	2.00611E-05	7.32752E-05
4091	ENSG00000286119.1			1.798879988	2.00796E-05	7.33361E-05
4092	ENSG00000258500.2			1.548520322	2.01632E-05	7.3628E-05
4093	ENSG00000241765.2	RPS26P45		1.818047221	2.01929E-05	7.37227E-05
4094	ENSG00000228980.4	LINC01205	401082	2.614485156	2.03463E-05	7.42622E-05
4095	ENSG00000231185.7	SPRY4-AS1		1.028206634	2.0419E-05	7.45069E-05
4096	ENSG00000224857.1	LINC01691		1.790579845	2.04811E-05	7.472E-05
4097	ENSG00000237371.1			1.272990016	2.05028E-05	7.47852E-05
4098	ENSG00000252916.1	RNU6-762P		1.378392329	2.05316E-05	7.48765E-05
4099	ENSG00000269842.5			1.833448458	2.06223E-05	7.51934E-05
4100	ENSG00000255363.3	LINC02757	101928813	1.119628765	2.0786E-05	7.57625E-05
4101	ENSG00000260046.1			1.086244468	2.07894E-05	7.57679E-05
4102	ENSG00000215196.5	BASP1-AS1		1.161370689	2.09545E-05	7.63135E-05
4103	ENSG00000285970.1			1.574780811	2.099E-05	7.64287E-05
4104	ENSG00000116785.14	CFHR3	10878	1.052544547	2.09992E-05	7.64482E-05
4105	ENSG00000248432.1			2.906683011	2.11836E-05	7.7084E-05
4106	ENSG00000187037.8	GPR141	353345	1.0430246	2.12318E-05	7.7242E-05
4107	ENSG00000197721.17	CR1L	1379	1.544451121	2.12919E-05	7.74426E-05
4108	ENSG00000257935.2	LHX5-AS1		2.28426509	2.1324E-05	7.75452E-05
4109	ENSG00000262003.1		101927727	1.306132961	2.13691E-05	7.76878E-05
4110	ENSG00000183396.4	TMEM89	440955	1.575994143	2.14366E-05	7.79261E-05
4111	ENSG00000225721.5			1.177783308	2.15352E-05	7.82703E-05
4112	ENSG00000279791.1			1.868640077	2.17686E-05	7.90896E-05
4113	ENSG00000235899.2	LINC01564		1.187123514	2.18251E-05	7.92802E-05
4114	ENSG00000260828.1	HMGB3P32		1.41984233	2.21623E-05	8.04536E-05
4115	ENSG00000272715.1			1.001735685	2.2167E-05	8.04558E-05
4116	ENSG00000188219.14	POTEE	445582	1.234167308	2.221E-05	8.05972E-05
4117	ENSG00000134545.14	KLRC1	3821	1.027078279	2.24321E-05	8.13362E-05
4118	ENSG00000288266.1			1.952463609	2.24383E-05	8.13427E-05
4119	ENSG00000242610.2	OR5BH1P		2.507822981	2.25563E-05	8.17339E-05
4120	ENSG00000229393.1			1.286582107	2.26241E-05	8.19722E-05
4121	ENSG00000224821.5	COL4A2-AS2	100129836	1.96683921	2.28663E-05	8.27894E-05
4122	ENSG00000133636.11	NTS	4922	2.26818075	2.29317E-05	8.29806E-05
4123	ENSG00000267301.1	RPL23AP77		1.486125578	2.29509E-05	8.3035E-05
4124	ENSG00000267908.2	ZSCAN5DP		1.490453564	2.3261E-05	8.40648E-05
4125	ENSG00000182583.12	VCX	26609	2.201248743	2.34764E-05	8.47584E-05
4126	ENSG00000251538.7	LINC02201	101927379	2.233165177	2.35807E-05	8.50808E-05
4127	ENSG00000213082.3	PPP1R14BP2		1.000453697	2.37707E-05	8.57118E-05
4128	ENSG00000186431.19	FCAR	2204	1.180455747	2.40038E-05	8.64736E-05
4129	ENSG00000261156.6	LINC01989	101927239	2.645257991	2.42391E-05	8.72496E-05
4130	ENSG00000240244.3	GAPDHP33		1.736666145	2.4318E-05	8.74863E-05
4131	ENSG00000286874.1			1.065937838	2.46362E-05	8.85425E-05
4132	ENSG00000252915.1	Y_RNA		1.345387964	2.46546E-05	8.86007E-05
4133	ENSG00000287071.1	CDRT15P4		1.15869775	2.47856E-05	8.90553E-05
4134	ENSG00000231393.1			1.787174488	2.48595E-05	8.92883E-05
4135	ENSG00000234593.3	KAZN-AS1		1.29593769	2.49683E-05	8.96385E-05
4136	ENSG00000249618.6	LINC02465	107986313	2.039385965	2.51221E-05	9.01743E-05
4137	ENSG00000264808.1			1.027894858	2.52658E-05	9.06821E-05
4138	ENSG00000226777.7	FAM30A	9834	1.346439469	2.52786E-05	9.07197E-05
4139	ENSG00000232389.1			1.12633191	2.53753E-05	9.10561E-05
4140	ENSG00000228823.1	PDE4DIPP10		2.430557672	2.53769E-05	9.10561E-05
4141	ENSG00000181215.15	C4orf50	389197	1.127364052	2.56216E-05	9.18425E-05
4142	ENSG00000228626.1			1.610616488	2.56636E-05	9.19681E-05
4143	ENSG00000280023.1			1.676179533	2.57261E-05	9.21754E-05
4144	ENSG00000265912.1			1.48059142	2.58453E-05	9.25776E-05
4145	ENSG00000259155.2	STK16P1		1.617189883	2.59381E-05	9.28846E-05
4146	ENSG00000223432.1			1.882021958	2.59706E-05	9.29929E-05
4147	ENSG00000214273.4	AGGF1P1		1.65570316	2.61246E-05	9.34936E-05
4148	ENSG00000224763.1	FDPSP8		1.490443382	2.62587E-05	9.39312E-05
4149	ENSG00000253766.1			2.016528465	2.62623E-05	9.39354E-05
4150	ENSG00000155052.14	CNTNAP5	129684	2.212857142	2.63687E-05	9.42903E-05
4151	ENSG00000230922.1		101927551	2.212306311	2.64137E-05	9.44429E-05
4152	ENSG00000286797.1			1.579012558	2.64424E-05	9.45284E-05
4153	ENSG00000179292.5	TMEM151A	256472	1.33078531	2.64506E-05	9.45494E-05
4154	ENSG00000179253.3	CDH4-AS2	100128310	2.118874754	2.65038E-05	9.47222E-05
4155	ENSG00000230649.3			1.47333831	2.66025E-05	9.50578E-05
4156	ENSG00000277007.1			1.002551638	2.66538E-05	9.52068E-05
4157	ENSG00000279742.1			1.11545197	2.67233E-05	9.54294E-05
4158	ENSG00000231776.5	LINC01611		2.670000421	2.67344E-05	9.54606E-05
4159	ENSG00000279653.1			1.382508165	2.68563E-05	9.58869E-05
4160	ENSG00000236543.3		102723971	2.435270868	2.6897E-05	9.59893E-05
4161	ENSG00000233388.2	TRMT112P8		2.021660688	2.69487E-05	9.61563E-05
4162	ENSG00000256292.2	LINC02376		2.389199055	2.71003E-05	9.66799E-05
4163	ENSG00000187862.12	TTC24	164118	1.118988159	2.73797E-05	9.76327E-05
4164	ENSG00000214121.4	PRDX1P1		1.208670306	2.74565E-05	9.78626E-05
4165	ENSG00000277383.1	BICRA-AS2	128667229	1.032000028	2.74707E-05	9.79045E-05
4166	ENSG00000236924.1	LINC02851	105375968	1.77279368	2.76122E-05	9.83558E-05
4167	ENSG00000211946.3	IGHV3-20		1.736691796	2.76976E-05	9.86332E-05
4168	ENSG00000227195.11	MIR663AHG	284801	2.379280017	2.77702E-05	9.88515E-05
4169	ENSG00000221962.5	TMEM14EP		1.574566267	2.77714E-05	9.88515E-05
4170	ENSG00000171903.16	CYP4F11	57834	1.44320913	2.78282E-05	9.90362E-05
4171	ENSG00000253848.1	RBM12B-DT		1.159358336	2.78717E-05	9.91723E-05
4172	ENSG00000254256.1	RPSAP71		2.909598421	2.78883E-05	9.92055E-05
4173	ENSG00000151572.18	ANO4	121601	1.424121084	2.80986E-05	9.98906E-05
4174	ENSG00000258083.2	OR9A4	130075	2.186112806	2.83191E-05	0.000100638
4175	ENSG00000255314.4		101928894	2.024629052	2.83387E-05	0.000100696
4176	ENSG00000215448.3	SRMP1		2.026599364	2.83402E-05	0.000100696
4177	ENSG00000241081.1	RPL22P2		1.167097205	2.83542E-05	0.000100736
4178	ENSG00000286011.1			1.362759472	2.84641E-05	0.000101109
4179	ENSG00000253642.6			2.729680268	2.85202E-05	0.000101299
4180	ENSG00000225226.2	LINC01795		2.712790508	2.86537E-05	0.000101718
4181	ENSG00000251922.1		124902573	1.674398976	2.88907E-05	0.000102532
4182	ENSG00000107831.13	FGF8	2253	1.54003854	2.89332E-05	0.000102664
4183	ENSG00000278733.1			1.088289654	2.89571E-05	0.00010274
4184	ENSG00000213070.3	HMGB3P6		1.481778585	2.91279E-05	0.00010329
4185	ENSG00000287369.1			1.201271463	2.91588E-05	0.000103391
4186	ENSG00000272769.1			1.307053717	2.92138E-05	0.000103567
4187	ENSG00000182156.10	ENPP7	339221	1.826741987	2.93492E-05	0.000103982
4188	ENSG00000168070.12	MAJIN	283129	1.969222545	2.96395E-05	0.000104964
4189	ENSG00000250447.6	LINC02105	105378966	2.322344629	2.97336E-05	0.000105278
4190	ENSG00000187621.15	TCL6	124903372	1.640624259	2.98522E-05	0.000105679
4191	ENSG00000120160.11	EQTN	54586	1.479795787	2.98939E-05	0.000105815
4192	ENSG00000236345.1	SCAT8	112935969	1.299665793	2.99165E-05	0.000105869
4193	ENSG00000236772.1			1.325809728	3.00935E-05	0.000106439
4194	ENSG00000287967.1			1.018227442	3.08103E-05	0.000108867
4195	ENSG00000235689.1			2.112688781	3.08432E-05	0.000108974
4196	ENSG00000264714.1	KIAA0895LP1		1.396974205	3.08529E-05	0.000108998
4197	ENSG00000201151.1		124900460	1.47118818	3.08727E-05	0.000109058
4198	ENSG00000197241.3	SLC2A7	155184	1.201887253	3.11287E-05	0.000109914
4199	ENSG00000234142.2	LINC01675		1.79785555	3.11923E-05	0.000110109
4200	ENSG00000080618.17	CPB2	1361	1.597584595	3.1215E-05	0.000110165
4201	ENSG00000132204.14	LINC00470		2.174453027	3.13027E-05	0.00011045
4202	ENSG00000167779.9	IGFBP6	3489	1.132269444	3.13443E-05	0.000110577
4203	ENSG00000281593.1			1.240320607	3.13619E-05	0.000110619
4204	ENSG00000261159.1			1.098071447	3.13697E-05	0.000110637
4205	ENSG00000240041.1	IGHJ4		1.696052997	3.13972E-05	0.000110704
4206	ENSG00000018236.15	CNTN1	1272	1.153113235	3.16685E-05	0.000111581
4207	ENSG00000225951.1	ODF2-AS1		1.50321761	3.16932E-05	0.000111658
4208	ENSG00000287727.1			1.02437891	3.20543E-05	0.00011289
4209	ENSG00000185972.6	CCIN	881	1.09824088	3.23915E-05	0.000113997
4210	ENSG00000230109.1	LINC02643	105376444	2.077357413	3.24077E-05	0.000114044
4211	ENSG00000187682.2	ERAS	3266	1.677188129	3.25525E-05	0.000114523
4212	ENSG00000104918.8	RETN	56729	1.308895535	3.26101E-05	0.000114705
4213	ENSG00000233012.2	HDAC1P2		1.044669598	3.26711E-05	0.000114899
4214	ENSG00000227676.4	LINC01068		1.268527035	3.27578E-05	0.000115194
4215	ENSG00000261402.1			1.668404442	3.28324E-05	0.000115446
4216	ENSG00000179242.16	CDH4	1002	1.274840582	3.317E-05	0.000116592
4217	ENSG00000174255.8	ZNF80	7634	1.322318681	3.326E-05	0.000116888
4218	ENSG00000253414.3			1.026434341	3.36327E-05	0.000118093
4219	ENSG00000287158.1			1.912868584	3.36601E-05	0.000118168
4220	ENSG00000211891.6	IGHE		1.366194378	3.37388E-05	0.000118392
4221	ENSG00000253824.2		124901990	1.343736033	3.37991E-05	0.000118582
4222	ENSG00000228290.7	TBX18-AS1	102724201	1.488842984	3.40981E-05	0.000119589
4223	ENSG00000255394.4			2.892126501	3.41287E-05	0.000119686
4224	ENSG00000262171.1			1.609000524	3.41918E-05	0.000119897
4225	ENSG00000268499.1			1.168333533	3.4221E-05	0.000119988
4226	ENSG00000253497.1	IGKV1-13		2.075959164	3.42746E-05	0.000120155
4227	ENSG00000259098.1			2.662706568	3.43025E-05	0.000120242
4228	ENSG00000211659.2	IGLV3-25		1.538034089	3.44166E-05	0.000120632
4229	ENSG00000233379.2			3.272598798	3.46581E-05	0.000121427
4230	ENSG00000171487.15	NLRP5	126206	2.319396262	3.47254E-05	0.000121649
4231	ENSG00000287655.1			1.302271632	3.49399E-05	0.000122336
4232	ENSG00000285922.1			2.506599477	3.50569E-05	0.000122702
4233	ENSG00000231793.5	DOC2GP		1.160514153	3.51484E-05	0.000123012
4234	ENSG00000179674.4	ARL14	80117	1.305030048	3.51888E-05	0.000123142
4235	ENSG00000253659.2			3.262435131	3.52337E-05	0.000123278
4236	ENSG00000282980.1			1.171516285	3.54127E-05	0.000123849
4237	ENSG00000234423.2	LINC01250	101927554	2.442405987	3.55261E-05	0.000124224
4238	ENSG00000253338.1	IGLV3-29		2.60902429	3.55303E-05	0.000124228
4239	ENSG00000260284.1	TPSP2		2.025663786	3.55395E-05	0.000124249
4240	ENSG00000231331.1			1.917792075	3.56897E-05	0.000124719
4241	ENSG00000218713.1			1.773318805	3.58003E-05	0.000125083
4242	ENSG00000145526.12	CDH18	1016	1.732551908	3.59964E-05	0.000125709
4243	ENSG00000179284.5	DAND5	199699	1.132778514	3.60549E-05	0.000125884
4244	ENSG00000226652.3	PSMD10P2		1.021045314	3.6205E-05	0.000126353
4245	ENSG00000279894.1			1.001611094	3.62603E-05	0.000126534
4246	ENSG00000199961.1	SNORD1B		1.384550176	3.64005E-05	0.000126957
4247	ENSG00000227053.1	MUC12-AS1	102724094	1.310873218	3.64932E-05	0.000127235
4248	ENSG00000254233.1	LINC02365		1.684245535	3.67179E-05	0.000127962
4249	ENSG00000253584.1			3.573211694	3.67937E-05	0.000128204
4250	ENSG00000230023.2	LINC02800	284632	1.261971699	3.68163E-05	0.00012826
4251	ENSG00000287964.1			1.687331086	3.68764E-05	0.000128447
4252	ENSG00000287212.1			1.846751171	3.69859E-05	0.000128817
4253	ENSG00000235289.1	BRD7P6		1.932116819	3.72414E-05	0.00012965
4254	ENSG00000244468.1	TM4SF18-AS1		2.366238213	3.78064E-05	0.000131467
4255	ENSG00000228510.2			1.330058097	3.78448E-05	0.000131577
4256	ENSG00000161860.8	SYCE2	256126	1.075652846	3.82386E-05	0.000132842
4257	ENSG00000231728.5	TMSB15B-AS1		1.091710639	3.82758E-05	0.000132959
4258	ENSG00000277558.1			1.595238108	3.83137E-05	0.000133067
4259	ENSG00000269954.2	BLK-AS1		1.550655089	3.84956E-05	0.000133653
4260	ENSG00000234515.1	PPP1R2P1		1.232276513	3.89216E-05	0.000135061
4261	ENSG00000229586.2	TNPO1P3		1.266791476	3.93372E-05	0.000136443
4262	ENSG00000287477.1			1.262675637	3.93616E-05	0.000136504
4263	ENSG00000221878.13	PSG7	5676	2.240958941	3.93771E-05	0.000136534
4264	ENSG00000187553.10	CYP26C1	340665	1.976588497	3.94703E-05	0.000136845
4265	ENSG00000204277.1	LINC01993	100996291	1.387659124	3.95936E-05	0.000137236
4266	ENSG00000272196.2			1.512085903	3.96082E-05	0.000137275
4267	ENSG00000274737.1			1.243461274	4.00233E-05	0.000138653
4268	ENSG00000274576.2	IGHV2-70		1.742965316	4.01737E-05	0.00013915
4269	ENSG00000253535.5	ADAM7-AS1		1.968362985	4.02247E-05	0.000139314
4270	ENSG00000100473.18	COCH	1690	1.479352241	4.03141E-05	0.000139581
4271	ENSG00000231772.5			1.468128423	4.03159E-05	0.000139581
4272	ENSG00000287459.1			3.623508118	4.04051E-05	0.000139854
4273	ENSG00000163254.5	CRYGC	1420	2.440996055	4.05216E-05	0.000140244
4274	ENSG00000125823.12	CSTL1	128817	1.282579066	4.05441E-05	0.00014031
4275	ENSG00000251127.2			2.096110009	4.06819E-05	0.000140738
4276	ENSG00000236993.2	GAPDHP21		1.06989621	4.10586E-05	0.000141936
4277	ENSG00000238793.1	SNORD124	101340251	1.603963297	4.10736E-05	0.000141969
4278	ENSG00000212371.1			1.272249836	4.1135E-05	0.000142169
4279	ENSG00000242971.3	RN7SL233P		1.689959176	4.12746E-05	0.000142626
4280	ENSG00000168621.15	GDNF	2668	1.208871153	4.15378E-05	0.000143486
4281	ENSG00000277581.1			1.661768627	4.15541E-05	0.00014353
4282	ENSG00000266445.1	NARF-AS1		1.446534813	4.15811E-05	0.000143598
4283	ENSG00000161270.20	NPHS1	4868	1.63953958	4.16435E-05	0.000143789
4284	ENSG00000230662.1	TNPO1P2		1.949406038	4.16626E-05	0.000143829
4285	ENSG00000254397.1			1.138923175	4.17535E-05	0.000144131
4286	ENSG00000214534.5	ZNF705EP		1.19460585	4.18409E-05	0.000144395
4287	ENSG00000187123.15	LYPD6	130574	1.156862349	4.20382E-05	0.00014505
4288	ENSG00000130045.16	NXNL2	158046	1.108309734	4.23883E-05	0.000146195
4289	ENSG00000255931.2			1.417134621	4.24002E-05	0.000146223
4290	ENSG00000287958.1			2.184815738	4.24449E-05	0.000146352
4291	ENSG00000244346.1	C9orf85P2		1.441724479	4.25751E-05	0.000146737
4292	ENSG00000138075.14	ABCG5	64240	1.204924731	4.26077E-05	0.000146832
4293	ENSG00000156414.19	TDRD9	122402	1.201986218	4.26611E-05	0.000146965
4294	ENSG00000225282.1	KLHL5P1		1.264859805	4.30057E-05	0.000148105
4295	ENSG00000235118.8	FAM237A	200726	2.057592587	4.34343E-05	0.000149459
4296	ENSG00000263667.3	LINC03066	285766	2.136429472	4.35833E-05	0.000149886
4297	ENSG00000257221.4			1.013181788	4.39808E-05	0.000151187
4298	ENSG00000236956.2	NF1P8		4.072249051	4.43674E-05	0.000152437
4299	ENSG00000257859.2	CASC18	101929110	1.072788724	4.44165E-05	0.000152592
4300	ENSG00000180152.3	XIAPP3		1.808940006	4.44665E-05	0.000152751
4301	ENSG00000258633.1	RRAGAP1-AS1	101927351	2.537147126	4.46799E-05	0.000153458
4302	ENSG00000231612.1			1.626626711	4.4838E-05	0.000153987
4303	ENSG00000266369.1			2.669565955	4.51217E-05	0.000154908
4304	ENSG00000183668.18	PSG9	5678	1.55042556	4.52586E-05	0.000155351
4305	ENSG00000229404.6	LINC00858	170425	2.144811	4.52817E-05	0.000155417
4306	ENSG00000207217.1	SNORA80D	109616999	1.462916099	4.5358E-05	0.000155652
4307	ENSG00000230912.1			1.082633116	4.54359E-05	0.000155892
4308	ENSG00000189409.14	MMP23B	8510	1.058824943	4.57975E-05	0.000157038
4309	ENSG00000197251.3	LINC00336	401253	1.686093647	4.59244E-05	0.000157445
4310	ENSG00000237281.1	CATIP-AS2	103689911	1.878257273	4.59321E-05	0.000157458
4311	ENSG00000268818.2			1.573701895	4.60479E-05	0.000157814
4312	ENSG00000285923.1			1.075064261	4.63961E-05	0.000158884
4313	ENSG00000226012.1			1.057091269	4.65029E-05	0.000159208
4314	ENSG00000258428.5			1.680761114	4.69246E-05	0.0001605
4315	ENSG00000205212.5	CCDC144NL		2.221598195	4.71044E-05	0.000161101
4316	ENSG00000270182.1			1.877316493	4.7155E-05	0.000161246
4317	ENSG00000124391.5	IL17C	27189	1.590126775	4.73008E-05	0.000161703
4318	ENSG00000272942.1			2.138206221	4.73602E-05	0.000161878
4319	ENSG00000278637.2	H4C1	8359	1.623752651	4.76136E-05	0.000162702
4320	ENSG00000143452.16	HORMAD1	84072	1.620948717	4.76962E-05	0.00016297
4321	ENSG00000211947.2	IGHV3-21		1.528829206	4.83122E-05	0.00016495
4322	ENSG00000250590.6	LINC02492		3.774300406	4.83132E-05	0.00016495
4323	ENSG00000287784.1			2.087945791	4.84978E-05	0.00016551
4324	ENSG00000255484.2	LINC02764	387810	1.958994478	4.90618E-05	0.000167276
4325	ENSG00000224559.2	LINC01087	101927994	2.728761324	4.91372E-05	0.000167504
4326	ENSG00000262905.1			1.55542666	4.91825E-05	0.00016763
4327	ENSG00000163746.12	PLSCR2	57047	1.165736831	4.95363E-05	0.000168778
4328	ENSG00000280375.1			1.725266416	4.99281E-05	0.000170043
4329	ENSG00000286216.1			1.004072308	5.00813E-05	0.000170481
4330	ENSG00000278910.4	BANCR		1.463079091	5.02616E-05	0.000171043
4331	ENSG00000253818.1	IGLV1-41		1.945051249	5.02793E-05	0.000171088
4332	ENSG00000159374.17	M1AP	130951	1.476249274	5.03588E-05	0.000171344
4333	ENSG00000256950.2			1.601391979	5.08911E-05	0.000173066
4334	ENSG00000211959.2	IGHV4-39		1.54012781	5.1352E-05	0.000174574
4335	ENSG00000214815.2	IMPDH1P3		1.803054947	5.14151E-05	0.000174774
4336	ENSG00000120337.8	TNFSF18	8995	1.307809819	5.14815E-05	0.000174924
4337	ENSG00000131668.14	BARX1	56033	1.245800165	5.1514E-05	0.000175005
4338	ENSG00000230191.1	IFITM3P4		1.141102476	5.16074E-05	0.000175292
4339	ENSG00000203801.8	LINC00222	387111	1.147249896	5.19354E-05	0.000176315
4340	ENSG00000183444.10	OR7E38P		1.050088642	5.20656E-05	0.000176727
4341	ENSG00000254486.1	LINC02547	105376554	1.933604364	5.21201E-05	0.000176867
4342	ENSG00000278200.1	LINC01971		1.679091216	5.21376E-05	0.000176911
4343	ENSG00000254887.1	LNROP	128071540	1.025828628	5.23956E-05	0.000177741
4344	ENSG00000287446.1			1.914036488	5.25789E-05	0.000178317
4345	ENSG00000237669.1			1.158984597	5.26183E-05	0.000178435
4346	ENSG00000286795.1			2.095176697	5.26698E-05	0.000178594
4347	ENSG00000257046.5	SLCO1B3-SLCO1B7	115072896	2.04935127	5.27277E-05	0.000178775
4348	ENSG00000277299.1			1.391926522	5.28429E-05	0.000179135
4349	ENSG00000261305.2			1.174432118	5.29369E-05	0.000179439
4350	ENSG00000242578.1			1.267953298	5.30705E-05	0.000179876
4351	ENSG00000223811.1			1.906050852	5.326E-05	0.00018041
4352	ENSG00000233817.1			1.135677312	5.35413E-05	0.000181348
4353	ENSG00000232212.1	LINC01701		2.531032897	5.3749E-05	0.000181989
4354	ENSG00000237161.4			1.73353165	5.38681E-05	0.000182377
4355	ENSG00000269729.1		105372424	1.495498369	5.40265E-05	0.000182851
4356	ENSG00000178809.11	TRIM73	375593	1.078578644	5.41625E-05	0.000183248
4357	ENSG00000211964.3	IGHV3-48		1.52117914	5.42909E-05	0.000183652
4358	ENSG00000278743.1			1.01675182	5.43246E-05	0.00018375
4359	ENSG00000265136.1			1.03539422	5.44345E-05	0.000184074
4360	ENSG00000063438.19	AHRR	57491	1.00267509	5.44867E-05	0.000184235
4361	ENSG00000218153.2	KRT18P22		1.36540586	5.46518E-05	0.000184778
4362	ENSG00000220267.1	ACTBP8		1.209221443	5.46565E-05	0.000184778
4363	ENSG00000213081.3	ARPC1BP1		2.254226599	5.51233E-05	0.000186261
4364	ENSG00000165566.12	AMER2	219287	1.888616389	5.52385E-05	0.000186591
4365	ENSG00000283033.1			1.520476022	5.52398E-05	0.000186591
4366	ENSG00000272115.1			1.463828897	5.59034E-05	0.000188688
4367	ENSG00000233381.3	AK4P3		1.542310492	5.62686E-05	0.000189823
4368	ENSG00000242435.1	UPK3BP1		1.674687582	5.64138E-05	0.000190257
4369	ENSG00000230601.7	TEX48	100505478	1.597322588	5.66475E-05	0.000190988
4370	ENSG00000241984.2	RPL7AP2		1.220364661	5.6912E-05	0.000191798
4371	ENSG00000258659.7	TRIM34	53840	1.10110282	5.70559E-05	0.000192267
4372	ENSG00000227617.9	CERS6-AS1	100861402	1.219312284	5.72188E-05	0.000192783
4373	ENSG00000206838.1	SNORA5A	654319	1.02040246	5.73723E-05	0.000193284
4374	ENSG00000188095.6	MESP2	145873	1.150110472	5.75532E-05	0.000193844
4375	ENSG00000100593.18	ISM2	145501	1.354582612	5.77083E-05	0.000194333
4376	ENSG00000253633.2	LINC03047		1.168906206	5.79642E-05	0.000195162
4377	ENSG00000234906.11	APOC2	344	1.625045305	5.81797E-05	0.00019587
4378	ENSG00000056291.17	NPFFR2	10886	2.265881944	5.82694E-05	0.000196156
4379	ENSG00000106133.18	NSUN5P2		1.106357428	5.8487E-05	0.000196805
4380	ENSG00000236519.1	LINC01424		1.004465796	5.87549E-05	0.000197576
4381	ENSG00000258294.5			2.745067446	5.8756E-05	0.000197576
4382	ENSG00000286426.1		105372042	2.475466388	5.8894E-05	0.000198006
4383	ENSG00000108849.8	PPY	5539	1.602127542	5.89612E-05	0.000198215
4384	ENSG00000267149.2			1.318527415	5.92738E-05	0.000199215
4385	ENSG00000170454.6	KRT75	9119	1.449257926	5.9424E-05	0.000199704
4386	ENSG00000211941.3	IGHV3-11		1.530295012	5.96906E-05	0.000200515
4387	ENSG00000100665.12	SERPINA4	5267	2.419480461	5.97354E-05	0.000200648
4388	ENSG00000164458.10	TBXT	6862	2.186681909	5.99193E-05	0.000201232
4389	ENSG00000260458.3	KCNJ18	100134444	1.652782161	6.00111E-05	0.000201506
4390	ENSG00000207445.1	SNORD15B	114599	1.049528662	6.01069E-05	0.00020181
4391	ENSG00000236182.1	ENOPH1P1		1.613643103	6.03407E-05	0.00020251
4392	ENSG00000287927.1		107986954	1.607283656	6.03409E-05	0.00020251
4393	ENSG00000145832.14	SLC25A48	153328	1.24203122	6.06972E-05	0.000203603
4394	ENSG00000233613.5	DCUN1D2-AS		1.252954162	6.11374E-05	0.00020494
4395	ENSG00000279206.1			1.528291516	6.14771E-05	0.000205957
4396	ENSG00000180316.12	PNPLA1	285848	1.290321462	6.2042E-05	0.000207684
4397	ENSG00000243323.7	PTPRVP		1.20927568	6.21103E-05	0.000207885
4398	ENSG00000255804.2	OR6J1	79549	1.637965881	6.21277E-05	0.000207926
4399	ENSG00000282897.1			1.621883925	6.22369E-05	0.000208239
4400	ENSG00000208037.1	MIR320A		1.535553466	6.25378E-05	0.000209127
4401	ENSG00000259823.6	LYPD8	646627	2.579920516	6.27021E-05	0.000209636
4402	ENSG00000241818.1	SCG5-AS1	105370757	1.303353717	6.30923E-05	0.00021087
4403	ENSG00000276712.1	MIR7111	102465668	1.128334872	6.32231E-05	0.000211253
4404	ENSG00000287460.1			1.816570141	6.38297E-05	0.000213083
4405	ENSG00000260815.3	PPIAP47		1.544961905	6.38493E-05	0.00021313
4406	ENSG00000257842.6	NOVA1-DT	102725045	2.619999044	6.38612E-05	0.000213152
4407	ENSG00000236878.1	MTATP6P26		1.494849993	6.40207E-05	0.00021363
4408	ENSG00000206028.1			1.661528986	6.40566E-05	0.000213732
4409	ENSG00000217482.2	HMGB1P17		1.803345844	6.46295E-05	0.000215499
4410	ENSG00000286208.1			2.87900141	6.47005E-05	0.000215699
4411	ENSG00000236986.6			1.405008821	6.50091E-05	0.000216655
4412	ENSG00000236890.2			1.540272712	6.53262E-05	0.000217693
4413	ENSG00000142319.18	SLC6A3	6531	1.426883465	6.56392E-05	0.000218645
4414	ENSG00000134259.5	NGF	4803	1.127126497	6.56584E-05	0.00021869
4415	ENSG00000205853.12	RFPL3S	10737	1.072463383	6.59886E-05	0.000219735
4416	ENSG00000260277.2		105371129	1.066055945	6.65523E-05	0.000221556
4417	ENSG00000156475.18	PPP2R2B	5521	1.105762387	6.66422E-05	0.000221818
4418	ENSG00000275882.1	IKBKGP1		1.023735739	6.67227E-05	0.000222067
4419	ENSG00000214300.7	SPDYE3	441272	1.280258477	6.6886E-05	0.000222566
4420	ENSG00000287692.1			1.508526074	6.71083E-05	0.000223276
4421	ENSG00000261723.1	PALB2-AS1		1.052874292	6.72566E-05	0.000223726
4422	ENSG00000254458.1			1.353096704	6.72606E-05	0.000223726
4423	ENSG00000166049.11	PASD1	139135	3.040871426	6.73222E-05	0.000223912
4424	ENSG00000202379.1	SNORA70	124900556	1.711260867	6.74085E-05	0.00022418
4425	ENSG00000259256.2	LINC01895		2.17785179	6.74934E-05	0.000224444
4426	ENSG00000258446.1			1.394062723	6.76432E-05	0.000224886
4427	ENSG00000227758.1	HCG9P5		1.67585272	6.79685E-05	0.000225906
4428	ENSG00000174279.4	EVX2	344191	2.300519169	6.8209E-05	0.000226672
4429	ENSG00000237167.1			1.546755319	6.827E-05	0.000226843
4430	ENSG00000221241.1	SNORD88A	692202	1.375496796	6.89535E-05	0.000228973
4431	ENSG00000224413.1			1.236905194	6.89863E-05	0.000229045
4432	ENSG00000249803.6	CTB-1I21.1	105379191	2.709220748	6.89914E-05	0.000229045
4433	ENSG00000224809.2	BEND3P2		2.294113876	6.90637E-05	0.000229243
4434	ENSG00000260908.1	PLA2G10CP		1.469074687	6.95648E-05	0.000230829
4435	ENSG00000287485.1		105375082	1.562081698	6.9695E-05	0.000231222
4436	ENSG00000119283.15	TRIM67	440730	1.015966281	6.97302E-05	0.00023132
4437	ENSG00000274895.1			1.84197282	6.97654E-05	0.000231398
4438	ENSG00000228874.1	RPS24P10		2.059481052	7.06249E-05	0.000234053
4439	ENSG00000263878.2	DLGAP1-AS4		2.154262245	7.09542E-05	0.000235085
4440	ENSG00000177455.15	CD19	930	1.267005582	7.13881E-05	0.000236463
4441	ENSG00000131142.13	CCL25	6370	1.517323108	7.18471E-05	0.000237865
4442	ENSG00000229522.1	LINC01523	101060004	2.468104635	7.1977E-05	0.000238255
4443	ENSG00000203581.7	OR1F2P	26184	1.783353463	7.23331E-05	0.000239334
4444	ENSG00000272362.1			1.520205074	7.23777E-05	0.000239461
4445	ENSG00000232591.2	LINC02642	105376388	1.187121843	7.252E-05	0.000239892
4446	ENSG00000198106.8			1.211822259	7.26763E-05	0.000240389
4447	ENSG00000215833.3	QRSL1P1		1.692754016	7.27218E-05	0.000240519
4448	ENSG00000272832.1			1.7003552	7.29119E-05	0.000241128
4449	ENSG00000117215.15	PLA2G2D	26279	1.490862867	7.30476E-05	0.000241537
4450	ENSG00000271329.1			1.752051282	7.3888E-05	0.000244071
4451	ENSG00000257454.1			2.139939861	7.42011E-05	0.000245079
4452	ENSG00000280107.1			1.008769947	7.42416E-05	0.000245158
4453	ENSG00000115596.4	WNT6	7475	1.038401547	7.44609E-05	0.000245861
4454	ENSG00000143297.19	FCRL5	83416	1.319009324	7.53329E-05	0.000248616
4455	ENSG00000285108.1			1.087158869	7.54225E-05	0.000248871
4456	ENSG00000286591.1			1.270753679	7.55206E-05	0.000249152
4457	ENSG00000285294.1	LINC00842	643650	1.007028798	7.55637E-05	0.000249274
4458	ENSG00000224533.4	TMLHE-AS1	100507404	1.440726039	7.58341E-05	0.000250104
4459	ENSG00000267255.1			1.616343129	7.58744E-05	0.000250216
4460	ENSG00000211669.3	IGLV3-10		1.62810279	7.58817E-05	0.000250219
4461	ENSG00000267673.6	FDX2	112812	1.458301916	7.61589E-05	0.000251112
4462	ENSG00000241207.1	SSX18P		2.563546925	7.63023E-05	0.000251522
4463	ENSG00000229291.1	LINC02768		1.156017708	7.67997E-05	0.000253078
4464	ENSG00000246863.2	GPR176-DT	100505534	1.235895979	7.74221E-05	0.000254917
4465	ENSG00000087495.17	PHACTR3	116154	1.241842001	7.77699E-05	0.000255998
4466	ENSG00000272727.2	LINC02266	105377466	1.958784111	7.78629E-05	0.000256283
4467	ENSG00000254507.2	DEFB131E		1.703081935	7.87228E-05	0.000258941
4468	ENSG00000137441.8	FGFBP2	83888	1.71662998	7.89585E-05	0.000259652
4469	ENSG00000286594.1			2.001466935	7.91867E-05	0.000260316
4470	ENSG00000269463.1			1.039809822	7.92626E-05	0.000260479
4471	ENSG00000253274.1	IGHV1-67		1.77207101	7.9364E-05	0.000260769
4472	ENSG00000215131.11	C16orf90	646174	1.790482898	7.94477E-05	0.000261023
4473	ENSG00000257681.1			1.02898693	7.94855E-05	0.000261125
4474	ENSG00000224611.1	TBPL1P1		1.487606532	7.95208E-05	0.00026122
4475	ENSG00000235990.2	RPL23AP20		1.672884022	7.95928E-05	0.000261434
4476	ENSG00000251301.7	LINC02384		1.049192406	8.02183E-05	0.000263336
4477	ENSG00000270822.1			1.036030407	8.09666E-05	0.000265683
4478	ENSG00000229774.1			2.810599015	8.10097E-05	0.000265802
4479	ENSG00000275401.1			1.142103152	8.1188E-05	0.000266321
4480	ENSG00000223650.1	UHRF2P1		1.548058462	8.16068E-05	0.000267587
4481	ENSG00000258679.1			1.587328743	8.16078E-05	0.000267587
4482	ENSG00000138684.9	IL21	59067	1.467717443	8.1704E-05	0.000267881
4483	ENSG00000231898.9	MYO3B-AS1		1.691396987	8.18509E-05	0.000268296
4484	ENSG00000260586.2			1.733406552	8.25804E-05	0.000270508
4485	ENSG00000268089.3	GABRQ	55879	1.152399863	8.26812E-05	0.000270776
4486	ENSG00000228157.4		102724624	1.46473772	8.26826E-05	0.000270776
4487	ENSG00000235237.1	IL2RB-AS1		1.62055611	8.28149E-05	0.000271142
4488	ENSG00000245526.11	MIR9-2HG	645323	1.681296055	8.28768E-05	0.000271322
4489	ENSG00000258404.1	LINC02320		1.310989144	8.30001E-05	0.000271693
4490	ENSG00000237529.2			1.26876313	8.30037E-05	0.000271693
4491	ENSG00000254088.1	SLC2A3P4		1.045791989	8.31536E-05	0.000272161
4492	ENSG00000215347.4	SLC25A5P1		1.085584838	8.36621E-05	0.00027378
4493	ENSG00000204613.11	TRIM10	10107	1.497687784	8.44428E-05	0.000276221
4494	ENSG00000264188.1			1.067911057	8.64681E-05	0.00028238
4495	ENSG00000251639.2			1.774849378	8.65244E-05	0.000282528
4496	ENSG00000267506.5			1.560514258	8.67166E-05	0.000283075
4497	ENSG00000269945.1			1.904979131	8.68964E-05	0.000283638
4498	ENSG00000189167.12	ZAR1L	646799	1.544026421	8.69848E-05	0.000283871
4499	ENSG00000156150.9	ALX3	257	2.511843613	8.69891E-05	0.000283871
4500	ENSG00000244757.1			1.684291348	8.71561E-05	0.000284392
4501	ENSG00000106384.12	MOGAT3	346606	1.892437898	8.7391E-05	0.000285075
4502	ENSG00000260549.2	MT1L		1.120762525	8.78091E-05	0.000286358
4503	ENSG00000253256.1			1.029291132	8.84713E-05	0.000288352
4504	ENSG00000203411.3	Metazoa_SRP		1.849975463	8.92714E-05	0.000290792
4505	ENSG00000240535.8			1.511060568	9.04516E-05	0.00029425
4506	ENSG00000287275.1			1.405919817	9.11156E-05	0.000296264
4507	ENSG00000271659.1			1.333647727	9.14735E-05	0.000297355
4508	ENSG00000253475.1			1.653762654	9.15597E-05	0.000297586
4509	ENSG00000132958.17	TPTE2	93492	1.299474475	9.16216E-05	0.000297738
4510	ENSG00000286741.1			1.503701123	9.16755E-05	0.000297865
4511	ENSG00000239951.1	IGKV3-20		1.362882198	9.16832E-05	0.000297865
4512	ENSG00000248483.7	POU5F2	134187	1.175540804	9.17775E-05	0.000298147
4513	ENSG00000216775.3			1.167470883	9.1807E-05	0.000298218
4514	ENSG00000284699.1			1.538174787	9.3376E-05	0.000302918
4515	ENSG00000130294.17	KIF1A	547	1.616855788	9.38537E-05	0.000304318
4516	ENSG00000253720.2			1.137499891	9.38976E-05	0.000304436
4517	ENSG00000229401.1			1.363847001	9.40308E-05	0.000304818
4518	ENSG00000226261.1	PIMREGP1		1.428807949	9.41962E-05	0.000305304
4519	ENSG00000267298.1			1.074468591	9.43443E-05	0.000305734
4520	ENSG00000272461.1			2.228489457	9.43777E-05	0.000305818
4521	ENSG00000287214.1			1.048443444	9.46556E-05	0.000306693
4522	ENSG00000188263.10	IL17REL	400935	1.584791442	9.47262E-05	0.000306896
4523	ENSG00000243415.2			1.164015231	9.48644E-05	0.000307319
4524	ENSG00000253326.2			1.143814967	9.49558E-05	0.00030759
4525	ENSG00000211934.3	IGHV1-2		1.537672788	9.55106E-05	0.000309337
4526	ENSG00000228286.3			3.124444289	9.57543E-05	0.000309999
4527	ENSG00000236528.1			1.415578757	9.63777E-05	0.000311941
4528	ENSG00000256448.5			1.170879781	9.6509E-05	0.000312315
4529	ENSG00000252192.1			1.543950006	9.68493E-05	0.000313365
4530	ENSG00000126266.3	FFAR1	2864	2.03202876	9.68975E-05	0.000313496
4531	ENSG00000272533.1	SNORA28	677811	1.515265743	9.72006E-05	0.000314374
4532	ENSG00000213587.3			1.508698621	9.72442E-05	0.000314489
4533	ENSG00000271215.1	RPL21P136		1.463525683	9.73147E-05	0.000314691
4534	ENSG00000279485.1			2.778387959	9.74953E-05	0.000315224
4535	ENSG00000287575.1		101927712	1.358933743	9.78033E-05	0.000316168
4536	ENSG00000286294.1			1.234564208	9.79202E-05	0.00031652
4537	ENSG00000218048.2			2.528932806	9.79703E-05	0.000316656
4538	ENSG00000235427.1			1.558965782	9.81545E-05	0.000317148
4539	ENSG00000287030.1			1.313716408	9.88027E-05	0.000319139
4540	ENSG00000221986.7	MYBPHL	343263	1.304055379	9.90397E-05	0.000319878
4541	ENSG00000279078.1	SND1-IT1	27099	1.324528951	9.93417E-05	0.000320828
4542	ENSG00000272094.1			2.378076948	9.99364E-05	0.000322669
4543	ENSG00000174963.18	ZIC4	84107	1.777870228	0.000100658	0.000324841
4544	ENSG00000136573.14	BLK	640	1.266651113	0.000100931	0.000325669
4545	ENSG00000241217.3	RN7SL809P		1.181023011	0.000100975	0.000325756
4546	ENSG00000265646.2	TUFMP1		1.656146299	0.000101197	0.000326422
4547	ENSG00000247763.2	TUBAP14		1.0736768	0.000101267	0.000326621
4548	ENSG00000104827.12	CGB3	1082	2.147796043	0.000101631	0.000327739
4549	ENSG00000183171.5			1.139316721	0.000101705	0.000327952
4550	ENSG00000275232.1			1.946664788	0.000101912	0.000328514
4551	ENSG00000225675.2	LRP8-DT		1.484137456	0.000102596	0.000330477
4552	ENSG00000270354.1			1.261553309	0.000102783	0.000331053
4553	ENSG00000237200.1	ZBTB40-IT1		1.193067619	0.000102976	0.000331566
4554	ENSG00000244437.1	IGKV3-15		1.375623603	0.000103447	0.000332999
4555	ENSG00000223350.2	IGLV9-49		1.73595291	0.000103488	0.000333107
4556	ENSG00000237418.1			1.222259344	0.000103672	0.00033359
4557	ENSG00000258121.1			1.379381052	0.000104088	0.000334847
4558	ENSG00000258525.2		100506071	1.761765877	0.000104633	0.000336545
4559	ENSG00000149972.11	CNTN5	53942	1.539225669	0.000104728	0.000336822
4560	ENSG00000198857.3	HSD3BP5		1.010618617	0.000105098	0.000337905
4561	ENSG00000264513.1			1.39495146	0.00010516	0.000338049
4562	ENSG00000249731.1			1.485509037	0.00010647	0.000341982
4563	ENSG00000232712.6	KIZ-AS1	101929591	1.081021924	0.000106493	0.000342001
4564	ENSG00000166104.15			2.031306132	0.000106696	0.000342596
4565	ENSG00000284930.1			1.618907662	0.000106724	0.000342659
4566	ENSG00000235308.1			1.156598619	0.000106795	0.000342804
4567	ENSG00000241388.5	HNF1A-AS1	283460	1.513861922	0.000107077	0.000343652
4568	ENSG00000201499.1	RNU6-312P		1.395034866	0.00010716	0.000343891
4569	ENSG00000250654.7			1.147906338	0.00010736	0.000344449
4570	ENSG00000015413.9	DPEP1	1800	1.098535402	0.000107375	0.000344471
4571	ENSG00000277794.1	Metazoa_SRP		1.406576005	0.000107847	0.000345844
4572	ENSG00000166035.11	LIPC	3990	1.158535758	0.000107898	0.000345982
4573	ENSG00000236463.1	LINC00427	100507040	1.519528787	0.000108171	0.000346799
4574	ENSG00000224807.5	DUX4L9		1.740744892	0.000108191	0.000346835
4575	ENSG00000185758.9	CLDN24	100132463	1.202917426	0.000108539	0.000347811
4576	ENSG00000233215.6	LINC01687	101927843	3.228706213	0.000108809	0.000348646
4577	ENSG00000228358.6			2.086912173	0.000108905	0.000348815
4578	ENSG00000232211.1			2.487455552	0.000109059	0.000349281
4579	ENSG00000105549.11	SPMAP2	51298	1.161861587	0.00010916	0.000349545
4580	ENSG00000225851.1	HLA-S		1.283889969	0.000109232	0.00034972
4581	ENSG00000260066.1			1.786538297	0.000109503	0.000350559
4582	ENSG00000204709.4	LINC01556		1.347320936	0.000110387	0.000353191
4583	ENSG00000236908.2	LINC02827		1.150021882	0.000110952	0.000354855
4584	ENSG00000183145.9	RIPPLY3	53820	1.278737451	0.000111142	0.000355378
4585	ENSG00000264772.6			1.368473258	0.000111346	0.00035597
4586	ENSG00000268581.1	SIGLEC18P		1.416122496	0.000111732	0.000357121
4587	ENSG00000278153.1			1.184096512	0.000112144	0.00035835
4588	ENSG00000275139.1			1.86750917	0.00011232	0.000358853
4589	ENSG00000261024.7			1.387287951	0.000112518	0.00035941
4590	ENSG00000229526.2	KRT16P4		1.063189486	0.000113504	0.000362374
4591	ENSG00000250432.5	FAM242C		1.662762722	0.000113686	0.000362897
4592	ENSG00000276292.1			1.143721015	0.00011383	0.000363298
4593	ENSG00000144227.5	NXPH2	11249	1.886501494	0.000113865	0.000363349
4594	ENSG00000261471.1			1.554678295	0.000114427	0.000364998
4595	ENSG00000231270.1	COBLP1		1.956006258	0.000114604	0.000365418
4596	ENSG00000236527.1	ARF4P2		1.533478141	0.000114763	0.000365864
4597	ENSG00000215057.4	PKMP5		1.073035235	0.000115103	0.000366918
4598	ENSG00000255445.1			1.161658433	0.000115238	0.000367318
4599	ENSG00000250948.1			1.403837188	0.000115476	0.000367989
4600	ENSG00000237566.1	CD24P5		2.434021747	0.000116147	0.000370007
4601	ENSG00000217455.8		105375130	1.49443344	0.000116424	0.000370802
4602	ENSG00000249022.2			1.230833204	0.000116754	0.000371792
4603	ENSG00000183128.7	CALHM3	119395	1.579508295	0.000117111	0.000372868
4604	ENSG00000278893.1			1.655540995	0.000117156	0.000372982
4605	ENSG00000285599.1			1.562868674	0.000117891	0.000375142
4606	ENSG00000241983.3	RN7SL566P		1.145154113	0.000118042	0.000375593
4607	ENSG00000230183.4	CNOT6LP1		1.151845818	0.000118223	0.000376076
4608	ENSG00000249721.1			1.326388047	0.000118306	0.000376281
4609	ENSG00000275328.1	SPDYE19P		1.413572315	0.000118519	0.000376898
4610	ENSG00000242472.1	IGHJ5		1.707096794	0.000118931	0.000378088
4611	ENSG00000170683.6	OR10A3	26496	1.707784211	0.000119059	0.000378434
4612	ENSG00000145198.14	VWA5B2	90113	1.04176504	0.00011993	0.000381049
4613	ENSG00000232814.2	COL4A2-AS1	100874203	1.393638644	0.000120479	0.000382732
4614	ENSG00000268606.5	MAGEA2	4101	3.210621257	0.000121429	0.000385565
4615	ENSG00000172073.4	SPMIP9	200523	2.033453397	0.000121575	0.000385965
4616	ENSG00000279328.1		124906280	1.127708313	0.000121785	0.000386602
4617	ENSG00000270068.1			1.42007291	0.000121836	0.000386733
4618	ENSG00000259337.4	IGHV1OR15-2		1.656415662	0.000122231	0.000387863
4619	ENSG00000279670.1			1.142462876	0.000122262	0.000387928
4620	ENSG00000242076.2	IGKV1-33		1.6739013	0.000122512	0.00038869
4621	ENSG00000234471.1			1.878647804	0.000122829	0.000389604
4622	ENSG00000262521.1			2.142516216	0.000122995	0.000390036
4623	ENSG00000273720.1			1.491580086	0.000123084	0.000390289
4624	ENSG00000242766.1	IGKV1D-17		1.857718182	0.000123403	0.000391144
4625	ENSG00000236003.1			1.794549623	0.000123938	0.000392807
4626	ENSG00000280269.1			1.179101856	0.000124011	0.000393008
4627	ENSG00000287785.1			2.163467335	0.000124067	0.000393153
4628	ENSG00000231292.6	IGKV1OR2-108		1.412700525	0.000124095	0.000393178
4629	ENSG00000147465.12	STAR	6770	1.01986114	0.000125102	0.000395989
4630	ENSG00000266265.4	KLF14	136259	1.137429516	0.00012608	0.000398989
4631	ENSG00000244485.1	RPL18P13		1.561424188	0.000126319	0.000399683
4632	ENSG00000174448.8	STARD6	147323	1.372000644	0.00012662	0.000400604
4633	ENSG00000158815.11	FGF17	8822	1.522669017	0.000127129	0.000402116
4634	ENSG00000277738.1			2.593776596	0.000128321	0.000405693
4635	ENSG00000275620.1	FLJ16779	100192386	1.264042552	0.000128332	0.000405695
4636	ENSG00000261250.1			1.883075289	0.000128687	0.000406656
4637	ENSG00000235351.1			1.028507858	0.000129265	0.000408353
4638	ENSG00000228966.2	HOMER2P1		3.052641893	0.00012984	0.000409964
4639	ENSG00000213302.3	RPL31P37		1.543500276	0.00013014	0.000410823
4640	ENSG00000226629.1	LINC00974	147093	1.779646092	0.000130456	0.000411785
4641	ENSG00000138161.14	CUZD1	50624	1.468413677	0.000131138	0.000413674
4642	ENSG00000100024.15	UPB1	51733	1.329393195	0.00013121	0.00041387
4643	ENSG00000147614.4	ATP6V0D2	245972	1.002285963	0.000131694	0.000415332
4644	ENSG00000278842.1			1.522894454	0.000132452	0.000417555
4645	ENSG00000280953.2	LINC01163	101927381	1.910745297	0.00013256	0.000417861
4646	ENSG00000257256.1			1.454765812	0.000132654	0.000418126
4647	ENSG00000266720.2	RN7SL14P		2.261703791	0.000133216	0.00041973
4648	ENSG00000278698.1			2.317619075	0.00013362	0.000420937
4649	ENSG00000250929.2	LINC01181		1.732639479	0.000133788	0.000421431
4650	ENSG00000266579.1			1.2253606	0.000133856	0.000421579
4651	ENSG00000226148.1	SLC25A39P1		1.571362239	0.000134037	0.000422114
4652	ENSG00000239096.1		124906348	1.562767943	0.000134244	0.000422706
4653	ENSG00000236049.1	LINC01920	101929260	1.719899571	0.000134246	0.000422706
4654	ENSG00000259803.7	SLC22A31	146429	1.249964208	0.000134461	0.000423329
4655	ENSG00000248926.1			2.07270696	0.000134623	0.000423743
4656	ENSG00000286801.1			1.839425788	0.000134629	0.000423743
4657	ENSG00000205584.6	FKBP6P1		1.795331435	0.000134779	0.000424183
4658	ENSG00000229494.2		101927948	1.755643507	0.000135222	0.000425455
4659	ENSG00000243514.2	RPL32P33		1.373711275	0.000135226	0.000425455
4660	ENSG00000242536.2			1.851519393	0.000135444	0.000426107
4661	ENSG00000233009.1	NALCN-AS1	100885778	1.601643854	0.000135922	0.000427508
4662	ENSG00000235601.1	BARX1-DT	101928040	1.467649972	0.000135977	0.000427646
4663	ENSG00000286145.2			1.246564323	0.000136073	0.000427914
4664	ENSG00000272170.1	KLC4-AS1		1.096176774	0.000136159	0.000428151
4665	ENSG00000249162.1	ADAM20P3		2.604598493	0.000136953	0.000430443
4666	ENSG00000279864.2	NCOR1P4		3.627004163	0.000137517	0.000432113
4667	ENSG00000257139.1	LINC02821		1.528970294	0.000137535	0.000432138
4668	ENSG00000188933.16	USP32P1		1.430444877	0.00013761	0.000432298
4669	ENSG00000253821.2			1.67087077	0.000138653	0.00043534
4670	ENSG00000255980.1	LINC02953	102724265	2.766602452	0.000138832	0.000435866
4671	ENSG00000287799.1			1.409237695	0.000140246	0.000440062
4672	ENSG00000254406.1			2.114799186	0.000140898	0.000442038
4673	ENSG00000232613.6	LINC02576	102724233	1.204561925	0.000141518	0.000443877
4674	ENSG00000211593.2	IGKJ5		1.557131838	0.000141545	0.00044389
4675	ENSG00000287213.1			1.292765421	0.000142558	0.000446572
4676	ENSG00000266830.1			2.684778626	0.000143082	0.000448038
4677	ENSG00000241351.3	IGKV3-11		1.349705981	0.000143114	0.000448079
4678	ENSG00000225166.2		102724849	2.046968783	0.000143118	0.000448079
4679	ENSG00000284820.1			1.281699276	0.000143407	0.000448948
4680	ENSG00000233445.1	RPL17P11		2.092091034	0.000144024	0.000450682
4681	ENSG00000259294.1			1.459263468	0.000144029	0.000450682
4682	ENSG00000283433.1	RNA5SP532		1.455121502	0.000144802	0.000452889
4683	ENSG00000287913.1			1.510663759	0.00014492	0.000453222
4684	ENSG00000187140.6	FOXD3	27022	1.238939371	0.000144981	0.000453377
4685	ENSG00000270133.1			1.060655041	0.000145238	0.000454108
4686	ENSG00000276058.1	STMN1P1		1.278794747	0.000145721	0.00045551
4687	ENSG00000267515.1			1.513373309	0.000145767	0.000455618
4688	ENSG00000143340.6	FAM163A	148753	1.211703949	0.00014587	0.000455903
4689	ENSG00000261319.1	LINC02152		3.616989285	0.000146092	0.0004565
4690	ENSG00000239670.1			1.400119519	0.000147945	0.000461878
4691	ENSG00000101405.3	OXT	5020	1.260007363	0.000148016	0.000462064
4692	ENSG00000273687.1			1.045093434	0.000148309	0.000462906
4693	ENSG00000280042.1			1.070560507	0.000148463	0.000463278
4694	ENSG00000279601.1			1.488758533	0.000149094	0.000465028
4695	ENSG00000268266.1	PKMYT1AR		1.354803596	0.000149729	0.000466897
4696	ENSG00000135406.14	PRPH	5630	1.198801081	0.000150478	0.000469012
4697	ENSG00000231704.6			1.092077979	0.000150625	0.000469401
4698	ENSG00000202461.1	Y_RNA		1.384488976	0.00015196	0.000473333
4699	ENSG00000273363.1			1.397194435	0.000152185	0.000473961
4700	ENSG00000258558.1			1.368131916	0.00015228	0.000474218
4701	ENSG00000189325.7	BNIP5	389384	1.327345654	0.000152693	0.000475391
4702	ENSG00000256262.1	USP30-AS1		1.034209463	0.000152755	0.00047555
4703	ENSG00000142698.15	C1orf94	84970	1.861832478	0.000153325	0.000477135
4704	ENSG00000167754.13	KLK5	25818	1.393084256	0.0001536	0.000477916
4705	ENSG00000235221.3	LINC00383		3.426611427	0.000154027	0.000479131
4706	ENSG00000250497.1			2.142709976	0.000154224	0.000479706
4707	ENSG00000196433.13	ASMT	438	1.09126509	0.000154958	0.000481765
4708	ENSG00000211592.8	IGKC		1.291986802	0.000155409	0.00048309
4709	ENSG00000253500.6			2.356227383	0.000157431	0.000488952
4710	ENSG00000249973.2	CHCHD2P7		1.930063166	0.000157998	0.000490637
4711	ENSG00000258123.1	LINC02444	101928137	2.426394971	0.000158212	0.000491149
4712	ENSG00000268316.1			1.216175324	0.000158551	0.000492125
4713	ENSG00000161973.11	CCDC42	146849	1.104142664	0.000158567	0.000492134
4714	ENSG00000231290.6	APCDD1L-DT	149773	1.175427364	0.000158638	0.000492316
4715	ENSG00000160951.4	PTGER1	5731	1.167425615	0.00015868	0.000492408
4716	ENSG00000278716.1			1.543102517	0.000158893	0.0004928
4717	ENSG00000236530.2	KPNA2P1		1.517457009	0.000159391	0.000494306
4718	ENSG00000007038.11	PRSS21	10942	1.501628844	0.000160105	0.000496364
4719	ENSG00000266553.2	RN7SL356P		1.946722124	0.000160696	0.000498119
4720	ENSG00000186645.10	SPDYE17	102723849	1.417542035	0.000160786	0.000498358
4721	ENSG00000241697.5	TMEFF1	8577	1.647312399	0.000161078	0.000499226
4722	ENSG00000259977.1			2.913759934	0.000161196	0.000499552
4723	ENSG00000184434.8	LRRC19	64922	1.097275848	0.000161326	0.000499876
4724	ENSG00000260744.1			1.670315146	0.000161794	0.000501209
4725	ENSG00000146166.17	LGSN	51557	1.862445046	0.000163475	0.000506259
4726	ENSG00000280776.2	LINC01202		2.865995101	0.000164533	0.000509216
4727	ENSG00000288021.1			1.500932767	0.000165035	0.000510611
4728	ENSG00000225751.2			1.344923485	0.000165283	0.000511339
4729	ENSG00000266913.1	LINC01841		1.191581993	0.000165636	0.00051231
4730	ENSG00000205108.6	SPATA31F1	259308	1.32319983	0.000166099	0.000513621
4731	ENSG00000260896.6	ARLNC1	100996425	1.940664127	0.00016692	0.00051592
4732	ENSG00000248515.2			1.870861312	0.000167181	0.000516605
4733	ENSG00000211640.4	IGLV6-57		1.398472522	0.00016756	0.000517573
4734	ENSG00000287423.1			1.540559564	0.000167613	0.000517658
4735	ENSG00000271811.1			1.098184457	0.000168072	0.000518955
4736	ENSG00000124557.12	BTN1A1	696	1.260257506	0.000168321	0.000519682
4737	ENSG00000279175.1			1.24759148	0.000168374	0.000519805
4738	ENSG00000220884.2	MESTP1		1.384267242	0.000169138	0.000522003
4739	ENSG00000214843.3	ZFYVE9P2		1.580604505	0.000169393	0.000522748
4740	ENSG00000248510.2	LINC02267		3.615422319	0.000169722	0.000523682
4741	ENSG00000253692.3	IGHEP1		1.689015894	0.000170302	0.000525348
4742	ENSG00000229981.8	LINC01435	103695365	2.132460282	0.000170988	0.000527176
4743	ENSG00000164256.11	PRDM9	56979	2.333142432	0.000172484	0.000531418
4744	ENSG00000270813.2	NANOGNBP3		1.059808729	0.000173298	0.000533761
4745	ENSG00000255091.1			1.755337824	0.000173936	0.000535592
4746	ENSG00000164729.7	SLC35G3	146861	1.710930752	0.00017481	0.000537999
4747	ENSG00000288082.1		124908011	1.770436284	0.000175338	0.00053933
4748	ENSG00000261273.1			1.459514824	0.000177537	0.000545714
4749	ENSG00000188676.13	IDO2	169355	1.007030032	0.00017775	0.000546325
4750	ENSG00000268649.5			1.683158057	0.000178128	0.000547362
4751	ENSG00000243772.8	KIR2DL3	3804	1.476631128	0.000178168	0.000547442
4752	ENSG00000234622.6			1.837778415	0.000178759	0.000549088
4753	ENSG00000257037.1	RARS1P1		1.451954742	0.000179677	0.000551692
4754	ENSG00000188620.11	HMX3	340784	2.13175952	0.00018004	0.000552723
4755	ENSG00000259156.7	CHEK2P2		2.567145006	0.000180194	0.00055311
4756	ENSG00000267413.1	LINC01901		2.645624074	0.000180355	0.000553518
4757	ENSG00000272729.1			1.396091197	0.00018189	0.000557927
4758	ENSG00000284879.1	NCAL1		1.023986796	0.000182454	0.000559613
4759	ENSG00000250039.4			1.247196873	0.00018271	0.000560312
4760	ENSG00000230162.1	CT45A11P		1.111280214	0.000183109	0.000561448
4761	ENSG00000265908.1			1.455542752	0.000183403	0.000562305
4762	ENSG00000262380.1			1.125699137	0.000184196	0.000564606
4763	ENSG00000270429.1	KNOP1P2		1.313058254	0.000184684	0.00056597
4764	ENSG00000240864.3	IGKV1-16		1.483573397	0.000185674	0.000568874
4765	ENSG00000241449.6			1.921974193	0.000185899	0.00056952
4766	ENSG00000161031.13	PGLYRP2	114770	1.221310865	0.000188256	0.000576206
4767	ENSG00000285849.1			1.219934815	0.000188344	0.000576429
4768	ENSG00000253121.1			1.602552309	0.000188512	0.000576874
4769	ENSG00000226107.1	RPL36AP54		1.035465348	0.000188518	0.000576874
4770	ENSG00000243290.3	IGKV1-12		1.403973222	0.000189444	0.000579527
4771	ENSG00000181790.11	ADGRB1	575	1.111397132	0.000190275	0.000581755
4772	ENSG00000277049.1	RNA5SP530		1.23058213	0.000193396	0.000590571
4773	ENSG00000286501.1			1.312750905	0.000193675	0.00059124
4774	ENSG00000152591.15	DSPP	1834	1.463655868	0.000195227	0.000595564
4775	ENSG00000275875.1			1.766719851	0.000195941	0.000597606
4776	ENSG00000224060.3	ARSLP1		1.855500371	0.000197263	0.000601499
4777	ENSG00000272689.1			1.108904903	0.000198271	0.000604387
4778	ENSG00000199890.1	Y_RNA		1.546612972	0.000198329	0.000604511
4779	ENSG00000230534.6			1.255357285	0.000198342	0.000604511
4780	ENSG00000211966.2	IGHV5-51		1.404816267	0.000198377	0.000604568
4781	ENSG00000228636.3	LINC02663	124902542	2.666890255	0.000198882	0.000605969
4782	ENSG00000229312.2			1.498211032	0.000200403	0.00061032
4783	ENSG00000211749.1	TRBV23-1		1.697666622	0.000202372	0.000615845
4784	ENSG00000259683.1			1.033626632	0.000202499	0.000616138
4785	ENSG00000266975.1	FARSA-AS1	106144598	1.387254786	0.000203166	0.000618073
4786	ENSG00000268480.3	NFILZ	105372267	1.937634493	0.000203274	0.000618306
4787	ENSG00000269836.1			1.246354937	0.000204309	0.000621407
4788	ENSG00000232846.1	SLC25A6P3		2.833908316	0.000206358	0.000627205
4789	ENSG00000102021.11	LUZP4	51213	2.35700664	0.000208257	0.000632686
4790	ENSG00000226472.8			1.073530289	0.000208307	0.000632789
4791	ENSG00000273816.1			1.336812422	0.000208363	0.000632861
4792	ENSG00000211651.3	IGLV1-44		1.379680001	0.000208644	0.00063357
4793	ENSG00000280183.1			1.097618415	0.000210238	0.000637824
4794	ENSG00000275465.7		158434	2.485370086	0.000211113	0.000640331
4795	ENSG00000285753.1	LCEP3		1.663687811	0.0002112	0.000640547
4796	ENSG00000267046.1	E2F3P1		1.004718923	0.000211273	0.000640718
4797	ENSG00000287434.1			1.512008034	0.000212348	0.000643782
4798	ENSG00000270095.1			1.473572548	0.000212552	0.000644351
4799	ENSG00000245688.1			1.77065814	0.000213778	0.000647573
4800	ENSG00000211937.3	IGHV2-5		1.462592633	0.000213859	0.000647769
4801	ENSG00000162692.12	VCAM1	7412	1.07466775	0.00021408	0.000648339
4802	ENSG00000265962.1	GACAT2		1.363160304	0.000214932	0.000650771
4803	ENSG00000253452.6		124902062	2.028484901	0.00021554	0.000652411
4804	ENSG00000235946.1	H2BW3P		1.653902246	0.000216295	0.000654598
4805	ENSG00000172969.7	FRG2C	100288801	2.028227094	0.000217167	0.000657086
4806	ENSG00000168925.12	CTRB1	1504	1.594443244	0.000217523	0.000658012
4807	ENSG00000250822.1	LINC02111	401177	1.908414465	0.00021934	0.000663306
4808	ENSG00000189064.8	GAGE2A	26749	5.364820582	0.000219933	0.000665018
4809	ENSG00000227744.4	LINC01940	401039	1.471779064	0.000221484	0.000669331
4810	ENSG00000188848.16	BEND4	389206	1.418315105	0.000222197	0.000671382
4811	ENSG00000200953.1	Y_RNA		1.391999574	0.00022246	0.000672127
4812	ENSG00000228019.1			1.261482137	0.000222635	0.000672604
4813	ENSG00000211637.2	IGLV4-69		1.472257587	0.000223165	0.000674154
4814	ENSG00000176571.12	CNBD1	168975	2.217115537	0.000223683	0.000675564
4815	ENSG00000207004.1	RNU6-301P		1.354205451	0.000223843	0.000675995
4816	ENSG00000203995.10	ZYG11A	440590	1.138815699	0.00022616	0.000682681
4817	ENSG00000236242.1	MYO16-AS1	100885782	1.165234989	0.000226334	0.000683102
4818	ENSG00000279067.1			1.728435027	0.000226936	0.000684657
4819	ENSG00000234345.2	ELF2P1		1.54335514	0.000227246	0.000685491
4820	ENSG00000225796.2	MTND4P23		1.519599192	0.000227938	0.000687265
4821	ENSG00000216754.2	RPL6P17		1.948031926	0.00022857	0.000689064
4822	ENSG00000259469.1			1.286239194	0.000230147	0.00069345
4823	ENSG00000243902.6	ELFN2	114794	1.868808192	0.000230991	0.000695727
4824	ENSG00000256948.1	IQSEC3-AS2		1.475927337	0.000231828	0.00069809
4825	ENSG00000259125.1	LRP1-AS	105751187	1.387006307	0.000234516	0.000705754
4826	ENSG00000254294.1	IMPDH1P6		1.052495875	0.000235212	0.000707796
4827	ENSG00000255223.4	OR5M11	219487	1.864862166	0.000235829	0.000709384
4828	ENSG00000259099.2			1.740848146	0.000237235	0.000713289
4829	ENSG00000287414.1			2.077262275	0.000237425	0.000713806
4830	ENSG00000237872.4	POU5F1P4	645682	1.450347912	0.000238063	0.000715616
4831	ENSG00000167798.17	C3P1		2.042683605	0.000239205	0.00071894
4832	ENSG00000239480.1			1.017963387	0.000239836	0.00072078
4833	ENSG00000215910.7	C1orf167	284498	1.581651822	0.000240143	0.000721594
4834	ENSG00000235621.9	LINC00494	284749	1.376559451	0.000240415	0.000722356
4835	ENSG00000183889.12	NPIPA6	131675794	1.324839822	0.000240589	0.000722825
4836	ENSG00000287959.1			1.830099018	0.000241988	0.000726643
4837	ENSG00000232325.4			1.681365298	0.000242029	0.000726711
4838	ENSG00000104938.18	CLEC4M	10332	1.346637145	0.000242921	0.000729219
4839	ENSG00000224273.2	RABGEF1P3		1.335475095	0.000244802	0.000734423
4840	ENSG00000211677.2	IGLC2		1.21376938	0.00024502	0.000735022
4841	ENSG00000248685.6	LINC02484		3.597711241	0.000245605	0.000736602
4842	ENSG00000164532.11	TBX20	57057	2.526205344	0.000246058	0.000737745
4843	ENSG00000258153.1	HSPE1P4		1.196869855	0.0002462	0.000738059
4844	ENSG00000211780.3	TRAV6		1.237983712	0.000246376	0.000738532
4845	ENSG00000275549.1	STPG3-AS1	100129722	1.247075665	0.000247528	0.000741817
4846	ENSG00000226088.1	LINC03102		1.413006146	0.000247714	0.000742317
4847	ENSG00000255133.2	LINC02721		1.306249605	0.000248122	0.000743486
4848	ENSG00000287249.1			1.101428343	0.000248342	0.000744088
4849	ENSG00000185668.8	POU3F1	5453	1.00430208	0.000248875	0.000745572
4850	ENSG00000129991.13	TNNI3	7137	1.274099784	0.00024908	0.000746131
4851	ENSG00000274525.1			1.49996791	0.000250065	0.000748968
4852	ENSG00000137674.4	MMP20	9313	1.613768047	0.000250108	0.000749039
4853	ENSG00000203783.5	PRR9	574414	1.72997797	0.000251487	0.000752942
4854	ENSG00000205035.8	GRM5P1		2.284051118	0.000252429	0.000755647
4855	ENSG00000251163.1	PRELID3BP4		2.045440992	0.000252549	0.000755949
4856	ENSG00000235111.1			1.307678208	0.000252753	0.000756504
4857	ENSG00000185053.14	SGCZ	137868	2.2310943	0.000253012	0.000757223
4858	ENSG00000262298.1			1.078891544	0.000253523	0.000758636
4859	ENSG00000199846.1	RNU1-72P		1.552230697	0.000254121	0.000760311
4860	ENSG00000256394.3	ASIC5	51802	1.984873388	0.000254272	0.000760704
4861	ENSG00000272948.2			1.097321596	0.000254365	0.000760926
4862	ENSG00000254997.4	KRTAP5-9	3846	1.204138159	0.000254664	0.000761763
4863	ENSG00000129437.11	KLK14	43847	1.5195586	0.000254754	0.000761976
4864	ENSG00000287796.1			2.275609386	0.000255116	0.000762964
4865	ENSG00000259555.1			1.629448478	0.000255153	0.000762996
4866	ENSG00000224764.1	AOPEP-AS1		1.408223617	0.000255553	0.000764078
4867	ENSG00000266117.1	FBXO36P1		1.665419043	0.000255619	0.00076416
4868	ENSG00000267559.5			1.211661664	0.000256687	0.000767179
4869	ENSG00000237849.1	NFYAP1		2.414101843	0.00025723	0.000768568
4870	ENSG00000125798.15	FOXA2	3170	1.501223343	0.00025811	0.000770965
4871	ENSG00000279085.1			1.437227398	0.000258978	0.000773385
4872	ENSG00000277968.1			1.417805478	0.000260263	0.000776811
4873	ENSG00000196844.9	PATE2	399967	1.607961647	0.000260315	0.000776849
4874	ENSG00000241666.2			1.179187323	0.000260716	0.000777989
4875	ENSG00000171864.5	PRND	23627	1.138793249	0.000260959	0.000778654
4876	ENSG00000226937.9	CEP164P1		1.206681318	0.0002621	0.000781943
4877	ENSG00000282977.1	PCBP2-OT1	102157401	1.169655241	0.00026223	0.000782272
4878	ENSG00000261231.6	LINC02133	101927132	1.398726767	0.000262486	0.000782858
4879	ENSG00000226632.1	UBE2V1P1		1.286338421	0.000263499	0.000785582
4880	ENSG00000188029.8	CTSL3P		1.98023782	0.000263538	0.000785599
4881	ENSG00000231047.1	GCNT1P3		1.332092692	0.000263564	0.000785599
4882	ENSG00000253775.2		105379311	2.17307489	0.000263704	0.000785959
4883	ENSG00000261924.1		400627	1.009423166	0.000263864	0.000786376
4884	ENSG00000272810.1			1.284541534	0.000265455	0.000790819
4885	ENSG00000279607.1			2.091271142	0.000266629	0.000794139
4886	ENSG00000128276.10	RFPL3	10738	1.790993727	0.000267929	0.000797712
4887	ENSG00000236489.1	RPL34P10		1.460509802	0.00026807	0.000798011
4888	ENSG00000254174.1	IGHV1-12		1.677786842	0.000268813	0.000800044
4889	ENSG00000288530.1			1.80109862	0.000268985	0.000800491
4890	ENSG00000287991.1			1.639253324	0.000269004	0.000800491
4891	ENSG00000251303.1	CAB39P1		1.103976698	0.000269319	0.000801247
4892	ENSG00000127377.10	CRYGN	155051	1.096132627	0.000269461	0.000801609
4893	ENSG00000244564.2	PRICKLE2-DT	101929316	1.440116484	0.000269807	0.00080252
4894	ENSG00000254992.1			2.210842779	0.000271114	0.000805742
4895	ENSG00000201736.1	RNA5SP160		1.569320696	0.000272166	0.000808626
4896	ENSG00000255082.1	GRM5-AS1	100873989	1.909035476	0.000272301	0.000808965
4897	ENSG00000233536.2			1.780537038	0.000273442	0.000812112
4898	ENSG00000205396.11	LINC00661	126536	2.455093437	0.000274702	0.000815427
4899	ENSG00000251045.1	SLC25A48-AS1	340074	1.625813572	0.000275111	0.000816397
4900	ENSG00000213033.4	AURKAP1		1.463387694	0.00027663	0.000820596
4901	ENSG00000224221.1	RPS6P8		1.727504099	0.00027692	0.000821335
4902	ENSG00000213118.4	CHCHD2P4		1.408815114	0.000277082	0.000821754
4903	ENSG00000279954.1			1.215418147	0.000278211	0.000824979
4904	ENSG00000248409.2	CCDC188BP		1.816665508	0.000278626	0.000825963
4905	ENSG00000259261.2	IGHV4OR15-8		1.934863274	0.000279733	0.000829119
4906	ENSG00000283740.1	TAF11L11	112488746	2.252631781	0.000280817	0.000832209
4907	ENSG00000260382.1	TMEM183AP3		1.382096503	0.000282451	0.000836801
4908	ENSG00000263727.1			1.406776256	0.000282705	0.00083749
4909	ENSG00000242675.1	RPS16P9		1.895291283	0.000283157	0.000838705
4910	ENSG00000267909.3	CCDC177	56936	1.245379452	0.000283482	0.000839605
4911	ENSG00000165805.10	C12orf50	160419	1.552104082	0.000283878	0.000840588
4912	ENSG00000267490.1			2.404656607	0.000287994	0.0008519
4913	ENSG00000225218.1			1.062174661	0.000288377	0.000852956
4914	ENSG00000286853.1			2.278580296	0.000288403	0.000852967
4915	ENSG00000262959.2	RPL23AP86		1.355030862	0.000288807	0.000854035
4916	ENSG00000251230.6	MIR3945HG	731424	1.097906477	0.000290603	0.000859155
4917	ENSG00000245928.2	SDAD1-AS1	101928809	1.62890694	0.000293526	0.000867214
4918	ENSG00000232537.2			1.761121361	0.000293669	0.000867509
4919	ENSG00000177418.2			1.381124307	0.000295234	0.000871935
4920	ENSG00000251453.1	HAUS1P1		1.415186059	0.000295666	0.000873145
4921	ENSG00000232946.1	RPL35AP20		1.645249906	0.000297317	0.000877761
4922	ENSG00000112038.18	OPRM1	4988	1.508403202	0.000301848	0.000890276
4923	ENSG00000234233.1	KCNH1-IT1		1.819579498	0.000305318	0.000899639
4924	ENSG00000132855.5	ANGPTL3	27329	1.169424923	0.000305693	0.00090061
4925	ENSG00000269226.7	TMSB15C	286527	1.206313572	0.000305979	0.000901321
4926	ENSG00000267504.1			1.46547258	0.00030601	0.000901343
4927	ENSG00000152086.9	TUBA3E	112714	1.717375989	0.00030693	0.000903987
4928	ENSG00000238280.2			1.18624589	0.000308154	0.000907389
4929	ENSG00000185899.1	TAS2R60	338398	1.294727326	0.000308628	0.000908651
4930	ENSG00000234795.10	RFTN1P1		2.181305769	0.000310996	0.000915011
4931	ENSG00000111291.8	GPRC5D	55507	1.039617048	0.000311892	0.000917511
4932	ENSG00000201264.1	SNORD73B	114655	1.373744109	0.000315295	0.000926902
4933	ENSG00000273950.1	Metazoa_SRP		1.229793686	0.00031783	0.000933524
4934	ENSG00000237852.1			1.052886733	0.000318561	0.000935532
4935	ENSG00000237208.1		101928495	2.166052877	0.000319025	0.000936755
4936	ENSG00000225334.3	LINC02813		1.543012567	0.000319414	0.000937689
4937	ENSG00000260470.1			1.472799239	0.0003215	0.000943256
4938	ENSG00000162068.2	NTN3	4917	1.22481752	0.000322186	0.000945198
4939	ENSG00000288585.1			1.195381955	0.00032745	0.000959506
4940	ENSG00000287538.1			1.891661605	0.000330259	0.000966994
4941	ENSG00000277373.1			1.484351344	0.000331761	0.000970989
4942	ENSG00000177725.5			1.51593507	0.000332433	0.000972814
4943	ENSG00000284623.1	LINC02786		1.570737015	0.000334441	0.000977905
4944	ENSG00000101282.9	RSPO4	343637	1.373656417	0.000338079	0.000987803
4945	ENSG00000206914.1	Y_RNA		1.330972586	0.000339205	0.00099102
4946	ENSG00000166159.11	LRTM2	654429	1.613089171	0.000340401	0.000994222
4947	ENSG00000248956.1	HMGB1P44		1.808540706	0.000343474	0.001002605
4948	ENSG00000287238.1			1.580739663	0.000343516	0.00100262
4949	ENSG00000203812.2			1.21553518	0.000343654	0.001002836
4950	ENSG00000103832.10	GOLGA8UP		1.287848515	0.000344067	0.001003851
4951	ENSG00000276127.1			2.106035109	0.000344492	0.001004763
4952	ENSG00000270379.6	HEATR9	256957	1.062290297	0.000345289	0.001006718
4953	ENSG00000211949.3	IGHV3-23		1.246571561	0.000345417	0.00100687
4954	ENSG00000287656.1			1.050011611	0.000347104	0.001011118
4955	ENSG00000251066.1			1.147969419	0.000349002	0.001016125
4956	ENSG00000200389.1	RNU6-509P		1.528065207	0.00034981	0.001018253
4957	ENSG00000211655.3	IGLV1-36		1.555746749	0.000350404	0.001019681
4958	ENSG00000181499.2	OR6T1	219874	1.801831589	0.000350787	0.001020572
4959	ENSG00000287872.1			1.350605548	0.000351681	0.001022947
4960	ENSG00000234511.10	C5orf58	133874	1.049157835	0.000353894	0.001029009
4961	ENSG00000288235.1	FAM106C	100129396	1.807339621	0.000354826	0.001031416
4962	ENSG00000198610.11	AKR1C4	1109	1.609221479	0.00035566	0.001033459
4963	ENSG00000173404.5	INSM1	3642	1.362804982	0.000356467	0.001035655
4964	ENSG00000283683.1	MYOCOS	110806290	1.35323065	0.000357452	0.001038362
4965	ENSG00000272386.1			1.47354153	0.000357494	0.001038396
4966	ENSG00000133519.12	ZDHHC8BP		1.040640313	0.000359507	0.001043797
4967	ENSG00000226801.2	OSTCP8		1.387579275	0.000361777	0.001050004
4968	ENSG00000274569.1			1.130692236	0.00036199	0.001050544
4969	ENSG00000251363.3	LINC02315		1.851636631	0.000362909	0.001053058
4970	ENSG00000284999.1			1.18737528	0.000363294	0.001053943
4971	ENSG00000183324.11	REC114	283677	1.252416615	0.000363325	0.001053957
4972	ENSG00000198723.11	SAXO5	374877	1.055566394	0.000365094	0.001058779
4973	ENSG00000287709.1		101928505	1.701876783	0.000365298	0.001059292
4974	ENSG00000268743.1			1.078577173	0.000365335	0.00105932
4975	ENSG00000286717.1			1.575854666	0.000367798	0.001065918
4976	ENSG00000279812.1			1.410439839	0.00036866	0.001068024
4977	ENSG00000249986.1	YWHAQP6		1.237387895	0.0003688	0.001068318
4978	ENSG00000060566.14	CREB3L3	84699	1.115462339	0.000369046	0.001068833
4979	ENSG00000269289.6			1.486555815	0.000373282	0.001079918
4980	ENSG00000204140.10	CLPSL1	340204	2.376569994	0.000374435	0.001082779
4981	ENSG00000213438.2	YBX2P1		1.377727541	0.000374506	0.001082826
4982	ENSG00000288528.1			1.343606248	0.000376243	0.001087529
4983	ENSG00000260213.6	CENPN-AS1		1.111047723	0.000376308	0.001087638
4984	ENSG00000235066.7		102723413	1.277942137	0.000377172	0.001089978
4985	ENSG00000198326.10	TMEM239	100288797	1.655982944	0.000378708	0.001094258
4986	ENSG00000266604.1	LINC01887		1.671894377	0.00037922	0.001095576
4987	ENSG00000242019.1	KIR3DL3	115653	1.989747364	0.000379827	0.001097022
4988	ENSG00000120329.7	SLC25A2	83884	1.343413415	0.000380256	0.001098089
4989	ENSG00000214107.8	MAGEB1	4112	4.960731491	0.000383569	0.00110677
4990	ENSG00000268047.1			1.002203158	0.000383852	0.001107425
4991	ENSG00000204644.10	ZFP57	346171	1.243142573	0.000384154	0.001108215
4992	ENSG00000269404.7	SPIB	6689	1.248648859	0.000384207	0.001108287
4993	ENSG00000238025.1	ZDHHC4P1		1.485211072	0.000384843	0.001110041
4994	ENSG00000188738.15	FSIP2	401024	1.004413531	0.000385998	0.001112806
4995	ENSG00000286423.1			1.187092741	0.000386358	0.001113683
4996	ENSG00000231749.3	ABCA9-AS1	104355297	1.65570329	0.00038654	0.001114045
4997	ENSG00000204933.3	CD177P1		1.500326504	0.000387023	0.001115356
4998	ENSG00000265871.1	MIR3174	100422841	1.310672614	0.000388911	0.001120554
4999	ENSG00000279394.1			1.659441178	0.000390432	0.00112469
5000	ENSG00000236117.1	LINC01639		2.940832481	0.000390565	0.00112499
5001	ENSG00000280402.1			1.166773326	0.000390801	0.00112559
5002	ENSG00000196188.12	CTSE	1510	1.272255762	0.000391748	0.001128155
5003	ENSG00000213731.2	RAB5CP1		1.516619887	0.000392316	0.001129461
5004	ENSG00000256943.1	GALNT9-AS1		2.042308531	0.000393063	0.001131528
5005	ENSG00000286358.1			1.754971823	0.000393592	0.001132722
5006	ENSG00000235244.4	DANT2	642776	1.219223944	0.000394772	0.001136037
5007	ENSG00000251821.1	RNU6-583P		1.39139971	0.000395528	0.001138046
5008	ENSG00000252361.1	RNU6-118P		1.010058759	0.000397804	0.001144014
5009	ENSG00000186152.6	LILRP1		1.34951509	0.000399525	0.001148631
5010	ENSG00000203908.4	KHDC3L	154288	2.06903396	0.000399682	0.001148999
5011	ENSG00000260585.1			1.728066913	0.000404486	0.001162218
5012	ENSG00000260792.1	LINC02280	101929634	1.269085891	0.000406535	0.001167599
5013	ENSG00000216835.3	RBMXP1		1.583460564	0.000407605	0.001170587
5014	ENSG00000249084.1			1.465566569	0.000413656	0.001186762
5015	ENSG00000259982.1	CDC37P1		1.499208656	0.000413822	0.001187152
5016	ENSG00000250986.1	LINC02600		1.189765271	0.000414345	0.001188479
5017	ENSG00000243466.1	IGKV1-5		1.254746688	0.000416628	0.001194596
5018	ENSG00000186115.13	CYP4F2	8529	1.424315916	0.000417529	0.00119666
5019	ENSG00000253507.5	IADEN		1.825340393	0.000419366	0.001201406
5020	ENSG00000186226.9	LCE1E	353135	1.472135996	0.000419532	0.001201793
5021	ENSG00000237166.2	LINC01792		1.159377995	0.000421998	0.001208421
5022	ENSG00000251054.1			1.481085616	0.000422716	0.001210389
5023	ENSG00000136750.13	GAD2	2572	2.124866334	0.000423354	0.00121213
5024	ENSG00000180043.11	GARIN5B	284418	1.996856179	0.000424157	0.001214339
5025	ENSG00000284713.1	SMIM38	107984345	1.047004091	0.000424252	0.001214526
5026	ENSG00000286115.1			1.361066924	0.000424755	0.001215788
5027	ENSG00000220418.1	TUBB3P1		1.71908561	0.000424961	0.00121629
5028	ENSG00000285808.1			1.933398829	0.000425112	0.001216548
5029	ENSG00000244116.3	IGKV2-28		1.577288033	0.00042671	0.001220857
5030	ENSG00000279378.1			1.369353722	0.000428504	0.001225573
5031	ENSG00000262663.1			1.024148401	0.000428605	0.001225747
5032	ENSG00000236760.1			2.087545327	0.000430018	0.001229523
5033	ENSG00000229052.2	MND1P1		1.033660657	0.00043019	0.001229749
5034	ENSG00000235246.1	TMEM184B-AS1	128385376	1.35620488	0.000431806	0.001233833
5035	ENSG00000224137.1	LINC01857		1.127818452	0.0004336	0.001238692
5036	ENSG00000111704.11	NANOG	79923	1.406203364	0.000435252	0.001242694
5037	ENSG00000231345.3	BEND3P1		1.014628954	0.000437082	0.001247561
5038	ENSG00000229703.6	LYPD9P		2.165851022	0.000438892	0.001252456
5039	ENSG00000287154.1			1.561535256	0.000439023	0.001252738
5040	ENSG00000229433.2	LINC02069	101928992	1.451892396	0.000442847	0.001262653
5041	ENSG00000260978.1			1.229021791	0.000443029	0.001262989
5042	ENSG00000286056.1			2.464876029	0.000444011	0.001265424
5043	ENSG00000231937.1			2.480044993	0.000444873	0.001267609
5044	ENSG00000213880.3	RPL7AP7		1.580625243	0.000445963	0.00127053
5045	ENSG00000285857.1			1.080856965	0.000447297	0.001274058
5046	ENSG00000258454.1			1.045372333	0.000447931	0.001275589
5047	ENSG00000266104.1	MIR4326	100422945	1.321735436	0.000452476	0.00128742
5048	ENSG00000120054.12	CPN1	1369	1.729560374	0.000453945	0.001291047
5049	ENSG00000258628.1	RHPN2P1		1.306672679	0.000454098	0.001291294
5050	ENSG00000281131.1	SCHLAP1	101669767	2.446957304	0.000454707	0.001292655
5051	ENSG00000146722.11	FKBP6P2		1.477467029	0.000455713	0.001295053
5052	ENSG00000227145.2	IL21-AS1	100996941	1.250242792	0.00045694	0.001298354
5053	ENSG00000229989.4	MIR181A1HG		1.30481596	0.000458422	0.001302284
5054	ENSG00000287046.1			1.503358842	0.000459239	0.001304419
5055	ENSG00000238178.7	MAGEB17-AS1		1.389008388	0.000459517	0.001305115
5056	ENSG00000211598.2	IGKV4-1		1.265590758	0.000459616	0.001305301
5057	ENSG00000225735.1	NEK4P1		2.092769226	0.000461702	0.001310663
5058	ENSG00000234956.7	LINC02539	100507406	1.041491132	0.000463701	0.001315772
5059	ENSG00000265293.2	ARGFXP2		1.247165965	0.000467837	0.001326748
5060	ENSG00000176165.12	FOXG1	2290	2.010354962	0.000469819	0.001331797
5061	ENSG00000258028.3			2.736578666	0.000469997	0.001332206
5062	ENSG00000250997.1			2.018814225	0.000470503	0.001333545
5063	ENSG00000231726.2	HMGN2P38		1.370206281	0.000470932	0.001334381
5064	ENSG00000285750.1			1.448357895	0.000471207	0.001335064
5065	ENSG00000165323.15	FAT3	120114	1.148440363	0.000471883	0.001336777
5066	ENSG00000260357.1	DNM1P34		1.964131469	0.000471913	0.001336777
5067	ENSG00000280228.1	AMANZI		1.270248543	0.000472631	0.001338715
5068	ENSG00000130287.14	NCAN	1463	1.207930563	0.000472666	0.001338719
5069	ENSG00000130167.14	TSPAN16	26526	1.36289405	0.000472826	0.001339077
5070	ENSG00000132704.16	FCRL2	79368	1.272441185	0.00047338	0.00134055
5071	ENSG00000282815.1	TEX13C	100129520	3.517231192	0.00047375	0.001341503
5072	ENSG00000204528.3	PSORS1C3		1.222154959	0.000477123	0.001350475
5073	ENSG00000244134.1	RPS12P20		1.408086739	0.000479286	0.001356016
5074	ENSG00000197421.10	GGT3P		1.494118859	0.000480568	0.001359546
5075	ENSG00000213483.4	NDUFAF4P2		1.591946986	0.000480888	0.001360256
5076	ENSG00000211638.2	IGLV8-61		1.518646586	0.000485487	0.00137219
5077	ENSG00000279200.1			1.45443431	0.000486323	0.001374357
5078	ENSG00000221044.2	U3		1.110717062	0.000488156	0.001379439
5079	ENSG00000172155.10	LCE1D	353134	2.393781555	0.000489599	0.001383319
5080	ENSG00000227962.1			1.198335336	0.000489767	0.001383696
5081	ENSG00000257265.1			2.05718725	0.000492338	0.001390761
5082	ENSG00000272057.1	ANKH-DT		1.511303768	0.000492705	0.001391603
5083	ENSG00000172020.13	GAP43	2596	1.081947162	0.000493329	0.001393036
5084	ENSG00000225420.1	EIF2AK3-AS1	101928371	1.312719119	0.000494006	0.001394578
5085	ENSG00000236510.1			1.361862217	0.000495488	0.001398264
5086	ENSG00000232422.1	KNOP1P4		1.411080909	0.000497409	0.001402987
5087	ENSG00000201916.1	Y_RNA		1.504563403	0.000500296	0.001410396
5088	ENSG00000162747.12	FCGR3B	2215	1.045046936	0.000500321	0.001410396
5089	ENSG00000281990.1	IGHV1-69-2		1.909134014	0.000501978	0.001414866
5090	ENSG00000146276.12	GABRR1	2569	1.06816025	0.000502225	0.001415462
5091	ENSG00000223795.2			1.845047493	0.000506624	0.00142715
5092	ENSG00000275665.1			1.371648823	0.000508553	0.001432379
5093	ENSG00000214855.9	APOC1P1		1.164385522	0.000508816	0.00143302
5094	ENSG00000260648.3			1.244299376	0.00051324	0.001444349
5095	ENSG00000223829.6			1.703888322	0.000513835	0.001445819
5096	ENSG00000248530.1	BCL2L12P1		1.421507368	0.000517962	0.001456914
5097	ENSG00000286509.1			1.393285984	0.000519755	0.001461543
5098	ENSG00000258331.1	LINC02461	105369740	1.542204228	0.000523219	0.001470451
5099	ENSG00000233144.1			1.190459063	0.000527089	0.001480591
5100	ENSG00000182256.13	GABRG3	2567	1.946797639	0.000528652	0.001484561
5101	ENSG00000283907.1			1.093010793	0.000529172	0.00148581
5102	ENSG00000239791.2			1.107928776	0.000529273	0.00148599
5103	ENSG00000111732.11	AICDA	57379	1.271418797	0.00052935	0.0014861
5104	ENSG00000286498.1		124901457	2.280446367	0.000529823	0.001487112
5105	ENSG00000226631.1	SLC9A3P3		1.846004463	0.000531095	0.001490261
5106	ENSG00000287089.1			1.483937239	0.000533202	0.001495625
5107	ENSG00000206931.1	RNU6-1042P		1.663725109	0.00053777	0.001507711
5108	ENSG00000249655.1			1.246307142	0.000537859	0.001507853
5109	ENSG00000266736.1	GTF2IP6		3.323445189	0.0005379	0.001507861
5110	ENSG00000220666.2	RCC2P7		1.456412577	0.000539749	0.001512617
5111	ENSG00000273111.6	LYPD4	147719	2.056424107	0.000543895	0.001523698
5112	ENSG00000269300.1			1.276365848	0.000545343	0.00152754
5113	ENSG00000270292.1			1.154838552	0.000545501	0.001527873
5114	ENSG00000281491.1	DNAJB5-DT	101926900	1.104032579	0.000546419	0.001530109
5115	ENSG00000229057.1	RPS3AP54		1.721684807	0.000546437	0.001530109
5116	ENSG00000270722.1	RNVU1-31	124904619	1.639984081	0.000547554	0.001532864
5117	ENSG00000267453.7	CLEC4OP		1.226543651	0.000553056	0.001547393
5118	ENSG00000263257.3	LINC03107		1.248904064	0.000554992	0.001552263
5119	ENSG00000241369.6	LINC01192	647107	1.903471996	0.000556481	0.001555989
5120	ENSG00000258811.1			1.053089043	0.000557045	0.001557235
5121	ENSG00000225513.1	RPL29P20		1.198156604	0.000559832	0.001564036
5122	ENSG00000249835.2	VCAN-AS1	105379054	1.575125065	0.000560258	0.001564893
5123	ENSG00000277999.1			1.017651551	0.000560403	0.001565189
5124	ENSG00000252206.1	RNU7-40P		1.121097929	0.000560866	0.00156637
5125	ENSG00000229191.1			1.211747857	0.000561683	0.001568544
5126	ENSG00000273102.1			1.427673693	0.000563674	0.001573881
5127	ENSG00000262075.3	DKFZP434A062	26102	1.427622826	0.000565402	0.001578485
5128	ENSG00000230086.1	VN1R96P		2.795006474	0.000565522	0.001578707
5129	ENSG00000231744.1			1.508181439	0.000565978	0.001579758
5130	ENSG00000204055.5	IER5L-AS1		2.020949101	0.000567311	0.001583034
5131	ENSG00000220161.4	LINC02076	400579	1.020359224	0.00056852	0.001586294
5132	ENSG00000288029.1			1.052079166	0.000570518	0.001591535
5133	ENSG00000279878.1			1.236733274	0.000572807	0.001597582
5134	ENSG00000227788.1	MTCO3P43		1.315952409	0.000573386	0.001599049
5135	ENSG00000125788.6	DEFB126	81623	1.648871154	0.000573414	0.001599049
5136	ENSG00000265102.1	MIR3942	100500904	1.138784308	0.000574448	0.001601597
5137	ENSG00000267474.1			1.152632127	0.000574542	0.001601746
5138	ENSG00000250869.2			1.30627385	0.000576442	0.001606589
5139	ENSG00000229256.1	ST13P13		1.055640033	0.00057871	0.001612344
5140	ENSG00000288234.1			1.372615126	0.000579097	0.001613309
5141	ENSG00000238562.1	RNU7-159P		3.427874356	0.000580198	0.00161591
5142	ENSG00000203565.3			1.60765633	0.000580234	0.00161591
5143	ENSG00000257507.1	LINC02373		1.449766196	0.000582586	0.001621889
5144	ENSG00000236779.1			1.22390271	0.000583467	0.001624146
5145	ENSG00000261786.1			1.038891256	0.000583686	0.001624496
5146	ENSG00000285771.1			1.063436002	0.00058423	0.001625897
5147	ENSG00000247311.3			1.079240493	0.000586903	0.001632188
5148	ENSG00000186297.12	GABRA5	2558	2.209647138	0.000586965	0.001632246
5149	ENSG00000259954.1	IL21R-AS1	283888	1.110244873	0.000590987	0.001642626
5150	ENSG00000260417.1			1.688721784	0.000591359	0.001643545
5151	ENSG00000283052.1	LINC02456		2.015880061	0.000591535	0.001643917
5152	ENSG00000235124.1	KRT8P17		1.839957144	0.000594237	0.001650849
5153	ENSG00000104722.14	NEFM	4741	1.510691848	0.000594429	0.001651151
5154	ENSG00000232111.2			2.354685174	0.000595592	0.001654034
5155	ENSG00000285966.1			1.110595413	0.000598381	0.001660963
5156	ENSG00000282173.1	TRBJ1-5		1.17393145	0.000600128	0.001665463
5157	ENSG00000207003.1	RNU6-611P		1.087098324	0.000602289	0.001670992
5158	ENSG00000168135.4	KCNJ4	3761	1.536098112	0.000602592	0.001671715
5159	ENSG00000264924.1			1.037592126	0.000604986	0.001677655
5160	ENSG00000230951.1	GPS2P2		1.696325786	0.000608393	0.00168663
5161	ENSG00000262902.1	MTCO1P40		1.111304135	0.000610131	0.001691094
5162	ENSG00000249965.2	CDC42P4		1.150765112	0.000614958	0.001703282
5163	ENSG00000225976.5	CKS1BP2		1.392566714	0.000616073	0.001706104
5164	ENSG00000244060.2	RPS2P41		1.463561357	0.000616468	0.001706866
5165	ENSG00000288565.1			2.02621168	0.000616956	0.00170798
5166	ENSG00000225572.1	DOCK4-AS1	100506413	1.477519221	0.000618949	0.001713376
5167	ENSG00000244104.3	RN7SL659P		1.950168359	0.000619602	0.001715066
5168	ENSG00000286772.1			2.735085125	0.000619934	0.001715866
5169	ENSG00000287494.1			1.0759204	0.000620033	0.00171602
5170	ENSG00000250710.3	OR7E99P		1.923635	0.000620636	0.001717569
5171	ENSG00000257545.5		100287944	1.110843614	0.000622313	0.00172185
5172	ENSG00000232621.1	C4BPAP2		1.736389309	0.000624143	0.001726551
5173	ENSG00000285578.1	LIRIL2R		1.49369343	0.000626202	0.00173128
5174	ENSG00000287794.1			1.541555445	0.000626422	0.001731767
5175	ENSG00000158553.4	POM121L2	94026	1.883135848	0.000629614	0.001740108
5176	ENSG00000248279.5	LINC02120	340113	1.630733496	0.000630614	0.001742506
5177	ENSG00000274816.1	MIR6772	102465463	1.140395381	0.000631698	0.00174538
5178	ENSG00000261124.1			1.072761732	0.000633082	0.001748959
5179	ENSG00000261812.6	TUBB8P7		1.064891256	0.000638811	0.001763804
5180	ENSG00000285367.1			1.485732615	0.000641741	0.001770906
5181	ENSG00000222915.1	RNU6-564P		1.369990285	0.000641964	0.001771313
5182	ENSG00000212135.1	SNORD67	692108	1.07770034	0.000641977	0.001771313
5183	ENSG00000221826.10	PSG3	5671	1.911750462	0.000643042	0.001773633
5184	ENSG00000278677.2	H2AC17	8336	1.379183534	0.000643516	0.001774694
5185	ENSG00000174417.3	TRHR	7201	1.2553922	0.000644432	0.001777095
5186	ENSG00000285644.1			1.205031932	0.000645089	0.001778683
5187	ENSG00000251169.2	LINC01843		1.369000605	0.000646386	0.001781989
5188	ENSG00000286370.1			1.649400456	0.000646543	0.001782174
5189	ENSG00000247345.2	PDE4D-AS1	139281640	1.84244994	0.000648043	0.001785811
5190	ENSG00000177910.8	SPATA31C2	645961	2.040981288	0.000653843	0.001800918
5191	ENSG00000253983.3			1.162475316	0.00065562	0.001805563
5192	ENSG00000166862.7	CACNG2	10369	1.618308957	0.000657628	0.001810588
5193	ENSG00000262267.6			2.453557915	0.000658549	0.001812873
5194	ENSG00000237713.1			2.313245693	0.000660422	0.001817399
5195	ENSG00000068985.5	PAGE1	8712	2.85717462	0.000662152	0.001821653
5196	ENSG00000105398.4	SULT2A1	6822	2.130850126	0.000663405	0.001824721
5197	ENSG00000232163.2	RPLP1P13		1.333741108	0.000668555	0.00183705
5198	ENSG00000243944.5			1.38063675	0.000669759	0.001840027
5199	ENSG00000244273.1	PGBD4P1		1.36205218	0.000670113	0.001840746
5200	ENSG00000105392.16	CRX	1406	1.497846731	0.000672322	0.001846172
5201	ENSG00000231646.6	FSIP2-AS1	101927196	1.222583936	0.000675068	0.001853071
5202	ENSG00000287171.1	LINC03113		1.481609919	0.000675766	0.001854528
5203	ENSG00000143921.9	ABCG8	64241	1.256820552	0.000675785	0.001854528
5204	ENSG00000280326.1			1.327163948	0.000678132	0.00186071
5205	ENSG00000244575.3	IGKV1-27		1.344730901	0.00068549	0.001878819
5206	ENSG00000276775.1	IGHV4-4		1.354671024	0.000687815	0.00188493
5207	ENSG00000253832.1			1.600703426	0.00068911	0.001888218
5208	ENSG00000231976.8	FAM238B		2.255936405	0.000689728	0.001889782
5209	ENSG00000279756.1			1.37835664	0.000690805	0.00189247
5210	ENSG00000254002.2			2.338523186	0.000692927	0.001898154
5211	ENSG00000285666.1			1.678156725	0.000693487	0.001899423
5212	ENSG00000261052.6	SULT1A3	6818	1.2257716	0.000695144	0.001903625
5213	ENSG00000211679.2	IGLC3		1.156433046	0.000696495	0.001907135
5214	ENSG00000232874.1	LINC02924		1.079497506	0.000697221	0.001908729
5215	ENSG00000264207.1			1.105152432	0.00070013	0.001916032
5216	ENSG00000251087.3	ALG1L3P		1.487157003	0.000705631	0.001930019
5217	ENSG00000233623.2	PGAM1P11		1.264700655	0.000708684	0.001937168
5218	ENSG00000278267.1	MIR6859-1	102466751	1.500128395	0.000711293	0.001943366
5219	ENSG00000121211.8	MND1	84057	1.041394854	0.000712372	0.001945638
5220	ENSG00000236546.1	MYCL-AS1	105378668	1.0461758	0.000712634	0.00194622
5221	ENSG00000274008.1	Metazoa_SRP		1.306210762	0.000712865	0.001946718
5222	ENSG00000266446.1			1.265905108	0.000714698	0.001951454
5223	ENSG00000237850.7	GOLGA6L24	645202	1.933933211	0.000716245	0.001955006
5224	ENSG00000242995.1	RPL36AP1		2.029478349	0.000716793	0.001956231
5225	ENSG00000278857.1	IGKV1D-12		1.497341547	0.000725442	0.001978473
5226	ENSG00000010379.16	SLC6A13	6540	1.056777673	0.000729299	0.001988037
5227	ENSG00000217767.2	NDUFAB1P1		1.303527471	0.000733869	0.001999255
5228	ENSG00000205426.10	KRT81	3887	1.036533132	0.000734184	0.001999978
5229	ENSG00000232928.2	DDX3P1		1.322274847	0.000735492	0.002002988
5230	ENSG00000233980.1	FDPSP2		1.271807246	0.000736061	0.002004263
5231	ENSG00000255186.1			1.424989893	0.000736297	0.002004768
5232	ENSG00000275989.2	SPACA7BP		1.791264511	0.00073668	0.002005536
5233	ENSG00000251687.1			1.434897091	0.000741941	0.002018057
5234	ENSG00000216588.9	IGSF23	147710	1.196278547	0.000744364	0.002023953
5235	ENSG00000250050.1	MTND4P9		1.334719659	0.000744957	0.002025428
5236	ENSG00000182798.10	MAGEB17	645864	1.464009945	0.00074612	0.002028032
5237	ENSG00000211900.2	IGHJ6		1.59104623	0.000746749	0.002029603
5238	ENSG00000278090.3	LUNAR1		1.021805623	0.0007475	0.002031505
5239	ENSG00000273274.2	ZBTB8B	728116	1.335931599	0.000749169	0.002035501
5240	ENSG00000104818.15	CGB2	114336	2.38559788	0.000752736	0.002044895
5241	ENSG00000263443.1			1.46753565	0.00075378	0.002047449
5242	ENSG00000267424.1	RTBDN-AS1	105372281	1.704770771	0.000756524	0.002054199
5243	ENSG00000160505.16	NLRP4	147945	1.61747881	0.000756758	0.002054695
5244	ENSG00000225406.1	ELF2P4		1.518515371	0.000757941	0.002057764
5245	ENSG00000253170.1			2.991280673	0.000758573	0.002059198
5246	ENSG00000287887.1			1.071548316	0.0007587	0.002059402
5247	ENSG00000279511.1			1.010591234	0.00076195	0.002067517
5248	ENSG00000259163.2	TTC7B-AS1	101928909	1.263047879	0.000763394	0.002071153
5249	ENSG00000197919.6	IFNA1	3439	1.384293276	0.000763908	0.002072263
5250	ENSG00000243264.2	IGKV2D-29		1.441182797	0.000765014	0.002075121
5251	ENSG00000226510.1	UPK1A-AS1		1.941219288	0.000765196	0.00207533
5252	ENSG00000272980.4			1.147422635	0.000766895	0.002079655
5253	ENSG00000242325.1	RPS12P31		1.042809114	0.000767376	0.002080815
5254	ENSG00000288052.1			1.248039625	0.000770429	0.002088238
5255	ENSG00000276131.1			1.745662042	0.000773959	0.002097376
5256	ENSG00000287169.1			1.520950979	0.000775034	0.002100001
5257	ENSG00000266733.7	TBC1D29P		1.057809695	0.000775113	0.002100073
5258	ENSG00000261208.1			1.114639469	0.00077668	0.002103886
5259	ENSG00000235574.1			1.558936403	0.000780507	0.002113242
5260	ENSG00000102539.5	MLNR	2862	1.102053904	0.00078419	0.002121615
5261	ENSG00000204657.4	OR2H2	7932	1.375550506	0.000785524	0.002124619
5262	ENSG00000250992.2			1.962369635	0.000785752	0.002124977
5263	ENSG00000285768.1	LINC02849	105375622	1.15873157	0.000786842	0.002127343
5264	ENSG00000080007.8	DDX43	55510	1.082076525	0.0007873	0.002128436
5265	ENSG00000252355.1	RN7SKP287		1.159068028	0.000788171	0.002130646
5266	ENSG00000288013.1			2.124991793	0.000794678	0.00214648
5267	ENSG00000205927.5	OLIG2	10215	1.430269463	0.000795423	0.002148201
5268	ENSG00000286068.1			1.401317643	0.000796313	0.002150312
5269	ENSG00000228728.1	PPIAP91		1.065525976	0.000802315	0.002164895
5270	ENSG00000225972.1	MTND1P23		1.335517671	0.000802753	0.002165636
5271	ENSG00000144834.14	TAGLN3	29114	1.174596461	0.000803209	0.002166718
5272	ENSG00000176970.8	RPL7L1P11		1.614780883	0.000806092	0.002174201
5273	ENSG00000277387.1	MIR8058	102465863	1.396778662	0.00080815	0.002179154
5274	ENSG00000211952.3	IGHV4-28		1.29021559	0.000808204	0.002179154
5275	ENSG00000183837.9	PNMA3	29944	1.065146697	0.000809818	0.002182953
5276	ENSG00000178445.10	GLDC	2731	1.190130636	0.000809833	0.002182953
5277	ENSG00000284059.1	MIR5572	100847042	1.464442278	0.000810874	0.002185611
5278	ENSG00000254274.1	CRIPTOP5		1.252626431	0.000810944	0.00218565
5279	ENSG00000211976.2	IGHV3-73		1.38926677	0.000812464	0.00218945
5280	ENSG00000182111.9	ZNF716	441234	2.550629356	0.000814676	0.002194815
5281	ENSG00000231739.2	GAPDHP59		1.292727118	0.000815576	0.002196792
5282	ENSG00000211668.2	IGLV2-11		1.282266627	0.000820051	0.002207644
5283	ENSG00000181260.8	MTHFD2P7		1.256210644	0.000821947	0.002212299
5284	ENSG00000240373.1	SEC62-AS1		1.059238903	0.000823193	0.002215397
5285	ENSG00000261078.1			1.891114	0.00082321	0.002215397
5286	ENSG00000285413.1			1.686813482	0.000824528	0.00221858
5287	ENSG00000226699.1			1.370205197	0.00082456	0.00221858
5288	ENSG00000224209.8	LINC00466		1.361837608	0.000825435	0.002220782
5289	ENSG00000266978.1	LIMASI		1.869111059	0.000826073	0.002222348
5290	ENSG00000286954.1			1.801579064	0.000827985	0.002226585
5291	ENSG00000159398.16	CES5A	221223	1.406444127	0.000829883	0.002231237
5292	ENSG00000272180.1			1.302163432	0.000830065	0.002231428
5293	ENSG00000286678.1			1.670547122	0.000830067	0.002231428
5294	ENSG00000286809.1			1.14708262	0.000832545	0.002237481
5295	ENSG00000222179.1	RN7SKP26		1.302687902	0.000837571	0.002249313
5296	ENSG00000261815.1			2.076745276	0.000838626	0.002251994
5297	ENSG00000132911.5	NMUR2	56923	1.631164722	0.000841454	0.002259282
5298	ENSG00000285608.1			1.243644885	0.000846442	0.002272059
5299	ENSG00000250740.1			1.307039722	0.00084669	0.00227257
5300	ENSG00000288070.1			1.72439791	0.000847435	0.002274263
5301	ENSG00000240596.1	KCNAB1-AS2	100874083	1.888960879	0.000850666	0.002282469
5302	ENSG00000216331.2	H1-12P		1.21914494	0.000851083	0.002283434
5303	ENSG00000219553.2			1.241093601	0.000851152	0.002283464
5304	ENSG00000205267.5	DGAT2L7P		1.824360315	0.000851813	0.002285082
5305	ENSG00000128322.7	IGLL1	3543	1.457343667	0.000854226	0.002291245
5306	ENSG00000280242.1			1.198663036	0.000855015	0.002293052
5307	ENSG00000254275.6	LINC00824		1.429156315	0.000856164	0.002295513
5308	ENSG00000251039.2	IGKV2D-40		1.480466648	0.000856644	0.002296488
5309	ENSG00000247228.3			1.688914126	0.000859187	0.002302713
5310	ENSG00000286163.1			1.465489822	0.000859494	0.002303196
5311	ENSG00000282651.2	IGHV5-10-1		1.555103171	0.000861072	0.002306799
5312	ENSG00000231210.3	COMETT		1.327545526	0.000868362	0.002325229
5313	ENSG00000223734.2	LAPTM4A-DT		1.085150952	0.000871969	0.002333469
5314	ENSG00000227972.1	THAP12P3		1.534019304	0.000872169	0.002333849
5315	ENSG00000138892.11	TTLL8	164714	1.531612607	0.000875866	0.002343424
5316	ENSG00000223669.1	NTSR1-AS1		1.966204409	0.000877165	0.002346743
5317	ENSG00000228437.5	LINC02474		1.825554033	0.000877625	0.002347815
5318	ENSG00000236366.3		153910	1.917964669	0.000877863	0.002348293
5319	ENSG00000258731.1	DDHD1-DT	101927620	1.270583509	0.000879727	0.002352802
5320	ENSG00000232765.6			2.046751178	0.000884722	0.002365044
5321	ENSG00000261633.1			1.203084477	0.000886487	0.002369124
5322	ENSG00000187904.3			1.211926896	0.000891864	0.002382851
5323	ENSG00000271369.1			1.436516362	0.000892764	0.002385096
5324	ENSG00000211657.3	IGLV3-32		2.004907965	0.000894884	0.002390438
5325	ENSG00000231464.1	PPP4R2P3		1.118232045	0.00089621	0.002393494
5326	ENSG00000253218.1	KLF3P1		1.245631189	0.000900702	0.002404683
5327	ENSG00000234913.1			1.224631331	0.000901528	0.002406574
5328	ENSG00000224814.1			1.668685751	0.000904321	0.002413695
5329	ENSG00000233456.1	LINC01077		1.621238046	0.000905941	0.00241753
5330	ENSG00000119614.3	VSX2	338917	1.488606583	0.00090836	0.002423495
5331	ENSG00000259964.7	THSD4-AS1		1.587556443	0.000910772	0.002429604
5332	ENSG00000261848.5			1.112518794	0.000911815	0.002432059
5333	ENSG00000234354.3	RPS26P47		1.088112344	0.000914079	0.002437753
5334	ENSG00000229896.2			1.022043849	0.000917403	0.002445648
5335	ENSG00000253768.1			1.372463912	0.000917597	0.002445999
5336	ENSG00000284957.1			1.655843122	0.000922505	0.002458258
5337	ENSG00000253901.1			2.513328671	0.000925542	0.002466019
5338	ENSG00000238153.1			1.979141205	0.000926973	0.002469333
5339	ENSG00000251598.2			1.690060257	0.000927589	0.002470808
5340	ENSG00000213440.2	H2AZP1		1.296853692	0.000928924	0.0024737
5341	ENSG00000269859.1	ZNF628-DT		1.394208051	0.000938209	0.002497417
5342	ENSG00000244620.1			1.566086631	0.000938661	0.002498454
5343	ENSG00000211639.2	IGLV4-60		1.486208935	0.000940748	0.002503337
5344	ENSG00000283422.1	LINC02452		1.222834034	0.000941729	0.002505778
5345	ENSG00000255951.1	IFT57P1		1.987392746	0.000941877	0.002506005
5346	ENSG00000075043.19	KCNQ2	3785	1.363184256	0.000942242	0.00250679
5347	ENSG00000207606.1	MIR554	693139	1.355987917	0.000944762	0.002512837
5348	ENSG00000282943.1	PSG11-AS1		1.678224382	0.000947566	0.002519282
5349	ENSG00000233571.1			2.681846709	0.0009479	0.002519831
5350	ENSG00000278615.5	C11orf98	102288414	1.239841595	0.000950806	0.00252654
5351	ENSG00000275830.2	LINC03082	101927646	1.159016934	0.00095259	0.002531111
5352	ENSG00000154997.9	SEPTIN14	346288	1.8068192	0.000959186	0.002547613
5353	ENSG00000202347.1	RNU1-16P		1.348460892	0.00096167	0.002552702
5354	ENSG00000211951.2	IGHV2-26		1.406607525	0.000961746	0.002552702
5355	ENSG00000260877.2			1.442373512	0.000962523	0.002554423
5356	ENSG00000283283.2			1.377539421	0.000965195	0.002561
5357	ENSG00000257194.2			1.289448545	0.000965299	0.002561105
5358	ENSG00000270299.1			1.001823636	0.000968399	0.002568125
5359	ENSG00000170160.17	CCDC144A	9720	1.210774216	0.00096927	0.002570092
5360	ENSG00000287932.1			1.219763098	0.000973891	0.00258131
5361	ENSG00000260545.1			1.221962236	0.000973997	0.002581418
5362	ENSG00000249639.1		124901073	1.4252041	0.000974408	0.002582161
5363	ENSG00000273189.1			1.107303631	0.000975221	0.002583969
5364	ENSG00000211962.2	IGHV1-46		1.242979102	0.00098612	0.002610408
5365	ENSG00000140623.14	SEPTIN12	124404	1.679603906	0.000986481	0.002611189
5366	ENSG00000224717.1	LEMD1-DT	284577	1.763060847	0.00099155	0.002623289
5367	ENSG00000253369.2			1.407084925	0.000991582	0.002623289
5368	ENSG00000235615.2			2.033604007	0.000993942	0.002628831
5369	ENSG00000225595.2	LINC01623		1.747673645	0.000994515	0.002630172
5370	ENSG00000204529.4	GUCY2EP		1.959285645	0.000995877	0.002633246
5371	ENSG00000214546.4			1.370860137	0.000996325	0.002633988
5372	ENSG00000172752.14	COL6A5	256076	1.137729777	0.000996357	0.002633988
5373	ENSG00000259696.2			1.535584149	0.000998919	0.002640408
5374	ENSG00000203364.2			1.272718363	0.001001267	0.002646439
5375	ENSG00000227240.2			1.200829412	0.001005635	0.002656921
5376	ENSG00000136352.19	NKX2-1	7080	1.586510553	0.001010943	0.002669346
5377	ENSG00000254180.2			1.199392572	0.001012532	0.002672896
5378	ENSG00000170925.3	TEX13B	56156	1.411967231	0.001012558	0.002672896
5379	ENSG00000196071.5	OR2L13	284521	1.878996897	0.001013581	0.002675242
5380	ENSG00000075891.22	PAX2	5076	1.509348414	0.001026031	0.002705941
5381	ENSG00000234426.3			2.436424899	0.001028275	0.002710958
5382	ENSG00000239122.1	RNU2-11P		1.264778082	0.001029038	0.002712607
5383	ENSG00000213873.4	PPIAP86		1.31407321	0.001032223	0.002720164
5384	ENSG00000278599.5	TBC1D3E	102723859	2.13012478	0.001032493	0.002720631
5385	ENSG00000239148.1	U8		3.552288936	0.001034891	0.002726223
5386	ENSG00000233208.6	LINC00642	400238	1.461714509	0.001036064	0.002728951
5387	ENSG00000275898.1			1.49201325	0.001037905	0.002733256
5388	ENSG00000211667.3	IGLV3-12		1.381628227	0.001038286	0.002733895
5389	ENSG00000267178.3	PHF5AP1		1.199639069	0.001038916	0.002735262
5390	ENSG00000187855.5	ASCL4	121549	1.567729866	0.001039025	0.002735298
5391	ENSG00000186395.8	KRT10	3858	1.138807581	0.001043142	0.002745042
5392	ENSG00000207200.1	RNU6-45P		1.334879162	0.001048646	0.002758426
5393	ENSG00000285722.1		124905143	2.025360868	0.001050403	0.002762682
5394	ENSG00000213005.3	PTTG3P		1.185703606	0.001055182	0.002773962
5395	ENSG00000226349.1			1.444457095	0.001057333	0.002779065
5396	ENSG00000271131.1	PPIAP75		1.043014876	0.001059865	0.002785165
5397	ENSG00000283209.1			1.393224278	0.001061389	0.002788802
5398	ENSG00000267388.1			1.10319844	0.001063669	0.002794606
5399	ENSG00000239649.3	MYADML		1.927340378	0.001067493	0.00280391
5400	ENSG00000253739.1	RNU6-323P		1.280434859	0.001072284	0.002815002
5401	ENSG00000229390.1	MICD		1.704427434	0.0010745	0.002820447
5402	ENSG00000276454.1			1.247418081	0.001074912	0.002821341
5403	ENSG00000258730.1	ITPK1-AS1	319085	1.343348847	0.001075159	0.002821804
5404	ENSG00000183629.13	GOLGA8G	283768	1.840150678	0.001075831	0.002823288
5405	ENSG00000233845.1			1.36601596	0.00107648	0.002824334
5406	ENSG00000223528.7			1.137646841	0.001080634	0.002834672
5407	ENSG00000257884.2			1.321055709	0.001084061	0.002842346
5408	ENSG00000265753.2	RN7SL444P		1.262363628	0.001085429	0.002845743
5409	ENSG00000234844.1	CDC42P2		1.50640493	0.001087277	0.002850212
5410	ENSG00000102230.14	PCYT1B	9468	1.112679992	0.001090543	0.002858206
5411	ENSG00000231787.4	H2BP9		1.487343664	0.001097857	0.002875664
5412	ENSG00000230778.1	ANKRD63	100131244	2.040607667	0.00110219	0.002886252
5413	ENSG00000250933.1	GAPDHP66		1.362968413	0.001103069	0.002888361
5414	ENSG00000230699.2			1.371148852	0.001110026	0.002905427
5415	ENSG00000227009.1	FUNDC2P4		1.425151235	0.001111268	0.002908295
5416	ENSG00000262343.1			1.259414557	0.001111756	0.002909381
5417	ENSG00000257951.2			1.224801699	0.001114832	0.002916843
5418	ENSG00000168367.11	LINC00917	732275	2.201248048	0.001116036	0.002919425
5419	ENSG00000238269.9	PAGE2B	389860	1.912275202	0.001120088	0.002928671
5420	ENSG00000207321.1	RNU6-160P		1.741682693	0.00112178	0.002932514
5421	ENSG00000169059.12	VCX3A	51481	2.022848096	0.001127257	0.002945474
5422	ENSG00000228802.1			1.236058953	0.001128324	0.002948067
5423	ENSG00000250838.1			2.740224315	0.001131332	0.002955342
5424	ENSG00000167332.9	OR51E2	81285	1.034102407	0.001136948	0.002969035
5425	ENSG00000259654.1			1.01299807	0.001140422	0.002976734
5426	ENSG00000249779.1			1.160416584	0.001141528	0.002979424
5427	ENSG00000226604.2	PAPPA-AS2		1.443291169	0.001142201	0.002980803
5428	ENSG00000252585.1	Y_RNA		1.251057324	0.001142519	0.002981421
5429	ENSG00000285584.2			1.908912643	0.001157993	0.003018429
5430	ENSG00000278263.2			1.380692161	0.001159092	0.003021092
5431	ENSG00000279476.1			1.018159111	0.001159637	0.003022113
5432	ENSG00000237708.1	SPIN2P1		1.430837398	0.001160663	0.003024589
5433	ENSG00000279071.1			1.251217232	0.001161098	0.003025523
5434	ENSG00000255704.1			2.052072741	0.001162523	0.00302864
5435	ENSG00000211972.2	IGHV3-66		1.286235594	0.001164275	0.003032209
5436	ENSG00000206603.1	SNORA22B	109616964	1.362062786	0.001164896	0.003033426
5437	ENSG00000235649.2	MXRA5Y		1.187563361	0.001165998	0.003036098
5438	ENSG00000267440.2	LINC02594		1.000447428	0.00116836	0.003041249
5439	ENSG00000287248.1			1.148887211	0.001170641	0.003046788
5440	ENSG00000182050.13	MGAT4C	25834	1.185912006	0.001176511	0.003061462
5441	ENSG00000283045.1			1.283444102	0.001177102	0.003062597
5442	ENSG00000267462.1			1.342664554	0.001177995	0.003064719
5443	ENSG00000253973.3	NRIP3-DT	105376541	1.366606699	0.001180441	0.003070276
5444	ENSG00000222533.1	RNU6-705P		1.915012726	0.001188523	0.00308988
5445	ENSG00000270540.1			1.587269121	0.001189551	0.003092042
5446	ENSG00000287059.1			1.203990228	0.00119142	0.003096193
5447	ENSG00000213026.4	CFL1P4		1.035819086	0.001193684	0.003101061
5448	ENSG00000163914.5	RHO	6010	1.494756527	0.001194712	0.003103323
5449	ENSG00000264781.1	MIR4537	100616422	1.739626012	0.001196555	0.00310739
5450	ENSG00000279648.1			1.789163104	0.00120771	0.003133803
5451	ENSG00000198156.10	NPIPB6	728741	1.10883577	0.001209442	0.003137885
5452	ENSG00000152910.19	CNTNAP4	85445	2.018563192	0.001210467	0.003140133
5453	ENSG00000277352.1			2.630475904	0.00121132	0.003141935
5454	ENSG00000261837.1			1.303799574	0.00121264	0.003145155
5455	ENSG00000260073.2			3.237789533	0.001219809	0.003161885
5456	ENSG00000188425.4	NANOS2	339345	2.246353552	0.00122702	0.003178708
5457	ENSG00000197181.12	PIWIL2	55124	1.152596238	0.001232987	0.003193354
5458	ENSG00000287715.1			1.256838105	0.001232996	0.003193354
5459	ENSG00000249664.1			1.304293007	0.001236904	0.003201383
5460	ENSG00000267674.1			1.887855639	0.001237006	0.00320144
5461	ENSG00000279124.1	IGSF3P1		1.099349363	0.001237917	0.003203379
5462	ENSG00000222004.7	LINC02860	285941	1.387241903	0.001239198	0.003206484
5463	ENSG00000149435.12	GGTLC1	92086	1.66958508	0.001240431	0.003209256
5464	ENSG00000228997.1	ORMDL1P1		1.834682185	0.001244917	0.003220022
5465	ENSG00000266397.1	LPIN2-AS2		1.541329644	0.001247603	0.003226758
5466	ENSG00000211765.1	TRBJ2-2		1.336823543	0.001250945	0.003234348
5467	ENSG00000226191.3	CLK3P2		2.333186476	0.0012521	0.003236489
5468	ENSG00000276430.3	FAM25C	644054	1.446693289	0.001256158	0.00324591
5469	ENSG00000235848.4	RMDN2-AS1		1.169181864	0.001256317	0.00324591
5470	ENSG00000221845.4	LINC02902	401335	1.509409234	0.001267573	0.003273071
5471	ENSG00000231475.3	IGHV4-31		1.270146282	0.001268841	0.003275918
5472	ENSG00000275226.1	LINC00547	400121	1.60100279	0.001271335	0.003281502
5473	ENSG00000260412.1			1.453150795	0.001282914	0.00330859
5474	ENSG00000207171.1		124900181	1.329499321	0.001283065	0.003308764
5475	ENSG00000284695.1			1.972394126	0.001283449	0.003309539
5476	ENSG00000231896.1			1.743876012	0.001285516	0.003314438
5477	ENSG00000238188.1	SPRING1P2		1.175355308	0.001285937	0.003315308
5478	ENSG00000254709.8	IGLL5	100423062	1.134931141	0.001289435	0.00332411
5479	ENSG00000259425.5			1.676877071	0.001298363	0.003346039
5480	ENSG00000186732.14	MPPED1	758	1.4573569	0.001299401	0.003348495
5481	ENSG00000260695.1			1.977646742	0.001306786	0.003365995
5482	ENSG00000273584.1			1.380962141	0.001307818	0.003368214
5483	ENSG00000237090.1	MKRN8P		1.332582551	0.001310326	0.003373578
5484	ENSG00000264647.1			1.569731085	0.001312789	0.003379481
5485	ENSG00000271181.1	STMN3P1		1.207736888	0.001315744	0.003386647
5486	ENSG00000279065.1			1.185758961	0.001317964	0.003391041
5487	ENSG00000256732.1			1.950454752	0.001322676	0.003402284
5488	ENSG00000280053.1			1.119377241	0.001324683	0.003407004
5489	ENSG00000113211.5	PCDHB6	56130	1.089716257	0.001325583	0.003409097
5490	ENSG00000211710.3	TRBV4-1		1.070210349	0.001331265	0.00342149
5491	ENSG00000215838.4			1.101975788	0.001331961	0.003422834
5492	ENSG00000201723.2	Y_RNA		1.518581521	0.001332928	0.003424877
5493	ENSG00000095596.12	CYP26A1	1592	1.708223627	0.001334125	0.003427507
5494	ENSG00000256482.1	BCAT1-AS1		1.539470268	0.00134285	0.003447019
5495	ENSG00000264734.1			3.518621386	0.001343631	0.003448801
5496	ENSG00000113196.3	HAND1	9421	1.910778371	0.001351757	0.003468534
5497	ENSG00000274001.1			1.157874519	0.001354708	0.003475209
5498	ENSG00000282876.1			1.282790935	0.00135739	0.003481188
5499	ENSG00000234715.2	SLC26A5-AS1	101927870	2.020097683	0.001360216	0.003487759
5500	ENSG00000205537.2			1.159180252	0.00136036	0.003487902
5501	ENSG00000227706.4	ERVH-3		1.683617077	0.001364235	0.003496933
5502	ENSG00000236654.2			1.463456078	0.001364979	0.003498613
5503	ENSG00000232031.1			1.446500582	0.001371104	0.003513404
5504	ENSG00000256250.1			1.277824683	0.001376936	0.003526981
5505	ENSG00000266302.6			1.528838659	0.001379428	0.003532451
5506	ENSG00000168875.3	SOX14	8403	2.93275289	0.001380536	0.00353506
5507	ENSG00000279774.1			1.993813326	0.001380978	0.003535964
5508	ENSG00000253908.1			1.145996782	0.001383797	0.003542266
5509	ENSG00000249713.1		101929109	1.472339518	0.001384714	0.003544386
5510	ENSG00000171747.9	LGALS4	3960	1.004951358	0.001385993	0.003546973
5511	ENSG00000237641.1	RPL28P2		1.044278854	0.001388119	0.003551725
5512	ENSG00000235200.3	LINC01702	102724481	1.332215345	0.001402059	0.003581383
5513	ENSG00000249650.1	CEP72-DT		1.414153026	0.001410506	0.003600409
5514	ENSG00000251627.1			1.358869728	0.001413902	0.003607394
5515	ENSG00000237331.1	XIAP-AS1		1.353602662	0.00141397	0.003607394
5516	ENSG00000275381.1			1.305300015	0.001416407	0.003612682
5517	ENSG00000281958.1	TRBJ1-4		1.271666728	0.001418756	0.003618208
5518	ENSG00000250020.1	SPICP4		1.543699946	0.001423397	0.003629108
5519	ENSG00000133475.17	GGT2P		1.344381154	0.001429617	0.00364356
5520	ENSG00000253125.1	EGR3-AS1		1.103713873	0.001430453	0.003645457
5521	ENSG00000259283.2			1.440815126	0.001438043	0.003663553
5522	ENSG00000263413.2	MIR4538	100616276	1.664035974	0.001438108	0.003663553
5523	ENSG00000227912.2			1.479596802	0.001441878	0.003671505
5524	ENSG00000204684.4		284788	1.567760318	0.001442361	0.003672313
5525	ENSG00000211660.3	IGLV2-23		1.168734121	0.001443355	0.003674323
5526	ENSG00000243675.1	RPL35AP9		1.272043208	0.001444066	0.003675426
5527	ENSG00000230026.2			1.666287216	0.001445667	0.003679264
5528	ENSG00000165970.12	SLC6A5	9152	1.291362677	0.001448752	0.003686169
5529	ENSG00000282600.2	IGHV3-69-1		1.21976142	0.00145161	0.003693205
5530	ENSG00000273428.3			1.158132676	0.001455026	0.003700708
5531	ENSG00000279691.1			1.022097192	0.001460895	0.003714442
5532	ENSG00000267457.1			1.133468796	0.001462255	0.003717662
5533	ENSG00000267076.1	MIX23P3		1.156014144	0.00146579	0.003725694
5534	ENSG00000142182.9	DNMT3L	29947	2.11514402	0.001466892	0.003728255
5535	ENSG00000240403.5	KIR3DL2	3812	1.00394262	0.001469091	0.003732889
5536	ENSG00000264215.1			1.244324711	0.001469363	0.003733341
5537	ENSG00000207940.1	MIR567	693152	1.303633277	0.001471293	0.003737765
5538	ENSG00000168671.10	UGT3A2	167127	1.207684274	0.001472729	0.003741173
5539	ENSG00000233359.2			2.531153072	0.001473165	0.003742042
5540	ENSG00000254884.1	PRR13P2		1.205930963	0.001484435	0.003766807
5541	ENSG00000270433.1	H3P37		1.042850745	0.001485084	0.003767973
5542	ENSG00000231128.6			1.264137979	0.001494478	0.003790446
5543	ENSG00000214039.9	LINC02418		1.249471574	0.001494515	0.003790446
5544	ENSG00000131951.11	LRRC9	341883	1.017186626	0.00149984	0.003801513
5545	ENSG00000211967.3	IGHV3-53		1.237997185	0.001499934	0.003801513
5546	ENSG00000244342.5	LINC00698	285401	1.405479199	0.001501929	0.003806328
5547	ENSG00000206448.3	PPIAP30		1.025203154	0.0015073	0.00381823
5548	ENSG00000255519.1			1.970441147	0.001517234	0.003841431
5549	ENSG00000230829.3			1.313682844	0.001518184	0.00384359
5550	ENSG00000084453.16	SLCO1A2	6579	1.248893258	0.001529679	0.003871456
5551	ENSG00000260100.1			1.238762744	0.001539648	0.003894947
5552	ENSG00000257893.3	LINC02404	105369900	2.22469499	0.001543128	0.00390151
5553	ENSG00000094661.3	OR1I1	126370	1.355240921	0.001549846	0.003916996
5554	ENSG00000205414.1	NKD1-AS1		1.299346114	0.001550068	0.003917308
5555	ENSG00000284651.1			1.508220875	0.001550858	0.003919055
5556	ENSG00000234900.1			1.215238948	0.001553195	0.00392471
5557	ENSG00000121853.4	GHSR	2693	1.809392325	0.001553382	0.003924933
5558	ENSG00000242163.1			1.005823751	0.001556802	0.00393207
5559	ENSG00000138622.4	HCN4	10021	1.161431524	0.001568963	0.003961058
5560	ENSG00000238015.2			1.413644298	0.001568978	0.003961058
5561	ENSG00000261430.1			1.288289566	0.001577522	0.0039806
5562	ENSG00000239881.1	RPS27P25		1.750552684	0.001579283	0.003984536
5563	ENSG00000259211.1			1.016490298	0.001580854	0.003987992
5564	ENSG00000286905.1			1.208035901	0.001582882	0.0039926
5565	ENSG00000252577.1	SCARNA20	677681	1.118047501	0.001585489	0.003998668
5566	ENSG00000261773.1			1.063794096	0.001592573	0.004015513
5567	ENSG00000236673.5			1.523792569	0.001596273	0.004023818
5568	ENSG00000211653.2	IGLV1-40		1.127459134	0.001597124	0.004025706
5569	ENSG00000276427.1	IGLL4P		1.689326963	0.001600058	0.004031821
5570	ENSG00000197308.9	GATA3-AS1	399717	1.216866457	0.001601193	0.004034168
5571	ENSG00000243116.1	C9orf78P1		1.324588211	0.001602367	0.00403687
5572	ENSG00000206013.2	IFITM5	387733	1.659520349	0.001602568	0.00403712
5573	ENSG00000213361.2	RPL36P12		1.15991264	0.001607793	0.004049253
5574	ENSG00000276961.1	MIR7848	102466865	1.410065711	0.001615444	0.004068006
5575	ENSG00000211644.3	IGLV1-51		1.297426487	0.001615794	0.004068233
5576	ENSG00000260729.1			1.097101322	0.001615842	0.004068233
5577	ENSG00000267992.1			1.418536453	0.001619612	0.004076947
5578	ENSG00000226816.3			1.138289085	0.001621054	0.004080317
5579	ENSG00000214626.2	POLR3DP1		1.215377347	0.001623522	0.004086011
5580	ENSG00000274367.1			1.01368911	0.001635795	0.004113506
5581	ENSG00000224995.1	LINC01526	101928770	1.237610941	0.001639628	0.004122621
5582	ENSG00000227431.6	CSE1L-DT		1.066457469	0.001661022	0.004172329
5583	ENSG00000254303.1			1.139908661	0.001661185	0.004172329
5584	ENSG00000257604.1			1.253072004	0.00166236	0.004175013
5585	ENSG00000240991.3	RPL23AP67		1.037034506	0.001669201	0.004190335
5586	ENSG00000257126.6	FOXG1-AS1		1.5310619	0.001673823	0.004200607
5587	ENSG00000244474.6	UGT1A4	54657	1.693579269	0.001677031	0.004207585
5588	ENSG00000253175.1			1.199005395	0.001677134	0.004207585
5589	ENSG00000224222.2	LINC02636		1.408074315	0.00167769	0.004208714
5590	ENSG00000242371.1	IGKV1-39		1.298699949	0.00167918	0.004211652
5591	ENSG00000256442.2			1.301386387	0.001694815	0.00424818
5592	ENSG00000239701.1			1.220852159	0.001698747	0.004257228
5593	ENSG00000205424.2	PCBP3-AS1		1.414124121	0.001699612	0.004259128
5594	ENSG00000287300.1		105377848	1.234143746	0.001701765	0.004263714
5595	ENSG00000261435.1			1.300950568	0.00170738	0.004276972
5596	ENSG00000273838.2			1.310814436	0.001712143	0.004287548
5597	ENSG00000250815.1			1.24127469	0.001713454	0.00429029
5598	ENSG00000286266.1			1.504106377	0.001714333	0.00429195
5599	ENSG00000169668.11	BCRP2		1.261321939	0.001725933	0.004317992
5600	ENSG00000163352.5	LENEP	55891	1.138966439	0.001746906	0.004366053
5601	ENSG00000265490.1			1.186033781	0.001764227	0.004405178
5602	ENSG00000225370.1			1.140726226	0.001770691	0.004419649
5603	ENSG00000183760.11	ACP7	390928	1.018893459	0.001789712	0.004464035
5604	ENSG00000248909.1	HMGB1P21		1.132136011	0.001793565	0.004472801
5605	ENSG00000048545.16	GUCA1A	2978	1.281470518	0.001805946	0.00450141
5606	ENSG00000228280.1			1.073514755	0.001813207	0.004517523
5607	ENSG00000268238.1	IGFL1P2		2.253105023	0.001816716	0.004525696
5608	ENSG00000225980.4	OR7E19P		2.092301625	0.00181778	0.004528061
5609	ENSG00000279398.1			1.811246749	0.00182067	0.004534976
5610	ENSG00000211955.2	IGHV3-33		1.161731214	0.001826062	0.004546407
5611	ENSG00000287529.1			1.890745026	0.001835652	0.00456885
5612	ENSG00000288043.1			1.303129273	0.001837927	0.004573649
5613	ENSG00000259104.2	PTCSC3	100886964	1.707681462	0.001849547	0.004599679
5614	ENSG00000285743.1			1.486019104	0.001855432	0.004613448
5615	ENSG00000287029.1			1.096070029	0.001856881	0.00461676
5616	ENSG00000187714.7	SLC18A3	6572	1.962016487	0.001861982	0.004628572
5617	ENSG00000230698.1			1.626751602	0.001863141	0.004630871
5618	ENSG00000274354.1			1.396624382	0.001877027	0.004662787
5619	ENSG00000222640.1	RNU2-51P		1.16279746	0.001877039	0.004662787
5620	ENSG00000240405.7	SAMMSON	101927152	1.06555457	0.0018872	0.00468509
5621	ENSG00000277255.1	MIR7854	102465840	1.156771548	0.001889283	0.004689969
5622	ENSG00000232215.2	OR2L6P		1.775926576	0.001893421	0.004699359
5623	ENSG00000264078.1			1.150109863	0.00189534	0.004703531
5624	ENSG00000241219.1	LOLI1	130932198	1.216848952	0.001904484	0.004724154
5625	ENSG00000245832.7	MIR4300HG	101928989	1.619466972	0.001908221	0.00473224
5626	ENSG00000286580.1			2.419863299	0.001910424	0.004736968
5627	ENSG00000269873.1			1.213989872	0.0019182	0.004754905
5628	ENSG00000239254.1	MSL3P2		1.067346889	0.001936754	0.004797899
5629	ENSG00000258745.1			1.098286374	0.001940938	0.004807363
5630	ENSG00000211594.2	IGKJ4		1.24009075	0.001943927	0.004813862
5631	ENSG00000261359.2	PYCARD-AS1	100652740	1.123643654	0.001944795	0.004815711
5632	ENSG00000264630.5	PRKCA-AS1		1.55376605	0.00195995	0.004849081
5633	ENSG00000270550.1	IGHV3-30		1.098156002	0.001959971	0.004849081
5634	ENSG00000256714.1			1.320609106	0.001963507	0.004857497
5635	ENSG00000201451.1	Y_RNA		1.13630831	0.001964246	0.004859021
5636	ENSG00000205097.7	FRG2	448831	1.79681971	0.001965491	0.004861501
5637	ENSG00000224690.2	UBE2D3P3		1.266522595	0.00196921	0.004869607
5638	ENSG00000229400.1	CNIH3-AS1		2.204711433	0.001969262	0.004869607
5639	ENSG00000243918.1	EIF4BP8		1.034606461	0.001977761	0.004888014
5640	ENSG00000233246.1	PHC2-AS1		1.01999643	0.001984166	0.004901569
5641	ENSG00000226592.1			1.851383748	0.00198477	0.004902756
5642	ENSG00000265496.5			1.185057896	0.001985485	0.004904181
5643	ENSG00000260907.1			1.328970238	0.001988576	0.004910322
5644	ENSG00000130182.8	ZSCAN10	84891	1.30519366	0.001988757	0.004910463
5645	ENSG00000274769.1			1.272545538	0.002001496	0.004939148
5646	ENSG00000222460.1	RN7SKP271		1.042470791	0.002010554	0.004960576
5647	ENSG00000230251.1	PHB1P4		1.390681297	0.002016411	0.004974099
5648	ENSG00000241965.2	RPS2P25		1.554046918	0.002020343	0.004983178
5649	ENSG00000232486.1	DEPDC1P2		1.162011908	0.002027729	0.00499984
5650	ENSG00000256937.1	KRT17P8		1.26932981	0.002037567	0.005021912
5651	ENSG00000258472.9			1.20468853	0.002042916	0.005033531
5652	ENSG00000234289.6	H2BC12L	54145	1.211062431	0.00204514	0.00503807
5653	ENSG00000249993.1	BFSP2-AS1	85003	1.197191144	0.002047147	0.005041761
5654	ENSG00000217159.2	LARP1P1		1.061915624	0.002055772	0.005062061
5655	ENSG00000286478.1			2.082952249	0.002058563	0.005068619
5656	ENSG00000199059.3	MIR135B	442891	1.346947355	0.002061468	0.005075141
5657	ENSG00000282420.1	TRBJ1-2		1.275493395	0.002062649	0.005077732
5658	ENSG00000186509.4	OR9Q1	219956	1.332230458	0.002063697	0.005079367
5659	ENSG00000215869.4	THAP3P1		1.097391996	0.002067144	0.005086588
5660	ENSG00000241582.1	RPL23AP8		1.240915852	0.002069926	0.00509217
5661	ENSG00000231382.1	NBPF21P		1.628901077	0.002070533	0.005093347
5662	ENSG00000248428.1			1.355925702	0.002071787	0.005095655
5663	ENSG00000236478.2			1.089292516	0.002090219	0.005136993
5664	ENSG00000285486.1			1.473567043	0.002099348	0.005156798
5665	ENSG00000231845.3	HMGB3P14		1.033468601	0.002103353	0.005166067
5666	ENSG00000109851.7	DBX1	120237	2.003110147	0.002105625	0.005170688
5667	ENSG00000228549.4	LINC03126	112267871	1.227277826	0.00210703	0.005173818
5668	ENSG00000269043.2		105372316	2.167025219	0.002108143	0.005176229
5669	ENSG00000211970.3	IGHV4-61		1.269943652	0.002109639	0.005179582
5670	ENSG00000259950.1			1.164701709	0.002119353	0.005202466
5671	ENSG00000215481.9	BCRP3	644165	1.616435567	0.002122841	0.005210382
5672	ENSG00000277681.1	MIR6739	102466724	1.126586155	0.002132506	0.005232162
5673	ENSG00000233358.2		105374974	2.331520554	0.00213835	0.005244878
5674	ENSG00000259680.5	IGHV3OR16-17		1.376500675	0.002143806	0.00525761
5675	ENSG00000273321.1			1.030165852	0.002145582	0.00526164
5676	ENSG00000261465.7	COQ7-DT	102723385	1.135255441	0.002146756	0.005264192
5677	ENSG00000284638.1	SMIM44	122405565	1.507419423	0.002147873	0.00526628
5678	ENSG00000287700.1			1.660973876	0.002150595	0.005272301
5679	ENSG00000239513.6	LINC01210	100507274	3.013495237	0.002153397	0.005278519
5680	ENSG00000253756.1	CARS1P2		1.538316611	0.002156915	0.005286162
5681	ENSG00000211676.2	IGLJ2		1.209673749	0.002158926	0.005289783
5682	ENSG00000286780.1			2.291896005	0.002164138	0.005301243
5683	ENSG00000274258.1	MIR6728	102465436	1.179764298	0.002174603	0.005323589
5684	ENSG00000170498.9	KISS1	3814	1.193815263	0.002181613	0.005339101
5685	ENSG00000225899.8	FRG2B	441581	1.9353507	0.002191712	0.005362823
5686	ENSG00000225840.2			1.320872179	0.002199062	0.005379811
5687	ENSG00000287420.1			1.365753907	0.002202235	0.005386909
5688	ENSG00000286076.1			1.54074787	0.002214152	0.005412053
5689	ENSG00000236031.1			1.042102473	0.002217775	0.005419572
5690	ENSG00000285651.1			1.167558376	0.002223624	0.005433197
5691	ENSG00000273824.1			1.154539966	0.002228624	0.005444407
5692	ENSG00000234842.1	MTCO2P16		1.542835345	0.002234568	0.005457919
5693	ENSG00000286016.1			1.586802528	0.002236915	0.005462482
5694	ENSG00000261838.5		105371361	1.279734171	0.002236987	0.005462482
5695	ENSG00000249942.1			1.601407424	0.002241664	0.005472217
5696	ENSG00000248394.1	FOSL1P1		1.012538204	0.002244631	0.005478785
5697	ENSG00000206090.4		100652871	1.894732343	0.002245274	0.00547968
5698	ENSG00000255269.3	LINC02710	105376662	1.645238151	0.002249201	0.005488924
5699	ENSG00000166863.13	TAC3	6866	1.297311213	0.002251991	0.005494382
5700	ENSG00000277186.1			1.328461315	0.002255732	0.005502831
5701	ENSG00000223566.1	TNRC18P2		1.230126784	0.002264442	0.005522041
5702	ENSG00000234396.3			1.4877042	0.002270633	0.005534756
5703	ENSG00000235544.1	RPS11P1		1.401395817	0.00228204	0.005561535
5704	ENSG00000242968.1	SULT1D1P2		1.218625473	0.002284334	0.005566784
5705	ENSG00000276566.1	IGKV1D-13		1.432115187	0.002297529	0.005596187
5706	ENSG00000259254.1			1.254377403	0.002308233	0.005619843
5707	ENSG00000174038.13	SPATA31G1	138724	1.086352038	0.002311238	0.005626814
5708	ENSG00000249140.1	PRDX2P3		1.214027497	0.002312337	0.005629143
5709	ENSG00000276727.1			1.432649797	0.00231353	0.005631702
5710	ENSG00000259721.1	GREM1-AS1	100131315	1.093026027	0.002320734	0.005648198
5711	ENSG00000257464.1			1.053405742	0.002327514	0.005664003
5712	ENSG00000286113.1			1.246302593	0.002343342	0.005699373
5713	ENSG00000188269.10	OR7A5	26659	1.466902267	0.002345359	0.005703605
5714	ENSG00000279532.1			1.067854865	0.002346543	0.005706108
5715	ENSG00000224464.2	PGAM1P6		1.123173153	0.002374442	0.005768644
5716	ENSG00000274276.4		102724560	1.244603708	0.00237585	0.005771709
5717	ENSG00000287161.1			1.353887234	0.002376497	0.005772927
5718	ENSG00000274444.1			1.179419844	0.002378369	0.005777121
5719	ENSG00000260115.1	CDH8-AS1		1.237688428	0.002401237	0.005825529
5720	ENSG00000266448.1			1.943406438	0.002406107	0.00583663
5721	ENSG00000270104.1			1.196923381	0.002420727	0.005868505
5722	ENSG00000165828.15	PRAP1	118471	1.439973547	0.002427903	0.005885182
5723	ENSG00000239503.1	MARK2P8		1.028689369	0.002448176	0.005930698
5724	ENSG00000227339.1	THRAP3P1		1.260731996	0.002448998	0.005932328
5725	ENSG00000236921.1			1.891387583	0.00245371	0.005943015
5726	ENSG00000288050.1			1.353349793	0.002458231	0.005952147
5727	ENSG00000277282.1	IGHV1OR21-1		1.358552941	0.002465122	0.005966858
5728	ENSG00000211965.4	IGHV3-49		1.155552159	0.002469359	0.005976041
5729	ENSG00000232553.3	CLK2P1		1.158496934	0.002474469	0.005986712
5730	ENSG00000135502.19	SLC26A10P		1.447560669	0.002478897	0.005996693
5731	ENSG00000260209.1	LINC03136		1.679964298	0.002483322	0.006006663
5732	ENSG00000228212.1	OFD1P17		1.092678194	0.002485405	0.006011336
5733	ENSG00000213177.3	PRDX2P4		1.081082731	0.002487493	0.00601602
5734	ENSG00000224658.1			1.46883289	0.002492011	0.006025843
5735	ENSG00000229314.5	ORM1	5004	1.421918968	0.00249234	0.00602627
5736	ENSG00000257075.1	RPEP6		1.873180115	0.002514533	0.006076361
5737	ENSG00000271272.1			1.430972692	0.002532672	0.006115957
5738	ENSG00000248916.1	NUP210P3		1.339214667	0.002535732	0.006122228
5739	ENSG00000276410.4	H2BC3	3018	1.169226693	0.002556942	0.006168554
5740	ENSG00000165202.3	OR1Q1	158131	1.512355056	0.002557753	0.006170137
5741	ENSG00000287396.1		124903825	2.026161235	0.002557999	0.006170353
5742	ENSG00000232594.1			2.499012548	0.002590951	0.006243383
5743	ENSG00000278275.2			1.127281666	0.002598088	0.006259059
5744	ENSG00000265790.1	RNASEH1P1		2.857531196	0.002601203	0.006266182
5745	ENSG00000211725.3	TRBV5-5		1.179874819	0.002617585	0.006302583
5746	ENSG00000254098.1	IGKV2-26		1.486568191	0.002618629	0.006304714
5747	ENSG00000240671.4	IGKV1-8		1.244587688	0.002628519	0.006326989
5748	ENSG00000251517.1			1.207317578	0.002635938	0.006344075
5749	ENSG00000168928.13	CTRB2	440387	1.283104744	0.002644641	0.006363476
5750	ENSG00000249509.1	NEUROG2-AS1	105377372	1.871117127	0.002645784	0.006365841
5751	ENSG00000277301.1		101929698	1.075482923	0.002646748	0.006367773
5752	ENSG00000257137.7	LINC02874		1.016154246	0.002651544	0.006378538
5753	ENSG00000263426.2	RN7SL471P		1.465751507	0.002653959	0.00638396
5754	ENSG00000205644.5			1.309186509	0.00266377	0.006404841
5755	ENSG00000254948.1	OR7E158P		1.121683328	0.002664366	0.006405885
5756	ENSG00000270723.1	RPL23AP92		1.1274065	0.002684095	0.006448236
5757	ENSG00000272865.2			1.721842724	0.002687963	0.006456745
5758	ENSG00000237111.1	IGHJ3P		1.269260914	0.002692876	0.006468155
5759	ENSG00000262833.1		101928855	1.152638116	0.002694786	0.00647196
5760	ENSG00000288071.1			1.173057514	0.002705431	0.006495127
5761	ENSG00000159723.5	AGRP	181	1.081451042	0.002720974	0.006530898
5762	ENSG00000252641.2	RNU6-678P		1.203465099	0.002724281	0.006538044
5763	ENSG00000239835.1	PARLP1		1.170204175	0.002736101	0.006564027
5764	ENSG00000253513.1	SQLE-DT		1.156797331	0.002746983	0.006588539
5765	ENSG00000225127.3	LINC00237	105372556	1.435920052	0.002748643	0.006592122
5766	ENSG00000288032.1			1.184551614	0.002773571	0.006647484
5767	ENSG00000287635.1			1.564402391	0.002782222	0.006667007
5768	ENSG00000147432.7	CHRNB3	1142	1.293061932	0.002787776	0.006679107
5769	ENSG00000252174.1	RNU7-18P		1.187066551	0.002791995	0.006688002
5770	ENSG00000284783.1			1.218739075	0.002798935	0.006703223
5771	ENSG00000227717.4	GOLGA6L25	100132202	1.858405861	0.002806346	0.006719536
5772	ENSG00000226141.1			2.838126681	0.002825243	0.006763151
5773	ENSG00000205777.18	GAGE1	2543	3.050051021	0.002844048	0.006804038
5774	ENSG00000279388.1			1.784827878	0.002857177	0.006832582
5775	ENSG00000143105.7	KCNA10	3744	1.544033143	0.002865791	0.006850288
5776	ENSG00000279810.1			1.402170487	0.002869675	0.006858902
5777	ENSG00000244176.1			1.187489138	0.00286974	0.006858902
5778	ENSG00000271130.1	IGHV3OR16-8		1.254868367	0.002872375	0.006864785
5779	ENSG00000239322.1	ATP6V1B1-AS1		1.419539743	0.002924711	0.006980609
5780	ENSG00000196796.5	NPIPB10P		1.149200661	0.002929195	0.006989207
5781	ENSG00000286172.1	RNVU1-8	101447996	1.369516388	0.002934085	0.007000032
5782	ENSG00000286312.1			1.252336499	0.002945152	0.007025168
5783	ENSG00000273933.1			1.19870418	0.002951527	0.007039951
5784	ENSG00000236549.1	RPS27AP7		1.257846995	0.002954788	0.007047304
5785	ENSG00000183535.9	COL18A1-AS1	378832	1.119051498	0.002956015	0.007049808
5786	ENSG00000286063.1			1.515603001	0.002969012	0.007078666
5787	ENSG00000237676.1	RPL30P4		1.004041094	0.002981819	0.007106645
5788	ENSG00000237077.1	RPL38P2		1.260187628	0.002984924	0.007112761
5789	ENSG00000169393.9	ELSPBP1	64100	1.699098456	0.003002722	0.007149586
5790	ENSG00000241956.10	LINC03000		1.231491903	0.003003736	0.007151142
5791	ENSG00000188393.9	CLEC2A	387836	2.140584907	0.003011617	0.007168183
5792	ENSG00000248215.6	LINC02505		2.651957543	0.003014559	0.007174755
5793	ENSG00000260159.1			2.040779962	0.003018657	0.007182784
5794	ENSG00000211648.2	IGLV1-47		1.093771489	0.003045765	0.007242506
5795	ENSG00000254238.1			1.404924365	0.003046609	0.007243642
5796	ENSG00000255156.1	RNY1P9		1.270412199	0.003047688	0.007245775
5797	ENSG00000279006.1			1.153835847	0.003063915	0.007279553
5798	ENSG00000256981.1	TAS2R18P		1.019255537	0.003074981	0.007304093
5799	ENSG00000144820.8	ADGRG7	84873	1.444003643	0.00307738	0.007308916
5800	ENSG00000233308.2	OSTN-AS1	106480738	1.477794383	0.003083251	0.007321982
5801	ENSG00000211958.2	IGHV3-38		1.253083688	0.003085542	0.007326985
5802	ENSG00000176584.18	DMBT1L1		1.746908116	0.003086831	0.007329605
5803	ENSG00000279106.1			1.214597367	0.00310257	0.007362569
5804	ENSG00000286708.1			1.23451181	0.003109974	0.007378814
5805	ENSG00000232117.2	LINC00384		1.142254525	0.003111062	0.007380955
5806	ENSG00000252448.1	SNORA63		1.168493937	0.003114068	0.007387202
5807	ENSG00000259512.3	HNRNPA1P5		1.059141709	0.003122071	0.007403971
5808	ENSG00000241868.3	RN7SL434P		1.333049407	0.003136111	0.007435077
5809	ENSG00000185966.4	LCE3E	353145	1.22862077	0.003141093	0.007445965
5810	ENSG00000233485.1	FHAD1-AS1	101927417	1.354455331	0.003153667	0.007472198
5811	ENSG00000235526.1			1.426920178	0.003158194	0.007481884
5812	ENSG00000230131.6	LINC02641		1.230481709	0.003161778	0.007488729
5813	ENSG00000278462.1			1.203640964	0.003162071	0.007488976
5814	ENSG00000205653.2			1.921489936	0.003164138	0.007492978
5815	ENSG00000240972.2	MIF	4282	1.267831534	0.003167005	0.00749887
5816	ENSG00000281179.1			1.80925119	0.003172776	0.007510292
5817	ENSG00000164007.11	CLDN19	149461	1.311665234	0.003178242	0.007521884
5818	ENSG00000187166.1	H1-7	341567	1.222670137	0.003184324	0.007535378
5819	ENSG00000287359.1		101928463	1.585914049	0.003207315	0.007587519
5820	ENSG00000251018.2	HMMR-AS1		1.200714301	0.003214408	0.007602938
5821	ENSG00000132693.12	CRP	1401	1.340714284	0.003216054	0.00760561
5822	ENSG00000286283.1			2.499156339	0.003217378	0.007608148
5823	ENSG00000276298.1			1.430777277	0.003220211	0.007614057
5824	ENSG00000231295.1			1.689646011	0.003224323	0.007622752
5825	ENSG00000197888.2	UGT2B17	7367	1.09903971	0.003269131	0.007722699
5826	ENSG00000276405.1	TRBV13		1.171818923	0.003269395	0.007722862
5827	ENSG00000211805.1	TRAV24		1.059289604	0.003271827	0.007727686
5828	ENSG00000256789.1	LNCROPM	139225793	1.122975647	0.003280063	0.007745292
5829	ENSG00000268764.1			1.118876847	0.003283869	0.007753356
5830	ENSG00000283167.2			1.313075695	0.003295196	0.007779174
5831	ENSG00000268949.1	MRPS17P1		1.155239851	0.003314774	0.007822598
5832	ENSG00000138083.5	SIX3	6496	1.174539752	0.003317055	0.007827515
5833	ENSG00000272703.1			1.364674745	0.003322067	0.007837009
5834	ENSG00000204019.5	CT83	203413	2.517463446	0.003330873	0.007855447
5835	ENSG00000283504.1			1.763648678	0.003356811	0.007912854
5836	ENSG00000280139.1			1.38491703	0.003357294	0.007913521
5837	ENSG00000253463.1	HMGB1P19		1.135081853	0.003359551	0.0079179
5838	ENSG00000288552.1			2.771514949	0.003360068	0.007918649
5839	ENSG00000265929.1	MIR5195	100847062	1.681098387	0.003362323	0.007923218
5840	ENSG00000285879.1			1.159570441	0.003377907	0.007956434
5841	ENSG00000237501.1	MMACHCP1		1.234076286	0.003379754	0.007959838
5842	ENSG00000254980.1			1.147652361	0.003385664	0.007971862
5843	ENSG00000267676.1	THA1P		1.158194389	0.003394842	0.007992524
5844	ENSG00000257808.1			1.39446645	0.003399971	0.008003173
5845	ENSG00000270178.1			1.449257556	0.003406569	0.008017426
5846	ENSG00000148123.15	PLPPR1	54886	1.297618662	0.003407905	0.00801947
5847	ENSG00000283235.2			1.338977799	0.003416312	0.008038297
5848	ENSG00000273327.1	OR6L2P		1.363178045	0.003423041	0.008052698
5849	ENSG00000266980.1			1.057853988	0.003424742	0.00805622
5850	ENSG00000266124.1	MIR5587	100847028	1.001909583	0.003426163	0.008059086
5851	ENSG00000229769.2	TRBV10-2		1.270634154	0.003434859	0.008077624
5852	ENSG00000266049.1			1.797510775	0.003441781	0.008091982
5853	ENSG00000226690.8	C7orf78	102725191	1.142514457	0.003445624	0.008099577
5854	ENSG00000205274.3	TRBV20OR9-2		1.404297104	0.003447	0.008101851
5855	ENSG00000279650.1			1.385487964	0.003465262	0.008141877
5856	ENSG00000251129.2	LINC02506		1.188043752	0.003466104	0.008143373
5857	ENSG00000236048.2	SSBL5P		1.077383187	0.003467139	0.008145321
5858	ENSG00000228000.1	RPL7AP65		1.195873559	0.003474292	0.008161641
5859	ENSG00000211599.2	IGKV5-2		1.303191967	0.003483338	0.008181925
5860	ENSG00000267316.5			1.07566663	0.003492491	0.008202452
5861	ENSG00000234065.2	MTND4P26		1.014232748	0.003504527	0.008228769
5862	ENSG00000132832.10			1.411381722	0.003513617	0.008248158
5863	ENSG00000073067.14	CYP2W1	54905	1.124653334	0.003530323	0.008285414
5864	ENSG00000268869.6	ESPNP		1.31144562	0.003533258	0.008291321
5865	ENSG00000217385.1	PSMC1P11		1.269220962	0.003539874	0.00830488
5866	ENSG00000230549.3	USP17L1	401447	1.955064332	0.003545858	0.008317935
5867	ENSG00000237923.1	LINC02570	105375014	1.115789051	0.003551988	0.008331327
5868	ENSG00000230801.1	ATP5MFP7		1.12174017	0.00355525	0.008337499
5869	ENSG00000234210.1		101927914	1.070634332	0.003577057	0.008383186
5870	ENSG00000204894.4			1.721863962	0.003590662	0.008413081
5871	ENSG00000229105.1	ASTN2-AS1	100128505	1.258802885	0.003614748	0.008465519
5872	ENSG00000208892.1	SNORA49	677829	1.139347603	0.003627095	0.008490918
5873	ENSG00000249028.2			1.147293649	0.003628333	0.008493315
5874	ENSG00000225657.2	LINC01716	105372682	1.712989878	0.00366402	0.008569264
5875	ENSG00000236739.3	CLIC4P1		1.049083624	0.003687357	0.008620793
5876	ENSG00000267247.1			1.853062598	0.003689922	0.008626281
5877	ENSG00000274011.1	Metazoa_SRP		1.013786265	0.003693986	0.008633746
5878	ENSG00000254418.1	SPON1-AS1		1.138391654	0.003710721	0.008668261
5879	ENSG00000165762.3	OR4K2	390431	1.882796276	0.003729123	0.008707655
5880	ENSG00000261704.2	IGHV1OR16-1		1.439776477	0.003731293	0.008712209
5881	ENSG00000211595.2	IGKJ3		1.21210886	0.003732974	0.008715622
5882	ENSG00000254242.2		105379301	1.328458698	0.003746532	0.008742129
5883	ENSG00000171560.16	FGA	2243	1.584001872	0.003756302	0.008763392
5884	ENSG00000140522.12	RLBP1	6017	1.276695464	0.003763173	0.008775938
5885	ENSG00000187806.9	TMEM202	338949	2.447002132	0.003763235	0.008775938
5886	ENSG00000223305.1	RN7SKP30		1.004745179	0.00377752	0.008807696
5887	ENSG00000275126.2	H4C13	8368	1.241007025	0.003790207	0.008834161
5888	ENSG00000226747.6			1.150404681	0.003792804	0.008839175
5889	ENSG00000186895.4	FGF3	2248	2.697384587	0.003796766	0.008846849
5890	ENSG00000258863.1	SETP1		1.108631698	0.003804387	0.008862003
5891	ENSG00000202227.1	RNU6-282P		1.1103744	0.003811624	0.008878338
5892	ENSG00000102128.8	RAB40AL	282808	1.278171349	0.003815117	0.008885952
5893	ENSG00000266371.1			1.140110101	0.003817134	0.008890128
5894	ENSG00000275162.1			1.199245101	0.003817384	0.008890188
5895	ENSG00000255500.2			1.536198839	0.003824285	0.008905214
5896	ENSG00000254008.1	LINC00051	619434	1.766795857	0.003832626	0.008922017
5897	ENSG00000260710.1	UBE2I-AS1	124903624	1.207391635	0.003838739	0.008934149
5898	ENSG00000253239.1	IGLVI-70		1.425590227	0.003859916	0.008980801
5899	ENSG00000285810.1			1.473910812	0.003863422	0.008986849
5900	ENSG00000228171.1	C11orf98P3		1.192195237	0.003869551	0.009000049
5901	ENSG00000226345.1	ZDHHC1P1		1.165406154	0.003874857	0.009011331
5902	ENSG00000272966.1			1.278365256	0.003884232	0.009031545
5903	ENSG00000228766.3	RPL7L1P8		1.025337132	0.003886158	0.009035494
5904	ENSG00000132446.7	FTHL17	53940	3.330935413	0.003888262	0.009039325
5905	ENSG00000199805.1	RNU1-134P		1.084860947	0.003897108	0.009057235
5906	ENSG00000236862.1	RPS20P24		1.049857906	0.003903668	0.009071418
5907	ENSG00000287876.1			1.870568925	0.003904644	0.009073154
5908	ENSG00000266306.1			1.453117141	0.003913286	0.009091105
5909	ENSG00000213739.3	PPIAP68		1.254345829	0.003918071	0.009100621
5910	ENSG00000287914.1			1.47121048	0.003918929	0.009102081
5911	ENSG00000265262.1	TRMT112P3		1.14627191	0.003938052	0.009142535
5912	ENSG00000277579.1			1.22915436	0.003938422	0.009142535
5913	ENSG00000280425.2			1.302238675	0.003953302	0.009172245
5914	ENSG00000272954.1			1.305026924	0.003957351	0.009181101
5915	ENSG00000241755.1	IGKV1-9		1.103396148	0.00396993	0.00920658
5916	ENSG00000272609.1			1.254059341	0.003993545	0.009257487
5917	ENSG00000273777.5	CEACAM20	125931	1.34867416	0.003997284	0.009264169
5918	ENSG00000277568.1			1.131623776	0.003998648	0.009266608
5919	ENSG00000215049.3	PRDX2P1		1.422544537	0.004009564	0.009290277
5920	ENSG00000249150.2			1.469134959	0.004010623	0.009292186
5921	ENSG00000205976.4			1.823927506	0.004017341	0.009305575
5922	ENSG00000124194.17	GDAP1L1	78997	1.026575408	0.004029997	0.00933162
5923	ENSG00000223831.1	PES1-AS1		1.461154877	0.004044369	0.009358887
5924	ENSG00000215160.3	OR7E122P		1.142766854	0.004044909	0.009359592
5925	ENSG00000254444.1			1.5003263	0.004046272	0.009361107
5926	ENSG00000288551.1			1.134034328	0.004047234	0.009362786
5927	ENSG00000226673.2	LINC01108	102216342	1.049704683	0.004048296	0.009363513
5928	ENSG00000287042.1			1.113723702	0.004048493	0.009363513
5929	ENSG00000279075.1			1.055491475	0.00404886	0.009363816
5930	ENSG00000228294.6	BMS1P17		1.417771298	0.004060159	0.009386113
5931	ENSG00000242412.1	DBIL5P2		1.28789478	0.004060501	0.009386358
5932	ENSG00000253140.2	LINC02947	105375822	1.423925473	0.004068353	0.009402863
5933	ENSG00000216915.2	GPR89P		1.290484665	0.004105504	0.009481543
5934	ENSG00000287515.1			1.389520934	0.004109259	0.009489559
5935	ENSG00000244411.4	KRTAP5-7	440050	1.304777188	0.004109492	0.009489559
5936	ENSG00000270072.1			1.28236974	0.004115315	0.009500327
5937	ENSG00000233792.3	PUDPP2		1.107181214	0.004118397	0.009504675
5938	ENSG00000279714.1			1.214585673	0.004123496	0.00951589
5939	ENSG00000211935.3	IGHV1-3		1.185237062	0.004139553	0.009548498
5940	ENSG00000234197.1	ETV5-AS1	100873934	1.149454251	0.004139872	0.009548678
5941	ENSG00000211666.2	IGLV2-14		1.052938359	0.004143432	0.009555778
5942	ENSG00000255775.1			1.297179666	0.004148891	0.009566697
5943	ENSG00000278999.1			1.023375548	0.004171073	0.009613373
5944	ENSG00000266711.1			2.291710307	0.00417896	0.009629872
5945	ENSG00000205864.1	KRTAP5-6	440023	1.378402572	0.004188812	0.009651453
5946	ENSG00000229229.1			1.696280547	0.00419196	0.009657023
5947	ENSG00000287840.1			1.17736404	0.00422735	0.009732331
5948	ENSG00000236854.2		100271832	1.447903605	0.004249455	0.009779815
5949	ENSG00000106038.13	EVX1	2128	1.532133066	0.004265795	0.009814571
5950	ENSG00000287011.1			1.117423274	0.00427957	0.009843407
5951	ENSG00000204671.2	IL31	386653	1.918112569	0.004292887	0.009872894
5952	ENSG00000211685.3	IGLC7		1.212834271	0.004300095	0.009888323
5953	ENSG00000213218.11	CSH2	1443	2.241119825	0.004301969	0.00989206
5954	ENSG00000245869.2			1.224003236	0.004303174	0.009894255
5955	ENSG00000285639.1			1.189365409	0.00431212	0.009910803
5956	ENSG00000207923.1	MIR559	693144	1.488483737	0.004316811	0.009919858
5957	ENSG00000286481.1			1.513226972	0.004322006	0.009930645
5958	ENSG00000134438.10	RAX	30062	1.448924297	0.004333784	0.009953669
5959	ENSG00000279965.1	LINC03032		1.168302853	0.004345264	0.009975249
5960	ENSG00000188403.7	IGHV1OR15-9		1.268667247	0.004345695	0.009975249
5961	ENSG00000237799.2	CICP11		1.618674441	0.004352376	0.009987692
5962	ENSG00000234586.2			1.819060318	0.004375809	0.010037401
5963	ENSG00000136697.13	IL1F10	84639	1.086202151	0.00438623	0.010058975
5964	ENSG00000243016.1	PRR23E2P		1.613282359	0.004387846	0.0100621
5965	ENSG00000234112.2	LEFTY3P		1.075686538	0.004418726	0.010123544
5966	ENSG00000276521.1			1.651961139	0.004420084	0.010125658
5967	ENSG00000282850.2	RHOXF1P2		2.149198397	0.004428971	0.010144672
5968	ENSG00000276754.1			1.416823976	0.004438359	0.010163826
5969	ENSG00000221611.1		124903100	1.133727794	0.004442465	0.010172054
5970	ENSG00000253263.1			1.155232182	0.00444433	0.01017515
5971	ENSG00000279977.1			1.258637222	0.004455509	0.010197799
5972	ENSG00000279933.1			1.220934358	0.004470218	0.010229693
5973	ENSG00000230005.2	SNAP47-AS1		1.139425938	0.004471078	0.010231072
5974	ENSG00000223808.1			1.918889289	0.004476902	0.010243806
5975	ENSG00000144407.9	PTH2R	5746	1.328929671	0.004515424	0.010324801
5976	ENSG00000196431.4	CRYBA4	1413	1.050688278	0.004526928	0.010347524
5977	ENSG00000249742.2			1.65216024	0.004527915	0.010349184
5978	ENSG00000245719.1	SKOR1-AS1	101929076	1.363970505	0.004529379	0.010351338
5979	ENSG00000211930.1	IGHD3-3		1.582715854	0.004530774	0.010353929
5980	ENSG00000229083.1	PSMA6P2		1.008036072	0.004557922	0.010411767
5981	ENSG00000224585.1			1.319611202	0.004565326	0.010427478
5982	ENSG00000256254.1			1.425417147	0.004574143	0.01044461
5983	ENSG00000254013.1	MAP2K1P1		1.010750387	0.004576603	0.010448422
5984	ENSG00000174358.16	SLC6A19	340024	1.653074937	0.004595155	0.010486548
5985	ENSG00000248555.7	LINC01339	101929495	1.504305658	0.004597785	0.010491343
5986	ENSG00000283227.1	SPRR5	110806278	1.084842469	0.004617781	0.010532122
5987	ENSG00000230133.2	LINC01721	400839	1.41711953	0.004633942	0.010566551
5988	ENSG00000236065.2			1.101006619	0.004634801	0.010567901
5989	ENSG00000226309.1	SMARCE1P2		1.266852757	0.004673319	0.010648378
5990	ENSG00000211804.3	TRDV1		1.060890846	0.004679094	0.010660311
5991	ENSG00000224342.1			1.14344018	0.004684073	0.010671041
5992	ENSG00000259797.1			1.10594622	0.004691901	0.010686418
5993	ENSG00000248783.1		105374655	3.325416856	0.004693425	0.010688858
5994	ENSG00000258203.5			1.305500699	0.004708821	0.010721877
5995	ENSG00000217261.4	POM121L4P		1.040650919	0.004715583	0.01073604
5996	ENSG00000223564.1	CYP4F32P		1.717052183	0.004730501	0.010766928
5997	ENSG00000287388.1			2.072078719	0.004737902	0.010781282
5998	ENSG00000254717.2	GLYATL1P2		1.792839201	0.004741605	0.010787232
5999	ENSG00000261146.1			3.818520893	0.004770976	0.010849074
6000	ENSG00000259092.1	TRAV30		1.045988505	0.004800469	0.010910506
6001	ENSG00000236209.1			1.058695602	0.004815989	0.010942018
6002	ENSG00000187537.13	POTEG	404785	1.188895635	0.004849301	0.011011392
6003	ENSG00000233496.1	SYNJ2-IT1	100874300	1.135692216	0.004854027	0.011019574
6004	ENSG00000239365.2	RPS26P49		1.314307701	0.004864046	0.011038553
6005	ENSG00000232216.1	IGHV3-43		1.146195371	0.004875563	0.011060258
6006	ENSG00000133980.5	VRTN	55237	1.347547296	0.004878049	0.011064
6007	ENSG00000286565.1			1.423146072	0.004884589	0.011077785
6008	ENSG00000264311.1	MIX23P1		1.065512115	0.004884686	0.011077785
6009	ENSG00000226708.1			1.979272758	0.004896042	0.011100999
6010	ENSG00000267535.2	LINC00868	283994	1.527832907	0.004913614	0.011137656
6011	ENSG00000223523.1			1.397303832	0.00492989	0.011171995
6012	ENSG00000211642.3	IGLV10-54		1.263619238	0.004933618	0.011177887
6013	ENSG00000273063.1			1.307115295	0.00495192	0.01121743
6014	ENSG00000229492.1	TERF2IPP1		1.558452921	0.004952311	0.011217675
6015	ENSG00000215720.4	MFFP1		1.247901055	0.004965326	0.011243301
6016	ENSG00000178556.8	CKS1BP6		1.123710302	0.004971184	0.011254637
6017	ENSG00000239381.7			1.414858091	0.004972622	0.011257117
6018	ENSG00000273003.1	ARL2-SNX15		1.200954112	0.004972847	0.011257117
6019	ENSG00000275488.1			1.292143564	0.004986513	0.011286766
6020	ENSG00000287838.1		101927042	1.827898556	0.004992275	0.011297873
6021	ENSG00000255181.3	CCDC166	100130274	1.486958463	0.005010225	0.011332671
6022	ENSG00000258807.5			1.356221254	0.005011698	0.011335358
6023	ENSG00000277587.1			1.009605106	0.005046924	0.011411125
6024	ENSG00000243130.8	PSG11	5680	1.390421242	0.005048396	0.011413801
6025	ENSG00000256325.1			1.074892725	0.005054666	0.011425371
6026	ENSG00000197901.12	SLC22A6	9356	1.906670483	0.005063548	0.011444144
6027	ENSG00000213133.4	PPP1R14BP5		1.050460796	0.005106322	0.011531618
6028	ENSG00000282133.1	TRBJ1-3		1.119043837	0.005107828	0.011534105
6029	ENSG00000287456.1			1.062618531	0.005108006	0.011534105
6030	ENSG00000261310.1			1.997605611	0.005116194	0.01155062
6031	ENSG00000149735.7	GPHA2	170589	1.296830843	0.005125213	0.011568347
6032	ENSG00000226952.1			1.057035816	0.005130491	0.011578942
6033	ENSG00000279147.1			1.049608264	0.005137965	0.01159449
6034	ENSG00000228836.8	CT45A5	441521	2.703584237	0.005147333	0.011611664
6035	ENSG00000187516.7	H2AP	25763	2.211970565	0.005162958	0.011644262
6036	ENSG00000251199.6			1.469798205	0.005178084	0.011673728
6037	ENSG00000263343.1			1.190115359	0.005193088	0.011704891
6038	ENSG00000285711.1	LINC02835		3.243205151	0.005195843	0.011709769
6039	ENSG00000226577.1	MICC		1.3432991	0.005197559	0.011712971
6040	ENSG00000228503.1			1.523798838	0.005199152	0.011714564
6041	ENSG00000270993.1			1.034964259	0.005238096	0.011794268
6042	ENSG00000250071.1	ARPC1AP3		1.074476965	0.005246899	0.011810065
6043	ENSG00000179342.4			1.219072088	0.005277114	0.01186665
6044	ENSG00000261684.2			1.072266757	0.005287585	0.011886984
6045	ENSG00000232044.7	SILC1		1.158168319	0.005312292	0.011937603
6046	ENSG00000224739.2			1.116719312	0.005334531	0.011980788
6047	ENSG00000274600.1	RIMBP3B	440804	1.105763854	0.005359306	0.012033703
6048	ENSG00000287136.1			1.607936058	0.005363925	0.012042027
6049	ENSG00000270889.1	PPP1R14BP4		1.07249107	0.005382738	0.01208221
6050	ENSG00000234255.9		105374780	1.419222789	0.005396139	0.012108179
6051	ENSG00000249175.1			1.179919446	0.005407035	0.012129195
6052	ENSG00000224228.2			1.224597341	0.005419683	0.012156191
6053	ENSG00000260120.2			1.700031035	0.005422906	0.012162732
6054	ENSG00000274775.2	FSIP2LP		1.08507875	0.005438918	0.012193817
6055	ENSG00000184788.14	SATL1	340562	1.029730281	0.00544303	0.012201655
6056	ENSG00000248781.1	PSMC1P4		1.043803741	0.005452143	0.012220012
6057	ENSG00000279778.1			1.09609104	0.005479967	0.012270583
6058	ENSG00000262213.1			2.264910623	0.00548693	0.012285129
6059	ENSG00000261409.1			1.576333942	0.005499363	0.012309148
6060	ENSG00000236123.1	CEACAMP11		1.536831367	0.0055038	0.012318385
6061	ENSG00000278292.1	RFPL4AP6		1.258669042	0.005569416	0.012453293
6062	ENSG00000227417.3			1.0752443	0.005571606	0.012457487
6063	ENSG00000273925.1			1.182094188	0.00557583	0.012465525
6064	ENSG00000173678.14	SPDYE2B	100310812	1.471108579	0.005594812	0.012505142
6065	ENSG00000253593.2		101927822	1.418030099	0.005600627	0.012515316
6066	ENSG00000254616.1			1.686982219	0.005607376	0.012529692
6067	ENSG00000275801.1			1.183236505	0.005616563	0.012548806
6068	ENSG00000233847.1	ERCC8-AS1		1.054513235	0.005621915	0.01255961
6069	ENSG00000268336.1	SIGLEC20P		1.603367677	0.005634796	0.012585288
6070	ENSG00000237390.1			1.103679949	0.005647589	0.01261031
6071	ENSG00000269806.1	BICRA-AS1		1.40254614	0.005655774	0.012625346
6072	ENSG00000110245.12	APOC3	345	2.471125567	0.005655916	0.012625346
6073	ENSG00000232518.3	GALM-AS1		1.130550897	0.005681026	0.012677115
6074	ENSG00000206712.1	RNU6-26P		1.031744153	0.00569088	0.01269839
6075	ENSG00000237410.1	NRXN2-AS1		1.112448574	0.005695272	0.012706759
6076	ENSG00000255639.3			1.546535104	0.005698922	0.012714189
6077	ENSG00000156925.12	ZIC3	7547	2.445983331	0.005703561	0.012722324
6078	ENSG00000286232.1			1.531419154	0.005720614	0.012754689
6079	ENSG00000272006.1			1.030609424	0.005728192	0.012769429
6080	ENSG00000207484.1	Y_RNA		1.231242775	0.005729729	0.012772139
6081	ENSG00000260664.2			1.112569124	0.005750304	0.012815726
6082	ENSG00000261037.1			1.298670721	0.005767727	0.012850336
6083	ENSG00000285818.1		100131107	1.188538168	0.005776447	0.012868319
6084	ENSG00000267765.1			1.145598074	0.005786833	0.012890733
6085	ENSG00000261421.1			1.708315417	0.005807607	0.012931195
6086	ENSG00000251391.5			1.389915805	0.00581837	0.012950798
6087	ENSG00000276747.1	PADI6	353238	1.208312292	0.005848037	0.01301026
6088	ENSG00000247213.7	LINC01498	102723562	1.328371689	0.005867105	0.013051214
6089	ENSG00000183305.13	MAGEA2B	4101	2.233053581	0.005898163	0.013117358
6090	ENSG00000254760.1	SIGLEC10-AS2		1.093718667	0.005911177	0.013141141
6091	ENSG00000255539.1			1.304339374	0.005919882	0.013156805
6092	ENSG00000265243.1	IGLJCOR18		1.820656738	0.005922046	0.013160878
6093	ENSG00000264659.2			1.308997397	0.005930724	0.013175732
6094	ENSG00000261751.1			1.670207528	0.005933902	0.013182054
6095	ENSG00000188850.9			1.183899711	0.005935355	0.013184542
6096	ENSG00000261161.1			1.459827996	0.005937727	0.013188333
6097	ENSG00000263388.1			1.08787705	0.005945284	0.01320301
6098	ENSG00000213358.3	NAA50P2		1.175315322	0.005966617	0.013248049
6099	ENSG00000260247.1	SUB1P4		1.30728999	0.005975815	0.013264757
6100	ENSG00000286134.2			1.470747866	0.005992198	0.013295913
6101	ENSG00000280070.1			1.298291342	0.005994788	0.01330017
6102	ENSG00000211968.3	IGHV1-58		1.255494057	0.006001916	0.013314494
6103	ENSG00000285698.1			1.42137273	0.006037367	0.013387146
6104	ENSG00000258527.1	ASB9P1		1.00510749	0.006050882	0.013416364
6105	ENSG00000253528.2			1.116319515	0.006055358	0.013424036
6106	ENSG00000176566.5	DCAF4L2	138009	3.358703986	0.006073089	0.013461837
6107	ENSG00000256564.1			1.074906622	0.006078184	0.013472379
6108	ENSG00000260765.2	CES1P2		1.209121045	0.006085594	0.013486005
6109	ENSG00000218632.3	RPL7P28		1.231872522	0.006116191	0.013542993
6110	ENSG00000273555.1	MIR6812	102465487	1.131167112	0.006121554	0.013553355
6111	ENSG00000006611.17	USH1C	10083	1.292642388	0.006146769	0.013604624
6112	ENSG00000287689.1			1.970833079	0.00616435	0.013639729
6113	ENSG00000248975.2			1.579520909	0.006188626	0.013686793
6114	ENSG00000234818.1			1.05142302	0.006204669	0.0137144
6115	ENSG00000242199.1	RPS26P22		1.155169651	0.006211479	0.013727157
6116	ENSG00000227726.1			1.355586732	0.006234468	0.013771054
6117	ENSG00000237001.7	WASF3-AS1		1.476333339	0.006249604	0.013799106
6118	ENSG00000267637.1			1.053992229	0.006275614	0.013851908
6119	ENSG00000231208.4	ZBTB46-AS1		2.217872946	0.006277235	0.013854714
6120	ENSG00000269480.1			1.009600732	0.006287767	0.013876414
6121	ENSG00000266955.1			1.06382641	0.006290363	0.013881371
6122	ENSG00000267291.1	MAP4K1-AS1	105372397	1.376431863	0.006306483	0.013913847
6123	ENSG00000265813.2	RN7SL300P		1.190258352	0.006311509	0.013922612
6124	ENSG00000236668.2	LHX2-AS1	100505588	1.712500994	0.006314221	0.013927819
6125	ENSG00000256995.8	LINC02955	107984516	1.257605798	0.006315702	0.01393031
6126	ENSG00000286831.1			1.039127027	0.006323076	0.013944247
6127	ENSG00000283906.1	IGHV4-80		1.125014664	0.006360857	0.01401899
6128	ENSG00000228031.4			1.455992554	0.006387441	0.014070539
6129	ENSG00000226126.2	PSMC1P12		1.309904457	0.006413409	0.014122251
6130	ENSG00000236867.1			1.054403721	0.006423029	0.014140293
6131	ENSG00000253515.2		124906002	1.141912619	0.006437208	0.014167574
6132	ENSG00000258998.2			1.778189956	0.006457682	0.014208692
6133	ENSG00000253168.1			1.251437224	0.006477358	0.014248821
6134	ENSG00000251768.1	RNA5SP217		1.023734669	0.00650624	0.01430779
6135	ENSG00000236046.1			1.438702647	0.006516734	0.014328287
6136	ENSG00000264845.2			1.54604035	0.006521208	0.014336532
6137	ENSG00000100557.10	CCDC198	55195	1.392833632	0.006526058	0.014344243
6138	ENSG00000178107.2			1.790705308	0.006540664	0.014373728
6139	ENSG00000230611.1	HMGB1P27		1.037649408	0.006546713	0.014384629
6140	ENSG00000267703.1			1.367608452	0.00654897	0.01438879
6141	ENSG00000257226.2			1.10182408	0.006564362	0.01442101
6142	ENSG00000226203.2	LINC01733	101926955	1.510333283	0.006579504	0.014449942
6143	ENSG00000225564.6	LINC01492	101928496	1.342540783	0.006587883	0.014466732
6144	ENSG00000122728.6	TAF1L	138474	1.000192417	0.006588093	0.014466732
6145	ENSG00000235763.1	SNRPGP5		1.136013557	0.006599876	0.014491001
6146	ENSG00000277892.1	MIR6746	102465446	1.164949898	0.006602419	0.014494979
6147	ENSG00000248522.1	SBF1P1	100133234	1.437563067	0.006604547	0.014498847
6148	ENSG00000242887.1	IGHJ3		1.056302817	0.006627528	0.014542378
6149	ENSG00000274827.4	LINC01297		2.01137803	0.006627677	0.014542378
6150	ENSG00000224655.7	LINC01823	101927801	1.066384116	0.0066455	0.014580075
6151	ENSG00000226567.2	LINC00606	285370	1.298658434	0.006645593	0.014580075
6152	ENSG00000221844.2	DPP3P2		1.726283448	0.006652436	0.014593187
6153	ENSG00000259079.1			1.02138789	0.006661799	0.014609972
6154	ENSG00000224541.1			1.128330629	0.006667687	0.014621266
6155	ENSG00000253952.1	HIGD1AP18		1.125672842	0.006671459	0.01462873
6156	ENSG00000249584.1	LINC02225	101928539	1.107523044	0.006684483	0.014656477
6157	ENSG00000174495.12	PPIAP80		1.093934906	0.006690579	0.01466822
6158	ENSG00000182931.9	WFDC10B	280664	1.216509696	0.006694159	0.014675257
6159	ENSG00000163217.2	BMP10	27302	1.033444081	0.00670344	0.014693166
6160	ENSG00000279501.1			2.096772423	0.006704798	0.014695129
6161	ENSG00000229356.1	LRRC3-DT	100861510	1.117521639	0.006705076	0.014695129
6162	ENSG00000165204.3	OR1K1	392392	1.766401605	0.006707196	0.014698962
6163	ENSG00000240106.2	RN7SL146P		1.026884002	0.00673671	0.014753856
6164	ENSG00000234066.1	TAS2R62P		1.265295181	0.006762989	0.01480732
6165	ENSG00000248694.1			1.398026914	0.006767459	0.014814653
6166	ENSG00000165066.13	NKX6-3	157848	1.74701512	0.006779283	0.014835623
6167	ENSG00000223443.2	USP17L2	377630	1.693361882	0.006781306	0.01483923
6168	ENSG00000266885.2			1.93722481	0.006782154	0.014840267
6169	ENSG00000250954.6		101928622	2.659364943	0.006789724	0.014852733
6170	ENSG00000230894.2		107987014	1.051032641	0.006833757	0.014944933
6171	ENSG00000168148.4	H3-4	8290	1.211911734	0.006835686	0.014948326
6172	ENSG00000239969.5			1.012763466	0.006846147	0.014966103
6173	ENSG00000170279.3	C7orf33	202865	2.070506401	0.006887698	0.0150463
6174	ENSG00000251880.1	RNU7-75P		1.054256033	0.006913594	0.015096217
6175	ENSG00000277718.1	LINC02255		1.496246799	0.006967065	0.015200232
6176	ENSG00000211647.1	IGLV5-48		1.246790951	0.006970104	0.015204536
6177	ENSG00000249363.1	TRPC6P2		1.03572221	0.006982208	0.015225915
6178	ENSG00000207588.1	MIR593	693178	1.060183484	0.006984259	0.015229225
6179	ENSG00000279024.1			1.29604792	0.006984495	0.015229225
6180	ENSG00000211943.2	IGHV3-15		1.014312894	0.007012081	0.015286013
6181	ENSG00000144015.5	TRIM43	129868	2.254872028	0.007035397	0.01533473
6182	ENSG00000233196.2			1.143096531	0.007041927	0.0153477
6183	ENSG00000170790.5	OR10A2	341276	1.078301474	0.007044959	0.015352621
6184	ENSG00000268834.2	OR7A1P		2.114162595	0.007060838	0.015382998
6185	ENSG00000274589.1	Metazoa_SRP		1.019144593	0.007083483	0.015429789
6186	ENSG00000279825.1			1.129427893	0.007139611	0.015542577
6187	ENSG00000282911.1	DUX4L35		1.802913314	0.007147572	0.01555828
6188	ENSG00000285699.1			1.57297595	0.007150113	0.015562427
6189	ENSG00000227348.1	MTND5P25		1.241057215	0.007150262	0.015562427
6190	ENSG00000227096.1	HMGB3P8		1.265716033	0.007153609	0.015568857
6191	ENSG00000259847.2	LINC02126		1.139939207	0.007184015	0.015630742
6192	ENSG00000280095.1			1.243327533	0.007188315	0.015638382
6193	ENSG00000189108.13	IL1RAPL2	26280	1.084052715	0.007195408	0.015652953
6194	ENSG00000181965.6	NEUROG1	4762	1.683441117	0.007245196	0.01575325
6195	ENSG00000234816.2	H2AC5P		1.205280976	0.007248076	0.015757154
6196	ENSG00000226761.3	TAS2R46	259292	1.071767226	0.007251621	0.015763995
6197	ENSG00000151963.4	ZNF37CP		1.047080545	0.00725954	0.015780345
6198	ENSG00000253229.1	HIGD1AP6		1.1792372	0.007282836	0.015824911
6199	ENSG00000279222.1			1.866922687	0.007292329	0.015844672
6200	ENSG00000270863.1	DDX55P1		1.027499154	0.007299157	0.015858376
6201	ENSG00000271049.1			1.065104838	0.007299437	0.015858376
6202	ENSG00000264196.1			1.127850254	0.007303805	0.015866127
6203	ENSG00000201944.1	SNORA72	124900423	1.058791805	0.007308727	0.015874212
6204	ENSG00000234761.1	MGAT4FP		1.4109631	0.007310084	0.015876289
6205	ENSG00000262339.5			1.041063085	0.007310756	0.015876878
6206	ENSG00000240382.3	IGKV1-17		1.043957997	0.007334945	0.015925921
6207	ENSG00000244676.5			1.030078894	0.007349882	0.015955731
6208	ENSG00000285254.2		124902392	1.254487033	0.00735464	0.015964775
6209	ENSG00000237064.1	EIF3IP1	442720	1.00950146	0.00737731	0.016007704
6210	ENSG00000250579.2	ADAMTS16-DT		1.212884096	0.007396989	0.016043062
6211	ENSG00000261697.5		101928682	1.230970197	0.007411772	0.016071672
6212	ENSG00000215867.4	KRT18P57		1.115890167	0.007429563	0.016104901
6213	ENSG00000284952.1			1.130819951	0.007433291	0.016111221
6214	ENSG00000286829.1			1.425033669	0.007437706	0.016119909
6215	ENSG00000237633.4			1.113441738	0.007445868	0.016133189
6216	ENSG00000248588.2			1.490598031	0.007453414	0.016147773
6217	ENSG00000201464.1	Y_RNA		1.641262067	0.007454198	0.01614859
6218	ENSG00000224458.3	GUSBP6		1.930757204	0.007493599	0.016218571
6219	ENSG00000223850.1	MYCNUT	103752554	1.47655938	0.007493864	0.016218571
6220	ENSG00000229092.2	IGHV3-47		1.104193416	0.007498334	0.016227359
6221	ENSG00000284928.1			1.158205813	0.007500466	0.016231086
6222	ENSG00000230390.1	LINC01048	103695431	1.267545309	0.007532906	0.016293283
6223	ENSG00000287351.1			1.049797099	0.007557766	0.016341706
6224	ENSG00000250167.1			1.224558378	0.007559061	0.016343614
6225	ENSG00000235189.2			1.031387575	0.007577345	0.016380467
6226	ENSG00000167612.13	ANKRD33	341405	1.169291442	0.007580254	0.016385862
6227	ENSG00000275158.1	TRBV12-5		1.327042264	0.007608747	0.016438492
6228	ENSG00000286592.1	LINC02885		1.005541283	0.007635922	0.016490015
6229	ENSG00000253273.2			1.065315386	0.007641918	0.016501166
6230	ENSG00000231840.2	TMEM139-AS1		1.054593172	0.007648967	0.01651369
6231	ENSG00000142025.16	DMRTC2	63946	1.685578108	0.007653773	0.01652183
6232	ENSG00000226947.1	LCEP4		1.201617876	0.007658582	0.016530846
6233	ENSG00000224802.2	TUBB4BP2		1.24076772	0.007661233	0.016535668
6234	ENSG00000228050.1	TOP3BP1		1.30172527	0.007666527	0.016545294
6235	ENSG00000265874.1	MIR4489	100616284	1.03550565	0.007675532	0.016563825
6236	ENSG00000263862.2	LINC01543		1.108164915	0.007719994	0.016655899
6237	ENSG00000131482.10	G6PC1	2538	1.341132637	0.00772778	0.016670225
6238	ENSG00000249840.2	GAPDHP76		1.13053652	0.007729262	0.016672516
6239	ENSG00000248796.1	MED15P8		2.339167616	0.007739566	0.016692926
6240	ENSG00000233492.2	BETALINC1		2.210507169	0.007780424	0.016776487
6241	ENSG00000143107.10	FNDC7	163479	1.187574066	0.007786739	0.016787365
6242	ENSG00000240521.1			1.257189123	0.00782647	0.016870268
6243	ENSG00000263331.1			1.32487318	0.007838353	0.016891861
6244	ENSG00000176654.12	NANOGP1		1.31120619	0.007911429	0.017035803
6245	ENSG00000249699.1	LINC02261	101929199	1.550915015	0.007918872	0.017049052
6246	ENSG00000287786.1			1.302779704	0.007921451	0.017052753
6247	ENSG00000165606.9	DRGX	644168	1.269876199	0.007925125	0.01705881
6248	ENSG00000260599.1		101929124	1.693137921	0.007938042	0.017082903
6249	ENSG00000256518.3	FOLR1P1		1.580736859	0.007952156	0.017109563
6250	ENSG00000249098.1	CSRP2P2		1.403728768	0.007957694	0.017119903
6251	ENSG00000072315.3	TRPC5	7224	1.149328087	0.00795967	0.017122069
6252	ENSG00000137392.10	CLPS	1208	1.853970886	0.007959696	0.017122069
6253	ENSG00000257004.1			1.211580581	0.007961498	0.017125017
6254	ENSG00000164334.15	FAM170A	340069	1.247723065	0.007985429	0.017171834
6255	ENSG00000233064.2			1.329554969	0.008029563	0.017256444
6256	ENSG00000258844.2			1.428432326	0.008036456	0.017268451
6257	ENSG00000254080.1	LINC03071		1.594934227	0.008060897	0.017315339
6258	ENSG00000234223.2			1.280671108	0.008069152	0.017331192
6259	ENSG00000265394.1			1.093278104	0.008087813	0.017365629
6260	ENSG00000252652.1	Y_RNA		1.067349848	0.008093166	0.01737524
6261	ENSG00000234921.1			1.429359199	0.008100568	0.017389249
6262	ENSG00000273628.1			1.023710201	0.008102555	0.017392572
6263	ENSG00000226443.3	GAPDHP32		1.06197974	0.008140718	0.017469044
6264	ENSG00000257078.1			1.980862113	0.008205892	0.017597242
6265	ENSG00000235091.2			1.2643747	0.008283405	0.017747149
6266	ENSG00000199536.1	RNU6-315P		1.037981926	0.008289211	0.017757668
6267	ENSG00000242999.3	RN7SL239P		1.045456544	0.008297847	0.017773524
6268	ENSG00000257264.5			1.094727598	0.008297856	0.017773524
6269	ENSG00000232716.2			1.079905066	0.008310004	0.017796443
6270	ENSG00000259922.1	CFAP69P1		1.068698178	0.008332092	0.017841819
6271	ENSG00000215606.4	KRT18P35		1.346024618	0.008332993	0.017842735
6272	ENSG00000214301.4	MAF1P1		1.123864768	0.008340554	0.017857046
6273	ENSG00000265334.1			1.125809852	0.008390932	0.017958867
6274	ENSG00000256274.1	TAS2R64P		1.02366156	0.008393072	0.017961727
6275	ENSG00000197487.9	GALP	85569	1.852505327	0.008402797	0.017979629
6276	ENSG00000228739.1			1.312898959	0.00841052	0.017993295
6277	ENSG00000240328.1			1.079378005	0.008432745	0.01803884
6278	ENSG00000182070.6	OR52A1	23538	1.3889262	0.008467709	0.01810658
6279	ENSG00000224924.7	LINC00320	387486	1.631284603	0.008520311	0.018208474
6280	ENSG00000203434.2			2.098446839	0.008536763	0.0182397
6281	ENSG00000214761.3	HNRNPA1P15		1.000318186	0.008567836	0.018300173
6282	ENSG00000266979.1	LINC01180	101927017	1.430174036	0.008584212	0.018332188
6283	ENSG00000270035.1			1.117266234	0.008595042	0.018351364
6284	ENSG00000259946.1			1.788488503	0.008625394	0.018409229
6285	ENSG00000270864.1	IGHV3OR16-6		1.211135555	0.008658738	0.018473435
6286	ENSG00000232455.2	LARS2-AS1		1.183888345	0.008679122	0.018513934
6287	ENSG00000202402.1	Y_RNA		2.760243333	0.008703426	0.018560787
6288	ENSG00000272121.1			1.037926495	0.008708344	0.018569276
6289	ENSG00000255029.2			1.671635026	0.00872184	0.018595055
6290	ENSG00000273853.1			2.007070302	0.008725685	0.018602251
6291	ENSG00000286385.1			2.919523111	0.008737231	0.018623789
6292	ENSG00000211904.2	IGHJ2		1.110178152	0.008737666	0.018623789
6293	ENSG00000205642.10	VCX3B	425054	1.219921956	0.008746237	0.018639052
6294	ENSG00000257761.3			1.150799041	0.008758541	0.018661904
6295	ENSG00000260347.1	MOCS1P1		1.464362429	0.008763004	0.018667761
6296	ENSG00000224391.1	LINC01280		1.619249422	0.008779664	0.018699231
6297	ENSG00000230515.1			1.124696409	0.00882891	0.018794656
6298	ENSG00000241361.8	SLC25A24P1		1.924727554	0.008860431	0.018847965
6299	ENSG00000229456.1	RLIMP1		1.015658933	0.008876393	0.018877869
6300	ENSG00000176840.12	MIR7-3HG	284424	1.23464636	0.008888823	0.018902275
6301	ENSG00000225877.1	PSG8-AS1	100289650	1.422152299	0.008890235	0.018904264
6302	ENSG00000224604.1			1.19275564	0.008920854	0.018954125
6303	ENSG00000105141.6	CASP14	23581	1.149737919	0.008944069	0.019000397
6304	ENSG00000279884.1			1.070552635	0.008953669	0.019018753
6305	ENSG00000261369.1	ITFG1-AS2		1.198409805	0.008955814	0.019022289
6306	ENSG00000206846.1	Y_RNA		1.100161183	0.008981472	0.019064531
6307	ENSG00000287301.1			1.255086069	0.008987526	0.019076361
6308	ENSG00000231364.3	LINC01712	105375358	1.40368769	0.008999521	0.019100798
6309	ENSG00000253359.1	IGHV3-37		1.390679536	0.009009965	0.019120655
6310	ENSG00000254395.1	IGHV4-55		1.008041164	0.009022206	0.019139723
6311	ENSG00000223584.1	TVP23CP1		1.306713142	0.009062522	0.019217024
6312	ENSG00000211821.2	TRDV2		1.261712175	0.009095808	0.019282452
6313	ENSG00000254852.8	NPIPA2	642799	1.436957715	0.009102599	0.019295817
6314	ENSG00000239855.1	IGKV1-6		1.000728422	0.009110782	0.019310065
6315	ENSG00000265442.1	MIR3941	100500866	1.164751139	0.009137531	0.019361585
6316	ENSG00000260009.1	LINC02130		1.230198796	0.009144805	0.01937389
6317	ENSG00000235934.2			1.03160181	0.00917519	0.019435149
6318	ENSG00000231586.2	RPS23P9		1.499068376	0.009178398	0.019440906
6319	ENSG00000259133.5			1.941599465	0.009194591	0.019472084
6320	ENSG00000226668.5	SPATA31B2P		1.638246213	0.009238797	0.019560478
6321	ENSG00000264028.2	RN7SL744P		1.002463809	0.009263861	0.019608308
6322	ENSG00000259631.1			1.07386808	0.009272178	0.019624865
6323	ENSG00000242580.1	IGKV1D-43		1.226745385	0.009274372	0.01962846
6324	ENSG00000277010.1			1.14915659	0.009296608	0.019669221
6325	ENSG00000141946.1	ZIM3	114026	1.439794237	0.009302509	0.019680656
6326	ENSG00000255875.2	PABPN1P2		1.055773235	0.009306178	0.019687367
6327	ENSG00000265203.2	RBP3	5949	1.052939078	0.009362171	0.019797371
6328	ENSG00000255395.1			1.239621714	0.009373998	0.019821325
6329	ENSG00000253230.9	MIR124-1HG	157627	1.508014131	0.009387457	0.019846609
6330	ENSG00000265094.1			1.03640037	0.009425214	0.019918998
6331	ENSG00000287624.1			1.087605232	0.009439586	0.019948307
6332	ENSG00000252279.1	RNU6-406P		1.484465611	0.009455422	0.019977513
6333	ENSG00000188101.5	ALOX15P2		1.781673107	0.00947435	0.020015373
6334	ENSG00000149635.3	OCSTAMP	128506	1.155216889	0.009533975	0.020128465
6335	ENSG00000242531.1			1.096119422	0.009541438	0.02014315
6336	ENSG00000276476.3	LINC00540	100506622	1.126432337	0.009559552	0.020178166
6337	ENSG00000238117.1	NTM-AS3	139355216	1.485663621	0.009609531	0.020273948
6338	ENSG00000286713.1			1.457662601	0.009615952	0.020286415
6339	ENSG00000275613.2			1.420924442	0.009621298	0.020294454
6340	ENSG00000211978.2	IGHV5-78		1.074730019	0.009657995	0.020358863
6341	ENSG00000223220.1	Y_RNA		1.084345121	0.009659241	0.020360406
6342	ENSG00000144035.4	NAT8	9027	1.169351441	0.009736184	0.020505149
6343	ENSG00000242324.1			1.045082715	0.009746226	0.020523029
6344	ENSG00000270986.1	HMGB1P51		1.036338611	0.009820644	0.020661998
6345	ENSG00000231682.1	LINC01891	105373934	1.144466823	0.00983389	0.020684556
6346	ENSG00000250891.2	LINC02208		1.084128744	0.009859053	0.020730297
6347	ENSG00000211813.2	TRAV34		1.084939245	0.009859295	0.020730297
6348	ENSG00000228717.1	NAB1P1		1.107595606	0.009897563	0.020803036
6349	ENSG00000229111.1	MED4-AS1	100873965	1.002844283	0.009908434	0.020820368
6350	ENSG00000280655.1			1.390022127	0.009937357	0.020876717
6351	ENSG00000233625.1			1.173492297	0.009940046	0.020880152
6352	ENSG00000260840.2	LINC01964	100996478	1.266462413	0.009960881	0.020920593
6353	ENSG00000267590.1	NDUFA3P1		1.004226646	0.009961607	0.02092101
6354	ENSG00000230535.1	BASP1P1		1.733757477	0.009989104	0.020971357
6355	ENSG00000203811.1	H3C14	126961	1.377987806	0.010014843	0.021018745
6356	ENSG00000285525.1			1.573468718	0.010046516	0.021080351
6357	ENSG00000232407.3	PPIAP70		1.018014654	0.010066497	0.02111669
6358	ENSG00000286739.1		102724497	1.00144893	0.010071329	0.021125708
6359	ENSG00000145321.13	GC	2638	2.65654201	0.010081205	0.021144187
6360	ENSG00000231087.2	FDPSP7		1.014532979	0.01012282	0.021223611
6361	ENSG00000285577.1			1.067182977	0.01013837	0.021254978
6362	ENSG00000232080.5		102725019	1.944542443	0.010156265	0.021288102
6363	ENSG00000285527.1			1.256172856	0.010208885	0.021389351
6364	ENSG00000235454.1	HAUS6P3		1.068077103	0.010234089	0.021434323
6365	ENSG00000249867.6	LINC02742	105376603	1.566067363	0.010239206	0.021441549
6366	ENSG00000262154.1	EIF1P4		1.289381411	0.010286632	0.021531764
6367	ENSG00000213958.2	KRT18P29		1.066503349	0.010402761	0.021746142
6368	ENSG00000229798.1	KRT18P26		1.145621755	0.010410675	0.021761538
6369	ENSG00000211672.2	IGLV4-3		1.2509958	0.010435487	0.021806982
6370	ENSG00000196734.9	LCE1B	353132	1.059656898	0.010435716	0.021806982
6371	ENSG00000201608.1	RNU4-42P		1.145676376	0.010483701	0.021899175
6372	ENSG00000265121.1			1.014092115	0.010514652	0.021958044
6373	ENSG00000235627.1	SNRPFP4		1.206134425	0.010534831	0.02199671
6374	ENSG00000284744.1			1.377298466	0.010580186	0.022082107
6375	ENSG00000121351.8	IAPP	3375	1.468923277	0.010584757	0.022089322
6376	ENSG00000256542.2			1.176006154	0.010626167	0.022168739
6377	ENSG00000225280.7		105372558	2.955776658	0.010660368	0.022231903
6378	ENSG00000251292.1	ERVH-1		1.097407645	0.010674622	0.022256946
6379	ENSG00000248949.1			1.186955594	0.01073662	0.022376801
6380	ENSG00000288068.1			1.25937663	0.010745665	0.022390943
6381	ENSG00000285919.1			1.185021209	0.010764746	0.022429525
6382	ENSG00000254789.3			1.289742075	0.010839465	0.022576903
6383	ENSG00000241438.1	CRIPTOP6		1.054119991	0.010841723	0.022579235
6384	ENSG00000234159.1	RBPMSLP		1.630787873	0.010901552	0.022688343
6385	ENSG00000226134.1			1.535522486	0.01093998	0.022762347
6386	ENSG00000157005.4	SST	6750	1.334098652	0.010989211	0.022857582
6387	ENSG00000255532.1	NOX4P1		1.317750044	0.011010952	0.022898553
6388	ENSG00000278486.1	YRDCP1		1.210597062	0.011034545	0.022941045
6389	ENSG00000269321.1			1.006822663	0.011046395	0.022959661
6390	ENSG00000249020.1	SNORA58		1.024634975	0.011067306	0.022998303
6391	ENSG00000270685.1	IGHV1OR15-6		1.543309951	0.011121563	0.023102576
6392	ENSG00000189186.10	DCAF8L2	347442	2.454211819	0.011209042	0.023267226
6393	ENSG00000207277.1	RNA5SP284		1.303563301	0.011250224	0.023342433
6394	ENSG00000287677.2			1.562107776	0.011263418	0.023365224
6395	ENSG00000101204.18	CHRNA4	1137	1.18880778	0.011265926	0.023366955
6396	ENSG00000253361.3			1.331943133	0.011305778	0.023441028
6397	ENSG00000275016.5			1.802718428	0.011308254	0.023444936
6398	ENSG00000224109.3	CENPVL3	347549	2.610910748	0.01131154	0.02344942
6399	ENSG00000261673.1			1.014372953	0.011312191	0.02344942
6400	ENSG00000267656.1			1.194898135	0.011329926	0.02347391
6401	ENSG00000229335.1	DANT1		2.379098266	0.01135356	0.023515504
6402	ENSG00000105205.7	CLC	1178	1.092049211	0.011402227	0.023603973
6403	ENSG00000285895.1			1.067337095	0.011431358	0.023660571
6404	ENSG00000131914.11	LIN28A	79727	1.138338252	0.011456294	0.023699812
6405	ENSG00000253709.1	IGHV1-14		1.26750947	0.011476897	0.023737479
6406	ENSG00000207034.1	Y_RNA		1.023827053	0.011519105	0.023819333
6407	ENSG00000276575.1	MIR6895	102465539	1.086247889	0.01153557	0.023847636
6408	ENSG00000243478.9	AOX2P		1.097254381	0.011606642	0.023977061
6409	ENSG00000257302.1	FAHD2P1		1.572818868	0.01161533	0.023993145
6410	ENSG00000265916.1			1.08774356	0.01163677	0.024035544
6411	ENSG00000279829.1			1.099139545	0.011654928	0.024068032
6412	ENSG00000095777.17	MYO3A	53904	1.101569616	0.011695254	0.024139993
6413	ENSG00000244321.1	LINC01326		2.020797746	0.011706169	0.02415875
6414	ENSG00000225380.4	IGKV1OR9-2		1.28580007	0.011719859	0.024181968
6415	ENSG00000188691.5	OR56A5	390084	1.642807573	0.011744482	0.024225209
6416	ENSG00000197549.9	PRAMENP		1.236233888	0.011755593	0.024243082
6417	ENSG00000237735.2			1.289616478	0.011757516	0.024245786
6418	ENSG00000278996.1			1.83294547	0.011761112	0.024251185
6419	ENSG00000270472.2	IGHV3OR16-9		1.067973813	0.011761357	0.024251185
6420	ENSG00000224928.2	KRT8P30		1.240998216	0.011818536	0.02435895
6421	ENSG00000258081.4			1.314562141	0.011819166	0.024358983
6422	ENSG00000223631.2	LINC01120		1.382881138	0.011837298	0.024390013
6423	ENSG00000256064.1			1.167093796	0.011855062	0.02442027
6424	ENSG00000180919.3	OR56B4	196335	1.172188074	0.011883688	0.024470337
6425	ENSG00000204658.5	CST9LP2		1.084280983	0.011968399	0.024628143
6426	ENSG00000284490.1			1.175248767	0.011971515	0.024633276
6427	ENSG00000255191.1			1.001170334	0.011976129	0.024641492
6428	ENSG00000271723.5	MROH7-TTC4		1.18249535	0.011990555	0.02466769
6429	ENSG00000279785.1			1.131342994	0.012001978	0.024686989
6430	ENSG00000105507.3	CABP5	56344	1.528813575	0.012043889	0.024765489
6431	ENSG00000197403.4	OR6N1	128372	1.40260269	0.012084437	0.024839159
6432	ENSG00000254187.1			1.771898536	0.012103166	0.024865456
6433	ENSG00000205810.9	KLRC3	3823	1.070563672	0.012135336	0.024925089
6434	ENSG00000230248.1			1.289094609	0.012160419	0.024971435
6435	ENSG00000172689.2	MS4A10	341116	1.226895633	0.012277519	0.02518581
6436	ENSG00000279770.1	LINC00552	100130386	1.471380549	0.012345731	0.025308716
6437	ENSG00000244158.1	CALHM6-AS1		1.03061429	0.012382809	0.025375543
6438	ENSG00000237402.1	CAMTA1-IT1		1.199226031	0.012414442	0.025437738
6439	ENSG00000226355.2			1.360734461	0.012501805	0.025602193
6440	ENSG00000259265.1			1.263606054	0.012536442	0.025665173
6441	ENSG00000287563.1			1.218021104	0.012540264	0.025671672
6442	ENSG00000251165.6	F11-AS1	285441	1.453928324	0.012726433	0.026008471
6443	ENSG00000234177.5			1.433180128	0.012803953	0.026148025
6444	ENSG00000228325.5	IGKV3D-7		1.374816694	0.012804851	0.026148514
6445	ENSG00000287442.1			1.826934372	0.012811562	0.02616087
6446	ENSG00000203784.3	LELP1	149018	1.509882526	0.012827851	0.026192782
6447	ENSG00000251688.2	LINC02507		1.122241468	0.012832474	0.026199523
6448	ENSG00000188674.11	C2orf80	389073	1.2496788	0.012848335	0.026227856
6449	ENSG00000274397.1			1.211897614	0.012899666	0.026323154
6450	ENSG00000227743.2			2.200425307	0.012931954	0.026379536
6451	ENSG00000283400.1			2.015446094	0.012942595	0.026399884
6452	ENSG00000228466.1	TUBB4AP1		1.228543408	0.01296329	0.026438016
6453	ENSG00000270356.1	IGHV1OR15-4		1.669382481	0.013015405	0.026533384
6454	ENSG00000272395.7	IFNL4	101180976	1.40685635	0.013025878	0.02655337
6455	ENSG00000178171.11	AMER3	205147	1.281159848	0.013037005	0.026570588
6456	ENSG00000234208.1			1.083167241	0.013079444	0.026648864
6457	ENSG00000256963.1	PPDPFP2		1.452768564	0.013139348	0.02676129
6458	ENSG00000231093.1	TRMT1P1		1.484854249	0.013151326	0.026780183
6459	ENSG00000233421.4	LINC01783	100132147	1.147844977	0.013186953	0.026848594
6460	ENSG00000101074.5	R3HDML	140902	2.103198379	0.013240914	0.026950153
6461	ENSG00000234572.1	LINC01800	101927438	1.02054854	0.013266693	0.026997079
6462	ENSG00000253209.1	IGHV3-65		1.404169726	0.013355186	0.027150683
6463	ENSG00000237013.1	LINC01812		1.336541962	0.013476153	0.027371343
6464	ENSG00000260048.2	IGHV1OR16-3		1.296648676	0.013513829	0.027439433
6465	ENSG00000255374.3	TAS2R43	259289	1.098326933	0.013545313	0.027499136
6466	ENSG00000278239.1			1.370361126	0.013565408	0.027534293
6467	ENSG00000257966.1	OLA1P3		1.06371995	0.013578847	0.027560159
6468	ENSG00000286819.1			1.336208678	0.013584774	0.027566544
6469	ENSG00000253691.2	IGKV2OR22-4		1.074885453	0.013641109	0.027668116
6470	ENSG00000270926.1			1.22064484	0.01365286	0.027689117
6471	ENSG00000253376.2			1.800386116	0.013746789	0.027851121
6472	ENSG00000287952.1			1.148322947	0.013771457	0.027893971
6473	ENSG00000253957.1	IGHV3-22		1.179670519	0.013800469	0.027945598
6474	ENSG00000232234.4		105374910	1.116865914	0.013862743	0.028064535
6475	ENSG00000288638.1	LNCDAT		1.399536289	0.013885966	0.028107242
6476	ENSG00000248125.2			1.057140741	0.013915889	0.028163497
6477	ENSG00000287038.1			1.425324186	0.013947532	0.028223668
6478	ENSG00000259897.2	FRG2JP		1.396241582	0.013947755	0.028223668
6479	ENSG00000261599.6	HERC2P8		1.218418504	0.013958646	0.02823994
6480	ENSG00000217718.1	NDUFB4P4		1.76739645	0.013965108	0.02825077
6481	ENSG00000286099.1			2.270582449	0.013968781	0.028254679
6482	ENSG00000232780.2	GAPDHP74		1.083018117	0.01397276	0.028259844
6483	ENSG00000255538.1	OR10V2P	81343	1.016759161	0.014003958	0.028318608
6484	ENSG00000263765.5		105372058	1.334222157	0.014082278	0.028461018
6485	ENSG00000200816.1	SNORA38	677820	1.021852292	0.014105353	0.028503294
6486	ENSG00000211905.1	IGHJ1		1.126799165	0.014112139	0.028515553
6487	ENSG00000228020.2	HNRNPA1P46		1.121611394	0.014120562	0.028529666
6488	ENSG00000237379.1	CBX1P3		1.051886601	0.014124791	0.028536756
6489	ENSG00000218027.2	ELF2P2		1.032507956	0.014173137	0.028628596
6490	ENSG00000287713.1			1.075766228	0.01417808	0.028637065
6491	ENSG00000196600.12	SLC22A25	387601	1.078231704	0.014211933	0.028698189
6492	ENSG00000232886.1			1.280478135	0.014215697	0.028704327
6493	ENSG00000124610.5	H1-1	3024	1.047444502	0.014220548	0.028712659
6494	ENSG00000285909.1			1.0464653	0.014238283	0.028745542
6495	ENSG00000287832.1			1.560415276	0.014251392	0.028767612
6496	ENSG00000283234.1			1.078079406	0.014256046	0.028775542
6497	ENSG00000234464.2		105373220	1.211066514	0.014289142	0.028833537
6498	ENSG00000075388.4	FGF4	2249	1.740337763	0.014295621	0.028845143
6499	ENSG00000243953.1		105374171	1.586551007	0.014351878	0.0289454
6500	ENSG00000287000.1		124903585	1.284236527	0.014385683	0.029004726
6501	ENSG00000271803.1			1.179622651	0.014389699	0.029011347
6502	ENSG00000236389.1			1.25639173	0.014442282	0.029102564
6503	ENSG00000215398.11			2.185449224	0.014484209	0.029178153
6504	ENSG00000236140.1			1.035027634	0.014503407	0.029210891
6505	ENSG00000189398.5	OR7E12P		1.266735155	0.014563284	0.02931964
6506	ENSG00000257624.1			1.147959461	0.014617173	0.029414622
6507	ENSG00000250358.6	LINC02200		1.967615344	0.014618046	0.029414886
6508	ENSG00000211650.2	IGLV5-45		1.03449798	0.01462426	0.029425896
6509	ENSG00000261760.8			1.42656621	0.014633528	0.029441557
6510	ENSG00000248719.2	PRDM8-AS1		1.015273941	0.014749729	0.029652773
6511	ENSG00000267719.1	LINC02078	100652929	1.018090435	0.014761244	0.029674418
6512	ENSG00000284823.1			1.926393787	0.01481184	0.029762551
6513	ENSG00000264116.6			1.0984361	0.014867495	0.029855833
6514	ENSG00000206260.4	PRR23A	729627	1.664053065	0.014923525	0.029962672
6515	ENSG00000261764.1	KRT18P18		1.10613182	0.014931402	0.029975452
6516	ENSG00000260750.7			1.076446661	0.014949005	0.030004713
6517	ENSG00000255128.1	HSPD1P3		1.314745088	0.015018463	0.030130396
6518	ENSG00000239893.2	ZNF736P9Y		1.350531599	0.015066209	0.030213956
6519	ENSG00000231096.1	NDUFB4P3		1.529676404	0.015089944	0.030255434
6520	ENSG00000237955.1			1.021652651	0.01509548	0.030265003
6521	ENSG00000238076.1	MRPL48P1		1.240970289	0.015181737	0.030430246
6522	ENSG00000242079.1	RN7SL318P		1.422024936	0.01521691	0.030488416
6523	ENSG00000279020.1	C18orf15	147276	1.113569	0.015226148	0.030505384
6524	ENSG00000222000.7			1.158134299	0.015313886	0.03067157
6525	ENSG00000245870.4	LINC00682	101927074	1.705569725	0.015365193	0.030753315
6526	ENSG00000237862.2			1.382012754	0.015439056	0.030891356
6527	ENSG00000102239.5	BRS3	680	1.029028285	0.015454349	0.030910769
6528	ENSG00000236098.1		124905716	1.155459135	0.015506229	0.030999166
6529	ENSG00000236747.3	LINC01282	101927449	1.230121969	0.015536959	0.031055903
6530	ENSG00000261834.2	IGHV3OR16-15		1.141423394	0.015586198	0.031140196
6531	ENSG00000204965.9	PCDHA5	56143	1.141144727	0.015765588	0.031462141
6532	ENSG00000228334.2			1.03609324	0.015770191	0.031469743
6533	ENSG00000257846.2	SSBP3P5		1.124537136	0.015839471	0.031590503
6534	ENSG00000232746.1	LINC02022	105376955	2.117593695	0.015862855	0.031627593
6535	ENSG00000205022.9	PABPN1L	390748	1.303809219	0.016029681	0.031929706
6536	ENSG00000233534.3	LINC02524	105378013	1.212118325	0.016041475	0.031949989
6537	ENSG00000231241.1	RPS3AP3		1.085803364	0.01612268	0.032102052
6538	ENSG00000284654.1			1.014868426	0.016187895	0.032217341
6539	ENSG00000268056.5			1.011315351	0.016206305	0.032249123
6540	ENSG00000270748.1	IGKV2OR2-1		1.3059905	0.016247584	0.032321533
6541	ENSG00000234919.1	LINC01827	105373818	1.600612763	0.016250598	0.032325905
6542	ENSG00000286769.1			2.129855111	0.016310789	0.032434871
6543	ENSG00000236838.2			1.186847827	0.016338797	0.032480169
6544	ENSG00000240457.3	RN7SL472P		1.244376759	0.016348928	0.03249705
6545	ENSG00000280025.1			1.188990064	0.016388815	0.032564905
6546	ENSG00000253488.1	SINHCAFP3		1.099796401	0.016416468	0.03261168
6547	ENSG00000180745.5	CLRN3	119467	1.020770401	0.016515067	0.032776348
6548	ENSG00000175676.15	GOLGA8DP	100132979	1.314048796	0.01652058	0.032785648
6549	ENSG00000240427.1	RPS26P34		1.850167986	0.016521926	0.032786679
6550	ENSG00000260338.2	LINC01570		1.400537125	0.016531186	0.032801771
6551	ENSG00000261010.1	RARRES2P6		1.601598957	0.016603745	0.032930913
6552	ENSG00000266994.1			1.227319198	0.016658031	0.033030321
6553	ENSG00000159455.9	LCE2B	26239	1.537130289	0.016676583	0.033062146
6554	ENSG00000287834.1			1.244687693	0.016737213	0.033170736
6555	ENSG00000258860.1			1.024257864	0.016839201	0.033352857
6556	ENSG00000171561.4	OR2AT4	341152	1.152080003	0.016841285	0.03335389
6557	ENSG00000273044.1			1.223809707	0.016922147	0.033495393
6558	ENSG00000251215.1	GOLGA5P1		1.007905065	0.016960108	0.033565918
6559	ENSG00000232936.6	PANK1-AS1	105378421	1.082971356	0.017016341	0.033663352
6560	ENSG00000174990.8	CA5A	763	1.126314022	0.017034082	0.033691725
6561	ENSG00000211678.2	IGLJ3		1.224038218	0.017053938	0.033724268
6562	ENSG00000268987.1			1.161044632	0.01710439	0.033807176
6563	ENSG00000287343.1			1.292723764	0.017109819	0.033816221
6564	ENSG00000176988.9	FMR1NB	158521	1.478480981	0.017220954	0.034011111
6565	ENSG00000244400.2	RPS4XP12		1.405060997	0.017288892	0.034132713
6566	ENSG00000188162.12	OTOG	340990	1.14964362	0.0172943	0.034141588
6567	ENSG00000225647.2		105375448	1.109579051	0.017299808	0.034147463
6568	ENSG00000287673.1			1.220310862	0.01730725	0.034157051
6569	ENSG00000242512.9	LINC01206	100996490	1.003163255	0.017340756	0.034216364
6570	ENSG00000179994.11	SPDYE7P		1.114300102	0.017538508	0.034561843
6571	ENSG00000259848.9			1.49575407	0.017581584	0.034637788
6572	ENSG00000287490.1			1.142367721	0.017599784	0.034667089
6573	ENSG00000279996.1			1.016833786	0.017700343	0.034840933
6574	ENSG00000233186.2	KLF4P1		1.634211271	0.017721514	0.034880874
6575	ENSG00000276520.1			1.633289245	0.017725076	0.034886153
6576	ENSG00000239482.6		105374042	1.314009047	0.017732268	0.034898575
6577	ENSG00000285940.2		124904288	1.412018557	0.017768613	0.034957959
6578	ENSG00000286365.1			1.061513494	0.017799234	0.035010638
6579	ENSG00000271288.1	IGHV1OR15-3		1.352981622	0.017805373	0.035019313
6580	ENSG00000206181.6	ELOA2	51224	1.104920142	0.017813381	0.035032129
6581	ENSG00000268100.1	ZNF725P		1.508028747	0.017840753	0.03507552
6582	ENSG00000214919.3			1.260012707	0.017887832	0.035157619
6583	ENSG00000287741.1			1.266583914	0.017910822	0.03519757
6584	ENSG00000232900.2		101929413	1.260276561	0.017939162	0.035248022
6585	ENSG00000270187.1	IGKV1OR2-11		1.190775019	0.018003441	0.035363807
6586	ENSG00000277498.1			1.139388633	0.018108045	0.035542865
6587	ENSG00000236595.1	MED15P5		1.41586781	0.018404827	0.03607004
6588	ENSG00000287862.1			1.305609682	0.01845528	0.036154622
6589	ENSG00000241721.1	SUMO1P1		1.528793987	0.018478429	0.036196394
6590	ENSG00000255723.1			1.187021452	0.018486952	0.036211301
6591	ENSG00000257185.2	LINC02293		1.735422232	0.018498151	0.036229657
6592	ENSG00000251439.1	KRTAP4-17P		1.149107481	0.018526858	0.036280506
6593	ENSG00000282925.1			1.376083172	0.018562119	0.036340581
6594	ENSG00000206187.4	LINC02346	100128714	1.281744073	0.018611009	0.036418314
6595	ENSG00000228422.5	LINC00687		1.086777086	0.018654768	0.036490606
6596	ENSG00000196119.8	OR8A1	390275	1.792649996	0.018716226	0.036597113
6597	ENSG00000231426.7	FILNC1	100132735	1.278490372	0.018755005	0.03666769
6598	ENSG00000257611.1			1.032123995	0.018805238	0.036758474
6599	ENSG00000286572.1			1.042072701	0.018827955	0.036801065
6600	ENSG00000224679.1	MED15P4		1.726092784	0.018867567	0.036863952
6601	ENSG00000189252.4	SPANXN3	139067	1.961761847	0.018958137	0.037029962
6602	ENSG00000259000.1	DOCK11P1		1.203155793	0.018965986	0.037041642
6603	ENSG00000271426.1	CENATACP1		1.022218059	0.019072795	0.037226407
6604	ENSG00000286712.1			1.014457235	0.019084332	0.037245258
6605	ENSG00000101883.5	RHOXF1	158800	1.004984546	0.019107475	0.037279415
6606	ENSG00000279107.1			1.304162965	0.01911847	0.037299032
6607	ENSG00000225794.3			1.339331322	0.019131768	0.037320276
6608	ENSG00000172016.16	REG3A	5068	2.07351546	0.019191831	0.037401666
6609	ENSG00000286876.1			1.648295145	0.019216085	0.037439732
6610	ENSG00000242686.4	PDE6B-AS1		1.456506114	0.019244773	0.037486446
6611	ENSG00000224015.1			2.162214086	0.019321756	0.037619736
6612	ENSG00000267084.1			1.029179283	0.019469416	0.037875613
6613	ENSG00000272130.1			1.030260514	0.019547702	0.03801299
6614	ENSG00000253880.1			1.227826796	0.019579542	0.038065571
6615	ENSG00000261266.2			1.019087276	0.019605693	0.038112548
6616	ENSG00000171759.10	PAH	5053	1.051509446	0.019617438	0.038131767
6617	ENSG00000204363.5	SPANXN5	494197	1.546590655	0.019626621	0.038145876
6618	ENSG00000230866.2	LINC02558		1.118645853	0.019634651	0.038159613
6619	ENSG00000253291.1	TRBV7-7		1.102755544	0.019637372	0.038161517
6620	ENSG00000238575.1		124904380	1.05403891	0.019650159	0.038180396
6621	ENSG00000274621.1	MIR6867	102465523	1.014887054	0.019652555	0.038183179
6622	ENSG00000258108.2			1.421838558	0.019670177	0.038213673
6623	ENSG00000258159.1	IMMP1LP2		1.002468443	0.019730261	0.038309752
6624	ENSG00000267593.2	LINC01926	105372147	1.01913438	0.019898489	0.038621264
6625	ENSG00000250804.1	OR7E111FP		1.158692243	0.019998001	0.038795417
6626	ENSG00000064489.23	BORCS8-MEF2B		1.06028737	0.020001892	0.038800374
6627	ENSG00000214880.3	OR7E5P		2.002760296	0.02002508	0.038838448
6628	ENSG00000287893.1		124901616	1.335768496	0.020041073	0.038865664
6629	ENSG00000283069.1	DUX4L32		1.178027572	0.020063452	0.038899551
6630	ENSG00000189023.10	MAGEB16	139604	2.25596935	0.020095302	0.038946067
6631	ENSG00000260096.1	DNM1P33		1.103541409	0.020107127	0.038963271
6632	ENSG00000158497.5	HMHB1	57824	1.053672359	0.020324613	0.039334728
6633	ENSG00000267770.1			1.120677222	0.020348937	0.039370273
6634	ENSG00000260232.3	PWRN4		1.346737326	0.020372074	0.039409268
6635	ENSG00000264370.1	MIR3125	100422986	1.019433338	0.02040876	0.039468682
6636	ENSG00000180777.14	ANKRD30B	374860	1.219234881	0.020410686	0.039470482
6637	ENSG00000204542.3	C6orf15	29113	1.012492209	0.020420972	0.039482302
6638	ENSG00000223899.1	SEC13P1		1.004527251	0.020464508	0.0395572
6639	ENSG00000230668.2			1.843732348	0.020514492	0.039648018
6640	ENSG00000287339.1			1.178444683	0.020547309	0.039699833
6641	ENSG00000239279.3	RN7SL184P		1.004849283	0.020624757	0.039826181
6642	ENSG00000268889.1	LIM2-AS1		1.131524338	0.020681087	0.039918372
6643	ENSG00000247381.3	PLUT		1.413695923	0.020728542	0.039999316
6644	ENSG00000267476.1			1.080778097	0.02074272	0.040018884
6645	ENSG00000255041.1			1.457787944	0.020762752	0.04004413
6646	ENSG00000278095.1			1.023420033	0.020784115	0.040083144
6647	ENSG00000265992.1	ESRG	790952	1.228485195	0.020814102	0.040131217
6648	ENSG00000214946.14	TBC1D26	353149	1.23388844	0.020858141	0.040206352
6649	ENSG00000267489.5	LINC01837		1.391647583	0.020899684	0.040278597
6650	ENSG00000250993.1			2.177854502	0.020904838	0.040286571
6651	ENSG00000185203.12	WASIR1	100128260	1.456251627	0.020986439	0.040430072
6652	ENSG00000258794.3	DUX4L27		1.179341447	0.021006387	0.040458674
6653	ENSG00000267761.3	MIR4527HG	110175910	1.447112301	0.021148272	0.040704265
6654	ENSG00000227646.7	STEAP2-AS1	100874100	1.018530816	0.021154207	0.040711735
6655	ENSG00000161652.12	IZUMO2	126123	1.351425517	0.021245108	0.04087477
6656	ENSG00000280270.1			1.061586277	0.021253516	0.040888963
6657	ENSG00000280744.1	LINC01173		1.305689366	0.021264658	0.040908415
6658	ENSG00000253247.1	IGHV3-76		1.156761803	0.021311197	0.040984024
6659	ENSG00000223962.1	UBBP3		1.093597569	0.021315931	0.040991142
6660	ENSG00000223318.1	RNA5SP111		1.086603628	0.021575615	0.04144228
6661	ENSG00000286284.1			2.804791541	0.021594317	0.041476195
6662	ENSG00000286933.1			1.07580692	0.021793046	0.041807262
6663	ENSG00000225117.2	ARSDP1		1.189869236	0.021817958	0.041853027
6664	ENSG00000143032.8	BARHL2	343472	1.400621732	0.021924385	0.042038879
6665	ENSG00000260850.2		102723323	1.239830323	0.021952925	0.042085462
6666	ENSG00000236303.4	LINC02646	124900609	1.116622666	0.022119395	0.042377961
6667	ENSG00000287936.1			1.097153014	0.022140509	0.042414953
6668	ENSG00000118702.9	GHRH	2691	1.731972289	0.022192659	0.042506002
6669	ENSG00000181903.5	OR4C6	219432	1.566722526	0.022237826	0.04258017
6670	ENSG00000206120.11	EGFEM1P		1.035748665	0.022273278	0.042639815
6671	ENSG00000198128.4	OR2L3	391192	1.304552365	0.022320964	0.042708421
6672	ENSG00000258872.2	FDPSP3		1.00210856	0.022352568	0.042761239
6673	ENSG00000253569.1	VENTXP5		2.135685731	0.022428381	0.04288084
6674	ENSG00000283416.1	MIR1910	100302261	1.06349923	0.022655329	0.043256339
6675	ENSG00000287164.1		124900656	1.155589003	0.022753271	0.043420343
6676	ENSG00000228923.1			1.406024911	0.022768366	0.043440784
6677	ENSG00000220248.1	ZNF402P		1.033741171	0.023041955	0.043893075
6678	ENSG00000163424.9	TEX55	152405	1.407468138	0.023047861	0.043900107
6679	ENSG00000186090.11	HTR3D	200909	1.352578084	0.023056619	0.04391468
6680	ENSG00000235010.1	JAKMIP3-AS1		1.355488196	0.0231441	0.044068599
6681	ENSG00000255680.1			1.08278671	0.023187017	0.044146076
6682	ENSG00000287894.1			1.429014271	0.023299707	0.044347851
6683	ENSG00000170615.15	SLC26A5	375611	1.09402534	0.023321666	0.044376865
6684	ENSG00000272366.1			1.121542017	0.023401879	0.044506002
6685	ENSG00000285992.1			1.319575735	0.023404965	0.044507601
6686	ENSG00000271227.1	SREK1IP1P2		1.301245998	0.023498101	0.044667571
6687	ENSG00000259587.3			1.176260236	0.023536088	0.044726915
6688	ENSG00000235942.3	LCE6A	448835	1.390467088	0.023553423	0.044753422
6689	ENSG00000205293.5	LINC01602	100505477	1.234018962	0.023684996	0.044981865
6690	ENSG00000275811.1	HTR1DP1		1.000087138	0.023709647	0.045020056
6691	ENSG00000258316.1	KLF17P1		1.415773161	0.02380525	0.045182114
6692	ENSG00000260053.2	ABCB10P4		1.385156111	0.023853629	0.045258771
6693	ENSG00000218274.2			1.494997397	0.024015463	0.04551573
6694	ENSG00000268535.1	MTDHP4		1.30409287	0.02414887	0.045732154
6695	ENSG00000278911.1			1.78444326	0.024165618	0.045758757
6696	ENSG00000252797.1	RN7SKP272		1.002482235	0.024175904	0.045771675
6697	ENSG00000271901.2			1.568490661	0.024272953	0.045929097
6698	ENSG00000260838.3			1.272415127	0.024275872	0.045932429
6699	ENSG00000279154.1			1.496983785	0.024384786	0.046116393
6700	ENSG00000274508.1			1.104480216	0.024425294	0.046186498
6701	ENSG00000227497.1	PABPC1P6		1.931226026	0.024428003	0.046189417
6702	ENSG00000179817.5	MRGPRX4	117196	1.236011001	0.024445866	0.046218785
6703	ENSG00000237282.3	LINC00851	440757	1.623884839	0.024458004	0.046237325
6704	ENSG00000204347.4	BTBD17	388419	1.13757859	0.024528376	0.046354889
6705	ENSG00000237941.2	KCNQ1DN	55539	1.170536161	0.024554336	0.046392894
6706	ENSG00000252484.1	RN7SKP49		1.25072754	0.02461132	0.046480624
6707	ENSG00000255406.3	LINC02730	107984414	1.190924652	0.024646729	0.046540847
6708	ENSG00000264739.1			1.332013835	0.024653667	0.046551731
6709	ENSG00000288617.1			1.510577786	0.024658666	0.046558953
6710	ENSG00000260834.1			1.552995013	0.02477385	0.046758622
6711	ENSG00000260865.1			1.083246255	0.024884551	0.046929584
6712	ENSG00000181323.8	SPEM1	374768	1.184094328	0.02493159	0.047004879
6713	ENSG00000287332.1			1.225218277	0.024969812	0.047061277
6714	ENSG00000111783.13	RFX4	5992	1.227899	0.024972155	0.047063455
6715	ENSG00000226430.7	USP17L7	392197	1.459755381	0.025013411	0.047132143
6716	ENSG00000206159.11	GYG2P1		1.204207606	0.025158541	0.047384792
6717	ENSG00000286671.1			1.114961167	0.025199739	0.047451769
6718	ENSG00000235824.5	LINC00837	100507605	1.468701675	0.025286953	0.047579841
6719	ENSG00000211633.3	IGKV1D-42		1.022269369	0.025405412	0.04777326
6720	ENSG00000231421.8			2.473899152	0.025503015	0.047931791
6721	ENSG00000259761.2			1.283827251	0.025517601	0.047952384
6722	ENSG00000182271.13	TMIGD1	388364	1.209829914	0.02557321	0.048040946
6723	ENSG00000233014.1	TBC1D3P7		1.112152162	0.025577292	0.048044061
6724	ENSG00000234601.1	CHRM3-AS1	100873984	1.082489891	0.025639039	0.048148413
6725	ENSG00000267563.1			1.404357026	0.025935657	0.048645767
6726	ENSG00000269466.4	H3Y1	391769	1.707312108	0.025989004	0.048727385
6727	ENSG00000211974.3	IGHV2-70D		1.013909674	0.025998204	0.04874233
6728	ENSG00000187223.4	LCE2D	353141	1.286954779	0.026047159	0.048827248
6729	ENSG00000207568.1	MIR203A	406986	1.092822911	0.026192012	0.049073202
6730	ENSG00000227406.1	RNF6P1		1.36659506	0.026341272	0.049317897
6731	ENSG00000242925.1	PLCH1-AS2		1.403338368	0.02640322	0.049424545
6732	ENSG00000231358.4			2.220548684	0.026486067	0.049563247
6733	ENSG00000231031.6	LINC01804	101926966	1.465500097	0.026597957	0.049756188
6734	ENSG00000282381.1			1.325996013	0.026637646	0.049823381
6735	ENSG00000222398.1	RNU6-554P		1.073500673	0.026646268	0.049833507
6736	ENSG00000225671.2	FCF1P6		1.516867636	0.026746677	0.049994296
6737	ENSG00000224593.1	RPL21P72		1.080565123	0.026754113	0.050005837
6738	ENSG00000266155.2			1.024386911	0.026790979	0.050063629
6739	ENSG00000286983.1			1.667915013	0.026796541	0.050070977
6740	ENSG00000224712.12	NPIPA3	642778	1.105648013	0.026883224	0.050211652
6741	ENSG00000256299.1			1.011929105	0.026891388	0.050224535
6742	ENSG00000255109.6	LINC02740		1.439652951	0.026916776	0.050260114
6743	ENSG00000259572.7	LINC01491		1.613009331	0.026921629	0.050266807
6744	ENSG00000187021.15	PNLIPRP1	5407	1.292381938	0.026980393	0.0503623
6745	ENSG00000229459.1			1.099277592	0.027065203	0.050496834
6746	ENSG00000254157.1	IGKV2-18		1.16520978	0.027127067	0.050597972
6747	ENSG00000249383.2	LINC02071	101927274	1.102652373	0.027235612	0.050769383
6748	ENSG00000253502.1	ATP6V1G1P2		1.208768174	0.027342287	0.050929923
6749	ENSG00000287550.1			1.136768041	0.027372322	0.050978684
6750	ENSG00000226375.1			1.018279556	0.027596504	0.051347965
6751	ENSG00000228259.3			1.122492716	0.027653237	0.051435136
6752	ENSG00000204479.4	PRAMEF17	391004	1.509096522	0.027770467	0.051627855
6753	ENSG00000167768.4	KRT1	3848	1.010960293	0.02781496	0.051693792
6754	ENSG00000287347.1			1.08558604	0.027816761	0.051694716
6755	ENSG00000230676.1			1.627730174	0.02782522	0.051705588
6756	ENSG00000182613.3	OR2V2	285659	1.449195357	0.027838476	0.051725373
6757	ENSG00000226057.7	PHF2P2		1.340247999	0.027933991	0.051880963
6758	ENSG00000232873.1	RPL23AP93		1.032535932	0.027986548	0.051966404
6759	ENSG00000237760.1			1.222321986	0.028015065	0.052012048
6760	ENSG00000162460.7	TMEM82	388595	1.265879026	0.02803173	0.052038115
6761	ENSG00000244217.1	RPS4XP10		1.079042602	0.028044133	0.052058703
6762	ENSG00000270938.1	RAP2CP1		1.123300072	0.028097543	0.052143201
6763	ENSG00000224682.1	SOCS5P2		1.142684912	0.028114976	0.052173111
6764	ENSG00000227269.1		105376174	1.768002208	0.028176977	0.052271042
6765	ENSG00000287905.1			1.471162767	0.028273486	0.052432903
6766	ENSG00000287746.1			1.430543259	0.028334815	0.052519632
6767	ENSG00000204993.3	CRTC1P1		1.386932879	0.028382942	0.052601444
6768	ENSG00000273325.1			1.151740997	0.028510049	0.052801943
6769	ENSG00000287008.1			1.55682512	0.028791442	0.053248991
6770	ENSG00000187180.3	LCE2C	353140	1.253020015	0.028836802	0.053310502
6771	ENSG00000235335.3	B3GALT1-AS1		1.222325252	0.028871788	0.053370204
6772	ENSG00000270930.1	GRAMD4P8		1.242285271	0.028959802	0.053517931
6773	ENSG00000230404.1			1.247939468	0.029092135	0.053737435
6774	ENSG00000227016.3	LINC02796		1.074472384	0.029219919	0.053948339
6775	ENSG00000225385.3			1.050516864	0.029295612	0.054067947
6776	ENSG00000237751.2	LINC01143	104355141	1.404977797	0.029356745	0.054158087
6777	ENSG00000234274.1	COX7BP2		1.124643501	0.029405397	0.054232554
6778	ENSG00000165471.7	MBL2	4153	1.15888815	0.029449252	0.054298428
6779	ENSG00000227289.2	HSFY3P		1.089106495	0.029571148	0.054500749
6780	ENSG00000286146.1			1.534378147	0.029767602	0.054834401
6781	ENSG00000227463.3		101927575	1.130621745	0.029880671	0.055014567
6782	ENSG00000287847.1			1.107349058	0.030107984	0.05536367
6783	ENSG00000237339.5	LINC01502	100130954	1.225857838	0.030216626	0.055540179
6784	ENSG00000213886.4	UBD	10537	1.068776408	0.030552671	0.056103318
6785	ENSG00000254746.6		105376654	1.056878379	0.030659016	0.056277747
6786	ENSG00000272396.1			1.307336092	0.030675193	0.05630402
6787	ENSG00000236380.5	VENTXP7		1.643004087	0.030856274	0.056587438
6788	ENSG00000022355.18	GABRA1	2554	1.384681967	0.03103805	0.056878704
6789	ENSG00000254046.1	IGHV1-17		1.004961647	0.031120192	0.057002886
6790	ENSG00000263567.1			1.548668061	0.031234596	0.057188664
6791	ENSG00000145194.19	ECE2	9718	1.553327	0.031484744	0.05760145
6792	ENSG00000232208.2			1.062796251	0.03162897	0.057830621
6793	ENSG00000260343.1	LINC01043	101928752	1.563284125	0.031638825	0.057845973
6794	ENSG00000258803.1		101927690	1.144805336	0.031658945	0.057874752
6795	ENSG00000272311.1	IFNL4P1		1.562271661	0.031845741	0.058170634
6796	ENSG00000238166.2			1.024686101	0.031991898	0.058394567
6797	ENSG00000233109.4			1.403925915	0.032172461	0.058686322
6798	ENSG00000229613.2	LINC01501	340515	1.2185166	0.032243839	0.058802997
6799	ENSG00000248363.5			1.763320683	0.03235607	0.058980544
6800	ENSG00000270492.1	MARK2P3		1.017337334	0.032377947	0.059014997
6801	ENSG00000124449.7	IRGC	56269	1.010144295	0.032458825	0.059146099
6802	ENSG00000269446.2			1.313201083	0.032466979	0.059155519
6803	ENSG00000285609.1	5S_rRNA		2.334169462	0.032692325	0.059505945
6804	ENSG00000287469.1			1.048957556	0.032785872	0.059654308
6805	ENSG00000234647.2			1.005060056	0.032909034	0.059850938
6806	ENSG00000273644.1			1.042150933	0.032942729	0.059892987
6807	ENSG00000284839.1			1.270557707	0.032991085	0.059969345
6808	ENSG00000235659.1		102723678	1.102434123	0.033061244	0.060072649
6809	ENSG00000250043.1			1.463488843	0.03315555	0.060216409
6810	ENSG00000134640.3	MTNR1B	4544	1.161059738	0.033204112	0.060288035
6811	ENSG00000169551.13	CT55	54967	1.413907471	0.033215867	0.060306618
6812	ENSG00000171557.17	FGG	2266	1.138144416	0.03340543	0.060617477
6813	ENSG00000257943.2			1.121947254	0.033473089	0.060712466
6814	ENSG00000234259.3			1.085672875	0.033493477	0.060741109
6815	ENSG00000287705.1			1.54608323	0.033822125	0.061258665
6816	ENSG00000269350.1			1.133819588	0.034055442	0.06164775
6817	ENSG00000177138.17	FAM9B	171483	1.245949459	0.034056417	0.06164775
6818	ENSG00000224294.1	PINCR	101927501	1.362459428	0.034170552	0.061833245
6819	ENSG00000168619.16	ADAM18	8749	1.498282406	0.034240449	0.061948418
6820	ENSG00000287429.1			1.386061126	0.03426439	0.061980419
6821	ENSG00000186977.3	KRTAP19-5	337972	2.048421487	0.034431256	0.062248427
6822	ENSG00000272915.1			1.000241186	0.034610772	0.062524262
6823	ENSG00000259108.3	LINC01595		1.586087056	0.034746716	0.062744116
6824	ENSG00000246350.1	ZBED9-AS1	121676925	1.143709654	0.03480626	0.062837327
6825	ENSG00000158639.13	PAGE5	90737	1.019900398	0.035116982	0.063306048
6826	ENSG00000229859.10	PGA3	643834	1.126670483	0.035326492	0.063617208
6827	ENSG00000230314.7	ELOVL2-AS1	100506409	1.008269741	0.035520716	0.063917621
6828	ENSG00000260922.2			1.050601053	0.03552498	0.063919492
6829	ENSG00000266537.1	SPDYE22P		1.161646433	0.035546361	0.063949257
6830	ENSG00000150676.13	CCDC83	220047	1.119872975	0.035557695	0.063966745
6831	ENSG00000255882.1			1.005422103	0.035585123	0.064013182
6832	ENSG00000253171.1			1.218888559	0.035722048	0.064224527
6833	ENSG00000227432.1	ASIC4-AS1		1.112818978	0.035761181	0.064289053
6834	ENSG00000231724.1			1.276299499	0.035911807	0.06453058
6835	ENSG00000176302.13	FOXR1	283150	1.054975216	0.036208147	0.065007101
6836	ENSG00000287215.1		105377209	1.673452509	0.036244226	0.065054201
6837	ENSG00000223389.1	SDCBP2P1		1.363319254	0.036281809	0.065115333
6838	ENSG00000225349.2	ANKRD49P2		1.145724741	0.03651423	0.065476582
6839	ENSG00000178804.7	H1-8	132243	1.625236137	0.036721389	0.065794494
6840	ENSG00000153266.13	FEZF2	55079	2.297689488	0.037012705	0.066220691
6841	ENSG00000286631.1			1.121501374	0.037445196	0.066903906
6842	ENSG00000283303.1			2.215984338	0.037466478	0.066935898
6843	ENSG00000270747.1	S100A11P9		1.51146808	0.037557277	0.06707998
6844	ENSG00000212712.2			1.153976251	0.037628333	0.067179656
6845	ENSG00000255866.2			1.483668923	0.037638031	0.067193944
6846	ENSG00000279096.2			1.171715869	0.037745175	0.067367024
6847	ENSG00000171804.11	WDR87	83889	1.088092177	0.038018416	0.067802814
6848	ENSG00000277297.1	ATP5F1AP10		1.071863538	0.038175952	0.068050097
6849	ENSG00000261752.1	AGGF1P8		1.480682576	0.038384678	0.068372977
6850	ENSG00000270002.1	GRID1-AS2		1.101088235	0.038573022	0.068674529
6851	ENSG00000282718.1			1.187067023	0.038669842	0.068822182
6852	ENSG00000231933.7	MYO18B-AS1		1.09629975	0.038714083	0.068894733
6853	ENSG00000238077.1	NMNAT1P3		1.338234578	0.038836246	0.069081127
6854	ENSG00000232125.5	DYTN	391475	1.106949642	0.038950789	0.069266229
6855	ENSG00000207955.5	MIR219A2HG	123464509	1.025524647	0.039147574	0.069558627
6856	ENSG00000286212.2			1.235447572	0.039265769	0.069738778
6857	ENSG00000287719.1			1.495912011	0.03947798	0.07007801
6858	ENSG00000235415.1			1.145819833	0.039529521	0.070156937
6859	ENSG00000223786.1		101928516	1.359675109	0.039632661	0.070321104
6860	ENSG00000214283.4	RAD51AP1P1		1.192458293	0.039731799	0.070478082
6861	ENSG00000284617.1			1.33167182	0.039762406	0.070516601
6862	ENSG00000205682.2			1.071179415	0.040008786	0.070886958
6863	ENSG00000250753.2			2.053664726	0.040016241	0.070896999
6864	ENSG00000225680.1			1.381412127	0.040124524	0.071059811
6865	ENSG00000136275.10	PKD1L1-AS1	80099	1.062958383	0.040136056	0.071074341
6866	ENSG00000248327.1	NOL8P1		1.103649229	0.040445487	0.071564758
6867	ENSG00000270960.1			1.518979622	0.040740637	0.072019506
6868	ENSG00000253734.2	LINC01289	286184	1.142337304	0.041002059	0.072429968
6869	ENSG00000186143.11	PRR30	339779	1.544391694	0.041085142	0.072554106
6870	ENSG00000153779.11	TGIF2LX	90316	2.742168514	0.041278039	0.072865544
6871	ENSG00000269543.5			1.600695369	0.04137379	0.073008562
6872	ENSG00000278301.1	GRAMD4P3		1.576595676	0.041536912	0.073244253
6873	ENSG00000253410.1			1.133357572	0.041610679	0.073348234
6874	ENSG00000286551.1			1.065654153	0.041641334	0.073389219
6875	ENSG00000223611.6	SUPT20HL2	170067	1.082946798	0.04166505	0.073417962
6876	ENSG00000253283.1			1.051733611	0.041691193	0.073454236
6877	ENSG00000206026.7	SMIM21	284274	1.083044	0.041797513	0.073612117
6878	ENSG00000201920.1	RNA5SP442		1.165007762	0.042149055	0.07414232
6879	ENSG00000133124.11	IRS4	8471	1.155058799	0.042459063	0.074628043
6880	ENSG00000237210.1			1.186112638	0.042469179	0.074642514
6881	ENSG00000178235.8	SLITRK1	114798	1.298725579	0.042609787	0.07485646
6882	ENSG00000287633.1			1.185000197	0.042617815	0.074867247
6883	ENSG00000228734.2			1.102438133	0.042700548	0.074989326
6884	ENSG00000138813.10	C4orf17	84103	1.081984932	0.042836184	0.075187563
6885	ENSG00000124467.19	PSG8	440533	1.101066084	0.042846345	0.075202069
6886	ENSG00000261070.1		338694	1.328460711	0.043007486	0.075461513
6887	ENSG00000236893.1	ASS1P7		1.081260264	0.043019133	0.075475269
6888	ENSG00000279565.1			1.164076885	0.043166974	0.075701153
6889	ENSG00000251389.1	YTHDF1P1		1.160946087	0.043225668	0.075794025
6890	ENSG00000259111.1			1.288365788	0.043454335	0.076157933
6891	ENSG00000225236.1			1.295472854	0.043551451	0.076311273
6892	ENSG00000241640.2	RPL21P113		1.0539388	0.043996566	0.076985738
6893	ENSG00000257275.6			1.110857774	0.044026474	0.077031273
6894	ENSG00000286764.1			1.107685552	0.044151182	0.077208583
6895	ENSG00000261041.1			1.255401571	0.04443714	0.077629898
6896	ENSG00000258779.8	LOHAN2	100506172	1.04978967	0.04484989	0.078261302
6897	ENSG00000284668.2	LINC02780		2.005107435	0.044991347	0.078480506
6898	ENSG00000286928.1			1.160750531	0.045082074	0.078621472
6899	ENSG00000248844.6	LINC02753	101928443	1.324779009	0.045131658	0.078694099
6900	ENSG00000148215.4	OR5C1	392391	1.449101964	0.045193962	0.078788876
6901	ENSG00000178928.8	TPRX1	284355	1.658121749	0.045349652	0.079032497
6902	ENSG00000276400.1	VN1R9P		1.375625945	0.045477745	0.079220909
6903	ENSG00000230246.7	SPATA31C1	441452	1.632938694	0.045584842	0.079376083
6904	ENSG00000281548.1	LINC00895		1.131324515	0.045678034	0.079517402
6905	ENSG00000237557.1	YWHABP1		1.235369965	0.045964171	0.079952295
6906	ENSG00000265980.1			1.692662748	0.046065949	0.080101254
6907	ENSG00000264699.2	LINC01912		1.083345376	0.046175171	0.080255309
6908	ENSG00000287841.1			1.746632734	0.046390295	0.080598137
6909	ENSG00000285894.2			2.069206922	0.046468712	0.080716348
6910	ENSG00000266961.1			1.117151749	0.046626063	0.080961583
6911	ENSG00000197123.9	ZNF679	168417	1.643554223	0.046782188	0.081193565
6912	ENSG00000234626.2			1.390282543	0.046806402	0.081232034
6913	ENSG00000239921.6	LINC01471	101927149	1.137128278	0.047436931	0.082239917
6914	ENSG00000230991.2			1.549553316	0.047543874	0.082389294
6915	ENSG00000228100.2	LINC01820	105374582	1.590672113	0.047582875	0.082449672
6916	ENSG00000287179.1			1.315915104	0.047741865	0.08267458
6917	ENSG00000287176.1			1.45837143	0.04809476	0.083213001
6918	ENSG00000229649.1	LINC02681	101927419	1.435756192	0.048144372	0.083280874
6919	ENSG00000188585.9	CLEC20A	400797	1.179888761	0.04822766	0.083407851
6920	ENSG00000279043.1			1.192290184	0.048248971	0.083432485
6921	ENSG00000279397.1			1.141778648	0.048252413	0.083432679
6922	ENSG00000271275.1			1.176889737	0.048323699	0.083525278
6923	ENSG00000286635.1			1.285965805	0.048382786	0.083612827
6924	ENSG00000253311.3	LINC01847	101927766	1.057431687	0.048418594	0.083663768
6925	ENSG00000258550.1	OR7E106P		1.13624392	0.048668814	0.084037532
6926	ENSG00000271766.1			1.056083169	0.048872032	0.084344019
6927	ENSG00000270390.1	SFXN4P1		1.043831336	0.049204082	0.084832484
6928	ENSG00000224310.1	LINC01567	400511	1.10755374	0.049457491	0.085224915
6929	ENSG00000250855.1			1.406278044	0.049597986	0.085437309
6930	ENSG00000258320.1			1.209932564	0.049619909	0.085467648
6931	ENSG00000226278.1	PSPHP1		1.360718902	0.049668097	0.085535785
6932	ENSG00000286911.1			1.037988832	0.04983267	0.08578567

**Table 4:** Raw data of Down-regulated DEGs

	ENSEMBL	GENENAME	entrezgene_id	log2FoldChange	p-value	padj
1	ENSG00000236780.7	LINC01829	644838	-4.687076089	3.15E-97	1.25E-92
2	ENSG00000070081.17	NUCB2	4925	-2.151594889	3.16E-86	6.27E-82
3	ENSG00000106351.13	AGFG2	3268	-2.36880628	7.60E-86	7.53E-82
4	ENSG00000244040.7	IL12A-AS1	101928376	-4.125123063	1.33E-82	1.06E-78
5	ENSG00000102547.19	CAB39L	81617	-2.211503108	5.52E-82	3.65E-78
6	ENSG00000151882.11	CCL28	56477	-4.180018322	1.36E-79	7.72E-76
7	ENSG00000163072.16	NOSTRIN	115677	-2.49002706	8.36E-79	4.09E-75
8	ENSG00000152642.11	GPD1L	23171	-2.750437316	3.83E-76	1.52E-72
9	ENSG00000134531.10	EMP1	2012	-3.192275656	4.94E-75	1.78E-71
10	ENSG00000227028.6	SLC8A1-AS1		-4.777969597	1.14E-73	3.76E-70
11	ENSG00000163815.6	CLEC3B	7123	-3.592230748	2.44E-73	7.45E-70
12	ENSG00000101441.4	CST4	1472	-8.163819807	3.86E-73	1.02E-69
13	ENSG00000005381.8	MPO	4353	-4.62778501	1.76E-69	4.35E-66
14	ENSG00000187642.9	PERM1	84808	-4.834205994	3.07E-69	6.75E-66
15	ENSG00000167588.13	GPD1	2819	-5.193447452	1.08E-68	2.26E-65
16	ENSG00000166819.12	PLIN1	5346	-5.532939143	1.64E-68	3.25E-65
17	ENSG00000171503.12	ETFDH	2110	-1.581317573	2.06E-67	3.72E-64
18	ENSG00000131050.11	BPIFA2	140683	-11.52651463	3.12E-67	5.38E-64
19	ENSG00000126550.10	HTN1	3346	-11.44213502	4.05E-65	6.70E-62
20	ENSG00000280916.2	FOXCUT		-4.259635461	1.03E-63	1.57E-60
21	ENSG00000126861.5	OMG	4974	-3.843558776	6.29E-63	8.91E-60
22	ENSG00000151729.11	SLC25A4	291	-3.254704022	7.86E-63	1.07E-59
23	ENSG00000122042.10	UBL3	5412	-1.653729931	1.74E-62	2.15E-59
24	ENSG00000158571.11	PFKFB1	5207	-2.989597364	2.00E-62	2.40E-59
25	ENSG00000215187.12	CIMIP2B	730112	-3.458953966	4.90E-61	5.40E-58
26	ENSG00000198478.8	SH3BGRL2	83699	-3.743297288	7.55E-61	8.09E-58
27	ENSG00000162078.11	ZG16B	124220	-7.445124296	1.73E-60	1.81E-57
28	ENSG00000165795.23	NDRG2	57447	-2.928166113	5.14E-60	5.23E-57
29	ENSG00000184908.18	CLCNKB	1188	-5.589229438	3.45E-58	3.11E-55
30	ENSG00000280339.1			-3.212798855	5.67E-58	4.98E-55
31	ENSG00000172340.15	SUCLG2	8801	-1.205415781	8.39E-58	7.00E-55
32	ENSG00000163491.16	NEK10	152110	-3.566450786	8.47E-58	7.00E-55
33	ENSG00000167676.4	PLIN4	729359	-4.503696458	7.92E-57	6.15E-54
34	ENSG00000167419.11	LPO	4025	-8.009636717	2.29E-56	1.71E-53
35	ENSG00000158458.21	NRG2	9542	-3.854637545	7.51E-56	5.51E-53
36	ENSG00000169885.10	CALML6	163688	-5.192412259	3.59E-55	2.45E-52
37	ENSG00000134551.13	PRH2	5555	-7.976673013	7.30E-55	4.83E-52
38	ENSG00000151365.2	THRSP	7069	-6.365279467	9.83E-55	6.29E-52
39	ENSG00000186952.15	TMEM232	642987	-3.203197033	1.58E-54	9.64E-52
40	ENSG00000060762.19	MPC1	51660	-1.577531442	2.02E-54	1.21E-51
41	ENSG00000160097.20	FNDC5	252995	-4.883864607	2.08E-54	1.23E-51
42	ENSG00000185267.10	CDNF	441549	-2.469022375	3.30E-54	1.92E-51
43	ENSG00000214097.5	SMCO1	255798	-5.541853822	2.28E-53	1.17E-50
44	ENSG00000187288.12	CIDEC	63924	-5.592587492	1.57E-52	7.58E-50
45	ENSG00000156049.7	GNA14	9630	-2.812581228	1.69E-52	8.10E-50
46	ENSG00000173947.14	CIMAP3	128344	-3.987425597	4.14E-52	1.96E-49
47	ENSG00000168309.18	FAM107A	11170	-3.560572559	7.14E-52	3.33E-49
48	ENSG00000197168.13	NEK5	341676	-2.333246347	2.54E-51	1.13E-48
49	ENSG00000108381.11	ASPA	443	-3.831926073	4.74E-51	2.00E-48
50	ENSG00000117054.14	ACADM	34	-1.559679486	4.75E-51	2.00E-48
51	ENSG00000177981.11	ASB8	140461	-1.162767476	6.66E-51	2.76E-48
52	ENSG00000126549.11	STATH	6779	-9.878894652	8.78E-51	3.59E-48
53	ENSG00000187533.14	PRR27	401137	-10.40824778	9.55E-51	3.86E-48
54	ENSG00000181856.15	SLC2A4	6517	-4.375263311	1.09E-50	4.34E-48
55	ENSG00000152556.17	PFKM	5213	-2.113065846	2.06E-50	7.93E-48
56	ENSG00000223561.7	LINC03007	646588	-5.45197994	2.09E-50	7.97E-48
57	ENSG00000186628.12	FSD2	123722	-5.17155454	4.60E-50	1.68E-47
58	ENSG00000121671.12	CRY2	1408	-1.341532091	5.17E-50	1.83E-47
59	ENSG00000135469.14	COQ10A	93058	-2.082001978	7.85E-50	2.76E-47
60	ENSG00000131686.15	CA6	765	-6.072051499	8.64E-50	2.98E-47
61	ENSG00000008441.18	NFIX	4784	-1.885992234	1.71E-49	5.74E-47
62	ENSG00000112425.16	EPM2A	7957	-1.910498376	2.00E-49	6.62E-47
63	ENSG00000170367.5	CST5	1473	-9.313635588	2.33E-49	7.63E-47
64	ENSG00000149260.18	CAPN5	726	-2.662774561	2.47E-49	8.02E-47
65	ENSG00000165269.13	AQP7	364	-4.167883073	2.66E-49	8.58E-47
66	ENSG00000154358.22	OBSCN	84033	-3.361559352	3.13E-49	9.93E-47
67	ENSG00000170509.12	HSD17B13	345275	-3.805899421	3.51E-49	1.10E-46
68	ENSG00000168291.13	PDHB	5162	-1.031032009	5.50E-49	1.72E-46
69	ENSG00000161281.11	COX7A1	1346	-3.633752526	4.17E-48	1.22E-45
70	ENSG00000117528.14	ABCD3	5825	-1.103048694	4.65E-48	1.33E-45
71	ENSG00000265158.1	LRRC37A7P		-4.440928179	5.79E-48	1.64E-45
72	ENSG00000187699.10	C2orf88	84281	-2.639479568	1.07E-47	2.95E-45
73	ENSG00000230825.2	LINC03016	101927354	-3.728505471	1.14E-47	3.11E-45
74	ENSG00000150672.17	DLG2	1740	-3.09043633	1.35E-47	3.65E-45
75	ENSG00000107537.14	PHYH	5264	-2.276973645	1.40E-47	3.76E-45
76	ENSG00000169764.16	UGP2	7360	-1.151459024	2.42E-47	6.44E-45
77	ENSG00000250073.3	ESAM-AS1		-2.33002887	2.76E-47	7.30E-45
78	ENSG00000107864.15	CPEB3	22849	-1.875420529	3.20E-47	8.39E-45
79	ENSG00000204136.11	GGTA1	2681	-2.767785918	4.18E-47	1.08E-44
80	ENSG00000153446.16	C16orf89	146556	-4.799533671	4.54E-47	1.17E-44
81	ENSG00000112699.11	GMDS	2762	-1.626828036	7.51E-47	1.91E-44
82	ENSG00000239388.9	ASB14	142686	-2.654305679	8.51E-47	2.15E-44
83	ENSG00000168306.13	ACOX2	8309	-2.545060613	1.44E-46	3.57E-44
84	ENSG00000124374.9	PAIP2B	400961	-2.067249202	2.60E-46	6.31E-44
85	ENSG00000178821.13	TMEM52	339456	-3.398912849	3.01E-46	7.24E-44
86	ENSG00000134324.12	LPIN1	23175	-1.785122153	6.19E-46	1.46E-43
87	ENSG00000094796.5	KRT31	3881	-3.64402138	6.46E-46	1.51E-43
88	ENSG00000138029.14	HADHB	3032	-1.040033878	8.26E-46	1.93E-43
89	ENSG00000138379.5	MSTN	2660	-4.417423656	8.77E-46	2.03E-43
90	ENSG00000112981.5	NME5	8382	-4.157862677	1.29E-45	2.96E-43
91	ENSG00000259291.2	ZNF710-AS1	109729181	-2.416038105	2.95E-45	6.57E-43
92	ENSG00000197870.12	PRB3	5544	-6.858719167	3.51E-45	7.68E-43
93	ENSG00000169282.18	KCNAB1	7881	-1.81047136	3.76E-45	8.19E-43
94	ENSG00000103150.7	MLYCD	23417	-1.239907581	4.18E-45	9.06E-43
95	ENSG00000114166.8	KAT2B	8850	-2.033245657	4.73E-45	1.01E-42
96	ENSG00000106258.15	CYP3A5	1577	-2.359300866	4.89E-45	1.04E-42
97	ENSG00000130957.5	FBP2	8789	-5.45123248	5.45E-45	1.16E-42
98	ENSG00000103994.18	ZNF106	64397	-2.144328196	6.12E-45	1.29E-42
99	ENSG00000198894.8	CIPC	85457	-1.432152021	7.40E-45	1.54E-42
100	ENSG00000162688.17	AGL	178	-1.6896943	1.11E-44	2.27E-42
101	ENSG00000182606.17	TRAK1	22906	-1.173704099	1.21E-44	2.47E-42
102	ENSG00000130988.13	RGN	9104	-3.106919909	1.64E-44	3.29E-42
103	ENSG00000163576.18	EFHB	151651	-3.545719049	1.68E-44	3.34E-42
104	ENSG00000250878.3	METTL21EP	121952	-5.63805148	1.76E-44	3.49E-42
105	ENSG00000115840.14	SLC25A12	8604	-1.075476195	3.97E-44	7.71E-42
106	ENSG00000120053.12	GOT1	2805	-1.385424329	6.64E-44	1.28E-41
107	ENSG00000163820.15	FYCO1	79443	-1.919223746	9.85E-44	1.87E-41
108	ENSG00000157103.12	SLC6A1	6529	-3.282745765	1.50E-43	2.83E-41
109	ENSG00000129749.3	CHRNA10	57053	-2.509891815	2.40E-43	4.38E-41
110	ENSG00000205362.11	MT1A	4489	-4.174091396	2.49E-43	4.52E-41
111	ENSG00000206559.8	ZCWPW2	152098	-1.895447122	2.59E-43	4.68E-41
112	ENSG00000145916.19	RMND5B	64777	-1.388434291	3.03E-43	5.44E-41
113	ENSG00000023228.14	NDUFS1	4719	-1.15225983	3.22E-43	5.75E-41
114	ENSG00000185271.9	KLHL33	123103	-4.670758499	8.14E-43	1.40E-40
115	ENSG00000108515.18	ENO3	2027	-4.959983726	8.58E-43	1.47E-40
116	ENSG00000185664.14	PMEL	6490	-2.689733883	1.60E-42	2.70E-40
117	ENSG00000143149.12	ALDH9A1	223	-1.34580577	1.66E-42	2.79E-40
118	ENSG00000161558.11	TMEM143	55260	-1.710990552	1.69E-42	2.82E-40
119	ENSG00000161849.3	KRT84	3890	-6.255678536	1.98E-42	3.29E-40
120	ENSG00000205649.9	HTN3	3347	-10.96988195	2.19E-42	3.61E-40
121	ENSG00000140986.8	RPL3L	6123	-4.099613175	3.63E-42	5.90E-40
122	ENSG00000151276.23	MAGI1	9223	-1.578438642	4.35E-42	6.95E-40
123	ENSG00000228789.8	HCG22	285834	-5.047125734	9.10E-42	1.43E-39
124	ENSG00000164434.12	FABP7	2173	-5.371817471	9.39E-42	1.47E-39
125	ENSG00000025039.15	RRAGD	58528	-2.482193217	9.97E-42	1.55E-39
126	ENSG00000133943.21	DGLUCY	80017	-1.377470767	1.03E-41	1.59E-39
127	ENSG00000143409.15	MINDY1	55793	-1.513911376	1.54E-41	2.36E-39
128	ENSG00000111215.12	PRR4	5554	-4.468370019	2.64E-41	4.00E-39
129	ENSG00000163145.13	C1QTNF7	114905	-3.37037941	2.72E-41	4.09E-39
130	ENSG00000141750.7	STAC2	342667	-3.975604489	4.00E-41	5.97E-39
131	ENSG00000170417.16	TMEM182	130827	-2.410745341	4.01E-41	5.97E-39
132	ENSG00000140795.13	MYLK3	91807	-5.143740535	4.73E-41	7.02E-39
133	ENSG00000167701.14	GPT	2875	-3.114131052	6.24E-41	9.20E-39
134	ENSG00000161649.13	CD300LG	146894	-3.870200467	6.60E-41	9.69E-39
135	ENSG00000186815.13	TPCN1	53373	-1.221633218	7.63E-41	1.11E-38
136	ENSG00000145949.11	MYLK4	340156	-2.794885444	1.27E-40	1.82E-38
137	ENSG00000144771.8	LRTM1	57408	-5.775163786	1.61E-40	2.28E-38
138	ENSG00000277893.2	SRD5A2	6716	-4.363233798	1.79E-40	2.53E-38
139	ENSG00000095637.22	SORBS1	10580	-2.850089401	2.24E-40	3.13E-38
140	ENSG00000197321.15	SVIL	6840	-1.78099303	2.39E-40	3.32E-38
141	ENSG00000125733.18	TRIP10	9322	-1.386433634	2.70E-40	3.74E-38
142	ENSG00000284968.1			-1.305255076	2.72E-40	3.75E-38
143	ENSG00000105929.16	ATP6V0A4	50617	-3.999854352	3.26E-40	4.49E-38
144	ENSG00000181322.15	NME9	347736	-2.689873415	3.69E-40	5.07E-38
145	ENSG00000158008.10	EXTL1	2134	-4.5414858	4.50E-40	6.14E-38
146	ENSG00000255471.1	PRSS23-AS1		-2.717556875	5.20E-40	7.01E-38
147	ENSG00000160868.15	CYP3A4	1576	-4.05800006	6.13E-40	8.24E-38
148	ENSG00000075239.14	ACAT1	38	-1.477282679	7.84E-40	1.04E-37
149	ENSG00000172458.4	IL17D	53342	-2.959345735	8.59E-40	1.13E-37
150	ENSG00000068976.14	PYGM	5837	-5.899413636	1.06E-39	1.39E-37
151	ENSG00000001626.16	CFTR	1080	-5.121662413	1.10E-39	1.43E-37
152	ENSG00000090382.7	LYZ	4069	-4.0155661	1.11E-39	1.44E-37
153	ENSG00000141965.5	FEM1A	55527	-1.910005989	1.26E-39	1.63E-37
154	ENSG00000183346.9	CABCOCO1	219621	-4.0992035	1.50E-39	1.90E-37
155	ENSG00000198099.9	ADH4	127	-4.354567101	1.62E-39	2.05E-37
156	ENSG00000066185.13	ZMYND12	84217	-2.645863152	1.63E-39	2.05E-37
157	ENSG00000074964.17	ARHGEF10L	55160	-1.590047068	1.93E-39	2.42E-37
158	ENSG00000157087.20	ATP2B2	491	-4.360636472	2.00E-39	2.50E-37
159	ENSG00000176387.7	HSD11B2	3291	-2.298754073	2.03E-39	2.53E-37
160	ENSG00000170011.14	MYRIP	25924	-3.554518147	2.56E-39	3.18E-37
161	ENSG00000170634.13	ACYP2	98	-1.387050605	2.76E-39	3.40E-37
162	ENSG00000214456.8	PLIN5	440503	-3.313638347	2.83E-39	3.47E-37
163	ENSG00000196296.14	ATP2A1	487	-5.545827876	3.85E-39	4.64E-37
164	ENSG00000185437.13	SH3BGR	6450	-3.448909913	4.08E-39	4.89E-37
165	ENSG00000021300.14	PLEKHB1	58473	-3.151687577	4.09E-39	4.89E-37
166	ENSG00000168477.19	TNXB	7148	-3.565992502	4.83E-39	5.76E-37
167	ENSG00000157150.5	TIMP4	7079	-3.291454794	5.58E-39	6.55E-37
168	ENSG00000165028.12	NIPSNAP3B	55335	-2.698518207	5.72E-39	6.70E-37
169	ENSG00000254343.2			-3.065426051	7.10E-39	8.26E-37
170	ENSG00000179636.15	TPPP2	122664	-3.837690665	1.00E-38	1.16E-36
171	ENSG00000117640.18	MTFR1L	56181	-1.068400442	1.31E-38	1.50E-36
172	ENSG00000150593.18	PDCD4	27250	-1.421145355	1.82E-38	2.07E-36
173	ENSG00000165410.15	CFL2	1073	-2.235181944	2.06E-38	2.32E-36
174	ENSG00000088992.18	TESC	54997	-3.348734069	2.18E-38	2.45E-36
175	ENSG00000135443.8	KRT85	3891	-5.342185859	2.50E-38	2.80E-36
176	ENSG00000154269.15	ENPP3	5169	-3.984325697	3.60E-38	3.98E-36
177	ENSG00000076555.15	ACACB	32	-2.386902095	3.82E-38	4.21E-36
178	ENSG00000204071.10	TCEAL6	158931	-4.129575171	4.67E-38	5.08E-36
179	ENSG00000197766.8	CFD	1675	-2.565282325	4.78E-38	5.18E-36
180	ENSG00000154556.18	SORBS2	8470	-2.932948791	5.92E-38	6.36E-36
181	ENSG00000004799.8	PDK4	5166	-4.235041497	6.42E-38	6.88E-36
182	ENSG00000196666.6	FAM180B	399888	-4.205108444	7.10E-38	7.58E-36
183	ENSG00000188338.15	SLC38A3	10991	-3.637050964	7.36E-38	7.84E-36
184	ENSG00000143365.19	RORC	6097	-3.177809104	7.55E-38	8.03E-36
185	ENSG00000155792.10	DEPTOR	64798	-2.84106525	8.01E-38	8.47E-36
186	ENSG00000189129.14	PLAC9	219348	-2.834173342	8.40E-38	8.86E-36
187	ENSG00000134020.8	PEBP4	157310	-5.237663555	1.14E-37	1.20E-35
188	ENSG00000198648.11	STK39	27347	-1.383674033	1.71E-37	1.77E-35
189	ENSG00000162461.8	SLC25A34	284723	-3.196579212	1.97E-37	2.03E-35
190	ENSG00000120215.10	MLANA	2315	-2.618027373	2.59E-37	2.64E-35
191	ENSG00000162998.5	FRZB	2487	-3.073719883	3.48E-37	3.52E-35
192	ENSG00000186481.16	ANKRD20A5P		-4.472649061	3.61E-37	3.63E-35
193	ENSG00000128849.11	CGNL1	84952	-3.037930372	3.98E-37	3.99E-35
194	ENSG00000253177.2		101926956	-3.260884076	6.04E-37	5.99E-35
195	ENSG00000124205.18	EDN3	1908	-6.045298569	6.18E-37	6.11E-35
196	ENSG00000164879.7	CA3	761	-5.186393397	7.35E-37	7.19E-35
197	ENSG00000178104.19	PDE4DIP	9659	-2.436173392	7.82E-37	7.63E-35
198	ENSG00000232046.7	LINC01798	101060019	-3.125124616	8.11E-37	7.88E-35
199	ENSG00000164776.10	PHKG1	5260	-3.640178748	8.68E-37	8.41E-35
200	ENSG00000118307.20	DNAI7	55259	-3.316951629	1.79E-36	1.72E-34
201	ENSG00000196616.14	ADH1B	125	-5.439466608	1.95E-36	1.88E-34
202	ENSG00000172508.10	CARNS1	57571	-2.940819419	2.12E-36	2.02E-34
203	ENSG00000283294.1			-4.159855067	2.17E-36	2.07E-34
204	ENSG00000263155.6	MYZAP	100820829	-2.893433008	2.80E-36	2.65E-34
205	ENSG00000116194.13	ANGPTL1	9068	-3.700695773	3.20E-36	3.01E-34
206	ENSG00000249249.2	TICAM2-AS1		-1.895593633	4.02E-36	3.78E-34
207	ENSG00000111961.18	SASH1	23328	-2.202116239	4.13E-36	3.87E-34
208	ENSG00000119457.8	SLC46A2	57864	-3.149318315	5.47E-36	5.10E-34
209	ENSG00000258604.1			-3.883565794	5.62E-36	5.23E-34
210	ENSG00000034971.17	MYOC	4653	-6.709922259	6.08E-36	5.60E-34
211	ENSG00000112782.19	CLIC5	53405	-3.455854439	6.63E-36	6.07E-34
212	ENSG00000185250.16	PPIL6	285755	-2.450282462	6.72E-36	6.14E-34
213	ENSG00000166912.17	MTMR10	54893	-1.043665878	8.17E-36	7.40E-34
214	ENSG00000111911.7	HINT3	135114	-1.025123117	8.18E-36	7.40E-34
215	ENSG00000234478.1	ACBD3-AS1		-2.262845803	8.34E-36	7.53E-34
216	ENSG00000103184.12	SEC14L5	9717	-2.911422791	9.81E-36	8.80E-34
217	ENSG00000177363.5	LRRN4CL	221091	-2.648286038	1.14E-35	1.02E-33
218	ENSG00000077157.22	PPP1R12B	4660	-2.189681094	1.32E-35	1.17E-33
219	ENSG00000272081.1	FCHO2-DT		-1.667235077	1.70E-35	1.50E-33
220	ENSG00000078596.11	ITM2A	9452	-2.332217398	2.36E-35	2.07E-33
221	ENSG00000248746.6	ACTN3	89	-4.53725348	2.41E-35	2.11E-33
222	ENSG00000178965.14	ERICH3	127254	-5.469180069	2.63E-35	2.29E-33
223	ENSG00000164265.9	SCGB3A2	117156	-5.407918456	3.27E-35	2.84E-33
224	ENSG00000163431.13	LMOD1	25802	-2.64984141	3.79E-35	3.27E-33
225	ENSG00000126218.12	F10	2159	-2.505867111	4.54E-35	3.91E-33
226	ENSG00000196177.13	ACADSB	36	-1.766031374	4.91E-35	4.19E-33
227	ENSG00000096006.12	CRISP3	10321	-6.278417552	7.00E-35	5.95E-33
228	ENSG00000231246.2	LINC02884	105378909	-2.792514017	7.22E-35	6.11E-33
229	ENSG00000138771.16	SHROOM3	57619	-2.127262294	8.85E-35	7.46E-33
230	ENSG00000179564.4	LSMEM2	132228	-3.73327773	1.21E-34	1.01E-32
231	ENSG00000163071.11	SPATA18	132671	-2.217178917	1.23E-34	1.02E-32
232	ENSG00000162643.13	DNAI3	126820	-2.851857148	1.39E-34	1.16E-32
233	ENSG00000101605.13	MYOM1	8736	-4.464013688	1.49E-34	1.24E-32
234	ENSG00000138593.9	SECISBP2L	9728	-1.247954715	1.49E-34	1.24E-32
235	ENSG00000006059.4	KRT33A	3883	-4.070270104	2.04E-34	1.68E-32
236	ENSG00000186868.16	MAPT	4137	-3.414741703	2.34E-34	1.92E-32
237	ENSG00000139914.6	FITM1	161247	-3.875437835	2.61E-34	2.14E-32
238	ENSG00000126337.13	KRT36	8689	-6.586362307	3.07E-34	2.49E-32
239	ENSG00000109906.14	ZBTB16	7704	-3.659478676	3.80E-34	3.08E-32
240	ENSG00000112530.11	PACRG	135138	-3.589343985	4.22E-34	3.40E-32
241	ENSG00000114738.11	MAPKAPK3	7867	-1.227071327	5.78E-34	4.56E-32
242	ENSG00000180875.5	GREM2	64388	-3.562686679	6.13E-34	4.80E-32
243	ENSG00000130653.16	PNPLA7	375775	-2.070175111	6.49E-34	5.08E-32
244	ENSG00000176533.13	GNG7	2788	-2.439730309	6.70E-34	5.23E-32
245	ENSG00000253368.4	TRNP1	388610	-2.434589207	8.58E-34	6.67E-32
246	ENSG00000171201.12	SMR3B	10879	-12.76290532	9.41E-34	7.28E-32
247	ENSG00000167748.11	KLK1	3816	-3.286625137	9.48E-34	7.30E-32
248	ENSG00000182253.15	SYNM	23336	-2.584396753	9.69E-34	7.43E-32
249	ENSG00000271850.2	LINC02343		-5.27243212	9.78E-34	7.49E-32
250	ENSG00000184811.4	TRARG1	286753	-4.91472812	1.01E-33	7.69E-32
251	ENSG00000184454.7	NCMAP	400746	-3.736558142	1.02E-33	7.75E-32
252	ENSG00000080546.13	SESN1	27244	-1.185488935	1.29E-33	9.80E-32
253	ENSG00000272549.1	LINC02538	401286	-4.274527591	1.42E-33	1.07E-31
254	ENSG00000164120.14	HPGD	3248	-3.276061716	1.52E-33	1.14E-31
255	ENSG00000157107.14	FCHO2	115548	-1.325056549	1.52E-33	1.14E-31
256	ENSG00000104763.20	ASAH1	427	-1.105800896	1.57E-33	1.17E-31
257	ENSG00000144655.15	CSRNP1	64651	-1.655732977	1.68E-33	1.25E-31
258	ENSG00000148218.16	ALAD	210	-1.014760106	1.80E-33	1.34E-31
259	ENSG00000099860.9	GADD45B	4616	-1.805076271	1.89E-33	1.40E-31
260	ENSG00000163694.15	RBM47	54502	-1.571663968	2.04E-33	1.50E-31
261	ENSG00000136546.16	SCN7A	6332	-3.69618562	2.19E-33	1.61E-31
262	ENSG00000000003.15	TSPAN6	7105	-1.470673913	2.85E-33	2.08E-31
263	ENSG00000006747.15	SCIN	85477	-3.080487965	2.91E-33	2.11E-31
264	ENSG00000230156.3			-4.918452763	3.34E-33	2.41E-31
265	ENSG00000198643.7	FAM3D	131177	-4.194397681	3.41E-33	2.46E-31
266	ENSG00000151498.12	ACAD8	27034	-1.070624028	3.60E-33	2.59E-31
267	ENSG00000170369.4	CST2	1470	-4.650106227	3.65E-33	2.62E-31
268	ENSG00000168658.19	VWA3B	200403	-3.025347722	4.22E-33	3.03E-31
269	ENSG00000172005.11	MAL	4118	-4.970467914	4.36E-33	3.11E-31
270	ENSG00000271926.1			-1.922901593	4.49E-33	3.20E-31
271	ENSG00000183154.1		102723701	-2.097558093	4.58E-33	3.26E-31
272	ENSG00000216921.9	FAM240C	285095	-4.189836622	4.95E-33	3.51E-31
273	ENSG00000143416.21	SELENBP1	8991	-2.711261657	6.43E-33	4.53E-31
274	ENSG00000214711.10	CAPN14	440854	-4.274672989	6.93E-33	4.87E-31
275	ENSG00000121577.14	POPDC2	64091	-2.624664978	7.18E-33	5.03E-31
276	ENSG00000232415.1	ELN-AS1	107986809	-3.269347427	8.04E-33	5.62E-31
277	ENSG00000100373.10	UPK3A	7380	-3.355542429	8.28E-33	5.77E-31
278	ENSG00000231170.7	PDK4-AS1		-2.733188991	8.57E-33	5.95E-31
279	ENSG00000158445.10	KCNB1	3745	-3.207897192	1.16E-32	8.05E-31
280	ENSG00000188931.4	CFAP126	257177	-1.724798303	1.17E-32	8.07E-31
281	ENSG00000072954.7	TMEM38A	79041	-2.986661998	1.40E-32	9.62E-31
282	ENSG00000265142.9	MIR133A1HG	102723167	-5.630762569	1.47E-32	1.01E-30
283	ENSG00000074416.14	MGLL	11343	-2.01357112	1.62E-32	1.11E-30
284	ENSG00000185942.13	NKAIN3	286183	-3.059251115	1.86E-32	1.27E-30
285	ENSG00000185345.23	PRKN	5071	-2.586171281	2.43E-32	1.65E-30
286	ENSG00000164068.16	RNF123	63891	-1.269477259	2.63E-32	1.78E-30
287	ENSG00000079739.17	PGM1	5236	-1.324941037	2.72E-32	1.83E-30
288	ENSG00000138796.17	HADH	3033	-1.144247765	2.73E-32	1.84E-30
289	ENSG00000166816.15	LDHD	197257	-2.366912782	3.10E-32	2.08E-30
290	ENSG00000281386.1			-3.901122769	3.45E-32	2.30E-30
291	ENSG00000170871.12	KIAA0232	9778	-1.067033784	3.48E-32	2.32E-30
292	ENSG00000230657.8	PRB4	5545	-8.194694022	4.50E-32	2.98E-30
293	ENSG00000064886.14	CHI3L2	1117	-3.326873326	4.64E-32	3.07E-30
294	ENSG00000170423.13	KRT78	196374	-4.234211147	4.65E-32	3.07E-30
295	ENSG00000174876.16	AMY1B	277	-6.830831213	5.42E-32	3.54E-30
296	ENSG00000122367.20	LDB3	11155	-4.721708327	7.18E-32	4.63E-30
297	ENSG00000185046.18	ANKS1B	56899	-2.572569629	7.44E-32	4.79E-30
298	ENSG00000170477.13	KRT4	3851	-5.509657543	7.45E-32	4.79E-30
299	ENSG00000130396.20	AFDN	4301	-1.262127014	7.79E-32	5.00E-30
300	ENSG00000007216.15	SLC13A2	9058	-4.718114061	7.83E-32	5.02E-30
301	ENSG00000144821.10	MYH15	22989	-3.346151841	9.02E-32	5.76E-30
302	ENSG00000022267.19	FHL1	2273	-3.66208352	9.08E-32	5.79E-30
303	ENSG00000125246.15	CLYBL	171425	-1.706229477	1.00E-31	6.39E-30
304	ENSG00000089101.19	CFAP61	26074	-2.948282088	1.14E-31	7.22E-30
305	ENSG00000131738.11	KRT33B	3884	-4.636423347	1.19E-31	7.52E-30
306	ENSG00000198467.16	TPM2	7169	-3.006307415	1.50E-31	9.44E-30
307	ENSG00000272975.1	MYHAS	100128560	-3.914912678	1.59E-31	9.95E-30
308	ENSG00000112186.12	CAP2	10486	-2.497731547	1.60E-31	1.00E-29
309	ENSG00000166123.14	GPT2	84706	-1.753850152	1.63E-31	1.01E-29
310	ENSG00000228126.1	FALEC	100874054	-3.288988971	1.63E-31	1.02E-29
311	ENSG00000113645.15	WWC1	23286	-1.451126204	1.93E-31	1.19E-29
312	ENSG00000198888.2	MT-ND1	4535	-1.600434538	2.10E-31	1.29E-29
313	ENSG00000118898.16	PPL	5493	-2.400978801	2.29E-31	1.41E-29
314	ENSG00000197769.6	MAP1LC3C	440738	-2.818914846	2.55E-31	1.56E-29
315	ENSG00000171033.13	PKIA	5569	-3.192610871	2.67E-31	1.62E-29
316	ENSG00000175564.13	UCP3	7352	-3.100912158	2.78E-31	1.69E-29
317	ENSG00000175198.17	PCCA	5095	-1.215642268	3.80E-31	2.28E-29
318	ENSG00000121769.8	FABP3	2170	-3.114833213	4.81E-31	2.87E-29
319	ENSG00000119938.9	PPP1R3C	5507	-3.203392628	4.98E-31	2.97E-29
320	ENSG00000249464.6	LINC01091	285419	-2.494706598	6.93E-31	4.07E-29
321	ENSG00000227695.6	DNMBP-AS1		-3.350483059	6.95E-31	4.08E-29
322	ENSG00000186687.16	LYRM7	90624	-1.033070272	7.05E-31	4.12E-29
323	ENSG00000245281.8	ASAH1-AS1		-1.641219077	8.75E-31	5.10E-29
324	ENSG00000157500.12	APPL1	26060	-1.018273121	9.21E-31	5.35E-29
325	ENSG00000159763.4	PIP	5304	-8.473275464	9.83E-31	5.70E-29
326	ENSG00000129250.12	KIF1C	10749	-1.260382151	1.08E-30	6.23E-29
327	ENSG00000203688.6	LINC02487		-4.675277334	1.20E-30	6.90E-29
328	ENSG00000198523.6	PLN	5350	-3.788213458	1.24E-30	7.15E-29
329	ENSG00000174611.12	KY	339855	-3.826174804	1.26E-30	7.23E-29
330	ENSG00000185069.2	KRT76	51350	-5.756120298	1.31E-30	7.49E-29
331	ENSG00000168564.6	CDKN2AIP	55602	-1.048864827	1.31E-30	7.49E-29
332	ENSG00000178568.15	ERBB4	2066	-4.107968721	1.46E-30	8.29E-29
333	ENSG00000164972.13	SPMIP6	84688	-3.267847693	1.56E-30	8.87E-29
334	ENSG00000181830.9	SLC35C1	55343	-1.203853103	1.65E-30	9.34E-29
335	ENSG00000161664.7	ASB16	92591	-2.383258035	1.72E-30	9.77E-29
336	ENSG00000266145.1	RHOT1P1		-4.490999784	1.73E-30	9.78E-29
337	ENSG00000145362.20	ANK2	287	-2.793454785	1.78E-30	1.01E-28
338	ENSG00000170545.17	SMAGP	57228	-1.439304496	1.79E-30	1.01E-28
339	ENSG00000260398.1			-3.580223851	1.79E-30	1.01E-28
340	ENSG00000154479.13	CFAP210	129881	-2.666432811	1.92E-30	1.08E-28
341	ENSG00000141655.17	TNFRSF11A	8792	-1.937728037	1.93E-30	1.08E-28
342	ENSG00000163879.11	DNALI1	7802	-2.619139458	2.00E-30	1.12E-28
343	ENSG00000079385.23	CEACAM1	634	-2.657340764	2.14E-30	1.20E-28
344	ENSG00000267653.1			-5.290434708	2.15E-30	1.20E-28
345	ENSG00000142623.11	PADI1	29943	-3.800670965	2.17E-30	1.21E-28
346	ENSG00000212900.3	KRTAP3-2	83897	-7.12089359	2.52E-30	1.40E-28
347	ENSG00000196482.18	ESRRG	2104	-3.854246962	2.54E-30	1.41E-28
348	ENSG00000188277.10	C15orf62	643338	-2.485180505	3.28E-30	1.81E-28
349	ENSG00000261272.1	MUC22	100507679	-5.854630639	3.61E-30	1.98E-28
350	ENSG00000166046.11	TCP11L2	255394	-1.605958834	3.70E-30	2.03E-28
351	ENSG00000183386.10	FHL3	2275	-1.672109976	5.34E-30	2.89E-28
352	ENSG00000196218.13	RYR1	6261	-2.97766604	5.64E-30	3.05E-28
353	ENSG00000119711.13	ALDH6A1	4329	-1.471714428	8.07E-30	4.33E-28
354	ENSG00000132554.20	RGS22	26166	-3.27438773	8.69E-30	4.65E-28
355	ENSG00000065717.15	TLE2	7089	-2.441752048	9.25E-30	4.93E-28
356	ENSG00000175946.8	KLHL38	340359	-4.046211028	9.74E-30	5.19E-28
357	ENSG00000196187.12	TMEM63A	9725	-1.138031386	1.16E-29	6.18E-28
358	ENSG00000114853.14	ZBTB47	92999	-1.591245322	1.23E-29	6.53E-28
359	ENSG00000230873.9	STMND1	401236	-4.489722486	1.25E-29	6.61E-28
360	ENSG00000163075.13	CFAP221	200373	-3.674058628	1.26E-29	6.67E-28
361	ENSG00000211445.12	GPX3	2878	-2.528240739	1.36E-29	7.16E-28
362	ENSG00000272274.2	ARGLU1-DT		-2.371496491	1.39E-29	7.31E-28
363	ENSG00000205683.11	DPF3	8110	-2.42356248	1.40E-29	7.35E-28
364	ENSG00000100626.17	GALNT16	57452	-3.175310854	1.80E-29	9.38E-28
365	ENSG00000058056.9	USP13	8975	-2.000589617	2.20E-29	1.15E-27
366	ENSG00000131781.13	FMO5	2330	-1.76683318	2.32E-29	1.20E-27
367	ENSG00000213171.3	LINGO4	339398	-3.726718814	2.53E-29	1.31E-27
368	ENSG00000142197.12	DOP1B	9980	-1.300721926	2.86E-29	1.47E-27
369	ENSG00000122477.13	LRRC39	127495	-3.483539176	3.02E-29	1.55E-27
370	ENSG00000214872.8	SMTNL1	219537	-4.087769036	3.05E-29	1.56E-27
371	ENSG00000108932.12	SLC16A6	9120	-2.215573452	3.51E-29	1.79E-27
372	ENSG00000164591.14	MYOZ3	91977	-4.339796002	3.56E-29	1.82E-27
373	ENSG00000153113.24	CAST		-1.063615934	3.65E-29	1.86E-27
374	ENSG00000164197.12	RNF180	285671	-2.067901402	3.94E-29	2.00E-27
375	ENSG00000124743.6	KLHL31	401265	-3.088850306	4.01E-29	2.04E-27
376	ENSG00000180769.10	WDFY3-AS2	404201	-1.359980227	4.64E-29	2.35E-27
377	ENSG00000108759.4	KRT32	3882	-3.264498208	4.66E-29	2.35E-27
378	ENSG00000134548.11	SPX	80763	-4.032659687	4.87E-29	2.45E-27
379	ENSG00000258952.2	SALRNA1		-2.500714593	4.89E-29	2.46E-27
380	ENSG00000004776.13	HSPB6	126393	-4.230304299	5.00E-29	2.50E-27
381	ENSG00000183347.15	GBP6	163351	-2.596348121	5.15E-29	2.57E-27
382	ENSG00000137634.10	NXPE4	54827	-4.780906282	5.62E-29	2.80E-27
383	ENSG00000234438.5	KBTBD13	390594	-5.304640202	6.09E-29	3.03E-27
384	ENSG00000246375.3	PPM1K-DT	105369192	-2.428566757	6.27E-29	3.11E-27
385	ENSG00000016602.9	CLCA4	22802	-3.991654544	6.42E-29	3.18E-27
386	ENSG00000198221.10	AFDN-DT	26238	-1.926460272	6.76E-29	3.34E-27
387	ENSG00000189134.4	NKAPL	222698	-2.449101529	7.34E-29	3.62E-27
388	ENSG00000172159.16	FRMD3	257019	-2.083724397	7.35E-29	3.62E-27
389	ENSG00000185873.8	TMPRSS11B	132724	-5.248873153	7.38E-29	3.63E-27
390	ENSG00000170271.11	FAXDC2	10826	-2.067047281	7.44E-29	3.66E-27
391	ENSG00000119929.13	CUTC	51076	-1.141138752	7.48E-29	3.67E-27
392	ENSG00000196169.15	KIF19	124602	-2.487003474	8.77E-29	4.28E-27
393	ENSG00000187098.17	MITF	4286	-2.044614805	8.83E-29	4.31E-27
394	ENSG00000198786.2	MT-ND5	4540	-1.699122905	9.49E-29	4.62E-27
395	ENSG00000185100.11	ADSS1	122622	-2.24254862	1.01E-28	4.92E-27
396	ENSG00000118997.14	DNAH7	56171	-2.805649107	1.11E-28	5.37E-27
397	ENSG00000143318.13	CASQ1	844	-5.056370776	1.13E-28	5.47E-27
398	ENSG00000171476.22	HOPX	84525	-2.870514248	1.15E-28	5.57E-27
399	ENSG00000183018.9	SPNS2	124976	-2.455345141	1.26E-28	6.08E-27
400	ENSG00000013297.12	CLDN11	5010	-2.606526036	1.51E-28	7.24E-27
401	ENSG00000267417.1			-4.47964857	1.70E-28	8.18E-27
402	ENSG00000204950.4	LRRC10B	390205	-3.222914912	1.87E-28	8.95E-27
403	ENSG00000187783.12	TMEM72	643236	-4.486093525	2.25E-28	1.07E-26
404	ENSG00000144674.16	GOLGA4	2803	-1.093222352	2.46E-28	1.17E-26
405	ENSG00000172348.15	RCAN2	10231	-2.294693883	2.71E-28	1.28E-26
406	ENSG00000177685.17	CRACR2B	283229	-2.554424642	2.77E-28	1.31E-26
407	ENSG00000116981.3	NT5C1A	84618	-5.267860329	2.87E-28	1.35E-26
408	ENSG00000144712.12	CAND2	23066	-2.957770064	3.08E-28	1.45E-26
409	ENSG00000267709.1		101928844	-3.615266722	3.18E-28	1.49E-26
410	ENSG00000160808.11	MYL3	4634	-4.553013612	3.25E-28	1.52E-26
411	ENSG00000150773.11	PIH1D2	120379	-1.484962607	3.62E-28	1.69E-26
412	ENSG00000214708.4		105371730	-2.18400566	3.78E-28	1.75E-26
413	ENSG00000250282.1	PDK2-AS1		-3.624087113	3.92E-28	1.81E-26
414	ENSG00000165197.5	VEGFD	2277	-3.019863174	4.09E-28	1.89E-26
415	ENSG00000064651.14	SLC12A2	6558	-1.568003132	4.18E-28	1.93E-26
416	ENSG00000122359.18	ANXA11	311	-1.012268199	4.60E-28	2.12E-26
417	ENSG00000197561.7	ELANE	1991	-3.174610993	4.78E-28	2.19E-26
418	ENSG00000172731.14	LRRC20	55222	-1.869483293	5.81E-28	2.66E-26
419	ENSG00000188488.14	SERPINA5	5104	-3.556422218	6.28E-28	2.87E-26
420	ENSG00000135046.14	ANXA1	301	-2.141692975	6.49E-28	2.96E-26
421	ENSG00000160539.6	PLPP7	84814	-2.805139404	6.83E-28	3.10E-26
422	ENSG00000113328.19	CCNG1	900	-1.102331093	7.35E-28	3.33E-26
423	ENSG00000253958.2	CLDN23	137075	-1.828523674	1.05E-27	4.72E-26
424	ENSG00000011198.10	ABHD5	51099	-1.135918261	1.10E-27	4.91E-26
425	ENSG00000134962.7	KLB	152831	-2.290902126	1.15E-27	5.12E-26
426	ENSG00000144644.14	GADL1	339896	-4.402611196	1.16E-27	5.18E-26
427	ENSG00000132837.15	DMGDH	29958	-1.693119971	1.28E-27	5.68E-26
428	ENSG00000287146.1			-2.401858899	1.44E-27	6.40E-26
429	ENSG00000196782.12	MAML3	55534	-1.716612322	1.48E-27	6.57E-26
430	ENSG00000160862.13	AZGP1	563	-4.507851833	1.70E-27	7.51E-26
431	ENSG00000095321.17	CRAT	1384	-2.180427715	1.74E-27	7.66E-26
432	ENSG00000143028.9	SYPL2	284612	-4.007043203	1.95E-27	8.53E-26
433	ENSG00000131730.16	CKMT2	1160	-4.183122448	1.97E-27	8.62E-26
434	ENSG00000188037.12	CLCN1	1180	-2.662054417	1.98E-27	8.67E-26
435	ENSG00000276445.1			-2.989930323	2.08E-27	9.06E-26
436	ENSG00000163412.13	EIF4E3	317649	-1.371064449	2.15E-27	9.39E-26
437	ENSG00000163827.13	LRRC2	79442	-3.986518991	2.47E-27	1.07E-25
438	ENSG00000277496.1	SLCO4A1-AS2		-1.97151053	2.64E-27	1.14E-25
439	ENSG00000281097.1	LINC01395	101929557	-3.157812095	3.22E-27	1.39E-25
440	ENSG00000198807.13	PAX9	5083	-2.410060983	3.65E-27	1.57E-25
441	ENSG00000189350.13	TOGARAM2	165186	-2.489747075	3.76E-27	1.61E-25
442	ENSG00000184530.9	C6orf58	352999	-5.761606635	3.98E-27	1.71E-25
443	ENSG00000204323.5	SMIM5	643008	-2.646244045	4.10E-27	1.75E-25
444	ENSG00000251443.2	LINC02160	105374716	-4.739515869	4.56E-27	1.95E-25
445	ENSG00000185847.8	VHRT	100131138	-4.160416889	4.58E-27	1.95E-25
446	ENSG00000215559.8	ANKRD20A11P		-3.796573052	4.87E-27	2.07E-25
447	ENSG00000249743.6			-3.523587652	5.02E-27	2.13E-25
448	ENSG00000198932.13	GPRASP1	9737	-1.749016234	5.17E-27	2.19E-25
449	ENSG00000203734.12	ECT2L	345930	-2.592635168	5.28E-27	2.24E-25
450	ENSG00000143412.10	ANXA9	8416	-2.436450421	5.41E-27	2.29E-25
451	ENSG00000152611.12	CAPSL	133690	-4.784072373	6.33E-27	2.67E-25
452	ENSG00000145743.17	FBXL17	64839	-1.074217072	6.73E-27	2.83E-25
453	ENSG00000178537.10	SLC25A20	788	-1.095847662	6.74E-27	2.83E-25
454	ENSG00000265787.2	CYP4F35P		-4.916952544	6.79E-27	2.85E-25
455	ENSG00000241644.2	INMT	11185	-2.159312357	6.87E-27	2.88E-25
456	ENSG00000116748.21	AMPD1	270	-4.364521948	8.15E-27	3.40E-25
457	ENSG00000228314.1	CYP4F29P		-4.720524693	9.76E-27	4.05E-25
458	ENSG00000198804.2	MT-CO1	4512	-1.323809621	9.86E-27	4.09E-25
459	ENSG00000110911.16	SLC11A2	4891	-1.03483004	1.09E-26	4.48E-25
460	ENSG00000146469.13	VIP	7432	-3.47259654	1.14E-26	4.68E-25
461	ENSG00000110013.13	SIAE	54414	-1.312199077	1.20E-26	4.95E-25
462	ENSG00000172478.18	MAB21L4	79919	-2.995105211	1.23E-26	5.06E-25
463	ENSG00000101306.11	MYLK2	85366	-3.608859452	1.25E-26	5.13E-25
464	ENSG00000139531.13	SUOX	6821	-1.092926038	1.32E-26	5.39E-25
465	ENSG00000147166.11	ITGB1BP2	26548	-2.558826879	1.50E-26	6.13E-25
466	ENSG00000274214.1			-4.12549082	1.70E-26	6.93E-25
467	ENSG00000177994.16	CIMIP6	129852	-3.82568873	1.76E-26	7.12E-25
468	ENSG00000101542.10	CDH20	28316	-2.894944099	1.86E-26	7.52E-25
469	ENSG00000107165.13	TYRP1	7306	-3.953865845	1.92E-26	7.74E-25
470	ENSG00000157423.18	HYDIN	54768	-3.463896635	1.97E-26	7.94E-25
471	ENSG00000168026.18	TTC21A	199223	-1.529839248	2.17E-26	8.68E-25
472	ENSG00000197446.9	CYP2F1	1572	-5.056490808	2.22E-26	8.86E-25
473	ENSG00000133710.16	SPINK5	11005	-3.181915408	2.23E-26	8.89E-25
474	ENSG00000276317.1			-2.091318437	2.26E-26	9.03E-25
475	ENSG00000106078.19	COBL	23242	-2.808997297	2.28E-26	9.10E-25
476	ENSG00000277494.2	GPIHBP1	338328	-2.615277043	2.56E-26	1.02E-24
477	ENSG00000163606.11	CD200R1	131450	-2.191709665	2.65E-26	1.05E-24
478	ENSG00000176136.6	MC5R	4161	-4.264992321	2.78E-26	1.10E-24
479	ENSG00000162599.17	NFIA	4774	-1.163514845	2.91E-26	1.15E-24
480	ENSG00000177791.12	MYOZ1	58529	-4.739368572	3.08E-26	1.21E-24
481	ENSG00000164309.15	CMYA5	202333	-4.054673411	3.21E-26	1.26E-24
482	ENSG00000148339.12	SLC25A25	114789	-1.567935314	3.36E-26	1.32E-24
483	ENSG00000267060.7	PTGES3L	100885848	-2.184487596	3.71E-26	1.45E-24
484	ENSG00000168631.13	MUCL3	135656	-4.525500948	3.71E-26	1.45E-24
485	ENSG00000109819.9	PPARGC1A	10891	-3.007239187	3.84E-26	1.50E-24
486	ENSG00000189157.14	FAM47E	100129583	-2.092761606	3.91E-26	1.52E-24
487	ENSG00000151623.15	NR3C2	4306	-2.943192599	3.92E-26	1.52E-24
488	ENSG00000251152.1			-3.139456321	3.94E-26	1.53E-24
489	ENSG00000124003.13	MOGAT1	116255	-4.381966232	4.23E-26	1.64E-24
490	ENSG00000160349.10	LCN1	3933	-4.20680728	4.46E-26	1.73E-24
491	ENSG00000132122.12	SPATA6	54558	-1.841035265	4.95E-26	1.91E-24
492	ENSG00000163884.4	KLF15	28999	-2.895194074	5.45E-26	2.10E-24
493	ENSG00000005882.12	PDK2	5164	-1.196589424	5.62E-26	2.17E-24
494	ENSG00000277449.1	CEBPB-AS1		-1.636962286	5.72E-26	2.20E-24
495	ENSG00000146147.15	MLIP	90523	-3.049886112	5.95E-26	2.29E-24
496	ENSG00000150628.7	SPATA4	132851	-3.691404551	6.39E-26	2.44E-24
497	ENSG00000241158.7	ADAMTS9-AS1	101929335	-2.82440865	6.90E-26	2.63E-24
498	ENSG00000019102.12	VSIG2	23584	-3.39626262	7.63E-26	2.89E-24
499	ENSG00000261168.1			-3.184264307	8.20E-26	3.11E-24
500	ENSG00000184481.17	FOXO4	4303	-1.154616792	8.66E-26	3.27E-24
501	ENSG00000236882.8	LINC01554	202299	-3.117148177	9.29E-26	3.50E-24
502	ENSG00000187720.14	THSD4	79875	-1.777487356	9.33E-26	3.51E-24
503	ENSG00000198695.2	MT-ND6	4541	-1.644114081	1.01E-25	3.78E-24
504	ENSG00000138356.14	AOX1	316	-2.419629147	1.02E-25	3.81E-24
505	ENSG00000148429.14	USP6NL	9712	-1.236945787	1.07E-25	4.01E-24
506	ENSG00000175262.14	CIROZ	148345	-2.545755466	1.13E-25	4.19E-24
507	ENSG00000231621.1	ANKRD44-AS1		-3.767545791	1.19E-25	4.43E-24
508	ENSG00000119636.16	BBOF1	80127	-1.274832417	1.24E-25	4.60E-24
509	ENSG00000069702.11	TGFBR3	7049	-2.172293164	1.30E-25	4.81E-24
510	ENSG00000275025.1			-3.039384428	1.38E-25	5.07E-24
511	ENSG00000030419.16	IKZF2	22807	-1.950491817	1.46E-25	5.38E-24
512	ENSG00000109794.13	FAM149A	25854	-2.284523113	1.48E-25	5.44E-24
513	ENSG00000082146.13	STRADB	55437	-1.10136188	1.48E-25	5.44E-24
514	ENSG00000066583.12	ISOC1	51015	-1.013405052	1.64E-25	5.99E-24
515	ENSG00000112992.18	NNT	23530	-1.434326434	1.72E-25	6.24E-24
516	ENSG00000001561.7	ENPP4	22875	-2.45357149	1.76E-25	6.39E-24
517	ENSG00000198125.13	MB	4151	-4.628505611	1.80E-25	6.53E-24
518	ENSG00000188316.15	ENO4	387712	-2.376279105	1.89E-25	6.84E-24
519	ENSG00000091128.13	LAMB4	22798	-3.268894259	1.92E-25	6.92E-24
520	ENSG00000143434.16	SEMA6C	10500	-2.35827944	1.97E-25	7.08E-24
521	ENSG00000284391.1		105373244	-2.090236303	1.97E-25	7.08E-24
522	ENSG00000266524.3	GDF10	2662	-3.388723407	1.98E-25	7.11E-24
523	ENSG00000134243.12	SORT1	6272	-1.494415979	2.07E-25	7.40E-24
524	ENSG00000094963.14	FMO2	2327	-3.614336095	2.26E-25	8.07E-24
525	ENSG00000091137.14	SLC26A4	5172	-2.686961391	2.29E-25	8.18E-24
526	ENSG00000085871.9	MGST2	4258	-1.136306084	2.34E-25	8.34E-24
527	ENSG00000154330.13	PGM5	5239	-2.594941269	2.63E-25	9.32E-24
528	ENSG00000213088.12	ACKR1	2532	-2.825278998	2.73E-25	9.65E-24
529	ENSG00000259319.1	JDP2-AS1		-1.547540275	2.76E-25	9.76E-24
530	ENSG00000111077.18	TNS2	23371	-1.324725889	2.88E-25	1.01E-23
531	ENSG00000176974.20	SHMT1	6470	-1.189598721	2.97E-25	1.04E-23
532	ENSG00000174429.4	ABRA	137735	-5.111684769	3.10E-25	1.09E-23
533	ENSG00000185442.13	FAM174B	400451	-1.693430738	3.12E-25	1.09E-23
534	ENSG00000147576.17	ADHFE1	137872	-2.265224042	3.36E-25	1.18E-23
535	ENSG00000047346.12	ATOSA	56204	-1.157480138	3.45E-25	1.21E-23
536	ENSG00000288670.1			-1.036848638	3.49E-25	1.22E-23
537	ENSG00000164626.9	KCNK5	8645	-1.829758931	3.53E-25	1.23E-23
538	ENSG00000124713.6	GNMT	27232	-2.010129412	3.86E-25	1.34E-23
539	ENSG00000125648.15	SLC25A23	79085	-1.609578053	3.86E-25	1.34E-23
540	ENSG00000279255.1			-2.330977002	3.92E-25	1.36E-23
541	ENSG00000164056.11	SPRY1	10252	-1.398189254	4.30E-25	1.49E-23
542	ENSG00000224383.8	PRR29	92340	-1.827678063	4.38E-25	1.52E-23
543	ENSG00000185681.13	MORN5	254956	-5.713309852	4.45E-25	1.54E-23
544	ENSG00000133800.9	LYVE1	10894	-2.308836464	4.53E-25	1.57E-23
545	ENSG00000185432.12	TMT1A	25840	-2.068474401	4.69E-25	1.62E-23
546	ENSG00000132975.8	GPR12	2835	-5.144694026	4.73E-25	1.63E-23
547	ENSG00000005187.12	ACSM3	6296	-2.098069837	4.81E-25	1.66E-23
548	ENSG00000002587.10	HS3ST1	9957	-2.019019626	5.08E-25	1.75E-23
549	ENSG00000105641.4	SLC5A5	6528	-3.656251246	5.16E-25	1.77E-23
550	ENSG00000143036.17	SLC44A3	126969	-1.549719135	5.24E-25	1.80E-23
551	ENSG00000105519.18	CAPS	828	-2.644490645	5.39E-25	1.85E-23
552	ENSG00000172164.15	SNTB1	6641	-1.63827248	5.68E-25	1.94E-23
553	ENSG00000115226.10	FNDC4	64838	-1.812642197	5.96E-25	2.04E-23
554	ENSG00000164089.9	ETNPPL	64850	-5.409756717	6.03E-25	2.06E-23
555	ENSG00000134138.20	MEIS2	4212	-1.29056321	6.18E-25	2.11E-23
556	ENSG00000164440.15	TXLNB	167838	-3.375373364	6.26E-25	2.13E-23
557	ENSG00000234281.5	LANCL1-AS1		-2.982509272	6.48E-25	2.21E-23
558	ENSG00000286156.1	BMS1P23		-2.171622208	6.71E-25	2.28E-23
559	ENSG00000010017.13	RANBP9	10048	-1.041584161	6.76E-25	2.30E-23
560	ENSG00000274002.1			-4.760132169	7.92E-25	2.68E-23
561	ENSG00000233198.4	RNF224	643596	-2.599442253	8.53E-25	2.87E-23
562	ENSG00000203985.11	LDLRAD1	388633	-4.37924075	8.71E-25	2.93E-23
563	ENSG00000197245.6	FAM110D	79927	-1.734731333	8.79E-25	2.96E-23
564	ENSG00000117115.13	PADI2	11240	-3.285605006	9.62E-25	3.23E-23
565	ENSG00000252061.1	RNU6-415P		-1.582091263	1.11E-24	3.72E-23
566	ENSG00000189283.10	FHIT	2272	-1.833794129	1.23E-24	4.11E-23
567	ENSG00000243836.5	WDR86-AS1		-2.764655009	1.29E-24	4.29E-23
568	ENSG00000204909.8	SPINK9	643394	-4.784249919	1.29E-24	4.30E-23
569	ENSG00000141338.14	ABCA8	10351	-2.969604556	1.29E-24	4.31E-23
570	ENSG00000263370.1			-2.820325564	1.50E-24	4.97E-23
571	ENSG00000158828.8	PINK1	65018	-1.273799175	1.53E-24	5.05E-23
572	ENSG00000162399.9	BSND	7809	-3.768921921	1.61E-24	5.30E-23
573	ENSG00000163110.15	PDLIM5	10611	-1.082663075	1.61E-24	5.31E-23
574	ENSG00000188959.9	C9orf152	401546	-3.785683808	1.64E-24	5.40E-23
575	ENSG00000109846.9	CRYAB	1410	-2.693555486	1.70E-24	5.58E-23
576	ENSG00000135605.13	TEC	7006	-1.616416051	1.71E-24	5.64E-23
577	ENSG00000181234.9	TMEM132C	92293	-3.727079639	1.74E-24	5.72E-23
578	ENSG00000189058.9	APOD	347	-2.807312758	1.87E-24	6.12E-23
579	ENSG00000083123.15	BCKDHB	594	-1.222281757	1.96E-24	6.41E-23
580	ENSG00000120262.10	CCDC170	80129	-2.249544937	1.97E-24	6.41E-23
581	ENSG00000136153.20	LMO7	4008	-1.783454325	1.97E-24	6.43E-23
582	ENSG00000043039.7	BARX2	8538	-2.347691768	2.05E-24	6.68E-23
583	ENSG00000287594.1			-4.507437611	2.07E-24	6.74E-23
584	ENSG00000101470.10	TNNC2	7125	-4.482541287	2.13E-24	6.90E-23
585	ENSG00000161955.16	TNFSF13	8741	-1.438839801	2.13E-24	6.91E-23
586	ENSG00000118804.8	STBD1	8987	-1.585665974	2.26E-24	7.30E-23
587	ENSG00000189337.17	KAZN	23254	-1.22998075	2.74E-24	8.86E-23
588	ENSG00000110723.12	EXPH5	23086	-1.680585333	3.01E-24	9.66E-23
589	ENSG00000126016.16	AMOT	154796	-2.927524969	3.12E-24	9.99E-23
590	ENSG00000136830.12	NIBAN2	64855	-1.182462756	3.13E-24	1.00E-22
591	ENSG00000144857.14	BOC	91653	-1.865839346	3.16E-24	1.01E-22
592	ENSG00000166689.16	PLEKHA7	144100	-1.715062232	3.58E-24	1.14E-22
593	ENSG00000143850.16	PLEKHA6	22874	-2.126628399	3.60E-24	1.14E-22
594	ENSG00000145911.6	N4BP3	23138	-1.447312264	3.84E-24	1.22E-22
595	ENSG00000214128.11	TMEM213	155006	-5.788562809	4.04E-24	1.28E-22
596	ENSG00000251615.3			-1.658984935	4.07E-24	1.29E-22
597	ENSG00000159450.12	TCHH	7062	-4.135852501	4.27E-24	1.35E-22
598	ENSG00000036448.10	MYOM2	9172	-3.294970633	4.55E-24	1.44E-22
599	ENSG00000198939.8	ZFP2	80108	-1.609862173	4.83E-24	1.52E-22
600	ENSG00000106025.9	TSPAN12	23554	-2.101278179	4.86E-24	1.53E-22
601	ENSG00000287865.1			-3.757121839	5.26E-24	1.65E-22
602	ENSG00000136155.17	SCEL	8796	-2.893731979	5.44E-24	1.70E-22
603	ENSG00000101443.18	WFDC2	10406	-4.070792748	5.47E-24	1.71E-22
604	ENSG00000168334.9	XIRP1	165904	-3.975211514	5.50E-24	1.72E-22
605	ENSG00000159921.19	GNE	10020	-1.651868315	5.62E-24	1.76E-22
606	ENSG00000223547.10	ZNF844	284391	-1.937943303	5.68E-24	1.77E-22
607	ENSG00000156453.14	PCDH1	5097	-1.598712308	6.45E-24	2.01E-22
608	ENSG00000172915.18	NBEA	26960	-2.56677861	6.90E-24	2.14E-22
609	ENSG00000241684.6	ADAMTS9-AS2		-2.095023363	7.12E-24	2.21E-22
610	ENSG00000105048.17	TNNT1	7138	-3.19619879	7.36E-24	2.28E-22
611	ENSG00000079393.20	DUSP13B	51207	-3.60589972	7.64E-24	2.36E-22
612	ENSG00000168079.17	SCARA5	286133	-2.890656758	8.20E-24	2.53E-22
613	ENSG00000277639.2	CHD9NB	105371267	-2.658094082	8.84E-24	2.72E-22
614	ENSG00000210049.1	MT-TF		-2.348631027	9.58E-24	2.94E-22
615	ENSG00000064042.18	LIMCH1	22998	-2.302929934	1.02E-23	3.12E-22
616	ENSG00000132915.11	PDE6A	5145	-2.784396877	1.12E-23	3.41E-22
617	ENSG00000278445.1			-6.883279064	1.23E-23	3.75E-22
618	ENSG00000138764.15	CCNG2	901	-1.193711868	1.25E-23	3.79E-22
619	ENSG00000149575.9	SCN2B	6327	-2.747715525	1.31E-23	3.95E-22
620	ENSG00000274642.1			-3.735190009	1.33E-23	4.01E-22
621	ENSG00000160145.15	KALRN	8997	-1.654838606	1.33E-23	4.01E-22
622	ENSG00000123191.16	ATP7B	540	-1.73590952	1.35E-23	4.06E-22
623	ENSG00000171199.11	OPRPN	58503	-9.428961027	1.38E-23	4.16E-22
624	ENSG00000183230.17	CTNNA3	29119	-4.017784094	1.43E-23	4.30E-22
625	ENSG00000203952.9	CCDC160	347475	-3.947034285	1.51E-23	4.54E-22
626	ENSG00000018625.15	ATP1A2	477	-4.302749628	1.52E-23	4.56E-22
627	ENSG00000138744.16	NAAA	27163	-1.147796001	1.58E-23	4.74E-22
628	ENSG00000134240.12	HMGCS2	3158	-4.8054219	1.68E-23	5.02E-22
629	ENSG00000163157.15	TMOD4	29765	-3.648487292	1.75E-23	5.22E-22
630	ENSG00000164035.10	EMCN	51705	-1.701379542	1.79E-23	5.34E-22
631	ENSG00000120049.20	KCNIP2	30819	-1.902594147	1.83E-23	5.44E-22
632	ENSG00000188039.16	NWD1	284434	-3.064150341	1.87E-23	5.55E-22
633	ENSG00000287500.1			-2.639756701	2.04E-23	6.05E-22
634	ENSG00000084676.15	NCOA1	8648	-1.012662651	2.07E-23	6.14E-22
635	ENSG00000226026.6	RWDD3-DT	101928118	-2.44811327	2.26E-23	6.67E-22
636	ENSG00000226330.1	PPP1R15B-AS1		-2.685506758	2.40E-23	7.07E-22
637	ENSG00000244998.1	ASTILCS	105375790	-3.128695481	2.40E-23	7.09E-22
638	ENSG00000261068.2			-2.143599502	2.48E-23	7.30E-22
639	ENSG00000205592.15	MUC19	283463	-4.659495755	2.49E-23	7.34E-22
640	ENSG00000198574.6	SH2D1B	117157	-2.760321674	2.51E-23	7.39E-22
641	ENSG00000198727.2	MT-CYB	4519	-1.393396666	2.57E-23	7.56E-22
642	ENSG00000279164.1			-2.53845184	2.58E-23	7.58E-22
643	ENSG00000257108.2	NHLRC4	283948	-2.071220891	2.67E-23	7.82E-22
644	ENSG00000172738.12	TMEM217	221468	-1.463630224	2.81E-23	8.22E-22
645	ENSG00000135951.16	TSGA10	80705	-1.322093435	2.81E-23	8.24E-22
646	ENSG00000138162.19	TACC2	10579	-1.170721704	2.97E-23	8.68E-22
647	ENSG00000092607.15	TBX15	6913	-2.640058322	3.04E-23	8.88E-22
648	ENSG00000259616.2			-4.451639764	3.27E-23	9.53E-22
649	ENSG00000171714.12	ANO5	203859	-3.586047239	3.29E-23	9.56E-22
650	ENSG00000110080.20	ST3GAL4	6484	-1.336864316	3.37E-23	9.79E-22
651	ENSG00000166578.10	IQCD	115811	-1.688842108	3.52E-23	1.02E-21
652	ENSG00000007174.18	DNAH9	1770	-2.550567859	3.88E-23	1.12E-21
653	ENSG00000160796.18	NBEAL2	23218	-1.325084944	4.01E-23	1.16E-21
654	ENSG00000115896.16	PLCL1	5334	-1.829855953	4.05E-23	1.17E-21
655	ENSG00000136383.7	ALPK3	57538	-2.548813708	4.10E-23	1.18E-21
656	ENSG00000198390.5	KRTAP13-1	140258	-9.013432451	4.19E-23	1.20E-21
657	ENSG00000175161.14	CADM2	253559	-3.283243987	4.20E-23	1.21E-21
658	ENSG00000172382.10	PRSS27	83886	-2.921916079	4.32E-23	1.24E-21
659	ENSG00000180871.8	CXCR2	3579	-2.596006375	4.33E-23	1.24E-21
660	ENSG00000156222.12	SLC28A1	9154	-2.806312179	4.37E-23	1.25E-21
661	ENSG00000126878.13	AIF1L	83543	-2.041666892	4.51E-23	1.29E-21
662	ENSG00000072201.14	LNX1	84708	-1.674407993	4.52E-23	1.29E-21
663	ENSG00000137198.10	GMPR	2766	-2.098292137	4.76E-23	1.36E-21
664	ENSG00000160190.14	SLC37A1	54020	-1.47848784	4.78E-23	1.36E-21
665	ENSG00000013016.16	EHD3	30845	-1.455318517	4.97E-23	1.41E-21
666	ENSG00000185739.13	SRL	6345	-3.850104714	5.01E-23	1.42E-21
667	ENSG00000185015.8	CA13	377677	-1.694303251	5.06E-23	1.44E-21
668	ENSG00000125965.9	GDF5	8200	-3.134687484	5.22E-23	1.48E-21
669	ENSG00000164129.12	NPY5R	4889	-4.155504021	5.84E-23	1.65E-21
670	ENSG00000116885.18	OSCP1	127700	-1.184317159	6.64E-23	1.87E-21
671	ENSG00000153531.13	ADPRHL1	113622	-2.794708435	6.80E-23	1.91E-21
672	ENSG00000110002.16	VWA5A	4013	-1.550068243	6.92E-23	1.95E-21
673	ENSG00000163704.12	PRRT3	285368	-1.386355503	7.15E-23	2.01E-21
674	ENSG00000172201.12	ID4	3400	-1.870495186	7.20E-23	2.02E-21
675	ENSG00000163687.14	DNASE1L3	1776	-2.537700883	7.82E-23	2.19E-21
676	ENSG00000288009.1			-2.418551977	7.97E-23	2.22E-21
677	ENSG00000166246.14	DNAAF8	146562	-1.73199018	8.49E-23	2.37E-21
678	ENSG00000170364.12	SETMAR	6419	-1.098072193	8.67E-23	2.41E-21
679	ENSG00000163833.8	FBXO40	51725	-5.299922199	8.74E-23	2.43E-21
680	ENSG00000012223.13	LTF	4057	-4.86617115	9.82E-23	2.72E-21
681	ENSG00000166348.18	USP54	159195	-1.327136606	9.96E-23	2.76E-21
682	ENSG00000125744.12	RTN2	6253	-1.558846873	1.01E-22	2.78E-21
683	ENSG00000204544.5	MUC21	394263	-5.062094523	1.03E-22	2.84E-21
684	ENSG00000205312.8	KRT17P4		-3.617289744	1.08E-22	2.97E-21
685	ENSG00000092529.25	CAPN3	825	-2.237188133	1.14E-22	3.13E-21
686	ENSG00000072195.15	SPEG	10290	-2.186564703	1.17E-22	3.19E-21
687	ENSG00000198881.10	ASB12	142689	-3.512194157	1.24E-22	3.38E-21
688	ENSG00000274736.5	CCL23	6368	-2.332212298	1.24E-22	3.39E-21
689	ENSG00000080493.18	SLC4A4	8671	-2.9386103	1.38E-22	3.76E-21
690	ENSG00000184709.7	LRRC26	389816	-5.460413729	1.46E-22	3.96E-21
691	ENSG00000114737.16	CISH	1154	-1.221038851	1.47E-22	3.97E-21
692	ENSG00000164128.7	NPY1R	4886	-3.256643872	1.70E-22	4.59E-21
693	ENSG00000224982.4	TMEM233	387890	-2.39920911	1.77E-22	4.75E-21
694	ENSG00000128016.7	ZFP36	7538	-1.589132126	1.91E-22	5.13E-21
695	ENSG00000248290.1	TNXA		-3.152765424	1.95E-22	5.22E-21
696	ENSG00000118596.12	SLC16A7	9194	-2.124984687	1.99E-22	5.32E-21
697	ENSG00000161798.7	AQP5	362	-4.336503431	2.05E-22	5.49E-21
698	ENSG00000108231.14	LGI1	9211	-3.155751726	2.09E-22	5.58E-21
699	ENSG00000047648.23	ARHGAP6	395	-1.653662467	2.20E-22	5.86E-21
700	ENSG00000116574.6	RHOU	58480	-1.637557366	2.20E-22	5.87E-21
701	ENSG00000132840.10	BHMT2	23743	-1.992283575	2.35E-22	6.26E-21
702	ENSG00000212899.3	KRTAP3-3	85293	-8.8334064	2.45E-22	6.51E-21
703	ENSG00000174804.4	FZD4	8322	-1.391587996	2.49E-22	6.59E-21
704	ENSG00000278921.2	EPB41L4A-DT	54508	-1.645733515	2.61E-22	6.90E-21
705	ENSG00000162896.6	PIGR	5284	-4.805541844	2.63E-22	6.95E-21
706	ENSG00000111341.10	MGP	4256	-2.443031655	2.70E-22	7.14E-21
707	ENSG00000203867.8	RBM20	282996	-2.361420741	2.74E-22	7.24E-21
708	ENSG00000286449.1			-2.810845918	2.79E-22	7.35E-21
709	ENSG00000280362.1			-4.28517624	2.84E-22	7.48E-21
710	ENSG00000171401.15	KRT13	3860	-3.939880921	2.98E-22	7.85E-21
711	ENSG00000188761.13	BCL2L15	440603	-2.75065347	3.06E-22	8.03E-21
712	ENSG00000133985.3	TTC9	23508	-2.007355565	3.19E-22	8.38E-21
713	ENSG00000111405.9	ENDOU	8909	-3.422090572	3.31E-22	8.67E-21
714	ENSG00000088280.19	ASAP3	55616	-1.269128803	3.40E-22	8.90E-21
715	ENSG00000105711.13	SCN1B	6324	-2.353560457	3.55E-22	9.25E-21
716	ENSG00000006128.12	TAC1	6863	-4.429317454	3.65E-22	9.51E-21
717	ENSG00000110315.7	RNF141	50862	-1.021061503	4.06E-22	1.05E-20
718	ENSG00000266588.1			-2.636450707	4.13E-22	1.07E-20
719	ENSG00000272106.1			-1.256191652	4.15E-22	1.08E-20
720	ENSG00000010319.7	SEMA3G	56920	-1.746083784	4.17E-22	1.08E-20
721	ENSG00000198947.16	DMD	1756	-2.18716282	4.29E-22	1.11E-20
722	ENSG00000214942.6			-4.131335497	4.32E-22	1.11E-20
723	ENSG00000152137.8	HSPB8	26353	-2.078970419	4.57E-22	1.18E-20
724	ENSG00000225969.2	BICDL3P		-2.294528506	4.57E-22	1.18E-20
725	ENSG00000285564.2			-3.55631269	4.91E-22	1.26E-20
726	ENSG00000155085.15	AK9	221264	-1.181158784	5.03E-22	1.29E-20
727	ENSG00000223914.4	LINC02471		-3.628202545	5.44E-22	1.39E-20
728	ENSG00000203616.2	RHOT1P2		-3.449806682	5.62E-22	1.44E-20
729	ENSG00000103316.12	CRYM	1428	-2.978638204	6.05E-22	1.54E-20
730	ENSG00000130005.13	GAMT	2593	-1.949310316	6.29E-22	1.60E-20
731	ENSG00000013293.6	SLC7A14	57709	-3.870064734	6.36E-22	1.62E-20
732	ENSG00000276409.5	CCL14	6358	-3.038742232	6.84E-22	1.74E-20
733	ENSG00000166960.16	CCDC178	374864	-2.542092369	6.92E-22	1.76E-20
734	ENSG00000186335.9	SLC36A2	153201	-4.015722822	7.32E-22	1.86E-20
735	ENSG00000135447.17	PPP1R1A	5502	-4.383043	7.76E-22	1.97E-20
736	ENSG00000157330.10	CFAP107	93190	-4.93954876	8.01E-22	2.02E-20
737	ENSG00000145936.9	KCNMB1	3779	-1.603456759	8.22E-22	2.08E-20
738	ENSG00000165125.22	TRPV6	55503	-3.151977479	8.30E-22	2.09E-20
739	ENSG00000171885.18	AQP4	361	-4.45569429	8.66E-22	2.18E-20
740	ENSG00000215861.6			-3.091034136	8.80E-22	2.22E-20
741	ENSG00000168539.4	CHRM1	1128	-4.742835145	9.11E-22	2.29E-20
742	ENSG00000138166.6	DUSP5	1847	-1.582426976	9.35E-22	2.35E-20
743	ENSG00000135373.13	EHF	26298	-1.813572861	9.75E-22	2.45E-20
744	ENSG00000014257.16	ACP3	55	-2.05650137	1.01E-21	2.54E-20
745	ENSG00000197079.8	KRT35	3886	-3.997092081	1.02E-21	2.56E-20
746	ENSG00000285155.1			-3.217803364	1.06E-21	2.64E-20
747	ENSG00000143536.7	CRNN	49860	-4.810964117	1.10E-21	2.75E-20
748	ENSG00000264449.6		121725015	-3.633767781	1.11E-21	2.77E-20
749	ENSG00000113282.14	CLINT1	9685	-1.00328182	1.12E-21	2.78E-20
750	ENSG00000258616.5	LINC02303		-6.145060766	1.12E-21	2.80E-20
751	ENSG00000186710.11	CFAP73	387885	-2.463245363	1.21E-21	3.01E-20
752	ENSG00000142973.14	CYP4B1	1580	-3.558465773	1.30E-21	3.21E-20
753	ENSG00000260228.6	CDH13-AS2	102724163	-1.811688261	1.32E-21	3.27E-20
754	ENSG00000067177.15	PHKA1	5255	-1.289205916	1.34E-21	3.32E-20
755	ENSG00000182054.10	IDH2	3418	-1.095897394	1.41E-21	3.47E-20
756	ENSG00000113742.14	CPEB4	80315	-1.43530679	1.49E-21	3.67E-20
757	ENSG00000164736.6	SOX17	64321	-1.652204703	1.52E-21	3.74E-20
758	ENSG00000185028.4	LRRC14B	389257	-3.730328204	1.52E-21	3.74E-20
759	ENSG00000155850.8	SLC26A2	1836	-1.469593174	1.74E-21	4.28E-20
760	ENSG00000111058.8	ACSS3	79611	-2.193994022	1.76E-21	4.30E-20
761	ENSG00000118094.12	TREH	11181	-2.861197557	1.80E-21	4.41E-20
762	ENSG00000141905.19	NFIC	4782	-1.063413956	1.84E-21	4.50E-20
763	ENSG00000183172.9	SMDT1	91689	-1.039033007	1.92E-21	4.69E-20
764	ENSG00000062096.15	ARSF	416	-3.892535648	2.00E-21	4.89E-20
765	ENSG00000149305.7	HTR3B	9177	-4.12555658	2.02E-21	4.93E-20
766	ENSG00000108839.12	ALOX12	239	-2.550906802	2.03E-21	4.95E-20
767	ENSG00000213424.9	KRT222	125113	-3.030610061	2.11E-21	5.13E-20
768	ENSG00000118407.15	FILIP1	27145	-2.38637046	2.12E-21	5.14E-20
769	ENSG00000175664.10	TEX26	122046	-4.678206368	2.17E-21	5.25E-20
770	ENSG00000100628.12	ASB2	51676	-2.114508966	2.18E-21	5.28E-20
771	ENSG00000054598.9	FOXC1	2296	-1.54183722	2.30E-21	5.55E-20
772	ENSG00000135587.9	SMPD2	6610	-1.017042852	2.41E-21	5.81E-20
773	ENSG00000280285.1			-2.472511133	2.44E-21	5.88E-20
774	ENSG00000104848.1	KCNA7	3743	-3.186777705	2.48E-21	5.97E-20
775	ENSG00000070808.16	CAMK2A	815	-2.900163388	2.56E-21	6.15E-20
776	ENSG00000203727.4	SAMD5	389432	-2.459480502	2.69E-21	6.44E-20
777	ENSG00000174437.17	ATP2A2	488	-1.083449755	2.73E-21	6.53E-20
778	ENSG00000104369.5	JPH1	56704	-1.977827434	2.76E-21	6.60E-20
779	ENSG00000153822.14	KCNJ16	3773	-4.072768692	2.86E-21	6.82E-20
780	ENSG00000137098.14	SPAG8	26206	-2.00502913	3.04E-21	7.22E-20
781	ENSG00000058404.20	CAMK2B	816	-3.016496334	3.18E-21	7.54E-20
782	ENSG00000255727.2	LINC01489		-4.359375605	3.26E-21	7.73E-20
783	ENSG00000120907.18	ADRA1A	148	-3.377289579	3.36E-21	7.96E-20
784	ENSG00000091513.16	TF	7018	-3.328461305	3.44E-21	8.15E-20
785	ENSG00000109610.6	SOD3	6649	-1.717336592	3.73E-21	8.82E-20
786	ENSG00000287417.1			-3.434530344	3.76E-21	8.88E-20
787	ENSG00000215834.10	FMO9P		-3.432256726	3.94E-21	9.29E-20
788	ENSG00000243384.1			-3.308363538	4.02E-21	9.47E-20
789	ENSG00000078804.13	TP53INP2	58476	-1.565577778	4.08E-21	9.59E-20
790	ENSG00000143867.7	OSR1	130497	-2.310831889	4.11E-21	9.65E-20
791	ENSG00000197928.10	ZNF677	342926	-1.881791913	4.27E-21	1.00E-19
792	ENSG00000166292.12	TMEM100	55273	-2.468925164	4.37E-21	1.02E-19
793	ENSG00000113396.13	SLC27A6	28965	-3.605765813	4.59E-21	1.08E-19
794	ENSG00000174226.9	SNX31	169166	-3.219006965	4.98E-21	1.16E-19
795	ENSG00000120913.23	PDLIM2	64236	-1.429509524	5.03E-21	1.17E-19
796	ENSG00000259840.1			-2.415829566	5.30E-21	1.23E-19
797	ENSG00000135063.19	ENTREP1	9413	-2.484466914	5.49E-21	1.28E-19
798	ENSG00000286957.1			-3.108492073	5.51E-21	1.28E-19
799	ENSG00000286646.1	MAP3K5-AS2		-2.178950136	5.52E-21	1.28E-19
800	ENSG00000119514.7	GALNT12	79695	-1.830915952	5.61E-21	1.30E-19
801	ENSG00000062282.15	DGAT2	84649	-2.05319036	5.63E-21	1.30E-19
802	ENSG00000112164.6	GLP1R	2740	-2.196043923	5.81E-21	1.34E-19
803	ENSG00000167880.8	EVPL	2125	-1.707031966	6.22E-21	1.44E-19
804	ENSG00000177054.14	ZDHHC13	54503	-1.065465826	6.50E-21	1.50E-19
805	ENSG00000102362.15	SYTL4	94121	-1.913506303	6.92E-21	1.59E-19
806	ENSG00000165192.14	ASB11	140456	-4.990271042	6.95E-21	1.59E-19
807	ENSG00000087008.16	ACOX3	8310	-1.002694208	7.26E-21	1.66E-19
808	ENSG00000136457.10	CHAD	1101	-2.840531884	7.35E-21	1.68E-19
809	ENSG00000177106.16	EPS8L2	64787	-1.178654423	7.36E-21	1.68E-19
810	ENSG00000150054.18	MPP7	143098	-1.54764125	7.52E-21	1.72E-19
811	ENSG00000073464.13	CLCN4	1183	-2.172265996	8.36E-21	1.91E-19
812	ENSG00000261253.2			-1.77018413	8.38E-21	1.91E-19
813	ENSG00000150873.12	CIMIP5	130813	-2.251431433	8.50E-21	1.94E-19
814	ENSG00000150510.17	FAM124A	220108	-1.628903869	8.79E-21	2.00E-19
815	ENSG00000134716.11	CYP2J2	1573	-1.701864676	9.03E-21	2.06E-19
816	ENSG00000244734.4	HBB	3043	-2.995209429	9.07E-21	2.06E-19
817	ENSG00000136842.14	TMOD1	7111	-3.006921439	9.08E-21	2.06E-19
818	ENSG00000177182.11	CLVS1	157807	-2.332783098	9.56E-21	2.17E-19
819	ENSG00000092054.13	MYH7	4625	-5.14773617	9.90E-21	2.24E-19
820	ENSG00000130598.16	TNNI2	7136	-3.833744352	1.00E-20	2.26E-19
821	ENSG00000127318.11	IL22	50616	-4.658470059	1.14E-20	2.57E-19
822	ENSG00000182389.20	CACNB4	785	-2.501939377	1.20E-20	2.71E-19
823	ENSG00000179023.8	KLHDC7A	127707	-3.574456666	1.21E-20	2.71E-19
824	ENSG00000249797.2	LINC02147	124901051	-2.677818495	1.31E-20	2.95E-19
825	ENSG00000120915.14	EPHX2	2053	-1.693322975	1.45E-20	3.24E-19
826	ENSG00000102886.15	GDPD3	79153	-2.302513701	1.46E-20	3.26E-19
827	ENSG00000134352.20	IL6ST	3572	-1.012359858	1.51E-20	3.37E-19
828	ENSG00000104879.5	CKM	1158	-4.671355461	1.52E-20	3.38E-19
829	ENSG00000285774.3	SAMD4A-AS1		-4.046457926	1.52E-20	3.38E-19
830	ENSG00000158887.19	MPZ	4359	-2.186177569	1.52E-20	3.38E-19
831	ENSG00000107147.14	KCNT1	57582	-2.607896953	1.54E-20	3.44E-19
832	ENSG00000187824.9	TMEM220	388335	-1.46714885	1.63E-20	3.62E-19
833	ENSG00000127903.14	ZNF835	90485	-1.923895624	1.64E-20	3.63E-19
834	ENSG00000165807.8	PPP1R36	145376	-1.703503022	1.71E-20	3.79E-19
835	ENSG00000155657.28	TTN	7273	-3.816886699	1.72E-20	3.82E-19
836	ENSG00000092445.12	TYRO3	7301	-1.165807524	1.76E-20	3.91E-19
837	ENSG00000286722.1			-2.405102123	1.87E-20	4.13E-19
838	ENSG00000151726.15	ACSL1	2180	-1.264431444	1.88E-20	4.16E-19
839	ENSG00000137571.11	SLCO5A1	81796	-2.527641215	1.89E-20	4.17E-19
840	ENSG00000253313.6	C1orf210	149466	-1.38824765	1.95E-20	4.29E-19
841	ENSG00000213144.2			-2.198775167	2.24E-20	4.92E-19
842	ENSG00000007237.19	GAS7	8522	-1.735964506	2.37E-20	5.20E-19
843	ENSG00000132170.23	PPARG	5468	-2.27227001	2.60E-20	5.70E-19
844	ENSG00000225526.5	MKRN2OS	100129480	-1.74825831	2.66E-20	5.82E-19
845	ENSG00000259459.6	LINC02568		-2.828892709	2.68E-20	5.86E-19
846	ENSG00000158816.15	VWA5B1	127731	-3.151462107	2.74E-20	5.98E-19
847	ENSG00000171195.11	MUC7	4589	-7.443585623	2.75E-20	6.01E-19
848	ENSG00000138722.10	MMRN1	22915	-2.591786292	2.78E-20	6.06E-19
849	ENSG00000140297.13	GCNT3	9245	-2.731055751	2.81E-20	6.12E-19
850	ENSG00000120885.22	CLU	1191	-2.362204561	2.83E-20	6.15E-19
851	ENSG00000180354.16	MTURN	222166	-1.379192499	2.89E-20	6.27E-19
852	ENSG00000157110.16	RBPMS	11030	-1.226975051	2.91E-20	6.31E-19
853	ENSG00000165475.15	CRYL1	51084	-1.120062026	3.01E-20	6.54E-19
854	ENSG00000082014.17	SMARCD3	6604	-1.649193137	3.19E-20	6.91E-19
855	ENSG00000172318.5	B3GALT1	8708	-3.325722517	3.24E-20	7.02E-19
856	ENSG00000188001.10	TPRG1	285386	-1.864556109	3.32E-20	7.18E-19
857	ENSG00000159388.6	BTG2	7832	-1.346900665	3.35E-20	7.25E-19
858	ENSG00000129595.14	EPB41L4A	64097	-1.795643389	3.37E-20	7.27E-19
859	ENSG00000275131.3	PDE4DIPP2		-1.931564806	3.39E-20	7.32E-19
860	ENSG00000174473.16	GALNTL6	442117	-2.567352316	3.48E-20	7.50E-19
861	ENSG00000279127.1			-6.01937962	3.63E-20	7.80E-19
862	ENSG00000165495.16	PKNOX2	63876	-2.467466899	3.66E-20	7.86E-19
863	ENSG00000115648.14	MLPH	79083	-2.373772642	3.75E-20	8.04E-19
864	ENSG00000167614.14	TTYH1	57348	-2.095430876	3.79E-20	8.12E-19
865	ENSG00000159495.8	TGM7	116179	-4.210950976	3.89E-20	8.33E-19
866	ENSG00000236699.9	ARHGEF38	54848	-2.373418478	3.99E-20	8.52E-19
867	ENSG00000187151.7	ANGPTL5	253935	-3.733244826	4.12E-20	8.81E-19
868	ENSG00000253598.3	SLC10A5	347051	-1.630077234	4.17E-20	8.91E-19
869	ENSG00000150995.20	ITPR1	3708	-1.219571414	4.30E-20	9.16E-19
870	ENSG00000140416.23	TPM1	7168	-1.897909336	4.47E-20	9.50E-19
871	ENSG00000287561.1			-2.322279495	4.65E-20	9.88E-19
872	ENSG00000110195.14	FOLR1	2348	-3.765805838	4.73E-20	1.00E-18
873	ENSG00000263400.8	TMEM220-AS1	101101775	-1.834178723	4.76E-20	1.01E-18
874	ENSG00000134769.21	DTNA	1837	-2.542263118	5.03E-20	1.06E-18
875	ENSG00000259120.3	ERLN	100130933	-2.497803476	5.25E-20	1.11E-18
876	ENSG00000282885.2			-1.39961838	5.58E-20	1.18E-18
877	ENSG00000105204.14	DYRK1B	9149	-1.429090289	5.88E-20	1.23E-18
878	ENSG00000180209.12	MYL11	29895	-4.060960434	6.25E-20	1.31E-18
879	ENSG00000162409.11	PRKAA2	5563	-2.95409471	6.27E-20	1.31E-18
880	ENSG00000141576.16	RNF157	114804	-2.039329337	6.70E-20	1.40E-18
881	ENSG00000188817.8	SNTN	132203	-4.9996674	6.92E-20	1.44E-18
882	ENSG00000264920.2			-1.188996042	7.21E-20	1.50E-18
883	ENSG00000240583.13	AQP1	358	-1.387448307	7.43E-20	1.54E-18
884	ENSG00000124406.16	ATP8A1	10396	-1.803505468	7.60E-20	1.58E-18
885	ENSG00000182568.17	SATB1	6304	-1.434369781	8.16E-20	1.69E-18
886	ENSG00000102383.14	ZDHHC15	158866	-2.504271521	8.28E-20	1.71E-18
887	ENSG00000103089.9	FA2H	79152	-2.577463136	8.57E-20	1.77E-18
888	ENSG00000169562.13	GJB1	2705	-3.957036296	8.68E-20	1.79E-18
889	ENSG00000181378.14	CFAP65	255101	-4.038286843	8.73E-20	1.80E-18
890	ENSG00000272129.1			-1.169241128	8.73E-20	1.80E-18
891	ENSG00000198624.13	CCDC69	26112	-1.4735423	8.96E-20	1.85E-18
892	ENSG00000152078.10	TLCD4	148534	-2.287833973	9.33E-20	1.92E-18
893	ENSG00000101210.13	EEF1A2	1917	-3.344188954	9.51E-20	1.96E-18
894	ENSG00000103534.17	TMC5	79838	-2.97441929	9.62E-20	1.98E-18
895	ENSG00000070193.5	FGF10	2255	-2.929870793	9.71E-20	2.00E-18
896	ENSG00000105289.15	TJP3	27134	-2.667248437	1.03E-19	2.12E-18
897	ENSG00000166743.9	ACSM1	116285	-1.787839973	1.07E-19	2.19E-18
898	ENSG00000262921.1			-2.774117022	1.08E-19	2.21E-18
899	ENSG00000224905.7		105377134	-2.089629046	1.08E-19	2.21E-18
900	ENSG00000273540.4	AGBL1	123624	-3.773116093	1.17E-19	2.39E-18
901	ENSG00000006534.16	ALDH3B1	221	-1.546758923	1.26E-19	2.55E-18
902	ENSG00000176194.18	CIDEA	1149	-3.540164688	1.26E-19	2.55E-18
903	ENSG00000188112.9	C6orf132	647024	-1.387139288	1.27E-19	2.58E-18
904	ENSG00000184350.11	MRGPRE	116534	-3.063321161	1.34E-19	2.71E-18
905	ENSG00000235269.2	LINC02331	105370503	-4.810715701	1.35E-19	2.74E-18
906	ENSG00000196535.17	MYO18A	399687	-1.16772631	1.47E-19	2.97E-18
907	ENSG00000280213.1	UCKL1-AS1	100113386	-1.643440849	1.48E-19	2.99E-18
908	ENSG00000198840.2	MT-ND3	4537	-1.242562178	1.49E-19	3.00E-18
909	ENSG00000204219.11	TCEA3	6920	-1.800863809	1.51E-19	3.04E-18
910	ENSG00000119147.10	ECRG4	84417	-3.367194189	1.57E-19	3.16E-18
911	ENSG00000248713.1	C4orf54	285556	-3.857631881	1.69E-19	3.39E-18
912	ENSG00000262484.1	CCER2	643669	-3.005713637	1.70E-19	3.41E-18
913	ENSG00000163590.14	PPM1L	151742	-1.700788492	1.80E-19	3.60E-18
914	ENSG00000005981.13	ASB4	51666	-3.317351311	1.83E-19	3.66E-18
915	ENSG00000235097.1	LINC00330	144817	-3.87691546	1.89E-19	3.76E-18
916	ENSG00000234814.8	SVIL2P		-1.487457313	1.94E-19	3.85E-18
917	ENSG00000101400.6	SNTA1	6640	-1.434742229	1.95E-19	3.88E-18
918	ENSG00000156413.14	FUT6	2528	-3.18346926	2.01E-19	3.98E-18
919	ENSG00000282840.1	MSL3-DT		-2.399532427	2.02E-19	4.01E-18
920	ENSG00000064787.13	BCAS1	8537	-2.589255112	2.07E-19	4.10E-18
921	ENSG00000183091.19	NEB	4703	-3.776015085	2.14E-19	4.23E-18
922	ENSG00000162598.13	C1orf87	127795	-5.873110397	2.22E-19	4.38E-18
923	ENSG00000230965.1	SNX18P13		-3.817267699	2.22E-19	4.38E-18
924	ENSG00000111404.7	RERGL	79785	-2.814348037	2.51E-19	4.94E-18
925	ENSG00000156219.17	ART3	419	-3.070640936	2.65E-19	5.20E-18
926	ENSG00000182836.10	PLCXD3	345557	-3.06183735	2.78E-19	5.46E-18
927	ENSG00000175445.17	LPL	4023	-2.294800769	2.85E-19	5.58E-18
928	ENSG00000108691.9	CCL2	6347	-1.89859003	2.97E-19	5.80E-18
929	ENSG00000120693.14	SMAD9	4093	-1.633231979	3.06E-19	5.97E-18
930	ENSG00000134339.9	SAA2	6289	-2.859121977	3.10E-19	6.04E-18
931	ENSG00000287901.1			-2.804234697	3.19E-19	6.22E-18
932	ENSG00000079308.19	TNS1	7145	-1.561012787	3.24E-19	6.31E-18
933	ENSG00000149809.16	TM7SF2	7108	-1.877179911	3.31E-19	6.44E-18
934	ENSG00000130222.11	GADD45G	10912	-1.952956988	3.34E-19	6.50E-18
935	ENSG00000133112.17	TPT1	7178	-1.009934581	3.39E-19	6.59E-18
936	ENSG00000116299.17	ELAPOR1	57535	-2.941071907	3.55E-19	6.89E-18
937	ENSG00000160213.9	CSTB	1476	-1.860289101	3.57E-19	6.92E-18
938	ENSG00000232977.7	LINC00327	100506697	-2.034545288	3.64E-19	7.07E-18
939	ENSG00000127324.9	TSPAN8	7103	-3.392338832	3.67E-19	7.10E-18
940	ENSG00000196872.12	CRACDL	343990	-1.753282243	3.78E-19	7.31E-18
941	ENSG00000197585.10			-3.575096064	3.86E-19	7.46E-18
942	ENSG00000251655.6	PRB1	5542	-6.964870083	4.11E-19	7.94E-18
943	ENSG00000109738.11	GLRB	2743	-2.232941466	4.12E-19	7.94E-18
944	ENSG00000237949.2	LINC00844		-2.785373922	4.14E-19	7.98E-18
945	ENSG00000285991.1			-1.502109361	4.36E-19	8.39E-18
946	ENSG00000158528.12	PPP1R9A	55607	-3.106448399	4.36E-19	8.39E-18
947	ENSG00000259675.2			-5.080445407	4.47E-19	8.59E-18
948	ENSG00000246022.3	ALDH1L1-AS2	100862662	-3.754729772	4.84E-19	9.26E-18
949	ENSG00000176009.3	ASCL3	56676	-5.354402104	4.95E-19	9.47E-18
950	ENSG00000167741.11	GGT6	124975	-1.973633335	5.17E-19	9.89E-18
951	ENSG00000164530.15	PI16	221476	-3.448076216	5.35E-19	1.02E-17
952	ENSG00000198553.9	KCNRG	283518	-1.518554291	5.42E-19	1.03E-17
953	ENSG00000131849.12	ZNF132	7691	-1.034890517	5.69E-19	1.08E-17
954	ENSG00000007062.12	PROM1	8842	-3.904911359	6.30E-19	1.20E-17
955	ENSG00000111666.11	CHPT1	56994	-1.745764319	6.39E-19	1.21E-17
956	ENSG00000143196.5	DPT	1805	-2.370768185	6.40E-19	1.21E-17
957	ENSG00000227959.1	EPHA2-AS1		-1.365702988	6.46E-19	1.22E-17
958	ENSG00000235295.1	LINC01634	100192420	-2.819408365	6.63E-19	1.25E-17
959	ENSG00000006555.11	TTC22	55001	-1.383572642	6.96E-19	1.32E-17
960	ENSG00000167306.20	MYO5B	4645	-1.685683926	7.19E-19	1.36E-17
961	ENSG00000187550.9	SBK2	646643	-4.001417439	7.62E-19	1.43E-17
962	ENSG00000185863.8	TMEM210	100505993	-4.592023532	8.03E-19	1.51E-17
963	ENSG00000184544.12	DHRS7C	201140	-5.772780561	8.14E-19	1.53E-17
964	ENSG00000100321.15	SYNGR1	9145	-1.720009747	8.17E-19	1.53E-17
965	ENSG00000227630.4	LINC01132	100506810	-1.282861752	8.78E-19	1.64E-17
966	ENSG00000144730.19	IL17RD	54756	-1.522642671	8.98E-19	1.68E-17
967	ENSG00000088543.15	C3orf18	51161	-1.410463781	9.28E-19	1.73E-17
968	ENSG00000229380.1	PRKAR1B-AS2	101927000	-3.720807594	9.53E-19	1.78E-17
969	ENSG00000257743.8	MGAM2	93432	-4.489454332	9.87E-19	1.84E-17
970	ENSG00000140519.14	RHCG	51458	-2.920013741	1.03E-18	1.91E-17
971	ENSG00000067191.16	CACNB1	782	-2.01286949	1.06E-18	1.96E-17
972	ENSG00000228172.5			-1.018514676	1.06E-18	1.98E-17
973	ENSG00000101049.17	SGK2	10110	-2.640508417	1.12E-18	2.07E-17
974	ENSG00000184905.9	TCEAL2	140597	-3.211309457	1.13E-18	2.10E-17
975	ENSG00000157404.16	KIT	3815	-2.002754355	1.20E-18	2.22E-17
976	ENSG00000168447.11	SCNN1B	6338	-2.482449744	1.21E-18	2.24E-17
977	ENSG00000139780.7	METTL21C	196541	-5.010774539	1.22E-18	2.25E-17
978	ENSG00000189143.9	CLDN4	1364	-1.833944662	1.24E-18	2.29E-17
979	ENSG00000136689.19	IL1RN	3557	-2.040070863	1.29E-18	2.37E-17
980	ENSG00000116678.20	LEPR	3953	-1.588969119	1.31E-18	2.42E-17
981	ENSG00000196465.10	MYL6B	140465	-1.219550986	1.41E-18	2.58E-17
982	ENSG00000119900.9	OGFRL1	79627	-1.155890125	1.43E-18	2.62E-17
983	ENSG00000178690.3	DYNAP	284254	-4.738631128	1.47E-18	2.69E-17
984	ENSG00000074370.18	ATP2A3	489	-1.616829392	1.55E-18	2.84E-17
985	ENSG00000255986.6	MT1JP		-3.088955221	1.63E-18	2.97E-17
986	ENSG00000145675.15	PIK3R1	5295	-1.342062813	1.64E-18	2.99E-17
987	ENSG00000146809.13	ASB15	142685	-4.53115268	1.72E-18	3.13E-17
988	ENSG00000135917.16	SLC19A3	80704	-1.928400424	1.76E-18	3.21E-17
989	ENSG00000129744.3	ART1	417	-4.744135952	1.76E-18	3.21E-17
990	ENSG00000176920.13	FUT2	2524	-1.57374241	1.87E-18	3.40E-17
991	ENSG00000187908.20	DMBT1	1755	-4.537139608	1.99E-18	3.60E-17
992	ENSG00000261195.1	IQCK-AS1	105371115	-2.744439681	2.13E-18	3.86E-17
993	ENSG00000155324.10	GRAMD2B	65983	-1.118770986	2.14E-18	3.86E-17
994	ENSG00000099204.20	ABLIM1	3983	-1.316941687	2.22E-18	4.00E-17
995	ENSG00000253632.1		105379350	-2.70496268	2.34E-18	4.22E-17
996	ENSG00000138798.13	EGF	1950	-2.495945057	2.36E-18	4.25E-17
997	ENSG00000225549.3	SELENOKP3		-3.261659563	2.48E-18	4.44E-17
998	ENSG00000225398.3	PGM5P4		-2.588066397	2.50E-18	4.48E-17
999	ENSG00000115290.10	GRB14	2888	-2.485351679	2.52E-18	4.51E-17
1000	ENSG00000187486.5	KCNJ11	3767	-2.460277983	2.52E-18	4.52E-17
1001	ENSG00000287401.1			-2.271568668	2.59E-18	4.63E-17
1002	ENSG00000162543.6	UBXN10	127733	-2.349550139	2.76E-18	4.93E-17
1003	ENSG00000148541.13	FAM13C	220965	-2.057595631	2.76E-18	4.93E-17
1004	ENSG00000257052.1	PRPF19-DT		-1.413626914	2.97E-18	5.29E-17
1005	ENSG00000233850.1	MAL-AS1		-3.429077139	3.02E-18	5.37E-17
1006	ENSG00000250958.1	LINC00492	100861468	-2.68970561	3.09E-18	5.48E-17
1007	ENSG00000224307.2	LINC02975		-1.981523862	3.11E-18	5.52E-17
1008	ENSG00000115252.18	PDE1A	5136	-1.55906358	3.18E-18	5.64E-17
1009	ENSG00000071282.12	LMCD1	29995	-1.546076666	3.19E-18	5.66E-17
1010	ENSG00000185482.8	STAC3	246329	-3.022256783	3.36E-18	5.94E-17
1011	ENSG00000143369.15	ECM1	1893	-1.94594584	3.41E-18	6.02E-17
1012	ENSG00000178538.10	CA8	767	-2.776792531	3.43E-18	6.07E-17
1013	ENSG00000131037.15	EPS8L1	54869	-1.838874459	3.46E-18	6.12E-17
1014	ENSG00000165698.16	SPACA9	11092	-1.164780516	3.61E-18	6.36E-17
1015	ENSG00000021355.13	SERPINB1	1992	-1.616792434	3.64E-18	6.42E-17
1016	ENSG00000135363.12	LMO2	4005	-1.242272036	3.71E-18	6.53E-17
1017	ENSG00000261616.1	TTC23-AS1	128193289	-1.571548815	3.73E-18	6.57E-17
1018	ENSG00000211452.11	DIO1	1733	-2.110412844	4.01E-18	7.04E-17
1019	ENSG00000248309.7	MEF2C-AS1	101929423	-2.062554386	4.21E-18	7.39E-17
1020	ENSG00000204099.12	NEU4	129807	-2.787175383	4.24E-18	7.43E-17
1021	ENSG00000203943.9	SAMD13	148418	-1.658421378	4.30E-18	7.54E-17
1022	ENSG00000156042.18	CFAP70	118491	-1.590008829	4.32E-18	7.57E-17
1023	ENSG00000078328.22	RBFOX1	54715	-3.611471376	4.32E-18	7.57E-17
1024	ENSG00000248527.1	MTATP6P1		-1.194068016	4.33E-18	7.58E-17
1025	ENSG00000198892.7	SHISA4	149345	-1.833614648	4.38E-18	7.66E-17
1026	ENSG00000143994.14	ABHD1	84696	-1.983134139	4.60E-18	8.02E-17
1027	ENSG00000095585.17	BLNK	29760	-1.586640515	4.61E-18	8.04E-17
1028	ENSG00000143001.5	TMEM61	199964	-2.124233127	4.84E-18	8.42E-17
1029	ENSG00000123570.4	RAB9B	51209	-2.114763793	5.17E-18	8.97E-17
1030	ENSG00000151789.12	ZNF385D	79750	-1.942532022	5.24E-18	9.09E-17
1031	ENSG00000251320.1			-3.007341876	5.38E-18	9.34E-17
1032	ENSG00000108823.16	SGCA	6442	-3.241539913	5.96E-18	1.03E-16
1033	ENSG00000258249.1			-3.14767396	6.01E-18	1.04E-16
1034	ENSG00000146374.14	RSPO3	84870	-2.108558574	6.02E-18	1.04E-16
1035	ENSG00000285973.2			-2.373179672	6.21E-18	1.07E-16
1036	ENSG00000136717.15	BIN1	274	-1.809768385	6.36E-18	1.10E-16
1037	ENSG00000136694.9	IL36A	27179	-3.856332041	6.39E-18	1.10E-16
1038	ENSG00000143869.7	GDF7	151449	-1.975787357	6.40E-18	1.10E-16
1039	ENSG00000065361.16	ERBB3	2065	-1.140015463	6.53E-18	1.12E-16
1040	ENSG00000124939.6	SCGB2A1	4246	-3.880506861	6.75E-18	1.16E-16
1041	ENSG00000205364.4	MT1M	4499	-2.235994362	6.79E-18	1.16E-16
1042	ENSG00000164385.9	LINC01600	154386	-1.852651369	6.95E-18	1.19E-16
1043	ENSG00000218416.4	GPC1-AS1	100130449	-2.490060407	7.24E-18	1.24E-16
1044	ENSG00000120738.8	EGR1	1958	-1.563433736	7.45E-18	1.28E-16
1045	ENSG00000286952.1			-2.879340801	7.62E-18	1.30E-16
1046	ENSG00000268297.1	CLEC4GP1		-2.765210861	8.43E-18	1.44E-16
1047	ENSG00000265215.1	MIR4269	100423043	-4.122552247	8.44E-18	1.44E-16
1048	ENSG00000069011.16	PITX1	5307	-1.549980103	8.53E-18	1.45E-16
1049	ENSG00000100146.18	SOX10	6663	-3.657152635	8.57E-18	1.46E-16
1050	ENSG00000241479.1	MECOM-AS1	105374205	-2.533196985	8.86E-18	1.51E-16
1051	ENSG00000124664.11	SPDEF	25803	-3.373828694	8.97E-18	1.52E-16
1052	ENSG00000140538.16	NTRK3	4916	-2.637256253	9.89E-18	1.67E-16
1053	ENSG00000163209.15	SPRR3	6707	-3.463487904	1.05E-17	1.77E-16
1054	ENSG00000212901.4	KRTAP3-1	83896	-5.505854762	1.10E-17	1.86E-16
1055	ENSG00000180535.4	BHLHA15	168620	-2.41418628	1.11E-17	1.87E-16
1056	ENSG00000073910.22	FRY	10129	-1.721649921	1.16E-17	1.95E-16
1057	ENSG00000124215.17	CDH26	60437	-2.197514569	1.22E-17	2.04E-16
1058	ENSG00000226306.6	NPY6R		-4.392732878	1.32E-17	2.21E-16
1059	ENSG00000185156.6	MFSD6L	162387	-2.695506076	1.32E-17	2.21E-16
1060	ENSG00000162398.11	CIMAP2	163747	-2.341981334	1.33E-17	2.23E-16
1061	ENSG00000108984.15	MAP2K6	5608	-1.556048614	1.36E-17	2.28E-16
1062	ENSG00000126838.10	PZP	5858	-2.237624974	1.46E-17	2.43E-16
1063	ENSG00000166313.19	APBB1	322	-1.455176413	1.53E-17	2.54E-16
1064	ENSG00000270112.4	ST8SIA5-DT	105372097	-2.057381381	1.54E-17	2.56E-16
1065	ENSG00000135222.6	CSN2	1447	-5.655484602	1.60E-17	2.66E-16
1066	ENSG00000185010.15	F8	2157	-1.214607043	1.61E-17	2.68E-16
1067	ENSG00000283662.1			-3.362415179	1.61E-17	2.68E-16
1068	ENSG00000198899.2	MT-ATP6	4508	-1.122521077	1.73E-17	2.86E-16
1069	ENSG00000237560.7	LINC01497		-4.649231295	1.77E-17	2.92E-16
1070	ENSG00000166317.12	SYNPO2L	79933	-3.198630297	1.77E-17	2.92E-16
1071	ENSG00000231367.6	LINC02613	101929596	-2.058637393	1.80E-17	2.97E-16
1072	ENSG00000167653.5	PSCA	8000	-2.954280646	1.81E-17	2.99E-16
1073	ENSG00000232079.7	LINC01697	284825	-3.416737681	1.81E-17	2.99E-16
1074	ENSG00000143995.20	MEIS1	4211	-1.326807381	1.81E-17	2.99E-16
1075	ENSG00000178776.5	C5orf46	389336	-2.74255202	1.82E-17	3.01E-16
1076	ENSG00000178796.13	RIIAD1	284485	-3.303143205	1.83E-17	3.02E-16
1077	ENSG00000186198.4	SLC51B	123264	-1.779249787	1.83E-17	3.02E-16
1078	ENSG00000132326.12	PER2	8864	-1.114706564	1.90E-17	3.12E-16
1079	ENSG00000111912.20	NCOA7	135112	-1.184404087	1.96E-17	3.23E-16
1080	ENSG00000186442.7	KRT3	3850	-3.704594338	2.01E-17	3.30E-16
1081	ENSG00000119138.4	KLF9	687	-1.287685874	2.05E-17	3.37E-16
1082	ENSG00000286892.1			-3.521318505	2.11E-17	3.46E-16
1083	ENSG00000275120.3	ZSCAN2-AS1	105370947	-1.506893199	2.13E-17	3.49E-16
1084	ENSG00000123358.20	NR4A1	3164	-1.936164607	2.15E-17	3.52E-16
1085	ENSG00000155093.19	PTPRN2	5799	-1.729255406	2.17E-17	3.55E-16
1086	ENSG00000283787.2	PRR33	102724536	-1.932090892	2.18E-17	3.57E-16
1087	ENSG00000256751.6	PLBD1-AS1		-1.499629782	2.22E-17	3.63E-16
1088	ENSG00000283383.1		124900824	-2.337194745	2.26E-17	3.69E-16
1089	ENSG00000182795.13	C1orf116	79098	-1.512697223	2.27E-17	3.70E-16
1090	ENSG00000167183.3	PRR15L	79170	-2.487573457	2.31E-17	3.76E-16
1091	ENSG00000186976.15	EFCAB6	64800	-1.701046886	2.39E-17	3.89E-16
1092	ENSG00000179813.7	FAM216B	144809	-4.531418633	2.47E-17	4.02E-16
1093	ENSG00000039537.14	C6	729	-4.134671079	2.65E-17	4.30E-16
1094	ENSG00000188176.12	SMTNL2	342527	-3.331936019	2.92E-17	4.73E-16
1095	ENSG00000171819.5	ANGPTL7	10218	-3.270014859	3.07E-17	4.97E-16
1096	ENSG00000148377.6	IDI2	91734	-2.941794334	3.22E-17	5.20E-16
1097	ENSG00000087085.16	ACHE	43	-2.331481241	3.35E-17	5.40E-16
1098	ENSG00000134444.15	RELCH	57614	-1.003862903	3.37E-17	5.43E-16
1099	ENSG00000120729.10	MYOT	9499	-4.062622724	3.46E-17	5.57E-16
1100	ENSG00000148357.16	HMCN2	256158	-2.258631828	3.46E-17	5.57E-16
1101	ENSG00000176402.6	GJC3	349149	-2.456302202	3.47E-17	5.58E-16
1102	ENSG00000137727.12	ARHGAP20	57569	-1.832162479	3.48E-17	5.60E-16
1103	ENSG00000165887.12	ANKRD2	26287	-2.69671202	3.58E-17	5.75E-16
1104	ENSG00000267454.6	ZNF582-DT	386758	-1.687586116	3.65E-17	5.85E-16
1105	ENSG00000174428.18	GTF2IRD2B	389524	-1.089562225	4.13E-17	6.60E-16
1106	ENSG00000170324.21	FRMPD2	143162	-3.208453288	4.18E-17	6.67E-16
1107	ENSG00000204711.9	CFAP95	138255	-5.53906604	4.29E-17	6.85E-16
1108	ENSG00000101333.17	PLCB4	5332	-2.350091087	4.30E-17	6.86E-16
1109	ENSG00000230490.3			-1.990353952	4.34E-17	6.92E-16
1110	ENSG00000186806.5	VSIG10L	147645	-2.215227741	4.43E-17	7.06E-16
1111	ENSG00000229782.1	SPNS2-AS1		-2.557938675	4.58E-17	7.27E-16
1112	ENSG00000142661.19	MYOM3	127294	-2.568285475	4.79E-17	7.60E-16
1113	ENSG00000272502.1	SNTB1-AS1		-1.843864974	4.80E-17	7.63E-16
1114	ENSG00000250508.1	LINC02701		-2.432442649	4.85E-17	7.70E-16
1115	ENSG00000152763.17	DNAI4	79819	-1.551619541	5.19E-17	8.22E-16
1116	ENSG00000267365.1	KCNJ2-AS1	400617	-1.564459816	5.19E-17	8.22E-16
1117	ENSG00000287906.1			-1.648212125	5.50E-17	8.70E-16
1118	ENSG00000148735.15	PLEKHS1	79949	-3.401226096	5.55E-17	8.76E-16
1119	ENSG00000249631.6		107986178	-2.362494333	5.62E-17	8.86E-16
1120	ENSG00000170522.10	ELOVL6	79071	-1.319412535	5.75E-17	9.06E-16
1121	ENSG00000186231.17	KLHL32	114792	-2.144681832	5.76E-17	9.08E-16
1122	ENSG00000077684.16	JADE1	79960	-1.000367395	5.79E-17	9.11E-16
1123	ENSG00000188171.16	ZNF626	199777	-1.778791497	5.81E-17	9.14E-16
1124	ENSG00000134827.8	TCN1	6947	-3.117294212	5.85E-17	9.21E-16
1125	ENSG00000133317.16	LGALS12	85329	-2.802420526	5.98E-17	9.40E-16
1126	ENSG00000108551.5	RASD1	51655	-1.871822386	6.14E-17	9.64E-16
1127	ENSG00000171124.13	FUT3	2525	-2.043457556	6.21E-17	9.74E-16
1128	ENSG00000223382.5	LINC01778		-2.801170407	6.28E-17	9.84E-16
1129	ENSG00000168329.14	CX3CR1	1524	-1.924103842	6.71E-17	1.05E-15
1130	ENSG00000147394.18	ZNF185	7739	-1.571536193	6.74E-17	1.05E-15
1131	ENSG00000144908.14	ALDH1L1	10840	-2.791985533	6.81E-17	1.06E-15
1132	ENSG00000267259.1	ERVE-1	112267992	-1.807472549	6.90E-17	1.08E-15
1133	ENSG00000172831.14	CES2	8824	-1.253667223	7.14E-17	1.11E-15
1134	ENSG00000143375.15	CGN	57530	-1.705394923	7.15E-17	1.11E-15
1135	ENSG00000131471.7	AOC3	8639	-1.654502715	7.33E-17	1.14E-15
1136	ENSG00000117479.15	SLC19A2	10560	-1.030067339	7.57E-17	1.18E-15
1137	ENSG00000146151.14	HMGCLL1	54511	-2.945961309	7.81E-17	1.21E-15
1138	ENSG00000100219.16	XBP1	7494	-1.06425623	7.85E-17	1.22E-15
1139	ENSG00000070182.21	SPTB	6710	-2.818514682	7.86E-17	1.22E-15
1140	ENSG00000276791.1			-1.423543995	8.14E-17	1.26E-15
1141	ENSG00000173198.6	CYSLTR1	10800	-1.60894854	8.89E-17	1.37E-15
1142	ENSG00000259685.3	IDH2-DT		-2.3188326	9.01E-17	1.39E-15
1143	ENSG00000112874.10	NUDT12	83594	-1.001242644	9.09E-17	1.40E-15
1144	ENSG00000101938.15	CHRDL1	91851	-3.078686073	9.13E-17	1.40E-15
1145	ENSG00000081026.19	MAGI3	260425	-1.097982008	9.13E-17	1.40E-15
1146	ENSG00000128591.16	FLNC	2318	-2.76567173	9.93E-17	1.52E-15
1147	ENSG00000205221.12	VIT	5212	-2.412335717	1.03E-16	1.57E-15
1148	ENSG00000260372.7	AQP4-AS1		-3.018471379	1.05E-16	1.61E-15
1149	ENSG00000184650.11	ODF4	146852	-2.799572428	1.06E-16	1.61E-15
1150	ENSG00000235830.2	SRGAP3-AS4	101927440	-3.126542284	1.10E-16	1.68E-15
1151	ENSG00000185737.13	NRG3	10718	-3.024917614	1.11E-16	1.69E-15
1152	ENSG00000122971.9	ACADS	35	-1.035587792	1.14E-16	1.74E-15
1153	ENSG00000124839.13	RAB17	64284	-1.974842079	1.14E-16	1.74E-15
1154	ENSG00000233967.7			-1.775736933	1.16E-16	1.77E-15
1155	ENSG00000227621.1	PHB1P11		-2.400515885	1.18E-16	1.80E-15
1156	ENSG00000171209.3	CSN3	1448	-6.04327144	1.28E-16	1.94E-15
1157	ENSG00000184343.11	SRPK3	26576	-2.459800457	1.31E-16	1.98E-15
1158	ENSG00000151892.15	GFRA1	2674	-2.339799996	1.33E-16	2.01E-15
1159	ENSG00000007314.12	SCN4A	6329	-3.682096698	1.44E-16	2.16E-15
1160	ENSG00000227082.3	LINC02798	107985194	-2.324728668	1.48E-16	2.22E-15
1161	ENSG00000099260.11	PALMD	54873	-1.302147342	1.48E-16	2.23E-15
1162	ENSG00000186326.4	RGS9BP	388531	-1.739608398	1.53E-16	2.30E-15
1163	ENSG00000070526.15	ST6GALNAC1	55808	-2.262980651	1.64E-16	2.45E-15
1164	ENSG00000165084.16	C8orf34	116328	-1.933737028	1.65E-16	2.48E-15
1165	ENSG00000108576.10	SLC6A4	6532	-2.696810218	1.67E-16	2.50E-15
1166	ENSG00000150201.15	FXYD4	53828	-3.123717794	1.73E-16	2.59E-15
1167	ENSG00000143632.14	ACTA1	58	-4.318851487	1.75E-16	2.61E-15
1168	ENSG00000173991.6	TCAP	8557	-4.000776616	1.75E-16	2.62E-15
1169	ENSG00000133315.12	MACROD1	28992	-1.357742763	1.81E-16	2.70E-15
1170	ENSG00000115353.11	TACR1	6869	-2.360598603	1.83E-16	2.72E-15
1171	ENSG00000226819.1	MEIS1-AS3		-2.540146606	1.85E-16	2.76E-15
1172	ENSG00000177692.12	DNAJC28	54943	-1.006307162	1.87E-16	2.78E-15
1173	ENSG00000148225.16	WDR31	114987	-1.076346739	1.87E-16	2.79E-15
1174	ENSG00000171533.11	MAP6	4135	-2.068746281	1.96E-16	2.91E-15
1175	ENSG00000076685.19	NT5C2	22978	-1.126835573	1.99E-16	2.95E-15
1176	ENSG00000234899.11	SOX9-AS1	400618	-1.888919913	2.00E-16	2.96E-15
1177	ENSG00000153292.16	ADGRF1	266977	-2.581305109	2.00E-16	2.97E-15
1178	ENSG00000111261.14	MANSC1	54682	-1.563590811	2.02E-16	2.99E-15
1179	ENSG00000287124.1			-1.862710289	2.03E-16	3.01E-15
1180	ENSG00000272360.1			-1.780573547	2.05E-16	3.03E-15
1181	ENSG00000111834.13	RSPH4A	345895	-1.875995407	2.05E-16	3.03E-15
1182	ENSG00000118298.12	CA14	23632	-1.300420229	2.06E-16	3.04E-15
1183	ENSG00000261064.1	LINC02256	100996255	-1.350426516	2.06E-16	3.04E-15
1184	ENSG00000225472.2	NFIB-AS1	123706550	-3.042213568	2.20E-16	3.24E-15
1185	ENSG00000074855.11	ANO8	57719	-1.012515717	2.20E-16	3.25E-15
1186	ENSG00000285781.1			-2.959398804	2.21E-16	3.26E-15
1187	ENSG00000167034.10	NKX3-1	4824	-1.665908968	2.25E-16	3.31E-15
1188	ENSG00000258689.1	LINC01269		-2.269260278	2.29E-16	3.37E-15
1189	ENSG00000197776.8	KLHDC1	122773	-1.158228247	2.38E-16	3.50E-15
1190	ENSG00000197191.6	CYSRT1	375791	-2.403719962	2.44E-16	3.57E-15
1191	ENSG00000124237.6	CIMIP1	128602	-5.954674873	2.46E-16	3.60E-15
1192	ENSG00000269985.1	FARS2-AS1	101927972	-2.817879163	2.54E-16	3.71E-15
1193	ENSG00000224609.8	FGGY-DT		-1.42449167	2.54E-16	3.71E-15
1194	ENSG00000233834.6	GIRGL		-1.825475578	2.59E-16	3.78E-15
1195	ENSG00000285524.1			-5.155196165	2.80E-16	4.07E-15
1196	ENSG00000233342.2			-2.404692855	2.87E-16	4.17E-15
1197	ENSG00000169194.9	IL13	3596	-2.905283519	2.90E-16	4.21E-15
1198	ENSG00000231852.9	CYP21A2	1589	-2.51388109	3.04E-16	4.41E-15
1199	ENSG00000080166.16	DCT	1638	-4.388152068	3.07E-16	4.45E-15
1200	ENSG00000178878.13	APOLD1	81575	-1.122045331	3.15E-16	4.55E-15
1201	ENSG00000168491.10	CCDC110	256309	-1.769680244	3.19E-16	4.60E-15
1202	ENSG00000152583.12	SPARCL1	8404	-1.450903342	3.32E-16	4.79E-15
1203	ENSG00000162614.19	NEXN	91624	-2.046723122	3.34E-16	4.82E-15
1204	ENSG00000146221.10	TCTE1	202500	-2.719898589	3.56E-16	5.12E-15
1205	ENSG00000179639.10	FCER1A	2205	-2.151133258	3.65E-16	5.24E-15
1206	ENSG00000018869.17	ZNF582	147948	-1.511402875	3.77E-16	5.41E-15
1207	ENSG00000253115.1			-4.467497076	3.86E-16	5.53E-15
1208	ENSG00000188460.4	ACTBP11		-1.550345008	4.07E-16	5.84E-15
1209	ENSG00000233554.6	B4GALT1-AS1	101929639	-1.108837739	4.10E-16	5.87E-15
1210	ENSG00000266094.8	RASSF5	83593	-1.011927249	4.11E-16	5.88E-15
1211	ENSG00000149596.7	JPH2	57158	-2.454476774	4.14E-16	5.93E-15
1212	ENSG00000158113.13	LRRC43	254050	-1.869981746	4.16E-16	5.94E-15
1213	ENSG00000162069.16	BICDL2	146439	-1.733808959	4.22E-16	6.03E-15
1214	ENSG00000180481.11	GLIPR1L2	144321	-1.407255566	4.24E-16	6.05E-15
1215	ENSG00000036473.8	OTC	5009	-3.510352584	4.28E-16	6.10E-15
1216	ENSG00000230303.6			-2.176260786	4.28E-16	6.10E-15
1217	ENSG00000198763.3	MT-ND2	4536	-1.14537145	4.29E-16	6.11E-15
1218	ENSG00000126778.12	SIX1	6495	-1.477469615	4.32E-16	6.16E-15
1219	ENSG00000133878.9	DUSP26	78986	-3.337782451	4.35E-16	6.19E-15
1220	ENSG00000135111.16	TBX3	6926	-1.19722075	4.41E-16	6.27E-15
1221	ENSG00000257252.6			-1.880408928	4.43E-16	6.30E-15
1222	ENSG00000279317.2			-2.963754277	4.50E-16	6.39E-15
1223	ENSG00000287331.1			-2.593280461	4.53E-16	6.43E-15
1224	ENSG00000171766.17	GATM	2628	-1.757412093	4.54E-16	6.44E-15
1225	ENSG00000173612.10	GPRC6A	222545	-4.423964995	4.57E-16	6.49E-15
1226	ENSG00000274180.1	NATD1	256302	-1.052350005	4.73E-16	6.71E-15
1227	ENSG00000147573.17	TRIM55	84675	-3.097166816	4.84E-16	6.86E-15
1228	ENSG00000104611.12	SH2D4A	63898	-1.164258079	4.88E-16	6.91E-15
1229	ENSG00000250961.2	RMEL3		-3.038680028	4.90E-16	6.93E-15
1230	ENSG00000257017.9	HP	3240	-3.422996435	4.93E-16	6.98E-15
1231	ENSG00000250305.9	TRMT9B	57604	-2.057599307	5.00E-16	7.06E-15
1232	ENSG00000082269.17	FAM135A	57579	-1.182252844	5.00E-16	7.06E-15
1233	ENSG00000198938.2	MT-CO3	4514	-1.015753815	5.08E-16	7.18E-15
1234	ENSG00000236915.2	CLCA4-AS1	105378828	-2.307891007	5.09E-16	7.19E-15
1235	ENSG00000237975.7			-1.973886128	5.18E-16	7.31E-15
1236	ENSG00000232328.3			-2.035397771	5.41E-16	7.62E-15
1237	ENSG00000106992.19	AK1	203	-1.278911389	5.80E-16	8.14E-15
1238	ENSG00000091262.16	ABCC6	368	-1.604947379	5.85E-16	8.21E-15
1239	ENSG00000135472.9	FAIM2	23017	-1.794747768	6.29E-16	8.80E-15
1240	ENSG00000101134.12	DOK5	55816	-1.988401656	6.41E-16	8.97E-15
1241	ENSG00000234262.1			-3.136558693	6.47E-16	9.04E-15
1242	ENSG00000204385.13	SLC44A4	80736	-2.865946844	6.56E-16	9.15E-15
1243	ENSG00000230970.4	HHATL-AS1	100874044	-3.509324049	6.57E-16	9.17E-15
1244	ENSG00000183742.13	MACC1	346389	-1.769263832	6.98E-16	9.73E-15
1245	ENSG00000078114.19	NEBL	10529	-1.496189198	7.25E-16	1.01E-14
1246	ENSG00000154493.19	C10orf90	118611	-2.889606191	7.56E-16	1.05E-14
1247	ENSG00000029534.21	ANK1	286	-2.166145463	7.59E-16	1.05E-14
1248	ENSG00000235122.4			-3.971473645	7.62E-16	1.06E-14
1249	ENSG00000183549.10	ACSM5	54988	-2.106833595	7.65E-16	1.06E-14
1250	ENSG00000102409.10	BEX4	56271	-1.617578098	7.66E-16	1.06E-14
1251	ENSG00000163380.16	LMOD3	56203	-3.431126965	7.72E-16	1.07E-14
1252	ENSG00000236091.1	LINC02243	340581	-2.066935756	7.80E-16	1.08E-14
1253	ENSG00000237276.8	ANO7L1		-1.258261238	7.81E-16	1.08E-14
1254	ENSG00000286322.1		105376020	-3.652196594	7.96E-16	1.10E-14
1255	ENSG00000262823.1			-1.651102338	8.05E-16	1.11E-14
1256	ENSG00000151376.17	ME3	10873	-1.466474938	8.11E-16	1.12E-14
1257	ENSG00000141337.12	ARSG	22901	-1.00422273	8.25E-16	1.14E-14
1258	ENSG00000170915.9	PAQR8	85315	-1.389063801	8.42E-16	1.16E-14
1259	ENSG00000253807.6	LINC01170		-2.287077436	8.57E-16	1.18E-14
1260	ENSG00000114854.8	TNNC1	7134	-3.759111459	9.07E-16	1.25E-14
1261	ENSG00000215475.5	SIAH3	283514	-3.024634328	9.12E-16	1.26E-14
1262	ENSG00000140403.12	DNAJA4	55466	-1.061236693	9.19E-16	1.26E-14
1263	ENSG00000248050.1	SYBU-AS1		-2.996532308	9.25E-16	1.27E-14
1264	ENSG00000254109.6	RBPMS-AS1	100128750	-1.991552209	9.48E-16	1.30E-14
1265	ENSG00000153237.18	CCDC148	130940	-1.63907601	9.92E-16	1.36E-14
1266	ENSG00000236466.2	RCAN2-DT	101926898	-2.509414528	9.93E-16	1.36E-14
1267	ENSG00000258766.1	DIO2-AS1	100628307	-3.313461951	1.04E-15	1.42E-14
1268	ENSG00000198483.13	ANKRD35	148741	-1.949752127	1.04E-15	1.43E-14
1269	ENSG00000112183.15	RBM24	221662	-2.856033123	1.11E-15	1.51E-14
1270	ENSG00000112379.9	ARFGEF3	57221	-2.267376365	1.11E-15	1.51E-14
1271	ENSG00000286272.1			-3.412673245	1.17E-15	1.59E-14
1272	ENSG00000104936.19	DMPK	1760	-1.40418616	1.18E-15	1.61E-14
1273	ENSG00000125409.13	TEKT3	64518	-1.936026315	1.20E-15	1.63E-14
1274	ENSG00000071073.13	MGAT4A	11320	-1.227424416	1.29E-15	1.75E-14
1275	ENSG00000117643.14	MAN1C1	57134	-1.371228488	1.31E-15	1.77E-14
1276	ENSG00000103742.12	IGDCC4	57722	-1.815782728	1.32E-15	1.79E-14
1277	ENSG00000225670.4	CADM3-AS1		-2.35409981	1.32E-15	1.79E-14
1278	ENSG00000181092.10	ADIPOQ	9370	-5.766617281	1.36E-15	1.84E-14
1279	ENSG00000233760.2	LINC03095	100506289	-2.524855593	1.38E-15	1.86E-14
1280	ENSG00000267690.1	LDLRAD4-AS1	100288122	-2.474758069	1.40E-15	1.89E-14
1281	ENSG00000092009.10	CMA1	1215	-2.599795875	1.42E-15	1.91E-14
1282	ENSG00000145687.16	SSBP2	23635	-1.113065456	1.43E-15	1.93E-14
1283	ENSG00000275178.1			-2.175951863	1.49E-15	2.00E-14
1284	ENSG00000156966.7	B3GNT7	93010	-1.629497167	1.53E-15	2.05E-14
1285	ENSG00000167315.18	ACAA2	10449	-1.28808682	1.54E-15	2.07E-14
1286	ENSG00000261664.5	TTC39A-AS1		-2.284443449	1.56E-15	2.09E-14
1287	ENSG00000152154.11	TMEM178A	130733	-2.379851909	1.74E-15	2.34E-14
1288	ENSG00000231412.2	LINC03078	128193286	-2.628534115	1.78E-15	2.38E-14
1289	ENSG00000163406.11	SLC15A2	6565	-1.676123043	1.78E-15	2.39E-14
1290	ENSG00000154258.17	ABCA9	10350	-1.739999686	1.81E-15	2.42E-14
1291	ENSG00000231062.1			-3.95896948	1.82E-15	2.43E-14
1292	ENSG00000107859.10	PITX3	5309	-2.010084012	1.82E-15	2.43E-14
1293	ENSG00000224186.8	PITX1-AS1	100996485	-1.310201117	1.84E-15	2.45E-14
1294	ENSG00000238078.1	LINC01352	101929730	-2.07665925	1.89E-15	2.52E-14
1295	ENSG00000204396.11	VWA7	80737	-1.393755758	1.89E-15	2.52E-14
1296	ENSG00000239474.7	KLHL41	10324	-4.025347021	1.90E-15	2.53E-14
1297	ENSG00000268355.1			-1.65451995	1.91E-15	2.54E-14
1298	ENSG00000235652.9	EPM2A-DT		-1.058877487	1.94E-15	2.58E-14
1299	ENSG00000167861.16	HID1	283987	-1.701802821	2.03E-15	2.69E-14
1300	ENSG00000163632.14	C3orf49	132200	-1.096849694	2.10E-15	2.78E-14
1301	ENSG00000130413.15	STK33	65975	-2.399812712	2.13E-15	2.82E-14
1302	ENSG00000079435.10	LIPE	3991	-1.435848995	2.14E-15	2.83E-14
1303	ENSG00000207291.1	RNU6-30P		-2.009521645	2.14E-15	2.83E-14
1304	ENSG00000103310.11	ZP2	7783	-3.074560554	2.14E-15	2.83E-14
1305	ENSG00000188906.16	LRRK2	120892	-1.604371235	2.16E-15	2.85E-14
1306	ENSG00000232882.1	PHKA1P1		-1.276209994	2.18E-15	2.87E-14
1307	ENSG00000006788.14	MYH13	8735	-2.195458447	2.22E-15	2.93E-14
1308	ENSG00000163995.21	ABLIM2	84448	-1.60252168	2.28E-15	3.01E-14
1309	ENSG00000144451.19	SPAG16	79582	-1.256747188	2.32E-15	3.05E-14
1310	ENSG00000159239.13			-2.210271593	2.32E-15	3.05E-14
1311	ENSG00000162817.7	C1orf115	79762	-1.487354524	2.34E-15	3.08E-14
1312	ENSG00000139874.6	SSTR1	6751	-2.463243869	2.55E-15	3.36E-14
1313	ENSG00000111452.13	ADGRD1	283383	-1.892020486	2.61E-15	3.43E-14
1314	ENSG00000136160.17	EDNRB	1910	-1.454617633	2.70E-15	3.55E-14
1315	ENSG00000117425.14	PTCH2	8643	-1.652574546	2.71E-15	3.56E-14
1316	ENSG00000077063.11	CTTNBP2	83992	-2.298829735	2.71E-15	3.56E-14
1317	ENSG00000078814.17	MYH7B	57644	-2.265583342	2.72E-15	3.56E-14
1318	ENSG00000134780.10	DAGLA	747	-1.566689842	2.72E-15	3.57E-14
1319	ENSG00000270332.2	SMC2-DT	101928550	-2.011979759	2.73E-15	3.58E-14
1320	ENSG00000188175.10	HEPACAM2	253012	-3.329194761	2.88E-15	3.76E-14
1321	ENSG00000286289.1		107984270	-2.079897806	2.93E-15	3.83E-14
1322	ENSG00000261101.2			-1.745755967	2.97E-15	3.87E-14
1323	ENSG00000132141.14	CCT6B	10693	-1.105647852	3.28E-15	4.27E-14
1324	ENSG00000140527.15	WDR93	56964	-2.13752201	3.32E-15	4.31E-14
1325	ENSG00000263429.3	TMEM238L	100289255	-3.248456041	3.36E-15	4.36E-14
1326	ENSG00000130595.19	TNNT3	7140	-3.303090936	3.37E-15	4.38E-14
1327	ENSG00000203280.4	KIAA1671-AS1	100128531	-2.735810903	3.38E-15	4.39E-14
1328	ENSG00000127528.6	KLF2	10365	-1.270891616	3.45E-15	4.48E-14
1329	ENSG00000170954.11	ZNF415	55786	-1.688377628	3.55E-15	4.61E-14
1330	ENSG00000140285.12	FGF7	2252	-2.118915758	3.58E-15	4.65E-14
1331	ENSG00000280073.1			-2.888775225	3.59E-15	4.65E-14
1332	ENSG00000271952.2	LINC01954	101929733	-2.786760349	3.63E-15	4.70E-14
1333	ENSG00000203797.12	DDO	8528	-1.755079514	3.73E-15	4.83E-14
1334	ENSG00000266964.6	FXYD1	5348	-2.894035048	4.09E-15	5.29E-14
1335	ENSG00000169550.14	MUC15	143662	-2.294193429	4.25E-15	5.48E-14
1336	ENSG00000182557.8	SPNS3	201305	-1.518041817	4.25E-15	5.49E-14
1337	ENSG00000187758.8	ADH1A	124	-2.750143429	4.28E-15	5.52E-14
1338	ENSG00000053438.11	NNAT	4826	-1.698432681	4.42E-15	5.69E-14
1339	ENSG00000225630.1	MTND2P28		-1.569625418	4.45E-15	5.72E-14
1340	ENSG00000204889.10	KRT40	125115	-3.594253639	4.56E-15	5.86E-14
1341	ENSG00000130528.12	HRC	3270	-3.135315708	4.56E-15	5.86E-14
1342	ENSG00000278576.1			-1.102120056	4.65E-15	5.97E-14
1343	ENSG00000102683.8	SGCG	6445	-3.108614582	4.69E-15	6.02E-14
1344	ENSG00000196091.15	MYBPC1	4604	-4.264497119	4.77E-15	6.12E-14
1345	ENSG00000181778.5	TMEM252	169693	-2.563630201	5.00E-15	6.40E-14
1346	ENSG00000179178.11	TMEM125	128218	-1.673863331	5.08E-15	6.50E-14
1347	ENSG00000206113.11	CFAP99	402160	-1.83440635	5.19E-15	6.63E-14
1348	ENSG00000107968.10	MAP3K8	1326	-1.074636095	5.22E-15	6.67E-14
1349	ENSG00000213065.2			-3.001626843	5.52E-15	7.04E-14
1350	ENSG00000152931.9	PART1	25859	-2.379972722	5.56E-15	7.08E-14
1351	ENSG00000283213.1			-2.891982871	5.90E-15	7.49E-14
1352	ENSG00000259275.4			-1.500275923	5.92E-15	7.52E-14
1353	ENSG00000123560.14	PLP1	5354	-2.794788196	6.23E-15	7.91E-14
1354	ENSG00000224746.2			-1.407578174	6.36E-15	8.06E-14
1355	ENSG00000258987.2			-2.095809777	6.36E-15	8.06E-14
1356	ENSG00000086289.12	EPDR1	54749	-1.867829909	6.43E-15	8.14E-14
1357	ENSG00000237489.5	C10orf143	387723	-1.062709602	6.47E-15	8.18E-14
1358	ENSG00000165929.13	TC2N	123036	-1.269051793	6.47E-15	8.18E-14
1359	ENSG00000181754.7	AMIGO1	57463	-1.456042853	6.54E-15	8.27E-14
1360	ENSG00000186439.14	TRDN	10345	-4.151210292	7.04E-15	8.88E-14
1361	ENSG00000174844.14	DNAH12	201625	-2.303015618	7.10E-15	8.95E-14
1362	ENSG00000171811.14	CFAP46	54777	-2.402678758	7.24E-15	9.12E-14
1363	ENSG00000112116.10	IL17F	112744	-3.544118292	7.37E-15	9.28E-14
1364	ENSG00000188549.12	CCDC9B	388115	-1.480700527	7.44E-15	9.36E-14
1365	ENSG00000278408.1			-2.945572471	7.58E-15	9.53E-14
1366	ENSG00000238099.3	LINC01625	645434	-2.06933527	7.58E-15	9.53E-14
1367	ENSG00000070601.10	FRMPD1	22844	-2.344427052	7.77E-15	9.76E-14
1368	ENSG00000116039.13	ATP6V1B1	525	-2.784335111	7.80E-15	9.79E-14
1369	ENSG00000187583.11	PLEKHN1	84069	-1.236966077	8.35E-15	1.05E-13
1370	ENSG00000120129.6	DUSP1	1843	-1.434557193	8.44E-15	1.06E-13
1371	ENSG00000250041.3			-1.99163154	8.53E-15	1.07E-13
1372	ENSG00000136826.15	KLF4	9314	-1.36095993	8.71E-15	1.09E-13
1373	ENSG00000166535.20	A2ML1	144568	-2.279269362	8.95E-15	1.12E-13
1374	ENSG00000288088.1			-5.242297651	9.04E-15	1.13E-13
1375	ENSG00000173210.20	ABLIM3	22885	-1.439166608	9.13E-15	1.14E-13
1376	ENSG00000197822.11	OCLN	100506658	-1.713121252	9.32E-15	1.16E-13
1377	ENSG00000197616.13	MYH6	4624	-4.115468602	9.64E-15	1.20E-13
1378	ENSG00000154153.13	RETREG1	54463	-1.703340037	9.65E-15	1.20E-13
1379	ENSG00000166780.11	BMERB1	89927	-1.257480663	1.02E-14	1.27E-13
1380	ENSG00000168497.5	CAVIN2	8436	-1.798212481	1.03E-14	1.28E-13
1381	ENSG00000267603.1	LINC01028	101928141	-4.862215752	1.03E-14	1.28E-13
1382	ENSG00000170345.10	FOS	2353	-1.616948915	1.05E-14	1.30E-13
1383	ENSG00000156564.9	LRFN2	57497	-2.287791626	1.06E-14	1.31E-13
1384	ENSG00000074527.13	NTN4	59277	-1.279169296	1.09E-14	1.35E-13
1385	ENSG00000154175.18	ABI3BP	25890	-2.118702178	1.14E-14	1.41E-13
1386	ENSG00000162407.9	PLPP3	8613	-1.251499368	1.19E-14	1.47E-13
1387	ENSG00000215853.3	RPTN	126638	-3.288555269	1.24E-14	1.53E-13
1388	ENSG00000279022.1			-1.547162218	1.24E-14	1.53E-13
1389	ENSG00000230555.2	LINC02916		-1.123514155	1.27E-14	1.57E-13
1390	ENSG00000250790.4			-1.872720958	1.28E-14	1.58E-13
1391	ENSG00000174808.12	BTC	685	-1.96971823	1.31E-14	1.61E-13
1392	ENSG00000136059.14	VILL	50853	-1.309597892	1.31E-14	1.61E-13
1393	ENSG00000204947.9	ZNF425	155054	-1.045195591	1.35E-14	1.66E-13
1394	ENSG00000144366.16	GULP1	51454	-1.833585505	1.39E-14	1.70E-13
1395	ENSG00000170989.10	S1PR1	1901	-1.211077181	1.41E-14	1.72E-13
1396	ENSG00000122121.11	XPNPEP2	7512	-2.29245505	1.41E-14	1.73E-13
1397	ENSG00000237973.1	MTCO1P12		-1.196716335	1.45E-14	1.77E-13
1398	ENSG00000196275.14	GTF2IRD2	84163	-1.040213631	1.46E-14	1.79E-13
1399	ENSG00000122756.15	CNTFR	1271	-2.486776519	1.48E-14	1.80E-13
1400	ENSG00000117013.17	KCNQ4	9132	-1.686814078	1.50E-14	1.83E-13
1401	ENSG00000119703.15	ZC2HC1C	79696	-1.010644967	1.52E-14	1.85E-13
1402	ENSG00000130038.10	CRACR2A	84766	-1.475677953	1.58E-14	1.92E-13
1403	ENSG00000108924.14	HLF	3131	-2.146629234	1.58E-14	1.92E-13
1404	ENSG00000229828.2	PDE4DIPP1		-2.912067092	1.58E-14	1.93E-13
1405	ENSG00000259523.1			-1.047488823	1.63E-14	1.98E-13
1406	ENSG00000158014.15	SLC30A2	7780	-2.393164013	1.77E-14	2.14E-13
1407	ENSG00000229589.1	ACVR2B-AS1	100128640	-1.45389792	1.80E-14	2.17E-13
1408	ENSG00000198300.14	PEG3	5178	-2.768829661	1.81E-14	2.19E-13
1409	ENSG00000145703.16	IQGAP2	10788	-1.621392588	1.92E-14	2.32E-13
1410	ENSG00000236830.7	CBR3-AS1	100506428	-1.021573387	1.93E-14	2.32E-13
1411	ENSG00000127863.16	TNFRSF19	55504	-1.593649391	1.94E-14	2.34E-13
1412	ENSG00000246523.8	FZD4-DT	100506368	-1.411556686	2.04E-14	2.46E-13
1413	ENSG00000138615.6	CILP	8483	-2.674180747	2.05E-14	2.47E-13
1414	ENSG00000108018.15	SORCS1	114815	-2.395880604	2.07E-14	2.49E-13
1415	ENSG00000106018.14	VIPR2	7434	-2.261445461	2.09E-14	2.52E-13
1416	ENSG00000212907.2	MT-ND4L	4539	-1.207562585	2.11E-14	2.54E-13
1417	ENSG00000100678.19	SLC8A3	6547	-2.594182513	2.11E-14	2.54E-13
1418	ENSG00000260466.1	SLC7A5-AS1	124903753	-2.209096257	2.20E-14	2.64E-13
1419	ENSG00000152430.18	BOLL	66037	-1.993974843	2.23E-14	2.67E-13
1420	ENSG00000167046.4			-1.988715911	2.27E-14	2.71E-13
1421	ENSG00000286638.1			-1.849324746	2.34E-14	2.80E-13
1422	ENSG00000214970.8			-3.288157405	2.34E-14	2.81E-13
1423	ENSG00000217733.2	CCT7P1		-1.954150527	2.44E-14	2.91E-13
1424	ENSG00000198886.2	MT-ND4	4538	-1.002460683	2.45E-14	2.92E-13
1425	ENSG00000135298.14	ADGRB3	577	-2.120893227	2.45E-14	2.93E-13
1426	ENSG00000188582.10	PAQR9	344838	-2.841709396	2.50E-14	2.98E-13
1427	ENSG00000101353.15	MROH8	140699	-1.137502232	2.55E-14	3.03E-13
1428	ENSG00000135773.13	CAPN9	10753	-2.753823437	2.56E-14	3.05E-13
1429	ENSG00000154553.16	PDLIM3	27295	-2.424348881	2.58E-14	3.07E-13
1430	ENSG00000168356.13	SCN11A	11280	-1.827234174	2.64E-14	3.14E-13
1431	ENSG00000286962.1			-2.359516212	2.72E-14	3.23E-13
1432	ENSG00000241399.7	CD302	9936	-1.3485172	2.76E-14	3.27E-13
1433	ENSG00000182591.6	KRTAP11-1	337880	-5.456309892	2.78E-14	3.30E-13
1434	ENSG00000007306.15	CEACAM7	1087	-3.302941866	2.79E-14	3.30E-13
1435	ENSG00000230084.6	ZBTB47-AS1		-1.310379941	2.81E-14	3.33E-13
1436	ENSG00000283098.2			-2.344677484	2.83E-14	3.34E-13
1437	ENSG00000274370.1			-1.360880717	2.84E-14	3.36E-13
1438	ENSG00000253389.2			-4.526251754	2.99E-14	3.53E-13
1439	ENSG00000270059.1			-1.365178635	3.02E-14	3.57E-13
1440	ENSG00000188559.15	RALGAPA2	57186	-1.000979478	3.16E-14	3.73E-13
1441	ENSG00000285791.1			-2.347380936	3.23E-14	3.81E-13
1442	ENSG00000245466.1	TTC9-DT	101928075	-2.268816062	3.24E-14	3.82E-13
1443	ENSG00000154415.8	PPP1R3A	5506	-4.991076429	3.34E-14	3.93E-13
1444	ENSG00000231769.3			-3.047368604	3.37E-14	3.96E-13
1445	ENSG00000156885.6	COX6A2	1339	-4.364887028	3.44E-14	4.04E-13
1446	ENSG00000146521.10			-1.39098532	3.45E-14	4.05E-13
1447	ENSG00000169989.3	TIGD4	201798	-1.346857946	3.49E-14	4.09E-13
1448	ENSG00000186369.10	SERTAD4BP		-3.382580821	3.53E-14	4.14E-13
1449	ENSG00000150540.14	HNMT	3176	-1.247430298	3.58E-14	4.20E-13
1450	ENSG00000226519.2	LINC00390	79024	-1.652414595	3.64E-14	4.26E-13
1451	ENSG00000262188.2	LINC01978	101928738	-2.02187316	3.79E-14	4.43E-13
1452	ENSG00000283288.1	SMIM33	111064649	-3.146199968	3.79E-14	4.43E-13
1453	ENSG00000112619.8	PRPH2	5961	-1.753105418	3.82E-14	4.47E-13
1454	ENSG00000198513.13	ATL1	51062	-1.211955959	3.83E-14	4.47E-13
1455	ENSG00000214553.10	LRRC37A11P		-1.750786836	3.85E-14	4.49E-13
1456	ENSG00000105388.16	CEACAM5	1048	-2.680875277	4.00E-14	4.66E-13
1457	ENSG00000166959.8	MS4A8	83661	-5.090586846	4.03E-14	4.69E-13
1458	ENSG00000101213.7	PTK6	5753	-1.398482352	4.22E-14	4.90E-13
1459	ENSG00000268912.1			-1.096824035	4.23E-14	4.90E-13
1460	ENSG00000112214.10	FHL5	9457	-1.780511795	4.32E-14	5.01E-13
1461	ENSG00000106541.12	AGR2	10551	-2.436707671	4.35E-14	5.04E-13
1462	ENSG00000258231.5		100506869	-3.694537233	4.44E-14	5.14E-13
1463	ENSG00000235314.1	LINC00957	255031	-1.261068765	4.50E-14	5.21E-13
1464	ENSG00000225742.6			-2.336334655	4.81E-14	5.55E-13
1465	ENSG00000145358.6	DDIT4L	115265	-2.892502229	4.86E-14	5.60E-13
1466	ENSG00000115112.8	TFCP2L1	29842	-1.518005958	4.87E-14	5.62E-13
1467	ENSG00000213938.3	SEPHS1P6		-2.070088074	5.04E-14	5.80E-13
1468	ENSG00000187889.13	FYB2	199920	-2.804591214	5.15E-14	5.92E-13
1469	ENSG00000137501.17	SYTL2	54843	-1.387365241	5.32E-14	6.11E-13
1470	ENSG00000185915.5	KLHL34	257240	-2.831816432	5.43E-14	6.23E-13
1471	ENSG00000184414.2	IRS3P		-2.106725319	5.47E-14	6.27E-13
1472	ENSG00000064199.7	SPA17	53340	-1.013757663	5.49E-14	6.29E-13
1473	ENSG00000171817.17	ZNF540	163255	-1.313651491	5.56E-14	6.37E-13
1474	ENSG00000119411.11	BSPRY	54836	-1.672937741	5.57E-14	6.39E-13
1475	ENSG00000170962.13	PDGFD	80310	-1.624581093	5.70E-14	6.53E-13
1476	ENSG00000258752.2	FOXN3-AS3		-2.068941856	5.90E-14	6.75E-13
1477	ENSG00000168333.14	PPDPFL	492307	-5.101185241	5.92E-14	6.76E-13
1478	ENSG00000141161.12	UNC45B	146862	-3.452422538	6.01E-14	6.87E-13
1479	ENSG00000174059.17	CD34	947	-1.069006354	6.22E-14	7.10E-13
1480	ENSG00000165370.3	GPR101	83550	-4.444776594	6.29E-14	7.17E-13
1481	ENSG00000258413.2	FBXO34-AS1	105370509	-1.477695943	6.62E-14	7.54E-13
1482	ENSG00000228035.1	NGF-AS1	112840934	-2.682509805	6.88E-14	7.81E-13
1483	ENSG00000137857.17	DUOX1	53905	-1.274510056	7.07E-14	8.03E-13
1484	ENSG00000224621.1			-1.308907999	7.18E-14	8.15E-13
1485	ENSG00000144031.12	ANKRD53	79998	-1.413977319	7.26E-14	8.24E-13
1486	ENSG00000280202.1			-1.570151229	8.01E-14	9.04E-13
1487	ENSG00000079689.14	SCGN	10590	-3.982451392	8.05E-14	9.07E-13
1488	ENSG00000189334.9	S100A14	57402	-1.474791835	8.27E-14	9.32E-13
1489	ENSG00000185105.5	MYADML2	255275	-3.324971898	8.27E-14	9.32E-13
1490	ENSG00000230102.8	LINC02028	285389	-2.266375356	8.44E-14	9.50E-13
1491	ENSG00000102934.10	PLLP	51090	-1.346961044	8.45E-14	9.51E-13
1492	ENSG00000166343.10	MSS51	118490	-1.174826439	8.48E-14	9.53E-13
1493	ENSG00000168077.14	SCARA3	51435	-1.427617704	8.84E-14	9.93E-13
1494	ENSG00000134042.14	MRO	83876	-1.61065977	8.86E-14	9.94E-13
1495	ENSG00000070371.16	CLTCL1	8218	-1.286646546	9.29E-14	1.04E-12
1496	ENSG00000111860.14	CEP85L	387119	-1.052424496	9.30E-14	1.04E-12
1497	ENSG00000125999.11	BPIFB1	92747	-4.966573922	9.33E-14	1.04E-12
1498	ENSG00000159173.19	TNNI1	7135	-2.884042998	9.43E-14	1.06E-12
1499	ENSG00000245293.3	CYP2U1-AS1	101929595	-1.359876948	9.94E-14	1.11E-12
1500	ENSG00000229847.9	EMX2OS	196047	-1.968462362	9.96E-14	1.11E-12
1501	ENSG00000285424.1			-2.403044953	1.03E-13	1.15E-12
1502	ENSG00000256612.7	CYP2B7P		-2.927421633	1.04E-13	1.16E-12
1503	ENSG00000134917.10	ADAMTS8	11095	-1.921854206	1.08E-13	1.20E-12
1504	ENSG00000160678.12	S100A1	6271	-1.483279896	1.11E-13	1.24E-12
1505	ENSG00000069667.16	RORA	6095	-1.205495671	1.20E-13	1.33E-12
1506	ENSG00000107562.16	CXCL12	6387	-1.754338137	1.23E-13	1.36E-12
1507	ENSG00000169862.19	CTNND2	1501	-2.381843402	1.26E-13	1.40E-12
1508	ENSG00000153002.12	CPB1	1360	-2.620651707	1.30E-13	1.44E-12
1509	ENSG00000248362.1			-2.842976624	1.30E-13	1.44E-12
1510	ENSG00000278989.1			-1.559267664	1.30E-13	1.44E-12
1511	ENSG00000182816.9	KRTAP13-2	337959	-6.508145531	1.31E-13	1.45E-12
1512	ENSG00000158022.7	TRIM63	84676	-3.428531335	1.32E-13	1.46E-12
1513	ENSG00000224389.9	C4B	721	-1.952754534	1.40E-13	1.54E-12
1514	ENSG00000163141.20	BNIPL	149428	-1.68872198	1.40E-13	1.55E-12
1515	ENSG00000278934.1			-2.017402987	1.40E-13	1.55E-12
1516	ENSG00000166069.13	TMCO5A	145942	-3.904678807	1.41E-13	1.55E-12
1517	ENSG00000189377.9	CXCL17	284340	-2.303398031	1.41E-13	1.55E-12
1518	ENSG00000251027.2	LINC01950	102467213	-3.883181434	1.44E-13	1.58E-12
1519	ENSG00000267532.5	MIR497HG	100506713	-1.659876226	1.44E-13	1.59E-12
1520	ENSG00000275064.1	PDE4DIPP5		-3.821111888	1.48E-13	1.62E-12
1521	ENSG00000225731.1	PDE9A-AS1	101928284	-2.080565031	1.51E-13	1.66E-12
1522	ENSG00000125780.12	TGM3	7053	-3.859117774	1.53E-13	1.67E-12
1523	ENSG00000167434.10	CA4	762	-2.975087009	1.62E-13	1.77E-12
1524	ENSG00000101695.9	RNF125	54941	-1.188954602	1.62E-13	1.77E-12
1525	ENSG00000228541.1			-2.603220625	1.63E-13	1.78E-12
1526	ENSG00000240045.2	STRIT1	100507537	-4.832361833	1.69E-13	1.84E-12
1527	ENSG00000188505.5	NCCRP1	342897	-2.116985206	1.80E-13	1.96E-12
1528	ENSG00000105668.7	UPK1A	11045	-3.0791382	2.00E-13	2.17E-12
1529	ENSG00000167191.12	GPRC5B	51704	-1.515142695	2.05E-13	2.22E-12
1530	ENSG00000250510.8	GPR162	27239	-1.299906825	2.09E-13	2.27E-12
1531	ENSG00000111713.3	GYS2	2998	-4.206747499	2.11E-13	2.28E-12
1532	ENSG00000254988.1	ACER3-AS1		-2.458668292	2.12E-13	2.29E-12
1533	ENSG00000223254.1	Y_RNA		-1.369447487	2.14E-13	2.32E-12
1534	ENSG00000153904.21	DDAH1	23576	-1.342127683	2.16E-13	2.33E-12
1535	ENSG00000164106.8	SCRG1	11341	-2.476021997	2.20E-13	2.38E-12
1536	ENSG00000081189.16	MEF2C	4208	-1.845771931	2.20E-13	2.38E-12
1537	ENSG00000136732.16	GYPC	2995	-1.315696049	2.25E-13	2.42E-12
1538	ENSG00000161634.12	DCD	117159	-5.041531125	2.25E-13	2.43E-12
1539	ENSG00000163545.11	NUAK2	81788	-1.040887005	2.30E-13	2.47E-12
1540	ENSG00000183578.8	TNFAIP8L3	388121	-1.250150471	2.43E-13	2.61E-12
1541	ENSG00000112320.12	SOBP	55084	-1.52948345	2.47E-13	2.66E-12
1542	ENSG00000260541.1			-1.399155212	2.56E-13	2.75E-12
1543	ENSG00000157368.11	IL34	146433	-1.598025888	2.70E-13	2.90E-12
1544	ENSG00000206072.13	SERPINB11	89778	-3.114500371	2.85E-13	3.05E-12
1545	ENSG00000086548.9	CEACAM6	4680	-2.098122799	2.89E-13	3.09E-12
1546	ENSG00000204666.3	ZNF473CR	400710	-2.101491986	2.91E-13	3.11E-12
1547	ENSG00000210144.1	MT-TY		-1.437106266	2.94E-13	3.14E-12
1548	ENSG00000179270.7	PCARE	388939	-2.010891032	2.96E-13	3.15E-12
1549	ENSG00000148935.11	GAS2	2620	-1.475397779	2.97E-13	3.17E-12
1550	ENSG00000233725.7	NRAD1	121838	-4.103488198	2.99E-13	3.19E-12
1551	ENSG00000131831.18	RAI2	10742	-1.49259081	3.00E-13	3.20E-12
1552	ENSG00000205269.6	TMEM170B	100113407	-1.319360393	3.01E-13	3.21E-12
1553	ENSG00000146926.11	ASB10	136371	-4.212523112	3.02E-13	3.22E-12
1554	ENSG00000280255.1			-2.112471832	3.03E-13	3.23E-12
1555	ENSG00000157578.13	LCA5L	150082	-1.077146203	3.06E-13	3.25E-12
1556	ENSG00000169218.14	RSPO1	284654	-2.075904079	3.07E-13	3.26E-12
1557	ENSG00000244383.2	FAM3D-AS1	105377108	-2.255248776	3.14E-13	3.33E-12
1558	ENSG00000253844.1			-1.87818571	3.15E-13	3.34E-12
1559	ENSG00000272021.1			-2.504343919	3.15E-13	3.34E-12
1560	ENSG00000111319.13	SCNN1A	6337	-1.659722907	3.18E-13	3.37E-12
1561	ENSG00000242607.1	RPS3AP34		-1.890787976	3.19E-13	3.39E-12
1562	ENSG00000132698.15	RAB25	57111	-1.210659991	3.21E-13	3.40E-12
1563	ENSG00000173698.18	ADGRG2	10149	-1.961848136	3.26E-13	3.46E-12
1564	ENSG00000251586.1	TET2-AS1		-1.948941246	3.28E-13	3.47E-12
1565	ENSG00000102010.15	BMX	660	-1.585448966	3.32E-13	3.51E-12
1566	ENSG00000138135.7	CH25H	9023	-1.654494018	3.72E-13	3.92E-12
1567	ENSG00000115556.14	PLCD4	84812	-1.804843429	3.76E-13	3.96E-12
1568	ENSG00000229388.1	TAF12-DT		-1.132981206	3.84E-13	4.04E-12
1569	ENSG00000264345.2	LINC01894		-2.811700403	3.85E-13	4.05E-12
1570	ENSG00000223656.1	HMGB3P10		-1.265981685	3.86E-13	4.06E-12
1571	ENSG00000176092.16	CRYBG2	55057	-1.322965539	4.14E-13	4.34E-12
1572	ENSG00000149926.13	TLCD3B	83723	-2.429480433	4.25E-13	4.45E-12
1573	ENSG00000170807.12	LMOD2	442721	-4.189451552	4.54E-13	4.74E-12
1574	ENSG00000236208.1	C10orf71-AS1		-4.525921017	4.58E-13	4.78E-12
1575	ENSG00000180347.13	ITPRID1	223075	-3.625051856	4.63E-13	4.83E-12
1576	ENSG00000250511.1			-3.906317552	4.65E-13	4.84E-12
1577	ENSG00000133392.18	MYH11	4629	-2.247483211	4.69E-13	4.89E-12
1578	ENSG00000003147.19	ICA1	3382	-1.60103571	4.73E-13	4.93E-12
1579	ENSG00000128833.13	MYO5C	55930	-1.688557503	4.84E-13	5.04E-12
1580	ENSG00000118849.10	RARRES1	5918	-2.022279576	4.85E-13	5.04E-12
1581	ENSG00000236975.2	LINC02814	105373158	-2.603225737	4.85E-13	5.04E-12
1582	ENSG00000245468.4	LINC02447		-1.39470404	4.92E-13	5.11E-12
1583	ENSG00000124701.5	APOBEC2	10930	-3.365193384	4.93E-13	5.12E-12
1584	ENSG00000175984.15	DENND2C	163259	-1.111371461	4.94E-13	5.13E-12
1585	ENSG00000133454.16	MYO18B	84700	-2.958084177	4.96E-13	5.14E-12
1586	ENSG00000259078.2	PTBP1P		-1.55325408	5.04E-13	5.22E-12
1587	ENSG00000122420.10	PTGFR	5737	-1.931901396	5.04E-13	5.22E-12
1588	ENSG00000147256.12	ARHGAP36	158763	-3.000795748	5.05E-13	5.23E-12
1589	ENSG00000203857.10	HSD3B1	3283	-4.867180164	5.08E-13	5.26E-12
1590	ENSG00000100012.12	SEC14L3	266629	-2.601464245	5.08E-13	5.26E-12
1591	ENSG00000169083.18	AR	367	-1.996589977	5.24E-13	5.41E-12
1592	ENSG00000144061.14	NPHP1	4867	-1.133682782	5.32E-13	5.49E-12
1593	ENSG00000132677.13	RHBG	57127	-2.045394854	5.38E-13	5.55E-12
1594	ENSG00000268555.2			-2.744846418	5.45E-13	5.63E-12
1595	ENSG00000095110.8	NXPE1	120400	-2.600852632	5.49E-13	5.67E-12
1596	ENSG00000224078.15	SNHG14		-1.543923183	5.76E-13	5.92E-12
1597	ENSG00000164520.11	RAET1E	135250	-1.914866169	5.82E-13	5.98E-12
1598	ENSG00000100307.13	CBX7	23492	-1.149470842	5.82E-13	5.99E-12
1599	ENSG00000111245.17	MYL2	4633	-4.037024926	5.89E-13	6.06E-12
1600	ENSG00000081248.11	CACNA1S	779	-3.88818328	5.96E-13	6.11E-12
1601	ENSG00000165626.17	BEND7	222389	-1.714546913	5.99E-13	6.14E-12
1602	ENSG00000112936.19	C7	730	-2.952861252	6.40E-13	6.54E-12
1603	ENSG00000286864.1			-1.478132622	6.68E-13	6.82E-12
1604	ENSG00000162520.15	SYNC	81493	-1.58188554	6.79E-13	6.92E-12
1605	ENSG00000165995.22	CACNB2	783	-1.181805632	6.94E-13	7.07E-12
1606	ENSG00000165124.18	SVEP1	79987	-1.285606146	7.03E-13	7.16E-12
1607	ENSG00000182575.8	NXPH3	11248	-1.477005953	7.04E-13	7.16E-12
1608	ENSG00000149295.14	DRD2	1813	-2.189334605	7.13E-13	7.25E-12
1609	ENSG00000121552.4	CSTA	1475	-1.780005979	7.14E-13	7.26E-12
1610	ENSG00000260572.1	PPM1L-DT		-1.574587313	7.18E-13	7.30E-12
1611	ENSG00000284669.1			-1.305177716	7.19E-13	7.31E-12
1612	ENSG00000127249.15	ATP13A4	84239	-2.445052997	7.22E-13	7.33E-12
1613	ENSG00000144063.4	MALL	7851	-1.512951611	7.27E-13	7.38E-12
1614	ENSG00000137103.20	TMEM8B	51754	-1.226014348	7.60E-13	7.69E-12
1615	ENSG00000227260.2	LINC01985	101928015	-2.237826615	7.60E-13	7.69E-12
1616	ENSG00000154721.15	JAM2	58494	-1.28360005	8.06E-13	8.13E-12
1617	ENSG00000131044.17	TTLL9	164395	-1.521858292	8.37E-13	8.43E-12
1618	ENSG00000273682.1	BLVRBP1		-1.240579308	8.56E-13	8.61E-12
1619	ENSG00000164707.15	SLC13A4	26266	-1.733597941	8.60E-13	8.65E-12
1620	ENSG00000164675.11	IQUB	154865	-1.311924572	8.87E-13	8.90E-12
1621	ENSG00000183844.17	FAM3B	54097	-2.828476113	8.92E-13	8.95E-12
1622	ENSG00000164442.10	CITED2	10370	-1.082021422	8.98E-13	9.00E-12
1623	ENSG00000236155.6	ZPLD2P		-2.106650215	9.21E-13	9.23E-12
1624	ENSG00000006468.14	ETV1	2115	-1.60355631	9.33E-13	9.33E-12
1625	ENSG00000224023.10	EDRF1-DT	399821	-1.426085412	9.45E-13	9.45E-12
1626	ENSG00000197892.13	KIF13B	23303	-1.174839458	9.59E-13	9.58E-12
1627	ENSG00000181722.17	ZBTB20	26137	-1.316774265	9.62E-13	9.61E-12
1628	ENSG00000179388.9	EGR3	1960	-1.57149083	9.86E-13	9.85E-12
1629	ENSG00000156675.16	RAB11FIP1	80223	-1.47950057	9.89E-13	9.87E-12
1630	ENSG00000203709.12	MIR29B2CHG		-1.296098599	9.93E-13	9.91E-12
1631	ENSG00000115593.15	SMYD1	150572	-4.386111094	1.00E-12	9.99E-12
1632	ENSG00000111275.13	ALDH2	217	-1.089945055	1.01E-12	1.01E-11
1633	ENSG00000138347.16	MYPN	84665	-3.390776933	1.02E-12	1.01E-11
1634	ENSG00000065809.13	FAM107B	83641	-1.025349065	1.05E-12	1.05E-11
1635	ENSG00000078725.13	BRINP1	1620	-2.224786098	1.06E-12	1.06E-11
1636	ENSG00000170681.7	CAVIN4	347273	-2.513007547	1.08E-12	1.07E-11
1637	ENSG00000106688.12	SLC1A1	6505	-1.731373638	1.11E-12	1.11E-11
1638	ENSG00000154975.14	CA10	56934	-3.257736262	1.15E-12	1.14E-11
1639	ENSG00000284606.1			-1.914476375	1.16E-12	1.15E-11
1640	ENSG00000117791.16	MTARC2	54996	-1.297946107	1.17E-12	1.16E-11
1641	ENSG00000261175.6	LINC02188		-2.174034552	1.18E-12	1.17E-11
1642	ENSG00000123104.12	ITPR2	3709	-1.087893923	1.20E-12	1.19E-11
1643	ENSG00000156427.8	FGF18	8817	-1.823698672	1.28E-12	1.26E-11
1644	ENSG00000100448.4	CTSG	1511	-1.980926982	1.34E-12	1.32E-11
1645	ENSG00000214614.3			-3.462268891	1.34E-12	1.32E-11
1646	ENSG00000170153.11	RNF150	57484	-2.058026118	1.35E-12	1.33E-11
1647	ENSG00000130037.5	KCNA5	3741	-1.939788642	1.35E-12	1.33E-11
1648	ENSG00000168811.7	IL12A	3592	-1.756321396	1.36E-12	1.34E-11
1649	ENSG00000255774.2	LINC02747	105379407	-2.528038463	1.45E-12	1.43E-11
1650	ENSG00000077498.9	TYR	7299	-4.826997071	1.47E-12	1.44E-11
1651	ENSG00000262185.3	LINC02861	102724927	-1.076593005	1.51E-12	1.48E-11
1652	ENSG00000077522.14	ACTN2	88	-3.562486189	1.51E-12	1.48E-11
1653	ENSG00000230978.2	LINC00160	54064	-1.988579745	1.51E-12	1.48E-11
1654	ENSG00000140057.9	AK7	122481	-1.621672469	1.61E-12	1.57E-11
1655	ENSG00000286415.1			-3.16362369	1.61E-12	1.58E-11
1656	ENSG00000163293.12	NIPAL1	152519	-1.237911623	1.65E-12	1.61E-11
1657	ENSG00000121413.13	ZSCAN18	65982	-1.525169593	1.65E-12	1.61E-11
1658	ENSG00000250103.2	PANCR		-4.182221537	1.73E-12	1.69E-11
1659	ENSG00000186329.9	TMEM212	389177	-4.54404508	1.77E-12	1.72E-11
1660	ENSG00000154080.14	CHST9	83539	-3.615690806	1.79E-12	1.74E-11
1661	ENSG00000281769.1	LINC-ADAIN		-2.391546814	1.80E-12	1.75E-11
1662	ENSG00000230487.8	PSMG3-AS1	114796	-1.035107061	1.81E-12	1.75E-11
1663	ENSG00000124490.14	CRISP2	7180	-4.998924389	1.84E-12	1.78E-11
1664	ENSG00000166482.12	MFAP4	4239	-1.873088742	1.86E-12	1.80E-11
1665	ENSG00000197013.10	ZNF429	353088	-1.400896977	1.87E-12	1.81E-11
1666	ENSG00000163017.14	ACTG2	72	-1.922248694	1.87E-12	1.82E-11
1667	ENSG00000079101.16	CLUL1	27098	-2.294124937	1.89E-12	1.83E-11
1668	ENSG00000254528.7	FXYD6-AS1		-2.530800398	1.94E-12	1.87E-11
1669	ENSG00000165113.13	GKAP1	80318	-1.386858217	1.94E-12	1.87E-11
1670	ENSG00000173212.4	MAB21L3	126868	-1.886270926	2.04E-12	1.97E-11
1671	ENSG00000261888.1			-1.441732608	2.05E-12	1.98E-11
1672	ENSG00000154274.15	PGCKA1	55286	-2.278242383	2.06E-12	1.98E-11
1673	ENSG00000165816.13	VWA2	340706	-1.601265675	2.13E-12	2.05E-11
1674	ENSG00000137878.17	GCOM1	145781	-1.976201259	2.20E-12	2.11E-11
1675	ENSG00000205678.8	TECRL	253017	-4.226933187	2.22E-12	2.13E-11
1676	ENSG00000111339.12	ART4	420	-2.163541933	2.25E-12	2.16E-11
1677	ENSG00000135338.14	LCA5	167691	-1.121123903	2.26E-12	2.17E-11
1678	ENSG00000260402.1			-3.591775322	2.27E-12	2.18E-11
1679	ENSG00000151150.22	ANK3	288	-1.024646927	2.29E-12	2.19E-11
1680	ENSG00000226808.3	LINC00840	100506835	-1.614348475	2.35E-12	2.26E-11
1681	ENSG00000272482.1			-1.543435256	2.44E-12	2.33E-11
1682	ENSG00000251665.1			-3.588118547	2.49E-12	2.38E-11
1683	ENSG00000168509.20	HJV	148738	-4.18030802	2.53E-12	2.42E-11
1684	ENSG00000186496.12	ZNF396	252884	-1.146847829	2.55E-12	2.43E-11
1685	ENSG00000198521.12	ZNF43	7594	-1.35873793	2.56E-12	2.44E-11
1686	ENSG00000160588.10	MPZL3	196264	-1.115741965	2.61E-12	2.49E-11
1687	ENSG00000186479.5	RGS7BP	401190	-2.574620346	2.66E-12	2.54E-11
1688	ENSG00000215874.3	CAPNS1P1		-1.879094886	2.70E-12	2.57E-11
1689	ENSG00000173093.13	CCDC63	160762	-2.409712535	2.78E-12	2.64E-11
1690	ENSG00000168405.17	CMAHP		-1.326344904	2.78E-12	2.64E-11
1691	ENSG00000272398.6	CD24	100133941	-1.460111914	2.78E-12	2.64E-11
1692	ENSG00000119508.18	NR4A3	8013	-1.657829682	2.84E-12	2.69E-11
1693	ENSG00000257683.1	LINC02463		-2.995622197	2.85E-12	2.70E-11
1694	ENSG00000260871.1			-1.423898302	2.88E-12	2.73E-11
1695	ENSG00000215182.8	MUC5AC	4586	-4.264503128	2.91E-12	2.76E-11
1696	ENSG00000176293.20	ZNF135	7694	-1.538734107	2.93E-12	2.77E-11
1697	ENSG00000231943.9	PGM5P4-AS1	124906859	-2.375086997	2.97E-12	2.81E-11
1698	ENSG00000158055.16	GRHL3	57822	-1.509915838	2.98E-12	2.82E-11
1699	ENSG00000184292.7	TACSTD2	4070	-1.049036497	3.04E-12	2.87E-11
1700	ENSG00000162804.14	SNED1	25992	-1.464145016	3.13E-12	2.95E-11
1701	ENSG00000171236.10	LRG1	116844	-1.719707543	3.18E-12	2.99E-11
1702	ENSG00000183784.7	DOCK8-AS1		-1.503523591	3.20E-12	3.01E-11
1703	ENSG00000166920.13	C15orf48	84419	-1.503184184	3.29E-12	3.09E-11
1704	ENSG00000196263.8	ZNF471	57573	-1.63910977	3.29E-12	3.09E-11
1705	ENSG00000134201.12	GSTM5	2949	-1.736461408	3.37E-12	3.16E-11
1706	ENSG00000053918.18	KCNQ1	3784	-1.298422486	3.42E-12	3.21E-11
1707	ENSG00000261268.1	QKILA		-1.903092225	3.45E-12	3.23E-11
1708	ENSG00000167037.19	SGSM1	129049	-1.790515727	3.48E-12	3.26E-11
1709	ENSG00000179914.5	ITLN1	55600	-3.17142699	3.55E-12	3.33E-11
1710	ENSG00000269855.2	RNF225	646862	-1.932929422	3.63E-12	3.40E-11
1711	ENSG00000286615.1		105379199	-1.535062349	3.64E-12	3.41E-11
1712	ENSG00000173627.8	APOBEC4	403314	-4.706404815	3.66E-12	3.42E-11
1713	ENSG00000160188.10	RSPH1	89765	-1.996459674	3.83E-12	3.57E-11
1714	ENSG00000128313.2	APOL5	80831	-1.989330951	3.89E-12	3.63E-11
1715	ENSG00000228253.1	MT-ATP8	4509	-1.280459177	3.93E-12	3.66E-11
1716	ENSG00000237530.2			-3.298505311	4.01E-12	3.74E-11
1717	ENSG00000205363.6	INSYN1	388135	-1.790646501	4.01E-12	3.74E-11
1718	ENSG00000265374.1	LINC01908	105372040	-4.12008221	4.05E-12	3.77E-11
1719	ENSG00000211459.2	MT-RNR1		-1.14885776	4.10E-12	3.82E-11
1720	ENSG00000197177.15	ADGRA1	84435	-2.704609863	4.19E-12	3.89E-11
1721	ENSG00000082397.17	EPB41L3	23136	-1.209298998	4.21E-12	3.91E-11
1722	ENSG00000283199.3	C13orf46	100507747	-1.693306029	4.25E-12	3.95E-11
1723	ENSG00000284237.1	LINC02767	105372883	-1.508788435	4.26E-12	3.96E-11
1724	ENSG00000111837.12	MAK	4117	-1.376183628	4.30E-12	3.99E-11
1725	ENSG00000131771.14	PPP1R1B	84152	-3.0701707	4.36E-12	4.04E-11
1726	ENSG00000265345.1	MIR5188	100847004	-2.062318805	4.42E-12	4.10E-11
1727	ENSG00000168874.13	ATOH8	84913	-1.777326223	4.45E-12	4.13E-11
1728	ENSG00000157765.13	SLC34A2	10568	-3.224641627	4.76E-12	4.40E-11
1729	ENSG00000285928.1			-1.06018337	4.84E-12	4.47E-11
1730	ENSG00000134533.6	RERG	85004	-1.713006319	4.87E-12	4.50E-11
1731	ENSG00000125414.19	MYH2	4620	-4.503570536	4.91E-12	4.53E-11
1732	ENSG00000211448.13	DIO2	1734	-1.508448679	4.94E-12	4.56E-11
1733	ENSG00000261600.1		124906033	-2.573120728	4.98E-12	4.59E-11
1734	ENSG00000267968.1			-6.067004008	5.01E-12	4.62E-11
1735	ENSG00000004848.8	ARX	170302	-2.270316365	5.05E-12	4.65E-11
1736	ENSG00000286891.1			-2.854467358	5.19E-12	4.78E-11
1737	ENSG00000126895.15	AVPR2	554	-1.420226168	5.23E-12	4.81E-11
1738	ENSG00000255202.1			-1.755624445	5.29E-12	4.85E-11
1739	ENSG00000205502.4	C2CD4B	388125	-1.688173306	5.34E-12	4.90E-11
1740	ENSG00000228692.2			-2.32042718	5.35E-12	4.91E-11
1741	ENSG00000005471.19	ABCB4	5244	-1.266473609	5.76E-12	5.27E-11
1742	ENSG00000287629.1		124906232	-1.653842634	5.91E-12	5.40E-11
1743	ENSG00000168490.14	PHYHIP	9796	-1.781360843	6.01E-12	5.49E-11
1744	ENSG00000162383.13	SLC1A7	6512	-2.03239657	6.03E-12	5.51E-11
1745	ENSG00000172602.11	RND1	27289	-1.477039012	6.05E-12	5.52E-11
1746	ENSG00000078295.17	ADCY2	108	-2.288441082	6.09E-12	5.55E-11
1747	ENSG00000152578.13	GRIA4	2893	-2.472992011	6.17E-12	5.62E-11
1748	ENSG00000057657.17	PRDM1	639	-1.075670042	6.21E-12	5.65E-11
1749	ENSG00000224479.6	CDHR18P		-1.347402881	6.81E-12	6.17E-11
1750	ENSG00000160460.16	SPTBN4	57731	-1.507404367	7.04E-12	6.37E-11
1751	ENSG00000229453.2	SPINK8	646424	-3.687537183	7.15E-12	6.47E-11
1752	ENSG00000251620.3	STPG2-AS1	101410545	-3.364879872	7.25E-12	6.55E-11
1753	ENSG00000234175.1			-1.071208382	7.26E-12	6.56E-11
1754	ENSG00000249082.2	EPIST	101927953	-2.226101856	7.42E-12	6.70E-11
1755	ENSG00000184557.4	SOCS3	9021	-1.003371453	7.48E-12	6.75E-11
1756	ENSG00000255495.1			-1.304004392	7.55E-12	6.81E-11
1757	ENSG00000240225.10	ZNF542P		-1.328906595	7.77E-12	6.99E-11
1758	ENSG00000152467.10	ZSCAN1	284312	-2.10126612	7.81E-12	7.04E-11
1759	ENSG00000197520.10	FAM177B	400823	-1.276933828	7.90E-12	7.11E-11
1760	ENSG00000255085.8			-2.20336477	8.07E-12	7.25E-11
1761	ENSG00000273958.1			-1.746726136	8.19E-12	7.36E-11
1762	ENSG00000172403.11	SYNPO2	171024	-2.287761754	8.37E-12	7.51E-11
1763	ENSG00000105499.14	PLA2G4C	8605	-1.332366481	8.41E-12	7.55E-11
1764	ENSG00000173467.9	AGR3	155465	-3.644824096	8.69E-12	7.79E-11
1765	ENSG00000086967.10	MYBPC2	4606	-3.227190723	8.70E-12	7.80E-11
1766	ENSG00000240063.1	ATP11B-DT		-2.076586409	8.95E-12	8.00E-11
1767	ENSG00000268307.1	LINC02560	110806301	-1.792480991	9.09E-12	8.12E-11
1768	ENSG00000266904.6	LINC00663		-1.020307902	9.21E-12	8.22E-11
1769	ENSG00000167759.13	KLK13	26085	-2.404184763	9.25E-12	8.25E-11
1770	ENSG00000269313.5	MAGIX	79917	-1.040780435	9.49E-12	8.46E-11
1771	ENSG00000197893.14	NRAP	4892	-3.961463654	9.60E-12	8.55E-11
1772	ENSG00000165072.10	MAMDC2	256691	-1.811454276	1.02E-11	9.03E-11
1773	ENSG00000108244.17	KRT23	25984	-2.008889848	1.02E-11	9.07E-11
1774	ENSG00000088538.13	DOCK3	1795	-1.451094919	1.03E-11	9.17E-11
1775	ENSG00000163050.18	COQ8A	56997	-1.515666642	1.05E-11	9.33E-11
1776	ENSG00000256139.2			-1.57052297	1.09E-11	9.66E-11
1777	ENSG00000287373.1			-4.532614307	1.11E-11	9.80E-11
1778	ENSG00000287636.1		124901766	-3.824125376	1.13E-11	9.97E-11
1779	ENSG00000004809.14	SLC22A16	85413	-2.040941944	1.13E-11	9.97E-11
1780	ENSG00000272501.1			-1.153739328	1.13E-11	1.00E-10
1781	ENSG00000271474.1	UNC5C-AS1		-2.048408013	1.15E-11	1.01E-10
1782	ENSG00000229108.2	LINC02587	101927524	-2.348206086	1.16E-11	1.02E-10
1783	ENSG00000167476.10	JSRP1	126306	-2.050508393	1.16E-11	1.02E-10
1784	ENSG00000177752.14	YIPF7	285525	-3.366517069	1.22E-11	1.07E-10
1785	ENSG00000227243.3	SLAMF6P1		-3.154258318	1.29E-11	1.13E-10
1786	ENSG00000287792.1			-2.682851858	1.29E-11	1.13E-10
1787	ENSG00000167549.18	CORO6	84940	-2.033882319	1.31E-11	1.15E-10
1788	ENSG00000132464.13	ENAM	10117	-2.83283306	1.31E-11	1.15E-10
1789	ENSG00000173926.6	MARCHF3	115123	-1.100681123	1.31E-11	1.15E-10
1790	ENSG00000187510.10	PLEKHG7	440107	-2.179719727	1.33E-11	1.16E-10
1791	ENSG00000141579.7	ZNF750	79755	-1.344898346	1.37E-11	1.20E-10
1792	ENSG00000283674.3		729732	-1.77100043	1.38E-11	1.21E-10
1793	ENSG00000143882.12	ATP6V1C2	245973	-1.856967381	1.39E-11	1.21E-10
1794	ENSG00000261863.1	LINC01996		-3.329528625	1.39E-11	1.21E-10
1795	ENSG00000165591.6	FAAH2	158584	-1.172405439	1.41E-11	1.23E-10
1796	ENSG00000288674.1			-1.248309032	1.47E-11	1.28E-10
1797	ENSG00000187715.13	KBTBD12	166348	-3.057009451	1.50E-11	1.30E-10
1798	ENSG00000101230.6	ISM1	140862	-1.446208772	1.51E-11	1.31E-10
1799	ENSG00000260510.1			-1.705315342	1.52E-11	1.32E-10
1800	ENSG00000205667.3	ARSH	347527	-1.730986724	1.52E-11	1.32E-10
1801	ENSG00000279348.1			-1.080633984	1.53E-11	1.33E-10
1802	ENSG00000223783.1	LINC01983		-2.834509683	1.56E-11	1.35E-10
1803	ENSG00000080709.16	KCNN2	3781	-1.426918739	1.57E-11	1.36E-10
1804	ENSG00000170162.14	VGLL2	245806	-3.362492082	1.61E-11	1.40E-10
1805	ENSG00000183032.12	SLC25A21	89874	-1.394617858	1.66E-11	1.44E-10
1806	ENSG00000173641.18	HSPB7	27129	-2.911940197	1.68E-11	1.45E-10
1807	ENSG00000117834.13	SLC5A9	200010	-1.41899635	1.69E-11	1.46E-10
1808	ENSG00000151320.11	AKAP6	9472	-1.822711508	1.70E-11	1.46E-10
1809	ENSG00000163661.4	PTX3	5806	-2.147738545	1.72E-11	1.48E-10
1810	ENSG00000270733.1			-1.365269734	1.74E-11	1.50E-10
1811	ENSG00000287143.1			-3.086930927	1.77E-11	1.52E-10
1812	ENSG00000287270.1			-2.322770382	1.81E-11	1.56E-10
1813	ENSG00000107954.10	NEURL1	9148	-1.837965083	1.87E-11	1.61E-10
1814	ENSG00000239801.1			-1.513610213	1.89E-11	1.62E-10
1815	ENSG00000165300.7	SLITRK5	26050	-2.543506817	1.89E-11	1.62E-10
1816	ENSG00000166682.13	TMPRSS5	80975	-1.621106632	1.89E-11	1.62E-10
1817	ENSG00000259925.1			-3.075976874	1.92E-11	1.65E-10
1818	ENSG00000125740.14	FOSB	2354	-1.818886262	1.93E-11	1.65E-10
1819	ENSG00000163749.17	CCDC158	339965	-1.571832586	2.02E-11	1.73E-10
1820	ENSG00000133742.14	CA1	759	-2.280455648	2.05E-11	1.75E-10
1821	ENSG00000126233.2	SLURP1	57152	-2.749904267	2.11E-11	1.80E-10
1822	ENSG00000260339.1	HEXA-AS1	80072	-1.077639254	2.21E-11	1.88E-10
1823	ENSG00000170290.4	SLN	6588	-3.324497574	2.31E-11	1.96E-10
1824	ENSG00000183690.13	EFHC2	80258	-1.751256378	2.35E-11	2.00E-10
1825	ENSG00000231013.2	SCTR-AS1	107105282	-3.215005743	2.47E-11	2.09E-10
1826	ENSG00000215045.8	GRID2IP	392862	-1.318248823	2.47E-11	2.09E-10
1827	ENSG00000188517.16	COL25A1	84570	-2.024706842	2.52E-11	2.14E-10
1828	ENSG00000230442.5	ASB15-AS1	102724555	-3.652480011	2.53E-11	2.14E-10
1829	ENSG00000081853.15	PCDHGA2	56113	-1.37594678	2.62E-11	2.21E-10
1830	ENSG00000200201.1	Y_RNA		-4.691628496	2.64E-11	2.23E-10
1831	ENSG00000173376.14	NDNF	79625	-1.864502631	2.68E-11	2.26E-10
1832	ENSG00000204361.10	NXPE2	120406	-3.626950257	2.71E-11	2.29E-10
1833	ENSG00000184489.12	PTP4A3	11156	-1.272819125	2.87E-11	2.41E-10
1834	ENSG00000233170.4			-1.312341508	2.92E-11	2.46E-10
1835	ENSG00000170381.14	SEMA3E	9723	-2.174338523	2.93E-11	2.47E-10
1836	ENSG00000268833.1		112268252	-1.987554697	2.97E-11	2.49E-10
1837	ENSG00000138100.14	TRIM54	57159	-2.98913471	3.01E-11	2.53E-10
1838	ENSG00000287191.1			-2.444968955	3.14E-11	2.63E-10
1839	ENSG00000258818.4	RNASE4	6038	-1.344552416	3.42E-11	2.86E-10
1840	ENSG00000236829.9			-1.159974576	3.57E-11	2.98E-10
1841	ENSG00000176485.12	PLAAT3	11145	-1.56586374	3.59E-11	3.00E-10
1842	ENSG00000205847.6	OR7E91P		-1.633350544	3.60E-11	3.00E-10
1843	ENSG00000209082.1	MT-TL1		-1.773193364	3.61E-11	3.01E-10
1844	ENSG00000229028.2	KRT223P		-2.422760244	3.62E-11	3.02E-10
1845	ENSG00000132681.17	ATP1A4	480	-1.702899105	3.69E-11	3.08E-10
1846	ENSG00000205978.6	NYNRIN	57523	-1.28886947	3.71E-11	3.08E-10
1847	ENSG00000106809.11	OGN	4969	-2.373179272	3.74E-11	3.11E-10
1848	ENSG00000172425.12	TTC36	143941	-1.625602354	3.80E-11	3.16E-10
1849	ENSG00000137675.5	MMP27	64066	-2.080281844	3.83E-11	3.18E-10
1850	ENSG00000264424.1	MYH4	4622	-2.616995783	3.83E-11	3.18E-10
1851	ENSG00000182916.8	TCEAL7	56849	-1.499830611	3.86E-11	3.20E-10
1852	ENSG00000142606.16	MMEL1	79258	-1.6120717	3.86E-11	3.20E-10
1853	ENSG00000287362.1			-1.308863412	4.00E-11	3.31E-10
1854	ENSG00000103710.11	RASL12	51285	-1.070561515	4.10E-11	3.39E-10
1855	ENSG00000141052.18	MYOCD	93649	-1.706088007	4.18E-11	3.45E-10
1856	ENSG00000263004.2	AKAP1-DT	119139901	-1.074372426	4.25E-11	3.51E-10
1857	ENSG00000139910.20	NOVA1	4857	-1.725286991	4.26E-11	3.52E-10
1858	ENSG00000236311.6	TLX1NB	100038246	-2.717838644	4.28E-11	3.53E-10
1859	ENSG00000184227.8	ACOT1	641371	-1.070046906	4.30E-11	3.54E-10
1860	ENSG00000175591.12	P2RY2	5029	-1.174659688	4.40E-11	3.63E-10
1861	ENSG00000286043.1			-1.348889333	4.57E-11	3.76E-10
1862	ENSG00000234456.8	MAGI2-AS3	100505881	-1.246360432	4.60E-11	3.78E-10
1863	ENSG00000163435.16	ELF3	1999	-1.541108287	4.69E-11	3.85E-10
1864	ENSG00000142627.13	EPHA2	1969	-1.05170644	4.71E-11	3.87E-10
1865	ENSG00000167769.5	ACER1	125981	-2.544020181	4.74E-11	3.89E-10
1866	ENSG00000244731.8	C4A	720	-1.639317154	4.76E-11	3.91E-10
1867	ENSG00000257588.1	AQP5-AS1	101927318	-3.600234014	4.83E-11	3.96E-10
1868	ENSG00000185630.19	PBX1	5087	-1.429240409	4.84E-11	3.97E-10
1869	ENSG00000084734.9	GCKR	2646	-1.530121533	4.92E-11	4.03E-10
1870	ENSG00000277758.5	SYT15B	102724488	-1.439023345	4.93E-11	4.03E-10
1871	ENSG00000183273.7	CCDC60	160777	-3.153588887	5.05E-11	4.13E-10
1872	ENSG00000183631.5	PRR32	100130613	-3.463571634	5.29E-11	4.32E-10
1873	ENSG00000210140.1	MT-TC		-1.568913341	5.38E-11	4.39E-10
1874	ENSG00000287527.1			-1.459906407	5.46E-11	4.45E-10
1875	ENSG00000287003.1		107986482	-4.78637437	5.46E-11	4.45E-10
1876	ENSG00000226889.3	ATF6-DT		-1.340014107	5.48E-11	4.47E-10
1877	ENSG00000130433.7	CACNG6	59285	-3.055317266	5.53E-11	4.50E-10
1878	ENSG00000198168.9	SVIP	258010	-1.537689618	5.66E-11	4.61E-10
1879	ENSG00000254290.1			-2.824035053	5.73E-11	4.66E-10
1880	ENSG00000241316.8	SUCLG2-DT	101927111	-1.122480581	5.79E-11	4.70E-10
1881	ENSG00000082929.8	LINC01587	10141	-1.820479174	5.84E-11	4.74E-10
1882	ENSG00000270457.1			-1.916416653	5.85E-11	4.75E-10
1883	ENSG00000183571.11	PGPEP1L	145814	-2.333312686	5.92E-11	4.80E-10
1884	ENSG00000135424.18	ITGA7	3679	-1.603936905	5.95E-11	4.83E-10
1885	ENSG00000212864.3	RNF208	727800	-1.239610605	5.98E-11	4.85E-10
1886	ENSG00000169583.13	CLIC3	9022	-1.754426167	6.12E-11	4.96E-10
1887	ENSG00000244067.3	GSTA2	2939	-4.284261328	6.13E-11	4.96E-10
1888	ENSG00000188716.5	DUSP29	338599	-3.459022683	6.14E-11	4.96E-10
1889	ENSG00000252051.1	RN7SKP276		-3.690603118	6.26E-11	5.05E-10
1890	ENSG00000117477.12	CCDC181	57821	-1.194426883	6.27E-11	5.06E-10
1891	ENSG00000177694.16	NAALADL2	254827	-1.652220676	6.30E-11	5.08E-10
1892	ENSG00000274560.1			-2.690909028	6.32E-11	5.10E-10
1893	ENSG00000170935.8	NCBP2L	392517	-1.821504982	6.40E-11	5.15E-10
1894	ENSG00000127743.6	IL17B	27190	-1.473250286	6.40E-11	5.15E-10
1895	ENSG00000147862.17	NFIB	4781	-1.12179846	6.47E-11	5.20E-10
1896	ENSG00000255435.7	TTC36-AS1	101929089	-1.117267881	6.62E-11	5.32E-10
1897	ENSG00000106511.6	MEOX2	4223	-1.673226305	6.67E-11	5.36E-10
1898	ENSG00000089250.19	NOS1	4842	-2.14941395	6.74E-11	5.41E-10
1899	ENSG00000256870.3	SLC5A8	160728	-3.815631882	6.79E-11	5.45E-10
1900	ENSG00000248905.10	FMN1	342184	-1.112961613	6.85E-11	5.49E-10
1901	ENSG00000233622.3	CYP2T1P		-1.126278364	6.93E-11	5.55E-10
1902	ENSG00000114547.10	ROPN1B	152015	-2.214371577	7.01E-11	5.62E-10
1903	ENSG00000205038.12	PKHD1L1	93035	-1.936547594	7.15E-11	5.72E-10
1904	ENSG00000245330.4	DEPTOR-AS1		-1.759708654	7.16E-11	5.73E-10
1905	ENSG00000147027.4	TMEM47	83604	-1.235872077	7.19E-11	5.75E-10
1906	ENSG00000233117.4	LINC00702		-1.73809002	7.20E-11	5.75E-10
1907	ENSG00000229444.1	ST3GAL3-AS1	101929592	-2.904317105	7.20E-11	5.76E-10
1908	ENSG00000134317.18	GRHL1	29841	-1.277794015	7.21E-11	5.76E-10
1909	ENSG00000050628.20	PTGER3	5733	-1.615983957	7.23E-11	5.77E-10
1910	ENSG00000287148.1	LINCADL	107986443	-5.807248481	7.34E-11	5.86E-10
1911	ENSG00000279026.1			-1.135627616	7.38E-11	5.89E-10
1912	ENSG00000121310.17	ECHDC2	55268	-1.034872376	7.41E-11	5.90E-10
1913	ENSG00000181016.9	LSMEM1	286006	-1.311181402	7.44E-11	5.93E-10
1914	ENSG00000168350.8	DEGS2	123099	-1.757503439	7.47E-11	5.95E-10
1915	ENSG00000182329.14	KIAA2012	100652824	-1.66489485	7.49E-11	5.96E-10
1916	ENSG00000166840.13	GLYATL1	92292	-2.045713014	7.49E-11	5.97E-10
1917	ENSG00000245857.3	GS1-24F4.2	100652791	-1.796189035	7.59E-11	6.04E-10
1918	ENSG00000196136.18	SERPINA3	12	-2.403604689	7.66E-11	6.08E-10
1919	ENSG00000146352.13	CLVS2	134829	-3.03287979	7.73E-11	6.14E-10
1920	ENSG00000199899.2	Y_RNA		-2.416432077	7.73E-11	6.14E-10
1921	ENSG00000237311.3			-2.00421976	7.76E-11	6.16E-10
1922	ENSG00000266729.5	DSG1-AS1	101927718	-1.8018289	7.76E-11	6.16E-10
1923	ENSG00000245812.3	LINC02202	101927740	-1.219658012	7.84E-11	6.22E-10
1924	ENSG00000200253.1	RNU6-529P		-1.0583029	7.87E-11	6.24E-10
1925	ENSG00000104371.5	DKK4	27121	-2.411508605	7.99E-11	6.33E-10
1926	ENSG00000198515.16	CNGA1	1259	-1.282037679	8.04E-11	6.37E-10
1927	ENSG00000116882.15	HAO2	51179	-2.270106199	8.21E-11	6.50E-10
1928	ENSG00000187554.14	TLR5	7100	-1.104429145	8.22E-11	6.50E-10
1929	ENSG00000206422.2	LRRC30	339291	-4.228555377	8.36E-11	6.61E-10
1930	ENSG00000114248.10	LRRC31	79782	-3.478494114	8.44E-11	6.67E-10
1931	ENSG00000248801.7	C8orf34-AS1		-2.731145982	8.49E-11	6.71E-10
1932	ENSG00000081803.16	CADPS2	93664	-1.18398943	8.54E-11	6.75E-10
1933	ENSG00000108242.13	CYP2C18	1562	-1.916598115	8.64E-11	6.82E-10
1934	ENSG00000224957.6	LINC01266	101927215	-2.167548795	8.64E-11	6.82E-10
1935	ENSG00000114771.14	AADAC	13	-2.584822208	8.76E-11	6.91E-10
1936	ENSG00000259188.6		102724587	-2.183359072	8.79E-11	6.93E-10
1937	ENSG00000235263.1			-4.065445671	8.95E-11	7.05E-10
1938	ENSG00000246430.7	LINC00968	100507632	-1.544885546	8.98E-11	7.07E-10
1939	ENSG00000284977.2			-1.458293387	9.01E-11	7.10E-10
1940	ENSG00000135744.8	AGT	183	-1.928662746	9.04E-11	7.11E-10
1941	ENSG00000172987.13	HPSE2	60495	-2.265291749	9.42E-11	7.41E-10
1942	ENSG00000121440.15	PDZRN3	23024	-1.342531137	9.56E-11	7.51E-10
1943	ENSG00000185483.12	ROR1	4919	-1.447245064	9.71E-11	7.62E-10
1944	ENSG00000225342.2	LRRK2-DT	107985190	-1.503886255	9.73E-11	7.64E-10
1945	ENSG00000165507.9	DEPP1	11067	-1.281969721	9.91E-11	7.77E-10
1946	ENSG00000174403.16	CRMA		-2.452872749	1.00E-10	7.85E-10
1947	ENSG00000088899.15	LZTS3	9762	-1.196083096	1.01E-10	7.90E-10
1948	ENSG00000258274.1	CRADD-AS1	101928731	-1.86332516	1.02E-10	8.00E-10
1949	ENSG00000176909.12	MAMSTR	284358	-1.157839884	1.05E-10	8.18E-10
1950	ENSG00000179299.17	NSUN7	79730	-1.663029549	1.07E-10	8.34E-10
1951	ENSG00000197361.8	FBXL22	283807	-1.009535864	1.12E-10	8.77E-10
1952	ENSG00000075673.12	ATP12A	479	-2.558804008	1.13E-10	8.79E-10
1953	ENSG00000230790.2			-2.174110711	1.15E-10	8.94E-10
1954	ENSG00000234477.1			-1.952504663	1.15E-10	8.96E-10
1955	ENSG00000135374.11	ELF5	2001	-2.504276515	1.16E-10	9.03E-10
1956	ENSG00000177291.4	GJD4	219770	-2.10156589	1.17E-10	9.08E-10
1957	ENSG00000170500.13	LONRF2	164832	-2.296306241	1.17E-10	9.08E-10
1958	ENSG00000111536.5	IL26	55801	-1.880107909	1.19E-10	9.26E-10
1959	ENSG00000111344.11	RASAL1	8437	-1.272919085	1.19E-10	9.28E-10
1960	ENSG00000182676.5	PPP1R27	116729	-2.774947251	1.20E-10	9.30E-10
1961	ENSG00000247011.2			-2.753005413	1.20E-10	9.35E-10
1962	ENSG00000174233.11	ADCY6	112	-1.004420077	1.22E-10	9.45E-10
1963	ENSG00000287292.1			-3.035564425	1.23E-10	9.55E-10
1964	ENSG00000100399.16	CHADL	150356	-1.194223758	1.23E-10	9.57E-10
1965	ENSG00000141469.18	SLC14A1	6563	-1.509121973	1.25E-10	9.68E-10
1966	ENSG00000237413.5	MGC27382	149047	-2.225829188	1.25E-10	9.71E-10
1967	ENSG00000184254.17	ALDH1A3	220	-1.398012175	1.26E-10	9.77E-10
1968	ENSG00000186212.4	SOWAHB	345079	-1.407074264	1.27E-10	9.81E-10
1969	ENSG00000285802.1			-2.359207597	1.28E-10	9.93E-10
1970	ENSG00000248441.7	LETR1	400456	-1.128755647	1.29E-10	9.95E-10
1971	ENSG00000104055.16	TGM5	9333	-1.662907917	1.30E-10	1.0054E-09
1972	ENSG00000273487.1			-1.581245933	1.33E-10	1.02565E-09
1973	ENSG00000182983.15	ZNF662	389114	-1.463355125	1.34E-10	1.03085E-09
1974	ENSG00000204897.7	KRT25	147183	-2.773899844	1.39E-10	1.07159E-09
1975	ENSG00000182177.14	ASB18	401036	-2.43746014	1.47E-10	1.13282E-09
1976	ENSG00000115361.8	ACADL	33	-2.3338555	1.52E-10	1.16735E-09
1977	ENSG00000130517.14	PGPEP1	54858	-1.021054854	1.52E-10	1.16754E-09
1978	ENSG00000215223.3	CYP51A1P3		-1.889941395	1.56E-10	1.19328E-09
1979	ENSG00000177238.14	TRIM72	493829	-2.51681068	1.71E-10	1.31053E-09
1980	ENSG00000160180.15	TFF3	7033	-2.812954627	1.72E-10	1.31908E-09
1981	ENSG00000255122.1			-2.050072856	1.76E-10	1.34586E-09
1982	ENSG00000249751.4	ECSCR	641700	-1.24231552	1.81E-10	1.37907E-09
1983	ENSG00000188729.6	OSTN	344901	-3.546478063	1.82E-10	1.39121E-09
1984	ENSG00000171130.18	ATP6V0E2	155066	-1.309694113	1.84E-10	1.40229E-09
1985	ENSG00000140459.18	CYP11A1	1583	-2.247856105	1.84E-10	1.40513E-09
1986	ENSG00000286647.1			-3.323075415	1.84E-10	1.40513E-09
1987	ENSG00000287067.1			-1.186052754	1.95E-10	1.48253E-09
1988	ENSG00000264500.1	MIR3124	100422879	-1.253404532	1.99E-10	1.51314E-09
1989	ENSG00000161905.13	ALOX15	246	-2.723162553	2.00E-10	1.51838E-09
1990	ENSG00000234949.2	RAB17-DT	105373958	-2.016715312	2.00E-10	1.5224E-09
1991	ENSG00000243004.6	MACC1-OT1		-1.560804651	2.04E-10	1.54636E-09
1992	ENSG00000136918.8	WDR38	401551	-1.958205732	2.04E-10	1.55044E-09
1993	ENSG00000242953.1			-3.771697449	2.05E-10	1.55565E-09
1994	ENSG00000237361.3	TUSC8		-3.713789289	2.11E-10	1.59954E-09
1995	ENSG00000079841.18	RIMS1	22999	-2.244645878	2.12E-10	1.6058E-09
1996	ENSG00000234690.7	EPCAM-DT	101927043	-1.65483599	2.16E-10	1.63593E-09
1997	ENSG00000036672.16	USP2	9099	-1.564289795	2.17E-10	1.6391E-09
1998	ENSG00000240665.1	LSP1P2		-3.797690499	2.18E-10	1.64801E-09
1999	ENSG00000226070.1	LINC00951		-3.992602668	2.22E-10	1.67305E-09
2000	ENSG00000103056.12	SMPD3	55512	-1.54362712	2.22E-10	1.67437E-09
2001	ENSG00000259417.3	CTXND1	100996492	-1.426441828	2.22E-10	1.67654E-09
2002	ENSG00000257337.7	TNS2-AS1	283335	-1.05107787	2.23E-10	1.68049E-09
2003	ENSG00000105784.15	RUNDC3B	154661	-1.142012643	2.23E-10	1.68337E-09
2004	ENSG00000095713.14	CRTAC1	55118	-2.050938839	2.24E-10	1.68915E-09
2005	ENSG00000189056.14	RELN	5649	-1.715582434	2.26E-10	1.7024E-09
2006	ENSG00000196724.13	ZNF418	147686	-1.355313652	2.36E-10	1.7768E-09
2007	ENSG00000175147.13	TMEM51-AS1	200197	-1.074714626	2.38E-10	1.79269E-09
2008	ENSG00000198488.10	B3GNT6	192134	-3.010159728	2.44E-10	1.83221E-09
2009	ENSG00000178055.13	PRSS42P		-2.116697057	2.45E-10	1.84129E-09
2010	ENSG00000167858.13	TEKT1	83659	-2.018210568	2.46E-10	1.84757E-09
2011	ENSG00000178075.20	GRAMD1C	54762	-1.085453131	2.50E-10	1.87452E-09
2012	ENSG00000232803.1	SLCO4A1-AS1	100127888	-1.8897685	2.51E-10	1.88237E-09
2013	ENSG00000168427.9	KLHL30	377007	-2.116563999	2.52E-10	1.88874E-09
2014	ENSG00000178734.5	LMO7DN	729420	-1.873439169	2.59E-10	1.9395E-09
2015	ENSG00000104332.12	SFRP1	6422	-1.9965323	2.61E-10	1.95408E-09
2016	ENSG00000132874.15	SLC14A2	8170	-1.490178031	2.62E-10	1.95845E-09
2017	ENSG00000270765.6	GAS2L2	246176	-2.053735184	2.64E-10	1.975E-09
2018	ENSG00000204928.2	GRXCR2	643226	-1.126849426	2.65E-10	1.97584E-09
2019	ENSG00000184012.13	TMPRSS2	7113	-2.254171816	2.67E-10	1.99244E-09
2020	ENSG00000233110.1	SORBS2-AS1	125177376	-1.78548475	2.73E-10	2.03554E-09
2021	ENSG00000134873.10	CLDN10	9071	-2.883544954	2.74E-10	2.04339E-09
2022	ENSG00000146005.4	PSD2	84249	-1.634219146	2.75E-10	2.0497E-09
2023	ENSG00000184588.18	PDE4B	5142	-1.332582631	2.77E-10	2.06372E-09
2024	ENSG00000139648.7	KRT71	112802	-2.013719566	2.86E-10	2.12568E-09
2025	ENSG00000273132.1		124901427	-1.842247667	2.92E-10	2.1714E-09
2026	ENSG00000104490.18	NCALD	83988	-1.289418727	2.98E-10	2.21103E-09
2027	ENSG00000165917.10	RAPSN	5913	-2.119075571	3.00E-10	2.22504E-09
2028	ENSG00000186204.15	CYP4F12	66002	-2.061843805	3.00E-10	2.22882E-09
2029	ENSG00000243422.2	RPL23AP49		-1.742311896	3.07E-10	2.27593E-09
2030	ENSG00000185669.6	SNAI3	333929	-1.07601833	3.07E-10	2.27689E-09
2031	ENSG00000288109.1		101928045	-2.135134092	3.10E-10	2.29751E-09
2032	ENSG00000263011.1		124903629	-1.290681088	3.14E-10	2.3289E-09
2033	ENSG00000198453.13	ZNF568	374900	-1.209545032	3.16E-10	2.3408E-09
2034	ENSG00000283138.1			-1.998934354	3.26E-10	2.41274E-09
2035	ENSG00000168679.18	SLC16A4	9122	-1.210125819	3.27E-10	2.42016E-09
2036	ENSG00000273077.1		101927259	-2.086770224	3.30E-10	2.44294E-09
2037	ENSG00000254533.1			-2.142976653	3.35E-10	2.47542E-09
2038	ENSG00000127325.19	BEST3	144453	-2.692885008	3.36E-10	2.48343E-09
2039	ENSG00000177191.2	B3GNT8	374907	-1.060595389	3.41E-10	2.5155E-09
2040	ENSG00000010282.15	HHATL	57467	-3.419120501	3.49E-10	2.57265E-09
2041	ENSG00000161055.4	SCGB3A1	92304	-3.54992659	3.52E-10	2.59521E-09
2042	ENSG00000197748.13	CFAP43	80217	-1.391235374	3.53E-10	2.60323E-09
2043	ENSG00000130649.10	CYP2E1	1571	-1.942005006	3.64E-10	2.67994E-09
2044	ENSG00000145287.11	PLAC8	51316	-2.076734464	3.79E-10	2.78288E-09
2045	ENSG00000260951.2			-4.361556246	3.83E-10	2.81222E-09
2046	ENSG00000214652.6	ZNF727	442319	-1.726393173	3.84E-10	2.81697E-09
2047	ENSG00000265751.1	GREB1L-AS1	101927521	-4.37239646	3.99E-10	2.92348E-09
2048	ENSG00000162873.15	KLHDC8A	55220	-2.117945887	4.00E-10	2.92628E-09
2049	ENSG00000129596.5	CDO1	1036	-1.908695151	4.00E-10	2.93039E-09
2050	ENSG00000148488.17	ST8SIA6	338596	-1.526717474	4.01E-10	2.93154E-09
2051	ENSG00000178146.9	GK4P		-1.158311271	4.09E-10	2.99058E-09
2052	ENSG00000188396.4	DYNLT4	343521	-1.284312362	4.12E-10	3.008E-09
2053	ENSG00000244968.7	LIFR-AS1	100506495	-1.549125547	4.20E-10	3.07053E-09
2054	ENSG00000016391.11	CHDH	55349	-1.792407534	4.27E-10	3.11849E-09
2055	ENSG00000168530.16	MYL1	4632	-3.919046723	4.39E-10	3.19965E-09
2056	ENSG00000109158.11	GABRA4	2557	-3.108017727	4.39E-10	3.20258E-09
2057	ENSG00000148948.8	LRRC4C	57689	-1.849908789	4.40E-10	3.2091E-09
2058	ENSG00000092295.12	TGM1	7051	-1.841901273	4.50E-10	3.27612E-09
2059	ENSG00000136542.9	GALNT5	11227	-1.709698272	4.53E-10	3.29855E-09
2060	ENSG00000124107.5	SLPI	6590	-1.645451963	4.53E-10	3.29891E-09
2061	ENSG00000157119.12	KLHL40	131377	-3.749479795	4.61E-10	3.35016E-09
2062	ENSG00000286463.1			-2.869977692	4.67E-10	3.39436E-09
2063	ENSG00000255794.9	RMST	196475	-3.09179639	4.81E-10	3.49135E-09
2064	ENSG00000169122.11	FAM110B	90362	-1.28407205	4.85E-10	3.51875E-09
2065	ENSG00000126562.17	WNK4	65266	-1.754260209	4.87E-10	3.53384E-09
2066	ENSG00000145491.12	ROPN1L	83853	-1.609061056	4.88E-10	3.53495E-09
2067	ENSG00000287860.1			-1.43061167	4.89E-10	3.54525E-09
2068	ENSG00000122012.14	SV2C	22987	-1.305708206	4.90E-10	3.55412E-09
2069	ENSG00000106772.18	PRUNE2	158471	-2.034557375	4.92E-10	3.56804E-09
2070	ENSG00000005249.13	PRKAR2B	5577	-1.293400347	5.08E-10	3.67441E-09
2071	ENSG00000287117.1			-1.720722659	5.20E-10	3.75949E-09
2072	ENSG00000130176.8	CNN1	1264	-1.505507327	5.28E-10	3.81234E-09
2073	ENSG00000242317.1			-3.626353579	5.32E-10	3.83724E-09
2074	ENSG00000236609.4	ZNF853	54753	-1.47508426	5.60E-10	4.02629E-09
2075	ENSG00000196381.11	ZNF781		-1.162057722	5.61E-10	4.03578E-09
2076	ENSG00000254024.1	GRHL2-DT	107986962	-1.513825184	5.62E-10	4.04392E-09
2077	ENSG00000248383.5	PCDHAC1	56135	-2.224476301	5.63E-10	4.0494E-09
2078	ENSG00000155761.14	SPAG17	200162	-1.674243505	5.66E-10	4.07151E-09
2079	ENSG00000224958.6	PGM5-AS1	572558	-2.540807994	5.66E-10	4.07215E-09
2080	ENSG00000223345.3	H2BP1		-1.648397185	5.71E-10	4.10296E-09
2081	ENSG00000149451.18	ADAM33	80332	-1.308931568	5.86E-10	4.20698E-09
2082	ENSG00000185133.14	INPP5J	27124	-1.259021435	5.99E-10	4.29291E-09
2083	ENSG00000183134.5	PTGDR2	11251	-1.279337393	6.01E-10	4.30618E-09
2084	ENSG00000233517.1			-2.024552781	6.08E-10	4.35306E-09
2085	ENSG00000170893.4	TRH	7200	-2.095231477	6.19E-10	4.42389E-09
2086	ENSG00000250266.2	LINC01612		-2.706071978	6.21E-10	4.43568E-09
2087	ENSG00000183833.16	CFAP91	89876	-2.523535093	6.41E-10	4.57214E-09
2088	ENSG00000234219.1	CDCA4P4		-1.278385078	6.61E-10	4.71473E-09
2089	ENSG00000163263.7	CFAP141	388701	-1.619458288	6.62E-10	4.71473E-09
2090	ENSG00000182463.16	TSHZ2	128553	-1.070744454	6.63E-10	4.72467E-09
2091	ENSG00000197641.12	SERPINB13	5275	-1.655130839	6.72E-10	4.78354E-09
2092	ENSG00000261040.7	WFDC21P		-1.809751032	6.74E-10	4.79533E-09
2093	ENSG00000287184.1			-4.293096185	6.79E-10	4.82459E-09
2094	ENSG00000265519.1			-1.415427895	6.80E-10	4.8292E-09
2095	ENSG00000088726.16	TMEM40	55287	-1.221593751	6.82E-10	4.84621E-09
2096	ENSG00000273259.3			-2.374827552	7.08E-10	5.02108E-09
2097	ENSG00000221923.9	ZNF880	400713	-1.211219639	7.18E-10	5.08698E-09
2098	ENSG00000205106.4	LINC02716		-1.513988951	7.25E-10	5.13574E-09
2099	ENSG00000152580.8	IGSF10	285313	-2.029542934	7.25E-10	5.13719E-09
2100	ENSG00000225050.2		102724929	-4.121527405	7.28E-10	5.15484E-09
2101	ENSG00000263489.1			-4.121317533	7.36E-10	5.20883E-09
2102	ENSG00000009950.16	MLXIPL	51085	-2.097443251	7.41E-10	5.24053E-09
2103	ENSG00000270659.1			-1.29434201	7.45E-10	5.26667E-09
2104	ENSG00000160339.16	FCN2	2220	-3.217053773	7.48E-10	5.287E-09
2105	ENSG00000159212.13	CLIC6	54102	-1.722721646	7.61E-10	5.37649E-09
2106	ENSG00000173702.7	MUC13	56667	-2.755908466	7.63E-10	5.38643E-09
2107	ENSG00000276855.1			-1.163259423	7.65E-10	5.40171E-09
2108	ENSG00000147642.17	SYBU	55638	-1.389064728	7.82E-10	5.51242E-09
2109	ENSG00000230627.1			-2.898172376	7.91E-10	5.56943E-09
2110	ENSG00000166405.15	RIC3	79608	-1.76066964	7.93E-10	5.5865E-09
2111	ENSG00000136158.12	SPRY2	10253	-1.009287204	8.14E-10	5.72665E-09
2112	ENSG00000064655.19	EYA2	2139	-1.827184289	8.20E-10	5.76448E-09
2113	ENSG00000185985.11	SLITRK2	84631	-1.436128625	8.31E-10	5.83702E-09
2114	ENSG00000117472.10	TSPAN1	10103	-1.807070281	8.38E-10	5.87937E-09
2115	ENSG00000087128.10	TMPRSS11E	28983	-1.971059901	8.49E-10	5.95704E-09
2116	ENSG00000088002.12	SULT2B1	6820	-1.585795774	8.51E-10	5.97103E-09
2117	ENSG00000144642.22	RBMS3	27303	-1.005798536	8.57E-10	6.00686E-09
2118	ENSG00000124212.6	PTGIS	5740	-1.989658615	8.59E-10	6.02516E-09
2119	ENSG00000073737.17	DHRS9	10170	-1.738914528	8.60E-10	6.02683E-09
2120	ENSG00000270571.2	TACR1-AS1	105374811	-1.137596245	8.83E-10	6.17701E-09
2121	ENSG00000144668.12	ITGA9	3680	-1.075902047	8.92E-10	6.23291E-09
2122	ENSG00000121335.12	PRB2	653247	-3.793567298	8.99E-10	6.27534E-09
2123	ENSG00000046889.19	PREX2	80243	-1.145251581	9.13E-10	6.36995E-09
2124	ENSG00000258661.1		105370455	-1.849965682	9.14E-10	6.37245E-09
2125	ENSG00000267659.5	LINC01482	101928104	-1.385332539	9.49E-10	6.61577E-09
2126	ENSG00000262133.1			-2.121502545	9.58E-10	6.67272E-09
2127	ENSG00000272542.1			-2.634477759	9.66E-10	6.7235E-09
2128	ENSG00000007402.12	CACNA2D2	9254	-1.068837335	9.74E-10	6.77457E-09
2129	ENSG00000255189.1	GLYATL1P1		-1.866300155	1.00259E-09	6.96176E-09
2130	ENSG00000225028.1			-1.999744819	1.01665E-09	7.05321E-09
2131	ENSG00000129295.9	DNAAF11	23639	-1.321279052	1.02308E-09	7.09535E-09
2132	ENSG00000122707.12	RECK	8434	-1.051686672	1.03134E-09	7.14886E-09
2133	ENSG00000285996.1			-1.336999953	1.03521E-09	7.17323E-09
2134	ENSG00000065675.15	PRKCQ	5588	-1.257767859	1.05302E-09	7.28766E-09
2135	ENSG00000099957.16	P2RX6	9127	-1.619340441	1.0586E-09	7.3225E-09
2136	ENSG00000286410.1			-2.097903057	1.07401E-09	7.42114E-09
2137	ENSG00000206145.8	P2RX6P		-2.075910832	1.08293E-09	7.47774E-09
2138	ENSG00000131080.15	EDA2R	60401	-1.29327782	1.10078E-09	7.5957E-09
2139	ENSG00000185559.16	DLK1	8788	-3.833386194	1.11738E-09	7.70756E-09
2140	ENSG00000166979.13	EVA1C	59271	-1.068555717	1.11906E-09	7.71782E-09
2141	ENSG00000285373.2	LINC02478	100505984	-1.598152063	1.12772E-09	7.77209E-09
2142	ENSG00000181634.8	TNFSF15	9966	-1.293012522	1.13433E-09	7.81492E-09
2143	ENSG00000277737.3			-2.567174029	1.14335E-09	7.87437E-09
2144	ENSG00000184669.9	OR7E14P		-1.538681208	1.14595E-09	7.8909E-09
2145	ENSG00000279314.1			-1.531265324	1.17821E-09	8.10461E-09
2146	ENSG00000200550.1	RNU6-137P		-1.165217526	1.18348E-09	8.13925E-09
2147	ENSG00000214274.10	ANG	283	-1.070253556	1.20274E-09	8.26327E-09
2148	ENSG00000180251.5	SLC9A4	389015	-2.633053746	1.20555E-09	8.28113E-09
2149	ENSG00000184185.10	KCNJ12	3768	-1.662797978	1.20669E-09	8.28677E-09
2150	ENSG00000260583.1			-1.292677928	1.20882E-09	8.29929E-09
2151	ENSG00000287559.1			-3.017522634	1.2208E-09	8.37868E-09
2152	ENSG00000255308.1	CSRP3-AS1		-1.616146407	1.22502E-09	8.40468E-09
2153	ENSG00000204065.3	TCEAL5	340543	-2.232748951	1.25334E-09	8.58863E-09
2154	ENSG00000174348.14	PODN	127435	-1.611598045	1.26296E-09	8.64708E-09
2155	ENSG00000187955.12	COL14A1	7373	-1.360003917	1.27453E-09	8.71873E-09
2156	ENSG00000163492.15	CCDC141	285025	-1.568453667	1.28267E-09	8.76991E-09
2157	ENSG00000285805.1			-2.062761234	1.31353E-09	8.96388E-09
2158	ENSG00000155970.12	MICU3	286097	-1.344074569	1.31984E-09	9.00231E-09
2159	ENSG00000113594.10	LIFR	3977	-1.407458453	1.32978E-09	9.06783E-09
2160	ENSG00000172399.6	MYOZ2	51778	-3.138008628	1.33497E-09	9.09925E-09
2161	ENSG00000179277.9	MEIS3P1		-1.464944456	1.34365E-09	9.15526E-09
2162	ENSG00000187942.12	LDLRAD2	401944	-1.06183157	1.35044E-09	9.19995E-09
2163	ENSG00000234810.4			-1.987543439	1.35924E-09	9.25828E-09
2164	ENSG00000235535.8	TRDN-AS1	101927990	-2.564220624	1.388E-09	9.44767E-09
2165	ENSG00000267423.1			-3.843708524	1.405E-09	9.55683E-09
2166	ENSG00000184347.15	SLIT3	6586	-1.395838494	1.43525E-09	9.7526E-09
2167	ENSG00000251664.5	PCDHA12	56137	-3.214107869	1.44316E-09	9.80028E-09
2168	ENSG00000243566.7	UPK3B	105375355	-1.777183656	1.44722E-09	9.82548E-09
2169	ENSG00000158481.13	CD1C	911	-1.293454365	1.47457E-09	9.99917E-09
2170	ENSG00000142657.21	PGD	5226	-1.037287191	1.49013E-09	1.00995E-08
2171	ENSG00000158423.17	RIBC1	158787	-1.005356026	1.4993E-09	1.01599E-08
2172	ENSG00000169347.17	GP2	2813	-4.97623175	1.50797E-09	1.02169E-08
2173	ENSG00000107242.20	PIP5K1B	8395	-1.594893601	1.51424E-09	1.02577E-08
2174	ENSG00000286280.1			-3.408711637	1.51763E-09	1.02771E-08
2175	ENSG00000188013.6	MEIS3P2		-1.601632	1.55958E-09	1.05432E-08
2176	ENSG00000102349.18	KLF8	11279	-1.164943387	1.56678E-09	1.05887E-08
2177	ENSG00000173966.7	COMMD5P1		-2.143921231	1.56685E-09	1.05887E-08
2178	ENSG00000286781.1			-1.191201714	1.60046E-09	1.08103E-08
2179	ENSG00000143839.15	REN	5972	-2.095428242	1.61589E-09	1.09108E-08
2180	ENSG00000260653.6	MIR3147HG		-2.958432869	1.62146E-09	1.09466E-08
2181	ENSG00000236473.1	KRT43P		-2.626500634	1.64887E-09	1.11241E-08
2182	ENSG00000198416.9	ZNF658B		-1.015091104	1.68722E-09	1.13711E-08
2183	ENSG00000184601.11	C14orf180	400258	-4.376481789	1.70328E-09	1.14755E-08
2184	ENSG00000144339.12	TMEFF2	23671	-2.429282966	1.73557E-09	1.16791E-08
2185	ENSG00000185275.6	CD24P4		-1.534193351	1.79594E-09	1.20629E-08
2186	ENSG00000288103.1			-1.345594341	1.81465E-09	1.21824E-08
2187	ENSG00000238102.2			-5.325382741	1.85885E-09	1.24601E-08
2188	ENSG00000237440.9	ZNF737	100129842	-1.254839164	1.87694E-09	1.25792E-08
2189	ENSG00000175084.12	DES	1674	-2.9739066	1.89162E-09	1.2669E-08
2190	ENSG00000178750.3	STX19	415117	-1.335522252	1.89576E-09	1.26946E-08
2191	ENSG00000230635.2	CYP4F60P		-2.586838129	2.00458E-09	1.33894E-08
2192	ENSG00000145808.10	ADAMTS19	171019	-2.715157404	2.01218E-09	1.34334E-08
2193	ENSG00000069431.11	ABCC9	10060	-1.353544047	2.01307E-09	1.3437E-08
2194	ENSG00000246100.4	LINC00900		-1.467640239	2.0206E-09	1.34828E-08
2195	ENSG00000174407.13	MIR1-1HG	128826	-3.986280048	2.03149E-09	1.35486E-08
2196	ENSG00000272143.1	FGF14-AS2	283481	-1.397530446	2.03714E-09	1.3584E-08
2197	ENSG00000188257.12	PLA2G2A	5320	-2.534229145	2.11393E-09	1.40842E-08
2198	ENSG00000244756.1			-1.764090899	2.16878E-09	1.4423E-08
2199	ENSG00000259883.2	EHD4-AS1	101928363	-1.11872829	2.16993E-09	1.44282E-08
2200	ENSG00000235186.1			-2.883745986	2.18148E-09	1.45002E-08
2201	ENSG00000187957.8	DNER	92737	-1.84286535	2.18296E-09	1.45076E-08
2202	ENSG00000243232.6	PCDHAC2	56134	-1.879192758	2.24095E-09	1.4868E-08
2203	ENSG00000253490.5	LINC02099	101929450	-1.224068181	2.28129E-09	1.5118E-08
2204	ENSG00000171408.14	PDE7B	27115	-1.039635702	2.29115E-09	1.51808E-08
2205	ENSG00000165272.16	AQP3	360	-1.550098326	2.29254E-09	1.51874E-08
2206	ENSG00000176490.5	DIRAS1	148252	-1.761540135	2.34633E-09	1.5523E-08
2207	ENSG00000111241.2	FGF6	2251	-3.400805585	2.35533E-09	1.55748E-08
2208	ENSG00000274173.1	LINC02967		-1.304780511	2.3576E-09	1.55872E-08
2209	ENSG00000153956.16	CACNA2D1	781	-1.463245895	2.38231E-09	1.57401E-08
2210	ENSG00000171798.18	KNDC1	85442	-1.750684733	2.41809E-09	1.59632E-08
2211	ENSG00000265369.3	PCAT18	728606	-3.428553778	2.42176E-09	1.59794E-08
2212	ENSG00000164588.8	HCN1	348980	-2.804444283	2.44974E-09	1.61533E-08
2213	ENSG00000179902.13	CFAP276	127003	-2.650154116	2.47213E-09	1.62955E-08
2214	ENSG00000076344.16	RGS11	8786	-1.389775927	2.48686E-09	1.63899E-08
2215	ENSG00000112232.10	KHDRBS2	202559	-2.084148669	2.50493E-09	1.65008E-08
2216	ENSG00000230062.5	ANKRD66	100287718	-3.213530733	2.50784E-09	1.65172E-08
2217	ENSG00000224090.1		100129175	-3.053487964	2.51421E-09	1.65536E-08
2218	ENSG00000117507.6	FMO6P		-2.859744091	2.51466E-09	1.65538E-08
2219	ENSG00000003249.15	DBNDD1	79007	-1.236697749	2.54139E-09	1.67208E-08
2220	ENSG00000183196.10	CHST6	4166	-1.565713634	2.55781E-09	1.68184E-08
2221	ENSG00000135437.10	RDH5	5959	-1.126151384	2.56342E-09	1.6844E-08
2222	ENSG00000177354.12	C10orf71	118461	-3.58341536	2.62565E-09	1.72302E-08
2223	ENSG00000249852.1			-1.511993503	2.66077E-09	1.74519E-08
2224	ENSG00000285731.1			-1.895383081	2.78155E-09	1.8205E-08
2225	ENSG00000154822.18	PLCL2	23228	-1.100929845	2.79468E-09	1.82848E-08
2226	ENSG00000134215.16	VAV3	10451	-1.028523912	2.83695E-09	1.85492E-08
2227	ENSG00000198842.10	STYXL2	92235	-3.056056995	2.85753E-09	1.86776E-08
2228	ENSG00000187054.16	TMPRSS11A	339967	-2.181385442	2.93948E-09	1.91848E-08
2229	ENSG00000182175.14	RGMA	56963	-1.253952514	2.94212E-09	1.91988E-08
2230	ENSG00000137033.11	IL33	90865	-1.773487099	2.99524E-09	1.95294E-08
2231	ENSG00000135409.11	AMHR2	269	-1.715090764	3.0038E-09	1.9582E-08
2232	ENSG00000115255.12	REEP6	92840	-1.075037612	3.05022E-09	1.98683E-08
2233	ENSG00000285591.1			-2.106078551	3.06908E-09	1.99813E-08
2234	ENSG00000158486.13	DNAH3	55567	-1.167486969	3.11141E-09	2.02502E-08
2235	ENSG00000261026.1			-1.591958939	3.12256E-09	2.03161E-08
2236	ENSG00000251000.1	GGCTP1		-1.04552037	3.13517E-09	2.03881E-08
2237	ENSG00000182533.7	CAV3	859	-3.212983614	3.16143E-09	2.05488E-08
2238	ENSG00000204687.4	MAS1L	116511	-2.410297444	3.22884E-09	2.09526E-08
2239	ENSG00000257501.6	PAFAH1B2P2		-2.92640443	3.23934E-09	2.10173E-08
2240	ENSG00000181143.15	MUC16	94025	-2.184808576	3.28398E-09	2.1293E-08
2241	ENSG00000167363.14	FN3K	64122	-1.076551608	3.34502E-09	2.16711E-08
2242	ENSG00000119862.13	LGALSL	29094	-1.186682702	3.36891E-09	2.18115E-08
2243	ENSG00000275718.2	CCL15	6359	-2.458774774	3.4323E-09	2.22038E-08
2244	ENSG00000235105.1			-1.098215732	3.4396E-09	2.22438E-08
2245	ENSG00000164825.4	DEFB1	1672	-1.737636652	3.48192E-09	2.24955E-08
2246	ENSG00000267640.6			-1.624601101	3.49848E-09	2.25914E-08
2247	ENSG00000242767.1	ZBTB20-AS4	100874131	-1.850113476	3.6201E-09	2.33388E-08
2248	ENSG00000276772.1			-2.218318858	3.64531E-09	2.3486E-08
2249	ENSG00000267272.5	LINC01140	339524	-1.200309179	3.66693E-09	2.36176E-08
2250	ENSG00000186642.16	PDE2A	5138	-1.050224611	3.68551E-09	2.37296E-08
2251	ENSG00000241186.10	CRIPTO	6997	-2.12368641	3.69525E-09	2.37807E-08
2252	ENSG00000162148.11	SAXO4	220004	-1.009234392	3.78618E-09	2.43343E-08
2253	ENSG00000226197.3			-1.545903428	3.7916E-09	2.43652E-08
2254	ENSG00000111110.12	PPM1H	57460	-1.486864893	3.82464E-09	2.45655E-08
2255	ENSG00000260971.4			-1.51910069	3.84251E-09	2.46643E-08
2256	ENSG00000281969.1			-1.815313571	3.95982E-09	2.53681E-08
2257	ENSG00000279017.1			-2.216648624	3.98383E-09	2.55136E-08
2258	ENSG00000000005.6	TNMD	64102	-3.467150743	3.9848E-09	2.55157E-08
2259	ENSG00000275678.1			-2.875143422	4.1013E-09	2.62151E-08
2260	ENSG00000235663.1	SAPCD1-AS1		-1.414498113	4.12285E-09	2.63401E-08
2261	ENSG00000103460.17	TOX3	27324	-2.864103157	4.25124E-09	2.71254E-08
2262	ENSG00000271579.1			-1.757143683	4.35853E-09	2.77832E-08
2263	ENSG00000175699.15	CCDC197	256369	-2.270267642	4.3782E-09	2.78906E-08
2264	ENSG00000276644.5	DACH1	1602	-1.480030632	4.44706E-09	2.83111E-08
2265	ENSG00000156265.16	MAP3K7CL	56911	-1.075104143	4.48589E-09	2.85446E-08
2266	ENSG00000287978.1			-1.110961033	4.49311E-09	2.85859E-08
2267	ENSG00000231993.1	EP300-AS1	101927279	-1.091855329	4.60361E-09	2.9256E-08
2268	ENSG00000257002.1	CHRM1-AS1	124902683	-2.693233155	4.60434E-09	2.9256E-08
2269	ENSG00000258789.1			-1.020846333	4.66146E-09	2.96047E-08
2270	ENSG00000214691.9	LINC01913	388942	-2.841173805	4.67028E-09	2.9656E-08
2271	ENSG00000118322.14	ATP10B	23120	-1.616315968	4.6834E-09	2.97346E-08
2272	ENSG00000229619.4	MBNL1-AS1	401093	-1.090530482	4.75304E-09	3.01284E-08
2273	ENSG00000255772.5	LINC01479	101927922	-2.189198666	4.7801E-09	3.02854E-08
2274	ENSG00000262884.1		101927911	-1.562099396	4.86332E-09	3.07881E-08
2275	ENSG00000144649.9	GASK1A	729085	-1.499405906	4.86924E-09	3.08207E-08
2276	ENSG00000275152.5	CCL16	6360	-1.662031488	4.8833E-09	3.09047E-08
2277	ENSG00000117114.20	ADGRL2	23266	-1.036051696	4.94758E-09	3.12965E-08
2278	ENSG00000154262.13	ABCA6	23460	-1.218493584	5.01728E-09	3.1697E-08
2279	ENSG00000236712.1			-3.232017944	5.06223E-09	3.19707E-08
2280	ENSG00000264151.6			-2.680561445	5.11514E-09	3.22844E-08
2281	ENSG00000272411.1			-1.529162597	5.20931E-09	3.28473E-08
2282	ENSG00000055813.6	CCDC85A	114800	-1.708224769	5.22114E-09	3.291E-08
2283	ENSG00000186912.6	P2RY4	5030	-1.555981354	5.22704E-09	3.29382E-08
2284	ENSG00000286452.1			-4.018021323	5.30699E-09	3.3426E-08
2285	ENSG00000151715.8	TMEM45B	120224	-1.604621513	5.45195E-09	3.42791E-08
2286	ENSG00000259828.1			-2.277585568	5.49292E-09	3.45258E-08
2287	ENSG00000094755.17	GABRP	2568	-2.474576105	5.54539E-09	3.48501E-08
2288	ENSG00000241151.1	LINC02066	105374158	-3.140141293	5.58768E-09	3.51103E-08
2289	ENSG00000129159.9	KCNC1	3746	-1.658623213	5.69493E-09	3.57107E-08
2290	ENSG00000176945.17	MUC20	200958	-1.778433047	5.75593E-09	3.60703E-08
2291	ENSG00000283156.1			-1.176522288	5.85431E-09	3.66637E-08
2292	ENSG00000131737.5	KRT34	3885	-1.860320015	5.90805E-09	3.69827E-08
2293	ENSG00000234132.2		101929305	-2.823954863	5.91685E-09	3.70279E-08
2294	ENSG00000215105.4	TTC3P1		-1.13631375	5.91713E-09	3.70279E-08
2295	ENSG00000223863.1	LINC01805	105374774	-1.695933449	5.98575E-09	3.74307E-08
2296	ENSG00000244953.1			-1.400413489	6.06821E-09	3.79015E-08
2297	ENSG00000165621.8	OXGR1	27199	-1.910890488	6.10609E-09	3.8126E-08
2298	ENSG00000110786.18	PTPN5	84867	-1.516712842	6.15695E-09	3.84315E-08
2299	ENSG00000130226.17	DPP6	1804	-1.956216363	6.24398E-09	3.89441E-08
2300	ENSG00000229267.3	SNHG31		-1.134640588	6.34584E-09	3.95172E-08
2301	ENSG00000196800.6	SPINK14	408187	-4.067424843	6.42815E-09	4.0011E-08
2302	ENSG00000276527.1		105370185	-1.649598002	6.44357E-09	4.01006E-08
2303	ENSG00000286996.1			-1.14880127	6.45827E-09	4.01795E-08
2304	ENSG00000273175.1			-1.156765931	6.53268E-09	4.06106E-08
2305	ENSG00000210195.2	MT-TT		-1.644791472	6.58217E-09	4.0899E-08
2306	ENSG00000097096.9	SYDE2	84144	-1.121942428	6.64498E-09	4.12634E-08
2307	ENSG00000148346.12	LCN2	3934	-1.671569556	6.73264E-09	4.17816E-08
2308	ENSG00000105650.22	PDE4C	5143	-2.141581343	6.87573E-09	4.26096E-08
2309	ENSG00000153820.13	SPHKAP	80309	-2.842900396	6.88703E-09	4.26697E-08
2310	ENSG00000129910.8	CDH15	1013	-1.91877948	7.08897E-09	4.38693E-08
2311	ENSG00000183034.13	OTOP2	92736	-1.819591158	7.16388E-09	4.42914E-08
2312	ENSG00000228723.6	SRGAP3-AS2	101927416	-3.295353258	7.27811E-09	4.49556E-08
2313	ENSG00000269067.2	ZNF728	388523	-2.192410209	7.33484E-09	4.52848E-08
2314	ENSG00000237179.6	LINC01797		-2.85474952	7.36681E-09	4.54751E-08
2315	ENSG00000168772.11	CXXC4	80319	-1.55095368	7.3694E-09	4.5484E-08
2316	ENSG00000223392.1	CLDN10-AS1	100874194	-3.213787234	7.37263E-09	4.54898E-08
2317	ENSG00000214425.8	LRRC37A4P		-1.361368711	7.42505E-09	4.57868E-08
2318	ENSG00000196260.5	SFTA2	389376	-2.735120826	7.42539E-09	4.57868E-08
2319	ENSG00000273252.1	OR7E39P		-2.030060003	7.45096E-09	4.59231E-08
2320	ENSG00000250426.2	FTLP10		-2.85780282	7.54302E-09	4.64616E-08
2321	ENSG00000236427.1	CCDST	339400	-1.848773569	7.58978E-09	4.67206E-08
2322	ENSG00000281655.1			-2.583854484	7.72229E-09	4.75067E-08
2323	ENSG00000250479.9	CHCHD10	400916	-1.025391247	7.75961E-09	4.7729E-08
2324	ENSG00000105357.19	MYH14	79784	-1.350538271	7.78748E-09	4.78633E-08
2325	ENSG00000101292.8	PROKR2	128674	-3.207398554	7.818E-09	4.80434E-08
2326	ENSG00000283376.1			-2.820100434	7.98914E-09	4.90495E-08
2327	ENSG00000004838.14	ZMYND10	51364	-1.520745737	8.0134E-09	4.91908E-08
2328	ENSG00000267385.1			-1.796192452	8.06183E-09	4.94728E-08
2329	ENSG00000185149.6	NPY2R	4887	-3.256985088	8.06538E-09	4.94869E-08
2330	ENSG00000259134.7	LINC00924	145820	-1.116528481	8.07636E-09	4.95466E-08
2331	ENSG00000186399.10	GOLGA8R	101059918	-1.042420095	8.14752E-09	4.9929E-08
2332	ENSG00000113924.12	HGD	3081	-1.850874258	8.19417E-09	5.01994E-08
2333	ENSG00000115468.12	EFHD1	80303	-1.365685908	8.31126E-09	5.08853E-08
2334	ENSG00000184828.10	ZBTB7C	201501	-1.454016669	8.36071E-09	5.11723E-08
2335	ENSG00000261553.5			-1.445867267	8.40751E-09	5.14428E-08
2336	ENSG00000235904.3	RBMS3-AS3	100873979	-1.504489829	8.71503E-09	5.32259E-08
2337	ENSG00000213876.4	RPL7AP64		-1.218434198	8.81054E-09	5.37595E-08
2338	ENSG00000287720.1			-1.864829091	8.81961E-09	5.38065E-08
2339	ENSG00000232680.2			-1.824221423	8.83563E-09	5.3896E-08
2340	ENSG00000165071.15	TMEM71	137835	-1.219155157	8.9037E-09	5.42861E-08
2341	ENSG00000168269.10	FOXI1	2299	-3.338887617	8.91331E-09	5.43364E-08
2342	ENSG00000008196.13	TFAP2B	7021	-3.21965067	8.99273E-09	5.48037E-08
2343	ENSG00000237330.3	RNF223	401934	-1.348770963	9.03943E-09	5.50629E-08
2344	ENSG00000248464.1	FGF10-AS1	101927075	-2.396609269	9.20053E-09	5.6027E-08
2345	ENSG00000260711.2			-1.22428125	9.47076E-09	5.76195E-08
2346	ENSG00000278518.1			-2.088848203	9.47293E-09	5.76238E-08
2347	ENSG00000261472.1			-3.2282132	9.74997E-09	5.9191E-08
2348	ENSG00000109654.16	TRIM2	23321	-1.098745443	9.97714E-09	6.04591E-08
2349	ENSG00000262519.1	TXNP4		-1.320660019	1.00504E-08	6.08768E-08
2350	ENSG00000059728.11	MXD1	4084	-1.019865057	1.02443E-08	6.19737E-08
2351	ENSG00000169418.10	NPR1	4881	-1.057625714	1.03766E-08	6.26978E-08
2352	ENSG00000227220.1			-2.041822058	1.04303E-08	6.30029E-08
2353	ENSG00000181885.18	CLDN7	1366	-1.205126774	1.05763E-08	6.38364E-08
2354	ENSG00000164746.14	C7orf57	136288	-1.426246011	1.06673E-08	6.43462E-08
2355	ENSG00000185818.8	NAT8L	339983	-1.628506474	1.06878E-08	6.44524E-08
2356	ENSG00000248485.2	PCP4L1	654790	-1.853950017	1.0714E-08	6.45987E-08
2357	ENSG00000106113.19	CRHR2	1395	-1.561506779	1.10001E-08	6.62631E-08
2358	ENSG00000183783.7	KCTD8	386617	-2.319236216	1.10741E-08	6.6668E-08
2359	ENSG00000174502.19	SLC26A9	115019	-1.833888015	1.11923E-08	6.73336E-08
2360	ENSG00000139737.22	SLAIN1	122060	-1.343328505	1.11931E-08	6.73336E-08
2361	ENSG00000204338.8	CYP21A1P		-1.561636586	1.12059E-08	6.74005E-08
2362	ENSG00000173432.12	SAA1	6288	-1.75653894	1.13797E-08	6.84041E-08
2363	ENSG00000161896.12	IP6K3	117283	-2.279848341	1.13894E-08	6.84314E-08
2364	ENSG00000120156.22	TEK	7010	-1.009718165	1.14979E-08	6.90626E-08
2365	ENSG00000135413.9	LACRT	90070	-8.887288904	1.15457E-08	6.9339E-08
2366	ENSG00000287340.1			-3.326560827	1.18919E-08	7.13531E-08
2367	ENSG00000234332.1	BCAS2P2		-1.535439988	1.20579E-08	7.22728E-08
2368	ENSG00000134398.15	ERN2	10595	-2.471258086	1.21009E-08	7.25084E-08
2369	ENSG00000285907.1			-1.463467648	1.2203E-08	7.30873E-08
2370	ENSG00000197496.6	SLC2A10	81031	-1.175517367	1.23892E-08	7.41798E-08
2371	ENSG00000213754.2	KPNA4P1		-1.387986771	1.24283E-08	7.43913E-08
2372	ENSG00000149021.7	SCGB1A1	7356	-3.289119949	1.25468E-08	7.50784E-08
2373	ENSG00000077279.19	DCX	1641	-2.08891322	1.28246E-08	7.66366E-08
2374	ENSG00000242770.2	CD200R1L-AS1		-3.166900343	1.28999E-08	7.70745E-08
2375	ENSG00000163126.15	ANKRD23	200539	-1.244476998	1.29384E-08	7.7293E-08
2376	ENSG00000198838.14	RYR3	6263	-1.435885864	1.30473E-08	7.7897E-08
2377	ENSG00000147872.10	PLIN2	123	-1.065637938	1.31431E-08	7.84097E-08
2378	ENSG00000234224.3	TMEM229A	730130	-3.33749774	1.3201E-08	7.87434E-08
2379	ENSG00000153347.10	FAM81B	153643	-2.657623192	1.33964E-08	7.98366E-08
2380	ENSG00000214313.8	AZGP1P1		-2.71132453	1.35086E-08	8.04932E-08
2381	ENSG00000250392.2	LINC02502	100996694	-2.771051955	1.36159E-08	8.10842E-08
2382	ENSG00000235491.1			-4.483507948	1.36336E-08	8.11649E-08
2383	ENSG00000259045.1	MTCO1P2		-1.219389941	1.36975E-08	8.15332E-08
2384	ENSG00000112796.10	ENPP5	59084	-1.805171482	1.3994E-08	8.3173E-08
2385	ENSG00000261217.2	BCAP31P1		-1.750692718	1.40534E-08	8.34761E-08
2386	ENSG00000286150.1			-1.800473934	1.41167E-08	8.38272E-08
2387	ENSG00000163092.21	XIRP2	129446	-3.542786954	1.45019E-08	8.59727E-08
2388	ENSG00000078579.9	FGF20	26281	-2.483554751	1.4524E-08	8.60908E-08
2389	ENSG00000207019.1	RNU6-167P		-1.91633152	1.47821E-08	8.75161E-08
2390	ENSG00000285873.1			-1.247728426	1.49277E-08	8.83519E-08
2391	ENSG00000272636.4	DOC2B	8447	-1.336073229	1.50714E-08	8.91623E-08
2392	ENSG00000175336.10	APOF	319	-1.851363889	1.50959E-08	8.92808E-08
2393	ENSG00000169509.6	CRCT1	54544	-2.135596105	1.51447E-08	8.95424E-08
2394	ENSG00000132514.14	CLEC10A	10462	-1.32089698	1.51961E-08	8.98299E-08
2395	ENSG00000286750.1			-2.019948938	1.52583E-08	9.01468E-08
2396	ENSG00000151322.19	NPAS3	64067	-1.322584481	1.55307E-08	9.16743E-08
2397	ENSG00000287620.1		105377035	-1.053072532	1.55458E-08	9.175E-08
2398	ENSG00000226622.6			-1.726055636	1.55494E-08	9.17573E-08
2399	ENSG00000248923.1	MTND5P11		-1.184674844	1.56874E-08	9.25445E-08
2400	ENSG00000123096.12	SSPN	8082	-1.078265897	1.5705E-08	9.26344E-08
2401	ENSG00000102935.12	ZNF423	23090	-1.187804203	1.57645E-08	9.29712E-08
2402	ENSG00000239556.4	UPK3BL3P		-1.349295857	1.60176E-08	9.43376E-08
2403	ENSG00000205002.4	AARD	441376	-2.180386676	1.61089E-08	9.48473E-08
2404	ENSG00000260721.1			-2.329722387	1.61597E-08	9.51325E-08
2405	ENSG00000221818.9	EBF2	64641	-1.31176008	1.63007E-08	9.59053E-08
2406	ENSG00000154678.18	PDE1C	5137	-1.18178884	1.67268E-08	9.82667E-08
2407	ENSG00000267507.1			-1.893694788	1.68594E-08	9.90017E-08
2408	ENSG00000196268.12	ZNF493	284443	-1.023658092	1.70504E-08	1.00049E-07
2409	ENSG00000156282.5	CLDN17	26285	-2.599262804	1.72339E-08	1.01066E-07
2410	ENSG00000267038.1			-5.483426222	1.74657E-08	1.02334E-07
2411	ENSG00000249203.2			-2.837786975	1.75189E-08	1.02625E-07
2412	ENSG00000112414.15	ADGRG6	57211	-1.056893408	1.75593E-08	1.02838E-07
2413	ENSG00000257277.2			-2.004491441	1.76927E-08	1.03573E-07
2414	ENSG00000197647.11	ZNF433	163059	-1.14608359	1.77373E-08	1.03818E-07
2415	ENSG00000125730.17	C3	718	-1.253126307	1.7934E-08	1.04939E-07
2416	ENSG00000224613.7	RNPC3-DT	101928436	-1.894680095	1.79694E-08	1.0513E-07
2417	ENSG00000286661.1			-2.954129494	1.80209E-08	1.05416E-07
2418	ENSG00000244699.1	EVA1CP5		-3.459021286	1.80727E-08	1.05688E-07
2419	ENSG00000255248.9	MIR100HG	399959	-1.026230711	1.8078E-08	1.05703E-07
2420	ENSG00000188992.11	LIPI	149998	-1.631079694	1.83339E-08	1.07105E-07
2421	ENSG00000255507.6			-1.232105743	1.84397E-08	1.07675E-07
2422	ENSG00000185652.12	NTF3	4908	-1.58239953	1.85229E-08	1.0813E-07
2423	ENSG00000261795.1			-1.687489182	1.87224E-08	1.09246E-07
2424	ENSG00000043591.6	ADRB1	153	-1.411540657	1.91838E-08	1.11823E-07
2425	ENSG00000071991.9	CDH19	28513	-2.127138371	1.94999E-08	1.13565E-07
2426	ENSG00000279013.1			-2.421169076	1.9891E-08	1.15758E-07
2427	ENSG00000258531.2	BANF1P1		-1.708131247	2.06133E-08	1.19816E-07
2428	ENSG00000188732.11	FAM221A	340277	-1.195188529	2.06155E-08	1.19816E-07
2429	ENSG00000250596.2			-1.906244917	2.07491E-08	1.20504E-07
2430	ENSG00000149043.16	SYT8	90019	-1.762578088	2.16984E-08	1.25686E-07
2431	ENSG00000189367.15	KIAA0408	9729	-2.392932999	2.19279E-08	1.2696E-07
2432	ENSG00000145708.11	CRHBP	1393	-1.508105038	2.23594E-08	1.29269E-07
2433	ENSG00000273771.1			-1.38483755	2.26785E-08	1.30999E-07
2434	ENSG00000218682.1			-1.529583231	2.28048E-08	1.31633E-07
2435	ENSG00000130701.4	RBBP8NL	140893	-1.130131638	2.32058E-08	1.3385E-07
2436	ENSG00000244753.2	RPL15P21		-2.052715658	2.38337E-08	1.37253E-07
2437	ENSG00000196376.11	SLC35F1	222553	-1.468690538	2.39112E-08	1.37639E-07
2438	ENSG00000198157.11	HMGN5	79366	-1.091439774	2.45952E-08	1.4133E-07
2439	ENSG00000166183.16	ASPG	374569	-1.523046418	2.50767E-08	1.43909E-07
2440	ENSG00000132846.6	ZBED3	84327	-1.057469626	2.54389E-08	1.45924E-07
2441	ENSG00000286970.1			-1.166649562	2.54949E-08	1.46203E-07
2442	ENSG00000279484.2	KLHL30-AS1		-2.105541684	2.65958E-08	1.5223E-07
2443	ENSG00000107317.13	PTGDS	5730	-1.5019545	2.67688E-08	1.53154E-07
2444	ENSG00000261520.5	DLGAP1-AS5		-3.307785083	2.69534E-08	1.54143E-07
2445	ENSG00000288047.1			-3.68387752	2.72766E-08	1.55812E-07
2446	ENSG00000287774.1			-2.028833032	2.72973E-08	1.55908E-07
2447	ENSG00000146038.12	DCDC2	51473	-1.654701739	2.73055E-08	1.55932E-07
2448	ENSG00000140284.11	SLC27A2	11001	-1.475462629	2.73838E-08	1.56334E-07
2449	ENSG00000288073.1			-2.868130392	2.78483E-08	1.58849E-07
2450	ENSG00000105427.10	CNFN	84518	-1.813590257	2.8805E-08	1.64141E-07
2451	ENSG00000259420.5		105370906	-2.581629641	2.90592E-08	1.6547E-07
2452	ENSG00000147724.12	FAM135B	51059	-1.692935519	2.98605E-08	1.6974E-07
2453	ENSG00000226124.8	FTCDNL1	348751	-1.077450197	3.01628E-08	1.71385E-07
2454	ENSG00000244094.2	SPRR2F	6705	-2.251389271	3.03047E-08	1.72117E-07
2455	ENSG00000091482.8	SMPX	23676	-2.936492107	3.08621E-08	1.75082E-07
2456	ENSG00000064309.14	CDON	50937	-1.077614554	3.09545E-08	1.75557E-07
2457	ENSG00000253105.6			-1.886703262	3.14677E-08	1.78212E-07
2458	ENSG00000205018.2			-1.327769019	3.19029E-08	1.80471E-07
2459	ENSG00000163873.10	GRIK3	2899	-2.088742644	3.20303E-08	1.81165E-07
2460	ENSG00000250026.5	TMPRSS11BNL		-2.711387063	3.26733E-08	1.84644E-07
2461	ENSG00000188993.3	LRRC66	339977	-1.119723313	3.26927E-08	1.84728E-07
2462	ENSG00000198944.6	SOWAHA	134548	-1.714437675	3.28778E-08	1.85721E-07
2463	ENSG00000286695.1			-2.567007346	3.36997E-08	1.90201E-07
2464	ENSG00000123843.13	C4BPB	725	-1.780860577	3.40431E-08	1.91893E-07
2465	ENSG00000163485.17	ADORA1	134	-1.121744061	3.42573E-08	1.92936E-07
2466	ENSG00000156218.13	ADAMTSL3	57188	-1.557710938	3.4284E-08	1.93058E-07
2467	ENSG00000277619.1			-1.423999215	3.44898E-08	1.94162E-07
2468	ENSG00000167311.14	ART5	116969	-1.830840585	3.47329E-08	1.9542E-07
2469	ENSG00000159307.19	SCUBE1	80274	-1.189175955	3.47856E-08	1.95689E-07
2470	ENSG00000102837.7	OLFM4	10562	-2.597094119	3.49409E-08	1.96479E-07
2471	ENSG00000279943.1	FLJ38576	651430	-1.185535728	3.50646E-08	1.97146E-07
2472	ENSG00000176716.5	OR10AB1P		-1.830234885	3.53221E-08	1.98566E-07
2473	ENSG00000196196.3	HRCT1	646962	-1.48495499	3.56424E-08	2.00282E-07
2474	ENSG00000255690.3	TRIL	9865	-1.297557378	3.56786E-08	2.00428E-07
2475	ENSG00000160182.3	TFF1	7031	-3.471718988	3.57354E-08	2.00719E-07
2476	ENSG00000198771.11	RCSD1	92241	-1.018593809	3.58462E-08	2.01312E-07
2477	ENSG00000145700.10	ANKRD31	256006	-1.599580428	3.59022E-08	2.01598E-07
2478	ENSG00000144191.12	CNGA3	1261	-1.720323964	3.62952E-08	2.03603E-07
2479	ENSG00000278925.1			-1.486112072	3.67521E-08	2.05904E-07
2480	ENSG00000102043.16	MTMR8	55613	-1.3130839	3.72795E-08	2.08623E-07
2481	ENSG00000164855.16	TMEM184A	202915	-1.032592498	3.78892E-08	2.11885E-07
2482	ENSG00000248771.5	SMIM31	100505989	-3.309711446	3.83522E-08	2.14293E-07
2483	ENSG00000198796.7	ALPK2	115701	-1.363609477	3.87551E-08	2.16422E-07
2484	ENSG00000236819.2	LINC01563	101060544	-2.150835632	3.88722E-08	2.16955E-07
2485	ENSG00000184785.6	SMIM10	644538	-1.021435272	3.93195E-08	2.19297E-07
2486	ENSG00000134463.15	ECHDC3	79746	-1.42374019	3.98961E-08	2.22356E-07
2487	ENSG00000198734.12	F5	2153	-1.288518006	3.99937E-08	2.22806E-07
2488	ENSG00000172554.12	SNTG2	54221	-1.485011473	4.00481E-08	2.23078E-07
2489	ENSG00000124935.4	SCGB1D2	10647	-3.756994812	4.03781E-08	2.2479E-07
2490	ENSG00000223701.3	RAET1E-AS1	100652739	-1.719868047	4.10485E-08	2.28394E-07
2491	ENSG00000225940.7	C5orf67		-1.413767833	4.19297E-08	2.331E-07
2492	ENSG00000254063.2	DUXAP2		-2.389297039	4.34553E-08	2.41243E-07
2493	ENSG00000229699.1		101928009	-1.57546668	4.52692E-08	2.50997E-07
2494	ENSG00000189051.5	RNF222	643904	-1.775835203	4.53503E-08	2.51412E-07
2495	ENSG00000254352.1	C4orf46P3		-1.081993635	4.55508E-08	2.52417E-07
2496	ENSG00000118308.15	IRAG2	4033	-1.436802453	4.65757E-08	2.57772E-07
2497	ENSG00000115474.7	KCNJ13	3769	-2.254864562	4.69127E-08	2.59514E-07
2498	ENSG00000276308.1			-2.384484004	4.80395E-08	2.65318E-07
2499	ENSG00000143546.10	S100A8	6279	-1.578394164	4.85913E-08	2.68254E-07
2500	ENSG00000272690.6	LINC02018	107986100	-1.044582331	4.96394E-08	2.73696E-07
2501	ENSG00000228639.8	ROCR		-3.081898989	4.97448E-08	2.74125E-07
2502	ENSG00000197705.10	KLHL14	57565	-1.628834604	5.03825E-08	2.77446E-07
2503	ENSG00000167757.14	KLK11	11012	-1.452143863	5.19435E-08	2.85567E-07
2504	ENSG00000205696.4	ADARB2-AS1	642394	-2.339068034	5.29416E-08	2.90812E-07
2505	ENSG00000254550.1	OMP	4975	-1.605671589	5.36195E-08	2.94251E-07
2506	ENSG00000228058.2	LINC01736		-2.158642906	5.57855E-08	3.05081E-07
2507	ENSG00000172014.12	ANKRD20A4P		-2.019574599	5.66216E-08	3.09068E-07
2508	ENSG00000226087.2		105374588	-2.118077812	5.67341E-08	3.09628E-07
2509	ENSG00000250327.1	RPSAP70		-1.187351659	5.67645E-08	3.09752E-07
2510	ENSG00000135226.18	UGT2B28	54490	-3.372031671	5.75869E-08	3.13894E-07
2511	ENSG00000154734.16	ADAMTS1	9510	-1.103976687	5.77444E-08	3.14709E-07
2512	ENSG00000111046.4	MYF6	4618	-3.0871737	5.79426E-08	3.15702E-07
2513	ENSG00000169618.7	PROKR1	10887	-1.570235013	5.85615E-08	3.18812E-07
2514	ENSG00000133640.20	LRRIQ1	84125	-1.695414496	6.03345E-08	3.27969E-07
2515	ENSG00000241935.9	HOGA1	112817	-1.245544518	6.07099E-08	3.29919E-07
2516	ENSG00000069535.14	MAOB	4129	-1.616721774	6.13239E-08	3.32982E-07
2517	ENSG00000186675.7	MAGEE2	139599	-2.034325596	6.19019E-08	3.3589E-07
2518	ENSG00000287115.1	LINC02993		-1.470040258	6.20392E-08	3.36543E-07
2519	ENSG00000233930.4	KRTAP5-AS1	338651	-1.445881375	6.28788E-08	3.40911E-07
2520	ENSG00000204767.4	INSYN2B	100131897	-1.540163896	6.33407E-08	3.43134E-07
2521	ENSG00000140092.14	FBLN5	10516	-1.011405272	6.34441E-08	3.43647E-07
2522	ENSG00000269888.1			-1.540218447	6.36453E-08	3.4459E-07
2523	ENSG00000172817.4	CYP7B1	9420	-1.096094861	6.3653E-08	3.4459E-07
2524	ENSG00000226944.1	RNF207-AS1		-1.253849184	6.64191E-08	3.58878E-07
2525	ENSG00000236393.2	LINC03099	101927476	-1.406045415	6.67128E-08	3.60416E-07
2526	ENSG00000287481.1			-2.605060327	6.75591E-08	3.64591E-07
2527	ENSG00000228412.9	LNC-LBCS	100506885	-1.26130709	6.81417E-08	3.67585E-07
2528	ENSG00000254786.1			-1.909684681	6.84061E-08	3.68961E-07
2529	ENSG00000164512.18	ANKRD55	79722	-1.167288788	6.99018E-08	3.76721E-07
2530	ENSG00000197353.4	LYPD2	137797	-2.146729943	7.04204E-08	3.79464E-07
2531	ENSG00000261399.1	MAPK8IP3-AS1		-2.582231113	7.0841E-08	3.81575E-07
2532	ENSG00000109208.5	SMR3A	26952	-6.000842915	7.12976E-08	3.83826E-07
2533	ENSG00000137473.18	TTC29	83894	-2.732154282	7.26387E-08	3.90674E-07
2534	ENSG00000124466.9	LYPD3	27076	-1.115814925	7.27387E-08	3.91159E-07
2535	ENSG00000143845.15	ETNK2	55224	-1.101250535	7.33993E-08	3.94444E-07
2536	ENSG00000158246.8	TENT5B	115572	-1.297229699	7.51569E-08	4.0337E-07
2537	ENSG00000238087.3	FMO8P		-3.535421609	7.51619E-08	4.0337E-07
2538	ENSG00000276757.1	RN7SL192P		-1.147665399	7.57223E-08	4.06212E-07
2539	ENSG00000006740.17	ARHGAP44	9912	-1.0416429	7.72275E-08	4.13839E-07
2540	ENSG00000279699.1			-1.241429841	7.77466E-08	4.16508E-07
2541	ENSG00000223764.2	LINC02593	100130417	-1.420951879	7.89103E-08	4.22229E-07
2542	ENSG00000283703.2	VSIG10L2	338667	-2.052554513	7.93908E-08	4.24571E-07
2543	ENSG00000228063.2	LYPLAL1-DT	643723	-1.171527547	8.00627E-08	4.27818E-07
2544	ENSG00000152760.10	DYNLT5	200132	-1.072297448	8.08219E-08	4.317E-07
2545	ENSG00000151948.12	GLT1D1	144423	-1.213595035	8.08612E-08	4.31851E-07
2546	ENSG00000256029.6			-2.11003484	8.13442E-08	4.34255E-07
2547	ENSG00000133115.12	STOML3	161003	-2.123314137	8.20266E-08	4.37781E-07
2548	ENSG00000237928.6	NFIA-AS2	100996570	-1.424691985	8.24859E-08	4.39936E-07
2549	ENSG00000181585.7	TMIE	259236	-1.059777355	8.40058E-08	4.475E-07
2550	ENSG00000272823.1			-1.762017387	8.49184E-08	4.51997E-07
2551	ENSG00000136244.12	IL6	3569	-1.586291382	8.49643E-08	4.52181E-07
2552	ENSG00000152766.6	ANKRD22	118932	-1.063721201	8.52261E-08	4.53453E-07
2553	ENSG00000213316.10	LTC4S	4056	-1.019483087	8.59898E-08	4.57209E-07
2554	ENSG00000163121.10	NEURL3	93082	-1.401654877	8.66526E-08	4.60548E-07
2555	ENSG00000156689.7	GLYATL2	219970	-2.192367396	8.91788E-08	4.73404E-07
2556	ENSG00000102802.10	MEDAG	84935	-1.234160888	9.07907E-08	4.81187E-07
2557	ENSG00000286527.1			-1.654466251	9.09394E-08	4.81846E-07
2558	ENSG00000122735.16	DNAI1	27019	-2.357726248	9.11949E-08	4.83006E-07
2559	ENSG00000156968.9	MPV17L	255027	-1.501139651	9.2285E-08	4.88258E-07
2560	ENSG00000285960.1			-3.365448471	9.23874E-08	4.8873E-07
2561	ENSG00000085831.15	TTC39A	22996	-1.126156069	9.23988E-08	4.8873E-07
2562	ENSG00000152582.14	SPEF2	79925	-1.258038686	9.32343E-08	4.92952E-07
2563	ENSG00000154188.10	ANGPT1	284	-1.171984387	9.33953E-08	4.93737E-07
2564	ENSG00000009765.15	IYD	389434	-1.956070303	9.4546E-08	4.99288E-07
2565	ENSG00000169181.13	GSG1L	146395	-1.462028747	9.49118E-08	5.00953E-07
2566	ENSG00000287666.1		105369591	-1.975149969	9.49973E-08	5.0127E-07
2567	ENSG00000171368.12	TPPP	11076	-1.115261252	9.51341E-08	5.01859E-07
2568	ENSG00000266968.2			-1.608910685	9.76685E-08	5.14476E-07
2569	ENSG00000108602.18	ALDH3A1	218	-1.840762293	9.786E-08	5.15416E-07
2570	ENSG00000077009.13	NMRK2	27231	-3.027681408	9.88151E-08	5.20032E-07
2571	ENSG00000197408.10	CYP2B6	1555	-2.362309236	9.89014E-08	5.20348E-07
2572	ENSG00000120756.13	PLS1	5357	-1.10175677	9.91028E-08	5.21338E-07
2573	ENSG00000231419.6	LINC00689	154822	-2.013047243	9.9766E-08	5.24688E-07
2574	ENSG00000255021.1			-1.984252314	1.00046E-07	5.26071E-07
2575	ENSG00000280040.1	SNX7-DT		-1.230802465	1.00055E-07	5.26071E-07
2576	ENSG00000115828.17	QPCT	25797	-1.036733736	1.00239E-07	5.26896E-07
2577	ENSG00000152785.8	BMP3	651	-2.616376259	1.0055E-07	5.2832E-07
2578	ENSG00000157445.15	CACNA2D3	55799	-1.099418374	1.01961E-07	5.35169E-07
2579	ENSG00000167608.12	TMC4	147798	-1.19000941	1.03216E-07	5.41323E-07
2580	ENSG00000234286.1			-1.727621666	1.0426E-07	5.46581E-07
2581	ENSG00000225649.6		100506474	-1.297293438	1.05732E-07	5.53862E-07
2582	ENSG00000276972.1			-1.716680745	1.06088E-07	5.55545E-07
2583	ENSG00000135960.10	EDAR	10913	-1.428717716	1.07018E-07	5.59929E-07
2584	ENSG00000237238.3	BMS1P10		-1.511952101	1.07473E-07	5.62164E-07
2585	ENSG00000197838.5	CYP2A13	1553	-3.169609648	1.0778E-07	5.63559E-07
2586	ENSG00000064205.10	CCN5	8839	-1.718244507	1.08065E-07	5.64963E-07
2587	ENSG00000287189.1		107986669	-3.106454521	1.08904E-07	5.68975E-07
2588	ENSG00000081818.4	PCDHB4	56131	-1.236625419	1.12036E-07	5.84489E-07
2589	ENSG00000231424.3	FMO1-AS1		-1.267083704	1.13145E-07	5.89885E-07
2590	ENSG00000108878.5	CACNG1	786	-2.694378539	1.14586E-07	5.97005E-07
2591	ENSG00000173208.4	ABCD2	225	-1.312022374	1.14817E-07	5.98054E-07
2592	ENSG00000227107.1			-1.837261975	1.14896E-07	5.98384E-07
2593	ENSG00000163220.11	S100A9	6280	-1.430867144	1.15134E-07	5.99548E-07
2594	ENSG00000135480.16	KRT7	3855	-1.936295161	1.15761E-07	6.02496E-07
2595	ENSG00000145113.22	MUC4	4585	-2.124483318	1.19323E-07	6.19979E-07
2596	ENSG00000286191.1			-2.244367842	1.19866E-07	6.22552E-07
2597	ENSG00000263508.6			-2.058626297	1.20795E-07	6.26969E-07
2598	ENSG00000186510.12	CLCNKA	1187	-1.917800201	1.21537E-07	6.30652E-07
2599	ENSG00000184113.9	CLDN5	7122	-1.044759383	1.24928E-07	6.47315E-07
2600	ENSG00000143171.13	RXRG	6258	-1.702022869	1.26064E-07	6.52863E-07
2601	ENSG00000224106.1	CYP4F25P		-2.12999953	1.27856E-07	6.61451E-07
2602	ENSG00000275494.1			-1.036368346	1.28393E-07	6.64054E-07
2603	ENSG00000008323.15	PLEKHG6	55200	-1.138801213	1.29553E-07	6.69705E-07
2604	ENSG00000153234.15	NR4A2	4929	-1.277462068	1.29895E-07	6.71212E-07
2605	ENSG00000286692.1			-1.662488753	1.30144E-07	6.72413E-07
2606	ENSG00000175164.15	ABO	28	-1.602996103	1.30874E-07	6.75919E-07
2607	ENSG00000197632.9	SERPINB2	5055	-1.562700134	1.31266E-07	6.77767E-07
2608	ENSG00000157388.19	CACNA1D	776	-1.269797936	1.31642E-07	6.79621E-07
2609	ENSG00000254862.5	LGR4-AS1	105376671	-1.136654754	1.31681E-07	6.79731E-07
2610	ENSG00000253671.3			-1.375102766	1.32717E-07	6.84547E-07
2611	ENSG00000142583.18	SLC2A5	6518	-1.095405371	1.34005E-07	6.90738E-07
2612	ENSG00000258489.4		105370993	-2.380153465	1.34575E-07	6.93589E-07
2613	ENSG00000249494.6	DMXL1-DT		-1.209224811	1.37875E-07	7.0995E-07
2614	ENSG00000010932.17	FMO1	2326	-1.304036288	1.38481E-07	7.12792E-07
2615	ENSG00000121653.11	MAPK8IP1	9479	-1.065165437	1.38754E-07	7.14104E-07
2616	ENSG00000243284.1	VSIG8	391123	-2.396128277	1.41161E-07	7.25738E-07
2617	ENSG00000257817.2	TBX3-AS1	105370000	-1.733955581	1.43375E-07	7.36263E-07
2618	ENSG00000226051.8	ZNF503-AS1	253264	-1.593497682	1.43836E-07	7.38341E-07
2619	ENSG00000233070.1	ZFY-AS1		-2.25794869	1.44338E-07	7.40727E-07
2620	ENSG00000266936.1			-1.079465102	1.46877E-07	7.52878E-07
2621	ENSG00000223774.5			-2.012104454	1.47993E-07	7.58306E-07
2622	ENSG00000143333.7	RGS16	6004	-1.038040521	1.49657E-07	7.66041E-07
2623	ENSG00000124159.15	MATN4	8785	-1.33180134	1.51261E-07	7.73551E-07
2624	ENSG00000162951.11	LRRTM1	347730	-2.787465394	1.51299E-07	7.73644E-07
2625	ENSG00000135914.6	HTR2B	3357	-1.257930649	1.52743E-07	7.80528E-07
2626	ENSG00000287170.1			-1.119272656	1.53113E-07	7.82113E-07
2627	ENSG00000172901.20	LVRN	206338	-1.518837953	1.54268E-07	7.87203E-07
2628	ENSG00000260047.2	BCAP31P2		-1.869387989	1.5591E-07	7.95068E-07
2629	ENSG00000255364.1	SMILR		-1.280952605	1.56683E-07	7.98704E-07
2630	ENSG00000239614.1	HMGN1P7		-1.319200567	1.57035E-07	8.00394E-07
2631	ENSG00000172782.12	FADS6	283985	-1.907542325	1.60213E-07	8.15441E-07
2632	ENSG00000185499.16	MUC1	4582	-1.275373135	1.61344E-07	8.20989E-07
2633	ENSG00000115705.22	TPO	7173	-1.408409343	1.64272E-07	8.34815E-07
2634	ENSG00000267581.1			-1.618689077	1.64692E-07	8.36675E-07
2635	ENSG00000160307.10	S100B	6285	-1.076327348	1.66185E-07	8.43888E-07
2636	ENSG00000282304.1		128966597	-1.630338256	1.67042E-07	8.47479E-07
2637	ENSG00000214146.3	LINC02026	647323	-1.157477579	1.67386E-07	8.49005E-07
2638	ENSG00000185052.12	SLC24A3	57419	-1.087026256	1.70126E-07	8.62127E-07
2639	ENSG00000215217.7	CFAP90	134121	-1.862697841	1.70176E-07	8.62168E-07
2640	ENSG00000116652.6	DLEU2L		-1.109828421	1.70638E-07	8.64175E-07
2641	ENSG00000186377.8	CYP4X1	260293	-1.602974759	1.73114E-07	8.76041E-07
2642	ENSG00000227038.3	GTF2IP7		-1.4106438	1.77581E-07	8.97387E-07
2643	ENSG00000102466.16	FGF14	2259	-1.474685774	1.77806E-07	8.98298E-07
2644	ENSG00000224568.2	LINC01886	105373537	-2.213156986	1.78844E-07	9.03078E-07
2645	ENSG00000143847.15	PPFIA4	8497	-1.175491237	1.79785E-07	9.07485E-07
2646	ENSG00000105894.12	PTN	5764	-1.513666357	1.79931E-07	9.08107E-07
2647	ENSG00000145879.11	SPINK7	84651	-2.417530087	1.80343E-07	9.09837E-07
2648	ENSG00000257771.6	LINC02395	101927292	-1.860830047	1.82523E-07	9.19934E-07
2649	ENSG00000286339.1		107986524	-2.559131332	1.82823E-07	9.21296E-07
2650	ENSG00000286292.1			-1.077403265	1.857E-07	9.35082E-07
2651	ENSG00000255427.1	LINC02707		-2.522966146	1.91557E-07	9.63105E-07
2652	ENSG00000135299.17	ANKRD6	22881	-1.004227501	1.92544E-07	9.67454E-07
2653	ENSG00000198491.3		101929106	-2.640779314	1.93904E-07	9.73793E-07
2654	ENSG00000010438.17	PRSS3	5646	-1.867410807	1.95492E-07	9.80775E-07
2655	ENSG00000234141.1			-1.14103113	1.9558E-07	9.8109E-07
2656	ENSG00000278035.1			-1.206561359	1.97454E-07	9.89867E-07
2657	ENSG00000174950.11	CD164L2	388611	-1.267829032	1.97512E-07	9.90034E-07
2658	ENSG00000174326.14	SLC16A11	162515	-1.271829455	1.97564E-07	9.90165E-07
2659	ENSG00000188783.6	PRELP	5549	-1.44916795	1.98992E-07	9.96819E-07
2660	ENSG00000017427.17	IGF1	3479	-1.408726999	2.02131E-07	1.01217E-06
2661	ENSG00000182255.7	KCNA4	3739	-2.286686719	2.02132E-07	1.01217E-06
2662	ENSG00000007001.13	UPP2	151531	-1.327158176	2.07706E-07	1.03837E-06
2663	ENSG00000166592.12	RRAD	6236	-1.540605423	2.09065E-07	1.04477E-06
2664	ENSG00000140505.7	CYP1A2	1544	-2.537291865	2.09682E-07	1.04772E-06
2665	ENSG00000087250.9	MT3	4504	-2.186103291	2.10738E-07	1.05273E-06
2666	ENSG00000267072.2			-1.627576502	2.13122E-07	1.06411E-06
2667	ENSG00000233670.7	PIRT	644139	-2.607087928	2.17092E-07	1.08311E-06
2668	ENSG00000276251.1			-1.528161168	2.19184E-07	1.093E-06
2669	ENSG00000133665.13	DYDC2	84332	-1.879401665	2.21522E-07	1.10396E-06
2670	ENSG00000203506.5	RBMS3-AS2		-1.546654921	2.21708E-07	1.10475E-06
2671	ENSG00000138039.15	LHCGR	3973	-2.300882674	2.22324E-07	1.1074E-06
2672	ENSG00000261458.1			-1.774372164	2.26979E-07	1.1296E-06
2673	ENSG00000077092.19	RARB	5915	-1.269085956	2.27817E-07	1.13334E-06
2674	ENSG00000151090.20	THRB	7068	-1.024535474	2.29218E-07	1.13931E-06
2675	ENSG00000149300.10	C11orf52	91894	-1.937852779	2.29346E-07	1.1398E-06
2676	ENSG00000268416.1			-2.52251443	2.31273E-07	1.1488E-06
2677	ENSG00000204403.9	CASP12	100506742	-1.060709004	2.32647E-07	1.15505E-06
2678	ENSG00000229558.2	SACS-AS1	100506680	-2.178858939	2.36512E-07	1.17336E-06
2679	ENSG00000250343.1	STK32A-AS1		-1.992022023	2.42403E-07	1.20123E-06
2680	ENSG00000226496.3	LINC00323	284835	-1.223623595	2.49567E-07	1.2358E-06
2681	ENSG00000242107.2	LINC01100		-3.795663464	2.59098E-07	1.28028E-06
2682	ENSG00000101098.13	RIMS4	140730	-2.165428177	2.65017E-07	1.30757E-06
2683	ENSG00000077327.16	SPAG6	9576	-2.12102105	2.65064E-07	1.30764E-06
2684	ENSG00000198569.10	SLC34A3	142680	-1.367904025	2.6693E-07	1.31619E-06
2685	ENSG00000260542.1			-3.173113374	2.66971E-07	1.31623E-06
2686	ENSG00000072858.11	SIDT1	54847	-1.104015489	2.68847E-07	1.32499E-06
2687	ENSG00000065371.17	ROPN1	54763	-2.39452114	2.69378E-07	1.32744E-06
2688	ENSG00000163898.10	LIPH	200879	-1.290350518	2.86514E-07	1.40768E-06
2689	ENSG00000259916.1	AQP7B	100509620	-2.203896274	2.91786E-07	1.43234E-06
2690	ENSG00000172995.16	ARPP21	10777	-2.847637608	2.97325E-07	1.45755E-06
2691	ENSG00000249996.1	PPIC-AS1		-1.044509755	2.97753E-07	1.45928E-06
2692	ENSG00000235641.5	LINC00484	100129347	-1.283154692	2.99307E-07	1.46654E-06
2693	ENSG00000158488.16	CD1E	913	-1.277195039	3.01191E-07	1.4754E-06
2694	ENSG00000160111.14	CPAMD8	27151	-1.037929176	3.07719E-07	1.50515E-06
2695	ENSG00000205832.7	C16orf96	342346	-1.268218108	3.07777E-07	1.50525E-06
2696	ENSG00000271420.1			-1.243588838	3.10144E-07	1.51645E-06
2697	ENSG00000188467.11	SLC24A5	283652	-3.677222762	3.16037E-07	1.54374E-06
2698	ENSG00000142224.17	IL19	29949	-1.675833525	3.17433E-07	1.5498E-06
2699	ENSG00000287644.1			-1.35200711	3.22314E-07	1.57208E-06
2700	ENSG00000169704.5	GP9	2815	-2.132534735	3.24369E-07	1.58093E-06
2701	ENSG00000086205.18	FOLH1	2346	-1.21684726	3.2535E-07	1.58498E-06
2702	ENSG00000249125.2			-1.785958794	3.28396E-07	1.5986E-06
2703	ENSG00000101638.14	ST8SIA5	29906	-1.468655881	3.31031E-07	1.61044E-06
2704	ENSG00000186474.15	KLK12	43849	-1.937167202	3.31373E-07	1.6117E-06
2705	ENSG00000256969.2	LINC03141		-2.202342959	3.37717E-07	1.64054E-06
2706	ENSG00000270638.1			-1.143533117	3.39228E-07	1.64748E-06
2707	ENSG00000179542.16	SLITRK4	139065	-1.619093683	3.4329E-07	1.66578E-06
2708	ENSG00000283849.1			-1.166947001	3.4687E-07	1.68213E-06
2709	ENSG00000267784.1			-2.455696461	3.53984E-07	1.71495E-06
2710	ENSG00000233987.2			-3.317236148	3.55588E-07	1.7225E-06
2711	ENSG00000111879.20	FAM184A	79632	-1.264743523	3.58378E-07	1.73538E-06
2712	ENSG00000140835.10	CHST4	10164	-2.091065654	3.59021E-07	1.73786E-06
2713	ENSG00000254139.1	LINC03018	286178	-1.727141542	3.70308E-07	1.79075E-06
2714	ENSG00000287096.1			-2.143416244	3.74664E-07	1.81071E-06
2715	ENSG00000226005.5	LINC02660	101927904	-2.103361678	3.78187E-07	1.8265E-06
2716	ENSG00000253302.1	STAU2-AS1	100128126	-1.127319168	3.80378E-07	1.83631E-06
2717	ENSG00000258077.2			-1.432599325	3.82489E-07	1.84582E-06
2718	ENSG00000231863.2		102724087	-1.479440833	3.87161E-07	1.86746E-06
2719	ENSG00000230371.1	KARS1P2		-1.932177493	3.92852E-07	1.89307E-06
2720	ENSG00000085563.15	ABCB1	5243	-1.000890747	3.92937E-07	1.89325E-06
2721	ENSG00000138136.6	LBX1	10660	-2.404123889	3.93787E-07	1.89712E-06
2722	ENSG00000159713.11	TPPP3	51673	-1.157748765	3.93895E-07	1.8974E-06
2723	ENSG00000126217.21	MCF2L	23263	-1.005985233	3.95696E-07	1.90421E-06
2724	ENSG00000187193.9	MT1X	4501	-1.045552576	3.9574E-07	1.90421E-06
2725	ENSG00000105679.9	GAPDHS	26330	-1.883788369	3.99567E-07	1.92076E-06
2726	ENSG00000104313.20	EYA1	2138	-1.621390292	4.01626E-07	1.93019E-06
2727	ENSG00000165449.11	SLC16A9	220963	-1.309756993	4.13914E-07	1.98633E-06
2728	ENSG00000260645.2			-1.278967775	4.19571E-07	2.01034E-06
2729	ENSG00000233912.1			-1.080213532	4.21762E-07	2.01986E-06
2730	ENSG00000173431.2	RNASE8	122665	-3.19119277	4.26688E-07	2.0405E-06
2731	ENSG00000197497.11	ZNF665	79788	-1.097344281	4.29785E-07	2.05432E-06
2732	ENSG00000223458.2			-1.88753552	4.3466E-07	2.07612E-06
2733	ENSG00000215386.13	MIR99AHG	388815	-1.19013495	4.4416E-07	2.11945E-06
2734	ENSG00000166091.21	CMTM5	116173	-1.730133701	4.57584E-07	2.17921E-06
2735	ENSG00000099864.18	PALM	5064	-1.15886007	4.62436E-07	2.19951E-06
2736	ENSG00000264490.3			-2.867294895	4.64703E-07	2.2095E-06
2737	ENSG00000166770.11	ZNF667-AS1	100128252	-1.171970023	4.66125E-07	2.21573E-06
2738	ENSG00000286035.1			-1.300956538	4.6656E-07	2.21753E-06
2739	ENSG00000103355.13	PRSS33	260429	-2.133505609	4.68489E-07	2.22617E-06
2740	ENSG00000260249.3			-1.087188573	4.75116E-07	2.25603E-06
2741	ENSG00000272573.6	MUSTN1	389125	-1.908643895	4.78242E-07	2.26979E-06
2742	ENSG00000235772.1			-1.251921365	4.89237E-07	2.31809E-06
2743	ENSG00000170262.13	MRAP	56246	-1.278344961	4.90067E-07	2.32119E-06
2744	ENSG00000271714.1	FAM169A-AS1		-1.240965789	4.91299E-07	2.32647E-06
2745	ENSG00000231274.5	SBK3	100130827	-1.67268658	4.91693E-07	2.32806E-06
2746	ENSG00000262583.1	TMEM231P1		-1.192617974	4.95682E-07	2.3461E-06
2747	ENSG00000144596.13	GRIP2	80852	-1.082704042	4.97584E-07	2.3537E-06
2748	ENSG00000259038.1			-1.767059327	4.97697E-07	2.35395E-06
2749	ENSG00000285971.1			-2.689473651	5.00751E-07	2.36783E-06
2750	ENSG00000182853.12	VMO1	284013	-1.039708411	5.0201E-07	2.3735E-06
2751	ENSG00000283361.2	CFAP97D2	101929355	-2.508256743	5.04875E-07	2.38648E-06
2752	ENSG00000227544.10	LINC03013		-1.041691217	5.14785E-07	2.43071E-06
2753	ENSG00000233836.7			-1.528450065	5.16121E-07	2.43644E-06
2754	ENSG00000227128.5	LBX1-AS1		-2.00957728	5.19308E-07	2.45061E-06
2755	ENSG00000145626.12	UGT3A1	133688	-2.985139994	5.19731E-07	2.45232E-06
2756	ENSG00000134376.16	CRB1	23418	-1.579610553	5.42961E-07	2.55524E-06
2757	ENSG00000256723.1			-1.882018059	5.51209E-07	2.59098E-06
2758	ENSG00000148826.9	NKX6-2	84504	-2.676093703	5.52215E-07	2.59448E-06
2759	ENSG00000286986.1			-3.610145219	5.622E-07	2.64014E-06
2760	ENSG00000251015.1	SLC25A30-AS1		-1.259408573	5.63269E-07	2.64453E-06
2761	ENSG00000082175.15	PGR	5241	-1.54479978	5.6675E-07	2.65961E-06
2762	ENSG00000115423.19	DNAH6	1768	-1.343432642	5.6806E-07	2.66544E-06
2763	ENSG00000274092.1			-1.464055768	5.74947E-07	2.69617E-06
2764	ENSG00000162755.14	KLHDC9	126823	-1.232111109	5.77855E-07	2.70948E-06
2765	ENSG00000256609.1	HSPB8-AS1	107984440	-1.526647633	5.9064E-07	2.76648E-06
2766	ENSG00000137976.7	DNASE2B	58511	-2.220870342	5.91296E-07	2.76858E-06
2767	ENSG00000176532.4	PRR15	222171	-1.406427413	5.91556E-07	2.76947E-06
2768	ENSG00000255007.1	LINC02489		-2.896832312	5.97442E-07	2.79504E-06
2769	ENSG00000178343.5	SHISA3	152573	-2.036360962	6.09441E-07	2.84782E-06
2770	ENSG00000226869.6	LHFPL3-AS1	645591	-3.154392213	6.1198E-07	2.85934E-06
2771	ENSG00000232876.1		124901405	-1.242917265	6.18253E-07	2.88695E-06
2772	ENSG00000259342.1			-1.481583544	6.19819E-07	2.89358E-06
2773	ENSG00000227590.2	ATP5MC1P5		-2.124300978	6.25568E-07	2.91802E-06
2774	ENSG00000232872.2	CTAGE3P		-1.549910851	6.34768E-07	2.95815E-06
2775	ENSG00000128918.15	ALDH1A2	8854	-1.40415873	6.3792E-07	2.97179E-06
2776	ENSG00000179796.12	LRRC3B	116135	-2.322185551	6.5533E-07	3.04859E-06
2777	ENSG00000165309.14	ARMC3	219681	-2.401408745	6.55769E-07	3.05028E-06
2778	ENSG00000258933.1	LINC02279		-3.081089152	6.62949E-07	3.08006E-06
2779	ENSG00000272491.1			-1.387711811	6.69949E-07	3.11149E-06
2780	ENSG00000272701.3	MESTIT1	317751	-1.521576115	6.99437E-07	3.24161E-06
2781	ENSG00000181031.16	RPH3AL	9501	-1.046817863	7.0112E-07	3.24827E-06
2782	ENSG00000149654.11	CDH22	64405	-1.841103194	7.123E-07	3.2966E-06
2783	ENSG00000210194.1	MT-TE		-1.671667408	7.13285E-07	3.30039E-06
2784	ENSG00000230910.4	ADGRB3-DT		-1.746481017	7.1337E-07	3.3004E-06
2785	ENSG00000229035.2	SPRR2C		-1.806484444	7.1449E-07	3.30481E-06
2786	ENSG00000168916.15	ZNF608	57507	-1.003219332	7.24181E-07	3.34612E-06
2787	ENSG00000275585.2	PDE4DIPP4		-1.632370083	7.251E-07	3.34998E-06
2788	ENSG00000168589.15	DYNLRB2	83657	-1.215795581	7.32019E-07	3.38037E-06
2789	ENSG00000115592.11	PRKAG3	53632	-2.121797148	7.33918E-07	3.38874E-06
2790	ENSG00000131016.17	AKAP12	9590	-1.123037991	7.34411E-07	3.39062E-06
2791	ENSG00000228330.1			-1.102237739	7.55592E-07	3.48233E-06
2792	ENSG00000237642.1	HMGB3P5		-2.549552524	7.57439E-07	3.49003E-06
2793	ENSG00000256884.1			-1.850305969	7.60115E-07	3.50154E-06
2794	ENSG00000236508.1	ATP13A5-AS1		-2.247155545	7.70588E-07	3.5469E-06
2795	ENSG00000287381.1			-2.778977883	7.97746E-07	3.66298E-06
2796	ENSG00000253190.4			-1.475113624	7.97975E-07	3.66361E-06
2797	ENSG00000169302.16	STK32A	202374	-1.307787613	8.03329E-07	3.68605E-06
2798	ENSG00000139144.11	PIK3C2G	5288	-1.622157473	8.09077E-07	3.71023E-06
2799	ENSG00000133083.15	DCLK1	9201	-1.335347937	8.13353E-07	3.7273E-06
2800	ENSG00000251584.2			-1.762089988	8.14364E-07	3.73065E-06
2801	ENSG00000112319.19	EYA4	2070	-1.542182385	8.16941E-07	3.74159E-06
2802	ENSG00000077274.9	CAPN6	827	-2.049996954	8.43848E-07	3.85591E-06
2803	ENSG00000206432.4	TMEM200C	645369	-1.513905473	8.47189E-07	3.8685E-06
2804	ENSG00000287348.1			-2.453526695	8.67555E-07	3.95603E-06
2805	ENSG00000118271.12	TTR	7276	-2.844656386	8.72375E-07	3.97618E-06
2806	ENSG00000166257.9	SCN3B	55800	-1.158511934	8.80487E-07	4.01022E-06
2807	ENSG00000115461.5	IGFBP5	3488	-1.183068921	8.94728E-07	4.07151E-06
2808	ENSG00000234998.1			-1.566659945	8.9662E-07	4.07872E-06
2809	ENSG00000177112.8	IRAG1-AS1	100129827	-1.095006355	9.04676E-07	4.11253E-06
2810	ENSG00000287167.1			-1.006410431	9.33729E-07	4.23877E-06
2811	ENSG00000261572.1			-1.026930734	9.40979E-07	4.26924E-06
2812	ENSG00000081041.9	CXCL2	2920	-1.250761719	9.43137E-07	4.27805E-06
2813	ENSG00000272195.1	EFCAB2-AS1		-1.029319975	9.49087E-07	4.30307E-06
2814	ENSG00000236732.1	RPL21P137		-1.490062207	9.54122E-07	4.32491E-06
2815	ENSG00000236816.3	ANKRD20A7P		-1.973182787	9.59846E-07	4.34886E-06
2816	ENSG00000196344.11	ADH7	131	-1.852748532	9.83566E-07	4.45024E-06
2817	ENSG00000287603.1			-1.03528499	9.87217E-07	4.46522E-06
2818	ENSG00000124429.18	POF1B	79983	-1.386897602	1.00125E-06	4.52662E-06
2819	ENSG00000176601.13	MAP3K19	80122	-1.446090139	1.00807E-06	4.55537E-06
2820	ENSG00000269001.2	ZNF818P		-1.120610284	1.01108E-06	4.56845E-06
2821	ENSG00000148965.10	SAA4	6291	-1.843051404	1.01137E-06	4.56928E-06
2822	ENSG00000276790.1			-2.056189904	1.01463E-06	4.58348E-06
2823	ENSG00000186466.5	AQP7P1		-1.619156923	1.03336E-06	4.66278E-06
2824	ENSG00000239922.2			-2.114802592	1.03802E-06	4.68114E-06
2825	ENSG00000286529.1			-1.167849883	1.04562E-06	4.71432E-06
2826	ENSG00000180999.11	C1orf105	92346	-2.109850449	1.05311E-06	4.74542E-06
2827	ENSG00000164684.13	ZNF704	619279	-1.028032406	1.07209E-06	4.82487E-06
2828	ENSG00000117983.17	MUC5B	727897	-2.846385947	1.10582E-06	4.96767E-06
2829	ENSG00000119715.15	ESRRB	2103	-1.670234418	1.12208E-06	5.03605E-06
2830	ENSG00000232560.7	LINC01549	100505929	-1.692927372	1.15077E-06	5.15675E-06
2831	ENSG00000214754.3	SRSF8CP		-2.578386932	1.15138E-06	5.15772E-06
2832	ENSG00000204001.9	LCN8	138307	-2.53364367	1.16869E-06	5.23055E-06
2833	ENSG00000272799.1			-1.111700363	1.17373E-06	5.25192E-06
2834	ENSG00000258837.1			-2.297724826	1.17852E-06	5.27216E-06
2835	ENSG00000262061.6	RPH3AL-AS1		-1.561599792	1.18019E-06	5.27845E-06
2836	ENSG00000237328.1	RAI1-AS1	100861516	-1.638175367	1.18897E-06	5.31591E-06
2837	ENSG00000254731.1			-1.663702085	1.19222E-06	5.32922E-06
2838	ENSG00000153802.12	TMPRSS11D	9407	-1.419268678	1.19547E-06	5.34254E-06
2839	ENSG00000226094.1	RPL7P3		-3.062008106	1.1971E-06	5.34925E-06
2840	ENSG00000152672.8	CLEC4F	165530	-1.342555296	1.19847E-06	5.35354E-06
2841	ENSG00000157152.17	SYN2	6854	-1.634803008	1.20281E-06	5.37173E-06
2842	ENSG00000287312.1			-1.643307635	1.2228E-06	5.45672E-06
2843	ENSG00000231359.3	RPL31P34		-1.356313654	1.24406E-06	5.5416E-06
2844	ENSG00000196542.8	SPTSSB	165679	-1.43952448	1.26212E-06	5.61635E-06
2845	ENSG00000288605.1			-2.596636306	1.2631E-06	5.62007E-06
2846	ENSG00000109255.11	NMU	10874	-1.222390293	1.26799E-06	5.63995E-06
2847	ENSG00000131910.5	NR0B2	8431	-1.424341946	1.27298E-06	5.66024E-06
2848	ENSG00000226770.1	LINC03012	100506682	-2.047483826	1.27785E-06	5.6787E-06
2849	ENSG00000124249.7	KCNK15	60598	-1.282806033	1.2809E-06	5.691E-06
2850	ENSG00000239899.3	RN7SL674P		-1.205546557	1.30182E-06	5.78004E-06
2851	ENSG00000266256.3	LINC00908	284276	-1.209188934	1.31728E-06	5.84409E-06
2852	ENSG00000165383.11	LRRC18	474354	-1.872002127	1.32966E-06	5.8951E-06
2853	ENSG00000279526.1			-1.220603551	1.33372E-06	5.91243E-06
2854	ENSG00000240240.9			-1.904515441	1.37398E-06	6.07661E-06
2855	ENSG00000224093.5	BCAR3-AS1		-1.219921541	1.38229E-06	6.10522E-06
2856	ENSG00000280423.1			-1.858726255	1.38316E-06	6.10837E-06
2857	ENSG00000136011.15	STAB2	55576	-1.347620385	1.39122E-06	6.14189E-06
2858	ENSG00000156284.6	CLDN8	9073	-2.212087412	1.39217E-06	6.14541E-06
2859	ENSG00000166573.6	GALR1	2587	-1.452484751	1.44117E-06	6.34478E-06
2860	ENSG00000140600.17	SH3GL3	6457	-1.119092542	1.44187E-06	6.34712E-06
2861	ENSG00000234336.7	JAZF1-AS1		-1.143250895	1.44411E-06	6.35559E-06
2862	ENSG00000229116.3			-1.670442932	1.44545E-06	6.36077E-06
2863	ENSG00000258630.3	LINC02292	101929080	-2.078214559	1.4498E-06	6.37899E-06
2864	ENSG00000116690.13	PRG4	10216	-1.727499851	1.48877E-06	6.54052E-06
2865	ENSG00000253573.2			-1.272305792	1.48944E-06	6.54208E-06
2866	ENSG00000198758.11	EPS8L3	79574	-2.289119468	1.50248E-06	6.59782E-06
2867	ENSG00000267206.6	LCN6	158062	-2.233830219	1.51549E-06	6.64614E-06
2868	ENSG00000104953.20	TLE6	79816	-1.085793388	1.53702E-06	6.73458E-06
2869	ENSG00000144619.15	CNTN4	152330	-1.337646407	1.57416E-06	6.8897E-06
2870	ENSG00000234338.1			-1.036880471	1.5776E-06	6.90402E-06
2871	ENSG00000144891.18	AGTR1	185	-1.575746587	1.58451E-06	6.93196E-06
2872	ENSG00000066230.12	SLC9A3	6550	-1.225415713	1.59429E-06	6.97012E-06
2873	ENSG00000184388.6	PABPC1L2B	645974	-3.82153039	1.59652E-06	6.97757E-06
2874	ENSG00000174776.11	WDR49	151790	-2.017493582	1.60301E-06	7.00362E-06
2875	ENSG00000250522.1			-3.165657176	1.63648E-06	7.14198E-06
2876	ENSG00000234553.1			-1.582168645	1.69585E-06	7.38968E-06
2877	ENSG00000233705.7	SLC26A4-AS1		-1.873925282	1.70424E-06	7.42462E-06
2878	ENSG00000181072.11	CHRM2	1129	-1.726260439	1.71766E-06	7.47731E-06
2879	ENSG00000178826.11	TMEM139	135932	-1.696457643	1.71926E-06	7.48345E-06
2880	ENSG00000131126.19	TEX101	83639	-1.533953165	1.75662E-06	7.63853E-06
2881	ENSG00000267938.1	EIF1P6		-1.073035236	1.76351E-06	7.6668E-06
2882	ENSG00000262801.6			-2.368519838	1.77672E-06	7.72002E-06
2883	ENSG00000215875.4	ST13P20		-1.467420505	1.80737E-06	7.84118E-06
2884	ENSG00000234756.1	LINC02621		-1.725304997	1.81179E-06	7.85862E-06
2885	ENSG00000254799.1	SLC25A47P1		-2.647564608	1.88063E-06	8.12963E-06
2886	ENSG00000248586.2			-2.104249057	1.89022E-06	8.16933E-06
2887	ENSG00000132259.13	CNGA4	1262	-1.169796221	1.89256E-06	8.17674E-06
2888	ENSG00000157703.16	SVOPL	136306	-1.58780555	1.9127E-06	8.25659E-06
2889	ENSG00000127252.7	PLAAT1	57110	-1.362656436	1.92766E-06	8.31753E-06
2890	ENSG00000185960.14	SHOX	6473	-2.150412987	1.979E-06	8.52419E-06
2891	ENSG00000255039.1	LINC02553		-4.749639707	1.98893E-06	8.56512E-06
2892	ENSG00000196096.3	SPAG16-DT		-1.555302903	2.03261E-06	8.74278E-06
2893	ENSG00000287776.1			-2.901614696	2.03328E-06	8.7447E-06
2894	ENSG00000249601.2	LINC01187		-2.665794941	2.041E-06	8.77698E-06
2895	ENSG00000204173.11	LRRC37A5P		-1.03823371	2.17943E-06	9.34086E-06
2896	ENSG00000286201.1			-2.33063155	2.20169E-06	9.43118E-06
2897	ENSG00000272279.1		102723944	-1.249407099	2.20557E-06	9.44471E-06
2898	ENSG00000106034.18	CPED1	79974	-1.113766334	2.20844E-06	9.45599E-06
2899	ENSG00000275155.1			-1.654889587	2.25068E-06	9.63374E-06
2900	ENSG00000003989.18	SLC7A2	6542	-1.286118687	2.2762E-06	9.73882E-06
2901	ENSG00000231768.2	LINC01354	100506795	-1.36937608	2.28604E-06	9.77665E-06
2902	ENSG00000275320.1			-3.072181366	2.33181E-06	9.96162E-06
2903	ENSG00000249119.1	MTND6P4		-1.062997583	2.37476E-06	1.01309E-05
2904	ENSG00000241794.2	SPRR2A	6700	-1.600854728	2.39283E-06	1.02025E-05
2905	ENSG00000261671.1			-1.093318954	2.39953E-06	1.02278E-05
2906	ENSG00000242052.1	RPL10P7		-1.166168152	2.43249E-06	1.03616E-05
2907	ENSG00000118402.6	ELOVL4	6785	-1.174505872	2.48488E-06	1.05745E-05
2908	ENSG00000181997.8	AQP7P2		-2.64324256	2.49343E-06	1.06075E-05
2909	ENSG00000165478.7	HEPACAM	220296	-2.286329588	2.50185E-06	1.06422E-05
2910	ENSG00000078549.15	ADCYAP1R1	117	-1.258627908	2.53352E-06	1.07677E-05
2911	ENSG00000226101.2	LINC02097	101928205	-1.936063156	2.54047E-06	1.0796E-05
2912	ENSG00000288079.1			-1.225088542	2.54185E-06	1.08008E-05
2913	ENSG00000232077.2	LINC01031	101929184	-1.106392288	2.56084E-06	1.08768E-05
2914	ENSG00000287965.1			-1.115326269	2.64292E-06	1.1199E-05
2915	ENSG00000080572.13	DNAAF6	139212	-2.600266491	2.69984E-06	1.14146E-05
2916	ENSG00000139364.10	TMEM132B	114795	-1.088390329	2.73107E-06	1.15417E-05
2917	ENSG00000164393.8	ADGRF2P		-1.16935578	2.73844E-06	1.15716E-05
2918	ENSG00000248869.7	LINC02511	105377441	-1.948452733	2.78659E-06	1.17513E-05
2919	ENSG00000286645.1			-2.140412467	2.81906E-06	1.18781E-05
2920	ENSG00000105996.7	HOXA2	3199	-1.005541901	2.84442E-06	1.19798E-05
2921	ENSG00000150045.12	KLRF1	51348	-1.104263074	2.86146E-06	1.20452E-05
2922	ENSG00000232110.8			-1.247729419	2.88845E-06	1.21537E-05
2923	ENSG00000139549.4	DHH	50846	-1.059288349	2.90922E-06	1.22359E-05
2924	ENSG00000164946.20	FREM1	158326	-1.163537455	2.90982E-06	1.22371E-05
2925	ENSG00000162989.5	KCNJ3	3760	-1.905375616	2.92698E-06	1.23015E-05
2926	ENSG00000134121.10	CHL1	10752	-1.549699445	2.95975E-06	1.24313E-05
2927	ENSG00000101892.12	ATP1B4	23439	-2.527160165	2.9763E-06	1.24941E-05
2928	ENSG00000270876.1	ZNF30-AS1		-1.709607902	3.06504E-06	1.28449E-05
2929	ENSG00000284650.1		124904027	-2.794896576	3.08063E-06	1.29062E-05
2930	ENSG00000273409.3	LINC02712	101929497	-1.806428069	3.09234E-06	1.29538E-05
2931	ENSG00000239911.2	PRKAG2-AS1		-1.164507913	3.13065E-06	1.31047E-05
2932	ENSG00000276289.4		102723475	-1.813312328	3.13218E-06	1.31097E-05
2933	ENSG00000171595.14	DNAI2	64446	-1.386242568	3.13392E-06	1.31142E-05
2934	ENSG00000165105.10	RASEF	158158	-1.03883847	3.14289E-06	1.31503E-05
2935	ENSG00000240023.1	RPLP0P3		-1.027131667	3.14616E-06	1.31626E-05
2936	ENSG00000275395.6	FCGBP	8857	-1.318573703	3.17732E-06	1.32874E-05
2937	ENSG00000263586.1	HID1-AS1	102723641	-1.090670863	3.19022E-06	1.33371E-05
2938	ENSG00000286705.1			-1.055205297	3.27837E-06	1.36898E-05
2939	ENSG00000263368.1	MSANTD3P1		-1.584401768	3.27928E-06	1.36921E-05
2940	ENSG00000237169.1	RPL12P27		-1.187696236	3.31276E-06	1.38217E-05
2941	ENSG00000261632.1		107984714	-1.499957697	3.31497E-06	1.38295E-05
2942	ENSG00000165966.16	PDZRN4	29951	-1.484686031	3.34846E-06	1.39618E-05
2943	ENSG00000287599.1			-1.334612251	3.38785E-06	1.41157E-05
2944	ENSG00000163710.9	PCOLCE2	26577	-1.443145178	3.45975E-06	1.4391E-05
2945	ENSG00000266916.7	ZNF793-AS1	101927720	-1.133112076	3.51679E-06	1.4613E-05
2946	ENSG00000152463.15	OLAH	55301	-1.436783424	3.72639E-06	1.54217E-05
2947	ENSG00000278683.1			-2.028006454	3.7333E-06	1.54463E-05
2948	ENSG00000229976.2			-1.642200531	3.73856E-06	1.54664E-05
2949	ENSG00000162444.12	RBP7	116362	-1.125238915	3.74874E-06	1.55015E-05
2950	ENSG00000229891.2	LINC01315	102723775	-1.262829011	3.78166E-06	1.5629E-05
2951	ENSG00000175920.18	DOK7	285489	-1.238438027	3.80494E-06	1.57115E-05
2952	ENSG00000231160.10	KLF3-AS1		-1.002729967	3.81953E-06	1.57701E-05
2953	ENSG00000149124.11	GLYAT	10249	-1.688296876	3.82804E-06	1.58036E-05
2954	ENSG00000188833.10	ENTPD8	377841	-1.868123857	3.84183E-06	1.5854E-05
2955	ENSG00000203782.6	LORICRIN	4014	-2.360919699	3.86113E-06	1.5922E-05
2956	ENSG00000185008.17	ROBO2	6092	-1.467586136	3.95925E-06	1.63029E-05
2957	ENSG00000233220.3	PRDM10-DT	440072	-1.059285176	4.05469E-06	1.66544E-05
2958	ENSG00000116031.9	CD207	50489	-1.296193734	4.05626E-06	1.66573E-05
2959	ENSG00000164093.17	PITX2	5308	-1.33351986	4.09909E-06	1.68175E-05
2960	ENSG00000166391.15	MOGAT2	80168	-1.894415452	4.15569E-06	1.70357E-05
2961	ENSG00000230285.1			-1.950827401	4.15791E-06	1.7043E-05
2962	ENSG00000166828.3	SCNN1G	6340	-1.277004397	4.16275E-06	1.70611E-05
2963	ENSG00000132465.12	JCHAIN	3512	-1.538506402	4.17115E-06	1.7092E-05
2964	ENSG00000173908.9	KRT28	162605	-3.91636568	4.20016E-06	1.72055E-05
2965	ENSG00000265460.6			-1.932126097	4.21706E-06	1.72694E-05
2966	ENSG00000118492.17	ADGB	79747	-1.965949303	4.33066E-06	1.77053E-05
2967	ENSG00000168515.3	SCGB1D1	10648	-5.137718988	4.33152E-06	1.7707E-05
2968	ENSG00000085276.19	MECOM	2122	-1.010421692	4.39507E-06	1.79483E-05
2969	ENSG00000188803.15	SHISA6	388336	-1.642209047	4.453E-06	1.81737E-05
2970	ENSG00000286698.1			-2.501058395	4.50892E-06	1.83867E-05
2971	ENSG00000250230.3		101927495	-1.485247714	4.51847E-06	1.84238E-05
2972	ENSG00000128655.18	PDE11A	50940	-1.773574024	4.53501E-06	1.84855E-05
2973	ENSG00000279419.1			-1.0744201	4.54586E-06	1.85278E-05
2974	ENSG00000260691.6	ANKRD20A1	84210	-1.38284598	4.5816E-06	1.86563E-05
2975	ENSG00000261713.6	SSTR5-AS1	146336	-1.938373346	4.64917E-06	1.89139E-05
2976	ENSG00000237515.9	SHISA9	729993	-1.938861473	4.65961E-06	1.89544E-05
2977	ENSG00000259974.3	LINC00261	140828	-2.252960194	4.66759E-06	1.89811E-05
2978	ENSG00000263639.7	MSMB	4477	-2.070698347	4.67247E-06	1.89989E-05
2979	ENSG00000162595.7	DIRAS3	9077	-1.025990825	4.73337E-06	1.92328E-05
2980	ENSG00000224157.1	HCG14	414760	-1.386544287	4.79282E-06	1.94624E-05
2981	ENSG00000277235.1			-1.469370319	4.79438E-06	1.94667E-05
2982	ENSG00000185869.15	ZNF829	374899	-1.010560064	4.82388E-06	1.95805E-05
2983	ENSG00000012504.15	NR1H4	9971	-1.823035276	4.82573E-06	1.9586E-05
2984	ENSG00000249242.8	TMEM150C	441027	-1.022693476	4.8755E-06	1.97718E-05
2985	ENSG00000224239.1			-1.938974475	4.90238E-06	1.98726E-05
2986	ENSG00000269113.4	TRABD2B	388630	-1.03611031	5.02473E-06	2.03437E-05
2987	ENSG00000280924.1	LINC00628	127841	-1.395985731	5.0262E-06	2.03475E-05
2988	ENSG00000233029.3			-1.388288645	5.03004E-06	2.03605E-05
2989	ENSG00000285714.1		124903185	-1.408978484	5.05224E-06	2.04446E-05
2990	ENSG00000265485.7	LINC01915	729950	-1.142586751	5.05945E-06	2.04717E-05
2991	ENSG00000233334.3	FAM53B-AS1		-1.256925404	5.069E-06	2.05063E-05
2992	ENSG00000178662.16	CSRNP3	80034	-1.034791441	5.07074E-06	2.05111E-05
2993	ENSG00000188581.9	KRTAP1-1	81851	-2.418297289	5.07444E-06	2.0524E-05
2994	ENSG00000007908.16	SELE	6401	-1.330198676	5.19271E-06	2.09595E-05
2995	ENSG00000170370.12	EMX2	2018	-1.238974645	5.35852E-06	2.16089E-05
2996	ENSG00000253474.2			-2.748464023	5.57132E-06	2.24057E-05
2997	ENSG00000148677.7	ANKRD1	27063	-2.102577657	5.57238E-06	2.24076E-05
2998	ENSG00000153923.10	CLCA3P		-1.272234238	5.61379E-06	2.25581E-05
2999	ENSG00000260918.1			-1.456620175	5.74446E-06	2.30365E-05
3000	ENSG00000177459.11	ERICH5	203111	-1.556022745	5.77141E-06	2.31374E-05
3001	ENSG00000279675.1			-1.578867422	5.77195E-06	2.31374E-05
3002	ENSG00000287048.1			-2.081988875	5.78002E-06	2.31674E-05
3003	ENSG00000179913.11	B3GNT3	10331	-1.013279025	5.89217E-06	2.35859E-05
3004	ENSG00000232287.2	SLC6A1-AS1	100874090	-1.993304057	5.91327E-06	2.36656E-05
3005	ENSG00000229019.1			-2.309508554	5.95934E-06	2.38331E-05
3006	ENSG00000253711.1			-2.122548933	5.96508E-06	2.38537E-05
3007	ENSG00000254040.2			-1.892721246	5.97836E-06	2.3902E-05
3008	ENSG00000172828.13	CES3	23491	-1.19564158	6.00035E-06	2.39827E-05
3009	ENSG00000277288.5	LINC02881		-1.983445349	6.04992E-06	2.41704E-05
3010	ENSG00000165841.11	CYP2C19	1557	-1.457278963	6.11123E-06	2.44061E-05
3011	ENSG00000196867.8	ZFP28	140612	-1.001544003	6.16027E-06	2.45871E-05
3012	ENSG00000233860.2	SHROOM3-AS1		-1.206101741	6.32864E-06	2.52109E-05
3013	ENSG00000286788.1			-1.975211187	6.3993E-06	2.54642E-05
3014	ENSG00000235070.3			-2.387450054	6.4376E-06	2.56012E-05
3015	ENSG00000237115.2			-1.523907604	6.50435E-06	2.58537E-05
3016	ENSG00000245864.3	MEF2C-AS2	109729137	-1.560231751	6.61058E-06	2.62628E-05
3017	ENSG00000172482.5	AGXT	189	-1.732792589	6.66891E-06	2.64831E-05
3018	ENSG00000165092.13	ALDH1A1	216	-1.56352324	6.69606E-06	2.65784E-05
3019	ENSG00000173610.12	UGT2A1	10941	-2.813034743	6.70471E-06	2.66075E-05
3020	ENSG00000285684.1			-1.857313943	6.70939E-06	2.66234E-05
3021	ENSG00000179799.9	OR7E22P		-1.582908916	6.72195E-06	2.66705E-05
3022	ENSG00000259932.1			-1.440558182	6.75652E-06	2.67916E-05
3023	ENSG00000235902.1			-1.759728195	6.78606E-06	2.68953E-05
3024	ENSG00000205890.3		100128770	-1.107809831	6.90209E-06	2.73333E-05
3025	ENSG00000189316.3		441239	-1.44906319	6.96012E-06	2.75411E-05
3026	ENSG00000166407.14	LMO1	4004	-1.342383928	7.12096E-06	2.81466E-05
3027	ENSG00000250425.1	OR7E35P		-1.876049779	7.12686E-06	2.81671E-05
3028	ENSG00000198010.13	DLGAP2	9228	-1.218183181	7.16167E-06	2.82963E-05
3029	ENSG00000182508.14	LHFPL1	340596	-1.58703312	7.21025E-06	2.84768E-05
3030	ENSG00000204616.11	TRIM31	11074	-1.231674792	7.23016E-06	2.85527E-05
3031	ENSG00000124920.14	MYRF	745	-1.095544813	7.23711E-06	2.85773E-05
3032	ENSG00000203685.10	STUM	375057	-1.169767203	7.36076E-06	2.90279E-05
3033	ENSG00000240654.6	C1QTNF9	338872	-2.133509708	7.40006E-06	2.91684E-05
3034	ENSG00000280304.1			-1.114244494	7.49164E-06	2.95E-05
3035	ENSG00000073734.10	ABCB11	8647	-1.247279448	7.61009E-06	2.99397E-05
3036	ENSG00000287885.1		105371272	-1.573504333	7.61447E-06	2.9954E-05
3037	ENSG00000280323.1			-2.617113427	7.66312E-06	3.01364E-05
3038	ENSG00000226601.1			-1.719401558	7.70228E-06	3.02666E-05
3039	ENSG00000242136.1			-1.120437879	7.83917E-06	3.07555E-05
3040	ENSG00000100170.10	SLC5A1	6523	-1.355820028	7.88829E-06	3.0936E-05
3041	ENSG00000135218.19	CD36	948	-1.237147678	8.28842E-06	3.24122E-05
3042	ENSG00000256340.8	ABCC6P1		-1.625634333	8.31941E-06	3.2527E-05
3043	ENSG00000196090.12	PTPRT	11122	-1.979459086	8.33266E-06	3.25756E-05
3044	ENSG00000281106.4	TMEM272	283521	-1.35710914	8.34129E-06	3.26061E-05
3045	ENSG00000169271.3	HSPB3	8988	-1.598078229	8.50461E-06	3.31758E-05
3046	ENSG00000112077.17	RHAG	6005	-1.86306028	8.52205E-06	3.32406E-05
3047	ENSG00000188536.13	HBA2	3040	-1.274572902	8.60558E-06	3.35367E-05
3048	ENSG00000254814.1			-1.368139737	8.72998E-06	3.40115E-05
3049	ENSG00000260604.2	TGILR	107986558	-1.527013656	8.89883E-06	3.46183E-05
3050	ENSG00000106436.7	MYL10	93408	-1.92938694	8.92536E-06	3.47147E-05
3051	ENSG00000236318.2	LINC02888		-2.049103857	8.99272E-06	3.49527E-05
3052	ENSG00000169313.10	P2RY12	64805	-1.210011041	9.03775E-06	3.5114E-05
3053	ENSG00000255650.6	FAM222A-AS1	84983	-1.168013564	9.10674E-06	3.53473E-05
3054	ENSG00000288040.1			-1.614838217	9.15888E-06	3.55184E-05
3055	ENSG00000232057.1			-2.232913729	9.16089E-06	3.55227E-05
3056	ENSG00000230882.1			-1.232909604	9.48975E-06	3.6701E-05
3057	ENSG00000254251.1		105375635	-2.252328883	9.5101E-06	3.67726E-05
3058	ENSG00000182195.9	LDOC1	23641	-1.119463924	9.60404E-06	3.71032E-05
3059	ENSG00000177202.3	SPACA4	171169	-1.036335389	9.78362E-06	3.77593E-05
3060	ENSG00000124253.11	PCK1	5105	-2.068362011	9.89331E-06	3.8139E-05
3061	ENSG00000147041.12	SYTL5	94122	-1.444563534	9.92999E-06	3.82693E-05
3062	ENSG00000231738.11	TSPAN19	144448	-2.037296527	9.97843E-06	3.84372E-05
3063	ENSG00000279685.2	MAPT-IT1	100130148	-2.020664552	1.01913E-05	3.92002E-05
3064	ENSG00000106123.12	EPHB6	2051	-1.004285755	1.02228E-05	3.93062E-05
3065	ENSG00000142494.13	SLC47A1	55244	-1.05694515	1.03196E-05	3.96475E-05
3066	ENSG00000287627.1			-2.555645463	1.03591E-05	3.97915E-05
3067	ENSG00000242640.1	RPS29P11		-1.793905094	1.04641E-05	4.01482E-05
3068	ENSG00000136267.14	DGKB	1607	-1.4333506	1.04678E-05	4.01583E-05
3069	ENSG00000251632.1	LINC02172	105377443	-1.497909473	1.04732E-05	4.01754E-05
3070	ENSG00000253875.2			-1.147640775	1.06115E-05	4.06545E-05
3071	ENSG00000162571.14	TTLL10	254173	-1.077978752	1.06223E-05	4.06922E-05
3072	ENSG00000171433.12	GLOD5	392465	-1.505691964	1.06374E-05	4.07403E-05
3073	ENSG00000267299.1	RFX2-AS2		-1.370508902	1.06779E-05	4.08773E-05
3074	ENSG00000286257.2			-1.613634779	1.07537E-05	4.11519E-05
3075	ENSG00000287156.1		124903317	-1.02995399	1.08713E-05	4.15658E-05
3076	ENSG00000078898.7	BPIFB2	80341	-3.67746292	1.0896E-05	4.16482E-05
3077	ENSG00000250831.1			-1.935808749	1.09465E-05	4.18291E-05
3078	ENSG00000171724.3	VAT1L	57687	-1.223139923	1.09477E-05	4.18294E-05
3079	ENSG00000260156.1			-1.301024365	1.10382E-05	4.21389E-05
3080	ENSG00000170624.13	SGCD	6444	-1.288923725	1.11798E-05	4.26299E-05
3081	ENSG00000243709.1	LEFTY1	10637	-1.341840288	1.14233E-05	4.34707E-05
3082	ENSG00000288575.1			-1.485753724	1.16223E-05	4.41644E-05
3083	ENSG00000277977.1			-1.049656289	1.17233E-05	4.45185E-05
3084	ENSG00000164122.9	ASB5	140458	-2.455810772	1.18805E-05	4.50463E-05
3085	ENSG00000111262.6	KCNA1	3736	-1.789566184	1.18987E-05	4.5111E-05
3086	ENSG00000112539.15	C6orf118	168090	-2.029808831	1.20087E-05	4.5502E-05
3087	ENSG00000287852.1			-2.061662812	1.21325E-05	4.59402E-05
3088	ENSG00000255277.3	ABCC6P2		-1.428752273	1.21923E-05	4.61449E-05
3089	ENSG00000199024.1	MIR103A2	406896	-1.059078609	1.24406E-05	4.70271E-05
3090	ENSG00000167992.13	VWCE	220001	-1.049236036	1.26065E-05	4.76117E-05
3091	ENSG00000228236.2	TXNP5		-1.238378147	1.27375E-05	4.80798E-05
3092	ENSG00000136110.13	CNMD	11061	-2.926895996	1.27842E-05	4.82377E-05
3093	ENSG00000225802.3	WARS1P1		-2.070603003	1.28841E-05	4.8585E-05
3094	ENSG00000206069.6	LHFPL7	255349	-2.153713039	1.32011E-05	4.96972E-05
3095	ENSG00000198183.12	BPIFA1	51297	-3.43803517	1.36043E-05	5.11229E-05
3096	ENSG00000162687.19	KCNT2	343450	-1.02974163	1.36204E-05	5.11786E-05
3097	ENSG00000260866.2			-3.898141517	1.37632E-05	5.16856E-05
3098	ENSG00000175985.10	PLEKHD1	400224	-1.077962824	1.38738E-05	5.20614E-05
3099	ENSG00000287754.1			-2.992782877	1.4156E-05	5.30101E-05
3100	ENSG00000233395.2	LINC00841	283033	-1.493921816	1.42646E-05	5.33867E-05
3101	ENSG00000257551.1	HLX-AS1	100873924	-1.621402491	1.45201E-05	5.42709E-05
3102	ENSG00000256189.1	DPPA4P3		-1.534999265	1.45699E-05	5.44418E-05
3103	ENSG00000267339.7	LINC00906	148145	-1.926920054	1.5098E-05	5.62561E-05
3104	ENSG00000273650.1			-1.546769534	1.52015E-05	5.66205E-05
3105	ENSG00000275540.1			-1.137575659	1.52105E-05	5.66432E-05
3106	ENSG00000254153.1	LINC02950	123466212	-1.244390991	1.56625E-05	5.819E-05
3107	ENSG00000104435.14	STMN2	11075	-1.618890653	1.60125E-05	5.94011E-05
3108	ENSG00000275756.1			-1.618304502	1.60552E-05	5.95485E-05
3109	ENSG00000137766.17	UNC13C	440279	-1.625525676	1.61924E-05	6.00405E-05
3110	ENSG00000134258.17	VTCN1	79679	-1.718596552	1.63222E-05	6.04936E-05
3111	ENSG00000286689.1			-1.087080119	1.64846E-05	6.10495E-05
3112	ENSG00000235049.1	LINC00940	100271702	-2.208507181	1.65324E-05	6.12152E-05
3113	ENSG00000249085.1	TNPO1-DT		-1.799932865	1.66564E-05	6.16284E-05
3114	ENSG00000249588.1			-2.114463691	1.67339E-05	6.18804E-05
3115	ENSG00000152932.8	RAB3C	115827	-1.24128864	1.70933E-05	6.31623E-05
3116	ENSG00000196353.11	CPNE4	131034	-1.52265674	1.71779E-05	6.34571E-05
3117	ENSG00000266559.1	MIR4530	100616163	-1.611233876	1.72667E-05	6.37556E-05
3118	ENSG00000282556.3		101927825	-1.161161783	1.79885E-05	6.62112E-05
3119	ENSG00000250978.5		101928819	-2.618721078	1.82991E-05	6.72668E-05
3120	ENSG00000270307.1	MTATP6P2		-1.026643923	1.90688E-05	6.99148E-05
3121	ENSG00000251576.1	LINC01267	101927565	-1.053929975	1.93246E-05	7.07808E-05
3122	ENSG00000277631.5	PGM5P3-AS1	101929127	-1.522382774	1.95086E-05	7.13822E-05
3123	ENSG00000167916.5	KRT24	192666	-2.238043197	1.97996E-05	7.24002E-05
3124	ENSG00000186766.8	FOXI2	399823	-1.864184203	1.99065E-05	7.27642E-05
3125	ENSG00000240520.6	UOX		-1.557452696	1.99286E-05	7.28317E-05
3126	ENSG00000260015.1			-2.141192432	2.00335E-05	7.31947E-05
3127	ENSG00000261839.1			-1.041708776	2.02763E-05	7.40202E-05
3128	ENSG00000134193.15	REG4	83998	-1.61211755	2.03135E-05	7.41494E-05
3129	ENSG00000237009.2	GLIS3-AS1	84850	-1.587971706	2.05191E-05	7.4838E-05
3130	ENSG00000240038.6	AMY2B	280	-1.043241785	2.0759E-05	7.5678E-05
3131	ENSG00000248753.1			-1.684915017	2.08015E-05	7.58049E-05
3132	ENSG00000254092.2			-1.563410294	2.08577E-05	7.59889E-05
3133	ENSG00000144410.5	CPO	130749	-1.062967641	2.10344E-05	7.65692E-05
3134	ENSG00000157856.12	DRC1	92749	-1.234935184	2.10939E-05	7.6779E-05
3135	ENSG00000168702.18	LRP1B	53353	-1.480693784	2.11998E-05	7.71362E-05
3136	ENSG00000269699.6	ZIM2	23619	-2.101211449	2.13462E-05	7.76131E-05
3137	ENSG00000255250.2			-1.441225907	2.15806E-05	7.84208E-05
3138	ENSG00000255240.6	GLYATL1-AS1	283194	-1.349874298	2.1815E-05	7.92507E-05
3139	ENSG00000198312.4	BMS1P9		-2.487168799	2.18771E-05	7.9462E-05
3140	ENSG00000259702.3			-1.982818632	2.19819E-05	7.98278E-05
3141	ENSG00000240409.1	MTATP8P1		-1.30343584	2.20654E-05	8.01165E-05
3142	ENSG00000164451.13	CALHM4	221301	-2.082774841	2.21267E-05	8.03315E-05
3143	ENSG00000196172.9	ZNF681	148213	-1.027525537	2.21666E-05	8.04558E-05
3144	ENSG00000188523.9	CFAP77	389799	-1.316390228	2.22761E-05	8.08223E-05
3145	ENSG00000239389.8	PCDHA13	56136	-2.530039267	2.23438E-05	8.10457E-05
3146	ENSG00000234754.2	LINC02817	400804	-1.342244926	2.23531E-05	8.10677E-05
3147	ENSG00000225386.2	LRRC3B-AS1		-2.602201155	2.23759E-05	8.11399E-05
3148	ENSG00000258422.5		646548	-2.009298282	2.27327E-05	8.23583E-05
3149	ENSG00000236197.4	PPP1R9A-AS1	105375405	-1.964883538	2.27552E-05	8.24248E-05
3150	ENSG00000226251.6	LINC02608	101929541	-1.283638342	2.29055E-05	8.29087E-05
3151	ENSG00000200378.1	RNU5B-4P		-1.119200876	2.30194E-05	8.32752E-05
3152	ENSG00000147606.9	SLC26A7	115111	-1.093690348	2.31524E-05	8.37105E-05
3153	ENSG00000283473.4	FAM240A	100132146	-2.646046378	2.31784E-05	8.37891E-05
3154	ENSG00000189001.11	SBSN	374897	-1.408888518	2.3221E-05	8.39307E-05
3155	ENSG00000235140.2			-1.434342599	2.32218E-05	8.39307E-05
3156	ENSG00000260807.7	CEROX1	115804232	-1.238632538	2.33335E-05	8.43115E-05
3157	ENSG00000257512.1			-2.051442069	2.33499E-05	8.4363E-05
3158	ENSG00000267557.1			-2.307498205	2.3688E-05	8.54367E-05
3159	ENSG00000272514.6	CFAP206	154313	-1.316710598	2.39025E-05	8.61555E-05
3160	ENSG00000214043.8	LINC02347	100128554	-1.889141358	2.39252E-05	8.62215E-05
3161	ENSG00000228522.2			-1.422730751	2.40273E-05	8.65503E-05
3162	ENSG00000287570.1			-1.449867785	2.41167E-05	8.68563E-05
3163	ENSG00000215218.4	UBE2QL1	134111	-1.053591662	2.41245E-05	8.68767E-05
3164	ENSG00000256916.1	MMP20-AS1		-2.00930383	2.42667E-05	8.73333E-05
3165	ENSG00000279796.1			-1.635255282	2.43775E-05	8.76841E-05
3166	ENSG00000125851.10	PCSK2	5126	-2.096603526	2.45832E-05	8.8376E-05
3167	ENSG00000118729.12	CASQ2	845	-1.756555249	2.46001E-05	8.84287E-05
3168	ENSG00000223343.1		124909376	-1.043961079	2.48332E-05	8.92064E-05
3169	ENSG00000174175.17	SELP	6403	-1.117547356	2.54177E-05	9.11777E-05
3170	ENSG00000245384.2	CXXC4-AS1	101929468	-1.212257471	2.5625E-05	9.18466E-05
3171	ENSG00000239715.1		105375115	-2.70365888	2.58075E-05	9.24589E-05
3172	ENSG00000232480.1	TGFB2-AS1		-1.379308541	2.61466E-05	9.35638E-05
3173	ENSG00000207944.1	MIR574	693159	-1.11488774	2.61889E-05	9.36983E-05
3174	ENSG00000226644.5	LINC03086		-1.352730591	2.66059E-05	9.50615E-05
3175	ENSG00000072133.11	RPS6KA6	27330	-1.659168909	2.66281E-05	9.51236E-05
3176	ENSG00000282996.1	MPP7-DT		-1.437488378	2.68622E-05	9.58994E-05
3177	ENSG00000107807.13	TLX1	3195	-2.204608634	2.69685E-05	9.62185E-05
3178	ENSG00000258776.1			-2.310665107	2.72056E-05	9.70469E-05
3179	ENSG00000182793.12	GSTA5	221357	-2.747205222	2.7249E-05	9.71842E-05
3180	ENSG00000180432.6	CYP8B1	1582	-1.088114493	2.74051E-05	9.77057E-05
3181	ENSG00000285671.1			-1.990874344	2.77134E-05	9.86717E-05
3182	ENSG00000274330.1			-1.878298901	2.77216E-05	9.8692E-05
3183	ENSG00000268654.2	MIMT1	100073347	-2.283957998	2.7874E-05	9.91723E-05
3184	ENSG00000271687.1	MTND5P10		-1.425936025	2.79972E-05	9.9566E-05
3185	ENSG00000271858.5		101928965	-1.174544338	2.85434E-05	0.000101356
3186	ENSG00000141431.12	ASXL3	80816	-1.171054793	2.85435E-05	0.000101356
3187	ENSG00000145309.6	CABS1	85438	-2.757395531	2.91084E-05	0.000103231
3188	ENSG00000249679.2			-1.05705928	2.92552E-05	0.000103705
3189	ENSG00000241595.2	KRTAP9-4	85280	-2.868273744	2.92612E-05	0.000103717
3190	ENSG00000196109.9	ZNF676	163223	-1.380098788	2.92847E-05	0.000103781
3191	ENSG00000186471.13	AKAP14	158798	-1.817378378	2.94784E-05	0.000104421
3192	ENSG00000158428.4	CATIP	375307	-1.282986602	2.96043E-05	0.000104849
3193	ENSG00000247627.2	MTND4P12		-1.297292335	3.02326E-05	0.000106912
3194	ENSG00000178828.7	RNF186	54546	-2.230873441	3.03246E-05	0.000107218
3195	ENSG00000258675.2	LINC02308		-1.560926278	3.04944E-05	0.000107789
3196	ENSG00000171954.13	CYP4F22	126410	-1.46085944	3.07009E-05	0.0001085
3197	ENSG00000287745.1			-1.988514901	3.09704E-05	0.000109365
3198	ENSG00000140274.14	DUOXA2	405753	-1.281033555	3.11549E-05	0.000109987
3199	ENSG00000224743.7	TEX26-AS1	100507064	-1.040329866	3.1376E-05	0.000110649
3200	ENSG00000105131.8	EPHX3	79852	-1.394106514	3.13811E-05	0.000110657
3201	ENSG00000138207.14	RBP4	5950	-1.648383673	3.21484E-05	0.000113192
3202	ENSG00000229221.1	HNRNPA1P66		-1.247654504	3.29397E-05	0.000115803
3203	ENSG00000112115.7	IL17A	3605	-1.587050567	3.3408E-05	0.000117345
3204	ENSG00000257127.6	CLLU1	574028	-1.142026932	3.35599E-05	0.000117869
3205	ENSG00000258171.2	LINC02412		-1.653099547	3.36566E-05	0.000118166
3206	ENSG00000273336.1	OR7M1P		-1.10629035	3.37136E-05	0.000118335
3207	ENSG00000130600.19	H19	283120	-1.372693371	3.3726E-05	0.000118368
3208	ENSG00000104112.9	SCG3	29106	-1.479795053	3.47395E-05	0.000121678
3209	ENSG00000199627.1	RNU6-1010P		-1.53802553	3.47677E-05	0.000121765
3210	ENSG00000259508.1			-1.834765576	3.49701E-05	0.00012242
3211	ENSG00000273118.1			-1.405914171	3.50556E-05	0.000122702
3212	ENSG00000229498.1			-1.458949794	3.58274E-05	0.000125167
3213	ENSG00000227838.1	CPMER		-1.280244428	3.60012E-05	0.000125709
3214	ENSG00000261489.1			-2.14401332	3.60015E-05	0.000125709
3215	ENSG00000255408.4	PCDHA3	56145	-1.381234092	3.67237E-05	0.000127971
3216	ENSG00000280216.1			-1.225361647	3.68097E-05	0.000128248
3217	ENSG00000197083.11	ZNF300P1		-1.02664149	3.749E-05	0.00013047
3218	ENSG00000206150.4	RNASE13	440163	-1.714281183	3.79264E-05	0.000131838
3219	ENSG00000166823.5	MESP1	55897	-1.1766152	3.83994E-05	0.000133342
3220	ENSG00000285918.1			-1.016701978	3.84931E-05	0.000133653
3221	ENSG00000183778.17	B3GALT5	10317	-1.674503261	3.93559E-05	0.000136496
3222	ENSG00000153930.12	ANKFN1	162282	-1.38496008	3.99496E-05	0.00013841
3223	ENSG00000286503.1			-1.080619309	4.03732E-05	0.000139755
3224	ENSG00000232306.2			-2.941121541	4.10605E-05	0.000141936
3225	ENSG00000234206.5			-1.736687047	4.24066E-05	0.000146232
3226	ENSG00000280233.1			-1.228773917	4.28542E-05	0.000147601
3227	ENSG00000253339.2			-2.668615195	4.31521E-05	0.000148558
3228	ENSG00000234512.1	TLR12P		-1.373512904	4.34497E-05	0.000149491
3229	ENSG00000147588.7	PMP2	5375	-1.637888773	4.35606E-05	0.000149821
3230	ENSG00000255974.8	CYP2A6	1548	-1.941577754	4.40495E-05	0.00015141
3231	ENSG00000197953.6	AADACL2	344752	-1.881593871	4.43356E-05	0.000152341
3232	ENSG00000173578.8	XCR1	2829	-1.02228577	4.5469E-05	0.000155992
3233	ENSG00000279754.1			-1.375144247	4.54877E-05	0.000156029
3234	ENSG00000150627.16	WDR17	116966	-1.313371391	4.58582E-05	0.000157232
3235	ENSG00000203963.11	C1orf141	400757	-1.944098193	4.62173E-05	0.000158299
3236	ENSG00000237159.6	CNTFR-AS1	415056	-1.8921404	4.66632E-05	0.000159702
3237	ENSG00000235205.1	TATDN2P3		-1.64454525	4.67039E-05	0.000159804
3238	ENSG00000246379.8	GNAO1-DT	283856	-1.03854955	4.67496E-05	0.000159929
3239	ENSG00000215771.2	LRRC37A14P		-1.112372821	4.72508E-05	0.000161546
3240	ENSG00000255362.1	LINC02761		-1.248628192	4.73231E-05	0.000161765
3241	ENSG00000169474.4	SPRR1A	6698	-1.321879328	4.74719E-05	0.000162232
3242	ENSG00000227509.1	LINC01839		-2.52868657	4.79705E-05	0.000163879
3243	ENSG00000129152.4	MYOD1	4654	-2.203793306	4.8037E-05	0.000164092
3244	ENSG00000229628.1			-1.462938513	4.81143E-05	0.000164314
3245	ENSG00000099960.13	SLC7A4	6545	-1.36408344	4.83844E-05	0.000165179
3246	ENSG00000236469.1			-1.579813723	4.84176E-05	0.000165264
3247	ENSG00000197416.4	FABP12	646486	-1.424685213	4.86037E-05	0.000165842
3248	ENSG00000277112.3	ANKRD20A21P		-1.623067406	4.87071E-05	0.000166138
3249	ENSG00000284395.2	PERCC1	105371045	-1.381134034	4.91508E-05	0.000167536
3250	ENSG00000280415.1			-2.359672671	4.93551E-05	0.000168189
3251	ENSG00000149488.13	TMC2	117532	-1.116621826	4.97098E-05	0.000169354
3252	ENSG00000285420.1	PDGFRL2P		-1.029004199	4.98303E-05	0.00016975
3253	ENSG00000163982.6	OTOP1	133060	-1.86658374	4.99797E-05	0.000170186
3254	ENSG00000224968.2	LINC01645		-1.522612991	5.10326E-05	0.000173533
3255	ENSG00000259070.7	LINC00639	283547	-1.173452087	5.12948E-05	0.000174396
3256	ENSG00000285885.1			-2.019232725	5.18522E-05	0.000176078
3257	ENSG00000164175.15	SLC45A2	51151	-1.100355962	5.19116E-05	0.000176265
3258	ENSG00000267391.5	MIR122HG		-1.627268174	5.21137E-05	0.00017686
3259	ENSG00000254041.1	LINC03084		-1.915704977	5.31454E-05	0.000180076
3260	ENSG00000258847.1			-1.663961535	5.31466E-05	0.000180076
3261	ENSG00000254872.3	LINC02688		-1.647892621	5.31477E-05	0.000180076
3262	ENSG00000250821.3	EXOC1L	644145	-2.394624225	5.32331E-05	0.00018035
3263	ENSG00000214100.8	PLAC9P1		-1.61413314	5.39383E-05	0.000182583
3264	ENSG00000230809.1	RPS6P15		-2.175344736	5.42075E-05	0.000183385
3265	ENSG00000280409.1	LINC01101	84931	-2.379914492	5.4372E-05	0.000183895
3266	ENSG00000179002.5	TAS1R2	80834	-1.392499331	5.52347E-05	0.000186591
3267	ENSG00000233292.1			-1.629896277	5.84004E-05	0.000196547
3268	ENSG00000283646.2	LINC02009	105377068	-1.188751736	6.04613E-05	0.000202863
3269	ENSG00000286747.1			-1.003198428	6.05995E-05	0.000203309
3270	ENSG00000230368.2	FAM41C	284593	-2.353863718	6.09223E-05	0.000204271
3271	ENSG00000143631.11	FLG	2312	-1.539764592	6.13686E-05	0.000205698
3272	ENSG00000228983.9	SLC47A1P1		-1.668290665	6.14444E-05	0.000205883
3273	ENSG00000286849.1			-1.350347873	6.22069E-05	0.000208174
3274	ENSG00000181626.12	ANKRD62	342850	-1.234633903	6.23939E-05	0.000208694
3275	ENSG00000109061.10	MYH1	4619	-2.30868197	6.25115E-05	0.000209069
3276	ENSG00000273160.1			-1.035542341	6.27924E-05	0.00020992
3277	ENSG00000100095.19	SEZ6L	23544	-1.506678384	6.35006E-05	0.000212073
3278	ENSG00000170959.14	DCDC1	341019	-1.183262646	6.37052E-05	0.000212685
3279	ENSG00000279551.1			-2.565845705	6.38694E-05	0.000213161
3280	ENSG00000174514.13	MFSD4A	148808	-1.259169138	6.57724E-05	0.000219052
3281	ENSG00000205861.13	PCOTH	542767	-1.136586471	6.83282E-05	0.000226972
3282	ENSG00000224769.1	MUC20P1		-1.337416605	6.92642E-05	0.00022987
3283	ENSG00000162706.13	CADM3	57863	-1.032116813	6.97484E-05	0.000231361
3284	ENSG00000198092.6	TMPRSS11F	389208	-1.168728196	7.03487E-05	0.000233235
3285	ENSG00000224185.1	SNX18P9		-2.181692474	7.10229E-05	0.000235293
3286	ENSG00000213620.3	RPL21P70		-1.200853685	7.14579E-05	0.000236665
3287	ENSG00000133067.17	LGR6	59352	-1.168843763	7.15489E-05	0.000236937
3288	ENSG00000157502.13	PWWP3B	139221	-1.435347727	7.19566E-05	0.000238207
3289	ENSG00000287354.1		124904503	-1.837258189	7.21518E-05	0.000238794
3290	ENSG00000275965.1			-1.893567079	7.30713E-05	0.000241574
3291	ENSG00000287281.1		107985177	-1.209688059	7.32277E-05	0.000242051
3292	ENSG00000062524.16	LTK	4058	-1.023178165	7.33167E-05	0.000242325
3293	ENSG00000285188.1	PDE4C	5143	-1.570933905	7.42055E-05	0.000245079
3294	ENSG00000264443.1			-2.051384027	7.5352E-05	0.000248658
3295	ENSG00000233093.6	LINC00892	100128420	-1.00497853	7.62744E-05	0.000251451
3296	ENSG00000232139.6	LINC00867	100506126	-1.16105417	7.64705E-05	0.000252056
3297	ENSG00000230215.1	LINC01840	100874079	-1.787514824	7.65422E-05	0.000252258
3298	ENSG00000253295.2		101928093	-1.076804529	7.69555E-05	0.000253507
3299	ENSG00000163817.16	SLC6A20	54716	-1.254834296	7.7263E-05	0.000254435
3300	ENSG00000250626.3		101930028	-1.591932283	7.79743E-05	0.000256607
3301	ENSG00000158077.5	NLRP14	338323	-1.139563921	7.89241E-05	0.000259561
3302	ENSG00000253644.2	LINC02845	105375680	-1.734336679	7.91501E-05	0.000260217
3303	ENSG00000232903.2	LINC01166	101927590	-1.849078236	7.93414E-05	0.000260717
3304	ENSG00000272817.1			-1.020592821	7.9639E-05	0.000261565
3305	ENSG00000197320.5	CPAMD8P1		-1.866787496	8.00244E-05	0.000262743
3306	ENSG00000278737.1			-1.200142507	8.18255E-05	0.000268235
3307	ENSG00000129514.8	FOXA1	3169	-1.275305506	8.19277E-05	0.000268526
3308	ENSG00000215174.2	NLRP2B	286430	-1.271040053	8.20872E-05	0.000268937
3309	ENSG00000070019.5	GUCY2C	2984	-1.317458264	8.45882E-05	0.000276651
3310	ENSG00000229676.3	ZNF492	57615	-1.275856761	8.47244E-05	0.000277051
3311	ENSG00000288063.1		124906237	-1.038986529	8.56978E-05	0.000280095
3312	ENSG00000251616.1		105379178	-2.283646739	8.59129E-05	0.000280775
3313	ENSG00000134160.14	TRPM1	4308	-1.544058347	8.60339E-05	0.000281124
3314	ENSG00000205929.11	EPCIP	56245	-1.032671507	8.63857E-05	0.000282181
3315	ENSG00000234107.1	TPT1P1		-1.087548715	8.64564E-05	0.000282365
3316	ENSG00000287403.1		124904076	-1.347481675	8.67157E-05	0.000283075
3317	ENSG00000268101.2	CYP2G2P		-2.411627426	8.76072E-05	0.000285747
3318	ENSG00000144218.19	AFF3	3899	-1.019141439	8.88534E-05	0.000289549
3319	ENSG00000248514.1			-1.026114769	8.90256E-05	0.000290063
3320	ENSG00000263167.1			-1.155406252	8.94504E-05	0.000291259
3321	ENSG00000256812.2	CAPNS2	84290	-1.024765641	8.94515E-05	0.000291259
3322	ENSG00000285719.2			-1.302936806	9.02066E-05	0.000293573
3323	ENSG00000262686.1	GLIS2-AS1		-1.263470823	9.03066E-05	0.00029385
3324	ENSG00000260469.3	INSYN1-AS1		-1.826301217	9.04107E-05	0.000294141
3325	ENSG00000150625.16	GPM6A	2823	-1.330865884	9.1434E-05	0.00029725
3326	ENSG00000187905.10	LRRC74B	400891	-1.287812596	9.1652E-05	0.000297813
3327	ENSG00000232254.1	CSF2RBP1		-1.73085283	9.18547E-05	0.000298349
3328	ENSG00000129170.10	CSRP3	8048	-2.387246733	9.19593E-05	0.000298664
3329	ENSG00000155897.10	ADCY8	114	-2.346121727	9.28384E-05	0.000301297
3330	ENSG00000171815.6	PCDHB1	29930	-1.592395504	9.37096E-05	0.000303901
3331	ENSG00000185176.13	AQP12B	375318	-2.0838024	9.80476E-05	0.000316829
3332	ENSG00000149970.16	CNKSR2	22866	-1.127463021	9.85196E-05	0.00031825
3333	ENSG00000262097.2	LINC02185		-1.089974467	0.000100243	0.000323581
3334	ENSG00000251332.1	PSME2P4		-1.157609254	0.000102308	0.000329655
3335	ENSG00000233256.1			-2.210368881	0.000102527	0.000330279
3336	ENSG00000253404.1	CTNNA1-AS1	105379194	-1.342263228	0.000103564	0.000333325
3337	ENSG00000260969.2	WWOX-AS1		-1.502179094	0.000104027	0.000334705
3338	ENSG00000169031.20	COL4A3	1285	-1.105986031	0.00010505	0.000337777
3339	ENSG00000288045.1			-1.100389202	0.000105255	0.000338299
3340	ENSG00000248373.6			-1.448279114	0.000105548	0.000339184
3341	ENSG00000232699.3	BDH2P1		-1.114080295	0.000105595	0.000339311
3342	ENSG00000100565.15	LRRC74A	145497	-1.209250996	0.000106794	0.000342804
3343	ENSG00000179673.5	RPRML	388394	-1.979619506	0.000107709	0.000345485
3344	ENSG00000267421.6	ZFP28-DT	101928982	-1.132056113	0.000107838	0.000345843
3345	ENSG00000286050.1			-1.920075358	0.000108295	0.000347142
3346	ENSG00000220586.2	TUBBP9		-1.16919539	0.000108422	0.000347493
3347	ENSG00000165215.6	CLDN3	1365	-1.869794717	0.000110141	0.000352518
3348	ENSG00000213386.3			-1.090421791	0.000110555	0.000353699
3349	ENSG00000285694.1			-1.384266866	0.000112159	0.000358369
3350	ENSG00000229654.1	LINC02526		-1.580576456	0.000112935	0.000360645
3351	ENSG00000173714.8	WFIKKN2	124857	-1.780980284	0.000113502	0.000362374
3352	ENSG00000242899.1	RPL7P16		-1.119113593	0.000113671	0.000362878
3353	ENSG00000288075.1		105369250	-1.035940553	0.000114118	0.00036413
3354	ENSG00000287082.1		101928398	-1.776759897	0.000115347	0.000367636
3355	ENSG00000251189.1			-1.743995041	0.000116343	0.000370572
3356	ENSG00000141639.12	MAPK4	5596	-1.396572779	0.000116839	0.000372033
3357	ENSG00000288437.1			-1.011647899	0.000117423	0.00037377
3358	ENSG00000122859.5	NEUROG3	50674	-1.371251388	0.000119117	0.000378587
3359	ENSG00000268119.6		105372321	-1.094765801	0.000119868	0.000380882
3360	ENSG00000267097.1	SLC14A2-AS1		-1.387123209	0.00012071	0.000383342
3361	ENSG00000223991.1	TANAR	128266846	-2.081014209	0.000121994	0.000387202
3362	ENSG00000237125.11	HAND2-AS1	79804	-1.140353248	0.000122666	0.000389138
3363	ENSG00000189292.16	ALKAL2	285016	-1.231914028	0.000122896	0.000389754
3364	ENSG00000273597.1			-1.095888496	0.000124191	0.00039345
3365	ENSG00000159625.15	DRC7	84229	-1.372162606	0.000124406	0.000394007
3366	ENSG00000275392.1			-1.925716921	0.000124448	0.000394108
3367	ENSG00000102245.8	CD40LG	959	-1.045135026	0.000127279	0.000402526
3368	ENSG00000162669.16	HFM1	164045	-1.246365116	0.000129751	0.000409725
3369	ENSG00000185274.12	GALNT17	64409	-1.119458004	0.000129848	0.000409964
3370	ENSG00000214142.2	RPL7P60		-1.283967765	0.000130633	0.000412312
3371	ENSG00000147647.13	DPYS	1807	-1.193837446	0.000131781	0.000415539
3372	ENSG00000169126.16	ODAD2	55130	-1.328862352	0.000132875	0.000418786
3373	ENSG00000084674.15	APOB	338	-1.33657286	0.000133848	0.000421579
3374	ENSG00000232451.2			-2.596193037	0.000134931	0.000424594
3375	ENSG00000138823.13	MTTP	4547	-1.050614784	0.000141882	0.000444842
3376	ENSG00000287886.1			-1.292483672	0.000142575	0.000446592
3377	ENSG00000214814.7	FER1L6	654463	-1.275897615	0.000145602	0.00045521
3378	ENSG00000269933.1			-1.353671168	0.000145989	0.00045624
3379	ENSG00000184221.13	OLIG1	116448	-1.582266586	0.000146859	0.000458815
3380	ENSG00000285336.1		101928882	-1.42454823	0.000146954	0.000459074
3381	ENSG00000262966.2		101928266	-1.19906378	0.000148716	0.000464031
3382	ENSG00000159263.16	SIM2	6493	-1.224722941	0.000152417	0.000474607
3383	ENSG00000275645.1	NEIL1-AS1		-1.021139484	0.000152667	0.000475348
3384	ENSG00000286250.1			-1.807490962	0.000153673	0.000478069
3385	ENSG00000246528.3	SLCO5A1-AS1	121233925	-1.15048771	0.000156606	0.000486583
3386	ENSG00000273267.1			-1.032442801	0.000156847	0.000487217
3387	ENSG00000223502.1		102724719	-1.474884429	0.00016349	0.000506264
3388	ENSG00000188004.11	SNHG28	284677	-1.506607586	0.000164302	0.000508621
3389	ENSG00000261447.1			-1.054162281	0.000165586	0.000512194
3390	ENSG00000198003.12	ODAD3	115948	-1.001002763	0.000165982	0.000513299
3391	ENSG00000214797.3			-1.865003593	0.000166244	0.000513989
3392	ENSG00000237763.9	AMY1A	276	-2.972933454	0.000167287	0.000516853
3393	ENSG00000287820.1			-1.626237084	0.000167443	0.000517273
3394	ENSG00000040731.10	CDH10	1008	-1.69665176	0.000167449	0.000517273
3395	ENSG00000141744.4	PNMT	5409	-1.325144437	0.000167582	0.000517602
3396	ENSG00000224466.1	CDCA4P2		-1.765136177	0.000168898	0.000521322
3397	ENSG00000286206.1			-2.865692832	0.000171555	0.000528845
3398	ENSG00000286945.1			-1.616766141	0.000171656	0.000529114
3399	ENSG00000250538.6	NPY2R-AS1		-3.086824477	0.000174751	0.00053786
3400	ENSG00000234805.1	FAM151AP1		-1.487684505	0.000174892	0.000538168
3401	ENSG00000260592.1		124903659	-1.538924065	0.000175227	0.00053903
3402	ENSG00000286598.1		105370728	-2.817287702	0.000175638	0.000540213
3403	ENSG00000214433.4	GOLGA2P8		-1.072394124	0.000177156	0.000544628
3404	ENSG00000260776.6	GOLGA6FP		-1.311748268	0.000179949	0.000552486
3405	ENSG00000268628.2			-1.246704353	0.000181221	0.000556045
3406	ENSG00000284309.1			-1.948980777	0.000181436	0.000556663
3407	ENSG00000144290.17	SLC4A10	57282	-1.097083271	0.000181556	0.000556988
3408	ENSG00000127954.13	STEAP4	79689	-1.042952203	0.000182691	0.000560298
3409	ENSG00000140465.14	CYP1A1	1543	-1.977774938	0.00018438	0.000565082
3410	ENSG00000263312.1	LINC01975		-2.781173184	0.000185046	0.000567037
3411	ENSG00000264727.1			-1.545964699	0.000186351	0.00057077
3412	ENSG00000264932.3			-1.045396278	0.000189864	0.000580659
3413	ENSG00000237742.7	HEY2-AS1	105377986	-1.228024914	0.000192556	0.0005882
3414	ENSG00000204092.2			-2.499405578	0.00019413	0.000592556
3415	ENSG00000254333.1	NDST1-AS1		-1.119679574	0.000194335	0.00059307
3416	ENSG00000234638.1			-2.358163331	0.000195446	0.000596187
3417	ENSG00000177984.7	LCN15	389812	-1.612208685	0.000196337	0.000598768
3418	ENSG00000167580.8	AQP2	359	-2.015297577	0.000200188	0.000609762
3419	ENSG00000199990.1	VTRNA1-1	56664	-1.453576098	0.000201942	0.000614774
3420	ENSG00000173930.9	SLCO4C1	353189	-1.433643869	0.000201961	0.000614782
3421	ENSG00000225129.1			-1.443911744	0.000203126	0.000617997
3422	ENSG00000225521.1	SNED1-AS1		-1.2229636	0.000203221	0.000618192
3423	ENSG00000260253.1			-1.47219419	0.000206387	0.000627244
3424	ENSG00000204625.10	HCG9	10255	-1.058151856	0.000206415	0.000627283
3425	ENSG00000239831.1	RNF7P1		-1.248479614	0.000209474	0.0006358
3426	ENSG00000140279.13	DUOX2	50506	-1.073531507	0.000211796	0.000642158
3427	ENSG00000271239.1			-2.794687169	0.000213248	0.000646314
3428	ENSG00000233730.1	LINC01765		-1.795046529	0.000213299	0.000646406
3429	ENSG00000250126.1			-1.532053998	0.000215427	0.00065217
3430	ENSG00000274698.1			-1.665113827	0.000216351	0.000654716
3431	ENSG00000255084.1	NARS2-AS1	101928896	-1.4939495	0.000221196	0.000668563
3432	ENSG00000171234.14	UGT2B7	7364	-1.558069735	0.000221531	0.000669421
3433	ENSG00000203808.12	POPDC1-AS1	154442	-1.015293667	0.000223485	0.000675017
3434	ENSG00000231258.2	ZSWIM5P2		-1.344406178	0.000224167	0.000676924
3435	ENSG00000233539.5		730338	-1.27114063	0.000224525	0.00067785
3436	ENSG00000182531.7	OR7E115P		-1.309976274	0.000229186	0.000690815
3437	ENSG00000287287.1			-1.554069275	0.000232529	0.000700147
3438	ENSG00000255027.2	DDX25-AS1		-2.395192227	0.000232985	0.000701415
3439	ENSG00000169064.13	ZBBX	79740	-1.967042504	0.00023686	0.000712268
3440	ENSG00000137673.9	MMP7	4316	-1.187101012	0.000240023	0.000721288
3441	ENSG00000133020.4	MYH8	4626	-1.994640582	0.000247216	0.000740938
3442	ENSG00000279581.1			-1.46274506	0.000250496	0.000750089
3443	ENSG00000162761.14	LMX1A	4009	-1.626125033	0.000253144	0.000757561
3444	ENSG00000288002.1			-1.849836452	0.000256851	0.00076761
3445	ENSG00000264968.1			-1.169605874	0.000262313	0.00078246
3446	ENSG00000230710.2	LINC00332		-1.681362587	0.000262738	0.00078355
3447	ENSG00000188100.9	FAM25A	643161	-1.362570326	0.000268651	0.000799621
3448	ENSG00000231651.1	DLG3-AS1	100873930	-1.144216987	0.000269646	0.000802101
3449	ENSG00000114349.10	GNAT1	2779	-1.035796152	0.000274564	0.000815139
3450	ENSG00000243069.8	ARHGEF26-AS1	100507524	-1.24073821	0.00027594	0.000818794
3451	ENSG00000218357.3	LINC01644	101927722	-1.500945128	0.000287736	0.000851377
3452	ENSG00000187922.14	LCN10	414332	-1.295419757	0.000287941	0.0008519
3453	ENSG00000175287.19	PHYHD1	254295	-1.091699894	0.000293205	0.000866461
3454	ENSG00000100181.22	TPTEP1		-1.213198276	0.000296503	0.000875487
3455	ENSG00000154198.14	CYP4Z2P		-1.694413817	0.000301991	0.00089063
3456	ENSG00000196917.6	HCAR1	27198	-1.447447011	0.000307807	0.000906434
3457	ENSG00000267262.1	RFX2-AS1	139281648	-1.105131881	0.000314032	0.000923532
3458	ENSG00000221971.3	TTC4P1		-1.425722246	0.000314176	0.000923888
3459	ENSG00000281692.2	PACRG-AS1		-1.200309054	0.000318783	0.000936115
3460	ENSG00000287090.1			-1.467853426	0.000320512	0.000940705
3461	ENSG00000130876.11	SLC7A10	56301	-1.519285248	0.000329421	0.000964853
3462	ENSG00000112175.8	BMP5	653	-1.471131874	0.000338067	0.000987803
3463	ENSG00000199400.1	RNY4P19		-1.121454995	0.000341798	0.000998154
3464	ENSG00000177108.5	ZDHHC22	283576	-1.53383365	0.000345105	0.001006479
3465	ENSG00000184226.15	PCDH9	5101	-1.027397883	0.000349046	0.001016179
3466	ENSG00000124143.10	ARHGAP40	343578	-1.027713743	0.000353664	0.001028488
3467	ENSG00000288098.1			-1.891461071	0.000355254	0.00103257
3468	ENSG00000229155.1	LINC02038		-1.625921779	0.000355276	0.00103257
3469	ENSG00000280302.1			-1.428977024	0.000357131	0.001037505
3470	ENSG00000236711.2	SMAD9-IT1		-1.128563069	0.000358372	0.001040653
3471	ENSG00000170276.6	HSPB2	3316	-1.72794297	0.000365903	0.001060813
3472	ENSG00000283458.1		101927879	-2.090991142	0.000367928	0.001066138
3473	ENSG00000258603.4			-1.06473718	0.000370539	0.001072998
3474	ENSG00000287504.1			-1.691772633	0.000372017	0.001076649
3475	ENSG00000186190.7	BPIFB3	359710	-2.344942066	0.000374474	0.001082812
3476	ENSG00000168748.14	CA7	766	-1.4494094	0.000378827	0.00109452
3477	ENSG00000112494.10	UNC93A	54346	-1.210648272	0.000379304	0.00109574
3478	ENSG00000223802.8	CERS1	10715	-1.012044708	0.000379775	0.00109702
3479	ENSG00000138109.11	CYP2C9	1559	-1.249411423	0.000382376	0.00110365
3480	ENSG00000280350.1	LINC01726	101929608	-1.988260367	0.000382412	0.001103674
3481	ENSG00000278952.1			-1.069455377	0.000383053	0.001105361
3482	ENSG00000205266.10	KRT17P5		-1.049425319	0.000383771	0.001107273
3483	ENSG00000109047.8	RCVRN	5957	-1.005039046	0.000385361	0.001111295
3484	ENSG00000285691.2			-1.696060172	0.000392149	0.001129063
3485	ENSG00000235939.1			-1.237411734	0.000395601	0.001138174
3486	ENSG00000242948.1	EPS15P1		-1.233090676	0.000406459	0.001167465
3487	ENSG00000241829.1	RPL21P54		-1.725285424	0.000408356	0.00117249
3488	ENSG00000286504.1			-1.128531779	0.000409078	0.001174478
3489	ENSG00000233996.1	KDM3AP1		-1.37198245	0.000409442	0.001175268
3490	ENSG00000176728.10	TTTY14	83869	-1.431296062	0.000411813	0.001181732
3491	ENSG00000279913.1			-1.056936772	0.000417906	0.001197567
3492	ENSG00000232606.2	LINC01412		-1.493086443	0.000426681	0.001220857
3493	ENSG00000232806.1			-1.77794084	0.00043038	0.001230204
3494	ENSG00000279619.1			-1.248631336	0.000434969	0.001242138
3495	ENSG00000286818.1			-1.378467586	0.0004469	0.001273017
3496	ENSG00000243642.3	RN7SL526P		-1.22188251	0.000449868	0.001280553
3497	ENSG00000280429.1			-1.56342192	0.000450949	0.001283537
3498	ENSG00000124440.16	HIF3A	64344	-1.227716203	0.000452868	0.001288353
3499	ENSG00000230126.1	FGF12-AS2	100873987	-1.220963736	0.000476481	0.001348754
3500	ENSG00000134612.12	FOLH1B		-1.359962891	0.00048341	0.001366709
3501	ENSG00000256757.1			-1.378865624	0.000489288	0.001382539
3502	ENSG00000279414.1	CDRT15P9		-1.576252582	0.000490318	0.001385152
3503	ENSG00000077264.15	PAK3	5063	-1.092824485	0.000495439	0.001398226
3504	ENSG00000257057.3	C11orf97	643037	-3.064170219	0.000500855	0.001411801
3505	ENSG00000236373.2	LINC02653	101926942	-1.521419497	0.000503631	0.001419121
3506	ENSG00000204421.3	LY6G6C	80740	-1.162484168	0.000504689	0.001422001
3507	ENSG00000268055.1			-1.99833413	0.000504984	0.001422731
3508	ENSG00000249646.2	OR7E94P		-1.203601899	0.000511058	0.001438824
3509	ENSG00000224445.3	LINC01708	101928270	-1.528783364	0.000511726	0.001440498
3510	ENSG00000099769.6	IGFALS	3483	-1.030289694	0.000520426	0.001463224
3511	ENSG00000273291.5			-1.191730783	0.000522939	0.001469766
3512	ENSG00000229224.2			-1.134751606	0.000529746	0.001487002
3513	ENSG00000230173.1	LINC01790	101927431	-2.965309459	0.000530669	0.00148917
3514	ENSG00000167014.11	TERB2	145645	-1.602592529	0.000547166	0.001531994
3515	ENSG00000015520.15	NPC1L1	29881	-1.014486212	0.000554049	0.001549952
3516	ENSG00000137960.6	GIPC2	54810	-1.029726387	0.000556098	0.001555087
3517	ENSG00000176204.13	LRRTM4	80059	-1.448182522	0.000563879	0.001574343
3518	ENSG00000228792.3			-2.199773604	0.00056675	0.001581689
3519	ENSG00000042813.8	ZPBP	11055	-1.686428582	0.000569254	0.001588119
3520	ENSG00000234604.2			-1.049816998	0.000574194	0.001601112
3521	ENSG00000179930.6	ZNF648	127665	-1.695098126	0.000580409	0.001616284
3522	ENSG00000277651.1			-1.311732479	0.00058078	0.001617203
3523	ENSG00000275418.1	LINC03110	102724745	-1.086707349	0.000584334	0.00162607
3524	ENSG00000048540.15	LMO3	55885	-1.221528894	0.000585784	0.001629535
3525	ENSG00000257878.1			-1.447592864	0.000588681	0.001636789
3526	ENSG00000252920.1		124900435	-2.0829595	0.000594813	0.001651985
3527	ENSG00000162949.16	CAPN13	92291	-1.443367587	0.000597137	0.001658092
3528	ENSG00000285081.1			-1.443516893	0.000604794	0.001677239
3529	ENSG00000235399.1			-1.182234434	0.000605991	0.001680205
3530	ENSG00000126950.8	TMEM35A	59353	-1.103720678	0.000606118	0.001680441
3531	ENSG00000242353.1	RPL12P30		-1.431061892	0.00060984	0.001690403
3532	ENSG00000229886.1			-1.152878637	0.000611587	0.001694772
3533	ENSG00000120057.5	SFRP5	6425	-1.444411233	0.000612756	0.00169742
3534	ENSG00000287370.1			-1.432682964	0.000624757	0.001728008
3535	ENSG00000165188.13	RNF183	138065	-1.259022678	0.000626596	0.001732128
3536	ENSG00000262769.1			-1.429795442	0.000641273	0.001769984
3537	ENSG00000284748.1			-1.796825881	0.000642466	0.001772415
3538	ENSG00000185634.12	SHC4	399694	-1.018732763	0.000645097	0.001778683
3539	ENSG00000183067.5	IGSF5	150084	-1.402526075	0.00064814	0.001785955
3540	ENSG00000173597.9	SULT1B1	27284	-1.046728469	0.000660344	0.001817309
3541	ENSG00000251448.2			-1.177029065	0.000663541	0.001824968
3542	ENSG00000288531.1			-1.272623236	0.000676507	0.00185638
3543	ENSG00000132872.12	SYT4	6860	-1.50225782	0.000684956	0.001877615
3544	ENSG00000167104.11	BPIFB6	128859	-3.08639284	0.00069802	0.001910785
3545	ENSG00000168952.15	STXBP6	29091	-1.053600671	0.000707242	0.001933491
3546	ENSG00000286203.1			-1.44482015	0.000707725	0.00193468
3547	ENSG00000251385.2	MTCYBP16		-1.398339271	0.000709547	0.001939125
3548	ENSG00000244674.1	RPS3AP15		-1.517585553	0.000725663	0.001978941
3549	ENSG00000229246.2	LINC00377		-1.566054506	0.000729365	0.001988077
3550	ENSG00000255311.5	DLG2-AS2		-1.765096123	0.00074059	0.002014934
3551	ENSG00000255036.6	SUGT1P4-STRA6LP-CCDC180	57653	-1.549564845	0.00074865	0.002034351
3552	ENSG00000175267.15	VWA3A	146177	-1.13982002	0.000752632	0.00204475
3553	ENSG00000246394.7	ZNF84-DT	101928597	-1.502826997	0.000758326	0.002058668
3554	ENSG00000248799.1			-2.269498509	0.000777417	0.00210574
3555	ENSG00000119919.11	NKX2-3	159296	-1.529580249	0.000778242	0.002107685
3556	ENSG00000186652.10	PRG2	5553	-1.003187857	0.000782645	0.002118018
3557	ENSG00000223403.6	MEG9	378881	-1.095559466	0.000783715	0.002120624
3558	ENSG00000140798.16	ABCC12	94160	-1.733553082	0.000785566	0.002124619
3559	ENSG00000228835.1			-1.462151336	0.0007988	0.002156587
3560	ENSG00000177519.4	RPRM	56475	-1.141168562	0.000802396	0.002164967
3561	ENSG00000286848.1		101927297	-1.364582371	0.000820227	0.002207971
3562	ENSG00000102891.4	MT4	84560	-2.147139673	0.00082628	0.002222603
3563	ENSG00000225867.1			-1.215745681	0.000829624	0.002230689
3564	ENSG00000206172.8	HBA1	3039	-1.187366753	0.000832877	0.002238223
3565	ENSG00000251370.1	SEMA5A-AS1	101929338	-1.350926063	0.00085336	0.002289078
3566	ENSG00000286721.1			-1.097029822	0.00085841	0.002301043
3567	ENSG00000226074.5	PRSS44P		-1.609182935	0.000858459	0.002301043
3568	ENSG00000277572.1			-1.226710725	0.000859305	0.002302845
3569	ENSG00000231535.7	LINC00278		-1.359896489	0.000864609	0.00231565
3570	ENSG00000274749.1	KRTAP7-1	337878	-2.937942168	0.000867583	0.0023233
3571	ENSG00000227835.8	CARM1P1		-1.459054329	0.00087349	0.002337226
3572	ENSG00000054938.16	CHRDL2	25884	-1.02418945	0.000901551	0.002406574
3573	ENSG00000204179.10	PTPN20	26095	-1.192385914	0.000901593	0.002406574
3574	ENSG00000156687.11	UNC5D	137970	-1.334406111	0.000915166	0.002440341
3575	ENSG00000239552.2	HOXB-AS2		-1.322256878	0.000927759	0.002471095
3576	ENSG00000168004.10	PLAAT5	117245	-1.302295147	0.000928098	0.002471665
3577	ENSG00000166006.14	KCNC2	3747	-2.495366544	0.000942299	0.00250679
3578	ENSG00000259920.1			-1.070228839	0.000958646	0.002546348
3579	ENSG00000142515.15	KLK3	354	-2.022720118	0.000962139	0.002553573
3580	ENSG00000255474.1	GAU1	101929549	-1.045320394	0.000967607	0.00256637
3581	ENSG00000164871.17	SPAG11B	10407	-2.401486192	0.000976125	0.002585847
3582	ENSG00000186288.5	PABPC1L2A	340529	-3.434842794	0.000985315	0.002608451
3583	ENSG00000283625.1	TEDDM3P		-1.516907165	0.000992507	0.002625386
3584	ENSG00000260386.6	LDC1P		-1.21561679	0.000992713	0.002625755
3585	ENSG00000179593.16	ALOX15B	247	-1.030489566	0.001012428	0.002672896
3586	ENSG00000228918.4	LINC01344		-1.123481275	0.001014595	0.002677562
3587	ENSG00000205186.4	FABP9	646480	-1.539124927	0.00103214	0.002720164
3588	ENSG00000203697.12	CAPN8	388743	-1.106715738	0.001038943	0.002735262
3589	ENSG00000204538.4	PSORS1C2	170680	-1.24229272	0.00106946	0.002808519
3590	ENSG00000272568.6	CPVL-AS2	100506497	-1.020445343	0.001093163	0.002864694
3591	ENSG00000286330.1			-1.274016851	0.001107318	0.002898913
3592	ENSG00000254781.1	GVINP2		-1.608468205	0.001110407	0.002906233
3593	ENSG00000164270.18	HTR4	3360	-1.004542922	0.001119364	0.002927164
3594	ENSG00000223839.8	FAM95B1		-1.201768103	0.001120632	0.002929901
3595	ENSG00000285850.1			-1.072231977	0.001139544	0.002974833
3596	ENSG00000180772.7	AGTR2	186	-1.832317604	0.00114385	0.002984307
3597	ENSG00000139988.10	RDH12	145226	-1.100573328	0.001145213	0.002987666
3598	ENSG00000167800.9	TBX10	347853	-1.242606645	0.001152849	0.0030064
3599	ENSG00000271742.1			-1.161141514	0.00116164	0.003026738
3600	ENSG00000276241.1		102723851	-1.465055017	0.001164732	0.0030332
3601	ENSG00000180287.17	PLD5	200150	-1.334032889	0.001167114	0.003038405
3602	ENSG00000204460.3	LINC01854	151121	-2.157610657	0.001189443	0.003092042
3603	ENSG00000283051.1	LINC01668	101928311	-1.283506641	0.001203934	0.003124822
3604	ENSG00000233005.2			-1.648646541	0.001235789	0.003199335
3605	ENSG00000287319.1			-1.027443098	0.001250751	0.003234057
3606	ENSG00000154478.4	GPR26	2849	-1.221882296	0.001263952	0.003264358
3607	ENSG00000233377.1	MTND4P20		-1.391811371	0.001270658	0.003279969
3608	ENSG00000279302.3			-1.141028905	0.001284834	0.003312895
3609	ENSG00000137731.14	FXYD2	486	-1.04070677	0.001301216	0.003352736
3610	ENSG00000280809.4	LINC00836	101929052	-1.594573894	0.001301633	0.003353157
3611	ENSG00000182938.7	OTOP3	347741	-1.331982226	0.001310721	0.003374376
3612	ENSG00000277135.1			-1.103672916	0.001326385	0.003410716
3613	ENSG00000267332.1	BOLA3P2		-1.444905216	0.001338989	0.003438221
3614	ENSG00000175229.7	GAL3ST3	89792	-1.510890177	0.001346234	0.003455259
3615	ENSG00000267246.1			-1.190231586	0.001348209	0.003460103
3616	ENSG00000008394.13	MGST1	4257	-1.141141001	0.001356834	0.003479986
3617	ENSG00000244358.1			-1.694287484	0.00135779	0.003481987
3618	ENSG00000174898.16	CATSPERD	257062	-1.132370879	0.001366756	0.003502941
3619	ENSG00000165238.16	WNK2	65268	-1.066637218	0.001369729	0.003510334
3620	ENSG00000136929.13	HEMGN	55363	-1.123148882	0.001379338	0.003532451
3621	ENSG00000287435.1		105374802	-2.215250373	0.001400489	0.003578296
3622	ENSG00000274863.1			-1.1491823	0.001401191	0.003579731
3623	ENSG00000267345.1			-1.020171016	0.001401231	0.003579731
3624	ENSG00000006071.15	ABCC8	6833	-1.333429221	0.001403306	0.003584107
3625	ENSG00000261701.7	HPR	3250	-1.515966384	0.00141292	0.003605642
3626	ENSG00000260239.2	LINC02533		-1.076012714	0.001428078	0.003640107
3627	ENSG00000233639.7	PANTR1	100506421	-1.261676563	0.001439807	0.003667647
3628	ENSG00000184672.12	RALYL	138046	-1.578107356	0.001440935	0.003669341
3629	ENSG00000167910.4	CYP7A1	1581	-1.021416545	0.001497867	0.003797004
3630	ENSG00000239268.3	LINC03051	127138867	-2.146492409	0.001511349	0.003827265
3631	ENSG00000243543.9	WFDC6	140870	-2.658060464	0.001528055	0.003867594
3632	ENSG00000283413.1			-2.150664685	0.00154023	0.003895673
3633	ENSG00000171060.11	C8orf74	203076	-1.265651035	0.001576191	0.003977494
3634	ENSG00000264785.1			-1.431046808	0.00158571	0.003998971
3635	ENSG00000267594.5	CYP4F24P		-1.361365834	0.001594724	0.004020168
3636	ENSG00000227888.4	FAM66A		-1.0262753	0.001597803	0.004027161
3637	ENSG00000227922.1	SPTLC1P5		-1.404908513	0.001615762	0.004068233
3638	ENSG00000277479.1			-1.069399133	0.001617366	0.004071552
3639	ENSG00000254620.1			-1.229000773	0.001650192	0.004146552
3640	ENSG00000279668.3			-1.274394577	0.001670537	0.004193158
3641	ENSG00000259560.2			-2.02180436	0.001678273	0.00420991
3642	ENSG00000231720.1			-3.084340119	0.001712453	0.004288055
3643	ENSG00000265542.5			-1.023778657	0.001718961	0.004302719
3644	ENSG00000287919.1			-1.187495523	0.001721172	0.004307437
3645	ENSG00000169402.15	RSPH10B2	222967	-1.115964442	0.001725744	0.00431779
3646	ENSG00000263427.1			-1.530925072	0.001741429	0.004354012
3647	ENSG00000286805.1			-1.16319264	0.001748325	0.004368773
3648	ENSG00000233214.1			-1.063008178	0.001754081	0.004382329
3649	ENSG00000182077.12	PTCHD3	374308	-1.362599927	0.001765315	0.004407341
3650	ENSG00000254038.1			-1.062793789	0.001781197	0.004443915
3651	ENSG00000260693.1			-1.031898465	0.001791436	0.004467772
3652	ENSG00000241899.1	TPT1P3		-1.019015176	0.001806407	0.004501994
3653	ENSG00000162456.10	KNCN	148930	-2.029072049	0.001807732	0.004504731
3654	ENSG00000225539.7	LINC01821		-1.886702423	0.001839056	0.004575886
3655	ENSG00000231995.2	RPL7AP76		-1.012909447	0.001866237	0.004637768
3656	ENSG00000188784.4	PLA2G2E	30814	-2.046306098	0.001866888	0.004639023
3657	ENSG00000284646.1			-1.11502945	0.00187756	0.004663789
3658	ENSG00000258414.1			-1.013772986	0.00188352	0.004676834
3659	ENSG00000202000.1	RNU1-36P		-1.708989882	0.001949541	0.004825957
3660	ENSG00000255858.3			-1.134693139	0.001971051	0.004873422
3661	ENSG00000066032.18	CTNNA2	1496	-1.278443477	0.001980996	0.004894958
3662	ENSG00000281560.1	LSINCT5	101234261	-1.199630463	0.001981811	0.004896362
3663	ENSG00000204091.8	TDRG1	732253	-1.415378893	0.001987436	0.004907813
3664	ENSG00000273727.1	U1	124904631	-1.416276457	0.002012158	0.004964223
3665	ENSG00000279134.1		105369784	-1.349415745	0.002021216	0.004984711
3666	ENSG00000287237.1			-1.137021536	0.002022954	0.004988686
3667	ENSG00000226272.5	ARHGAP26-AS1	100874239	-1.254138504	0.002035201	0.005016797
3668	ENSG00000248965.1			-1.543035212	0.00208842	0.005133207
3669	ENSG00000125879.5	OTOR	56914	-2.0381729	0.002105204	0.005170247
3670	ENSG00000276842.1			-1.121511503	0.002148039	0.005266362
3671	ENSG00000231980.1			-2.541703537	0.002158518	0.005289721
3672	ENSG00000178162.8	FAR2P2		-1.212413032	0.0021808	0.005337769
3673	ENSG00000264589.4	MAPT-AS1		-1.106383393	0.002203825	0.005389801
3674	ENSG00000145242.13	EPHA5	2044	-1.326382735	0.002216222	0.005416444
3675	ENSG00000281008.1			-1.399441632	0.002219545	0.005423563
3676	ENSG00000248517.1	LINC02437		-1.394822219	0.002238219	0.005464817
3677	ENSG00000286691.1			-1.293001318	0.002240346	0.005469672
3678	ENSG00000279609.1			-1.288744839	0.002252528	0.005495354
3679	ENSG00000277863.1			-1.502444674	0.002292304	0.005585176
3680	ENSG00000152092.16	ASTN1	460	-1.274611777	0.00229297	0.005586455
3681	ENSG00000235321.1			-1.226229555	0.002318667	0.005643512
3682	ENSG00000262920.5			-1.47608385	0.002339401	0.005691532
3683	ENSG00000226872.2			-1.097451285	0.002349892	0.00571355
3684	ENSG00000248461.3			-1.520906162	0.002361806	0.005739702
3685	ENSG00000242366.3	UGT1A8	54576	-2.056350646	0.00236941	0.005756771
3686	ENSG00000240095.1			-1.766065274	0.002391615	0.005805027
3687	ENSG00000235280.2	MCF2L-AS1		-1.156528868	0.002395199	0.005812661
3688	ENSG00000287652.1			-1.171550749	0.002413519	0.005853178
3689	ENSG00000105707.14	HPN	3249	-1.165512154	0.002417228	0.005861099
3690	ENSG00000266441.1	ARHGAP28-AS1		-1.235828035	0.002438151	0.005909301
3691	ENSG00000285766.1			-1.133313308	0.002446677	0.005928516
3692	ENSG00000259473.1	LINC02205	105370880	-1.267549913	0.002447982	0.00593059
3693	ENSG00000268081.1			-1.159137073	0.002462973	0.005962536
3694	ENSG00000258888.1			-1.32004918	0.002469456	0.005976041
3695	ENSG00000223394.2	TRBV29OR9-2		-1.230379597	0.002472263	0.005982104
3696	ENSG00000213759.10	UGT2B11	10720	-2.698037175	0.002487941	0.006016735
3697	ENSG00000256347.1	OR8R1P		-1.051656796	0.002502791	0.006050064
3698	ENSG00000163046.15	ANKRD30BL	554226	-1.624515239	0.002511718	0.006070164
3699	ENSG00000053328.8	METTL24	728464	-1.011784897	0.002541968	0.006135043
3700	ENSG00000187398.12	LUZP2	338645	-1.0290305	0.002545553	0.006142948
3701	ENSG00000253181.2			-1.844413719	0.002568257	0.006193214
3702	ENSG00000146399.2	TAAR1	134864	-2.179288914	0.002665048	0.006407136
3703	ENSG00000267868.1			-1.298357317	0.002680873	0.006442448
3704	ENSG00000251613.4			-1.404034636	0.002696823	0.006476459
3705	ENSG00000263096.1			-1.165022707	0.002742486	0.006578947
3706	ENSG00000196859.8	KRT39	390792	-1.280149404	0.002754209	0.006604272
3707	ENSG00000235782.1	PTGER3-AS1		-1.552818441	0.002758346	0.006612992
3708	ENSG00000100987.15	VSX1	30813	-1.171564776	0.002767301	0.006632857
3709	ENSG00000181617.6	FDCSP	260436	-1.684606147	0.00279122	0.006686549
3710	ENSG00000285562.1			-1.855557788	0.002792409	0.00668859
3711	ENSG00000235268.2	KDM4E	390245	-1.218171911	0.002844211	0.006804038
3712	ENSG00000196805.7	SPRR2B	6701	-1.28315323	0.002856656	0.006831749
3713	ENSG00000286400.1			-1.092997988	0.002925134	0.006981198
3714	ENSG00000183036.11	PCP4	5121	-1.763774033	0.002925605	0.006981901
3715	ENSG00000261115.6	TMEM178B	100507421	-1.060904157	0.002928474	0.006988328
3716	ENSG00000248371.5			-1.411424935	0.002932572	0.006996843
3717	ENSG00000260802.1	SERTM2	401613	-1.108297125	0.002964556	0.007068901
3718	ENSG00000231324.1		105369308	-1.469759871	0.002966762	0.007073736
3719	ENSG00000275805.1			-1.23161796	0.002985985	0.007114436
3720	ENSG00000269345.1	VN1R85P		-1.454006859	0.00299503	0.007134271
3721	ENSG00000231156.1			-1.102681955	0.003015367	0.007176248
3722	ENSG00000204930.10	FAM221B	392307	-1.127573795	0.00301652	0.007178561
3723	ENSG00000224721.1	FLVCR2-AS1	102724153	-1.054322317	0.003018058	0.007181789
3724	ENSG00000280155.1			-1.935138655	0.003038915	0.007227083
3725	ENSG00000243480.7	AMY2A	279	-1.61364431	0.003121112	0.007402138
3726	ENSG00000203785.9	SPRR2E	6704	-1.091092522	0.003158322	0.007481884
3727	ENSG00000235077.1			-1.058722875	0.003159338	0.007483844
3728	ENSG00000224163.4			-1.637389153	0.00317745	0.007520458
3729	ENSG00000280913.2	CTSLP3		-1.097092226	0.003326384	0.007846262
3730	ENSG00000260846.2	FRG2HP		-2.025941333	0.003352527	0.007903695
3731	ENSG00000099721.14	AMELY	266	-2.092684403	0.003367098	0.007932857
3732	ENSG00000178125.14	PPP1R42	286187	-1.245314086	0.003385018	0.007970815
3733	ENSG00000198597.9	ZNF536	9745	-1.033599267	0.003415969	0.008037967
3734	ENSG00000174453.10	VWC2L	402117	-1.326481568	0.003460783	0.008132316
3735	ENSG00000287650.1			-1.273857515	0.003519975	0.008262594
3736	ENSG00000277748.1			-1.270721854	0.003541723	0.008308725
3737	ENSG00000244122.2	UGT1A7	54577	-1.498515201	0.003561799	0.008349895
3738	ENSG00000258676.5			-1.283395685	0.003621633	0.008479634
3739	ENSG00000214252.4	AZGP1P2		-1.977022317	0.003714958	0.008677136
3740	ENSG00000152254.11	G6PC2	57818	-1.813057474	0.003742215	0.008732569
3741	ENSG00000286736.1			-1.286888624	0.0037574	0.008764908
3742	ENSG00000251008.1	LTO1P1		-1.131020338	0.00379672	0.008846849
3743	ENSG00000212939.2			-1.174283106	0.00398579	0.009240912
3744	ENSG00000225588.2			-1.576217572	0.004009523	0.009290277
3745	ENSG00000253647.1	KCNIP1-OT1	101928033	-1.395461258	0.004014539	0.009300172
3746	ENSG00000288097.1			-1.611604083	0.004034506	0.009339335
3747	ENSG00000287903.1			-1.301266614	0.004145623	0.009560275
3748	ENSG00000272555.1			-1.009784893	0.004164965	0.009602646
3749	ENSG00000285930.1			-1.676604678	0.004236844	0.009753056
3750	ENSG00000225315.2			-1.234932115	0.00424761	0.009776135
3751	ENSG00000230118.1			-1.493570906	0.00427542	0.009835004
3752	ENSG00000285630.1		105376479	-1.806783573	0.004313735	0.009913939
3753	ENSG00000145864.14	GABRB2	2561	-1.067503349	0.004322503	0.009931212
3754	ENSG00000244137.1	CAPN9-AS1		-1.022317926	0.004323175	0.009931604
3755	ENSG00000047457.14	CP	1356	-1.130487589	0.004333052	0.009953141
3756	ENSG00000235296.1			-1.141513373	0.004415905	0.010118251
3757	ENSG00000185303.17	SFTPA2	729238	-1.366276188	0.004443754	0.010174419
3758	ENSG00000179751.7	SYCN	342898	-1.175119202	0.004481307	0.010253295
3759	ENSG00000124812.15	CRISP1	167	-1.701899992	0.004516925	0.010327637
3760	ENSG00000255606.1	LINC02952		-1.254914569	0.00452125	0.010335739
3761	ENSG00000269356.1			-1.036159746	0.004593802	0.010484669
3762	ENSG00000197826.12	CFAP299	255119	-1.290028675	0.004598867	0.010493207
3763	ENSG00000232400.1	RAD17P1		-1.390445345	0.00469943	0.010701724
3764	ENSG00000221880.4	KRTAP1-3	81850	-2.151205538	0.004703658	0.010710735
3765	ENSG00000224784.2	S100A15A		-1.209994953	0.00474784	0.010800798
3766	ENSG00000236164.1			-2.502522418	0.004955633	0.011224558
3767	ENSG00000242173.10	ARHGDIG	398	-1.064784826	0.004992153	0.011297873
3768	ENSG00000253434.8	LINC02237	105375706	-1.804579293	0.005000352	0.011313567
3769	ENSG00000285329.1			-1.108092288	0.005003616	0.01131966
3770	ENSG00000172538.7	FAM170B	170370	-1.054260877	0.005115884	0.011550577
3771	ENSG00000198797.7	BRINP2	57795	-1.112887222	0.005174828	0.011667715
3772	ENSG00000250803.6	ZNF475	100505841	-1.008039168	0.00518387	0.011686107
3773	ENSG00000262855.1			-1.921469906	0.005191468	0.011701905
3774	ENSG00000229352.1			-1.258648395	0.005202598	0.01172033
3775	ENSG00000260035.1			-1.395897742	0.005207306	0.011729603
3776	ENSG00000287157.1			-1.502060255	0.005217756	0.011751138
3777	ENSG00000180440.4	SERTM1	400120	-1.570252617	0.005224716	0.01176481
3778	ENSG00000187848.15	P2RX2	22953	-1.064000326	0.005245147	0.011806793
3779	ENSG00000162494.6	LRRC38	126755	-1.011093327	0.00525861	0.011832395
3780	ENSG00000196228.3	SULT1C3	442038	-1.870789426	0.005310126	0.011934088
3781	ENSG00000248332.6	TUB-AS1	101927917	-2.224018273	0.00532277	0.011959432
3782	ENSG00000276128.2			-1.171388426	0.005400185	0.012116571
3783	ENSG00000182021.10			-1.226343781	0.005468616	0.012249317
3784	ENSG00000204880.8	KRTAP4-8	728224	-1.857950408	0.00546956	0.012250739
3785	ENSG00000247416.4	NCAM1-AS2	101928847	-1.010187443	0.005479557	0.012270583
3786	ENSG00000253525.1	CPP		-1.293389698	0.005574845	0.012464025
3787	ENSG00000286944.1			-1.502889188	0.00572021	0.012754505
3788	ENSG00000286514.1		105376419	-1.430548616	0.005741321	0.01279654
3789	ENSG00000151360.10	ALLC	55821	-1.057822948	0.005760653	0.012836594
3790	ENSG00000287970.1			-1.344789194	0.005793746	0.012905406
3791	ENSG00000282033.1			-1.005462603	0.005902354	0.013123735
3792	ENSG00000165186.12	PTCHD1	139411	-1.001278296	0.00598292	0.013279784
3793	ENSG00000267478.1			-1.0722006	0.005985756	0.013283848
3794	ENSG00000224318.6	CHL1-AS2		-1.181863594	0.006117401	0.013544918
3795	ENSG00000196711.9	ALKAL1	389658	-1.454647595	0.00612965	0.013570523
3796	ENSG00000251226.1	LINC02714	729305	-1.366997794	0.00615114	0.01361278
3797	ENSG00000224565.2	LINC01754		-1.22678941	0.006176556	0.013663687
3798	ENSG00000237336.1	DENND1A-AS1		-1.684483989	0.006249474	0.013799106
3799	ENSG00000236969.2	GGT8P		-1.12062369	0.006267411	0.013835342
3800	ENSG00000213215.5	OR2F1	26211	-1.792382085	0.006390168	0.014075764
3801	ENSG00000264232.2	LINC01916	107985128	-3.854276146	0.006402131	0.014098983
3802	ENSG00000256925.3	ADGRA1-AS1		-1.662872809	0.006411835	0.014119569
3803	ENSG00000246016.2			-1.329284968	0.006523694	0.014341203
3804	ENSG00000280165.1	PCDH20	64881	-1.838934265	0.006528337	0.014348228
3805	ENSG00000237412.7	PRSS56	646960	-1.387208158	0.006546637	0.014384629
3806	ENSG00000122180.5	MYOG	4656	-1.549317473	0.006653553	0.014593502
3807	ENSG00000277493.1			-1.158823877	0.006731536	0.014744968
3808	ENSG00000184999.12	SLC22A10	387775	-1.324313383	0.006771042	0.01482086
3809	ENSG00000242902.1	FLNC-AS1	110806300	-1.399146264	0.006848043	0.014968744
3810	ENSG00000259725.1			-1.748355582	0.006951169	0.015168239
3811	ENSG00000285536.1	LINC02838		-1.415453599	0.007118001	0.015499418
3812	ENSG00000186860.5	KRTAP17-1	83902	-1.383318498	0.007245651	0.01575325
3813	ENSG00000255093.2			-1.139264173	0.007378577	0.01600926
3814	ENSG00000187272.7	KRTAP9-8	83901	-2.07771407	0.007404499	0.016058472
3815	ENSG00000259664.3			-1.051566785	0.007426736	0.016099653
3816	ENSG00000259541.5	POU2F3-AS1	649133	-1.156165683	0.007485419	0.016203832
3817	ENSG00000254675.1		124902723	-1.245024907	0.007632911	0.016486207
3818	ENSG00000244025.5	KRTAP19-3	337970	-2.444458936	0.007781824	0.016778593
3819	ENSG00000214510.10	SPINK13	153218	-1.316902459	0.007783567	0.016781437
3820	ENSG00000249885.1	ARHGEF38-IT1	100874374	-1.835776034	0.00793369	0.017074464
3821	ENSG00000134115.13	CNTN6	27255	-1.085088145	0.008087158	0.017365162
3822	ENSG00000214415.4	GNAT3	346562	-1.762568271	0.008176383	0.017538703
3823	ENSG00000172900.12	ACTE1P		-1.10803004	0.008305419	0.017787585
3824	ENSG00000258647.6	LINC00930		-1.006820809	0.008507379	0.018185738
3825	ENSG00000285683.2			-1.607289163	0.008517042	0.018203449
3826	ENSG00000254447.3			-1.259943896	0.008550006	0.018266811
3827	ENSG00000163630.11	SYNPR	132204	-1.338006571	0.008663152	0.018481858
3828	ENSG00000278912.1			-1.59471612	0.008707089	0.0185676
3829	ENSG00000282904.2		107986277	-1.166240774	0.00893459	0.018982294
3830	ENSG00000180332.6	KCTD4	386618	-1.098948425	0.008958598	0.019027182
3831	ENSG00000234406.1			-1.790473485	0.009034806	0.019163378
3832	ENSG00000255872.3			-1.368212886	0.009109398	0.019308163
3833	ENSG00000256779.2	SRSF8BP		-1.273142733	0.009236659	0.019556995
3834	ENSG00000284808.1			-1.23409718	0.009262624	0.019606846
3835	ENSG00000287831.1			-1.427725958	0.009293335	0.019663346
3836	ENSG00000235875.3	ARHGEF7-AS2	100874238	-1.028807098	0.009606963	0.020269609
3837	ENSG00000148604.15	RGR	5995	-1.397706232	0.009618553	0.020290822
3838	ENSG00000225833.1	ACE2-DT	104798195	-1.273582307	0.009740028	0.020511068
3839	ENSG00000268278.1			-1.607163969	0.009755456	0.020538102
3840	ENSG00000168412.6	MTNR1A	4543	-1.138615698	0.0097931	0.020611882
3841	ENSG00000225354.1	RPL7AP52		-1.227303241	0.009939312	0.020879717
3842	ENSG00000267192.1			-1.014737455	0.009972471	0.020941608
3843	ENSG00000143125.6	PROK1	84432	-1.100532723	0.009973631	0.020942935
3844	ENSG00000286980.1			-1.72297646	0.010332066	0.021615455
3845	ENSG00000196104.11	SPOCK3	50859	-1.059528606	0.010377818	0.02169972
3846	ENSG00000240093.1			-1.008976552	0.010720694	0.022345957
3847	ENSG00000249421.2	ADAMTS19-AS1		-1.531622067	0.010805697	0.0225113
3848	ENSG00000137561.5	TTPA	7274	-1.046715224	0.010873315	0.022638461
3849	ENSG00000186910.4	SERPINA11	256394	-1.002441559	0.011054006	0.022973073
3850	ENSG00000198739.11	LRRTM3	347731	-1.523190353	0.011102927	0.023068697
3851	ENSG00000197360.9	ZNF98	148198	-1.013528392	0.011291082	0.023415455
3852	ENSG00000225126.1	CAMTA1-AS1		-1.037346156	0.011318897	0.023456628
3853	ENSG00000256513.1			-1.892273819	0.011319218	0.023456628
3854	ENSG00000092377.15	TBL1Y	90665	-1.331282491	0.011393918	0.023590484
3855	ENSG00000249776.5			-1.436939044	0.011438138	0.023672134
3856	ENSG00000101203.16	COL20A1	57642	-1.013299393	0.011539287	0.023854058
3857	ENSG00000286154.1			-1.110415013	0.011561677	0.023892887
3858	ENSG00000187833.7	C2orf78	388960	-1.228533579	0.011743277	0.024223985
3859	ENSG00000248663.6	LINC00992	124900192	-1.083932039	0.011815377	0.024354987
3860	ENSG00000140506.17	LMAN1L	79748	-1.474552569	0.011851578	0.024416899
3861	ENSG00000258699.1			-1.064720874	0.012146811	0.024946074
3862	ENSG00000267361.1	SEC24AP1		-1.035684205	0.01221545	0.02506886
3863	ENSG00000288057.1			-1.953119833	0.012338935	0.025298709
3864	ENSG00000271824.2	SMIM32	389332	-1.018575923	0.012430056	0.025465782
3865	ENSG00000234422.1	UBE2WP1		-1.266131881	0.012582508	0.02574537
3866	ENSG00000230945.1	LINC01507		-1.225341162	0.012627157	0.025832214
3867	ENSG00000183566.10	BPIFA4P		-2.360050473	0.012841884	0.026216035
3868	ENSG00000251491.2	OR7E28P		-1.108346908	0.013089462	0.026666533
3869	ENSG00000182898.4	TCHHL1	126637	-1.043573379	0.013384262	0.027204213
3870	ENSG00000259663.2	ISL1-DT	642366	-1.077863973	0.013394026	0.027222665
3871	ENSG00000286117.2			-1.046861825	0.013423448	0.027276869
3872	ENSG00000183888.4	SRARP	149563	-1.430661653	0.01352006	0.027449273
3873	ENSG00000228528.2			-1.539739327	0.013699004	0.027768497
3874	ENSG00000258601.1			-2.032024595	0.013724259	0.027809738
3875	ENSG00000284672.1			-1.453455815	0.013774265	0.027896958
3876	ENSG00000233952.1	FTLP15		-1.010414938	0.013915252	0.028163497
3877	ENSG00000233766.7	CAVIN2-AS1		-1.13404174	0.013940663	0.028212196
3878	ENSG00000269354.1			-1.740788017	0.014065418	0.028431291
3879	ENSG00000182333.15	LIPF	8513	-1.333847513	0.014248819	0.028763883
3880	ENSG00000225473.1	ATP13A4-AS1		-1.226401782	0.014351733	0.0289454
3881	ENSG00000227279.1	CDH2-AS1		-1.308637801	0.014435858	0.029091097
3882	ENSG00000256618.2			-1.037100996	0.014779075	0.029705746
3883	ENSG00000118526.7	TCF21	6943	-1.206505457	0.014817145	0.029771702
3884	ENSG00000249231.8	CASC16	643714	-1.421875129	0.015021263	0.030132966
3885	ENSG00000106341.11	PPP1R17	10842	-1.276118978	0.015194889	0.030451989
3886	ENSG00000212657.1	KRTAP16-1	100505753	-1.142369945	0.015393658	0.030806741
3887	ENSG00000250360.1			-1.21186728	0.015412489	0.030841313
3888	ENSG00000250882.1			-1.113600772	0.016124916	0.032104891
3889	ENSG00000255595.5	LINC02350		-1.290560442	0.016189981	0.032219874
3890	ENSG00000230804.2	CDRT15P14		-1.33776068	0.016477321	0.032714537
3891	ENSG00000279460.1			-1.64216124	0.016545285	0.032824819
3892	ENSG00000273906.1			-1.351327793	0.016772225	0.033235142
3893	ENSG00000110680.13	CALCA	796	-1.205961002	0.01690527	0.033465328
3894	ENSG00000151418.12	ATP6V1G3	127124	-2.192628073	0.017087472	0.033780474
3895	ENSG00000224810.1			-1.173136096	0.017100688	0.033802134
3896	ENSG00000083622.8			-1.69135788	0.017669821	0.034787763
3897	ENSG00000174562.13	KLK15	55554	-1.011963267	0.01770014	0.034840933
3898	ENSG00000272710.2			-1.311979108	0.017757029	0.034942103
3899	ENSG00000243955.6	GSTA1	2938	-1.343642595	0.017788598	0.034992067
3900	ENSG00000219159.4			-1.172979893	0.018027107	0.03539977
3901	ENSG00000284471.1			-1.029376741	0.018157582	0.035629515
3902	ENSG00000267428.2	DYNAPP2		-2.005830543	0.018251153	0.03579895
3903	ENSG00000021852.13	C8B	732	-1.23961421	0.018978098	0.037063473
3904	ENSG00000156096.14	UGT2B4	7363	-1.300251203	0.019097911	0.037266256
3905	ENSG00000187658.7	C5orf52	100190949	-1.00275346	0.019320581	0.037619295
3906	ENSG00000224361.1			-1.161455031	0.019460041	0.037861091
3907	ENSG00000287272.1			-1.48297738	0.019500644	0.03793078
3908	ENSG00000105694.3	ELOCP28		-1.039228933	0.020232331	0.039185978
3909	ENSG00000278484.1			-1.642073158	0.021050347	0.040537434
3910	ENSG00000207797.1	MIR187	406963	-1.087078596	0.021421337	0.04117387
3911	ENSG00000267275.1	KLF2-DT		-1.127678415	0.021776887	0.041786372
3912	ENSG00000236013.7	LINC02941	100507477	-1.519723241	0.022171784	0.042470122
3913	ENSG00000263649.1	MIR3135B	100616218	-1.20947659	0.022539599	0.043074784
3914	ENSG00000258455.1			-1.044412447	0.023445181	0.044575524
3915	ENSG00000181123.8		107985579	-1.132696792	0.023679589	0.044975906
3916	ENSG00000283329.1	FAM240B	110806297	-1.465143699	0.023702888	0.045009377
3917	ENSG00000260057.6	LINC01571		-2.006548693	0.024116273	0.045684954
3918	ENSG00000120251.21	GRIA2	2891	-1.030027371	0.024147384	0.045732154
3919	ENSG00000121871.4	SLITRK3	22865	-1.059174745	0.024967209	0.047058607
3920	ENSG00000287101.1			-1.012062642	0.025455034	0.047852261
3921	ENSG00000250366.3	TUNAR	100507043	-1.345035012	0.025582276	0.048048872
3922	ENSG00000278464.2		105372061	-1.369593708	0.025641398	0.048148511
3923	ENSG00000236511.2	LINC01231		-1.20253179	0.025792307	0.048397501
3924	ENSG00000170788.14	DYDC1	143241	-1.288072567	0.025866979	0.048521543
3925	ENSG00000257582.5	LINC01475		-1.213178421	0.02597097	0.048702786
3926	ENSG00000111049.4	MYF5	4617	-1.258536488	0.026312505	0.049271017
3927	ENSG00000183733.6	FIGLA	344018	-1.217379982	0.026509519	0.04960011
3928	ENSG00000199080.1	MIR133B	442890	-1.537973803	0.026868833	0.050187136
3929	ENSG00000259961.1		107984918	-1.347649131	0.026901658	0.050241349
3930	ENSG00000124233.12	SEMG1	6406	-1.362868005	0.02731376	0.050886348
3931	ENSG00000232721.2			-1.071692124	0.027576458	0.051315482
3932	ENSG00000226992.1			-1.251865285	0.027696916	0.051510626
3933	ENSG00000261206.1	LINC00561	100861545	-1.585796139	0.027771669	0.051627855
3934	ENSG00000287881.1			-1.152873202	0.027813732	0.051693792
3935	ENSG00000267224.1			-1.016978058	0.027994903	0.051979485
3936	ENSG00000101323.5	HAO1	54363	-1.10616604	0.029193061	0.053903771
3937	ENSG00000206177.7	HBM	3042	-1.356747818	0.029626165	0.054599229
3938	ENSG00000233569.2		101928797	-1.473328164	0.030462967	0.055951554
3939	ENSG00000256847.1	CCND2P1		-1.124767902	0.030597641	0.056180692
3940	ENSG00000277350.2			-1.59805623	0.030692149	0.056328134
3941	ENSG00000266232.1	MIR3178	100422974	-1.030769679	0.030710214	0.056352553
3942	ENSG00000278685.4	IQCA1L	392843	-1.087282184	0.031147408	0.057047468
3943	ENSG00000228476.2	CSTP1		-1.472992929	0.031164773	0.057071364
3944	ENSG00000233643.2	LINC02625		-1.029066198	0.031761786	0.058041344
3945	ENSG00000285587.1		107984273	-1.286995571	0.032933988	0.059882587
3946	ENSG00000279940.1			-1.222125392	0.033051396	0.06006026
3947	ENSG00000264564.1	DYNAPP1		-1.833502561	0.033112575	0.060152134
3948	ENSG00000256650.1	RERG-IT1		-1.089246312	0.033279932	0.060406337
3949	ENSG00000214216.11	IQCJ	654502	-1.294711465	0.034049341	0.061642041
3950	ENSG00000275358.1		124904882	-1.12704443	0.034948974	0.063049044
3951	ENSG00000180988.2	OR52N2	390077	-1.035715193	0.035628743	0.064080019
3952	ENSG00000180116.15	REDIC1	283461	-1.140681763	0.035640044	0.064097438
3953	ENSG00000159289.6	GOLGA6A	342096	-1.016924641	0.036048298	0.064749432
3954	ENSG00000225330.1			-1.209818303	0.036123472	0.06486389
3955	ENSG00000009709.12	PAX7	5081	-1.092389018	0.036705245	0.065770573
3956	ENSG00000286022.1			-1.419910866	0.037690867	0.067279183
3957	ENSG00000227729.5	RD3L	647286	-1.635243201	0.038066059	0.067878623
3958	ENSG00000175497.17	DPP10	57628	-1.265311159	0.038338258	0.068305634
3959	ENSG00000251081.1		102725220	-1.446781525	0.038368621	0.068347446
3960	ENSG00000156076.10	WIF1	11197	-1.255636834	0.038479678	0.06853296
3961	ENSG00000284614.1			-1.080043802	0.038970894	0.06928955
3962	ENSG00000170419.11	VSTM2A	222008	-1.045162878	0.039141548	0.069552423
3963	ENSG00000185002.10	RFX6	222546	-1.018832841	0.039190939	0.069618348
3964	ENSG00000242104.5	LRRC78P		-1.126923437	0.03919557	0.069623456
3965	ENSG00000225249.6	LINC00378		-1.045870688	0.039376885	0.069914205
3966	ENSG00000248399.1			-1.150191235	0.040144826	0.071078468
3967	ENSG00000235743.2			-1.668802555	0.040234798	0.071219022
3968	ENSG00000280309.1			-1.289552993	0.041711723	0.073487141
3969	ENSG00000268997.1	DNAJC19P3		-1.491191012	0.043591097	0.076370616
3970	ENSG00000253619.1			-1.062338842	0.04376992	0.07665004
3971	ENSG00000277749.1	CCDC33-AS1	124903525	-1.139119635	0.04392055	0.076876475
3972	ENSG00000249158.7	PCDHA11	56138	-1.175007131	0.044615313	0.077917127
3973	ENSG00000240871.5	KRTAP4-7	100132476	-1.94524204	0.044734315	0.078090558
3974	ENSG00000179073.6	TAAR3P		-1.456311056	0.045936091	0.079912934
3975	ENSG00000258175.1	LINC02300		-1.268543651	0.045987205	0.079984433
3976	ENSG00000164326.5	CARTPT	9607	-1.160322499	0.04599058	0.079984433
3977	ENSG00000249621.1	SNCAIP-AS2	139281641	-2.065645104	0.046025539	0.080034499
3978	ENSG00000254586.2			-1.456144925	0.048892239	0.084364539
3979	ENSG00000228648.1			-3.192169575	0.000300647	NA
3980	ENSG00000224599.1	BMS1P12		-2.244880426	0.000353405	NA
3981	ENSG00000207922.1			-1.66192624	0.000369392	NA
3982	ENSG00000223373.1			-1.691516537	0.000732563	NA
3983	ENSG00000231701.1	BMS1P13		-2.165756575	0.000762841	NA
3984	ENSG00000273124.1		124902479	-2.613535703	0.000980414	NA
3985	ENSG00000228587.1			-2.696339448	0.001272517	NA
3986	ENSG00000251325.1			-2.13664818	0.001517424	NA
3987	ENSG00000265296.1	FEM1AP2		-1.489381304	0.001778542	NA
3988	ENSG00000222182.1	RNA5SP156		-1.933587052	0.002150784	NA
3989	ENSG00000286530.1			-2.556231937	0.002661593	NA
3990	ENSG00000258112.1	CCNG2P1		-2.516703597	0.002714707	NA
3991	ENSG00000254529.1	MRGPRX7P		-2.949355503	0.002899793	NA
3992	ENSG00000235933.1	LINC01779		-1.878580809	0.003679186	NA
3993	ENSG00000255480.1			-1.87252931	0.004106931	NA
3994	ENSG00000258133.1			-2.302602088	0.00441567	NA
3995	ENSG00000286716.1			-2.256632063	0.004699909	NA
3996	ENSG00000287018.1			-2.048123473	0.005618546	NA
3997	ENSG00000236576.1			-1.670518882	0.005684677	NA
3998	ENSG00000241159.3	RN7SL160P		-1.670628714	0.007128022	NA
3999	ENSG00000254089.2			-2.188897117	0.007178564	NA
4000	ENSG00000275676.1			-2.30697901	0.009526544	NA
4001	ENSG00000255494.1	LINC00681	101409254	-3.729367513	0.00980821	NA
4002	ENSG00000232072.1			-2.432742948	0.009875168	NA
4003	ENSG00000226497.1	LINC02549	101928280	-3.04058774	0.00999571	NA
4004	ENSG00000236495.2		105376429	-2.117286098	0.011203771	NA
4005	ENSG00000254372.1			-1.307975731	0.011366448	NA
4006	ENSG00000272519.1			-1.599631106	0.011973778	NA
4007	ENSG00000242034.1			-1.858332299	0.012003466	NA
4008	ENSG00000249710.1	DDIT4L-AS1	101929353	-2.463579214	0.012509372	NA
4009	ENSG00000213244.3	H3P4		-1.18482785	0.01282547	NA
4010	ENSG00000205025.8	OR5G5P		-2.268678642	0.013031059	NA
4011	ENSG00000226594.1			-1.524477963	0.013124788	NA
4012	ENSG00000255660.1	RERG-AS1	100873980	-2.601982256	0.014780109	NA
4013	ENSG00000223721.3	UQCRFS1P2		-1.773102151	0.020960326	NA
4014	ENSG00000248528.1	LINC02058		-2.289783034	0.021431608	NA
4015	ENSG00000286700.1			-2.128850163	0.02148557	NA
4016	ENSG00000227947.2	LINC01738	107984953	-1.63123475	0.02150185	NA
4017	ENSG00000271360.1	USP6NL-AS1	107984208	-1.338326429	0.022016259	NA
4018	ENSG00000250456.1	LINC02260	339978	-2.22721196	0.022420008	NA
4019	ENSG00000260166.2			-1.231685646	0.022653645	NA
4020	ENSG00000267373.1			-1.065385091	0.023450402	NA
4021	ENSG00000230525.6			-2.062157144	0.024525128	NA
4022	ENSG00000275944.1			-1.937485134	0.02472601	NA
4023	ENSG00000198083.9	KRTAP9-9	81870	-2.332837328	0.025581725	NA
4024	ENSG00000227293.1			-2.01435511	0.025683818	NA
4025	ENSG00000276489.1	MIR6829	102465977	-1.310134232	0.025750798	NA
4026	ENSG00000228771.1		107985659	-2.130212653	0.026318276	NA
4027	ENSG00000249752.1			-1.997717146	0.026490304	NA
4028	ENSG00000269736.1			-1.203301861	0.027994935	NA
4029	ENSG00000276531.1	SNX19P4		-1.381305294	0.033147051	NA
4030	ENSG00000259561.1			-1.453821776	0.033955445	NA
4031	ENSG00000286794.1		105373299	-2.837823501	0.034280192	NA
4032	ENSG00000271499.1			-1.186599386	0.035946729	NA
4033	ENSG00000184945.14	AQP12A	375318	-1.541491217	0.036127437	NA
4034	ENSG00000126545.14	CSN1S1	1446	-1.508711963	0.036976745	NA
4035	ENSG00000250692.1			-2.169529197	0.037733296	NA
4036	ENSG00000234723.1	FCRL4P1		-1.854240336	0.039872389	NA
4037	ENSG00000226337.3	TMEM252-DT	105376072	-1.576411711	0.040320765	NA
4038	ENSG00000254905.1			-1.032867402	0.041180398	NA
4039	ENSG00000257826.1			-2.281809439	0.042171504	NA
4040	ENSG00000254499.1			-1.217855169	0.042407019	NA
4041	ENSG00000204873.5	KRTAP9-3	83900	-2.074946027	0.044855361	NA
4042	ENSG00000205301.12	MGAT4D	152586	-1.356652633	0.046490228	NA
4043	ENSG00000263317.1	LINC01982	105371830	-1.758028556	0.046988351	NA
4044	ENSG00000287661.1			-1.397304652	0.049152112	NA
